# Association of body mass index and waist-to-height ratio with outcomes in ischemic stroke: results from the Third China National Stroke Registry

**DOI:** 10.1186/s12883-023-03165-y

**Published:** 2023-04-14

**Authors:** Xiaolin Li, Qin Xu, Anxin Wang, Pei Zheng, Huimin Zhu, Ai Guo, Xia Meng, Yong Jiang

**Affiliations:** 1 Department of Neurology, Beijing Tiantan Hospital, Capital Medical University, No.119 S Fourth Ring West Rd, Beijing, 100070 China; 2 China National Clinical Research Center for Neurological Diseases, Beijing Tiantan Hospital, Capital Medical University, Beijing, China; 3 Department of Neurology, Tianjin Neurological Institute, Tianjin Medical University General Hospital, Tianjin, China; 4 Advanced Innovation Center for Human Brain Protection, Capital Medical University, Beijing, China; 5 Research Unit of Artificial Intelligence in Cerebrovascular Disease (2019RU018), Chinese Academy of Medical Sciences, Beijing, China; 6 Center for Excellence in Brain Science and Intelligence Technology, Chinese Academy of Sciences, Shanghai, China

**Keywords:** Ischemic stroke, BMI, Waist-to-height ratio, Mortality, Abdominal obesity, General obesity

## Abstract

**Background and purpose:**

Conflicting reports of obesity paradox have led to confusion about weight management strategies for post-stroke patients. The main purpose of this study is to determine whether the obesity paradox measured by body mass index (BMI) or by waist-to-height ratio (WHtR) is real.

**Methods:**

We evaluated the association of general obesity measured by BMI, and abdominal obesity measured by WHtR with 1-year all-cause mortality, recurrence of stroke and combined vascular events of acute ischemic stroke (AIS) patients in a cohort —— the Third China National Stroke Registry (CNSR-III). Cox proportional hazards models and restricted cubic splines were performed to investigate the association between obesity and clinical outcomes.

**Results:**

A total of 14,146 patients with ischemic stroke were included. When BMI was used as a measure of obesity, compared to the normal weight patients, mortality decreased in overweight patients (hazard ratio [HR] 0.74 [95% confidence interval (CI) 0.61–0.91], *P* = 0.0035) and obese patients (HR 0.54 [0.40–0.73], *P* < 0.0001); and increased in underweight patients (HR 2.55 [1.75–3.73], *P *< 0.0001). After adjustment for confounding factors, the protective effect of obesity and overweight disappeared. BMI had no association with recurrence of stroke or combined vascular events. When WHtR was used as a measure of obesity, obese patients had lower 1-year all-cause mortality (HR 0.64 [0.43–0.97], *P* = 0.0357). After adjustment for confounding factors, this difference disappeared; overweight patients still had lower all-cause mortality (adjusted hazard ratio [aHR] 0.42 [0.26–0.67], *P* = 0.0003), recurrence of stroke (aHR 0.77 [0.60–0.99], *P* = 0.0440) and combined vascular events (aHR 0.75 [0.58–0.95], *P* = 0.0198).

**Conclusions:**

Among Chinese patients with AIS, our study does not support the BMI paradox; overweight patients measured by WHtR had a more favorable prognosis. TOAST subtypes did not modify the association.

**Supplementary Information:**

The online version contains supplementary material available at 10.1186/s12883-023-03165-y.

## Introduction

The prevalence of obesity among Chinese adults has been increasing since the beginning of this century. Obesity is recognized as a vital risk factor for ischemic stroke due to its remarkable regulatory effect on chronic inflammation and metabolic disorders [1]. However, many studies have shown that after stroke, obese and overweight patients had a marked survival advantage over individuals with normal weight or underweight [2-4], a phenomenon commonly known as the obesity paradox. Still, the obesity paradox is controversial. Two Asian population studies denied the obesity paradox [5, 6]. Etiological subtypes of stroke predicted risk of mortality independently of stroke severity and cardiovascular risk factors [7], and obesity is most closely associated with the risk of large-artery atherosclerosis stroke. The impact of etiological subtypes on the association between obesity and stroke prognosis was not considered in previous studies [2-4, 6]. A growing body of evidence implicates that visceral fat is more important than peripheral fat for regulating inflammation [8]. Body mass index (BMI) was used as a measure of obesity in most of the studies which does not reflect distribution of fat or total fat. Compared to BMI, markers of abdominal obesity showed a stronger association with risk of stroke [9]. In a heart failure cohort, obesity was associated with better outcomes only when defined by BMI, Whereas higher waist-to-height ratio (WHtR) conferred higher all-cause mortality [10]. However, the association between abdominal obesity and prognosis of ischemic stroke is still less well characterized.

The main purpose of this study is to determine whether the obesity paradox exists and whether the obesity paradox defined by BMI or by WHtR is consistent.

## Methods

### Study definitions

**Fig. 1:**
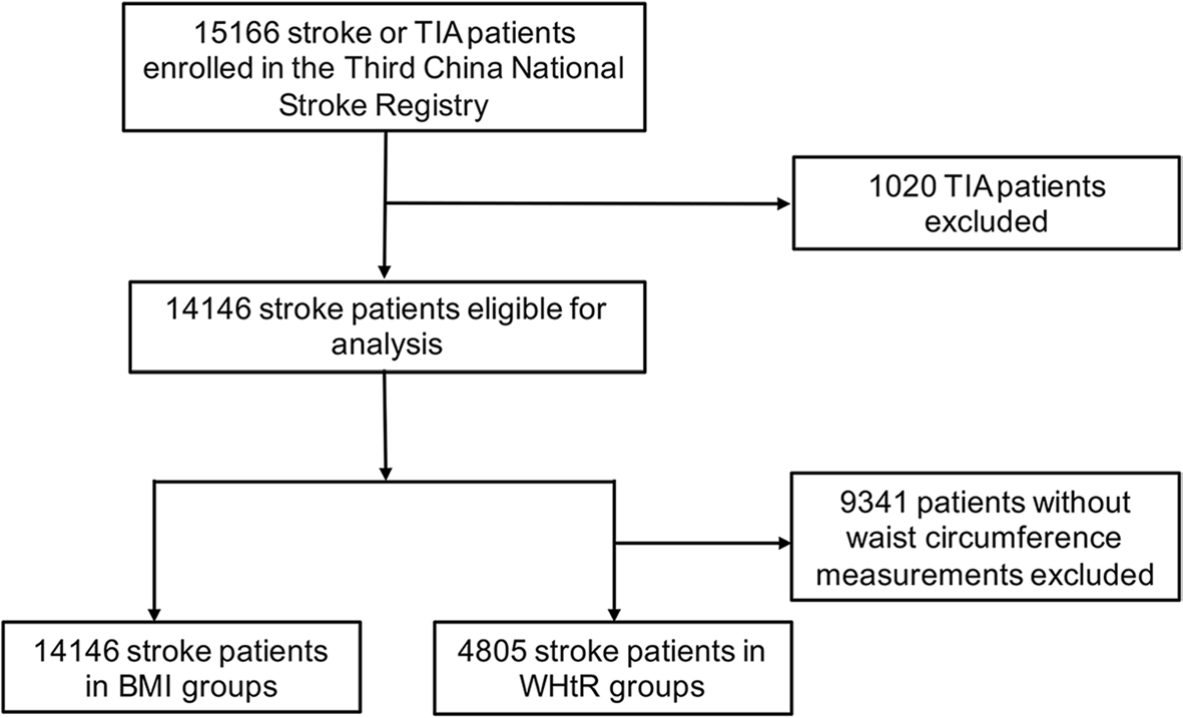
Flow chart of the study. TIA, transient ischemic attack; BMI, body mass index; WHtR, waist-to-height ratio

The Third China National Stroke Registry (CNSR-III) is a nationwide prospective registry of patients with 14,146 acute ischemic stroke (AIS) patients and 1020 transient ischemic attack (TIA) patients. Patients were recruited from a total of 201 hospitals all over the country from August 2015 to March 2018. In summary, AIS or TIA patients within 7 days after the onset of symptoms who were older than 18 years old were included in the registry. Patients who refused to participate in the study or without symptoms and signs were excluded. The design details of CNSR-III have been previously reported elsewhere [11]. The baseline data of participants were collected by well-trained researchers following a standard protocol. The study protocol was approved by the ethics committee at Beijing TianTan Hospital. Informed consent from patients or their legal representatives was obtained. BMI data were available for all the AIS patients (*n* = 14,146). Waist circumference was obtained in 4805 patients. Data on 14,146 patients with AIS from CNSR-III Registry were used in this study. The flow chart of our study is shown in Fig. 1.

### Grouping and outcome definitions

Patients were grouped based on (1) Asian cutoffs for BMI recommended by WHO [12]: (underweight: < 18.5 kg/ m^2^, normal weight: 18.5 to < 23.0 kg/ m^2^, overweight: 23.0 to < 27.5 kg/ m^2^, and obese: ≥ 27.5 kg/ m^2^), (2) WHtR quartiles [10] (< 0.47, 0.47 to < 0.52, 0.52 to < 0.57, ≥ 0.57 for underweight, normal weight, overweight, obese group respectively). The outcome was defined as 1-year all-cause mortality, recurrence of stroke and combined vascular events (including cardiovascular death, non-fatal stroke and non-fatal myocardial infarction). As a sensitivity analysis, the association of BMI recommended by the Chinese obesity working group [13] (< 18.5 kg/ m^2^, 18.5 to < 24 kg/ m^2^, 24 to < 28 kg/ m^2^, ≥ 28 kg/ m^2^ for underweight, normal weight, overweight, obese group respectively) and 1-year all-cause mortality was also analyzed.

### Statistical analysis

Differences in baseline variables among groups were performed using Kruskal–Wallis Test or non-parametric Wilcoxon for continuous variables and χ^2^ tests for categorical variables. P for linear trend was calculated by Kendall's Tau-b correlation analysis for continuous variables or Cochran-Armitage test for categorical variables. We performed univariate and multivariable Cox proportional hazards models to investigate the impact of obesity on the risk of 1-year all-cause mortality, recurrence of stroke and combined vascular events. After univariate analysis, independent variables were selected to be included in the multivariable model: variables with statistically significant differences in univariate analysis, and the P value was extended to 0.1; variables with no statistically significant differences in univariate analysis, but considered to be clinically related to the association between obesity and dependent variables. Variables adjusted in the multivariable analyses include age, gender, ethnicity, history of diabetes mellitus, history of atrial fibrillation, history of hypertension, history of myocardial infarction, history of lipid metabolism disorders, heavy drinking, smoking, intravenous thrombolysis, arterial thrombolysis or mechanical thrombectomy, National Institutes of Health Stroke Scale (NIHSS) at admission, Trial of Org 10172 in Acute Stroke Treatment (TOAST) subtypes. The interactions of BMI/WHtR with stroke subtype were investigated with the addition of BMI/WHtR by stroke subtype groups using multivariable Cox proportional hazards models. In addition, restricted cubic splines were used to show the shape of the associations between BMI/WHtR and clinical outcomes. Age-stratified and gender-stratified analyses of the association of BMI with all-cause mortality were also performed. *P *< 0.05 (two-tailed) was considered statistically significant. All statistical analyses were performed with SAS 9.4.

## Results

### Baseline characteristics

**Fig. 2:**
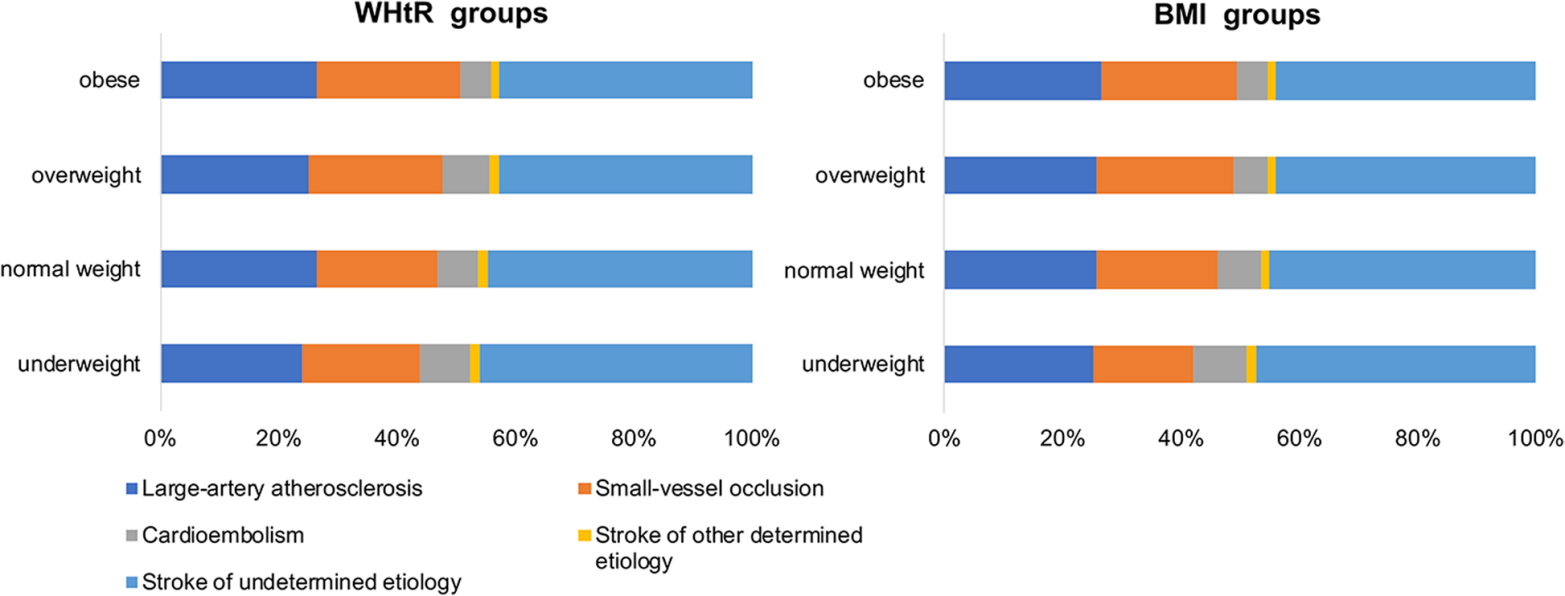
TOAST classification distribution in patients grouped by BMI or WHtR. BMI, body mass index; WHtR, waist-to-height ratio; TOAST, Trial of Org 10,172 in Acute Stroke Treatment

Information on BMI was available for the entire cohort with 14,146 AIS patients. Compared to underweight and normal weight patients, overweight and obese patients had larger waist measures, greater occurrence of diabetes, hypertension and lipid metabolism disorders, and had lower prevalence of atrial fibrillation, lower NIHSS scores at admission, much younger age (*P* < 0.0001, P_for linear trend_<0.0001) (Table 1). The proportion of cardioembolism in the low BMI group was significantly higher than that in the high BMI groups (*P* < 0.01) (Table 1, Fig. 2).

**Table 1:** Baseline characteristics by BMI groups

Characteristics	BMI groups, kg/m^2^				*P*-value	P _for linear trend_
	< 18.5	18.5- < 23	23- < 27.5	≥ 27.5		
N, %	309, 2.18%	3855, 27.25%	7491, 52.95%	2491, 17.61%		
Age (year), median (IQR)	72 (61–78)	65 (57–73)	62 (54–69)	60 (52–67)	< 0.0001	< 0.0001
Male, N (%)	177 (57.28%)	2599 (67.42%)	5303 (70.79%)	1641 (65.88%)	< 0.0001	0.2082
Ethnicity(Han), N (%)	299 (96.76%)	3776 (97.95%)	7268 (97.02%)	2387 (95.82%)	< 0.0001	< 0.0001
Stroke history, N (%)	80 (25.89%)	806 (20.91%)	1663 (22.20%)	585 (23.48%)	0.0361	0.1016
Medical comorbidities, N ( %)						
Diabetes mellitus	41 (13.27%)	760 (19.71%)	1829 (24.42%)	680 (27.30%)	< 0.0001	< 0.0001
Atrial fibrillation	42 (13.59%)	303 (7.86%)	489 (6.53%)	152 (6.10%)	< 0.0001	< 0.0001
Myocardial infarction	10 (3.24%)	69 (1.79%)	144 (1.92%)	55 (2.21%)	0.2572	0.6764
Hypertension	159 (51.46%)	2085 (54.09%)	4835 (64.54%)	1808 (72.58%)	< 0.0001	< 0.0001
Lipid metabolism disorders	16 (5.18%)	226 (5.86%)	572 (7.64%)	261 (10.48%)	< 0.0001	< 0.0001
Heavy drinking^a^, N ( %)	38 (12.30%)	552 (14.32%)	1075 (14.35%)	345 (13.85%)	0.7171	0.9632
Current smoker, N ( %)	99 (32.04%)	1210 (31.39%)	2411 (32.19%)	783 (31.43%)	0.8085	0.8819
Waist circumference (cm), median (IQR)^b^	72 (69–79)	80 (75–86)	87 (80–94)	96.5 (88–105)	0.0001	< 0.0001
TOAST subtypes, N (%)						
Large-artery atherosclerosis	78 (25.24%)	992 (25.73%)	1936 (25.84%)	661 (26.54%)	0.0016	
Small-vessel occlusion	52 (16.83%)	790 (20.49%)	1723 (23.00%)	572 (22.96%)		
Cardioembolism	28 (9.06%)	282 (7.32%)	442 (5.90%)	129 (5.18%)		
Stroke of other determined etiology	5 (1.62%)	50 (1.30%)	86 (1.15%)	30 (1.20%)		
Stroke of undetermined etiology	146 (47.25%)	1741 (45.16%)	3304 (44.11%)	1099 (44.12%)		
NIHSS at admission, median (IQR)	5 (2–9)	4 (2–6)	3 (2–6)	3 (2–6)	< 0.0001	< 0.0001
Intravenous thrombolysis, N (%)	55 (17.80%)	465 (12.06%)	751 (10.03%)	249 (10.00%)	< 0.0001	< 0.0001
Arterial thrombolysis or mechanical thrombectomy, N (%)	3 (0.97%)	25 (0.65%)	32 (0.43%)	11 (0.44%)	0.2573	0.096

Kruskal–Wallis test was used to compare group differences for continuous variables, and χ^2^ test was used for categorical variables. P for linear trend was calculated by Kendall's Tau-b correlation analysis for continuous variables or Cochran-Armitage test for categorical variables. ^a^Heavy drinking was defined as alcohol consumption ≥ 2 standard alcohol consumption /day. ^b^Waist circumference was available for 4805 patients. BMI, body mass index; TOAST, Trial of Org 10,172 in Acute Stroke Treatment; NIHSS, National Institutes of Health Stroke Scale

Information on WHtR was available for 4805 patients. The higher WHtR group had larger BMI measures, greater occurrence of hypertension (*P* < 0.0001, P_for linear trend_<0.0001) and lipid metabolism disorders (*P* = 0.0005, P_for linear trend_<0.0001), and less occurrence of atrial fibrillation (*P* = 0.0042, P_for linear trend_<0.0016), much younger age (*P* < 0.0001, P_for linear trend_<0.0001) (Table 2). The distribution of stroke subtypes is not significantly different among groups (Table 2, Fig. 2).

**Table 2:** Baseline characteristics by WHtR groups

Characteristics	WHtR groups				*P*-value	P _for linear trend_
	< 0.47	0.47- < 0.52	0.52- < 0.57	≥ 0.57		
N	1169	1316	1190	1130		
Age (years), median (IQR)	65 (57–74)	64 (56–72)	63 (55–70)	63 (55–71)	< 0.0001	< 0.0001
Male, N (%)	824 (70.49%)	943 (71.66%)	790 (66.39%)	657 (58.14%)	< 0.0001	< 0.0001
Ethnicity(Han), N (%)	1140 (97.52%)	1264 (96.05%)	1130 (94.96%)	1049 (92.83%)	< 0.0001	< 0.0001
Stroke history, N (%)	192 (16.42%)	269 (20.44%)	237 (19.92%)	234 (20.71%)	0.0297	0.0191
Medical comorbidities, N (%)						
Diabetes mellitus	279 (23.87%)	274 (20.82%)	268 (22.52%)	286 (25.31%)	0.0558	0.2682
Atrial fibrillation	111 (9.50%)	108 (8.21%)	102 (8.57%)	63 (5.58%)	0.0042	0.0016
Myocardial infarction	19 (1.63%)	14 (1.06%)	22 (1.85%)	18 (1.59%)	0.4208	0.6407
Hypertension	711 (60.82%)	819 (62.23%)	776 (65.21%)	801 (70.88%)	< 0.0001	< 0.0001
Lipid metabolism disorders	64 (5.47%)	71 (5.40%)	94 (7.90%)	101 (8.94%)	0.0005	< 0.0001
Heavy drinking^a^, N (%)	134 (11.46%)	188 (14.29%)	140 (11.76%)	130 (11.50%)	0.0882	0.5481
Current smoker, N (%)	315 (26.95%)	419 (31.84%)	359 (30.17%)	296 (26.19%)	0.0054	0.4982
Body mass index, median (IQR)	22.84 (20.76–24.80)	23.44 (21.72–25.12)	24.68 (23.05–26.36)	26.71 (24.49–29.05)	< 0.0001	< 0.0001
TOAST subtypes, N (%)						
Large-artery atherosclerosis	281 (24.04%)	347 (26.37%)	298 (25.04%)	298 (26.37%)	0.0575	
Small-vessel occlusion	233 (19.93%)	270 (20.52%)	269 (22.61%)	276 (24.42%)		
Cardioembolism	97 (8.30%)	91 (6.91%)	94 (7.90%)	59 (5.22%)		
Stroke of other determined etiology	21 (1.80%)	21 (1.60%)	20 (1.68%)	15 (1.33%)		
Stroke of undetermined etiology	537 (45.94%)	587 (44.60%)	509 (42.77%)	482 (42.65%)		
NIHSS at admission, median (IQR)	3 (2–6)	3 (2–6)	3 (2–6)	3 (2–6)	0.0832	0.0609
Intravenous thrombolysis, N (%)	143 (12.23%)	171 (12.99%)	128 (10.76%)	94 (8.32%)	0.0016	0.0007
Arterial thrombolysis or mechanical thrombectomy, N (%)	12 (1.03%)	10 (0.76%)	4 (0.34%)	2 (0.18%)	0.0274	0.0028

Kruskal–Wallis test was used to compare group differences for continuous variables, and χ^2^ test was used for categorical variables. P for linear trend was calculated by Kendall's Tau-b correlation analysis for continuous variables or Cochran-Armitage test for categorical variables. ^a^Heavy drinking was defined as alcohol consumption ≥ 2 standard alcohol consumption /day. WHtR, waist-to-height ratio; TOAST, Trial of Org 10172 in Acute Stroke Treatment; NIHSS, National Institutes of Health Stroke Scale

Patients with waist information available compared with patients without waist information had a lower proportion of men (*P* = 0.0008). There were slight differences between BMI data (*P* < 0.0001) and age (*P* < 0.0001), also in the distribution of stroke TOAST criteria (*P* = 0.0006). The NIHSS score at admission is similar between the two groups (*P* = 0.7350) (Table s1).

### Clinical outcomes

**Fig. 3:**
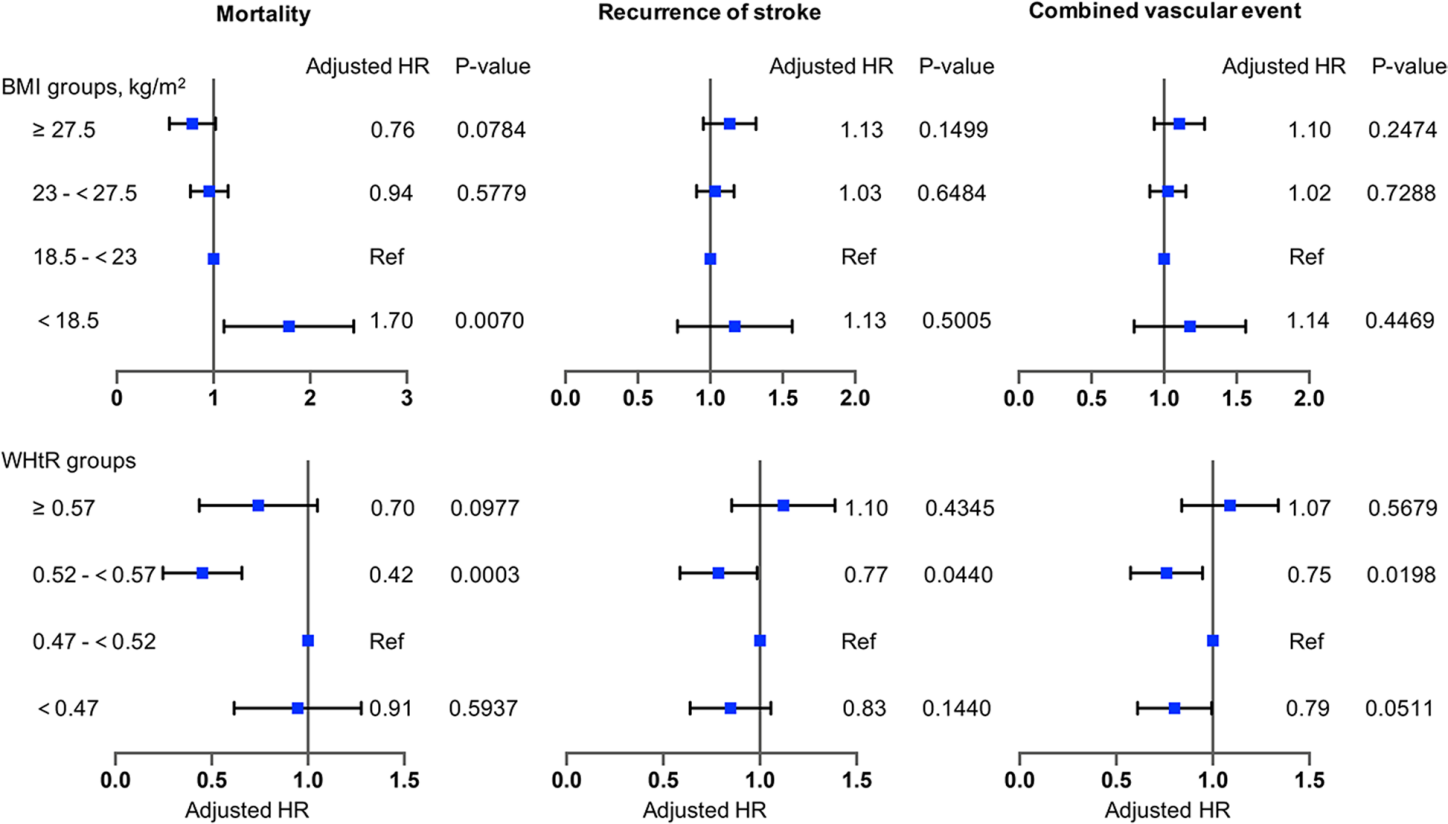
Association of BMI and WHtR with outcomes using Cox proportional hazards models. Adjusted for age, gender, ethnicity, history of diabetes mellitus, history of atrial fibrillation, history of hypertension, history of myocardial infarction, history of lipid metabolism disorders, heavy drinking, smoking, intravenous thrombolysis, arterial thrombolysis or mechanical thrombectomy, NIHSS at admission, TOAST subtypes. BMI, body mass index; WHtR, waist-to-height ratio; NIHSS, National Institutes of Health Stroke Scale; TOAST, Trial of Org 10,172 in Acute Stroke Treatment

When BMI was used as a measure of obesity, in the unadjusted model, compared to the normal weight patients, underweight patients had the highest all-cause mortality (hazard ratio [HR] 2.55 [95% confidence interval (CI) 1.75–3.73], *P* < 0.0001), and mortality decreased in overweight patients (HR 0.74 [0.61–0.91], *P* = 0.0035) and obese patients (HR 0.54 [0.40–0.73], *P* < 0.0001); after adjustment for age, gender, ethnicity, history of diabetes mellitus, history of atrial fibrillation, history of hypertension, history of myocardial infarction, history of lipid metabolism disorders, heavy drinking, smoking, intravenous thrombolysis, arterial thrombolysis or mechanical thrombectomy, NIHSS at admission, TOAST subtypes, the protective effect of obesity and overweight disappeared (Fig. 3, Table 3). Similar to the unadjusted model, underweight patients had higher mortality than normal weight patients (adjusted hazard ratio [aHR] 1.70 [1.16–2.49], *P* = 0.007). Recurrence of stroke and combined vascular events were not significantly lower in overweight and obese patients. Additionally, stroke subtype (TOAST classification) did not modify the association between BMI and clinical outcomes (Table 3).

**Table 3:** Association of BMI/WHtR with outcomes

	Number at risk	Number of events, N (%)	Unadjusted Hazard Ratio(95%CI^a^	*P*-value	Adjusted Hazard Ratio(95%CI) ^b^	*P*-value	P_interaction with stroke subtype_^c^
**mortality**							
BMI groups, kg/m^2^							0.6751
< 18.5	309	32 (10.36%)	2.55 (1.75–3.73)	< 0.0001	1.70 (1.16–2.49)	0.007	
18.5- < 23	3855	162 (4.20%)	1.00 (Ref)		1.00 (Ref)		
23- < 27.5	7491	235 (3.14%)	0.74 (0.61–0.91)	0.0035	0.94 (0.77–1.16)	0.5779	
≥ 27.5	2491	57 (2.29%)	0.54 (0.40–0.73)	< 0.0001	0.76 (0.56–1.03)	0.0784	
WHtR groups							0.6714
≥ 0.57	1130	35 (3.10%)	0.64 (0.43–0.97)	0.0357	0.70 (0.46–1.07)	0.0977	
0.52- < 0.57	1190	25 (2.10%)	0.43 (0.27–0.68)	0.0004	0.42 (0.26–0.67)	0.0003	
0.47- < 0.52	1316	63 (4.79%)	1.00 (Ref)		1.00 (Ref)		
< 0.47	1169	67 (5.73%)	1.21 (0.86–1.71)	0.2785	0.91 (0.64–1.29)	0.5937	
**Recurrence of stroke**							
BMI groups, kg/m^2^							0.6263
< 18.5	309	36 (11.65%)	1.22 (0.87–1.72)	0.2525	1.13 (0.80–1.59)	0.5005	
18.5- < 23	3855	382 (9.91%)	1.00 (Ref)		1.00 (Ref)		
23- < 27.5	7491	741 (9.89%)	1.00 (0.88–1.13)	0.9329	1.03 (0.91–1.17)	0.6484	
≥ 27.5	2491	265 (10.64%)	1.07 (0.92–1.26)	0.3709	1.13 (0.96–1.32)	0.1499	
WHtR groups							0.9101
≥ 0.57	1130	132 (11.68%)	1.07 (0.84–1.35)	0.6012	1.10 (0.87–1.40)	0.4345	
0.52- < 0.57	1190	101 (8.49%)	0.76 (0.59–0.98)	0.032	0.77 (0.60–0.99)	0.0440	
0.47- < 0.52	1316	145 (11.02%)	1.00 (Ref)		1.00 (Ref)		
< 0.47	1169	111 (9.50%)	0.86 (0.67–1.10)	0.2339	0.83 (0.65–1.07)	0.1440	
**Combined vascular event**							
BMI groups, kg/m^2^							0.6467
< 18.5	309	39 (12.62%)	1.24 (0.90–1.73)	0.1934	1.14 (0.82–1.58)	0.4469	
18.5- < 23	3855	307 (10.56%)	1.00 (Ref)		1.00 (Ref)		
23- < 27.5	7491	784 (10.47%)	0.99 (0.88–1.11)	0.8354	1.02 (0.91–1.15)	0.7288	
≥ 27.5	2491	275 (11.04%)	1.05 (0.90–1.22)	0.5664	1.10 (0.94–1.28)	0.2474	
WHtR groups							0.8802
≥ 0.57	1130	140 (12.39%)	1.04 (0.83–1.30)	0.7576	1.07 (0.85–1.35)	0.5679	
0.52- < 0.57	1190	107 (8.99%)	0.74 (0.58–0.94)	0.0194	0.75 (0.58–0.95)	0.0198	
0.47- < 0.52	1316	158 (12.01%)	1.00 (Ref)		1.00 (Ref)		
< 0.47	1169	115 (9.84%)	0.82 (0.64–1.04)	0.1005	0.79 (0.62–1.00)	0.0511	

^a^were calculated using univariate COX proportional hazard models; ^b^were calculated using multivariable COX proportional hazard models adjusted for age, gender, ethnicity, history of diabetes mellitus, history of atrial fibrillation, history of hypertension, history of myocardial infarction, history of lipid metabolism disorders, heavy drinking, smoking, intravenous thrombolysis, arterial thrombolysis or mechanical thrombectomy, NIHSS at admission, TOAST subtypes. ^c^P_interaction with stroke subtype_, the interactions of BMI/WHtR with stroke subtype were investigated with the addition of BMI/WHtR by stroke subtype groups using multivariable Cox proportional hazards models. *BMI* body mass index, *WHtR* waist-to-height ratio, *TOAST* Trial of Org 10,172 in Acute Stroke Treatment, *NIHSS* National Institutes of Health Stroke Scale

Our sensitivity analysis showed similar results when using the Chinese obesity working group-recommended BMI cutoffs (Table s4, s5). Stratified analysis showed that underweight patients had higher all-cause mortality than normal weight patients in females (aHR 2.35 [1.39–3.97], *P* = 0.0014) but not in males. Similar results were found in the old (aHR 1.66 [1.10–2.51], *P* = 0.0151) but not in the young (Table s2, s3).

When WHtR was used as a measure of obesity, obese patients had lower 1-year all-cause mortality (HR 0.64 [0.43–0.97], *P* = 0.0357); overweight patients had lower 1-year all-cause mortality (HR 0.43 [0.27–0.68], *P* = 0.0004), recurrence of stroke (0.76 [0.59–0.98], *P* = 0.032) and combined vascular events (0.74 [0.58–0.94], *P* = 0.0194). After adjustment for multiple confounding factors mentioned above, the protective effect of obesity disappeared; overweight patients still had lower all-cause mortality (aHR 0.42 [0.26–0.67], *P* = 0.0003), recurrence of stroke (aHR 0.77 [0.60–0.99], *P* = 0.0440) and combined vascular events (aHR 0.75 [0.58–0.95], *P* = 0.0198) (Fig. 3, Table 3). Stroke subtype did not modify the association (Table 3).

**Fig. 4:**
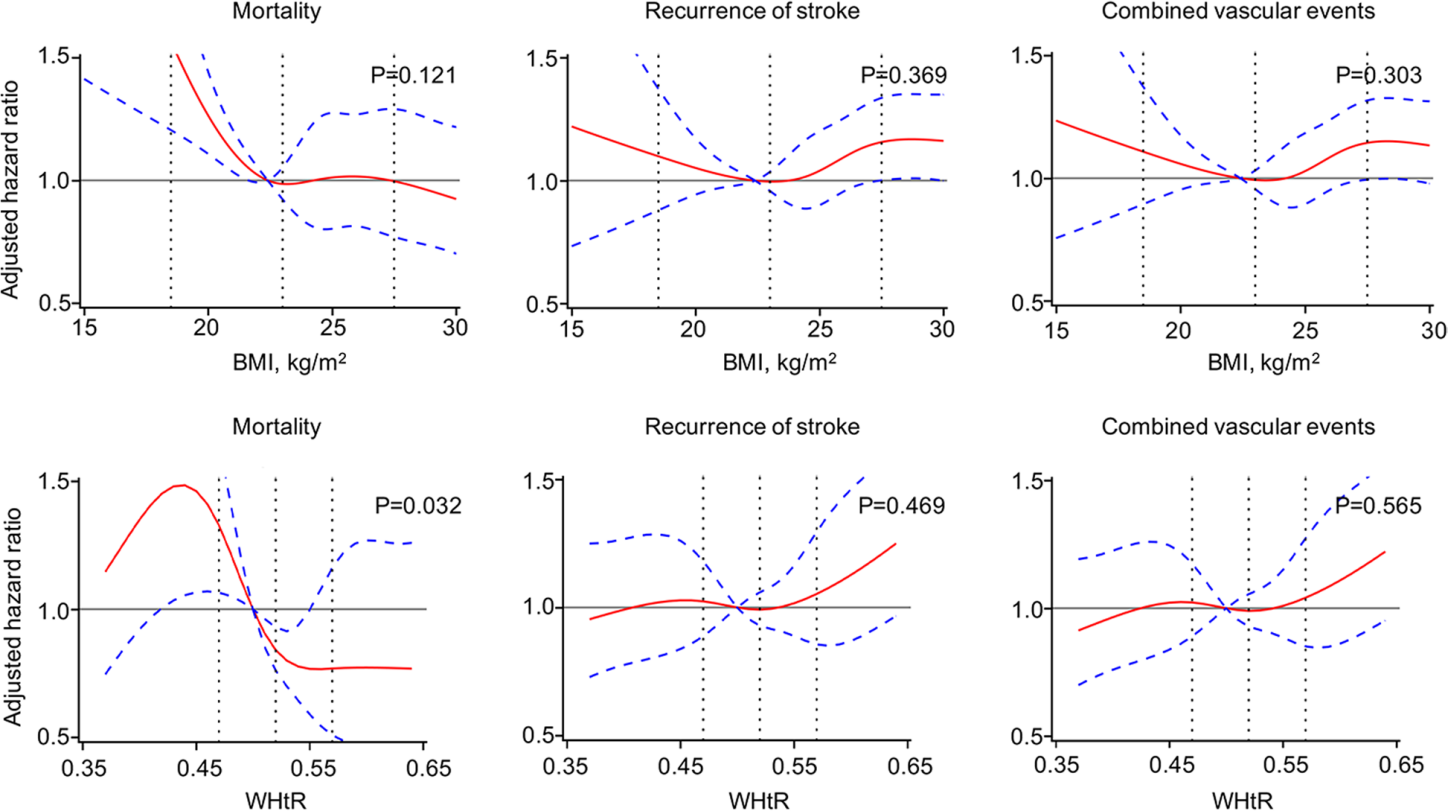
Association of BMI and WHtR with outcomes using cubic spline regression models. The red line represents adjusted HR, and the blue lines depict the 95% CI. Data were fitted with a Cox proportional hazards model adjusted for age, gender, ethnicity, history of diabetes mellitus, history of atrial fibrillation, history of hypertension, history of myocardial infarction, history of lipid metabolism disorders, heavy drinking, smoking, intravenous thrombolysis, arterial thrombolysis or mechanical thrombectomy, NIHSS at admission, TOAST subtypes. BMI, body mass index; WHtR, waist-to-height ratio; NIHSS, National Institutes of Health Stroke Scale; TOAST, Trial of Org 10,172 in Acute Stroke Treatment

Multivariable-adjusted spline regression models showed the relationships between BMI/WHtR and clinical outcomes. When BMI was lower, BMI was inversely associated with 1-year all-cause mortality; as the BMI level increased, the mortality did not decline significantly as before, showing an L-shaped association. Association between BMI and recurrence of stroke, combined vascular events was somewhat U-shaped. When WHtR was lower, WHtR was positively associated with mortality, as the WHtR level increased, association between WHtR and mortality was L-shaped. Association between WHtR and recurrence of stroke, combined vascular events was somewhat J-shaped (Fig. 4).

## Discussion

Our findings in this analysis of 14,146 AIS patients do not support the BMI paradox, TOAST subtypes did not modify the association between obesity and post-stroke mortality, recurrence of stroke and combined vascular events which was not considered in most of the previous studies.

The novel findings are that abdominal obesity also did not follow inverse pattern of association with outcomes, which have rarely been studied in stroke patients. Previous studies on obesity and stroke outcomes have been inconsistent, and most of them used BMI as a measure of obesity. These studies are summarized in Table 4. In our study we noted that overweight patients measured by WHtR had a better prognosis than normal weight patients. Aparicio HJ et al. [14] reported that overweight and mildly obese patients with ischemic stroke but not high obese patients had improved 10-year survival when BMI was used as a measure. This association was more significant in patients younger than 70 years old. Authors speculated that there may be some kind of protective factors associated with moderate weight gain. In addition, certain age is an important factor. In patients with abdominal obesity, excess visceral fat is associated with systemic inflammation [15], and the benefits of moderate weight gain may outweigh its negative effects in AIS patients. Despite WHtR instead of waist circumference or BMI was proposed to be an optimal anthropometric predictor for obesity and metabolic syndrome in previous studies [16, 17], our study still lacks a direct measure of body composition, and clinical trials are required to determine whether and how abdominal fat affects post-stroke outcomes.

**Table 4:** List of studies on the association between obesity and stroke outcomes

References	Design	Number of participants	Main Results
Liu Z et al. 2021 [18]	Placebo-controlled, randomized clinical trial	1033	BMI is inversely associated with short-term mortality, and U-shaped or J-shaped with short-term functional outcomes
Oesch L et al. 2017 [19]	Twenty-five studies, non-randomized studies	299,750	One study showed the association between WHtR and mortality was U-shaped BMI and mortality after stroke: ten of twelve studies support the existence of an obesity paradox BMI and non-fatal outcome: seven of nine studies reported the association between higher body weight and improved non-fatal outcomes
Rozen G et al. 2022 [20]	Real-world national cohort	84,185	Inverse association between BMI and in-hospital mortality
Aparicio HJ et al. 2017 [14]	Nested case–control study	782 (stroke patients)	Overweight and low obese patients but not high obese patients had reduced 10-year mortality
Akyea RK et al. 2021 [21]	A prospective cohort study	30,702	Overweight or obesity was associated with better long-term outcomes, including lower risk of major adverse cardiovascular events and mortality
Jang SY et al. 2015 [22]	A nested case study within a prospective nationwide cohort	2057	Extreme obesity (BMI > 30 kg/m^2^) is associated with short-term good functional outcomes, especially for the young
Pirson FAV et al. 2019 [23]	A post hoc analysis of a national randomized trial for acute ischemic stroke	366	Higher BMI was associated with better short-term prognosis, including improved functional outcome, reduced mortality for large vessel occlusion patients BMI did not affect endovascular treatment effect
Freeman C et al. 2022 [24]	Retrospective cohort study	392	The association between higher BMI and functional gains was affected by age, motor function on admission and diabetes
Scherbakov N et al. 2011 [25]	a multicenter randomized trial and six non-randomized studies	218,826	Higher mortality in undernourished patients (a randomized trial) Inverse association of BMI and mortality (three studies, follow-up time 5–10 years) Increased BMI associated with high mortality (two Asian studies) Weight loss > 3 kg associated with increased mortality (a population-based study)
Xu J et al. 2019 [26]	a nationwide prospective cohort	1227	A Chinese study showed that the BMI paradox existed in insulin-resistant patients but does not in insulin-sensitive ischemic stroke patients

*BMI* body mass index, *WHtR* waist-to-height ratio

The obesity paradox still lacks an accepted biological explanation. This observation has been frequently explained as an artifact of selection bias and that the survival advantage may be due to differences in disease severity or stroke subtypes [18, 27]. Obese patients are more likely to have mild initial neurological severity which is an important factor affecting stroke prognosis [28]. Compared to obesity, the association between initial neurological severity and outcomes might be more critical. In addition, obese patients had lower prevalence of atrial fibrillation and much younger age which is consistent with our study [18], and researchers speculated that the lower mortality in patients with higher BMI may be due to reduced risk of thromboembolic infarcts. However, our results suggested that stroke subtypes did not affect the association between BMI and stroke outcomes. Researchers have reported another explanation——survivor bias [29]. They thought overweight and obese people who died of obesity had already been excluded from the study, and the remaining overweight and obese people included in the study could strive against the rigors of obesity. What’s more, obese patients may get more attention from medical staff and receive prolonged intensive treatment, which may decrease mortality [30]. Lastly, a proposed explanation is that obesity may indicate a higher metabolic reserve to overcome an increased energy expenditure after stroke [2, 18]. Post-stroke weight loss of 3 kg or more has been regarded as a predictor of poor prognosis [31]. The higher metabolic reserve may counteract the negative effects of obesity in post-stroke patients. In our study, after adjustment for confounding factors including age, gender, ethnicity, history of diabetes mellitus, history of atrial fibrillation, history of hypertension, history of myocardial infarction, history of lipid metabolism disorders, heavy drinking, smoking, intravenous thrombolysis, arterial thrombolysis or mechanical thrombectomy, NIHSS at admission, TOAST subtypes, the protective effect of obesity disappeared. Our study did not provide sufficient evidence to support the BMI paradox.

It is interesting to note that, compared to normal weight patients, underweight patients measured by BMI had significantly higher mortality, but when measured by WHtR this did not hold true. We also noted that underweight patients when measured by BMI were much older. We did age-stratified analysis of BMI and mortality which showed that underweight patients had higher mortality than normal weight patients in the old but not in the young. Old people always have more physical and mental illnesses than the young. It is reasonable to speculate that the higher mortality is probably caused by poor quality of life due to physical and mental illness which we could not adjust in the statistical analyses. Kim BJ et al. reported that the inverse association between BMI and long-term mortality was more prominent in stroke patients less than 65 years old [2], and we need to pay attention to long-term prognosis and the dynamics of this phenomenon after stroke. We also did sex-stratified analysis of BMI and 1-year all-cause mortality which showed that underweight patients had higher mortality in females but not in males, and the mortality was almost twice as high in women as in men in underweight group. Female patients should pay more attention to avoiding low body weight after AIS.

This study has both strengths and limitations. This study was a prospective, multicenter registry with a large sample size, where trained research coordinators enrolled eligible patients and collected baseline data by face-to-face interviews with the participants, and an electronic data capture system was used to ensure data quality [11]. Additionally, the baseline data of stroke etiology classification were available which reduced selection bias due to stroke etiological subtypes. Finally, BMI was available for all the patients in our study, while the relatively high rate of missing data on BMI is a common problem in many other studies of BMI and stroke prognosis [2, 27, 32]. The main limitation of our study was the lack of waist circumference data that was available for only a subset of the enrolled patients (4805). Additionally, there may be some missing variables affecting stroke prognosis, such as treatment compliance, treatment options, which we couldn’t include in the multivariable model. Thirdly, CNSR-III study is a hospital-based study, and most of the recruited patients were minor stroke patients, so our conclusion requires further validation in population-based studies and cohorts of AIS patients of varying disease severity.

## Conclusions

Among Chinese patients with AIS, patients with higher BMI had lower mortality, and the protective effect of higher BMI disappeared after adjustment for multiple confounding factors. Our results do not support the BMI paradox. Overweight patients measured by WHtR had better outcomes compared with normal weight patients. Moderately elevated WHtR is associated with improved prognosis. This association was not modified by stroke subtypes. Future clinical studies using alternative measures of fat distribution can enhance our understanding of the obesity paradox.

## Supplementary Information

**Additional file 1: Table s1.** Baseline characteristics comparison of patients with (*n* = 4805 ) and without (*n* = 9341 ) waist circumference measurements. **Table s2.** Age-stratified analysis of BMI and 1-year all-cause mortality. **Table s3.** Sex-stratified analysis of BMI and 1-year all-cause mortality. **Table s4.** Baseline characteristics and outcomes by BMI groups (BMI categories according to Chinese obesity working group). **Table s5.** Association of BMI with 1-year all-cause mortality in the entire cohort (BMI categories according to Chinese obesity working group).

**Additional file 2.**

## Data Availability

The data that support the findings of this study are available from the corresponding author upon reasonable request.

## Abbreviations

aHR: Adjusted hazard ratio
AIS: Acute ischemic stroke
BMI: Body mass index
CI: Confidence interval
CNSR-III: Third China National Stroke Registry
HR: Hazard ratio
NIHSS: National Institutes of Health Stroke Scale
TIA: Transient ischemic attack
TOAST: Trial of Org 10172 in Acute Stroke Treatment
WHtR: Waist-to-height ratio

## References

[1] Ziccardi P, Nappo F, Giugliano G, Esposito K, Marfella R, Cioffi M (2002). Reduction of inflammatory cytokine concentrations and improvement of endothelial functions in obese women after weight loss over one year. Circulation.

[2] Kim BJ, Lee SH, Jung KH, Yu KH, Lee BC, Roh JK (2012). Dynamics of obesity paradox after stroke, related to time from onset, age, and causes of death. Neurology.

[3] Collaboration FT (2003). Poor nutritional status on admission predicts poor outcomes after stroke: observational data from the food trial. Stroke.

[4] Vemmos K, Ntaios G, Spengos K, Savvari P, Vemmou A, Pappa T (2011). Association between obesity and mortality after acute first-ever stroke: the obesity-stroke paradox. Stroke.

[5] Bazzano LA, Gu D, Whelton MR, Wu X, Chen CS, Duan X (2010). Body mass index and risk of stroke among chinese men and women. Ann Neurol.

[6] Yi SW, Odongua N, Nam CM, Sull JW, Ohrr H (2009). Body mass index and stroke mortality by smoking and age at menopause among korean postmenopausal women. Stroke.

[7] Redfors P, Jood K, Holmegaard L, Rosengren A, Blomstrand C, Jern C (2012). Stroke subtype predicts outcome in young and middle-aged stroke sufferers. Acta Neurol Scand.

[8] Despres JP, Lemieux I (2006). Abdominal obesity and metabolic syndrome. Nature.

[9] Da Le CE, Fatemifar G, Palmer TM, White J, Consortium M (2017). Causal associations of adiposity and body fat distribution with coronary heart disease, stroke subtypes, and type 2 diabetes mellitus: a mendelian randomization analysis. Circulation.

[10] Chandramouli C, Tay WT, Bamadhaj NS, Tromp J, Teng THK, Yap JJ (2019). Association of obesity with heart failure outcomes in 11 asian regions: a cohort study. PLoS Med.

[11] Wang Y, Jing J, Meng X, Pan Y, Wang Y, Zhao X (2019). The third china national stroke registry (cnsr-iii) for patients with acute ischaemic stroke or transient ischaemic attack: Design, rationale and baseline patient characteristics. Stroke Vasc Neurol.

[12] Consultation WHOE (2004). Appropriate body-mass index for asian populations and its implications for policy and intervention strategies. Lancet.

[13] Cheng TO (2004). Chinese body mass index is much lower as a risk factor for coronary artery disease. Circulation..

[14] Aparicio HJ, Himali JJ, Beiser AS, Davis-Plourde KL, Vasan RS, Kase CS (2017). Overweight, obesity, and survival after stroke in the framingham heart study. J Am Heart Assoc.

[15] Fontana L, Eagon JC, Trujillo ME, Scherer PE, Klein S (2007). Visceral fat adipokine secretion is associated with systemic inflammation in obese humans. Diabetes.

[16] Ashwell M, Gunn P, Gibson S (2012). Waist-to-height ratio is a better screening tool than waist circumference and bmi for adult cardiometabolic risk factors: Systematic review and meta-analysis. Obes Rev.

[17] Schneider HJ, Klotsche J, Silber S, Stalla GK, Wittchen HU (2011). Measuring abdominal obesity: Effects of height on distribution of cardiometabolic risk factors risk using waist circumference and waist-to-height ratio. Diabetes Care.

[18] Liu Z, Sanossian N, Starkman S, Avila-Rinek G, Eckstein M, Sharma LK (2021). Adiposity and outcome after ischemic stroke: Obesity paradox for mortality and obesity parabola for favorable functional outcomes. Stroke.

[19] Oesch L, Tatlisumak T, Arnold M, Sarikaya H (2017). Obesity paradox in stroke - myth or reality? A systematic review. PLoS ONE.

[20] Rozen G, Elbaz-Greener G, Margolis G, Marai I, Heist EK, Ruskin JN (2022). The obesity paradox in real-world nation-wide cohort of patients admitted for a stroke in the us. J Clin Med.

[21] Akyea RK, Doehner W, Iyen B, Weng SF, Qureshi N, Ntaios G (2021). Obesity and long-term outcomes after incident stroke: a prospective population-based cohort study. J Cachexia Sarcopenia Muscle.

[22] Jang SY, Shin YI, Kim DY, Sohn MK, Lee J, Lee SG (2015). Effect of obesity on functional outcomes at 6 months post-stroke among elderly koreans: a prospective multicentre study. BMJ Open.

[23] Pirson FAV, Hinsenveld WH, Staals J, de Greef BTA, van Zwam WH, Dippel DWJ (2019). The effect of body mass index on outcome after endovascular treatment in acute ischemic stroke patients: a post hoc analysis of the mr clean trial. Cerebrovasc Dis.

[24] Freeman C, Blough A, Rotich D, Curl A, Eickmeyer SM (2022). The obesity paradox may not lead to functional gains in stroke patients undergoing acute inpatient rehabilitation. PM R.

[25] Scherbakov N, Dirnagl U, Doehner W (2011). Body weight after stroke: lessons from the obesity paradox. Stroke.

[26] Xu J, Wang A, Meng X, Jing J, Wang Y, Wang Y, et al. Obesity-stroke paradox exists in insulin-resistant patients but not insulin sensitive patients. Stroke. 2019;50(6):1423–9. 10.1161/STROKEAHA.118.023817 31043152

[27] Dehlendorff C, Andersen KK, Olsen TS (2014). Body mass index and death by stroke: No obesity paradox. JAMA Neurol.

[28] Kim Y, Kim CK, Jung S, Yoon BW, Lee SH (2015). Obesity-stroke paradox and initial neurological severity. J Neurol Neurosurg Psychiatry.

[29] Schooling CM, Cowling BJ, Jones HE (2013). Selection bias in cohorts of cases. Prev Med.

[30] Tan XF, Shi JX, Chen AM (2016). Prolonged and intensive medication use are associated with the obesity paradox after percutaneous coronary intervention: a systematic review and meta-analysis of 12 studies. BMC Cardiovasc Disord.

[31] Jonsson AC, Lindgren I, Norrving B, Lindgren A (2008). Weight loss after stroke: a population-based study from the Lund stroke register. Stroke.

[32] Doehner W, Schenkel J, Anker SD, Springer J, Audebert HJ (2013). Overweight and obesity are associated with improved survival, functional outcome, and stroke recurrence after acute stroke or transient ischaemic attack: Observations from the tempis trial. Eur Heart J.

