# Supplementary material for: Association of body mass index and waist-to-height ratio with outcomes in ischemic stroke: results from the Third China National Stroke Registry
Source: BMC Neurol. 2023 Apr 14;23:152. doi: 10.1186/s12883-023-03165-y (PMC10103413; doi:10.1186/s12883-023-03165-y)
Supplement: Supplementary file 1 — Additional file 1: Table s1. Baseline characteristics comparison of patients with (n = 4805 ) and without (n = 9341 ) waist circumference measurements. Table s2. Age-stratified analysis of BMI and 1-year all-cause mortality. Table s3. Sex-stratified analysis of BMI and 1-year all-cause mortality. Table s4. Baseline characteristics and outcomes by BMI groups (BMI categories according to Chinese obesity working group). Table s5. Association of BMI with 1-year all-cause mortality in the entire cohort (BMI categories according to Chinese obesity working group). [file 12883_2023_3165_MOESM1_ESM.docx]

**Table s1** Baseline characteristics comparison of patients with (n = 4805 ) and without (n = 9341 ) waist circumference measurements

|  | Total | With waist circumference measurements | Without waist circumference measurements | P-value |
| --- | --- | --- | --- | --- |
| N | 14146 | 4805 | 9341 |  |
| Age, (year), median (IQR) | 63 (54-70) | 64 (56-72) | 62 (54-70) | ＜0.0001 |
| Male, N (%) | 9720 (68.71%) | 3214 (66.89%) | 6506 (69.65%) | 0.0008 |
| BMI( kg/m^2^), median (IQR) | 24.49 (22.60-26.56) | 24.22 (22.31-26.37) | 24.49 (22.84-26.57) | ＜0.0001 |
| TOAST subtypes, N (%) |  |  |  | 0.0006 |
| Large-artery atherosclerosis | 3667 (25.92%) | 1224 (25.47%) | 2443 (26.15%) |  |
| Small-vessel occlusion | 3137 (22.18%) | 1048 (21.81%) | 2089 (22.36%) |  |
| Cardioembolism | 881 (6.23%) | 341 (7.10%) | 540 (5.78%) |  |
| Stroke of other determined etiology | 171 (1.21%) | 77 (1.60%) | 94 (1.01%) |  |
| Stroke of undetermined etiology | 6290 (44.46%) | 2115 (44.02%) | 4175 (44.70%) |  |
| NIHSS at admission,  median (IQR) | 3 (2-6) | 3 (2-6) | 3 (2-6) | 0.7350 |

Kruskal-Wallis test was used to compare group differences for continuous variables, and χ2 test was used for categorical variables. BMI, body mass index; TOAST, Trial of Org 10172 in Acute Stroke Treatment; NIHSS, National Institutes of Health Stroke Scale.

| BMI groups,  kg/m^2^ | N at risk | Events, N (%) | Adjusted Hazard Ratio (95%CI)^*^ | P-value | P _interaction with age_ |
| --- | --- | --- | --- | --- | --- |
| Age＜65 0.9473 | | | | | |
| ＜18.5 | 98 | 3 (3.06%) | 1.68 (0.52-5.44) | 0.3909 |  |
| 18.5-＜23 | 1925 | 41(2.13%) | 1.000 (Ref) |  |  |
| 23-＜27.5 | 4448 | 72 (1.62%) | 0.84 (0.57-1.25) | 0.3981 |  |
| ≥ 27.5 | 1614 | 18 (1.12%) | 0.60 (0.34-1.05) | 0.0743 |  |
| Age ≥ 65 | | | | | |
| ＜18.5 | 211 | 29 (13.74%) | 1.66 (1.10-2.51) | 0.0151 |  |
| 18.5-＜23 | 1930 | 121 (6.27%) | 1.00 (Ref) |  |  |
| 23-＜27.5 | 3043 | 163 (5.36%) | 0.99 (0.78-1.26) | 0.9407 |  |
| ≥ 27.5 | 877 | 39 (4.45%) | 0.84 (0.58-1.22) | 0.3586 |  |

**Table s2** Age-stratified analysis of BMI and 1-year all-cause mortality

^*^ were calculated using a multivariate COX proportional hazard model adjusted for age, gender, ethnicity, history of diabetes mellitus, history of atrial fibrillation, history of hypertension, history of myocardial infarction, history of lipid metabolism disorders, history of heavy drinking, smoking, intravenous thrombolysis, arterial thrombolysis or mechanical thrombectomy, NIHSS at admission, TOAST subtypes. BMI, body mass index; TOAST, Trial of Org 10172 in Acute Stroke Treatment; NIHSS, National Institutes of Health Stroke Scale.

| BMI groups, kg/m^2^ | N at risk | Events, N (%) | Adjusted Hazard Ratio (95%CI)^*^ | P-value | P _interaction with gender_ |
| --- | --- | --- | --- | --- | --- |
| Male 0.3904 | | | | | |
| ＜18.5 | 177 | 13 (7.34%) | 1.23 (0.68-2.20) | 0.4952 |  |
| 18.5-＜23 | 2599 | 103 (3.96%) | 1.00 (Ref) |  |  |
| 23-＜27.5 | 5303 | 158 (2.98%) | 0.91 (0.71-1.17) | 0.4547 |  |
| ≥ 27.5 | 1641 | 34 (2.07%) | 0.72 (0.48-1.06) | 0.0977 |  |
| Female |  |  |  |  |  |
| ＜18.5 | 132 | 19 (14.39%) | 2.35 (1.39-3.97) | 0.0014 |  |
| 18.5-＜23 | 1256 | 59 (4.70%) | 1.00 (Ref) |  |  |
| 23-＜27.5 | 2188 | 77 (3.52%) | 1.01 (0.71-1.43) | 0.9754 |  |
| ≥ 27.5 | 850 | 23 (2.71%) | 0.80 (0.49-1.32) | 0.3866 |  |

**Table s3** Sex-stratified analysis of BMI and 1-year all-cause mortality

^*^ were calculated using a multivariate COX proportional hazard model adjusted for age, ethnicity, history of diabetes mellitus, history of atrial fibrillation, history of hypertension, history of myocardial infarction, history of lipid metabolism disorders, history of heavy drinking, smoking, intravenous thrombolysis, arterial thrombolysis or mechanical thrombectomy, NIHSS at admission, TOAST subtypes. BMI, body mass index; TOAST, Trial of Org 10172 in Acute Stroke Treatment; NIHSS, National Institutes of Health Stroke Scale.

**Table s4**  Baseline characteristics and outcomes by BMI groups (BMI categories according to Chinese obesity working group)

|  | BMI groups, kg/m^2^ | | | | P-value | P _for linear trend_ |
| --- | --- | --- | --- | --- | --- | --- |
|  | < 18.5 | 18.5-< 24 | 24-< 28 | ≥ 28 |  |  |
| N | 309 | 5678 | 6215 | 1944 |  |  |
|  |  |  |  |  |  |  |
| Age, (year), median (IQR) | 72 (61-78) | 64 (56-72) | 62 (54-69) | 60 (51-68) | ＜0.0001 | ＜0.0001 |
| Male, N (%) | 177 (57.28%) | 3858 (67.95%) | 4487 (72.20%) | 1198 (61.63%) | ＜0.0001 | 0.5563 |
| Ethnicity（Han), N (%) | 299 (96.76%) | 5547 (97.69%) | 6022 (96.89%) | 1862 (95.78%) | 0.0002 | ＜0.0001 |
| Stroke history, N (%) | 80 (25.89%) | 1198 (21.10%) | 1408 (22.65%) | 448 (23.05%) | 0.0467 | 0.1325 |
| Medical comorbidities, N (%) |  |  |  |  |  |  |
| Diabetes mellitus | 41 (13.27%) | 1163 (20.48%) | 1571 (25.28%) | 535 (27.52%) | ＜0.0001 | ＜0.0001 |
| Atrial fibrillation | 42 (13.59%) | 413 (7.27%) | 405 (6.52%) | 126 (6.48%) | ＜0.0001 | 0.0022 |
| Myocardial  infarction | 10 (3.24%) | 106 (1.87%) | 119 (1.91%) | 43 (2.21%) | 0.3115 | 0.8337 |
| Hypertension | 159 (51.46%) | 3193 (56.23%) | 4090 (65.81%) | 1445 (74.33%) | ＜0.0001 | ＜0.0001 |
| Lipid metabolism  disorders | 16 (5.18%) | 355 (6.25%) | 482 (7.76%) | 222 (11.42%) | ＜0.0001 | ＜0.0001 |
| Heavy drinking^*^, N (%) | 38 (12.30%) | 799 (14.07%) | 934 (15.03%) | 239 (12.29%) | 0.0163 | 0.5604 |
| Current smoker, N (%) | 99 (32.04%) | 1786 (31.45%) | 2026 (32.60%) | 592 (30.45%) | 0.2879 | 0.8691 |
| Waist circumference (cm), median (IQR)^#^ | 72 (69-79) | 82 (76-88) | 88 (81-95) | 98 (89-105) | ＜0.0001 | ＜0.0001 |
| TOAST subtypes, N (%) |  |  |  |  | 0.0237 |  |
| Large-artery atherosclerosis | 78 (25.24%) | 1454 (25.61%) | 1609 (25.89%) | 526 (27.06%) |  |  |
| Cardioembolism | 28 (9.06%) | 384 (6.76%) | 359 (5.78%) | 110 (5.66%) |  |  |
| Small-vessel occlusion | 52 (16.83%) | 1217 (21.43%) | 1413 (22.74%) | 455 (23.41%) |  |  |
| Stroke of other determined etiology | 5 (1.62%) | 75 (1.32%) | 65 (1.05%) | 26 (1.34%) |  |  |
| Stroke of undetermined etiology | 146 (47.25%) | 2548 (44.87%) | 2769 (44.55%) | 827 (42.54%) |  |  |
| NIHSS at admission,  median (IQR) | 5 (2-9) | 4 (2-6) | 3 (2-6) | 3 (1-6) | ＜0.0001 | ＜0.0001 |
| Intravenous thrombolysis, N (%) | 55 (17.80%) | 631 (11.11%) | 639 (10.28%) | 195 (10.03%) | 0.0002 | 0.0035 |
| Arterial thrombolysis or mechanical thrombectomy, N (%) | 3 (0.97%) | 34 (0.60%) | 25 (0.40%) | 9 (0.46%) | 0.2929 | 0.1351 |
| 1-year all-cause mortality, N (%) | 32 (10.36%) | 231 (4.07%) | 175 (2.82%) | 48 (2.47%) | ＜0.0001 | ＜0.0001 |
| 1-year recurrence of stroke, N (%) | 36 (11.65%) | 557 (9.81%) | 624 (10.04%) | 207 (10.65%) | 0.5722 | 0.5374 |
| 1-year combined vascular event, N (%) | 39 (12.62%) | 595 (10.48%) | 657 (10.57%) | 214 (11.01%) | 0.6283 | 0.8807 |

Kruskal-Wallis test was used to compare group differences for continuous variables, and χ2 test was used for categorical variables. P for linear trend was calculated by Kendall's Tau-b correlation analysis for continuous variables or Cochran-Armitage test for categorical variables. ^*^ Heavy drinking was defined as alcohol consumption ≥ 2 standard alcohol consumption /day. ^#^ Waist circumference was available for 4805 patients. BMI, body mass index; TOAST, Trial of Org 10172 in Acute Stroke Treatment; NIHSS, National Institutes of Health Stroke Scale.

**Table s5** Association of BMI with 1-year all-cause mortality in the entire cohort (BMI categories according to Chinese obesity working group)

|  | Number at risk | Number of events, N (%) | Unadjusted Hazard  Ratio （95%CI）^#^ | P-value | Adjusted Hazard  Ratio（95%CI）^*^ | P-value | P_interaction_  _With Stroke Subtype_ |
| --- | --- | --- | --- | --- | --- | --- | --- |
| BMI groups, kg/m^2^ |  |  |  |  |  |  | 0.5881 |
| ＜18.5 | 309 | 32 (10.36%) | 2.64 (1.83-3.82) | ＜ 0.0001 | 1.67 (1.15-2.43) | 0.0073 |  |
| 18.5-＜24 | 5678 | 231 (4.07%) | 1.00 (Ref) |  | 1.00 (Ref) |  |  |
| 24-＜28 | 6215 | 175 (2.82%) | 0.69 (0.57-0.84) | 0.0002 | 0.86 (0.70-1.05) | 0.1355 |  |
| ≥ 28 | 1944 | 48 (2.47%) | 0.60 (0.44-0.82) | 0.0014 | 0.80 (0.58-1.10) | 0.1671 |  |

^#^ were calculated using a univariate COX proportional hazard model; ^*^ were calculated using a multivariate COX proportional hazard model adjusted for age, gender, ethnicity, history of diabetes mellitus, history of atrial fibrillation, history of hypertension, history of myocardial infarction, history of lipid metabolism disorders, history of heavy drinking, smoking, intravenous thrombolysis, arterial thrombolysis or mechanical thrombectomy, NIHSS at admission, TOAST subtypes. The interaction of BMI with stroke subtype was investigated with the addition of BMI by stroke subtype groups using multivariable Cox proportional hazards models. BMI, body mass index; TOAST, Trial of Org 10172 in Acute Stroke Treatment; NIHSS, National Institutes of Health Stroke Scale.
