# Supplementary material for: Association of body mass index and waist-to-height ratio with outcomes in ischemic stroke: results from the Third China National Stroke Registry
Source: BMC Neurol. 2023 Apr 14;23:152. doi: 10.1186/s12883-023-03165-y (PMC10103413; doi:10.1186/s12883-023-03165-y)
Supplement: Supplementary file 2 — Additional file 2. [file 12883_2023_3165_MOESM2_ESM.zip › raw data/Table3 and fig3fig4-bmi.pdf]

FREQ 过程

N12.Follow-up events at 12 months: Whether the patient died: 0-survival;1-death;		
y1_death	频数	累积频数
0	13660	13660
1	486	14146

等比例的卡方检验	
卡方	12268.7881
自由度	1
Pr > 卡方	<.0001

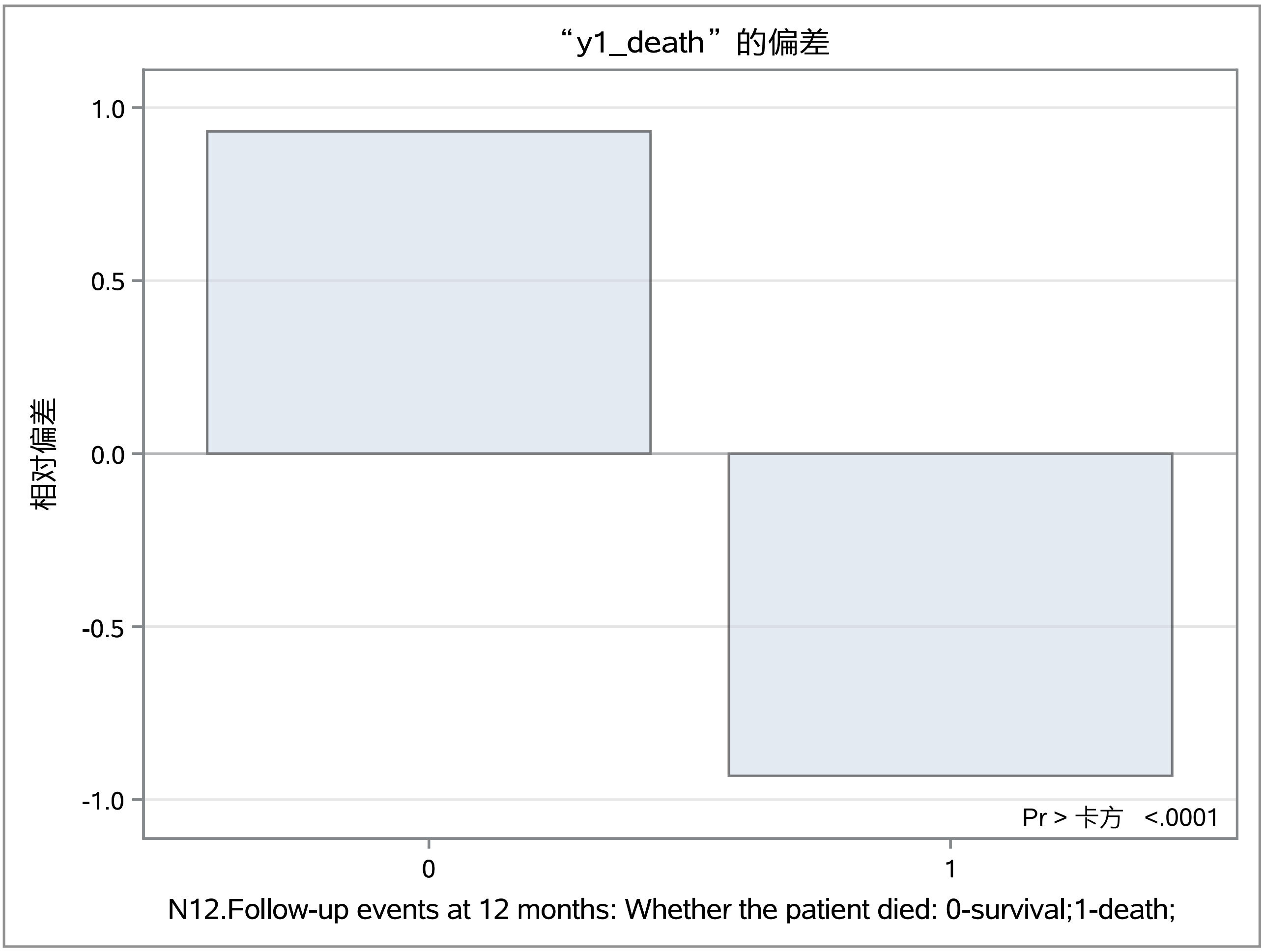

样本大小 = 14146

PHREG 过程

模型信息		
数据集	WORK.DATA2	
因变量	y1_death_dd	N12.Follow-up events at 12 months: Days from onset to death;(day);
删失变量	y1_death	N12.Follow-up events at 12 months: Whether the patient died: 0-survival;1-death;
删失值	0	
结值处理	BRESLOW	

读取的观测数	14146
使用的观测数	14146

事件和删失值个数汇总			
合计	事件	删失	删失百分比
14146	486	13660	96.56

收敛状态
满足收敛准则 (GCONV=1E-8)。

模型拟合统计量		
准则	无协变量	带协变量
-2 LOG L	9255.945	9214.018
AIC	9255.945	9216.018
SBC	9255.945	9220.204

检验全局原假设: BETA=0			
检验	卡方	自由度	Pr > 卡方
似然比	41.9270	1	<.0001
评分	39.2155	1	<.0001
Wald	40.2417	1	<.0001

最大似然估计分析									
参数	自由度	参数估计	标准误差	卡方	Pr > 卡方	危险率	95% 危险率置信限		标签
BMI	1	-0.09403	0.01482	40.2417	<.0001	0.910	0.884	0.937	F.Physical examination: Body mass index (kg/m2);

BMI with y1_death: adjusted model

PHREG 过程

模型信息		
数据集	WORK.DATA2	
因变量	y1_death_dd	N12.Follow-up events at 12 months: Days from onset to death;(day);
删失变量	y1_death	N12.Follow-up events at 12 months: Whether the patient died: 0-survival;1-death;
删失值	0	
结值处理	BRESLOW	

读取的观测数	14146
使用的观测数	14146

分类水平信息						
分类	值	设计变量				
GENDER	2	1				
	1	0				
ETHNIC	2	1				
	1	0				
H_DIAB01	1	1				
	0	0				
H_AF01	1	1				
	0	0				
H_HYPT01	1	1				
	0	0				
H_LIPID01	1	1				
	0	0				
AI	1	1				
	0	0				
H_DRINK_H01	1	1				
	0	0				
H_SMK_C01	1	1				
	0	0				
IT	1	1				
	0	0				
ET	1	1				
	0	0				
IMG_C_TOAST	5	1	0	0	0	
	4	0	1	0	0	
	3	0	0	1	0	
	2	0	0	0	1	
	1	0	0	0	0	

BMI with y1_death: adjusted model

PHREG 过程

事件和删失值个数汇总			
合计	事件	删失	删失<br>百分比
14146	486	13660	96.56

收敛状态
满足收敛准则 (GCONV=1E-8)。

模型拟合统计量		
准则	无<br>协变量	带<br>协变量
-2 LOG L	9255.945	8612.779
AIC	9255.945	8648.779
SBC	9255.945	8724.130

检验全局原假设: BETA=0			
检验	卡方	自由度	Pr > 卡方
似然比	643.1662	18	<.0001
评分	864.4894	18	<.0001
Wald	753.3524	18	<.0001

3 型检验			
效应	自由度	Wald 卡方	Pr > 卡方
BMI	1	7.6342	0.0057
AGE	1	151.3622	<.0001
GENDER	1	1.4715	0.2251
ETHNIC	1	3.7080	0.0542
H_DIAB01	1	12.2288	0.0005
H_AF01	1	29.5276	<.0001
H_HYPT01	1	0.2046	0.6511
H_LIPID01	1	2.1541	0.1422
AI	1	5.2230	0.0223
H_DRINK_H01	1	6.9709	0.0083
H_SMK_C01	1	0.9672	0.3254
IT	1	19.2782	<.0001
ET	1	7.3996	0.0065
IMG_C_TOAST	4	35.0394	<.0001
A_NIHSS	1	228.5723	<.0001

BMI with y1_death: adjusted model

PHREG 过程

最大似然估计分析										
参数		自由度	参数估计	标准误差	卡方	Pr > 卡方	危险率	95% 危险率置信限	
BMI		1	-0.03892	0.01409	7.6342	0.0057	0.962	0.936	0.989
AGE		1	0.05916	0.00481	151.3622	<.0001	1.061	1.051	1.071
GENDER	2	1	-0.12412	0.10232	1.4715	0.2251	0.883	0.723	1.079
ETHNIC	2	1	0.45284	0.23516	3.7080	0.0542	1.573	0.992	2.494
H_DIAB01	1	1	0.36395	0.10408	12.2288	0.0005	1.439	1.173	1.765
H_AF01	1	1	0.81245	0.14951	29.5276	<.0001	2.253	1.681	3.021
H_HYPT01	1	1	0.04425	0.09783	0.2046	0.6511	1.045	0.863	1.266
H_LIPID01	1	1	-0.30464	0.20757	2.1541	0.1422	0.737	0.491	1.108
AI	1	1	0.51703	0.22623	5.2230	0.0223	1.677	1.076	2.613
H_DRINK_H01	1	1	-0.51695	0.19580	6.9709	0.0083	0.596	0.406	0.875
H_SMK_C01	1	1	0.12210	0.12416	0.9672	0.3254	1.130	0.886	1.441
IT	1	1	-0.72950	0.16615	19.2782	<.0001	0.482	0.348	0.668
ET	1	1	0.85199	0.31321	7.3996	0.0065	2.344	1.269	4.331
IMG_C_TOAST	5	1	-0.23261	0.11130	4.3679	0.0366	0.792	0.637	0.986
IMG_C_TOAST	4	1	0.65387	0.32719	3.9938	0.0457	1.923	1.013	3.652
IMG_C_TOAST	3	1	-0.93227	0.18256	26.0775	<.0001	0.394	0.275	0.563
IMG_C_TOAST	2	1	-0.42895	0.19355	4.9118	0.0267	0.651	0.446	0.952
A_NIHSS		1	0.09488	0.00628	228.5723	<.0001	1.100	1.086	1.113

BMI with y1_death: adjusted model

PHREG 过程

最大似然估计分析		
参数		标签
BMI		F.Physical examination: Body mass index (kg/m2);
AGE		A.Basic Information: Age (years old);
GENDER	2	A.Basic Information: Gender; 1-male; 2-female; 2
ETHNIC	2	B.Demography: Race: 1-Han; 99-others; 2
H_DIAB01	1	D.History: Diabetes; 0-No; 1-Yes; 1
H_AF01	1	D.History: Heart disease category: Atrial fibrillation(Including medical history and hospitalization diagnosis); 0-No; 1-Yes; 1
H_HYPT01	1	D.History: Hypertension; 0-No; 1-Yes; 1
H_LIPID01	1	D.History: Lipid metabolism disorders; 0-No; 1-Yes; 1
AI	1	history:Myocardial infarction; 0=NO; 1=YES; 1
H_DRINK_H01	1	D.History: Heavy Drinking(Alcohol consumption>=20g/day); 0-No,1-Yes; 1
H_SMK_C01	1	D.History: Current Smoking; 0-No,1-Yes; 1
IT	1	intravenous thrombolysis, 1=YES,0=NO 1
ET	1	动脉溶栓或机械取栓, 1=YES,0=NO 1
IMG_C_TOAST	5	K.Final diagnosis: cerebral infarction; Etiology according to TOAST system; 1-large artery atherosclerosis; 2-cardiogenic embolism; 3-small artery occlusion; 4-stroke of another determined cause; 5-stroke of an undetermined cause. 5
IMG_C_TOAST	4	K.Final diagnosis: cerebral infarction; Etiology according to TOAST system; 1-large artery atherosclerosis; 2-cardiogenic embolism; 3-small artery occlusion; 4-stroke of another determined cause; 5-stroke of an undetermined cause. 4
IMG_C_TOAST	3	K.Final diagnosis: cerebral infarction; Etiology according to TOAST system; 1-large artery atherosclerosis; 2-cardiogenic embolism; 3-small artery occlusion; 4-stroke of another determined cause; 5-stroke of an undetermined cause. 3
IMG_C_TOAST	2	K.Final diagnosis: cerebral infarction; Etiology according to TOAST system; 1-large artery atherosclerosis; 2-cardiogenic embolism; 3-small artery occlusion; 4-stroke of another determined cause; 5-stroke of an undetermined cause. 2
A_NIHSS		F.Admitting NIHSS: Total score;

BMI with y1_death: interaction with stroke subtype

PHREG 过程

模型信息		
数据集	WORK.DATA2	
因变量	y1_death_dd	N12.Follow-up events at 12 months: Days from onset to death;(day);
删失变量	y1_death	N12.Follow-up events at 12 months: Whether the patient died: 0-survival;1-death;
删失值	0	
结值处理	BRESLOW	

读取的观测数<br>使用的观测数	14146<br>14146

分类水平信息					
分类	值	设计变量			
GENDER	2	1			
	1	0			
ETHNIC	2	1			
	1	0			
H_DIAB01	1	1			
	0	0			
H_AF01	1	1			
	0	0			
H_HYPT01	1	1			
	0	0			
H_LIPID01	1	1			
	0	0			
AI	1	1			
	0	0			
H_DRINK_H01	1	1			
	0	0			
H_SMK_C01	1	1			
	0	0			
IT	1	1			
	0	0			
ET	1	1			
	0	0			
IMG_C_TOAST	5	1	0	0	0
	4	0	1	0	0
	3	0	0	1	0
	2	0	0	0	1
	1	0	0	0	0

BMI with y1_death: interaction with stroke subtype

PHREG 过程

事件和删失值个数汇总			
合计	事件	删失	删失百分比
14146	486	13660	96.56

收敛状态
满足收敛准则 (GCONV=1E-8)。

模型拟合统计量		
准则	无协变量	带协变量
-2 LOG L	9255.945	8604.849
AIC	9255.945	8648.849
SBC	9255.945	8740.946

检验全局原假设: BETA=0			
检验	卡方	自由度	Pr > 卡方
似然比	651.0956	22	<.0001
评分	882.2772	22	<.0001
Wald	771.3525	22	<.0001

联合检验			
效应	自由度	Wald 卡方	Pr > 卡方
BMI	1	0.0006	0.9803
IMG_C_TOAST	4	9.6019	0.0477
BMI*IMG_C_TOAST	4	7.7130	0.1027
AGE	1	149.7490	<.0001
GENDER	1	1.4925	0.2218
ETHNIC	1	3.3670	0.0665
H_DIAB01	1	12.1795	0.0005
H_AF01	1	29.0943	<.0001
H_HYPT01	1	0.2355	0.6275
H_LIPID01	1	2.3037	0.1291
AI	1	5.3766	0.0204
H_DRINK_H01	1	7.0698	0.0078
H_SMK_C01	1	1.0994	0.2944
IT	1	19.1919	<.0001
ET	1	7.8276	0.0051
A_NIHSS	1	225.6003	<.0001

Note: Under full-rank parameterizations, Type 3 effect tests are replaced by joint tests. The joint test for an effect is a test that all of the parameters associated with that effect are zero. Such joint tests might not be equivalent to Type 3 effect tests under GLM parameterization.

BMI with y1_death: interaction with stroke subtype

PHREG 过程

最大似然估计分析									
参数		自由度	参数估计	标准误差	卡方	Pr > 卡方	危险率	95% 危险率置信限	
BMI		1	-0.0006048	0.02449	0.0006	0.9803	.	.	.
IMG_C_TOAST	5	1	1.36937	0.76936	3.1680	0.0751	.	.	.
IMG_C_TOAST	4	1	5.00261	2.46480	4.1194	0.0424	.	.	.
IMG_C_TOAST	3	1	-1.32487	1.31084	1.0215	0.3122	.	.	.
IMG_C_TOAST	2	1	0.43593	1.04831	0.1729	0.6775	.	.	.
BMI*IMG_C_TOAST	5	1	-0.06708	0.03178	4.4564	0.0348	.	.	.
BMI*IMG_C_TOAST	4	1	-0.18899	0.11044	2.9282	0.0870	.	.	.
BMI*IMG_C_TOAST	3	1	0.01555	0.05277	0.0869	0.7682	.	.	.
BMI*IMG_C_TOAST	2	1	-0.03556	0.04334	0.6730	0.4120	.	.	.
AGE		1	0.05886	0.00481	149.7490	<.0001	1.061	1.051	1.071
GENDER	2	1	-0.12511	0.10241	1.4925	0.2218	0.882	0.722	1.079
ETHNIC	2	1	0.43362	0.23631	3.3670	0.0665	1.543	0.971	2.452
H_DIAB01	1	1	0.36365	0.10420	12.1795	0.0005	1.439	1.173	1.765
H_AF01	1	1	0.80599	0.14943	29.0943	<.0001	2.239	1.670	3.001
H_HYPT01	1	1	0.04753	0.09794	0.2355	0.6275	1.049	0.866	1.271
H_LIPID01	1	1	-0.31527	0.20771	2.3037	0.1291	0.730	0.486	1.096
AI	1	1	0.52544	0.22661	5.3766	0.0204	1.691	1.085	2.637
H_DRINK_H01	1	1	-0.52042	0.19572	7.0698	0.0078	0.594	0.405	0.872
H_SMK_C01	1	1	0.13016	0.12414	1.0994	0.2944	1.139	0.893	1.453
IT	1	1	-0.72774	0.16612	19.1919	<.0001	0.483	0.349	0.669
ET	1	1	0.87728	0.31356	7.8276	0.0051	2.404	1.300	4.445
A_NIHSS		1	0.09449	0.00629	225.6003	<.0001	1.099	1.086	1.113

BMI with y1_death: interaction with stroke subtype

PHREG 过程

最大似然估计分析		
参数		标签
BMI		F.Physical examination: Body mass index (kg/m2);
IMG_C_TOAST	5	K.Final diagnosis: cerebral infarction; Etiology according to TOAST system; 1-large artery atherosclerosis; 2-cardiogenic embolism; 3-small artery occlusion; 4-stroke of another determined cause; 5-stroke of an undetermined cause. 5
IMG_C_TOAST	4	K.Final diagnosis: cerebral infarction; Etiology according to TOAST system; 1-large artery atherosclerosis; 2-cardiogenic embolism; 3-small artery occlusion; 4-stroke of another determined cause; 5-stroke of an undetermined cause. 4
IMG_C_TOAST	3	K.Final diagnosis: cerebral infarction; Etiology according to TOAST system; 1-large artery atherosclerosis; 2-cardiogenic embolism; 3-small artery occlusion; 4-stroke of another determined cause; 5-stroke of an undetermined cause. 3
IMG_C_TOAST	2	K.Final diagnosis: cerebral infarction; Etiology according to TOAST system; 1-large artery atherosclerosis; 2-cardiogenic embolism; 3-small artery occlusion; 4-stroke of another determined cause; 5-stroke of an undetermined cause. 2
BMI*IMG_C_TOAST	5	K.Final diagnosis: cerebral infarction; Etiology according to TOAST system; 1-large artery atherosclerosis; 2-cardiogenic embolism; 3-small artery occlusion; 4-stroke of another determined cause; 5-stroke of an undetermined cause. 5 * F.Physical examinatio
BMI*IMG_C_TOAST	4	K.Final diagnosis: cerebral infarction; Etiology according to TOAST system; 1-large artery atherosclerosis; 2-cardiogenic embolism; 3-small artery occlusion; 4-stroke of another determined cause; 5-stroke of an undetermined cause. 4 * F.Physical examinatio
BMI*IMG_C_TOAST	3	K.Final diagnosis: cerebral infarction; Etiology according to TOAST system; 1-large artery atherosclerosis; 2-cardiogenic embolism; 3-small artery occlusion; 4-stroke of another determined cause; 5-stroke of an undetermined cause. 3 * F.Physical examinatio
BMI*IMG_C_TOAST	2	K.Final diagnosis: cerebral infarction; Etiology according to TOAST system; 1-large artery atherosclerosis; 2-cardiogenic embolism; 3-small artery occlusion; 4-stroke of another determined cause; 5-stroke of an undetermined cause. 2 * F.Physical examinatio
AGE		A.Basic Information: Age (years old);
GENDER	2	A.Basic Information: Gender; 1-male; 2-female; 2
ETHNIC	2	B.Demography: Race: 1-Han; 99-others; 2
H_DIAB01	1	D.History: Diabetes; 0-No; 1-Yes; 1
H_AF01	1	D.History: Heart disease category: Atrial fibrillation(Including medical history and hospitalization diagnosis); 0-No; 1-Yes; 1
H_HYPT01	1	D.History: Hypertension; 0-No; 1-Yes; 1
H_LIPID01	1	D.History: Lipid metabolism disorders; 0-No; 1-Yes; 1
AI	1	history:Myocardial infarction; 0=NO; 1=YES; 1
H_DRINK_H01	1	D.History: Heavy Drinking(Alcohol consumption>=20g/day); 0-No,1-Yes; 1
H_SMK_C01	1	D.History: Current Smoking; 0-No,1-Yes; 1
IT	1	intravenous thrombolysis, 1=YES,0=NO 1
ET	1	动脉溶栓或机械取栓, 1=YES,0=NO 1
A_NIHSS		F.Admitting NIHSS: Total score;

FREQ 过程

N12.Follow-up events at 12 months:Occurrence of combined vascular event(including cardiovascular death,non-fatal stroke,non-fatal myocardial infarction):0-No;1-Yes;		
y1_comb	频数	累积频数
0	12641	12641
1	1505	14146

等比例的卡方检验	
卡方	8766.4708
自由度	1
Pr > 卡方	<.0001

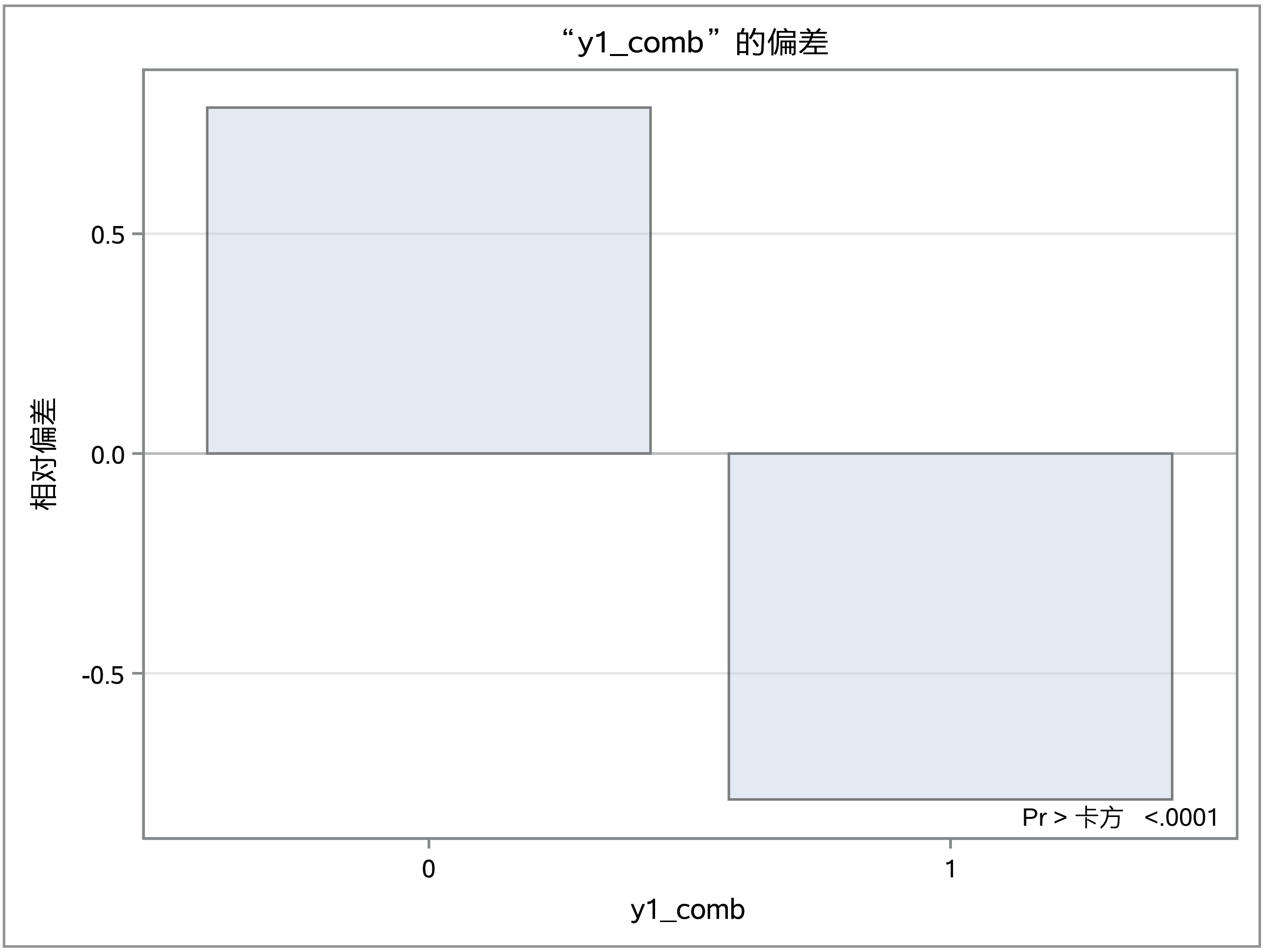

样本大小 = 14146

BMI with y1_comb: crude model

PHREG 过程

模型信息		
数据集	WORK.DATA2	
因变量	y1_comb_dd	N12.Follow-up events at 12 months: Days from onset to occurrence of combined vascular event;(day);
删失变量	y1_comb	N12.Follow-up events at 12 months:Occurrence of combined vascular event(including cardiovascular death,non-fatal stroke,non-fatal myocardial infarction):0-No;1-Yes;
删失值	0	
结值处理	BRESLOW	

读取的观测数	14146
使用的观测数	14146

事件和删失值个数汇总			
合计	事件	删失	删失百分比
14146	1505	12641	89.36

收敛状态
满足收敛准则 (GCONV=1E-8)。

模型拟合统计量		
准则	无协变量	带协变量
-2 LOG L	28553.433	28553.431
AIC	28553.433	28555.431
SBC	28553.433	28560.747

检验全局原假设: BETA=0			
检验	卡方	自由度	Pr > 卡方
似然比	0.0023	1	0.9622
评分	0.0023	1	0.9621
Wald	0.0023	1	0.9621

最大似然估计分析									
参数	自由度	参数估计	标准误差	卡方	Pr > 卡方	危险率	95% 危险率置信限		标签
BMI	1	0.0003691	0.00777	0.0023	0.9621	1.000	0.985	1.016	F.Physical examination: Body mass index (kg/m2);

BMI with y1_comb: adjusted model

PHREG 过程

模型信息		
数据集	WORK.DATA2	
因变量	y1_comb_dd	N12.Follow-up events at 12 months: Days from onset to occurrence of combined vascular event;(day);
删失变量	y1_comb	N12.Follow-up events at 12 months:Occurrence of combined vascular event(including cardiovascular death,non-fatal stroke,non-fatal myocardial infarction):0-No;1-Yes;
删失值	0	
结值处理	BRESLOW	

读取的观测数	14146
使用的观测数	14146

分类水平信息						
分类	值	设计变量				
GENDER	2	1				
	1	0				
ETHNIC	2	1				
	1	0				
H_DIAB01	1	1				
	0	0				
H_AF01	1	1				
	0	0				
H_HYPT01	1	1				
	0	0				
H_LIPID01	1	1				
	0	0				
AI	1	1				
	0	0				
H_DRINK_H01	1	1				
	0	0				
H_SMK_C01	1	1				
	0	0				
IT	1	1				
	0	0				
ET	1	1				
	0	0				
IMG_C_TOAST	5	1	0	0	0	
	4	0	1	0	0	
	3	0	0	1	0	
	2	0	0	0	1	
	1	0	0	0	0	

BMI with y1_comb: adjusted model

PHREG 过程

事件和删失值个数汇总			
合计	事件	删失	删失百分比
14146	1505	12641	89.36

收敛状态
满足收敛准则 (GCONV=1E-8)。

模型拟合统计量		
准则	无协变量	带协变量
-2 LOG L	28553.433	28382.468
AIC	28553.433	28418.468
SBC	28553.433	28514.166

检验全局原假设: BETA=0			
检验	卡方	自由度	Pr > 卡方
似然比	170.9650	18	<.0001
评分	182.2822	18	<.0001
Wald	178.1860	18	<.0001

3 型检验			
效应	自由度	Wald 卡方	Pr > 卡方
BMI	1	0.7350	0.3913
AGE	1	19.8835	<.0001
GENDER	1	0.2118	0.6453
ETHNIC	1	0.4049	0.5246
H_DIAB01	1	13.9884	0.0002
H_AF01	1	13.5341	0.0002
H_HYPT01	1	2.6768	0.1018
H_LIPID01	1	0.6356	0.4253
AI	1	2.1359	0.1439
H_DRINK_H01	1	1.3017	0.2539
H_SMK_C01	1	0.2681	0.6046
IT	1	0.2592	0.6107
ET	1	6.0265	0.0141
IMG_C_TOAST	4	54.3966	<.0001
A_NIHSS	1	16.2978	<.0001

BMI with y1_comb: adjusted model

PHREG 过程

最大似然估计分析									
参数		自由度	参数估计	标准误差	卡方	Pr > 卡方	危险率	95% 危险率置信限	
BMI		1	0.00673	0.00786	0.7350	0.3913	1.007	0.991	1.022
AGE		1	0.01116	0.00250	19.8835	<.0001	1.011	1.006	1.016
GENDER	2	1	0.02789	0.06060	0.2118	0.6453	1.028	0.913	1.158
ETHNIC	2	1	-0.10217	0.16056	0.4049	0.5246	0.903	0.659	1.237
H_DIAB01	1	1	0.22038	0.05892	13.9884	0.0002	1.247	1.111	1.399
H_AF01	1	1	0.42527	0.11560	13.5341	0.0002	1.530	1.220	1.919
H_HYPT01	1	1	0.09141	0.05587	2.6768	0.1018	1.096	0.982	1.223
H_LIPID01	1	1	-0.07951	0.09973	0.6356	0.4253	0.924	0.760	1.123
AI	1	1	0.23084	0.15795	2.1359	0.1439	1.260	0.924	1.717
H_DRINK_H01	1	1	0.09400	0.08239	1.3017	0.2539	1.099	0.935	1.291
H_SMK_C01	1	1	-0.03480	0.06722	0.2681	0.6046	0.966	0.847	1.102
IT	1	1	0.04163	0.08177	0.2592	0.6107	1.043	0.888	1.224
ET	1	1	0.61300	0.24971	6.0265	0.0141	1.846	1.132	3.011
IMG_C_TOAST	5	1	-0.32929	0.06148	28.6838	<.0001	0.719	0.638	0.812
IMG_C_TOAST	4	1	-0.02493	0.22327	0.0125	0.9111	0.975	0.630	1.511
IMG_C_TOAST	3	1	-0.52910	0.07915	44.6864	<.0001	0.589	0.504	0.688
IMG_C_TOAST	2	1	-0.42497	0.13550	9.8357	0.0017	0.654	0.501	0.853
A_NIHSS		1	0.02302	0.00570	16.2978	<.0001	1.023	1.012	1.035

BMI with y1_comb: adjusted model

PHREG 过程

最大似然估计分析		
参数		标签
BMI		F.Physical examination: Body mass index (kg/m2);
AGE		A.Basic Information: Age (years old);
GENDER	2	A.Basic Information: Gender; 1-male; 2-female; 2
ETHNIC	2	B.Demography: Race: 1-Han; 99-others; 2
H_DIAB01	1	D.History: Diabetes; 0-No; 1-Yes; 1
H_AF01	1	D.History: Heart disease category: Atrial fibrillation(Including medical history and hospitalization diagnosis); 0-No; 1-Yes; 1
H_HYPT01	1	D.History: Hypertension; 0-No; 1-Yes; 1
H_LIPID01	1	D.History: Lipid metabolism disorders; 0-No; 1-Yes; 1
AI	1	history:Myocardial infarction; 0=NO; 1=YES; 1
H_DRINK_H01	1	D.History: Heavy Drinking(Alcohol consumption>=20g/day); 0-No,1-Yes; 1
H_SMK_C01	1	D.History: Current Smoking; 0-No,1-Yes; 1
IT	1	intravenous thrombolysis, 1=YES,0=NO 1
ET	1	动脉溶栓或机械取栓, 1=YES,0=NO 1
IMG_C_TOAST	5	K.Final diagnosis: cerebral infarction; Etiology according to TOAST system; 1-large artery atherosclerosis; 2-cardiogenic embolism; 3-small artery occlusion; 4-stroke of another determined cause; 5-stroke of an undetermined cause. 5
IMG_C_TOAST	4	K.Final diagnosis: cerebral infarction; Etiology according to TOAST system; 1-large artery atherosclerosis; 2-cardiogenic embolism; 3-small artery occlusion; 4-stroke of another determined cause; 5-stroke of an undetermined cause. 4
IMG_C_TOAST	3	K.Final diagnosis: cerebral infarction; Etiology according to TOAST system; 1-large artery atherosclerosis; 2-cardiogenic embolism; 3-small artery occlusion; 4-stroke of another determined cause; 5-stroke of an undetermined cause. 3
IMG_C_TOAST	2	K.Final diagnosis: cerebral infarction; Etiology according to TOAST system; 1-large artery atherosclerosis; 2-cardiogenic embolism; 3-small artery occlusion; 4-stroke of another determined cause; 5-stroke of an undetermined cause. 2
A_NIHSS		F.Admitting NIHSS: Total score;

BMI with y1_comb: interaction with stroke subtype

PHREG 过程

模型信息		
数据集	WORK.DATA2	
因变量	y1_comb_dd	N12.Follow-up events at 12 months: Days from onset to occurrence of combined vascular event;(day);
删失变量	y1_comb	N12.Follow-up events at 12 months:Occurrence of combined vascular event(including cardiovascular death,non-fatal stroke,non-fatal myocardial infarction):0-No;1-Yes;
删失值	0	
结值处理	BRESLOW	

读取的观测数	14146
使用的观测数	14146

分类水平信息						
分类	值	设计变量				
GENDER	2	1				
	1	0				
ETHNIC	2	1				
	1	0				
H_DIAB01	1	1				
	0	0				
H_AF01	1	1				
	0	0				
H_HYPT01	1	1				
	0	0				
H_LIPID01	1	1				
	0	0				
AI	1	1				
	0	0				
H_DRINK_H01	1	1				
	0	0				
H_SMK_C01	1	1				
	0	0				
IT	1	1				
	0	0				
ET	1	1				
	0	0				
IMG_C_TOAST	5	1	0	0	0	
	4	0	1	0	0	
	3	0	0	1	0	
	2	0	0	0	1	
	1	0	0	0	0	

BMI with y1_comb: interaction with stroke subtype

PHREG 过程

事件和删失值个数汇总			
合计	事件	删失	删失<br>百分比
14146	1505	12641	89.36

收敛状态
满足收敛准则 (GCONV=1E-8)。

模型拟合统计量		
准则	无<br>协变量	带<br>协变量
-2 LOG L	28553.433	28381.118
AIC	28553.433	28425.118
SBC	28553.433	28542.082

检验全局原假设: BETA=0			
检验	卡方	自由度	Pr > 卡方
似然比	172.3148	22	<.0001
评分	183.9692	22	<.0001
Wald	179.8270	22	<.0001

联合检验			
效应	自由度	Wald 卡方	Pr > 卡方
BMI	1	0.4225	0.5157
IMG_C_TOAST	4	1.3505	0.8527
BMI*IMG_C_TOAST	4	1.3354	0.8553
AGE	1	19.9575	<.0001
GENDER	1	0.2019	0.6532
ETHNIC	1	0.4008	0.5267
H_DIAB01	1	14.2189	0.0002
H_AF01	1	13.6514	0.0002
H_HYPT01	1	2.7328	0.0983
H_LIPID01	1	0.6353	0.4254
AI	1	2.1618	0.1415
H_DRINK_H01	1	1.3098	0.2524
H_SMK_C01	1	0.2642	0.6072
IT	1	0.2322	0.6299
ET	1	6.1164	0.0134
A_NIHSS	1	16.0911	<.0001

Note: Under full-rank parameterizations, Type 3 effect tests are replaced by joint tests. The joint test for an effect is a test that all of the parameters associated with that effect are zero. Such joint tests might not be equivalent to Type 3 effect tests under GLM parameterization.

BMI with y1_comb: interaction with stroke subtype

PHREG 过程

最大似然估计分析										
参数		自由度	参数估计	标准误差	卡方	Pr > 卡方	危险率	95% 危险率置信限	
BMI		1	0.00886	0.01363	0.4225	0.5157	.	.	.
IMG_C_TOAST	5	1	-0.38413	0.44460	0.7465	0.3876	.	.	.
IMG_C_TOAST	4	1	-0.40146	1.20663	0.1107	0.7393	.	.	.
IMG_C_TOAST	3	1	-0.33131	0.59918	0.3057	0.5803	.	.	.
IMG_C_TOAST	2	1	0.26850	0.75077	0.1279	0.7206	.	.	.
BMI*IMG_C_TOAST	5	1	0.00222	0.01779	0.0156	0.9007	.	.	.
BMI*IMG_C_TOAST	4	1	0.01513	0.04732	0.1022	0.7492	.	.	.
BMI*IMG_C_TOAST	3	1	-0.00799	0.02397	0.1110	0.7390	.	.	.
BMI*IMG_C_TOAST	2	1	-0.02859	0.03048	0.8800	0.3482	.	.	.
AGE		1	0.01118	0.00250	19.9575	<.0001	1.011	1.006	1.016
GENDER	2	1	0.02724	0.06062	0.2019	0.6532	1.028	0.912	1.157
ETHNIC	2	1	-0.10166	0.16059	0.4008	0.5267	0.903	0.659	1.237
H_DIAB01	1	1	0.22231	0.05896	14.2189	0.0002	1.249	1.113	1.402
H_AF01	1	1	0.42727	0.11564	13.6514	0.0002	1.533	1.222	1.923
H_HYPT01	1	1	0.09240	0.05589	2.7328	0.0983	1.097	0.983	1.224
H_LIPID01	1	1	-0.07949	0.09973	0.6353	0.4254	0.924	0.760	1.123
AI	1	1	0.23221	0.15794	2.1618	0.1415	1.261	0.926	1.719
H_DRINK_H01	1	1	0.09432	0.08241	1.3098	0.2524	1.099	0.935	1.292
H_SMK_C01	1	1	-0.03457	0.06724	0.2642	0.6072	0.966	0.847	1.102
IT	1	1	0.03943	0.08183	0.2322	0.6299	1.040	0.886	1.221
ET	1	1	0.61785	0.24983	6.1164	0.0134	1.855	1.137	3.027
A_NIHSS		1	0.02290	0.00571	16.0911	<.0001	1.023	1.012	1.035

BMI with y1_comb: interaction with stroke subtype

PHREG 过程

最大似然估计分析		
参数		标签
BMI		F.Physical examination: Body mass index (kg/m2);
IMG_C_TOAST	5	K.Final diagnosis: cerebral infarction; Etiology according to TOAST system; 1-large artery atherosclerosis; 2-cardiogenic embolism; 3-small artery occlusion; 4-stroke of another determined cause; 5-stroke of an undetermined cause. 5
IMG_C_TOAST	4	K.Final diagnosis: cerebral infarction; Etiology according to TOAST system; 1-large artery atherosclerosis; 2-cardiogenic embolism; 3-small artery occlusion; 4-stroke of another determined cause; 5-stroke of an undetermined cause. 4
IMG_C_TOAST	3	K.Final diagnosis: cerebral infarction; Etiology according to TOAST system; 1-large artery atherosclerosis; 2-cardiogenic embolism; 3-small artery occlusion; 4-stroke of another determined cause; 5-stroke of an undetermined cause. 3
IMG_C_TOAST	2	K.Final diagnosis: cerebral infarction; Etiology according to TOAST system; 1-large artery atherosclerosis; 2-cardiogenic embolism; 3-small artery occlusion; 4-stroke of another determined cause; 5-stroke of an undetermined cause. 2
BMI*IMG_C_TOAST	5	K.Final diagnosis: cerebral infarction; Etiology according to TOAST system; 1-large artery atherosclerosis; 2-cardiogenic embolism; 3-small artery occlusion; 4-stroke of another determined cause; 5-stroke of an undetermined cause. 5 * F.Physical examinatio
BMI*IMG_C_TOAST	4	K.Final diagnosis: cerebral infarction; Etiology according to TOAST system; 1-large artery atherosclerosis; 2-cardiogenic embolism; 3-small artery occlusion; 4-stroke of another determined cause; 5-stroke of an undetermined cause. 4 * F.Physical examinatio
BMI*IMG_C_TOAST	3	K.Final diagnosis: cerebral infarction; Etiology according to TOAST system; 1-large artery atherosclerosis; 2-cardiogenic embolism; 3-small artery occlusion; 4-stroke of another determined cause; 5-stroke of an undetermined cause. 3 * F.Physical examinatio
BMI*IMG_C_TOAST	2	K.Final diagnosis: cerebral infarction; Etiology according to TOAST system; 1-large artery atherosclerosis; 2-cardiogenic embolism; 3-small artery occlusion; 4-stroke of another determined cause; 5-stroke of an undetermined cause. 2 * F.Physical examinatio
AGE		A.Basic Information: Age (years old);
GENDER	2	A.Basic Information: Gender; 1-male; 2-female; 2
ETHNIC	2	B.Demography: Race: 1-Han; 99-others; 2
H_DIAB01	1	D.History: Diabetes; 0-No; 1-Yes; 1
H_AF01	1	D.History: Heart disease category: Atrial fibrillation(Including medical history and hospitalization diagnosis); 0-No; 1-Yes; 1
H_HYPT01	1	D.History: Hypertension; 0-No; 1-Yes; 1
H_LIPID01	1	D.History: Lipid metabolism disorders; 0-No; 1-Yes; 1
AI	1	history:Myocardial infarction; 0=NO; 1=YES; 1
H_DRINK_H01	1	D.History: Heavy Drinking(Alcohol consumption>=20g/day); 0-No,1-Yes; 1
H_SMK_C01	1	D.History: Current Smoking; 0-No,1-Yes; 1
IT	1	intravenous thrombolysis, 1=YES,0=NO 1
ET	1	动脉溶栓或机械取栓, 1=YES,0=NO 1
A_NIHSS		F.Admitting NIHSS: Total score;

FREQ 过程

N12.Follow-up events at 12 months: Recurrence of stroke: 0-No; 1-Yes;		
y1_stroke	频数	累积频数
0	12722	12722
1	1424	14146

等比例的卡方检验	
卡方	9023.3850
自由度	1
Pr > 卡方	<.0001

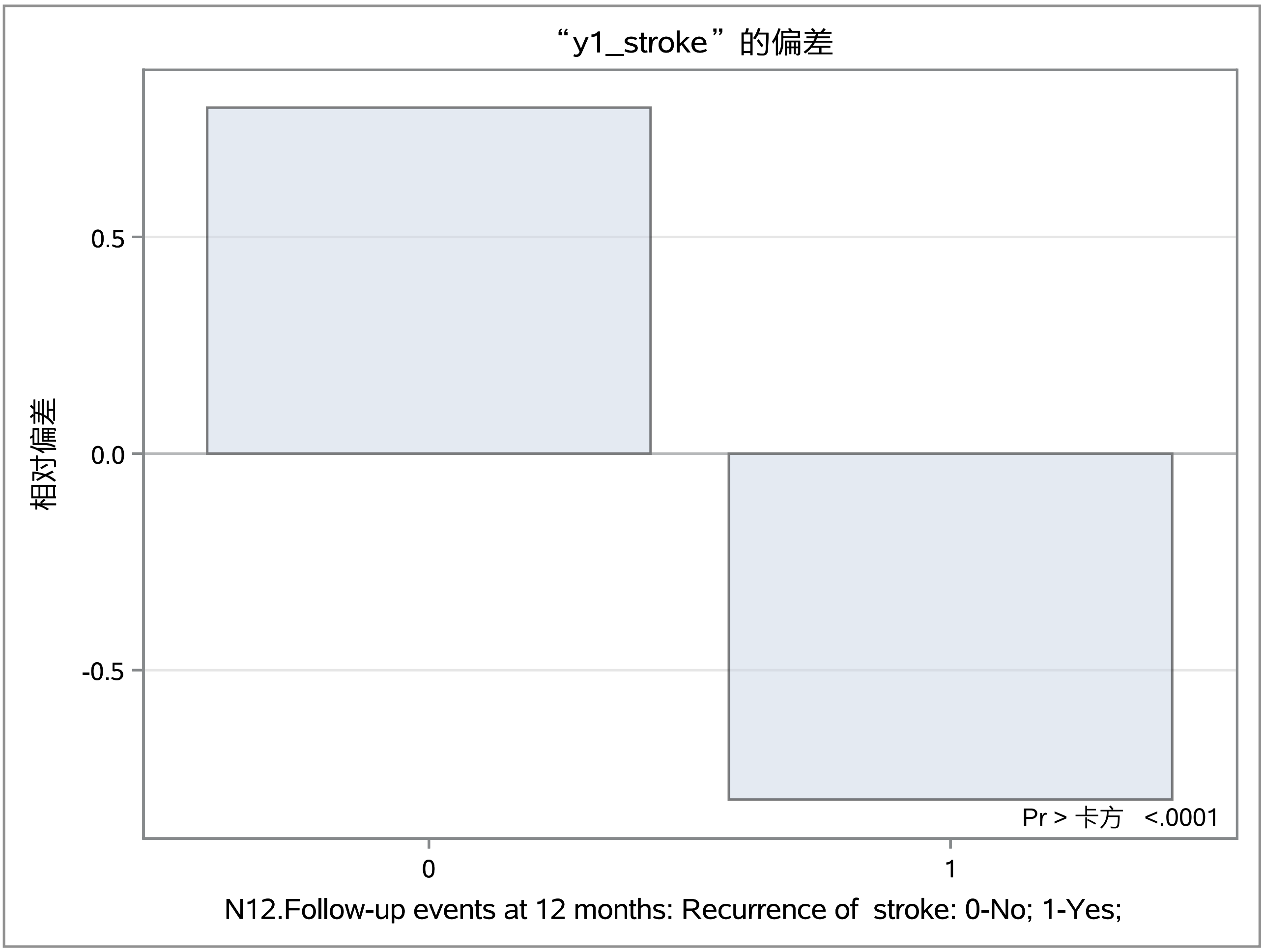

样本大小 = 14146

BMI with y1_stroke: crude model

PHREG 过程

模型信息		
数据集	WORK.DATA2	
因变量	y1_stroke_dd	N12.Follow-up events at 12 months: Days from onset to recurrence;(day);
删失变量	y1_stroke	N12.Follow-up events at 12 months: Recurrence of stroke: 0-No; 1-Yes;
删失值	0	
结值处理	BRESLOW	

读取的观测数<br>使用的观测数	14146<br>14146

事件和删失值个数汇总			
合计	事件	删失	删失百分比
14146	1424	12722	89.93

收敛状态
满足收敛准则 (GCONV=1E-8)。

模型拟合统计量		
准则	无协变量	带协变量
-2 LOG L	27024.892	27024.650
AIC	27024.892	27026.650
SBC	27024.892	27031.911

检验全局原假设: BETA=0			
检验	卡方	自由度	Pr > 卡方
似然比	0.2419	1	0.6228
评分	0.2429	1	0.6221
Wald	0.2457	1	0.6201

最大似然估计分析									
参数	自由度	参数估计	标准误差	卡方	Pr > 卡方	危险率	95% 危险率置信限		标签
BMI	1	0.00394	0.00795	0.2457	0.6201	1.004	0.988	1.020	F.Physical examination: Body mass index (kg/m2);

BMI with y1_stroke: adjusted model

PHREG 过程

模型信息		
数据集	WORK.DATA2	
因变量	y1_stroke_dd	N12.Follow-up events at 12 months: Days from onset to recurrence;(day);
删失变量	y1_stroke	N12.Follow-up events at 12 months: Recurrence of stroke: 0-No; 1-Yes;
删失值	0	
结值处理	BRESLOW	

读取的观测数<br>使用的观测数	14146<br>14146

分类水平信息					
分类	值	设计变量			
GENDER	2	1			
	1	0			
ETHNIC	2	1			
	1	0			
H_DIAB01	1	1			
	0	0			
H_AF01	1	1			
	0	0			
H_HYPT01	1	1			
	0	0			
H_LIPID01	1	1			
	0	0			
AI	1	1			
	0	0			
H_DRINK_H01	1	1			
	0	0			
H_SMK_C01	1	1			
	0	0			
IT	1	1			
	0	0			
ET	1	1			
	0	0			
IMG_C_TOAST	5	1	0	0	0
	4	0	1	0	0
	3	0	0	1	0
	2	0	0	0	1
	1	0	0	0	0

BMI with y1_stroke: adjusted model

PHREG 过程

事件和删失值个数汇总			
合计	事件	删失	删失百分比
14146	1424	12722	89.93

收敛状态
满足收敛准则 (GCONV=1E-8)。

模型拟合统计量		
准则	无协变量	带协变量
-2 LOG L	27024.892	26878.721
AIC	27024.892	26914.721
SBC	27024.892	27009.423

检验全局原假设: BETA=0			
检验	卡方	自由度	Pr > 卡方
似然比	146.1706	18	<.0001
评分	154.8140	18	<.0001
Wald	151.6134	18	<.0001

3 型检验			
效应	自由度	Wald 卡方	Pr > 卡方
BMI	1	1.5617	0.2114
AGE	1	15.4044	<.0001
GENDER	1	0.5341	0.4649
ETHNIC	1	0.5707	0.4500
H_DIAB01	1	11.7512	0.0006
H_AF01	1	8.5658	0.0034
H_HYPT01	1	1.2781	0.2583
H_LIPID01	1	0.5972	0.4396
AI	1	1.0132	0.3141
H_DRINK_H01	1	3.0215	0.0822
H_SMK_C01	1	0.7008	0.4025
IT	1	1.1319	0.2874
ET	1	5.9079	0.0151
IMG_C_TOAST	4	52.8403	<.0001
A_NIHSS	1	12.9654	0.0003

BMI with y1_stroke: adjusted model

PHREG 过程

最大似然估计分析

参数		自由度	参数估计	标准误差	卡方	Pr > 卡方	危险率	95% 危险率置信限	
BMI		1	0.01005	0.00804	1.5617	0.2114	1.010	0.994	1.026
AGE		1	0.01006	0.00256	15.4044	<.0001	1.010	1.005	1.015
GENDER	2	1	0.04557	0.06235	0.5341	0.4649	1.047	0.926	1.183
ETHNIC	2	1	-0.12608	0.16689	0.5707	0.4500	0.882	0.636	1.223
H_DIAB01	1	1	0.20819	0.06073	11.7512	0.0006	1.231	1.093	1.387
H_AF01	1	1	0.35694	0.12196	8.5658	0.0034	1.429	1.125	1.815
H_HYPT01	1	1	0.06470	0.05723	1.2781	0.2583	1.067	0.954	1.193
H_LIPID01	1	1	-0.07924	0.10253	0.5972	0.4396	0.924	0.756	1.129
AI	1	1	0.16913	0.16802	1.0132	0.3141	1.184	0.852	1.646
H_DRINK_H01	1	1	0.14553	0.08372	3.0215	0.0822	1.157	0.982	1.363
H_SMK_C01	1	1	-0.05793	0.06920	0.7008	0.4025	0.944	0.824	1.081
IT	1	1	0.08837	0.08306	1.1319	0.2874	1.092	0.928	1.286
ET	1	1	0.62569	0.25742	5.9079	0.0151	1.870	1.129	3.096
IMG_C_TOAST	5	1	-0.33713	0.06298	28.6538	<.0001	0.714	0.631	0.808
IMG_C_TOAST	4	1	-0.03587	0.22878	0.0246	0.8754	0.965	0.616	1.511
IMG_C_TOAST	3	1	-0.52847	0.08089	42.6836	<.0001	0.590	0.503	0.691
IMG_C_TOAST	2	1	-0.44750	0.14207	9.9214	0.0016	0.639	0.484	0.844
A_NIHSS		1	0.02135	0.00593	12.9654	0.0003	1.022	1.010	1.034

BMI with y1_stroke: adjusted model

PHREG 过程

最大似然估计分析		
参数		标签
BMI		F.Physical examination: Body mass index (kg/m2);
AGE		A.Basic Information: Age (years old);
GENDER	2	A.Basic Information: Gender; 1-male; 2-female; 2
ETHNIC	2	B.Demography: Race: 1-Han; 99-others; 2
H_DIAB01	1	D.History: Diabetes; 0-No; 1-Yes; 1
H_AF01	1	D.History: Heart disease category: Atrial fibrillation(Including medical history and hospitalization diagnosis); 0-No; 1-Yes; 1
H_HYPT01	1	D.History: Hypertension; 0-No; 1-Yes; 1
H_LIPID01	1	D.History: Lipid metabolism disorders; 0-No; 1-Yes; 1
AI	1	history:Myocardial infarction; 0=NO; 1=YES; 1
H_DRINK_H01	1	D.History: Heavy Drinking(Alcohol consumption>=20g/day); 0-No,1-Yes; 1
H_SMK_C01	1	D.History: Current Smoking; 0-No,1-Yes; 1
IT	1	intravenous thrombolysis, 1=YES,0=NO 1
ET	1	动脉溶栓或机械取栓, 1=YES,0=NO 1
IMG_C_TOAST	5	K.Final diagnosis: cerebral infarction; Etiology according to TOAST system; 1-large artery atherosclerosis; 2-cardiogenic embolism; 3-small artery occlusion; 4-stroke of another determined cause; 5-stroke of an undetermined cause. 5
IMG_C_TOAST	4	K.Final diagnosis: cerebral infarction; Etiology according to TOAST system; 1-large artery atherosclerosis; 2-cardiogenic embolism; 3-small artery occlusion; 4-stroke of another determined cause; 5-stroke of an undetermined cause. 4
IMG_C_TOAST	3	K.Final diagnosis: cerebral infarction; Etiology according to TOAST system; 1-large artery atherosclerosis; 2-cardiogenic embolism; 3-small artery occlusion; 4-stroke of another determined cause; 5-stroke of an undetermined cause. 3
IMG_C_TOAST	2	K.Final diagnosis: cerebral infarction; Etiology according to TOAST system; 1-large artery atherosclerosis; 2-cardiogenic embolism; 3-small artery occlusion; 4-stroke of another determined cause; 5-stroke of an undetermined cause. 2
A_NIHSS		F.Admitting NIHSS: Total score;

BMI with y1_stroke: interaction with stroke subtype

PHREG 过程

模型信息		
数据集	WORK.DATA2	
因变量	y1_stroke_dd	N12.Follow-up events at 12 months: Days from onset to recurrence;(day);
删失变量	y1_stroke	N12.Follow-up events at 12 months: Recurrence of stroke: 0-No; 1-Yes;
删失值	0	
结值处理	BRESLOW	

读取的观测数<br>使用的观测数	14146<br>14146

分类水平信息					
分类	值	设计变量			
GENDER	2	1			
	1	0			
ETHNIC	2	1			
	1	0			
H_DIAB01	1	1			
	0	0			
H_AF01	1	1			
	0	0			
H_HYPT01	1	1			
	0	0			
H_LIPID01	1	1			
	0	0			
AI	1	1			
	0	0			
H_DRINK_H01	1	1			
	0	0			
H_SMK_C01	1	1			
	0	0			
IT	1	1			
	0	0			
ET	1	1			
	0	0			
IMG_C_TOAST	5	1	0	0	0
	4	0	1	0	0
	3	0	0	1	0
	2	0	0	0	1
	1	0	0	0	0

BMI with y1_stroke: interaction with stroke subtype

PHREG 过程

事件和删失值个数汇总			
合计	事件	删失	删失百分比
14146	1424	12722	89.93

收敛状态
满足收敛准则 (GCONV=1E-8)。

模型拟合统计量		
准则	无协变量	带协变量
-2 LOG L	27024.892	26878.018
AIC	27024.892	26922.018
SBC	27024.892	27037.765

检验全局原假设: BETA=0			
检验	卡方	自由度	Pr > 卡方
似然比	146.8743	22	<.0001
评分	155.7789	22	<.0001
Wald	152.5498	22	<.0001

联合检验			
效应	自由度	Wald 卡方	Pr > 卡方
BMI	1	0.7083	0.4000
IMG_C_TOAST	4	0.9243	0.9211
BMI*IMG_C_TOAST	4	0.7120	0.9498
AGE	1	15.3961	<.0001
GENDER	1	0.5247	0.4688
ETHNIC	1	0.5651	0.4522
H_DIAB01	1	11.8983	0.0006
H_AF01	1	8.6321	0.0033
H_HYPT01	1	1.3082	0.2527
H_LIPID01	1	0.5997	0.4387
AI	1	1.0300	0.3102
H_DRINK_H01	1	3.0327	0.0816
H_SMK_C01	1	0.6977	0.4036
IT	1	1.0969	0.2949
ET	1	5.9700	0.0146
A_NIHSS	1	12.8188	0.0003

Note: Under full-rank parameterizations, Type 3 effect tests are replaced by joint tests. The joint test for an effect is a test that all of the parameters associated with that effect are zero. Such joint tests might not be equivalent to Type 3 effect tests under GLM parameterization.

BMI with y1_stroke: interaction with stroke subtype

PHREG 过程

最大似然估计分析									
参数		自由度	参数估计	标准误差	卡方	Pr > 卡方	危险率	95% 危险率置信限	
BMI		1	0.01168	0.01388	0.7083	0.4000	.	.	.
IMG_C_TOAST	5	1	-0.34848	0.45517	0.5862	0.4439	.	.	.
IMG_C_TOAST	4	1	-0.50067	1.20362	0.1730	0.6774	.	.	.
IMG_C_TOAST	3	1	-0.39598	0.60943	0.4222	0.5158	.	.	.
IMG_C_TOAST	2	1	0.05054	0.78756	0.0041	0.9488	.	.	.
BMI*IMG_C_TOAST	5	1	0.0004588	0.01819	0.0006	0.9799	.	.	.
BMI*IMG_C_TOAST	4	1	0.01860	0.04693	0.1571	0.6918	.	.	.
BMI*IMG_C_TOAST	3	1	-0.00534	0.02433	0.0482	0.8262	.	.	.
BMI*IMG_C_TOAST	2	1	-0.02047	0.03186	0.4129	0.5205	.	.	.
AGE		1	0.01006	0.00256	15.3961	<.0001	1.010	1.005	1.015
GENDER	2	1	0.04517	0.06236	0.5247	0.4688	1.046	0.926	1.182
ETHNIC	2	1	-0.12547	0.16691	0.5651	0.4522	0.882	0.636	1.223
H_DIAB01	1	1	0.20959	0.06076	11.8983	0.0006	1.233	1.095	1.389
H_AF01	1	1	0.35841	0.12199	8.6321	0.0033	1.431	1.127	1.818
H_HYPT01	1	1	0.06548	0.05725	1.3082	0.2527	1.068	0.954	1.194
H_LIPID01	1	1	-0.07940	0.10253	0.5997	0.4387	0.924	0.756	1.129
AI	1	1	0.17052	0.16802	1.0300	0.3102	1.186	0.853	1.648
H_DRINK_H01	1	1	0.14583	0.08374	3.0327	0.0816	1.157	0.982	1.363
H_SMK_C01	1	1	-0.05782	0.06923	0.6977	0.4036	0.944	0.824	1.081
IT	1	1	0.08704	0.08311	1.0969	0.2949	1.091	0.927	1.284
ET	1	1	0.62924	0.25753	5.9700	0.0146	1.876	1.133	3.108
A_NIHSS		1	0.02124	0.00593	12.8188	0.0003	1.021	1.010	1.033

BMI with y1_stroke: interaction with stroke subtype

PHREG 过程

最大似然估计分析		
参数		标签
BMI		F.Physical examination: Body mass index (kg/m2);
IMG_C_TOAST	5	K.Final diagnosis: cerebral infarction; Etiology according to TOAST system; 1-large artery atherosclerosis; 2-cardiogenic embolism; 3-small artery occlusion; 4-stroke of another determined cause; 5-stroke of an undetermined cause. 5
IMG_C_TOAST	4	K.Final diagnosis: cerebral infarction; Etiology according to TOAST system; 1-large artery atherosclerosis; 2-cardiogenic embolism; 3-small artery occlusion; 4-stroke of another determined cause; 5-stroke of an undetermined cause. 4
IMG_C_TOAST	3	K.Final diagnosis: cerebral infarction; Etiology according to TOAST system; 1-large artery atherosclerosis; 2-cardiogenic embolism; 3-small artery occlusion; 4-stroke of another determined cause; 5-stroke of an undetermined cause. 3
IMG_C_TOAST	2	K.Final diagnosis: cerebral infarction; Etiology according to TOAST system; 1-large artery atherosclerosis; 2-cardiogenic embolism; 3-small artery occlusion; 4-stroke of another determined cause; 5-stroke of an undetermined cause. 2
BMI*IMG_C_TOAST	5	K.Final diagnosis: cerebral infarction; Etiology according to TOAST system; 1-large artery atherosclerosis; 2-cardiogenic embolism; 3-small artery occlusion; 4-stroke of another determined cause; 5-stroke of an undetermined cause. 5 * F.Physical examinatio
BMI*IMG_C_TOAST	4	K.Final diagnosis: cerebral infarction; Etiology according to TOAST system; 1-large artery atherosclerosis; 2-cardiogenic embolism; 3-small artery occlusion; 4-stroke of another determined cause; 5-stroke of an undetermined cause. 4 * F.Physical examinatio
BMI*IMG_C_TOAST	3	K.Final diagnosis: cerebral infarction; Etiology according to TOAST system; 1-large artery atherosclerosis; 2-cardiogenic embolism; 3-small artery occlusion; 4-stroke of another determined cause; 5-stroke of an undetermined cause. 3 * F.Physical examinatio
BMI*IMG_C_TOAST	2	K.Final diagnosis: cerebral infarction; Etiology according to TOAST system; 1-large artery atherosclerosis; 2-cardiogenic embolism; 3-small artery occlusion; 4-stroke of another determined cause; 5-stroke of an undetermined cause. 2 * F.Physical examinatio
AGE		A.Basic Information: Age (years old);
GENDER	2	A.Basic Information: Gender; 1-male; 2-female; 2
ETHNIC	2	B.Demography: Race: 1-Han; 99-others; 2
H_DIAB01	1	D.History: Diabetes; 0-No; 1-Yes; 1
H_AF01	1	D.History: Heart disease category: Atrial fibrillation(Including medical history and hospitalization diagnosis); 0-No; 1-Yes; 1
H_HYPT01	1	D.History: Hypertension; 0-No; 1-Yes; 1
H_LIPID01	1	D.History: Lipid metabolism disorders; 0-No; 1-Yes; 1
AI	1	history:Myocardial infarction; 0=NO; 1=YES; 1
H_DRINK_H01	1	D.History: Heavy Drinking(Alcohol consumption>=20g/day); 0-No,1-Yes; 1
H_SMK_C01	1	D.History: Current Smoking; 0-No,1-Yes; 1
IT	1	intravenous thrombolysis, 1=YES,0=NO 1
ET	1	动脉溶栓或机械取栓, 1=YES,0=NO 1
A_NIHSS		F.Admitting NIHSS: Total score;

BMI_g with y1_death: Descriptive results

FREQ 过程

频数
行百分比

BMI_g-y1_death表			
	y1_death(N12.Follow-up events at 12 months: Whether the patient died: 0-survival;1-death;)		
BMI_g(1=<18.5;2=18.5-<23;3=23-<27.5;4= ≥ 27.5)	0	1	合计
1	277<br>89.64	32<br>10.36	309
2	3693<br>95.80	162<br>4.20	3855
3	7256<br>96.86	235<br>3.14	7491
4	2434<br>97.71	57<br>2.29	2491
合计	13660	486	14146

表“y1_death-BMI_g”的统计量

统计量	自由度	值	概率
卡方	3	63.3343	<.0001
似然比卡方检验	3	49.0477	<.0001
Mantel-Haenszel 卡方	1	42.0154	<.0001
Phi 系数		0.0669	
列联系数		0.0668	
Cramer V		0.0669	

样本大小 = 14146

BMI_g with y1_death: crude model

PHREG 过程

模型信息		
数据集	WORK.DATA2	
因变量	y1_death_dd	N12.Follow-up events at 12 months: Days from onset to death;(day);
删失变量	y1_death	N12.Follow-up events at 12 months: Whether the patient died: 0-survival;1-death;
删失值	0	
结值处理	BRESLOW	

读取的观测数<br>使用的观测数	14146<br>14146

分类水平信息				
分类	值	设计变量		
BMI_g	4	1	0	0
	3	0	1	0
	2	0	0	0
	1	0	0	1

事件和删失值个数汇总			
合计	事件	删失	删失<br>百分比
14146	486	13660	96.56

收敛状态
满足收敛准则 (GCONV=1E-8)。

模型拟合统计量		
准则	无<br>协变量	带<br>协变量
-2 LOG L	9255.945	9206.661
AIC	9255.945	9212.661
SBC	9255.945	9225.220

检验全局原假设: BETA=0			
检验	卡方	自由度	Pr > 卡方
似然比	49.2834	3	<.0001
评分	65.3278	3	<.0001
Wald	59.2052	3	<.0001

3 型检验			
效应	自由度	Wald 卡方	Pr > 卡方
BMI_g	3	59.2052	<.0001

BMI_g with y1_death: crude model

PHREG 过程

最大似然估计分析

参数		自由度	参数估计	标准误差	卡方	Pr > 卡方	危险率	95% 危险率置信限		标签
BMI_g	4	1	-0.61958	0.15400	16.1856	<.0001	0.538	0.398	0.728	1=<18.5;2=18.5-<23;3=23-<27.5;4=≥27.5 4
BMI_g	3	1	-0.29799	0.10212	8.5151	0.0035	0.742	0.608	0.907	1=<18.5;2=18.5-<23;3=23-<27.5;4=≥27.5 3
BMI_g	1	1	0.93748	0.19342	23.4920	<.0001	2.554	1.748	3.731	1=<18.5;2=18.5-<23;3=23-<27.5;4=≥27.5 1

BMI_g with y1_death: adjusted model

PHREG 过程

模型信息		
数据集	WORK.DATA2	
因变量	y1_death_dd	N12.Follow-up events at 12 months: Days from onset to death;(day);
删失变量	y1_death	N12.Follow-up events at 12 months: Whether the patient died: 0-survival;1-death;
删失值	0	
结值处理	BRESLOW	

读取的观测数<br>使用的观测数	14146<br>14146

分类水平信息					
分类	值	设计变量			
BMI_g	4	1	0	0	
	3	0	1	0	
	2	0	0	0	
	1	0	0	1	
GENDER	2	1			
	1	0			
ETHNIC	2	1			
	1	0			
H_DIAB01	1	1			
	0	0			
H_AF01	1	1			
	0	0			
H_HYPT01	1	1			
	0	0			
H_LIPID01	1	1			
	0	0			
AI	1	1			
	0	0			
H_DRINK_H01	1	1			
	0	0			
H_SMK_C01	1	1			
	0	0			
IT	1	1			
	0	0			
ET	1	1			
	0	0			
IMG_C_TOAST	5	1	0	0	0
	4	0	1	0	0
	3	0	0	1	0

BMI_g with y1_death: adjusted model

PHREG 过程

分类水平信息					
分类	值	设计变量			
	2	0	0	0	1
	1	0	0	0	0

事件和删失值个数汇总			
合计	事件	删失	删失百分比
14146	486	13660	96.56

收敛状态
满足收敛准则 (GCONV=1E-8)。

模型拟合统计量		
准则	无协变量	带协变量
-2 LOG L	9255.945	8609.158
AIC	9255.945	8649.158
SBC	9255.945	8732.882

检验全局原假设: BETA=0			
检验	卡方	自由度	Pr > 卡方
似然比	646.7864	20	<.0001
评分	880.9333	20	<.0001
Wald	756.7556	20	<.0001

3 型检验			
效应	自由度	Wald 卡方	Pr > 卡方
BMI_g	3	12.7663	0.0052
AGE	1	149.4314	<.0001
GENDER	1	1.5673	0.2106
ETHNIC	1	3.2674	0.0707
H_DIAB01	1	12.7146	0.0004
H_AF01	1	29.5599	<.0001
H_HYPT01	1	0.1454	0.7030
H_LIPID01	1	2.2506	0.1336
AI	1	5.3049	0.0213
H_DRINK_H01	1	6.8623	0.0088
H_SMK_C01	1	0.9070	0.3409
IT	1	19.3864	<.0001
ET	1	7.5416	0.0060
IMG_C_TOAST	4	34.5453	<.0001
A_NIHSS	1	226.8725	<.0001

BMI_g with y1_death: adjusted model

PHREG 过程

最大似然估计分析									
参数		自由度	参数估计	标准误差	卡方	Pr > 卡方	危险率	95% 危险率置信限	
BMI_g	4	1	-0.27722	0.15753	3.0967	0.0784	0.758	0.557	1.032
BMI_g	3	1	-0.05819	0.10459	0.3096	0.5779	0.943	0.769	1.158
BMI_g	1	1	0.52825	0.19572	7.2845	0.0070	1.696	1.156	2.489
AGE		1	0.05880	0.00481	149.4314	<.0001	1.061	1.051	1.071
GENDER	2	1	-0.12840	0.10256	1.5673	0.2106	0.880	0.719	1.075
ETHNIC	2	1	0.42606	0.23571	3.2674	0.0707	1.531	0.965	2.430
H_DIAB01	1	1	0.37200	0.10433	12.7146	0.0004	1.451	1.182	1.780
H_AF01	1	1	0.81129	0.14922	29.5599	<.0001	2.251	1.680	3.015
H_HYPT01	1	1	0.03727	0.09775	0.1454	0.7030	1.038	0.857	1.257
H_LIPID01	1	1	-0.31171	0.20778	2.2506	0.1336	0.732	0.487	1.100
AI	1	1	0.52128	0.22633	5.3049	0.0213	1.684	1.081	2.624
H_DRINK_H01	1	1	-0.51293	0.19581	6.8623	0.0088	0.599	0.408	0.879
H_SMK_C01	1	1	0.11831	0.12423	0.9070	0.3409	1.126	0.882	1.436
IT	1	1	-0.73166	0.16617	19.3864	<.0001	0.481	0.347	0.666
ET	1	1	0.86007	0.31319	7.5416	0.0060	2.363	1.279	4.366
IMG_C_TOAST	5	1	-0.23944	0.11135	4.6235	0.0315	0.787	0.633	0.979
IMG_C_TOAST	4	1	0.63699	0.32733	3.7871	0.0516	1.891	0.995	3.591
IMG_C_TOAST	3	1	-0.92982	0.18259	25.9309	<.0001	0.395	0.276	0.564
IMG_C_TOAST	2	1	-0.42458	0.19355	4.8122	0.0283	0.654	0.448	0.956
A_NIHSS		1	0.09504	0.00631	226.8725	<.0001	1.100	1.086	1.113

BMI_g with y1_death: adjusted model

PHREG 过程

最大似然估计分析		
参数		标签
BMI_g	4	1=<18.5;2=18.5-<23;3=23-<27.5;4= ≥ 27.5 4
BMI_g	3	1=<18.5;2=18.5-<23;3=23-<27.5;4= ≥ 27.5 3
BMI_g	1	1=<18.5;2=18.5-<23;3=23-<27.5;4= ≥ 27.5 1
AGE		A.Basic Information: Age (years old);
GENDER	2	A.Basic Information: Gender; 1-male; 2-female; 2
ETHNIC	2	B.Demography: Race: 1-Han; 99-others; 2
H_DIAB01	1	D.History: Diabetes; 0-No; 1-Yes; 1
H_AF01	1	D.History: Heart disease category: Atrial fibrillation(Including medical history and hospitalization diagnosis); 0-No; 1-Yes; 1
H_HYPT01	1	D.History: Hypertension; 0-No; 1-Yes; 1
H_LIPID01	1	D.History: Lipid metabolism disorders; 0-No; 1-Yes; 1
AI	1	history:Myocardial infarction; 0=NO; 1=YES; 1
H_DRINK_H01	1	D.History: Heavy Drinking(Alcohol consumption>=20g/day); 0-No,1-Yes; 1
H_SMK_C01	1	D.History: Current Smoking; 0-No,1-Yes; 1
IT	1	intravenous thrombolysis, 1=YES,0=NO 1
ET	1	动脉溶栓或机械取栓, 1=YES,0=NO 1
IMG_C_TOAST	5	K.Final diagnosis: cerebral infarction; Etiology according to TOAST system; 1-large artery atherosclerosis; 2-cardiogenic embolism; 3-small artery occlusion; 4-stroke of another determined cause; 5-stroke of an undetermined cause. 5
IMG_C_TOAST	4	K.Final diagnosis: cerebral infarction; Etiology according to TOAST system; 1-large artery atherosclerosis; 2-cardiogenic embolism; 3-small artery occlusion; 4-stroke of another determined cause; 5-stroke of an undetermined cause. 4
IMG_C_TOAST	3	K.Final diagnosis: cerebral infarction; Etiology according to TOAST system; 1-large artery atherosclerosis; 2-cardiogenic embolism; 3-small artery occlusion; 4-stroke of another determined cause; 5-stroke of an undetermined cause. 3
IMG_C_TOAST	2	K.Final diagnosis: cerebral infarction; Etiology according to TOAST system; 1-large artery atherosclerosis; 2-cardiogenic embolism; 3-small artery occlusion; 4-stroke of another determined cause; 5-stroke of an undetermined cause. 2
A_NIHSS		F.Admitting NIHSS: Total score;

BMI_g with y1_death: interaction with stroke subtype

PHREG 过程

模型信息		
数据集	WORK.DATA2	
因变量	y1_death_dd	N12.Follow-up events at 12 months: Days from onset to death;(day);
删失变量	y1_death	N12.Follow-up events at 12 months: Whether the patient died: 0-survival;1-death;
删失值	0	
结值处理	BRESLOW	

读取的观测数<br>使用的观测数	14146<br>14146

分类水平信息						
分类	值	设计变量				
BMI_g	4	1	0	0	
	3	0	1	0	
	2	0	0	0	
	1	0	0	1	
GENDER	2	1			
	1	0			
ETHNIC	2	1			
	1	0			
H_DIAB01	1	1			
	0	0			
H_AF01	1	1			
	0	0			
H_HYPT01	1	1			
	0	0			
H_LIPID01	1	1			
	0	0			
AI	1	1			
	0	0			
H_DRINK_H01	1	1			
	0	0			
H_SMK_C01	1	1			
	0	0			
IT	1	1			
	0	0			
ET	1	1			
	0	0			
IMG_C_TOAST	5	1	0	0	0
	4	0	1	0	0
	3	0	0	1	0

BMI_g with y1_death: interaction with stroke subtype

PHREG 过程

分类水平信息					
分类	值	设计变量			
	2	0	0	0	1
	1	0	0	0	0

事件和删失值个数汇总			
合计	事件	删失	删失百分比
14146	486	13660	96.56

收敛状态
满足收敛准则 (GCONV=1E-8)。

模型拟合统计量		
准则	无协变量	带协变量
-2 LOG L	9255.945	8596.529
AIC	9255.945	8660.529
SBC	9255.945	8794.488

检验全局原假设: BETA=0			
检验	卡方	自由度	Pr > 卡方
似然比	659.4158	32	<.0001
评分	930.1004	32	<.0001
Wald	782.0611	32	<.0001

联合检验			
效应	自由度	Wald 卡方	Pr > 卡方
BMI_g	3	2.0299	0.5662
IMG_C_TOAST	4	12.0785	0.0168
BMI_g*IMG_C_TOAST	12	9.3236	0.6751
AGE	1	148.5169	<.0001
GENDER	1	1.4170	0.2339
ETHNIC	1	2.9233	0.0873
H_DIAB01	1	12.0620	0.0005
H_AF01	1	28.9862	<.0001
H_HYPT01	1	0.1731	0.6774
H_LIPID01	1	2.2039	0.1377
AI	1	5.2080	0.0225
H_DRINK_H01	1	7.1889	0.0073
H_SMK_C01	1	1.1136	0.2913
IT	1	19.0182	<.0001

BMI_g with y1_death: interaction with stroke subtype

PHREG 过程

联合检验			
效应	自由度	Wald 卡方	Pr > 卡方
ET	1	8.3354	0.0039
A_NIHSS	1	220.1983	<.0001

Note: Under full-rank parameterizations, Type 3 effect tests are replaced by joint tests. The joint test for an effect is a test that all of the parameters associated with that effect are zero. Such joint tests might not be equivalent to Type 3 effect tests under GLM parameterization.

BMI_g with y1_death: interaction with stroke subtype

PHREG 过程

最大似然估计分析										
参数			自由度	参数估计	标准误差	卡方	Pr > 卡方	危险率	95% 危险率置信限	
BMI_g	4		1	-0.04588	0.27137	0.0286	0.8657	.	.	.
BMI_g	3		1	0.21351	0.18707	1.3027	0.2537	.	.	.
BMI_g	1		1	-0.06273	0.52370	0.0143	0.9047	.	.	.
IMG_C_TOAST	5		1	-0.05686	0.19449	0.0855	0.7700	.	.	.
IMG_C_TOAST	4		1	1.09982	0.52338	4.4159	0.0356	.	.	.
IMG_C_TOAST	3		1	-0.83143	0.33808	6.0480	0.0139	.	.	.
IMG_C_TOAST	2		1	-0.10192	0.27176	0.1407	0.7076	.	.	.
BMI_g*IMG_C_TOAST	4	5	1	-0.35827	0.36053	0.9875	0.3204	.	.	.
BMI_g*IMG_C_TOAST	4	4	1	-11.36569	205.58006	0.0031	0.9559	.	.	.
BMI_g*IMG_C_TOAST	4	3	1	-0.00223	0.57510	0.0000	0.9969	.	.	.
BMI_g*IMG_C_TOAST	4	2	1	-0.28109	0.48652	0.3338	0.5634	.	.	.
BMI_g*IMG_C_TOAST	3	5	1	-0.34549	0.24040	2.0653	0.1507	.	.	.
BMI_g*IMG_C_TOAST	3	4	1	-0.79411	0.73163	1.1781	0.2777	.	.	.
BMI_g*IMG_C_TOAST	3	3	1	-0.27444	0.42208	0.4228	0.5156	.	.	.
BMI_g*IMG_C_TOAST	3	2	1	-0.58514	0.33607	3.0314	0.0817	.	.	.
BMI_g*IMG_C_TOAST	1	5	1	0.78777	0.57901	1.8511	0.1737	.	.	.
BMI_g*IMG_C_TOAST	1	4	1	0.99052	1.02631	0.9315	0.3345	.	.	.
BMI_g*IMG_C_TOAST	1	3	1	0.92124	0.93064	0.9799	0.3222	.	.	.
BMI_g*IMG_C_TOAST	1	2	1	0.05087	0.80321	0.0040	0.9495	.	.	.
AGE			1	0.05886	0.00483	148.5169	<.0001	1.061	1.051	1.071
GENDER	2		1	-0.12227	0.10272	1.4170	0.2339	0.885	0.724	1.082
ETHNIC	2		1	0.41210	0.24102	2.9233	0.0873	1.510	0.941	2.422
H_DIAB01	1		1	0.36322	0.10458	12.0620	0.0005	1.438	1.171	1.765
H_AF01	1		1	0.80434	0.14940	28.9862	<.0001	2.235	1.668	2.996
H_HYPT01	1		1	0.04081	0.09810	0.1731	0.6774	1.042	0.859	1.262

BMI_g with y1_death: interaction with stroke subtype

PHREG 过程

最大似然估计分析

参数			标签
BMI_g	4		1=<18.5;2=18.5-<23;3=23-<27.5;4=≥27.5 4
BMI_g	3		1=<18.5;2=18.5-<23;3=23-<27.5;4=≥27.5 3
BMI_g	1		1=<18.5;2=18.5-<23;3=23-<27.5;4=≥27.5 1
IMG_C_TOAST	5		K.Final diagnosis: cerebral infarction; Etiology according to TOAST system; 1-large artery atherosclerosis; 2-cardiogenic embolism; 3-small artery occlusion; 4-stroke of another determined cause; 5-stroke of an undetermined cause. 5
IMG_C_TOAST	4		K.Final diagnosis: cerebral infarction; Etiology according to TOAST system; 1-large artery atherosclerosis; 2-cardiogenic embolism; 3-small artery occlusion; 4-stroke of another determined cause; 5-stroke of an undetermined cause. 4
IMG_C_TOAST	3		K.Final diagnosis: cerebral infarction; Etiology according to TOAST system; 1-large artery atherosclerosis; 2-cardiogenic embolism; 3-small artery occlusion; 4-stroke of another determined cause; 5-stroke of an undetermined cause. 3
IMG_C_TOAST	2		K.Final diagnosis: cerebral infarction; Etiology according to TOAST system; 1-large artery atherosclerosis; 2-cardiogenic embolism; 3-small artery occlusion; 4-stroke of another determined cause; 5-stroke of an undetermined cause. 2
BMI_g*IMG_C_TOAST	4	5	1=<18.5;2=18.5-<23;3=23-<27.5;4=≥27.5 4 * K.Final diagnosis: cerebral infarction; Etiology according to TOAST system; 1-large artery atherosclerosis; 2-cardiogenic embolism; 3-small artery occlusion; 4-stroke of another determined cause; 5-stroke of an un
BMI_g*IMG_C_TOAST	4	4	1=<18.5;2=18.5-<23;3=23-<27.5;4=≥27.5 4 * K.Final diagnosis: cerebral infarction; Etiology according to TOAST system; 1-large artery atherosclerosis; 2-cardiogenic embolism; 3-small artery occlusion; 4-stroke of another determined cause; 5-stroke of an un
BMI_g*IMG_C_TOAST	4	3	1=<18.5;2=18.5-<23;3=23-<27.5;4=≥27.5 4 * K.Final diagnosis: cerebral infarction; Etiology according to TOAST system; 1-large artery atherosclerosis; 2-cardiogenic embolism; 3-small artery occlusion; 4-stroke of another determined cause; 5-stroke of an un
BMI_g*IMG_C_TOAST	4	2	1=<18.5;2=18.5-<23;3=23-<27.5;4=≥27.5 4 * K.Final diagnosis: cerebral infarction; Etiology according to TOAST system; 1-large artery atherosclerosis; 2-cardiogenic embolism; 3-small artery occlusion; 4-stroke of another determined cause; 5-stroke of an un
BMI_g*IMG_C_TOAST	3	5	1=<18.5;2=18.5-<23;3=23-<27.5;4=≥27.5 3 * K.Final diagnosis: cerebral infarction; Etiology according to TOAST system; 1-large artery atherosclerosis; 2-cardiogenic embolism; 3-small artery occlusion; 4-stroke of another determined cause; 5-stroke of an un
BMI_g*IMG_C_TOAST	3	4	1=<18.5;2=18.5-<23;3=23-<27.5;4=≥27.5 3 * K.Final diagnosis: cerebral infarction; Etiology according to TOAST system; 1-large artery atherosclerosis; 2-cardiogenic embolism; 3-small artery occlusion; 4-stroke of another determined cause; 5-stroke of an un
BMI_g*IMG_C_TOAST	3	3	1=<18.5;2=18.5-<23;3=23-<27.5;4=≥27.5 3 * K.Final diagnosis: cerebral infarction; Etiology according to TOAST system; 1-large artery atherosclerosis; 2-cardiogenic embolism; 3-small artery occlusion; 4-stroke of another determined cause; 5-stroke of an un
BMI_g*IMG_C_TOAST	3	2	1=<18.5;2=18.5-<23;3=23-<27.5;4=≥27.5 3 * K.Final diagnosis: cerebral infarction; Etiology according to TOAST system; 1-large artery atherosclerosis; 2-cardiogenic embolism; 3-small artery occlusion; 4-stroke of another determined cause; 5-stroke of an un
BMI_g*IMG_C_TOAST	1	5	1=<18.5;2=18.5-<23;3=23-<27.5;4=≥27.5 1 * K.Final diagnosis: cerebral infarction; Etiology according to TOAST system; 1-large artery atherosclerosis; 2-cardiogenic embolism; 3-small artery occlusion; 4-stroke of another determined cause; 5-stroke of an un
BMI_g*IMG_C_TOAST	1	4	1=<18.5;2=18.5-<23;3=23-<27.5;4=≥27.5 1 * K.Final diagnosis: cerebral infarction; Etiology according to TOAST system; 1-large artery atherosclerosis; 2-cardiogenic embolism; 3-small artery occlusion; 4-stroke of another determined cause; 5-stroke of an un
BMI_g*IMG_C_TOAST	1	3	1=<18.5;2=18.5-<23;3=23-<27.5;4=≥27.5 1 * K.Final diagnosis: cerebral infarction; Etiology according to TOAST system; 1-large artery atherosclerosis; 2-cardiogenic embolism; 3-small artery occlusion; 4-stroke of another determined cause; 5-stroke of an un
BMI_g*IMG_C_TOAST	1	2	1=<18.5;2=18.5-<23;3=23-<27.5;4=≥27.5 1 * K.Final diagnosis: cerebral infarction; Etiology according to TOAST system; 1-large artery atherosclerosis; 2-cardiogenic embolism; 3-small artery occlusion; 4-stroke of another determined cause; 5-stroke of an un
AGE			A.Basic Information: Age (years old);
GENDER	2		A.Basic Information: Gender; 1-male; 2-female; 2
ETHNIC	2		B.Demography: Race: 1-Han; 99-others; 2
H_DIAB01	1		D.History: Diabetes; 0-No; 1-Yes; 1
H_AF01	1		D.History: Heart disease category: Atrial fibrillation(Including medical history and hospitalization diagnosis); 0-No; 1-Yes; 1
H_HYPT01	1		D.History: Hypertension; 0-No; 1-Yes; 1

BMI_g with y1_death: interaction with stroke subtype

PHREG 过程

最大似然估计分析										
参数			自由度	参数<br>估计	标准<br>误差	卡方	Pr > 卡方	危险<br>率	95%<br>危险率置信限	
H_LIPID01	1		1	-0.30891	0.20809	2.2039	0.1377	0.734	0.488	1.104
AI	1		1	0.51917	0.22750	5.2080	0.0225	1.681	1.076	2.625
H_DRINK_H01	1		1	-0.52497	0.19580	7.1889	0.0073	0.592	0.403	0.868
H_SMK_C01	1		1	0.13125	0.12438	1.1136	0.2913	1.140	0.894	1.455
IT	1		1	-0.72554	0.16637	19.0182	<.0001	0.484	0.349	0.671
ET	1		1	0.90664	0.31403	8.3354	0.0039	2.476	1.338	4.582
A_NIHSS			1	0.09376	0.00632	220.1983	<.0001	1.098	1.085	1.112

BMI_g with y1_death: interaction with stroke subtype

PHREG 过程

最大似然估计分析			
参数			标签
H_LIPID01	1		D.History: Lipid metabolism disorders; 0-No; 1-Yes; 1
AI	1		history:Myocardial infarction; 0=NO; 1=YES; 1
H_DRINK_H01	1		D.History: Heavy Drinking(Alcohol consumption>=20g/day); 0-No,1-Yes; 1
H_SMK_C01	1		D.History: Current Smoking; 0-No,1-Yes; 1
IT	1		intravenous thrombolysis, 1=YES,0=NO 1
ET	1		动脉溶栓或机械取栓, 1=YES,0=NO 1
A_NIHSS			F.Admitting NIHSS: Total score;

BMI_g with y1_comb: Descriptive results

FREQ 过程

频数
行百分比

BMI_g-y1_comb表

BMI_g(1=<18.5;2=18.5-<23;3=23-<27.5;4= ≥ 27.5)	y1_comb(N12.Follow-up events at 12 months:Occurrence of combined vascular event(including cardiovascular death,non-fatal stroke,non-fatal myocardial infarction):0-No;1-Yes;) 0	1	合计
1	270<br>87.38	39<br>12.62	309
2	3448<br>89.44	407<br>10.56	3855
3	6707<br>89.53	784<br>10.47	7491
4	2216<br>88.96	275<br>11.04	2491
合计	12641	1505	14146

表“y1_comb-BMI_g”的统计量

统计量	自由度	值	概率
卡方	3	1.9609	0.5806
似然比卡方检验	3	1.8936	0.5948
Mantel-Haenszel 卡方	1	0.0011	0.9738
Phi 系数		0.0118	
列联系数		0.0118	
Cramer V		0.0118	

样本大小 = 14146

BMI_g with y1_comb: crude model

PHREG 过程

模型信息		
数据集	WORK.DATA2	
因变量	y1_comb_dd	N12.Follow-up events at 12 months: Days from onset to occurrence of combined vascular event;(day);
删失变量	y1_comb	N12.Follow-up events at 12 months:Occurrence of combined vascular event(including cardiovascular death,non-fatal stroke,non-fatal myocardial infarction):0-No;1-Yes;
删失值	0	
结值处理	BRESLOW	

读取的观测数<br>使用的观测数	14146<br>14146

分类水平信息				
分类	值	设计变量		
BMI_g	4	1	0	0
	3	0	1	0
	2	0	0	0
	1	0	0	1

事件和删失值个数汇总			
合计	事件	删失	删失百分比
14146	1505	12641	89.36

收敛状态
满足收敛准则 (GCONV=1E-8)。

模型拟合统计量		
准则	无协变量	带协变量
-2 LOG L	28553.433	28551.104
AIC	28553.433	28557.104
SBC	28553.433	28573.054

检验全局原假设: BETA=0			
检验	卡方	自由度	Pr > 卡方
似然比	2.3287	3	0.5070
评分	2.4548	3	0.4835
Wald	2.4522	3	0.4840

3 型检验			
效应	自由度	Wald 卡方	Pr > 卡方
BMI_g	3	2.4522	0.4840

BMI_g with y1_comb: crude model

PHREG 过程

最大似然估计分析

参数		自由度	参数估计	标准误差	卡方	Pr > 卡方	危险率	95% 危险率置信限		标签
BMI_g	4	1	0.04476	0.07806	0.3288	0.5664	1.046	0.897	1.219	1=<18.5;2=18.5-<23;3=23-<27.5;4= ≥ 27.5 4
BMI_g	3	1	-0.01269	0.06109	0.0432	0.8354	0.987	0.876	1.113	1=<18.5;2=18.5-<23;3=23-<27.5;4= ≥ 27.5 3
BMI_g	1	1	0.21796	0.16761	1.6912	0.1934	1.244	0.895	1.727	1=<18.5;2=18.5-<23;3=23-<27.5;4= ≥ 27.5 1

BMI_g with y1_comb: adjusted model

PHREG 过程

模型信息		
数据集	WORK.DATA2	
因变量	y1_comb_dd	N12.Follow-up events at 12 months: Days from onset to occurrence of combined vascular event;(day);
删失变量	y1_comb	N12.Follow-up events at 12 months:Occurrence of combined vascular event(including cardiovascular death,non-fatal stroke,non-fatal myocardial infarction):0-No;1-Yes;
删失值	0	
结值处理	BRESLOW	

读取的观测数	14146
使用的观测数	14146

分类水平信息						
分类	值	设计变量				
BMI_g	4	1	0	0		
	3	0	1	0		
	2	0	0	0		
	1	0	0	1		
GENDER	2	1				
	1	0				
ETHNIC	2	1				
	1	0				
H_DIAB01	1	1				
	0	0				
H_AF01	1	1				
	0	0				
H_HYPT01	1	1				
	0	0				
H_LIPID01	1	1				
	0	0				
AI	1	1				
	0	0				
H_DRINK_H01	1	1				
	0	0				
H_SMK_C01	1	1				
	0	0				
IT	1	1				
	0	0				
ET	1	1				
	0	0				
IMG_C_TOAST	5	1	0	0	0	
	4	0	1	0	0	
	3	0	0	1	0	

BMI_g with y1_comb: adjusted model

PHREG 过程

分类水平信息					
分类	值	设计变量			
	2	0	0	0	1
	1	0	0	0	0

事件和删失值个数汇总			
合计	事件	删失	删失 百分比
14146	1505	12641	89.36

收敛状态
满足收敛准则 (GCONV=1E-8)。

模型拟合统计量		
准则	无 协变量	带 协变量
-2 LOG L	28553.433	28381.389
AIC	28553.433	28421.389
SBC	28553.433	28527.720

检验全局原假设: BETA=0			
检验	卡方	自由度	Pr > 卡方
似然比	172.0437	20	<.0001
评分	183.3947	20	<.0001
Wald	179.2576	20	<.0001

3 型检验			
效应	自由度	Wald 卡方	Pr > 卡方
BMI_g	3	1.8358	0.6072
AGE	1	19.5352	<.0001
GENDER	1	0.1742	0.6764
ETHNIC	1	0.4359	0.5091
H_DIAB01	1	14.1781	0.0002
H_AF01	1	13.4979	0.0002
H_HYPT01	1	2.7088	0.0998
H_LIPID01	1	0.6494	0.4203
AI	1	2.1128	0.1461
H_DRINK_H01	1	1.2928	0.2555
H_SMK_C01	1	0.2992	0.5844
IT	1	0.2335	0.6290
ET	1	6.0336	0.0140
IMG_C_TOAST	4	54.3572	<.0001
A_NIHSS	1	15.9744	<.0001

BMI_g with y1_comb: adjusted model

PHREG 过程

最大似然估计分析									
参数		自由度	参数估计	标准误差	卡方	Pr > 卡方	危险率	95% 危险率置信限	
BMI_g	4	1	0.09246	0.07993	1.3382	0.2474	1.097	0.938	1.283
BMI_g	3	1	0.02146	0.06191	0.1202	0.7288	1.022	0.905	1.154
BMI_g	1	1	0.12814	0.16847	0.5785	0.4469	1.137	0.817	1.581
AGE		1	0.01105	0.00250	19.5352	<.0001	1.011	1.006	1.016
GENDER	2	1	0.02534	0.06072	0.1742	0.6764	1.026	0.911	1.155
ETHNIC	2	1	-0.10604	0.16061	0.4359	0.5091	0.899	0.657	1.232
H_DIAB01	1	1	0.22205	0.05897	14.1781	0.0002	1.249	1.112	1.402
H_AF01	1	1	0.42476	0.11561	13.4979	0.0002	1.529	1.219	1.918
H_HYPT01	1	1	0.09194	0.05586	2.7088	0.0998	1.096	0.983	1.223
H_LIPID01	1	1	-0.08035	0.09971	0.6494	0.4203	0.923	0.759	1.122
AI	1	1	0.22957	0.15794	2.1128	0.1461	1.258	0.923	1.715
H_DRINK_H01	1	1	0.09370	0.08241	1.2928	0.2555	1.098	0.934	1.291
H_SMK_C01	1	1	-0.03679	0.06725	0.2992	0.5844	0.964	0.845	1.100
IT	1	1	0.03952	0.08179	0.2335	0.6290	1.040	0.886	1.221
ET	1	1	0.61344	0.24974	6.0336	0.0140	1.847	1.132	3.013
IMG_C_TOAST	5	1	-0.32927	0.06149	28.6790	<.0001	0.719	0.638	0.812
IMG_C_TOAST	4	1	-0.02484	0.22328	0.0124	0.9114	0.975	0.630	1.511
IMG_C_TOAST	3	1	-0.52891	0.07915	44.6532	<.0001	0.589	0.505	0.688
IMG_C_TOAST	2	1	-0.42424	0.13554	9.7968	0.0017	0.654	0.502	0.853
A_NIHSS		1	0.02281	0.00571	15.9744	<.0001	1.023	1.012	1.035

BMI_g with y1_comb: adjusted model

PHREG 过程

最大似然估计分析		
参数		标签
BMI_g	4	1=<18.5;2=18.5-<23;3=23-<27.5;4=≥27.5 4
BMI_g	3	1=<18.5;2=18.5-<23;3=23-<27.5;4=≥27.5 3
BMI_g	1	1=<18.5;2=18.5-<23;3=23-<27.5;4=≥27.5 1
AGE		A.Basic Information: Age (years old);
GENDER	2	A.Basic Information: Gender; 1-male; 2-female; 2
ETHNIC	2	B.Demography: Race: 1-Han; 99-others; 2
H_DIAB01	1	D.History: Diabetes; 0-No; 1-Yes; 1
H_AF01	1	D.History: Heart disease category: Atrial fibrillation(Including medical history and hospitalization diagnosis); 0-No; 1-Yes; 1
H_HYPT01	1	D.History: Hypertension; 0-No; 1-Yes; 1
H_LIPID01	1	D.History: Lipid metabolism disorders; 0-No; 1-Yes; 1
AI	1	history:Myocardial infarction; 0=NO; 1=YES; 1
H_DRINK_H01	1	D.History: Heavy Drinking(Alcohol consumption>=20g/day); 0-No,1-Yes; 1
H_SMK_C01	1	D.History: Current Smoking; 0-No,1-Yes; 1
IT	1	intravenous thrombolysis, 1=YES,0=NO 1
ET	1	动脉溶栓或机械取栓, 1=YES,0=NO 1
IMG_C_TOAST	5	K.Final diagnosis: cerebral infarction; Etiology according to TOAST system; 1-large artery atherosclerosis; 2-cardiogenic embolism; 3-small artery occlusion; 4-stroke of another determined cause; 5-stroke of an undetermined cause. 5
IMG_C_TOAST	4	K.Final diagnosis: cerebral infarction; Etiology according to TOAST system; 1-large artery atherosclerosis; 2-cardiogenic embolism; 3-small artery occlusion; 4-stroke of another determined cause; 5-stroke of an undetermined cause. 4
IMG_C_TOAST	3	K.Final diagnosis: cerebral infarction; Etiology according to TOAST system; 1-large artery atherosclerosis; 2-cardiogenic embolism; 3-small artery occlusion; 4-stroke of another determined cause; 5-stroke of an undetermined cause. 3
IMG_C_TOAST	2	K.Final diagnosis: cerebral infarction; Etiology according to TOAST system; 1-large artery atherosclerosis; 2-cardiogenic embolism; 3-small artery occlusion; 4-stroke of another determined cause; 5-stroke of an undetermined cause. 2
A_NIHSS		F.Admitting NIHSS: Total score;

BMI_g with y1_comb: interaction with stroke subtype

PHREG 过程

模型信息		
数据集	WORK.DATA2	
因变量	y1_comb_dd	N12.Follow-up events at 12 months: Days from onset to occurrence of combined vascular event;(day);
删失变量	y1_comb	N12.Follow-up events at 12 months:Occurrence of combined vascular event(including cardiovascular death,non-fatal stroke,non-fatal myocardial infarction):0-No;1-Yes;
删失值	0	
结值处理	BRESLOW	

读取的观测数	14146
使用的观测数	14146

分类水平信息					
分类	值	设计变量			
BMI_g	4	1	0	0	
	3	0	1	0	
	2	0	0	0	
	1	0	0	1	
GENDER	2	1			
	1	0			
ETHNIC	2	1			
	1	0			
H_DIAB01	1	1			
	0	0			
H_AF01	1	1			
	0	0			
H_HYPT01	1	1			
	0	0			
H_LIPID01	1	1			
	0	0			
AI	1	1			
	0	0			
H_DRINK_H01	1	1			
	0	0			
H_SMK_C01	1	1			
	0	0			
IT	1	1			
	0	0			
ET	1	1			
	0	0			
IMG_C_TOAST	5	1	0	0	0
	4	0	1	0	0
	3	0	0	1	0

BMI_g with y1_comb: interaction with stroke subtype

PHREG 过程

分类水平信息					
分类	值	设计变量			
	2	0	0	0	1
	1	0	0	0	0

事件和删失值个数汇总			
合计	事件	删失	删失<br>百分比
14146	1505	12641	89.36

收敛状态
满足收敛准则 (GCONV=1E-8)。

模型拟合统计量		
准则	无<br>协变量	带<br>协变量
-2 LOG L	28553.433	28372.286
AIC	28553.433	28436.286
SBC	28553.433	28606.416

检验全局原假设: BETA=0			
检验	卡方	自由度	Pr > 卡方
似然比	181.1467	32	<.0001
评分	193.7800	32	<.0001
Wald	189.2000	32	<.0001

联合检验			
效应	自由度	Wald 卡方	Pr > 卡方
BMI_g	3	0.0775	0.9944
IMG_C_TOAST	4	17.9530	0.0013
BMI_g*IMG_C_TOAST	12	9.6493	0.6467
AGE	1	19.7388	<.0001
GENDER	1	0.2000	0.6547
ETHNIC	1	0.4260	0.5140
H_DIAB01	1	14.0691	0.0002
H_AF01	1	13.8671	0.0002
H_HYPT01	1	2.6650	0.1026
H_LIPID01	1	0.5738	0.4488
AI	1	1.9182	0.1661
H_DRINK_H01	1	1.2416	0.2652
H_SMK_C01	1	0.3127	0.5760
IT	1	0.2215	0.6379

BMI_g with y1_comb: interaction with stroke subtype

PHREG 过程

联合检验			
效应	自由度	Wald 卡方	Pr > 卡方
ET	1	6.3230	0.0119
A_NIHSS	1	15.9211	<.0001

Note: Under full-rank parameterizations, Type 3 effect tests are replaced by joint tests. The joint test for an effect is a test that all of the parameters associated with that effect are zero. Such joint tests might not be equivalent to Type 3 effect tests under GLM parameterization.

BMI_g with y1_comb: interaction with stroke subtype

PHREG 过程

最大似然估计分析										
参数			自由度	参数估计	标准误差	卡方	Pr > 卡方	危险率	95% 危险率置信限	
BMI_g	4		1	0.03041	0.13774	0.0487	0.8253	.	.	.
BMI_g	3		1	0.02608	0.10659	0.0598	0.8067	.	.	.
BMI_g	1		1	0.04184	0.31393	0.0178	0.8940	.	.	.
IMG_C_TOAST	5		1	-0.39335	0.11744	11.2182	0.0008	.	.	.
IMG_C_TOAST	4		1	-0.20892	0.45599	0.2099	0.6468	.	.	.
IMG_C_TOAST	3		1	-0.57374	0.15674	13.3999	0.0003	.	.	.
IMG_C_TOAST	2		1	-0.18278	0.19288	0.8980	0.3433	.	.	.
BMI_g*IMG_C_TOAST	4	5	1	0.17812	0.18216	0.9561	0.3282	.	.	.
BMI_g*IMG_C_TOAST	4	4	1	0.51446	0.64730	0.6317	0.4267	.	.	.
BMI_g*IMG_C_TOAST	4	3	1	0.04429	0.24163	0.0336	0.8546	.	.	.
BMI_g*IMG_C_TOAST	4	2	1	-0.28478	0.31622	0.8111	0.3678	.	.	.
BMI_g*IMG_C_TOAST	3	5	1	0.05747	0.14291	0.1617	0.6876	.	.	.
BMI_g*IMG_C_TOAST	3	4	1	0.10292	0.55804	0.0340	0.8537	.	.	.
BMI_g*IMG_C_TOAST	3	3	1	0.03543	0.18916	0.0351	0.8514	.	.	.
BMI_g*IMG_C_TOAST	3	2	1	-0.39778	0.23141	2.9549	0.0856	.	.	.
BMI_g*IMG_C_TOAST	1	5	1	0.03321	0.40902	0.0066	0.9353	.	.	.
BMI_g*IMG_C_TOAST	1	4	1	0.77299	1.14075	0.4592	0.4980	.	.	.
BMI_g*IMG_C_TOAST	1	3	1	0.70919	0.49077	2.0882	0.1484	.	.	.
BMI_g*IMG_C_TOAST	1	2	1	-0.54248	0.67434	0.6471	0.4211	.	.	.
AGE			1	0.01111	0.00250	19.7388	<.0001	1.011	1.006	1.016
GENDER	2		1	0.02716	0.06073	0.2000	0.6547	1.028	0.912	1.157
ETHNIC	2		1	-0.10494	0.16078	0.4260	0.5140	0.900	0.657	1.234
H_DIAB01	1		1	0.22139	0.05902	14.0691	0.0002	1.248	1.111	1.401
H_AF01	1		1	0.43047	0.11560	13.8671	0.0002	1.538	1.226	1.929
H_HYPT01	1		1	0.09127	0.05591	2.6650	0.1026	1.096	0.982	1.222

BMI_g with y1_comb: interaction with stroke subtype

PHREG 过程

最大似然估计分析			
参数			标签
BMI_g	4		1=<18.5;2=18.5-<23;3=23-<27.5;4= ≥ 27.5 4
BMI_g	3		1=<18.5;2=18.5-<23;3=23-<27.5;4= ≥ 27.5 3
BMI_g	1		1=<18.5;2=18.5-<23;3=23-<27.5;4= ≥ 27.5 1
IMG_C_TOAST	5		K.Final diagnosis: cerebral infarction; Etiology according to TOAST system; 1-large artery atherosclerosis; 2-cardiogenic embolism; 3-small artery occlusion; 4-stroke of another determined cause; 5-stroke of an undetermined cause. 5
IMG_C_TOAST	4		K.Final diagnosis: cerebral infarction; Etiology according to TOAST system; 1-large artery atherosclerosis; 2-cardiogenic embolism; 3-small artery occlusion; 4-stroke of another determined cause; 5-stroke of an undetermined cause. 4
IMG_C_TOAST	3		K.Final diagnosis: cerebral infarction; Etiology according to TOAST system; 1-large artery atherosclerosis; 2-cardiogenic embolism; 3-small artery occlusion; 4-stroke of another determined cause; 5-stroke of an undetermined cause. 3
IMG_C_TOAST	2		K.Final diagnosis: cerebral infarction; Etiology according to TOAST system; 1-large artery atherosclerosis; 2-cardiogenic embolism; 3-small artery occlusion; 4-stroke of another determined cause; 5-stroke of an undetermined cause. 2
BMI_g*IMG_C_TOAST	4	5	1=<18.5;2=18.5-<23;3=23-<27.5;4= ≥ 27.5 4 * K.Final diagnosis: cerebral infarction; Etiology according to TOAST system; 1-large artery atherosclerosis; 2-cardiogenic embolism; 3-small artery occlusion; 4-stroke of another determined cause; 5-stroke of an un
BMI_g*IMG_C_TOAST	4	4	1=<18.5;2=18.5-<23;3=23-<27.5;4= ≥ 27.5 4 * K.Final diagnosis: cerebral infarction; Etiology according to TOAST system; 1-large artery atherosclerosis; 2-cardiogenic embolism; 3-small artery occlusion; 4-stroke of another determined cause; 5-stroke of an un
BMI_g*IMG_C_TOAST	4	3	1=<18.5;2=18.5-<23;3=23-<27.5;4= ≥ 27.5 4 * K.Final diagnosis: cerebral infarction; Etiology according to TOAST system; 1-large artery atherosclerosis; 2-cardiogenic embolism; 3-small artery occlusion; 4-stroke of another determined cause; 5-stroke of an un
BMI_g*IMG_C_TOAST	4	2	1=<18.5;2=18.5-<23;3=23-<27.5;4= ≥ 27.5 4 * K.Final diagnosis: cerebral infarction; Etiology according to TOAST system; 1-large artery atherosclerosis; 2-cardiogenic embolism; 3-small artery occlusion; 4-stroke of another determined cause; 5-stroke of an un
BMI_g*IMG_C_TOAST	3	5	1=<18.5;2=18.5-<23;3=23-<27.5;4= ≥ 27.5 3 * K.Final diagnosis: cerebral infarction; Etiology according to TOAST system; 1-large artery atherosclerosis; 2-cardiogenic embolism; 3-small artery occlusion; 4-stroke of another determined cause; 5-stroke of an un
BMI_g*IMG_C_TOAST	3	4	1=<18.5;2=18.5-<23;3=23-<27.5;4= ≥ 27.5 3 * K.Final diagnosis: cerebral infarction; Etiology according to TOAST system; 1-large artery atherosclerosis; 2-cardiogenic embolism; 3-small artery occlusion; 4-stroke of another determined cause; 5-stroke of an un
BMI_g*IMG_C_TOAST	3	3	1=<18.5;2=18.5-<23;3=23-<27.5;4= ≥ 27.5 3 * K.Final diagnosis: cerebral infarction; Etiology according to TOAST system; 1-large artery atherosclerosis; 2-cardiogenic embolism; 3-small artery occlusion; 4-stroke of another determined cause; 5-stroke of an un
BMI_g*IMG_C_TOAST	3	2	1=<18.5;2=18.5-<23;3=23-<27.5;4= ≥ 27.5 3 * K.Final diagnosis: cerebral infarction; Etiology according to TOAST system; 1-large artery atherosclerosis; 2-cardiogenic embolism; 3-small artery occlusion; 4-stroke of another determined cause; 5-stroke of an un
BMI_g*IMG_C_TOAST	1	5	1=<18.5;2=18.5-<23;3=23-<27.5;4= ≥ 27.5 1 * K.Final diagnosis: cerebral infarction; Etiology according to TOAST system; 1-large artery atherosclerosis; 2-cardiogenic embolism; 3-small artery occlusion; 4-stroke of another determined cause; 5-stroke of an un
BMI_g*IMG_C_TOAST	1	4	1=<18.5;2=18.5-<23;3=23-<27.5;4= ≥ 27.5 1 * K.Final diagnosis: cerebral infarction; Etiology according to TOAST system; 1-large artery atherosclerosis; 2-cardiogenic embolism; 3-small artery occlusion; 4-stroke of another determined cause; 5-stroke of an un
BMI_g*IMG_C_TOAST	1	3	1=<18.5;2=18.5-<23;3=23-<27.5;4= ≥ 27.5 1 * K.Final diagnosis: cerebral infarction; Etiology according to TOAST system; 1-large artery atherosclerosis; 2-cardiogenic embolism; 3-small artery occlusion; 4-stroke of another determined cause; 5-stroke of an un
BMI_g*IMG_C_TOAST	1	2	1=<18.5;2=18.5-<23;3=23-<27.5;4= ≥ 27.5 1 * K.Final diagnosis: cerebral infarction; Etiology according to TOAST system; 1-large artery atherosclerosis; 2-cardiogenic embolism; 3-small artery occlusion; 4-stroke of another determined cause; 5-stroke of an un
AGE			A.Basic Information: Age (years old);
GENDER	2		A.Basic Information: Gender; 1-male; 2-female; 2
ETHNIC	2		B.Demography: Race: 1-Han; 99-others; 2
H_DIAB01	1		D.History: Diabetes; 0-No; 1-Yes; 1
H_AF01	1		D.History: Heart disease category: Atrial fibrillation(Including medical history and hospitalization diagnosis); 0-No; 1-Yes; 1
H_HYPT01	1		D.History: Hypertension; 0-No; 1-Yes; 1

BMI_g with y1_comb: interaction with stroke subtype

PHREG 过程

最大似然估计分析										
参数			自由度	参数估计	标准误差	卡方	Pr > 卡方	危险率	95% 危险率置信限	
H_LIPID01	1		1	-0.07557	0.09977	0.5738	0.4488	0.927	0.763	1.127
AI	1		1	0.21894	0.15808	1.9182	0.1661	1.245	0.913	1.697
H_DRINK_H01	1		1	0.09188	0.08245	1.2416	0.2652	1.096	0.933	1.289
H_SMK_C01	1		1	-0.03764	0.06731	0.3127	0.5760	0.963	0.844	1.099
IT	1		1	0.03852	0.08185	0.2215	0.6379	1.039	0.885	1.220
ET	1		1	0.62878	0.25005	6.3230	0.0119	1.875	1.149	3.061
A_NIHSS			1	0.02274	0.00570	15.9211	<.0001	1.023	1.012	1.034

BMI_g with y1_comb: interaction with stroke subtype

PHREG 过程

最大似然估计分析			
参数			标签
H_LIPID01	1		D.History: Lipid metabolism disorders; 0-No; 1-Yes; 1
AI	1		history:Myocardial infarction; 0=NO; 1=YES; 1
H_DRINK_H01	1		D.History: Heavy Drinking(Alcohol consumption>=20g/day); 0-No,1-Yes; 1
H_SMK_C01	1		D.History: Current Smoking; 0-No,1-Yes; 1
IT	1		intravenous thrombolysis, 1=YES,0=NO 1
ET	1		动脉溶栓或机械取栓, 1=YES,0=NO 1
A_NIHSS			F.Admitting NIHSS: Total score;

BMI_g with y1_stroke: Descriptive results

FREQ 过程

频数<br>行百分比

BMI_g-y1_stroke表

BMI_g(1=<18.5;2=18.5-<23;3=23-<27.5;4= ≥ 27.5)	y1_stroke(N12.Follow-up events at 12 months: Recurrence of stroke: 0-No; 1-Yes;)		
	0	1	合计
1	273<br>88.35	36<br>11.65	309
2	3473<br>90.09	382<br>9.91	3855
3	6750<br>90.11	741<br>9.89	7491
4	2226<br>89.36	265<br>10.64	2491
合计	12722	1424	14146

表“y1_stroke-BMI_g”的统计量

统计量	自由度	值	概率
卡方	3	2.1137	0.5491
似然比卡方检验	3	2.0638	0.5593
Mantel-Haenszel 卡方	1	0.1673	0.6826
Phi 系数		0.0122	
列联系数		0.0122	
Cramer V		0.0122	

样本大小 = 14146

BMI_g with y1_stroke: crude model

PHREG 过程

模型信息		
数据集	WORK.DATA2	
因变量	y1_stroke_dd	N12.Follow-up events at 12 months: Days from onset to recurrence;(day);
删失变量	y1_stroke	N12.Follow-up events at 12 months: Recurrence of stroke: 0-No; 1-Yes;
删失值	0	
结值处理	BRESLOW	

读取的观测数<br>使用的观测数	14146<br>14146

分类水平信息				
分类	值	设计变量		
BMI_g	4	1	0	0
	3	0	1	0
	2	0	0	0
	1	0	0	1

事件和删失值个数汇总			
合计	事件	删失	删失百分比
14146	1424	12722	89.93

收敛状态
满足收敛准则 (GCONV=1E-8)。

模型拟合统计量		
准则	无协变量	带协变量
-2 LOG L	27024.892	27022.516
AIC	27024.892	27028.516
SBC	27024.892	27044.299

检验全局原假设: BETA=0			
检验	卡方	自由度	Pr > 卡方
似然比	2.3764	3	0.4980
评分	2.4658	3	0.4815
Wald	2.4632	3	0.4820

3 型检验			
效应	自由度	Wald 卡方	Pr > 卡方
BMI_g	3	2.4632	0.4820

BMI_g with y1_stroke: crude model

PHREG 过程

最大似然估计分析

参数		自由度	参数估计	标准误差	卡方	Pr > 卡方	危险率	95% 危险率置信限		标签
BMI_g	4	1	0.07153	0.07995	0.8005	0.3709	1.074	0.918	1.256	1=<18.5;2=18.5-<23;3=23-<27.5;4=≥27.5 4
BMI_g	3	1	-0.00530	0.06299	0.0071	0.9329	0.995	0.879	1.125	1=<18.5;2=18.5-<23;3=23-<27.5;4=≥27.5 3
BMI_g	1	1	0.19950	0.17433	1.3095	0.2525	1.221	0.867	1.718	1=<18.5;2=18.5-<23;3=23-<27.5;4=≥27.5 1

BMI_g with y1_stroke: adjusted model

PHREG 过程

模型信息		
数据集	WORK.DATA2	
因变量	y1_stroke_dd	N12.Follow-up events at 12 months: Days from onset to recurrence;(day);
删失变量	y1_stroke	N12.Follow-up events at 12 months: Recurrence of stroke: 0-No; 1-Yes;
删失值	0	
结值处理	BRESLOW	

读取的观测数	14146
使用的观测数	14146

分类水平信息					
分类	值	设计变量			
BMI_g	4	1	0	0	
	3	0	1	0	
	2	0	0	0	
	1	0	0	1	
GENDER	2	1			
	1	0			
ETHNIC	2	1			
	1	0			
H_DIAB01	1	1			
	0	0			
H_AF01	1	1			
	0	0			
H_HYPT01	1	1			
	0	0			
H_LIPID01	1	1			
	0	0			
AI	1	1			
	0	0			
H_DRINK_H01	1	1			
	0	0			
H_SMK_C01	1	1			
	0	0			
IT	1	1			
	0	0			
ET	1	1			
	0	0			
IMG_C_TOAST	5	1	0	0	0
	4	0	1	0	0
	3	0	0	1	0

BMI_g with y1_stroke: adjusted model

PHREG 过程

分类水平信息					
分类	值	设计变量			
	2	0	0	0	1
	1	0	0	0	0

事件和删失值个数汇总			
合计	事件	删失	删失百分比
14146	1424	12722	89.93

收敛状态
满足收敛准则 (GCONV=1E-8)。

模型拟合统计量		
准则	无协变量	带协变量
-2 LOG L	27024.892	26877.840
AIC	27024.892	26917.840
SBC	27024.892	27023.065

检验全局原假设: BETA=0			
检验	卡方	自由度	Pr > 卡方
似然比	147.0516	20	<.0001
评分	155.6933	20	<.0001
Wald	152.4562	20	<.0001

3 型检验			
效应	自由度	Wald 卡方	Pr > 卡方
BMI_g	3	2.4618	0.4822
AGE	1	14.9730	0.0001
GENDER	1	0.4799	0.4885
ETHNIC	1	0.6019	0.4378
H_DIAB01	1	11.9443	0.0005
H_AF01	1	8.5647	0.0034
H_HYPT01	1	1.3328	0.2483
H_LIPID01	1	0.6025	0.4376
AI	1	1.0005	0.3172
H_DRINK_H01	1	2.9967	0.0834
H_SMK_C01	1	0.7502	0.3864
IT	1	1.0693	0.3011
ET	1	5.9033	0.0151
IMG_C_TOAST	4	52.7955	<.0001
A_NIHSS	1	12.6445	0.0004

BMI_g with y1_stroke: adjusted model

PHREG 过程

最大似然估计分析									
参数		自由度	参数估计	标准误差	卡方	Pr > 卡方	危险率	95% 危险率置信限	
BMI_g	4	1	0.11789	0.08187	2.0737	0.1499	1.125	0.958	1.321
BMI_g	3	1	0.02910	0.06382	0.2080	0.6484	1.030	0.908	1.167
BMI_g	1	1	0.11803	0.17518	0.4539	0.5005	1.125	0.798	1.586
AGE		1	0.00991	0.00256	14.9730	0.0001	1.010	1.005	1.015
GENDER	2	1	0.04327	0.06246	0.4799	0.4885	1.044	0.924	1.180
ETHNIC	2	1	-0.12951	0.16693	0.6019	0.4378	0.879	0.633	1.219
H_DIAB01	1	1	0.21005	0.06078	11.9443	0.0005	1.234	1.095	1.390
H_AF01	1	1	0.35695	0.12197	8.5647	0.0034	1.429	1.125	1.815
H_HYPT01	1	1	0.06606	0.05722	1.3328	0.2483	1.068	0.955	1.195
H_LIPID01	1	1	-0.07957	0.10251	0.6025	0.4376	0.924	0.755	1.129
AI	1	1	0.16806	0.16801	1.0005	0.3172	1.183	0.851	1.644
H_DRINK_H01	1	1	0.14496	0.08374	2.9967	0.0834	1.156	0.981	1.362
H_SMK_C01	1	1	-0.05997	0.06923	0.7502	0.3864	0.942	0.822	1.079
IT	1	1	0.08591	0.08308	1.0693	0.3011	1.090	0.926	1.282
ET	1	1	0.62552	0.25745	5.9033	0.0151	1.869	1.129	3.096
IMG_C_TOAST	5	1	-0.33702	0.06298	28.6316	<.0001	0.714	0.631	0.808
IMG_C_TOAST	4	1	-0.03564	0.22879	0.0243	0.8762	0.965	0.616	1.511
IMG_C_TOAST	3	1	-0.52829	0.08089	42.6528	<.0001	0.590	0.503	0.691
IMG_C_TOAST	2	1	-0.44681	0.14210	9.8864	0.0017	0.640	0.484	0.845
A_NIHSS		1	0.02110	0.00593	12.6445	0.0004	1.021	1.010	1.033

BMI_g with y1_stroke: adjusted model

PHREG 过程

最大似然估计分析		
参数		标签
BMI_g	4	1=<18.5;2=18.5-<23;3=23-<27.5;4= ≥ 27.5 4
BMI_g	3	1=<18.5;2=18.5-<23;3=23-<27.5;4= ≥ 27.5 3
BMI_g	1	1=<18.5;2=18.5-<23;3=23-<27.5;4= ≥ 27.5 1
AGE		A.Basic Information: Age (years old);
GENDER	2	A.Basic Information: Gender; 1-male; 2-female; 2
ETHNIC	2	B.Demography: Race: 1-Han; 99-others; 2
H_DIAB01	1	D.History: Diabetes; 0-No; 1-Yes; 1
H_AF01	1	D.History: Heart disease category: Atrial fibrillation(Including medical history and hospitalization diagnosis); 0-No; 1-Yes; 1
H_HYPT01	1	D.History: Hypertension; 0-No; 1-Yes; 1
H_LIPID01	1	D.History: Lipid metabolism disorders; 0-No; 1-Yes; 1
AI	1	history:Myocardial infarction; 0=NO; 1=YES; 1
H_DRINK_H01	1	D.History: Heavy Drinking(Alcohol consumption>=20g/day); 0-No,1-Yes; 1
H_SMK_C01	1	D.History: Current Smoking; 0-No,1-Yes; 1
IT	1	intravenous thrombolysis, 1=YES,0=NO 1
ET	1	动脉溶栓或机械取栓, 1=YES,0=NO 1
IMG_C_TOAST	5	K.Final diagnosis: cerebral infarction; Etiology according to TOAST system; 1-large artery atherosclerosis; 2-cardiogenic embolism; 3-small artery occlusion; 4-stroke of another determined cause; 5-stroke of an undetermined cause. 5
IMG_C_TOAST	4	K.Final diagnosis: cerebral infarction; Etiology according to TOAST system; 1-large artery atherosclerosis; 2-cardiogenic embolism; 3-small artery occlusion; 4-stroke of another determined cause; 5-stroke of an undetermined cause. 4
IMG_C_TOAST	3	K.Final diagnosis: cerebral infarction; Etiology according to TOAST system; 1-large artery atherosclerosis; 2-cardiogenic embolism; 3-small artery occlusion; 4-stroke of another determined cause; 5-stroke of an undetermined cause. 3
IMG_C_TOAST	2	K.Final diagnosis: cerebral infarction; Etiology according to TOAST system; 1-large artery atherosclerosis; 2-cardiogenic embolism; 3-small artery occlusion; 4-stroke of another determined cause; 5-stroke of an undetermined cause. 2
A_NIHSS		F.Admitting NIHSS: Total score;

BMI_g with y1_stroke: interaction with stroke subtype

PHREG 过程

模型信息		
数据集	WORK.DATA2	
因变量	y1_stroke_dd	N12.Follow-up events at 12 months: Days from onset to recurrence;(day);
删失变量	y1_stroke	N12.Follow-up events at 12 months: Recurrence of stroke: 0-No; 1-Yes;
删失值	0	
结值处理	BRESLOW	

读取的观测数	14146
使用的观测数	14146

分类水平信息						
分类	值	设计变量				
BMI_g	4	1	0	0	
	3	0	1	0	
	2	0	0	0	
	1	0	0	1	
GENDER	2	1			
	1	0			
ETHNIC	2	1			
	1	0			
H_DIAB01	1	1			
	0	0			
H_AF01	1	1			
	0	0			
H_HYPT01	1	1			
	0	0			
H_LIPID01	1	1			
	0	0			
AI	1	1			
	0	0			
H_DRINK_H01	1	1			
	0	0			
H_SMK_C01	1	1			
	0	0			
IT	1	1			
	0	0			
ET	1	1			
	0	0			
IMG_C_TOAST	5	1	0	0	0
	4	0	1	0	0
	3	0	0	1	0

BMI_g with y1_stroke: interaction with stroke subtype

PHREG 过程

分类水平信息					
分类	值	设计变量			
	2	0	0	0	1
	1	0	0	0	0

事件和删失值个数汇总			
合计	事件	删失	删失百分比
14146	1424	12722	89.93

收敛状态
满足收敛准则 (GCONV=1E-8)。

模型拟合统计量		
准则	无协变量	带协变量
-2 LOG L	27024.892	26868.363
AIC	27024.892	26932.363
SBC	27024.892	27100.722

检验全局原假设: BETA=0			
检验	卡方	自由度	Pr > 卡方
似然比	156.5288	32	<.0001
评分	165.9605	32	<.0001
Wald	162.2628	32	<.0001

联合检验			
效应	自由度	Wald 卡方	Pr > 卡方
BMI_g	3	0.0973	0.9922
IMG_C_TOAST	4	18.1482	0.0012
BMI_g*IMG_C_TOAST	12	9.8824	0.6263
AGE	1	15.1308	0.0001
GENDER	1	0.5294	0.4669
ETHNIC	1	0.6016	0.4380
H_DIAB01	1	11.7900	0.0006
H_AF01	1	8.8763	0.0029
H_HYPT01	1	1.3156	0.2514
H_LIPID01	1	0.5310	0.4662
AI	1	0.8884	0.3459
H_DRINK_H01	1	2.9374	0.0866
H_SMK_C01	1	0.7637	0.3822
IT	1	1.0512	0.3052

BMI_g with y1_stroke: interaction with stroke subtype

PHREG 过程

联合检验			
效应	自由度	Wald 卡方	Pr > 卡方
ET	1	6.1912	0.0128
A_NIHSS	1	12.5726	0.0004

Note: Under full-rank parameterizations, Type 3 effect tests are replaced by joint tests. The joint test for an effect is a test that all of the parameters associated with that effect are zero. Such joint tests might not be equivalent to Type 3 effect tests under GLM parameterization.

BMI_g with y1_stroke: interaction with stroke subtype

PHREG 过程

最大似然估计分析										
参数			自由度	参数估计	标准误差	卡方	Pr > 卡方	危险率	95% 危险率置信限	
BMI_g	4		1	0.03928	0.14045	0.0782	0.7797	.	.	.
BMI_g	3		1	0.02630	0.10908	0.0581	0.8095	.	.	.
BMI_g	1		1	-0.00510	0.32863	0.0002	0.9876	.	.	.
IMG_C_TOAST	5		1	-0.38797	0.12006	10.4416	0.0012	.	.	.
IMG_C_TOAST	4		1	-0.39961	0.50824	0.6182	0.4317	.	.	.
IMG_C_TOAST	3		1	-0.62059	0.16277	14.5359	0.0001	.	.	.
IMG_C_TOAST	2		1	-0.23277	0.20382	1.3042	0.2534	.	.	.
BMI_g*IMG_C_TOAST	4	5	1	0.17875	0.18571	0.9265	0.3358	.	.	.
BMI_g*IMG_C_TOAST	4	4	1	0.73695	0.68539	1.1561	0.2823	.	.	.
BMI_g*IMG_C_TOAST	4	3	1	0.10467	0.24743	0.1789	0.6723	.	.	.
BMI_g*IMG_C_TOAST	4	2	1	-0.26393	0.33470	0.6218	0.4304	.	.	.
BMI_g*IMG_C_TOAST	3	5	1	0.02980	0.14648	0.0414	0.8388	.	.	.
BMI_g*IMG_C_TOAST	3	4	1	0.33418	0.60162	0.3085	0.5786	.	.	.
BMI_g*IMG_C_TOAST	3	3	1	0.09517	0.19523	0.2376	0.6259	.	.	.
BMI_g*IMG_C_TOAST	3	2	1	-0.33485	0.24341	1.8924	0.1689	.	.	.
BMI_g*IMG_C_TOAST	1	5	1	0.06694	0.42567	0.0247	0.8750	.	.	.
BMI_g*IMG_C_TOAST	1	4	1	1.04584	1.16648	0.8039	0.3699	.	.	.
BMI_g*IMG_C_TOAST	1	3	1	0.84251	0.50190	2.8179	0.0932	.	.	.
BMI_g*IMG_C_TOAST	1	2	1	-0.75697	0.79647	0.9033	0.3419	.	.	.
AGE			1	0.00997	0.00256	15.1308	0.0001	1.010	1.005	1.015
GENDER	2		1	0.04546	0.06248	0.5294	0.4669	1.047	0.926	1.183
ETHNIC	2		1	-0.12961	0.16710	0.6016	0.4380	0.878	0.633	1.219
H_DIAB01	1		1	0.20884	0.06082	11.7900	0.0006	1.232	1.094	1.388
H_AF01	1		1	0.36307	0.12186	8.8763	0.0029	1.438	1.132	1.826
H_HYPT01	1		1	0.06568	0.05726	1.3156	0.2514	1.068	0.955	1.195

BMI_g with y1_stroke: interaction with stroke subtype

PHREG 过程

最大似然估计分析			
参数			标签
BMI_g	4		1=<18.5;2=18.5-<23;3=23-<27.5;4= ≥ 27.5 4
BMI_g	3		1=<18.5;2=18.5-<23;3=23-<27.5;4= ≥ 27.5 3
BMI_g	1		1=<18.5;2=18.5-<23;3=23-<27.5;4= ≥ 27.5 1
IMG_C_TOAST	5		K.Final diagnosis: cerebral infarction; Etiology according to TOAST system; 1-large artery atherosclerosis; 2-cardiogenic embolism; 3-small artery occlusion; 4-stroke of another determined cause; 5-stroke of an undetermined cause. 5
IMG_C_TOAST	4		K.Final diagnosis: cerebral infarction; Etiology according to TOAST system; 1-large artery atherosclerosis; 2-cardiogenic embolism; 3-small artery occlusion; 4-stroke of another determined cause; 5-stroke of an undetermined cause. 4
IMG_C_TOAST	3		K.Final diagnosis: cerebral infarction; Etiology according to TOAST system; 1-large artery atherosclerosis; 2-cardiogenic embolism; 3-small artery occlusion; 4-stroke of another determined cause; 5-stroke of an undetermined cause. 3
IMG_C_TOAST	2		K.Final diagnosis: cerebral infarction; Etiology according to TOAST system; 1-large artery atherosclerosis; 2-cardiogenic embolism; 3-small artery occlusion; 4-stroke of another determined cause; 5-stroke of an undetermined cause. 2
BMI_g*IMG_C_TOAST	4	5	1=<18.5;2=18.5-<23;3=23-<27.5;4= ≥ 27.5 4 * K.Final diagnosis: cerebral infarction; Etiology according to TOAST system; 1-large artery atherosclerosis; 2-cardiogenic embolism; 3-small artery occlusion; 4-stroke of another determined cause; 5-stroke of an un
BMI_g*IMG_C_TOAST	4	4	1=<18.5;2=18.5-<23;3=23-<27.5;4= ≥ 27.5 4 * K.Final diagnosis: cerebral infarction; Etiology according to TOAST system; 1-large artery atherosclerosis; 2-cardiogenic embolism; 3-small artery occlusion; 4-stroke of another determined cause; 5-stroke of an un
BMI_g*IMG_C_TOAST	4	3	1=<18.5;2=18.5-<23;3=23-<27.5;4= ≥ 27.5 4 * K.Final diagnosis: cerebral infarction; Etiology according to TOAST system; 1-large artery atherosclerosis; 2-cardiogenic embolism; 3-small artery occlusion; 4-stroke of another determined cause; 5-stroke of an un
BMI_g*IMG_C_TOAST	4	2	1=<18.5;2=18.5-<23;3=23-<27.5;4= ≥ 27.5 4 * K.Final diagnosis: cerebral infarction; Etiology according to TOAST system; 1-large artery atherosclerosis; 2-cardiogenic embolism; 3-small artery occlusion; 4-stroke of another determined cause; 5-stroke of an un
BMI_g*IMG_C_TOAST	3	5	1=<18.5;2=18.5-<23;3=23-<27.5;4= ≥ 27.5 3 * K.Final diagnosis: cerebral infarction; Etiology according to TOAST system; 1-large artery atherosclerosis; 2-cardiogenic embolism; 3-small artery occlusion; 4-stroke of another determined cause; 5-stroke of an un
BMI_g*IMG_C_TOAST	3	4	1=<18.5;2=18.5-<23;3=23-<27.5;4= ≥ 27.5 3 * K.Final diagnosis: cerebral infarction; Etiology according to TOAST system; 1-large artery atherosclerosis; 2-cardiogenic embolism; 3-small artery occlusion; 4-stroke of another determined cause; 5-stroke of an un
BMI_g*IMG_C_TOAST	3	3	1=<18.5;2=18.5-<23;3=23-<27.5;4= ≥ 27.5 3 * K.Final diagnosis: cerebral infarction; Etiology according to TOAST system; 1-large artery atherosclerosis; 2-cardiogenic embolism; 3-small artery occlusion; 4-stroke of another determined cause; 5-stroke of an un
BMI_g*IMG_C_TOAST	3	2	1=<18.5;2=18.5-<23;3=23-<27.5;4= ≥ 27.5 3 * K.Final diagnosis: cerebral infarction; Etiology according to TOAST system; 1-large artery atherosclerosis; 2-cardiogenic embolism; 3-small artery occlusion; 4-stroke of another determined cause; 5-stroke of an un
BMI_g*IMG_C_TOAST	1	5	1=<18.5;2=18.5-<23;3=23-<27.5;4= ≥ 27.5 1 * K.Final diagnosis: cerebral infarction; Etiology according to TOAST system; 1-large artery atherosclerosis; 2-cardiogenic embolism; 3-small artery occlusion; 4-stroke of another determined cause; 5-stroke of an un
BMI_g*IMG_C_TOAST	1	4	1=<18.5;2=18.5-<23;3=23-<27.5;4= ≥ 27.5 1 * K.Final diagnosis: cerebral infarction; Etiology according to TOAST system; 1-large artery atherosclerosis; 2-cardiogenic embolism; 3-small artery occlusion; 4-stroke of another determined cause; 5-stroke of an un
BMI_g*IMG_C_TOAST	1	3	1=<18.5;2=18.5-<23;3=23-<27.5;4= ≥ 27.5 1 * K.Final diagnosis: cerebral infarction; Etiology according to TOAST system; 1-large artery atherosclerosis; 2-cardiogenic embolism; 3-small artery occlusion; 4-stroke of another determined cause; 5-stroke of an un
BMI_g*IMG_C_TOAST	1	2	1=<18.5;2=18.5-<23;3=23-<27.5;4= ≥ 27.5 1 * K.Final diagnosis: cerebral infarction; Etiology according to TOAST system; 1-large artery atherosclerosis; 2-cardiogenic embolism; 3-small artery occlusion; 4-stroke of another determined cause; 5-stroke of an un
AGE			A.Basic Information: Age (years old);
GENDER	2		A.Basic Information: Gender; 1-male; 2-female; 2
ETHNIC	2		B.Demography: Race: 1-Han; 99-others; 2
H_DIAB01	1		D.History: Diabetes; 0-No; 1-Yes; 1
H_AF01	1		D.History: Heart disease category: Atrial fibrillation(Including medical history and hospitalization diagnosis); 0-No; 1-Yes; 1
H_HYPT01	1		D.History: Hypertension; 0-No; 1-Yes; 1

BMI_g with y1_stroke: interaction with stroke subtype

PHREG 过程

最大似然估计分析										
参数			自由度	参数估计	标准误差	卡方	Pr > 卡方	危险率	95% 危险率置信限	
H_LIPID01	1		1	-0.07474	0.10256	0.5310	0.4662	0.928	0.759	1.135
AI	1		1	0.15848	0.16814	0.8884	0.3459	1.172	0.843	1.629
H_DRINK_H01	1		1	0.14360	0.08378	2.9374	0.0866	1.154	0.980	1.360
H_SMK_C01	1		1	-0.06056	0.06930	0.7637	0.3822	0.941	0.822	1.078
IT	1		1	0.08523	0.08313	1.0512	0.3052	1.089	0.925	1.282
ET	1		1	0.64137	0.25776	6.1912	0.0128	1.899	1.146	3.147
A_NIHSS			1	0.02100	0.00592	12.5726	0.0004	1.021	1.009	1.033

BMI_g with y1_stroke: interaction with stroke subtype

PHREG 过程

最大似然估计分析			
参数			标签
H_LIPID01	1		D.History: Lipid metabolism disorders; 0-No; 1-Yes; 1
AI	1		history:Myocardial infarction; 0=NO; 1=YES; 1
H_DRINK_H01	1		D.History: Heavy Drinking(Alcohol consumption>=20g/day); 0-No,1-Yes; 1
H_SMK_C01	1		D.History: Current Smoking; 0-No,1-Yes; 1
IT	1		intravenous thrombolysis, 1=YES,0=NO 1
ET	1		动脉溶栓或机械取栓, 1=YES,0=NO 1
A_NIHSS			F.Admitting NIHSS: Total score;
