# Supplementary material for: Association of body mass index and waist-to-height ratio with outcomes in ischemic stroke: results from the Third China National Stroke Registry
Source: BMC Neurol. 2023 Apr 14;23:152. doi: 10.1186/s12883-023-03165-y (PMC10103413; doi:10.1186/s12883-023-03165-y)
Supplement: Supplementary file 2 — Additional file 2. [file 12883_2023_3165_MOESM2_ESM.zip › raw data/Table2.pdf]

FREQ 过程

1=Q1(<0.47);2=Q2(0.47-<0.52);3=Q3(0.52-<0.57);4=Q4(>=0.57)				
whtr_g	频数	百分比	累积<br>频数	累积<br>百分比
1	1169	24.33	1169	24.33
2	1316	27.39	2485	51.72
3	1190	24.77	3675	76.48
4	1130	23.52	4805	100.00

MEANS PROCEDURE

变量	标签	数目	缺失值个数	均值	标准差	最小值	下四分位数	中位数	上四分位数	最大值
BMI	F.Physical examination: Body mass index (kg/m2);	4805	0	24.4833409	3.4273485	13.8410000	22.3130000	24.2210000	26.3700000	51.0730000
AGE	A.Basic Information: Age (years old);	4805	0	63.4472425	11.3680560	23.0000000	56.0000000	64.0000000	72.0000000	93.0000000
A_NIHSS	F.Admitting NIHSS: Total score;	4805	0	4.4278876	4.1805192	0	2.0000000	3.0000000	6.0000000	40.0000000

FREQ 过程

A.Basic Information: Gender; 1-male; 2-female;				
GENDER	频数	百分比	累积频数	累积百分比
1	3214	66.89	3214	66.89
2	1591	33.11	4805	100.00

B.Demography: Race: 1-Han; 99-others;				
ETHNIC	频数	百分比	累积频数	累积百分比
1	4583	95.38	4583	95.38
2	222	4.62	4805	100.00

D.History: Stroke History; 0-No; 1-Yes;				
H_STROKE01	频数	百分比	累积频数	累积百分比
0	3873	80.60	3873	80.60
1	932	19.40	4805	100.00

D.History: Diabetes; 0-No; 1-Yes;				
H_DIAB01	频数	百分比	累积频数	累积百分比
0	3698	76.96	3698	76.96
1	1107	23.04	4805	100.00

D.History: Heart disease category: Atrial fibrillation(Including medical history and hospitalization diagnosis); 0-No; 1-Yes;				
H_AF01	频数	百分比	累积频数	累积百分比
0	4421	92.01	4421	92.01
1	384	7.99	4805	100.00

history:Myocardial infarction; 0=NO; 1=YES;				
AI	频数	百分比	累积频数	累积百分比
0	4732	98.48	4732	98.48
1	73	1.52	4805	100.00

D.History: Hypertension; 0-No; 1-Yes;				
H_HYPT01	频数	百分比	累积频数	累积百分比
0	1698	35.34	1698	35.34
1	3107	64.66	4805	100.00

FREQ 过程

D.History: Lipid metabolism disorders; 0-No; 1-Yes;				
H_LIPID01	频数	百分比	累积频数	累积百分比
0	4475	93.13	4475	93.13
1	330	6.87	4805	100.00

D.History: Heavy Drinking(Alcohol consumption>=20g/day); 0-No,1-Yes;				
H_DRINK_H01	频数	百分比	累积频数	累积百分比
0	4213	87.68	4213	87.68
1	592	12.32	4805	100.00

D.History: Current Smoking; 0-No,1-Yes;				
H_SMK_C01	频数	百分比	累积频数	累积百分比
0	3416	71.09	3416	71.09
1	1389	28.91	4805	100.00

intravenous thrombolysis, 1=YES,0=NO				
IT	频数	百分比	累积频数	累积百分比
0	4269	88.84	4269	88.84
1	536	11.16	4805	100.00

动脉溶栓或机械取栓, 1=YES,0=NO				
ET	频数	百分比	累积频数	累积百分比
0	4777	99.42	4777	99.42
1	28	0.58	4805	100.00

K.Final diagnosis: cerebral infarction; Etiology according to TOAST system; 1-large artery atherosclerosis; 2-cardiogenic embolism; 3-small artery occlusion; 4-stroke of another determined cause; 5-stroke of an undetermined cause.				
IMG_C_TOAST	频数	百分比	累积频数	累积百分比
1	1224	25.47	1224	25.47
2	341	7.10	1565	32.57
3	1048	21.81	2613	54.38
4	77	1.60	2690	55.98
5	2115	44.02	4805	100.00

FREQ 过程

N12.Follow-up events at 12 months: Recurrence of stroke: 0-No; 1-Yes;				
y1_stroke	频数	百分比	累积频数	累积百分比
0	4316	89.82	4316	89.82
1	489	10.18	4805	100.00

N12.Follow-up events at 12 months: recurrence of ischemic stroke: 0-No; 1-Yes;				
y1_is	频数	百分比	累积频数	累积百分比
0	4360	90.74	4360	90.74
1	445	9.26	4805	100.00

N12.Follow-up events at 12 months: recurrence of hemorrhage stroke: 0-No; 1-Yes;				
y1_HS	频数	百分比	累积频数	累积百分比
0	4755	98.96	4755	98.96
1	50	1.04	4805	100.00

I.Inpatient Event: Hemorrhagic transformation after cerebral infarction; 1-No; 2-Yes; 98-UK;				
I_IS_HT	频数	百分比	累积频数	累积百分比
.	64	1.33	64	1.33
1	4659	96.96	4723	98.29
2	82	1.71	4805	100.00

1年心血管源性死亡，0=NO; 1=YES				
death_cvd	频数	百分比	累积频数	累积百分比
0	4740	98.65	4740	98.65
1	65	1.35	4805	100.00

N12.Follow-up events at 12 months:Occurrence of combined vascular event(including cardiovascular death,non-fatal stroke,non-fatal myocardial infarction):0-No;1-Yes;				
y1_comb	频数	百分比	累积频数	累积百分比
0	4285	89.18	4285	89.18
1	520	10.82	4805	100.00

FREQ 过程

N12.Follow-up events at 12 months:
Whether the patient died:
0-survival;1-death;

y1_death	频数	百分比	累积频数	累积百分比
0	4615	96.05	4615	96.05
1	190	3.95	4805	100.00

continuous variables, descriptive by group

MEANS PROCEDURE

1=Q1(<0.47);2=Q2(0.47-<0.52);3=Q3(0.52-<0.57);4=Q4 (>=0.57)	观测数	变量	标签	数目	缺失值个数	均值	标准差
1	1169	BMI<br>AGE<br>A_NIHSS	F.Physical examination: Body mass index (kg/m2);<br>A.Basic Information: Age (years old);<br>F.Admitting NIHSS: Total score;	1169<br>1169<br>1169	0<br>0<br>0	22.8716193<br>64.8374679<br>4.5252352	3.1886621<br>11.9566540<br>4.7884623
2	1316	BMI<br>AGE<br>A_NIHSS	F.Physical examination: Body mass index (kg/m2);<br>A.Basic Information: Age (years old);<br>F.Admitting NIHSS: Total score;	1316<br>1316<br>1316	0<br>0<br>0	23.5727842<br>63.6018237<br>4.4103343	2.8344671<br>11.1381015<br>4.1306072
3	1190	BMI<br>AGE<br>A_NIHSS	F.Physical examination: Body mass index (kg/m2);<br>A.Basic Information: Age (years old);<br>F.Admitting NIHSS: Total score;	1190<br>1190<br>1190	0<br>0<br>0	24.8104941<br>62.6000000<br>4.5176471	2.9212129<br>11.0995041<br>4.0415347
4	1130	BMI<br>AGE<br>A_NIHSS	F.Physical examination: Body mass index (kg/m2);<br>A.Basic Information: Age (years old);<br>F.Admitting NIHSS: Total score;	1130<br>1130<br>1130	0<br>0<br>0	26.8666000<br>62.7212389<br>4.2530973	3.4155359<br>11.1553643<br>3.6768992

continuous variables, descriptive by group

MEANS PROCEDURE

1=Q1(<0.47);2=Q2(0.47-<0.52);3=Q3(0.52-<0.57);4=Q4 (>=0.57)	观测数	变量	最小值	下四分位数	中位数	上四分位数
1	1169	BMI	13.8410000	20.7610000	22.8370000	24.8020000
		AGE	23.0000000	57.0000000	65.0000000	74.0000000
		A_NIHSS	0	2.0000000	3.0000000	6.0000000
2	1316	BMI	15.2110000	21.7180000	23.4380000	25.1225000
		AGE	27.0000000	56.0000000	64.0000000	72.0000000
		A_NIHSS	0	2.0000000	3.0000000	6.0000000
3	1190	BMI	13.8410000	23.0520000	24.6830000	26.3610000
		AGE	26.0000000	55.0000000	63.0000000	70.0000000
		A_NIHSS	0	2.0000000	3.0000000	6.0000000
4	1130	BMI	16.5290000	24.4900000	26.7090000	29.0530000
		AGE	28.0000000	55.0000000	63.0000000	71.0000000
		A_NIHSS	0	2.0000000	3.0000000	6.0000000

continuous variables, descriptive by group

MEANS PROCEDURE

1=Q1(<0.47);2=Q2(0.47-<0.52);3=Q3(0.52-<0.57);4=Q4 (>=0.57)	观测数	变量	最大值
1	1169	BMI<br>AGE<br>A_NIHSS	45.7250000<br>93.0000000<br>40.0000000
2	1316	BMI<br>AGE<br>A_NIHSS	48.0690000<br>93.0000000<br>36.0000000
3	1190	BMI<br>AGE<br>A_NIHSS	48.6840000<br>89.0000000<br>31.0000000
4	1130	BMI<br>AGE<br>A_NIHSS	51.0730000<br>90.0000000<br>26.0000000

Kruskal-Wallis Test among differenct group

NPAR1WAY 过程

变量“BMI”的 Wilcoxon 评分（秩和）按变量“whtr_g”分类					
whtr_g	数目	评分汇总	H0 之下的期望值	H0 之下的标准差	均值评分
4	1130	3816700.50	2715390.0	40780.9175	3377.61106
2	1316	2629978.00	3162348.0	42881.2591	1998.46353
1	1169	2023943.50	2809107.0	41258.0115	1731.34602
3	1190	3075793.00	2859570.0	41506.5590	2584.70000
已将平均评分用于结值。					

Kruskal-Wallis 检验		
卡方	自由度	Pr > 卡方
964.1758	3	<.0001

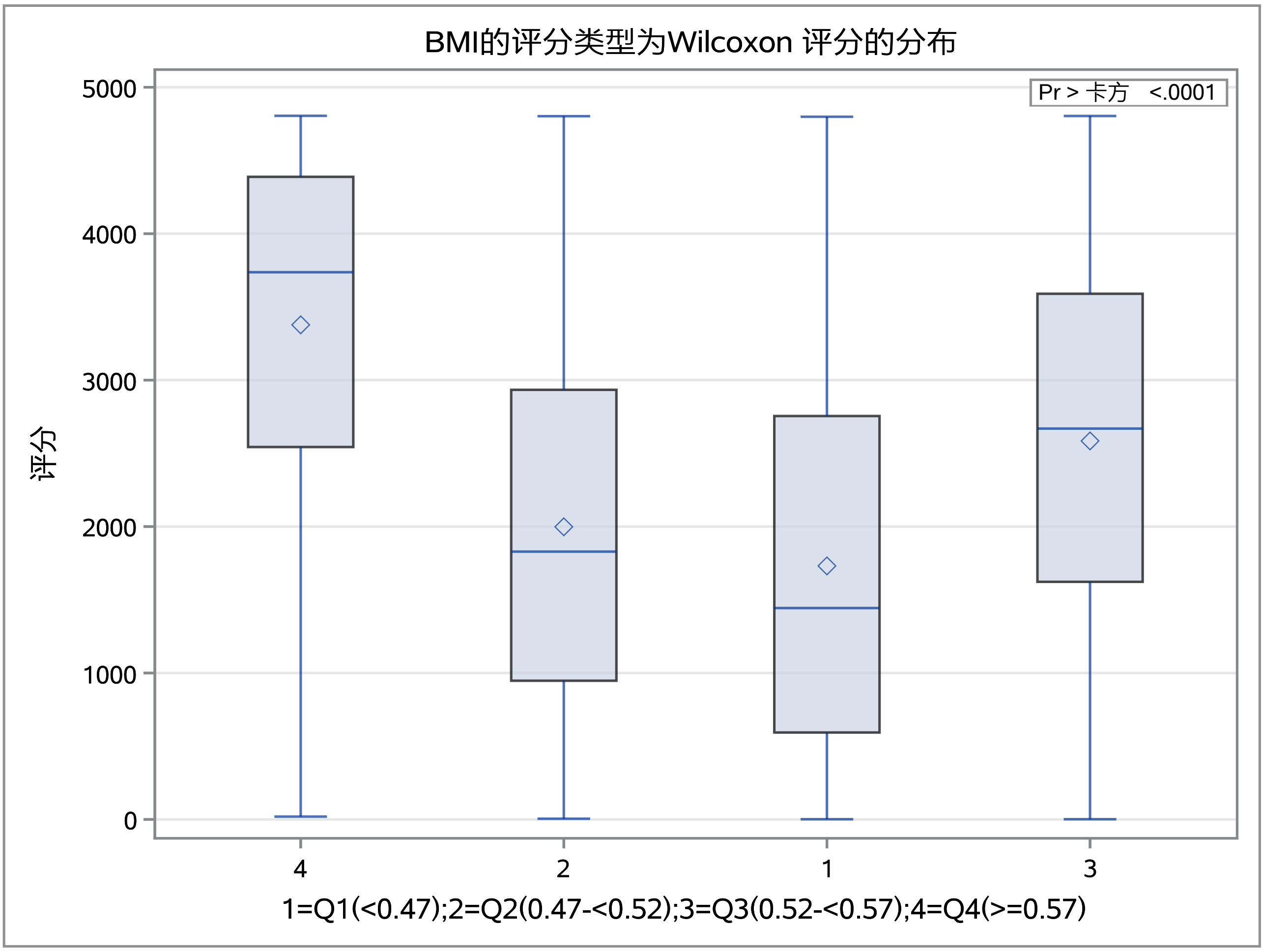

Kruskal-Wallis Test among differenct group

NPAR1WAY 过程

变量 "AGE" 的 Wilcoxon 评分（秩和）按变量 "whtr_g" 分类					
whtr_g	数目	评分汇总	H0 之下的期望值	H0 之下的标准差	均值评分
4	1130	2620769.00	2715390.0	40767.3172	2319.26460
2	1316	3181921.50	3162348.0	42866.9583	2417.87348
1	1169	3023117.00	2809107.0	41244.2520	2586.07100
3	1190	2720607.50	2859570.0	41492.7167	2286.22479
已将平均评分用于结值。					

Kruskal-Wallis 检验		
卡方	自由度	Pr > 卡方
33.0839	3	<.0001

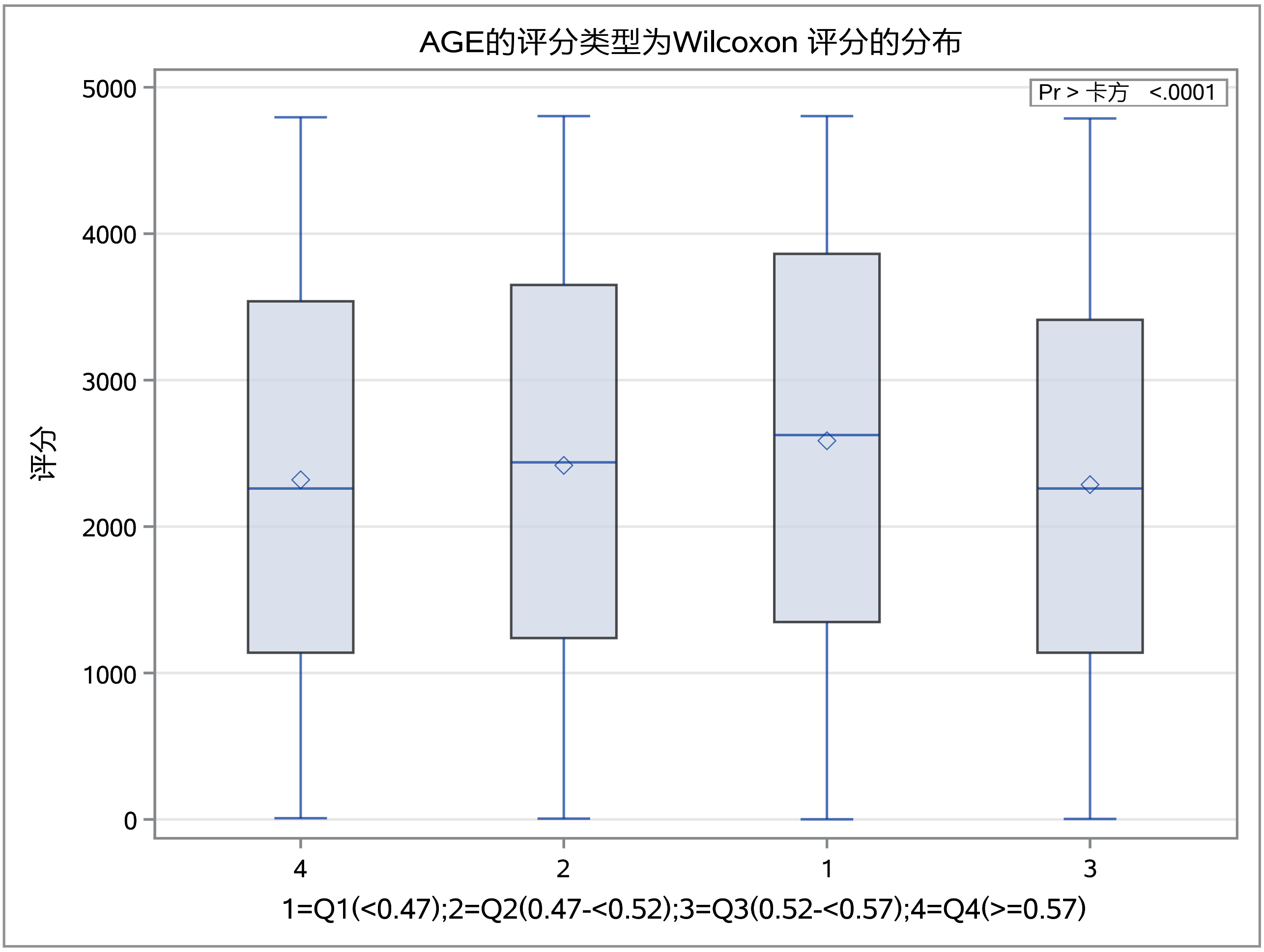

Kruskal-Wallis Test among differenct group

NPAR1WAY 过程

变量“A_NIHSS”的 Wilcoxon 评分（秩和）<br>按变量“whtr_g”分类					
whtr_g	数目	评分汇总	H0 之下的期望值	H0 之下的标准差	均值评分
4	1130	2726689.50	2715390.0	40518.3525	2412.99956
2	1316	3159833.00	3162348.0	42605.1711	2401.08891
1	1169	2718837.00	2809107.0	40992.3747	2325.78015
3	1190	2941055.50	2859570.0	41239.3220	2471.47521
已将平均评分用于结值。					

Kruskal-Wallis 检验		
卡方	自由度	Pr > 卡方
6.6689	3	0.0832

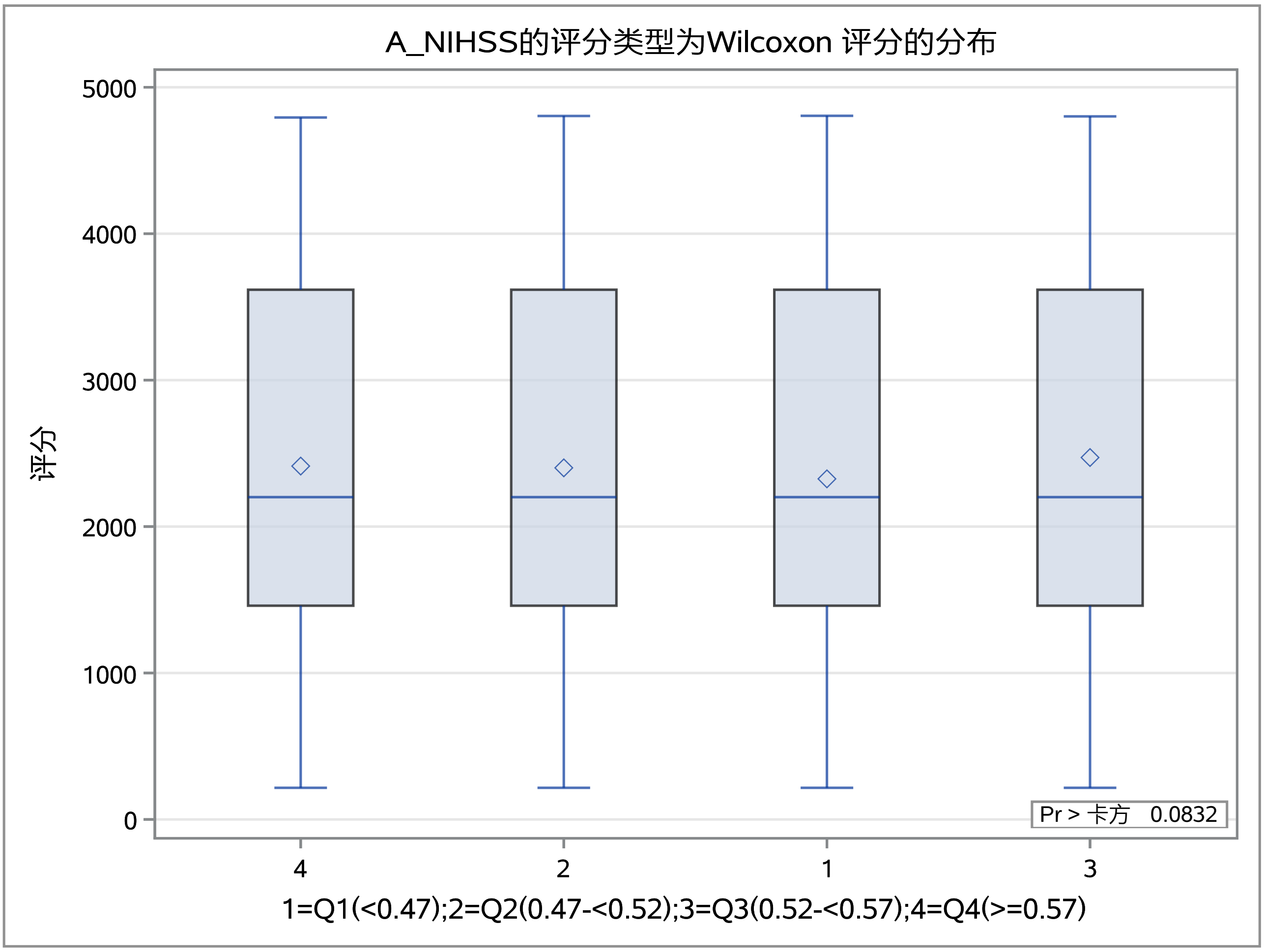

continuous variables,p for linear trend

CORR 过程

2 变量:	BMI whtr_g

简单统计量

变量	数目	均值	标准差	中位数	最小值	最大值	标签
BMI	4805	24.48334	3.42735	24.22100	13.84100	51.07300	F.Physical examination: Body mass index (kg/m2);
whtr_g	4805	2.47471	1.09842	2.00000	1.00000	4.00000	1=Q1(<0.47);2=Q2(0.47-<0.52);3=Q3(0.52-<0.57);4=Q4(>=0.57)

Kendall Tau b 相关系数, N = 4805
Prob > |tau|, H0: Tau=0

	BMI	whtr_g
BMI<br>F.Physical examination: Body mass index (kg/m2);	1.00000	0.33646<br><.0001
whtr_g<br>1=Q1(<0.47);2=Q2(0.47-<0.52);3=Q3(0.52-<0.57);4=Q4(>=0.57)	0.33646<br><.0001	1.00000

continuous variables,p for linear trend

CORR 过程

2 变量:	AGE whtr_g

简单统计量							
变量	数目	均值	标准差	中位数	最小值	最大值	标签
AGE	4805	63.44724	11.36806	64.00000	23.00000	93.00000	A.Basic Information: Age (years old);
whtr_g	4805	2.47471	1.09842	2.00000	1.00000	4.00000	1=Q1(<0.47);2=Q2(0.47-<0.52);3=Q3(0.52-<0.57);4=Q4(>=0.57)

Kendall Tau b 相关系数, N = 4805<br>Prob > |tau|, H0: Tau=0		
	AGE	whtr_g
AGE<br>A.Basic Information: Age (years old);	1.00000	-0.05653<br><.0001
whtr_g<br>1=Q1(<0.47);2=Q2(0.47-<0.52);3=Q3(0.52-<0.57);4=Q4(>=0.57)	-0.05653<br><.0001	1.00000

continuous variables,p for linear trend

CORR 过程

2 变量:	A_NIHSS whtr_g

简单统计量							
变量	数目	均值	标准差	中位数	最小值	最大值	标签
A_NIHSS	4805	4.42789	4.18052	3.00000	0	40.00000	F.Admitting NIHSS: Total score;
whtr_g	4805	2.47471	1.09842	2.00000	1.00000	4.00000	1=Q1(<0.47);2=Q2(0.47-<0.52);3=Q3(0.52-<0.57);4=Q4(>=0.57)

Kendall Tau b 相关系数, N = 4805<br>Prob > |tau|, H0: Tau=0		
	A_NIHSS	whtr_g
A_NIHSS<br>F.Admitting NIHSS: Total score;	1.00000	0.02113<br>0.0609
whtr_g<br>1=Q1(<0.47);2=Q2(0.47-<0.52);3=Q3(0.52-<0.57);4=Q4(>=0.57)	0.02113<br>0.0609	1.00000

categorical variables, descriptive by group and chisq test

FREQ 过程

频数
列百分比

GENDER-whtr_g表

GENDER(A.Basic Information: Gender; 1-male; 2-female;)	whtr_g(1=Q1(<0.47);2=Q2(0.47-<0.52);3=Q3(0.52-<0.57);4=Q4(>=0.57))				
	1	2	3	4	合计
1	824<br>70.49	943<br>71.66	790<br>66.39	657<br>58.14	3214
2	345<br>29.51	373<br>28.34	400<br>33.61	473<br>41.86	1591
合计	1169	1316	1190	1130	4805

表“whtr_g-GENDER”的统计量

统计量	自由度	值	概率
卡方	3	59.5163	<.0001
似然比卡方检验	3	58.5838	<.0001
Mantel-Haenszel 卡方	1	47.0279	<.0001
Phi 系数		0.1113	
列联系数		0.1106	
Cramer V		0.1113	

样本大小 = 4805

频数
列百分比

ETHNIC-whtr_g表

ETHNIC(B.Demography: Race: 1-Han; 99-others;)	whtr_g(1=Q1(<0.47);2=Q2(0.47-<0.52);3=Q3(0.52-<0.57);4=Q4(>=0.57))				
	1	2	3	4	合计
1	1140<br>97.52	1264<br>96.05	1130<br>94.96	1049<br>92.83	4583
2	29<br>2.48	52<br>3.95	60<br>5.04	81<br>7.17	222
合计	1169	1316	1190	1130	4805

表“whtr_g-ETHNIC”的统计量

统计量	自由度	值	概率
卡方	3	30.6059	<.0001
似然比卡方检验	3	30.7220	<.0001
Mantel-Haenszel 卡方	1	30.0465	<.0001
Phi 系数		0.0798	
列联系数		0.0796	
Cramer V		0.0798	

样本大小 = 4805

categorical variables, descriptive by group and chisq test

FREQ 过程

频数
列百分比

H_STROKE01-whtr_g表					
H_STROKE01(D.History: Stroke History; 0-No; 1-Yes;)	whtr_g(1=Q1(<0.47);2=Q2(0.47-<0.52);3=Q3(0.52-<0.57);4=Q4(>=0.57))				
	1	2	3	4	合计
0	977<br>83.58	1047<br>79.56	953<br>80.08	896<br>79.29	3873
1	192<br>16.42	269<br>20.44	237<br>19.92	234<br>20.71	932
合计	1169	1316	1190	1130	4805

表“whtr_g-H_STROKE01”的统计量

统计量	自由度	值	概率
卡方	3	8.9717	0.0297
似然比卡方检验	3	9.2171	0.0265
Mantel-Haenszel 卡方	1	5.4940	0.0191
Phi 系数		0.0432	
列联系数		0.0432	
Cramer V		0.0432	

样本大小 = 4805

频数
列百分比

H_DIAB01-whtr_g表					
H_DIAB01(D.History: Diabetes; 0-No; 1-Yes;)	whtr_g(1=Q1(<0.47);2=Q2(0.47-<0.52);3=Q3(0.52-<0.57);4=Q4(>=0.57))				
	1	2	3	4	合计
0	890<br>76.13	1042<br>79.18	922<br>77.48	844<br>74.69	3698
1	279<br>23.87	274<br>20.82	268<br>22.52	286<br>25.31	1107
合计	1169	1316	1190	1130	4805

表“whtr_g-H_DIAB01”的统计量

统计量	自由度	值	概率
卡方	3	7.5701	0.0558
似然比卡方检验	3	7.5825	0.0555
Mantel-Haenszel 卡方	1	1.2255	0.2683
Phi 系数		0.0397	
列联系数		0.0397	
Cramer V		0.0397	

样本大小 = 4805

categorical variables, descriptive by group and chisq test

FREQ 过程

频数
列百分比

H_AF01-whtr_g表					
H_AF01(D.History: Heart disease category: Atrial fibrillation(Including medical history and hospitalization diagnosis); 0-No; 1-Yes;)	whtr_g(1=Q1(<0.47);2=Q2(0.47-<0.52);3=Q3(0.52-<0.57);4=Q4(>=0.57))				
	1	2	3	4	合计
0	1058<br>90.50	1208<br>91.79	1088<br>91.43	1067<br>94.42	4421
1	111<br>9.50	108<br>8.21	102<br>8.57	63<br>5.58	384
合计	1169	1316	1190	1130	4805

表“whtr_g-H_AF01”的统计量

统计量	自由度	值	概率
卡方	3	13.1948	0.0042
似然比卡方检验	3	13.9747	0.0029
Mantel-Haenszel 卡方	1	9.9999	0.0016
Phi 系数		0.0524	
列联系数		0.0523	
Cramer V		0.0524	

样本大小 = 4805

频数
列百分比

AI-whtr_g表					
AI(history:Myocardial infarction; 0=NO; 1=YES;)	whtr_g(1=Q1(<0.47);2=Q2(0.47-<0.52);3=Q3(0.52-<0.57);4=Q4(>=0.57))				
	1	2	3	4	合计
0	1150<br>98.37	1302<br>98.94	1168<br>98.15	1112<br>98.41	4732
1	19<br>1.63	14<br>1.06	22<br>1.85	18<br>1.59	73
合计	1169	1316	1190	1130	4805

表“whtr_g-AI”的统计量

统计量	自由度	值	概率
卡方	3	2.8167	0.4208
似然比卡方检验	3	2.9710	0.3961
Mantel-Haenszel 卡方	1	0.2177	0.6408
Phi 系数		0.0242	
列联系数		0.0242	
Cramer V		0.0242	

样本大小 = 4805

categorical variables, descriptive by group and chisq test

FREQ 过程

频数
列百分比

H_HYPT01-whtr_g表					
H_HYPT01(D.History: Hypertension; 0-No; 1-Yes;)	whtr_g(1=Q1(<0.47);2=Q2(0.47-<0.52);3=Q3(0.52-<0.57);4=Q4(>=0.57))				
	1	2	3	4	合计
0	458<br>39.18	497<br>37.77	414<br>34.79	329<br>29.12	1698
1	711<br>60.82	819<br>62.23	776<br>65.21	801<br>70.88	3107
合计	1169	1316	1190	1130	4805

表“whtr_g-H_HYPT01”的统计量

统计量	自由度	值	概率
卡方	3	30.2488	<.0001
似然比卡方检验	3	30.6939	<.0001
Mantel-Haenszel 卡方	1	27.8463	<.0001
Phi 系数		0.0793	
列联系数		0.0791	
Cramer V		0.0793	

样本大小 = 4805

频数
列百分比

H_LIPID01-whtr_g表					
H_LIPID01(D.History: Lipid metabolism disorders; 0-No; 1-Yes;)	whtr_g(1=Q1(<0.47);2=Q2(0.47-<0.52);3=Q3(0.52-<0.57);4=Q4(>=0.57))				
	1	2	3	4	合计
0	1105<br>94.53	1245<br>94.60	1096<br>92.10	1029<br>91.06	4475
1	64<br>5.47	71<br>5.40	94<br>7.90	101<br>8.94	330
合计	1169	1316	1190	1130	4805

表“whtr_g-H_LIPID01”的统计量

统计量	自由度	值	概率
卡方	3	17.5597	0.0005
似然比卡方检验	3	17.4404	0.0006
Mantel-Haenszel 卡方	1	15.3091	<.0001
Phi 系数		0.0605	
列联系数		0.0603	
Cramer V		0.0605	

样本大小 = 4805

categorical variables, descriptive by group and chisq test

FREQ 过程

频数
列百分比

H_DRINK_H01-whtr_g表					
H_DRINK_H01(D.History: Heavy Drinking(Alcohol consumption>=20g/day); 0-No,1-Yes;)	whtr_g(1=Q1(<0.47);2=Q2(0.47-<0.52);3=Q3(0.52-<0.57);4=Q4(>=0.57))				
	1	2	3	4	合计
0	1035<br>88.54	1128<br>85.71	1050<br>88.24	1000<br>88.50	4213
1	134<br>11.46	188<br>14.29	140<br>11.76	130<br>11.50	592
合计	1169	1316	1190	1130	4805

表“whtr_g-H_DRINK_H01”的统计量

统计量	自由度	值	概率
卡方	3	6.5379	0.0882
似然比卡方检验	3	6.3736	0.0948
Mantel-Haenszel 卡方	1	0.3607	0.5481
Phi 系数		0.0369	
列联系数		0.0369	
Cramer V		0.0369	

样本大小 = 4805

频数
列百分比

H_SMK_C01-whtr_g表					
H_SMK_C01(D.History: Current Smoking; 0-No,1-Yes;)	whtr_g(1=Q1(<0.47);2=Q2(0.47-<0.52);3=Q3(0.52-<0.57);4=Q4(>=0.57))				
	1	2	3	4	合计
0	854<br>73.05	897<br>68.16	831<br>69.83	834<br>73.81	3416
1	315<br>26.95	419<br>31.84	359<br>30.17	296<br>26.19	1389
合计	1169	1316	1190	1130	4805

表“whtr_g-H_SMK_C01”的统计量

统计量	自由度	值	概率
卡方	3	12.6577	0.0054
似然比卡方检验	3	12.6569	0.0054
Mantel-Haenszel 卡方	1	0.4587	0.4982
Phi 系数		0.0513	
列联系数		0.0513	
Cramer V		0.0513	

样本大小 = 4805

categorical variables, descriptive by group and chisq test

FREQ 过程

频数<br>列百分比	IT-whtr_g表				
IT(intravenous thrombolysis, 1=YES,0=NO)	whtr_g(1=Q1(<0.47);2=Q2(0.47-<0.52);3=Q3(0.52-<0.57);4=Q4(>=0.57))				
	1	2	3	4	合计
0	1026<br>87.77	1145<br>87.01	1062<br>89.24	1036<br>91.68	4269
1	143<br>12.23	171<br>12.99	128<br>10.76	94<br>8.32	536
合计	1169	1316	1190	1130	4805

表“whtr_g-IT”的统计量

统计量	自由度	值	概率
卡方	3	15.2241	0.0016
似然比卡方检验	3	15.7742	0.0013
Mantel-Haenszel 卡方	1	11.5454	0.0007
Phi 系数		0.0563	
列联系数		0.0562	
Cramer V		0.0563	

样本大小 = 4805

频数<br>列百分比	ET-whtr_g表				
ET(动脉溶栓或机械取栓, 1=YES,0=NO)	whtr_g(1=Q1(<0.47);2=Q2(0.47-<0.52);3=Q3(0.52-<0.57);4=Q4(>=0.57))				
	1	2	3	4	合计
0	1157<br>98.97	1306<br>99.24	1186<br>99.66	1128<br>99.82	4777
1	12<br>1.03	10<br>0.76	4<br>0.34	2<br>0.18	28
合计	1169	1316	1190	1130	4805

表“whtr_g-ET”的统计量

统计量	自由度	值	概率
卡方	3	9.1471	0.0274
似然比卡方检验	3	9.7831	0.0205
Mantel-Haenszel 卡方	1	8.9029	0.0028
Phi 系数		0.0436	
列联系数		0.0436	
Cramer V		0.0436	

样本大小 = 4805

categorical variables, descriptive by group and chisq test

FREQ 过程

频数
列百分比

IMG_C_TOAST-whtr_g表

IMG_C_TOAST(K.Final diagnosis: cerebral infarction; Etiology according to TOAST system; 1-large artery atherosclerosis; 2-cardiogenic embolism; 3-small artery occlusion; 4-stroke of another determined cause; 5-stroke of an undetermined cause.)	whtr_g(1=Q1(<0.47);2=Q2(0.47-<0.52);3=Q3(0.52-<0.57);4=Q4(>=0.57))				
	1	2	3	4	合计
1	281<br>24.04	347<br>26.37	298<br>25.04	298<br>26.37	1224
2	97<br>8.30	91<br>6.91	94<br>7.90	59<br>5.22	341
3	233<br>19.93	270<br>20.52	269<br>22.61	276<br>24.42	1048
4	21<br>1.80	21<br>1.60	20<br>1.68	15<br>1.33	77
5	537<br>45.94	587<br>44.60	509<br>42.77	482<br>42.65	2115
合计	1169	1316	1190	1130	4805

表“whtr_g-IMG_C_TOAST”的统计量

统计量	自由度	值	概率
卡方	12	20.5395	0.0575
似然比卡方检验	12	20.9155	0.0516
Mantel-Haenszel 卡方	1	1.6083	0.2047
Phi 系数		0.0654	
列联系数		0.0652	
Cramer V		0.0377	

样本大小 = 4805

频数
列百分比

y1_stroke-whtr_g表

y1_stroke(N12.Follow-up events at 12 months: Recurrence of stroke: 0-No; 1-Yes;)	whtr_g(1=Q1(<0.47);2=Q2(0.47-<0.52);3=Q3(0.52-<0.57);4=Q4(>=0.57))				
	1	2	3	4	合计
0	1058<br>90.50	1171<br>88.98	1089<br>91.51	998<br>88.32	4316
1	111<br>9.50	145<br>11.02	101<br>8.49	132<br>11.68	489
合计	1169	1316	1190	1130	4805

categorical variables, descriptive by group and chisq test

FREQ 过程

表“whtr_g-y1_stroke”的统计量

统计量	自由度	值	概率
卡方	3	8.1272	0.0435
似然比卡方检验	3	8.2010	0.0420
Mantel-Haenszel 卡方	1	0.9021	0.3422
Phi 系数		0.0411	
列联系数		0.0411	
Cramer V		0.0411	

样本大小 = 4805

频数
列百分比

y1_is-whtr_g表

y1_is(N12.Follow-up events at 12 months: recurrence of ischemic stroke: 0-No; 1-Yes;)	whtr_g(1=Q1(<0.47);2=Q2(0.47-<0.52);3=Q3(0.52-<0.57);4=Q4(>=0.57))				
	1	2	3	4	合计
0	1068<br>91.36	1185<br>90.05	1097<br>92.18	1010<br>89.38	4360
1	101<br>8.64	131<br>9.95	93<br>7.82	120<br>10.62	445
合计	1169	1316	1190	1130	4805

表“whtr_g-y1_is”的统计量

统计量	自由度	值	概率
卡方	3	6.7316	0.0810
似然比卡方检验	3	6.7755	0.0794
Mantel-Haenszel 卡方	1	0.8840	0.3471
Phi 系数		0.0374	
列联系数		0.0374	
Cramer V		0.0374	

样本大小 = 4805

频数
列百分比

y1_HS-whtr_g表

y1_HS(N12.Follow-up events at 12 months: recurrence of hemorrhage stroke: 0-No; 1-Yes;)	whtr_g(1=Q1(<0.47);2=Q2(0.47-<0.52);3=Q3(0.52-<0.57);4=Q4(>=0.57))				
	1	2	3	4	合计
0	1158<br>99.06	1302<br>98.94	1180<br>99.16	1115<br>98.67	4755
1	11<br>0.94	14<br>1.06	10<br>0.84	15<br>1.33	50
合计	1169	1316	1190	1130	4805

categorical variables, descriptive by group and chisq test

FREQ 过程

表“whtr_g-y1_HS”的统计量

统计量	自由度	值	概率
卡方	3	1.4859	0.6855
似然比卡方检验	3	1.4498	0.6939
Mantel-Haenszel 卡方	1	0.4642	0.4957
Phi 系数		0.0176	
列联系数		0.0176	
Cramer V		0.0176	

样本大小 = 4805

频数
列百分比

I_IS_HT-whtr_g表					
I_IS_HT(I.Inpatient Event: Hemorrhagic transformation after cerebral infarction; 1-No; 2-Yes; 98-UK;)	whtr_g(1=Q1(<0.47);2=Q2(0.47-<0.52);3=Q3(0.52-<0.57);4=Q4(>=0.57))				
	1	2	3	4	合计
1	1081<br>97.56	1286<br>97.79	1173<br>98.57	1119<br>99.20	4659
2	27<br>2.44	29<br>2.21	17<br>1.43	9<br>0.80	82
合计	1108	1315	1190	1128	4741
频数缺失 = 64					

表“whtr_g-I_IS_HT”的统计量

统计量	自由度	值	概率
卡方	3	11.4072	0.0097
似然比卡方检验	3	12.3745	0.0062
Mantel-Haenszel 卡方	1	10.9410	0.0009
Phi 系数		0.0491	
列联系数		0.0490	
Cramer V		0.0491	

样本大小 = 4741
频数缺失 = 64

频数
列百分比

death_cvd-whtr_g表					
death_cvd(1年心血管源性死亡，0=NO; 1=YES)	whtr_g(1=Q1(<0.47);2=Q2(0.47-<0.52);3=Q3(0.52-<0.57);4=Q4(>=0.57))				
	1	2	3	4	合计
0	1151<br>98.46	1287<br>97.80	1182<br>99.33	1120<br>99.12	4740
1	18<br>1.54	29<br>2.20	8<br>0.67	10<br>0.88	65
合计	1169	1316	1190	1130	4805

categorical variables, descriptive by group and chisq test

FREQ 过程

表“whtr_g-death_cvd”的统计量

统计量	自由度	值	概率
卡方	3	13.4289	0.0038
似然比卡方检验	3	13.4706	0.0037
Mantel-Haenszel 卡方	1	5.6227	0.0177
Phi 系数		0.0529	
列联系数		0.0528	
Cramer V		0.0529	

样本大小 = 4805

频数<br>列百分比	y1_comb-whtr_g表				
y1_comb(N12.Follow-up events at 12 months:Occurrence of combined vascular event(including cardiovascular death,non-fatal stroke,non-fatal myocardial infarction):0-No;1-Yes;)	whtr_g(1=Q1(<0.47);2=Q2(0.47-<0.52);3=Q3(0.52-<0.57);4=Q4(>=0.57))				
	1	2	3	4	合计
0	1054<br>90.16	1158<br>87.99	1083<br>91.01	990<br>87.61	4285
1	115<br>9.84	158<br>12.01	107<br>8.99	140<br>12.39	520
合计	1169	1316	1190	1130	4805

表“whtr_g-y1_comb”的统计量

统计量	自由度	值	概率
卡方	3	10.0936	0.0178
似然比卡方检验	3	10.1789	0.0171
Mantel-Haenszel 卡方	1	1.1304	0.2877
Phi 系数		0.0458	
列联系数		0.0458	
Cramer V		0.0458	

样本大小 = 4805

频数<br>列百分比	y1_death-whtr_g表				
y1_death(N12.Follow-up events at 12 months: Whether the patient died: 0-survival;1-death;)	whtr_g(1=Q1(<0.47);2=Q2(0.47-<0.52);3=Q3(0.52-<0.57);4=Q4(>=0.57))				
	1	2	3	4	合计
0	1102<br>94.27	1253<br>95.21	1165<br>97.90	1095<br>96.90	4615
1	67<br>5.73	63<br>4.79	25<br>2.10	35<br>3.10	190
合计	1169	1316	1190	1130	4805

0.00608 0.00503 0.00215249 0.0032

categorical variables, descriptive by group and chisq test

FREQ 过程

表“whtr_g-y1_death”的统计量

统计量	自由度	值	概率
卡方	3	25.0738	<.0001
似然比卡方检验	3	26.0976	<.0001
Mantel-Haenszel 卡方	1	17.5691	<.0001
Phi 系数		0.0722	
列联系数		0.0720	
Cramer V		0.0722	

样本大小 = 4805

categorical variables, descriptive by group and P for linear trend

FREQ 过程

频数
列百分比

GENDER-whtr_g表					
GENDER(A.Basic Information: Gender; 1-male; 2-female;)	whtr_g(1=Q1(<0.47);2=Q2(0.47-<0.52);3=Q3(0.52-<0.57);4=Q4(>=0.57))				
	1	2	3	4	合计
1	824<br>70.49	943<br>71.66	790<br>66.39	657<br>58.14	3214
2	345<br>29.51	373<br>28.34	400<br>33.61	473<br>41.86	1591
合计	1169	1316	1190	1130	4805

表“whtr_g-GENDER”的统计量

Cochran-Armitage 趋势检验	
统计量 (Z)	-6.8584
单侧 Pr < Z	<.0001
双侧 Pr > |Z|	<.0001

样本大小 = 4805

频数
列百分比

ETHNIC-whtr_g表					
ETHNIC(B.Demography: Race: 1-Han; 99-others;)	whtr_g(1=Q1(<0.47);2=Q2(0.47-<0.52);3=Q3(0.52-<0.57);4=Q4(>=0.57))				
	1	2	3	4	合计
1	1140<br>97.52	1264<br>96.05	1130<br>94.96	1049<br>92.83	4583
2	29<br>2.48	52<br>3.95	60<br>5.04	81<br>7.17	222
合计	1169	1316	1190	1130	4805

表“whtr_g-ETHNIC”的统计量

Cochran-Armitage 趋势检验	
统计量 (Z)	-5.4820
单侧 Pr < Z	<.0001
双侧 Pr > |Z|	<.0001

样本大小 = 4805

频数
列百分比

H_STROKE01-whtr_g表					
H_STROKE01(D.History: Stroke History; 0-No; 1-Yes;)	whtr_g(1=Q1(<0.47);2=Q2(0.47-<0.52);3=Q3(0.52-<0.57);4=Q4(>=0.57))				
	1	2	3	4	合计
0	977<br>83.58	1047<br>79.56	953<br>80.08	896<br>79.29	3873
1	192<br>16.42	269<br>20.44	237<br>19.92	234<br>20.71	932
合计	1169	1316	1190	1130	4805

categorical variables, descriptive by group and P for linear trend

FREQ 过程

表“whtr_g-H_STROKE01”的统计量

Cochran-Armitage 趋势检验	
统计量 (Z)	-2.3442
单侧 Pr < Z	0.0095
双侧 Pr > |Z|	0.0191

样本大小 = 4805

频数 列百分比

H_DIAB01-whtr_g表

H_DIAB01(D.History: Diabetes; 0-No; 1-Yes;)	whtr_g(1=Q1(<0.47);2=Q2(0.47-<0.52);3=Q3(0.52-<0.57);4=Q4(>=0.57))				
	1	2	3	4	合计
0	890<br>76.13	1042<br>79.18	922<br>77.48	844<br>74.69	3698
1	279<br>23.87	274<br>20.82	268<br>22.52	286<br>25.31	1107
合计	1169	1316	1190	1130	4805

表“whtr_g-H_DIAB01”的统计量

Cochran-Armitage 趋势检验	
统计量 (Z)	-1.1071
单侧 Pr < Z	0.1341
双侧 Pr > |Z|	0.2682

样本大小 = 4805

频数 列百分比

H_AF01-whtr_g表

H_AF01(D.History: Heart disease category: Atrial fibrillation(Including medical history and hospitalization diagnosis); 0-No; 1-Yes;)	whtr_g(1=Q1(<0.47);2=Q2(0.47-<0.52);3=Q3(0.52-<0.57);4=Q4(>=0.57))				
	1	2	3	4	合计
0	1058<br>90.50	1208<br>91.79	1088<br>91.43	1067<br>94.42	4421
1	111<br>9.50	108<br>8.21	102<br>8.57	63<br>5.58	384
合计	1169	1316	1190	1130	4805

表“whtr_g-H_AF01”的统计量

Cochran-Armitage 趋势检验	
统计量 (Z)	3.1626
单侧 Pr > Z	0.0008
双侧 Pr > |Z|	0.0016

样本大小 = 4805

categorical variables, descriptive by group and P for linear trend

FREQ 过程

频数<br>列百分比	AI-whtr_g表				
AI(history:Myocardial infarction; 0=NO; 1=YES;)	whtr_g(1=Q1(<0.47);2=Q2(0.47-<0.52);3=Q3(0.52-<0.57);4=Q4(>=0.57))				
	1	2	3	4	合计
0	1150<br>98.37	1302<br>98.94	1168<br>98.15	1112<br>98.41	4732
1	19<br>1.63	14<br>1.06	22<br>1.85	18<br>1.59	73
合计	1169	1316	1190	1130	4805

表“whtr_g-AI”的统计量

Cochran-Armitage 趋势检验	
统计量 (Z)	-0.4667
单侧 Pr < Z	0.3204
双侧 Pr > |Z|	0.6407

样本大小 = 4805

频数<br>列百分比	H_HYPT01-whtr_g表				
H_HYPT01(D.History: Hypertension; 0-No; 1-Yes;)	whtr_g(1=Q1(<0.47);2=Q2(0.47-<0.52);3=Q3(0.52-<0.57);4=Q4(>=0.57))				
	1	2	3	4	合计
0	458<br>39.18	497<br>37.77	414<br>34.79	329<br>29.12	1698
1	711<br>60.82	819<br>62.23	776<br>65.21	801<br>70.88	3107
合计	1169	1316	1190	1130	4805

表“whtr_g-H_HYPT01”的统计量

Cochran-Armitage 趋势检验	
统计量 (Z)	-5.2775
单侧 Pr < Z	<.0001
双侧 Pr > |Z|	<.0001

样本大小 = 4805

频数<br>列百分比	H_LIPID01-whtr_g表				
H_LIPID01(D.History: Lipid metabolism disorders; 0-No; 1-Yes;)	whtr_g(1=Q1(<0.47);2=Q2(0.47-<0.52);3=Q3(0.52-<0.57);4=Q4(>=0.57))				
	1	2	3	4	合计
0	1105<br>94.53	1245<br>94.60	1096<br>92.10	1029<br>91.06	4475
1	64<br>5.47	71<br>5.40	94<br>7.90	101<br>8.94	330
合计	1169	1316	1190	1130	4805

categorical variables, descriptive by group and P for linear trend

FREQ 过程

表“whtr_g-H_LIPID01”的统计量

Cochran-Armitage 趋势检验	
统计量 (Z)	-3.9131
单侧 Pr < Z	<.0001
双侧 Pr > |Z|	<.0001

样本大小 = 4805

频数
列百分比

H_DRINK_H01-whtr_g表

H_DRINK_H01(D.History: Heavy Drinking(Alcohol consumption>=20g/day); 0-No,1-Yes;)	whtr_g(1=Q1(<0.47);2=Q2(0.47-<0.52);3=Q3(0.52-<0.57);4=Q4(>=0.57))				
	1	2	3	4	合计
0	1035<br>88.54	1128<br>85.71	1050<br>88.24	1000<br>88.50	4213
1	134<br>11.46	188<br>14.29	140<br>11.76	130<br>11.50	592
合计	1169	1316	1190	1130	4805

表“whtr_g-H_DRINK_H01”的统计量

Cochran-Armitage 趋势检验	
统计量 (Z)	0.6007
单侧 Pr > Z	0.2740
双侧 Pr > |Z|	0.5481

样本大小 = 4805

频数
列百分比

H_SMK_C01-whtr_g表

H_SMK_C01(D.History: Current Smoking; 0-No,1-Yes;)	whtr_g(1=Q1(<0.47);2=Q2(0.47-<0.52);3=Q3(0.52-<0.57);4=Q4(>=0.57))				
	1	2	3	4	合计
0	854<br>73.05	897<br>68.16	831<br>69.83	834<br>73.81	3416
1	315<br>26.95	419<br>31.84	359<br>30.17	296<br>26.19	1389
合计	1169	1316	1190	1130	4805

表“whtr_g-H_SMK_C01”的统计量

Cochran-Armitage 趋势检验	
统计量 (Z)	0.6773
单侧 Pr > Z	0.2491
双侧 Pr > |Z|	0.4982

样本大小 = 4805

categorical variables, descriptive by group and P for linear trend

FREQ 过程

频数
列百分比

IT-whtr_g表					
IT(intravenous thrombolysis, 1=YES,0=NO)	whtr_g(1=Q1(<0.47);2=Q2(0.47-<0.52);3=Q3(0.52-<0.57);4=Q4(>=0.57))				
	1	2	3	4	合计
0	1026<br>87.77	1145<br>87.01	1062<br>89.24	1036<br>91.68	4269
1	143<br>12.23	171<br>12.99	128<br>10.76	94<br>8.32	536
合计	1169	1316	1190	1130	4805

表“whtr_g-IT”的统计量

Cochran-Armitage 趋势检验	
统计量 (Z)	3.3982
单侧 Pr > Z	0.0003
双侧 Pr > |Z|	0.0007

样本大小 = 4805

频数
列百分比

ET-whtr_g表					
ET(动脉溶栓或机械取栓, 1=YES,0=NO)	whtr_g(1=Q1(<0.47);2=Q2(0.47-<0.52);3=Q3(0.52-<0.57);4=Q4(>=0.57))				
	1	2	3	4	合计
0	1157<br>98.97	1306<br>99.24	1186<br>99.66	1128<br>99.82	4777
1	12<br>1.03	10<br>0.76	4<br>0.34	2<br>0.18	28
合计	1169	1316	1190	1130	4805

表“whtr_g-ET”的统计量

Cochran-Armitage 趋势检验	
统计量 (Z)	2.9841
单侧 Pr > Z	0.0014
双侧 Pr > |Z|	0.0028

样本大小 = 4805

categorical variables, descriptive by group and P for linear trend

FREQ 过程

频数
列百分比

IMG_C_TOAST-whtr_g表

IMG_C_TOAST(K.Final diagnosis: cerebral infarction; Etiology according to TOAST system; 1-large artery atherosclerosis; 2-cardiogenic embolism; 3-small artery occlusion; 4-stroke of another determined cause; 5-stroke of an undetermined cause.)	whtr_g(1=Q1(<0.47);2=Q2(0.47-<0.52);3=Q3(0.52-<0.57);4=Q4(>=0.57))				
	1	2	3	4	合计
1	281<br>24.04	347<br>26.37	298<br>25.04	298<br>26.37	1224
2	97<br>8.30	91<br>6.91	94<br>7.90	59<br>5.22	341
3	233<br>19.93	270<br>20.52	269<br>22.61	276<br>24.42	1048
4	21<br>1.80	21<br>1.60	20<br>1.68	15<br>1.33	77
5	537<br>45.94	587<br>44.60	509<br>42.77	482<br>42.65	2115
合计	1169	1316	1190	1130	4805

频数
列百分比

y1_stroke-whtr_g表

y1_stroke(N12.Follow-up events at 12 months: Recurrence of stroke: 0-No; 1-Yes;)	whtr_g(1=Q1(<0.47);2=Q2(0.47-<0.52);3=Q3(0.52-<0.57);4=Q4(>=0.57))				
	1	2	3	4	合计
0	1058<br>90.50	1171<br>88.98	1089<br>91.51	998<br>88.32	4316
1	111<br>9.50	145<br>11.02	101<br>8.49	132<br>11.68	489
合计	1169	1316	1190	1130	4805

表“whtr_g-y1_stroke”的统计量

Cochran-Armitage 趋势检验	
统计量 (Z)	-0.9499
单侧 Pr < Z	0.1711
双侧 Pr > |Z|	0.3422

样本大小 = 4805

categorical variables, descriptive by group and P for linear trend

FREQ 过程

频数<br>列百分比	y1_is-whtr_g表				
y1_is(N12.Follow-up events at 12 months: recurrence of ischemic stroke: 0-No; 1-Yes;)	whtr_g(1=Q1(<0.47);2=Q2(0.47-<0.52);3=Q3(0.52-<0.57);4=Q4(>=0.57))				
	1	2	3	4	合计
0	1068<br>91.36	1185<br>90.05	1097<br>92.18	1010<br>89.38	4360
1	101<br>8.64	131<br>9.95	93<br>7.82	120<br>10.62	445
合计	1169	1316	1190	1130	4805

表“whtr_g-y1_is”的统计量

Cochran-Armitage 趋势检验	
统计量 (Z)	-0.9403
单侧 Pr < Z	0.1735
双侧 Pr > |Z|	0.3471

样本大小 = 4805

频数<br>列百分比	y1_HS-whtr_g表				
y1_HS(N12.Follow-up events at 12 months: recurrence of hemorrhage stroke: 0-No; 1-Yes;)	whtr_g(1=Q1(<0.47);2=Q2(0.47-<0.52);3=Q3(0.52-<0.57);4=Q4(>=0.57))				
	1	2	3	4	合计
0	1158<br>99.06	1302<br>98.94	1180<br>99.16	1115<br>98.67	4755
1	11<br>0.94	14<br>1.06	10<br>0.84	15<br>1.33	50
合计	1169	1316	1190	1130	4805

表“whtr_g-y1_HS”的统计量

Cochran-Armitage 趋势检验	
统计量 (Z)	-0.6814
单侧 Pr < Z	0.2478
双侧 Pr > |Z|	0.4956

样本大小 = 4805

categorical variables, descriptive by group and P for linear trend

FREQ 过程

频数<br>列百分比

I_IS_HT-whtr_g表

I_IS_HT(I.Inpatient Event: Hemorrhagic transformation after cerebral infarction; 1-No; 2-Yes; 98-UK;)	whtr_g(1=Q1(<0.47);2=Q2(0.47-<0.52);3=Q3(0.52-<0.57);4=Q4(>=0.57))				
	1	2	3	4	合计
1	1081<br>97.56	1286<br>97.79	1173<br>98.57	1119<br>99.20	4659
2	27<br>2.44	29<br>2.21	17<br>1.43	9<br>0.80	82
合计	1108	1315	1190	1128	4741
频数缺失 = 64					

表“whtr_g-I_IS_HT”的统计量

Cochran-Armitage<br>趋势检验	
统计量 (Z)	3.3081
单侧 Pr > Z	0.0005
双侧 Pr > |Z|	0.0009

样本大小 = 4741
频数缺失 = 64

频数<br>列百分比

death_cvd-whtr_g表

death_cvd(1年心血管源性死亡，0=NO; 1=YES)	whtr_g(1=Q1(<0.47);2=Q2(0.47-<0.52);3=Q3(0.52-<0.57);4=Q4(>=0.57))				
	1	2	3	4	合计
0	1151<br>98.46	1287<br>97.80	1182<br>99.33	1120<br>99.12	4740
1	18<br>1.54	29<br>2.20	8<br>0.67	10<br>0.88	65
合计	1169	1316	1190	1130	4805

表“whtr_g-death_cvd”的统计量

Cochran-Armitage<br>趋势检验	
统计量 (Z)	2.3715
单侧 Pr > Z	0.0089
双侧 Pr > |Z|	0.0177

样本大小 = 4805

categorical variables, descriptive by group and P for linear trend

FREQ 过程

频数
列百分比

y1_comb-whtr_g表					
y1_comb(N12.Follow-up events at 12 months:Occurrence of combined vascular event(including cardiovascular death,non-fatal stroke,non-fatal myocardial infarction):0-No;1-Yes;)	whtr_g(1=Q1(<0.47);2=Q2(0.47-<0.52);3=Q3(0.52-<0.57);4=Q4(>=0.57))				
	1	2	3	4	合计
0	1054<br>90.16	1158<br>87.99	1083<br>91.01	990<br>87.61	4285
1	115<br>9.84	158<br>12.01	107<br>8.99	140<br>12.39	520
合计	1169	1316	1190	1130	4805

表“whtr_g-y1_comb”的统计量

Cochran-Armitage 趋势检验	
统计量 (Z)	-1.0633
单侧 Pr < Z	0.1438
双侧 Pr > |Z|	0.2876

样本大小 = 4805

频数
列百分比

y1_death-whtr_g表					
y1_death(N12.Follow-up events at 12 months: Whether the patient died: 0-survival;1-death;)	whtr_g(1=Q1(<0.47);2=Q2(0.47-<0.52);3=Q3(0.52-<0.57);4=Q4(>=0.57))				
	1	2	3	4	合计
0	1102<br>94.27	1253<br>95.21	1165<br>97.90	1095<br>96.90	4615
1	67<br>5.73	63<br>4.79	25<br>2.10	35<br>3.10	190
合计	1169	1316	1190	1130	4805

表“whtr_g-y1_death”的统计量

Cochran-Armitage 趋势检验	
统计量 (Z)	4.1920
单侧 Pr > Z	<.0001
双侧 Pr > |Z|	<.0001

样本大小 = 4805
