# Supplementary material for: Association of body mass index and waist-to-height ratio with outcomes in ischemic stroke: results from the Third China National Stroke Registry
Source: BMC Neurol. 2023 Apr 14;23:152. doi: 10.1186/s12883-023-03165-y (PMC10103413; doi:10.1186/s12883-023-03165-y)
Supplement: Supplementary file 2 — Additional file 2. [file 12883_2023_3165_MOESM2_ESM.zip › raw data/Table1.pdf]

FREQ 过程

1=<18.5;2=18.5-<23;3=23-<27.5;4=≥27.5				
BMI_g	频数	百分比	累积频数	累积百分比
1	309	2.18	309	2.18
2	3855	27.25	4164	29.44
3	7491	52.95	11655	82.39
4	2491	17.61	14146	100.00

MEANS PROCEDURE

变量	标签	数目	缺失值个数	均值	标准差	最小值	下四分位数	中位数	上四分位数	最大值
WAIST	F.Physical examination: Waist circumference (cm) ;	4805	9341	85.5727367	14.1756542	30.0000000	78.0000000	86.0000000	94.0000000	170.0000000
AGE	A.Basic Information: Age (years old);	14146	0	62.3126679	11.2954852	19.0000000	54.0000000	63.0000000	70.0000000	96.0000000
A_NIHSS	F.Admitting NIHSS: Total score;	14146	0	4.4148876	4.1781470	0	2.0000000	3.0000000	6.0000000	40.0000000

FREQ 过程

A.Basic Information: Gender; 1-male; 2-female;				
GENDER	频数	百分比	累积频数	累积百分比
1	9720	68.71	9720	68.71
2	4426	31.29	14146	100.00

B.Demography: Race: 1-Han; 99-others;				
ETHNIC	频数	百分比	累积频数	累积百分比
1	13730	97.06	13730	97.06
2	416	2.94	14146	100.00

D.History: Stroke History; 0-No; 1-Yes;				
H_STROKE01	频数	百分比	累积频数	累积百分比
0	11012	77.85	11012	77.85
1	3134	22.15	14146	100.00

D.History: Diabetes; 0-No; 1-Yes;				
H_DIAB01	频数	百分比	累积频数	累积百分比
0	10836	76.60	10836	76.60
1	3310	23.40	14146	100.00

D.History: Heart disease category: Atrial fibrillation(Including medical history and hospitalization diagnosis); 0-No; 1-Yes;				
H_AF01	频数	百分比	累积频数	累积百分比
0	13160	93.03	13160	93.03
1	986	6.97	14146	100.00

history:Myocardial infarction; 0=NO; 1=YES;				
AI	频数	百分比	累积频数	累积百分比
0	13868	98.03	13868	98.03
1	278	1.97	14146	100.00

D.History: Hypertension; 0-No; 1-Yes;				
H_HYPT01	频数	百分比	累积频数	累积百分比
0	5259	37.18	5259	37.18
1	8887	62.82	14146	100.00

FREQ 过程

D.History: Lipid metabolism disorders; 0-No; 1-Yes;				
H_LIPID01	频数	百分比	累积频数	累积百分比
0	13071	92.40	13071	92.40
1	1075	7.60	14146	100.00

D.History: Heavy Drinking(Alcohol consumption>=20g/day); 0-No,1-Yes;				
H_DRINK_H01	频数	百分比	累积频数	累积百分比
0	12136	85.79	12136	85.79
1	2010	14.21	14146	100.00

D.History: Current Smoking; 0-No,1-Yes;				
H_SMK_C01	频数	百分比	累积频数	累积百分比
0	9643	68.17	9643	68.17
1	4503	31.83	14146	100.00

intravenous thrombolysis, 1=YES,0=NO				
IT	频数	百分比	累积频数	累积百分比
0	12626	89.25	12626	89.25
1	1520	10.75	14146	100.00

动脉溶栓或机械取栓, 1=YES,0=NO				
ET	频数	百分比	累积频数	累积百分比
0	14075	99.50	14075	99.50
1	71	0.50	14146	100.00

K.Final diagnosis: cerebral infarction; Etiology according to TOAST system; 1-large artery atherosclerosis; 2-cardiogenic embolism; 3-small artery occlusion; 4-stroke of another determined cause; 5-stroke of an undetermined cause.				
IMG_C_TOAST	频数	百分比	累积频数	累积百分比
1	3667	25.92	3667	25.92
2	881	6.23	4548	32.15
3	3137	22.18	7685	54.33
4	171	1.21	7856	55.54
5	6290	44.46	14146	100.00

FREQ 过程

N12.Follow-up events at 12 months: Recurrence of stroke: 0-No; 1-Yes;				
y1_stroke	频数	百分比	累积频数	累积百分比
0	12722	89.93	12722	89.93
1	1424	10.07	14146	100.00

N12.Follow-up events at 12 months: recurrence of ischemic stroke: 0-No; 1-Yes;				
y1_is	频数	百分比	累积频数	累积百分比
0	12829	90.69	12829	90.69
1	1317	9.31	14146	100.00

N12.Follow-up events at 12 months: recurrence of hemorrhage stroke: 0-No; 1-Yes;				
y1_HS	频数	百分比	累积频数	累积百分比
0	14022	99.12	14022	99.12
1	124	0.88	14146	100.00

I.Inpatient Event: Hemorrhagic transformation after cerebral infarction; 1-No; 2-Yes; 98-UK;				
I_IS_HT	频数	百分比	累积频数	累积百分比
.	86	0.61	86	0.61
1	13867	98.03	13953	98.64
2	193	1.36	14146	100.00

1年心血管源性死亡，0=NO; 1=YES				
death_cvd	频数	百分比	累积频数	累积百分比
0	13949	98.61	13949	98.61
1	197	1.39	14146	100.00

N12.Follow-up events at 12 months:Occurrence of combined vascular event(including cardiovascular death,non-fatal stroke,non-fatal myocardial infarction):0-No;1-Yes;				
y1_comb	频数	百分比	累积频数	累积百分比
0	12641	89.36	12641	89.36
1	1505	10.64	14146	100.00

N12.Follow-up events at 12 months: Whether the patient died: 0-survival;1-death;				
y1_death	频数	百分比	累积频数	累积百分比
0	13660	96.56	13660	96.56
1	486	3.44	14146	100.00

continuous variables, descriptive by group

MEANS PROCEDURE

1=<18.5;2=18.5-<23;3=23-<27.5;4= ≥ 27.5	观测数	变量	标签	数目	缺失值个数	均值	标准差	最小值
1	309	WAIST<br>AGE<br>A_NIHSS	F.Physical examination: Waist circumference (cm) ;<br>A.Basic Information: Age (years old);<br>F.Admitting NIHSS: Total score;	133<br>309<br>309	176<br>0<br>0	73.3984962<br>69.2588997<br>6.0711974	9.9460209<br>12.0781127<br>5.4820941	35.0000000<br>27.0000000<br>0
2	3855	WAIST<br>AGE<br>A_NIHSS	F.Physical examination: Waist circumference (cm) ;<br>A.Basic Information: Age (years old);<br>F.Admitting NIHSS: Total score;	1442<br>3855<br>3855	2413<br>0<br>0	79.5970874<br>64.3214008<br>4.8018158	12.4930767<br>11.1504173<br>4.6723076	30.0000000<br>25.0000000<br>0
3	7491	WAIST<br>AGE<br>A_NIHSS	F.Physical examination: Waist circumference (cm) ;<br>A.Basic Information: Age (years old);<br>F.Admitting NIHSS: Total score;	2420<br>7491<br>7491	5071<br>0<br>0	86.6247934<br>61.9217728<br>4.2498999	12.4240115<br>10.8662346<br>3.9231977	30.0000000<br>19.0000000<br>0
4	2491	WAIST<br>AGE<br>A_NIHSS	F.Physical examination: Waist circumference (cm) ;<br>A.Basic Information: Age (years old);<br>F.Admitting NIHSS: Total score;	810<br>2491<br>2491	1681<br>0<br>0	95.0666667<br>59.5178643<br>4.1067844	16.0004790<br>11.7739409<br>3.8228648	30.0000000<br>19.0000000<br>0

1=<18.5;2=18.5-<23;3=23-<27.5;4= ≥ 27.5	观测数	变量	标签	下四分位数	中位数	上四分位数	最大值
1	309	WAIST<br>AGE<br>A_NIHSS	F.Physical examination: Waist circumference (cm) ;<br>A.Basic Information: Age (years old);<br>F.Admitting NIHSS: Total score;	69.0000000<br>61.0000000<br>2.0000000	72.0000000<br>72.0000000<br>5.0000000	79.0000000<br>78.0000000<br>9.0000000	98.0000000<br>94.0000000<br>32.0000000
2	3855	WAIST<br>AGE<br>A_NIHSS	F.Physical examination: Waist circumference (cm) ;<br>A.Basic Information: Age (years old);<br>F.Admitting NIHSS: Total score;	75.0000000<br>57.0000000<br>2.0000000	80.0000000<br>65.0000000<br>4.0000000	86.0000000<br>73.0000000<br>6.0000000	165.0000000<br>96.0000000<br>40.0000000
3	7491	WAIST<br>AGE<br>A_NIHSS	F.Physical examination: Waist circumference (cm) ;<br>A.Basic Information: Age (years old);<br>F.Admitting NIHSS: Total score;	80.0000000<br>54.0000000<br>2.0000000	87.0000000<br>62.0000000<br>3.0000000	94.0000000<br>69.0000000<br>6.0000000	165.0000000<br>92.0000000<br>35.0000000
4	2491	WAIST<br>AGE<br>A_NIHSS	F.Physical examination: Waist circumference (cm) ;<br>A.Basic Information: Age (years old);<br>F.Admitting NIHSS: Total score;	88.0000000<br>52.0000000<br>2.0000000	96.5000000<br>60.0000000<br>3.0000000	105.0000000<br>67.0000000<br>6.0000000	170.0000000<br>89.0000000<br>36.0000000

Kruskal-Wallis Test among differenct group

NPAR1WAY 过程

变量“WAIST”的 Wilcoxon 评分（秩和）<br>按变量“BMI_g”分类					
BMI_g	数目	评分汇总	H0 之下的期望值	H0 之下的标准差	均值评分
3	2420	6176546.00	5815260.0	48049.1760	2552.29174
2	1442	2466605.50	3465126.0	44043.3286	1710.54473
4	810	2767592.00	1946430.0	35977.8305	3416.78025
1	133	135671.50	319599.0	15765.6292	1020.08647
已将平均评分用于结值。					

Kruskal-Wallis 检验		
卡方	自由度	Pr > 卡方
953.2607	3	<.0001

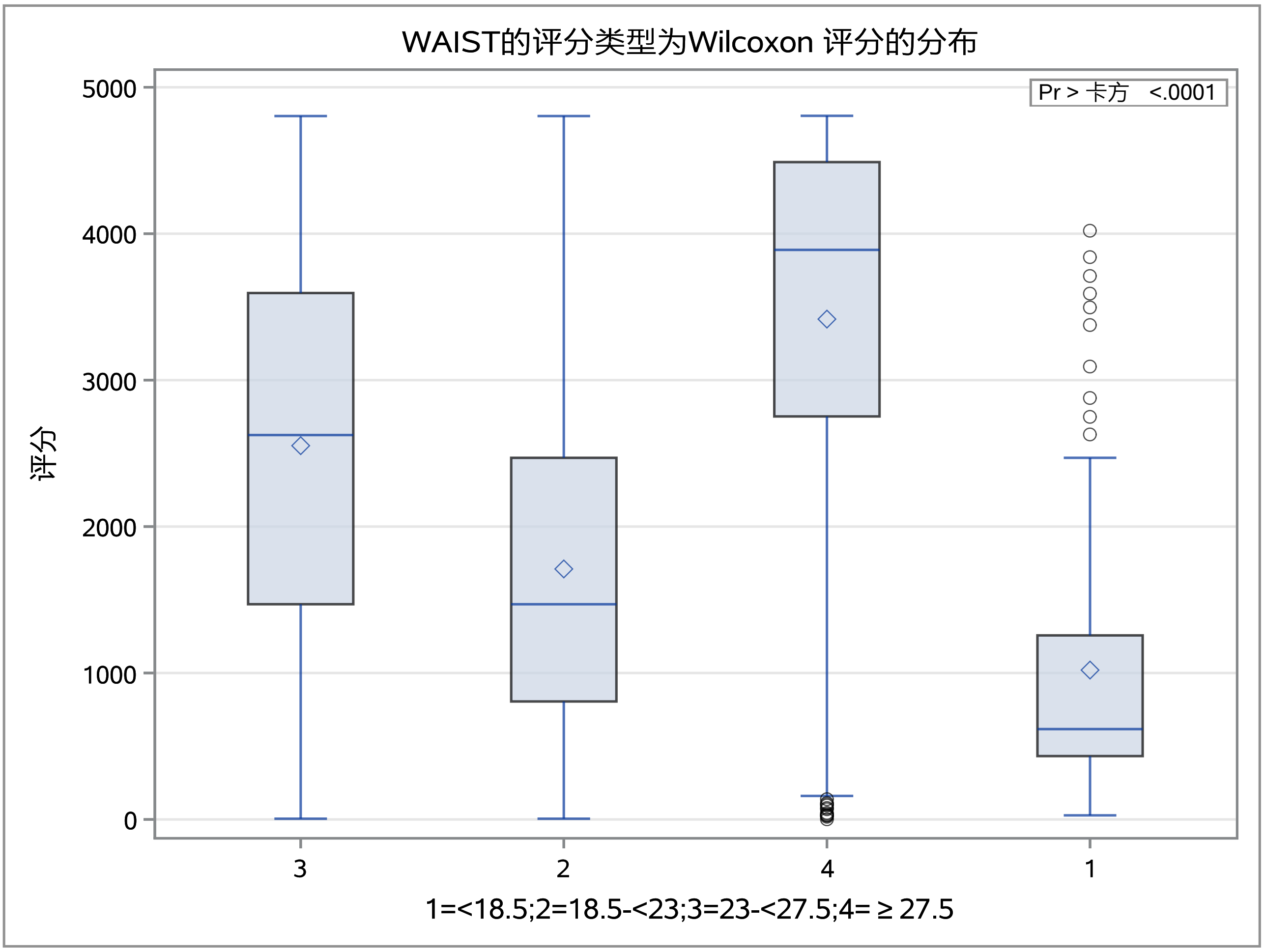

Kruskal-Wallis Test among differenct group

NPAR1WAY 过程

变量“AGE”的 Wilcoxon 评分（秩和）
按变量“BMI_g”分类

BMI_g	数目	评分汇总	H0 之下的期望值	H0 之下的标准差	均值评分
3	7491	51797336.0	52987588.5	242341.428	6914.60900
2	3855	30019592.5	27268342.5	216184.580	7787.18353
4	2491	15297882.0	17620088.5	184938.247	6141.26134
1	309	2946920.5	2185711.5	70971.493	9536.95955

已将平均评分用于结值。

Kruskal-Wallis 检验

卡方	自由度	Pr > 卡方
371.6028	3	<.0001

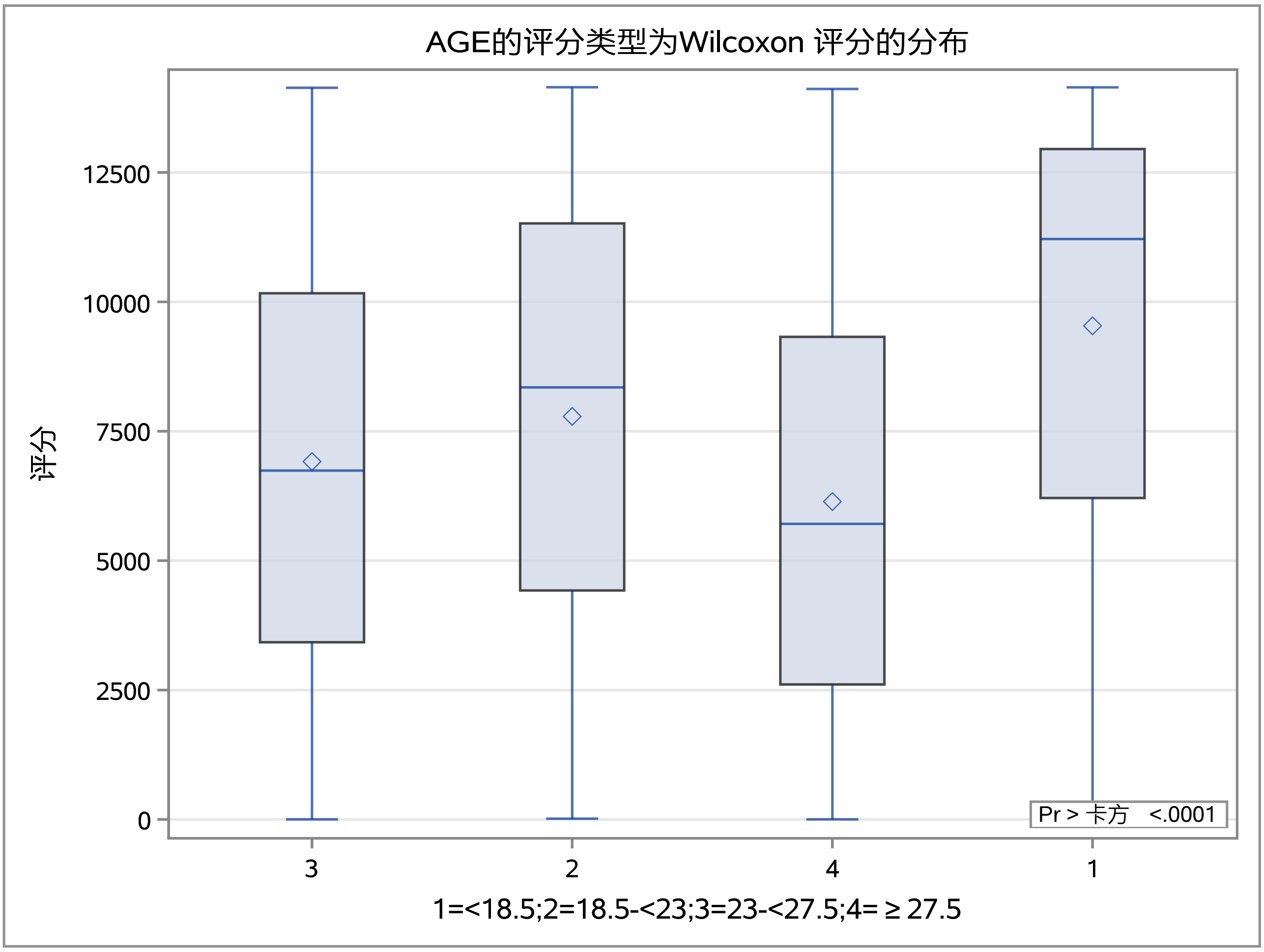

Kruskal-Wallis Test among differenct group

NPAR1WAY 过程

变量“A_NIHSS”的 Wilcoxon 评分（秩和）按变量“BMI_g”分类					
BMI_g	数目	评分汇总	H0 之下的期望值	H0 之下的标准差	均值评分
3	7491	52174221.0	52987588.5	240998.587	6964.92070
2	3855	28294607.5	27268342.5	214986.677	7339.71660
4	2491	17017090.0	17620088.5	183913.483	6831.42914
1	309	2575812.5	2185711.5	70578.232	8335.96278
已将平均评分用于结值。					

Kruskal-Wallis 检验		
卡方	自由度	Pr > 卡方
60.6759	3	<.0001

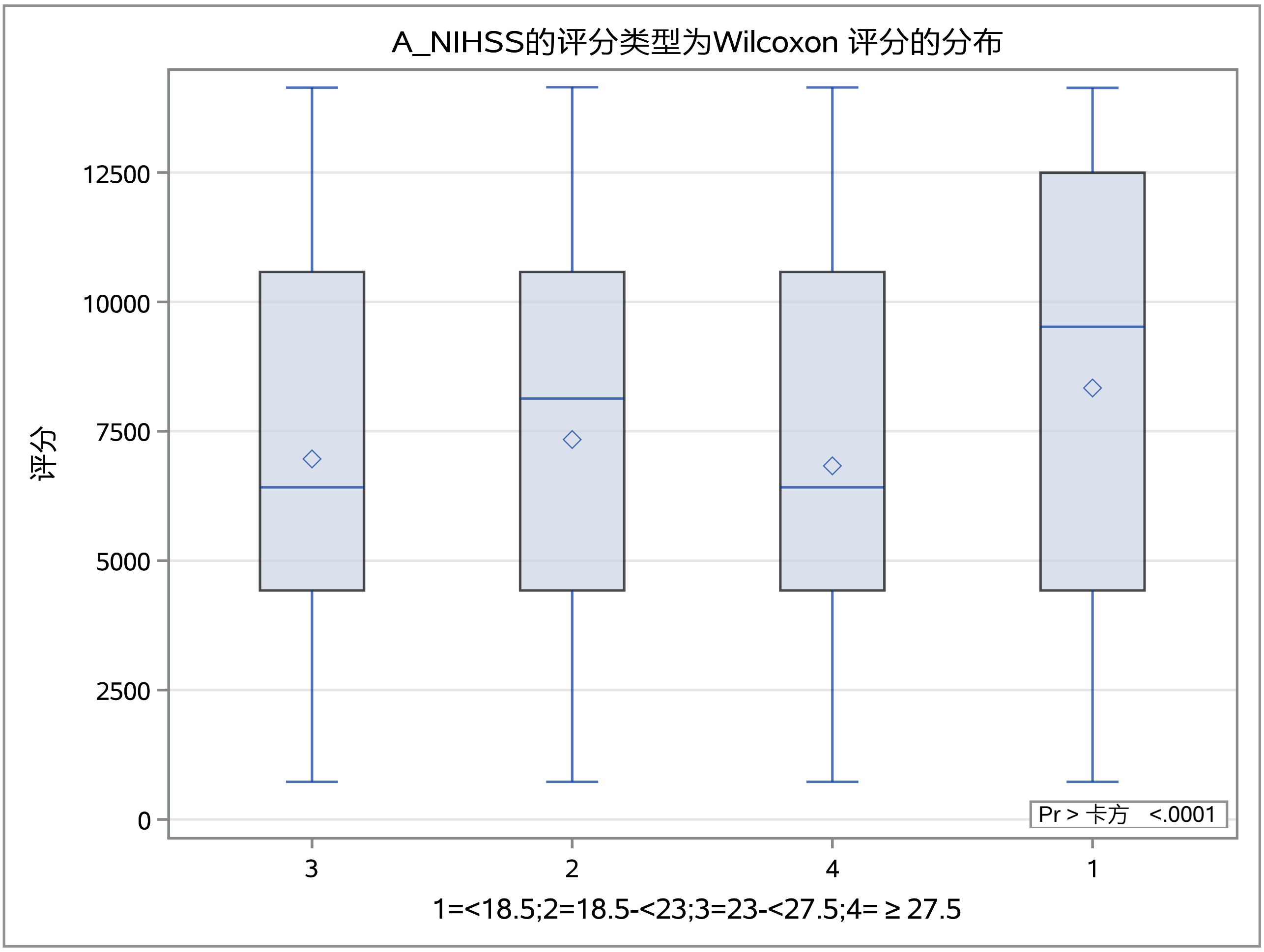

continuous variables,p for linear trend

CORR 过程

2 变量:	WAIST BMI_g

简单统计量

变量	数目	均值	标准差	中位数	最小值	最大值	标签
WAIST	4805	85.57274	14.17565	86.00000	30.00000	170.00000	F.Physical examination: Waist circumference (cm) ;
BMI_g	14146	2.85989	0.71860	3.00000	1.00000	4.00000	1=<18.5;2=18.5-<23;3=23-<27.5;4=≥27.5

Kendall Tau b 相关系数
Prob > \|tau\|, H0: Tau=0
观测数

	WAIST	BMI_g
WAIST<br>F.Physical examination: Waist circumference (cm) ;	1.00000<br><br>4805	0.35960<br><.0001<br>4805
BMI_g<br>1=<18.5;2=18.5-<23;3=23-<27.5;4=≥27.5	0.35960<br><.0001<br>4805	1.00000<br><br>14146

continuous variables,p for linear trend

CORR 过程

2 变量:	AGE BMI_g

简单统计量							
变量	数目	均值	标准差	中位数	最小值	最大值	标签
AGE	14146	62.31267	11.29549	63.00000	19.00000	96.00000	A.Basic Information: Age (years old);
BMI_g	14146	2.85989	0.71860	3.00000	1.00000	4.00000	1=<18.5;2=18.5-<23;3=23-<27.5;4=≥27.5

Kendall Tau b 相关系数, N = 14146<br>Prob > |tau|, H0: Tau=0		
	AGE	BMI_g
AGE<br>A.Basic Information: Age (years old);	1.00000	-0.12184<br><.0001
BMI_g<br>1=<18.5;2=18.5-<23;3=23-<27.5;4=≥27.5	-0.12184<br><.0001	1.00000

continuous variables,p for linear trend

CORR 过程

2 变量:	A_NIHSS BMI_g

简单统计量							
变量	数目	均值	标准差	中位数	最小值	最大值	标签
A_NIHSS	14146	4.41489	4.17815	3.00000	0	40.00000	F.Admitting NIHSS: Total score;
BMI_g	14146	2.85989	0.71860	3.00000	1.00000	4.00000	1=<18.5;2=18.5-<23;3=23-<27.5;4= ≥ 27.5

Kendall Tau b 相关系数, N = 14146<br>Prob > |tau|, H0: Tau=0		
	A_NIHSS	BMI_g
A_NIHSS<br>F.Admitting NIHSS: Total score;	1.00000	-0.04507<br><.0001
BMI_g<br>1=<18.5;2=18.5-<23;3=23-<27.5;4= ≥ 27.5	-0.04507<br><.0001	1.00000

categorical variables, descriptive by group and chisq test

FREQ 过程

频数<br>列百分比

GENDER-BMI_g表

GENDER(A.Basic Information: Gender; 1-male; 2-female;)	BMI_g(1=<18.5;2=18.5-<23;3=23-<27.5;4= ≥ 27.5)				
	1	2	3	4	合计
1	177<br>57.28	2599<br>67.42	5303<br>70.79	1641<br>65.88	9720
2	132<br>42.72	1256<br>32.58	2188<br>29.21	850<br>34.12	4426
合计	309	3855	7491	2491	14146

表“BMI_g-GENDER”的统计量

统计量	自由度	值	概率
卡方	3	46.1582	<.0001
似然比卡方检验	3	45.2037	<.0001
Mantel-Haenszel 卡方	1	1.5838	0.2082
Phi 系数		0.0571	
列联系数		0.0570	
Cramer V		0.0571	

样本大小 = 14146

频数<br>列百分比

ETHNIC-BMI_g表

ETHNIC(B.Demography: Race: 1-Han; 99-others;)	BMI_g(1=<18.5;2=18.5-<23;3=23-<27.5;4= ≥ 27.5)				
	1	2	3	4	合计
1	299<br>96.76	3776<br>97.95	7268<br>97.02	2387<br>95.82	13730
2	10<br>3.24	79<br>2.05	223<br>2.98	104<br>4.18	416
合计	309	3855	7491	2491	14146

表“BMI_g-ETHNIC”的统计量

统计量	自由度	值	概率
卡方	3	24.1577	<.0001
似然比卡方检验	3	23.9028	<.0001
Mantel-Haenszel 卡方	1	19.2091	<.0001
Phi 系数		0.0413	
列联系数		0.0413	
Cramer V		0.0413	

样本大小 = 14146

categorical variables, descriptive by group and chisq test

FREQ 过程

频数<br>列百分比					
H_STROKE01-BMI_g表					
H_STROKE01(D.History: Stroke History; 0-No; 1-Yes;)	BMI_g(1=<18.5;2=18.5-<23;3=23-<27.5;4=≥27.5)				
	1	2	3	4	合计
0	229<br>74.11	3049<br>79.09	5828<br>77.80	1906<br>76.52	11012
1	80<br>25.89	806<br>20.91	1663<br>22.20	585<br>23.48	3134
合计	309	3855	7491	2491	14146

表“BMI_g-H_STROKE01”的统计量

统计量	自由度	值	概率
卡方	3	8.5377	0.0361
似然比卡方检验	3	8.4585	0.0374
Mantel-Haenszel 卡方	1	2.6800	0.1016
Phi 系数		0.0246	
列联系数		0.0246	
Cramer V		0.0246	

样本大小 = 14146

频数<br>列百分比					
H_DIAB01-BMI_g表					
H_DIAB01(D.History: Diabetes; 0-No; 1-Yes;)	BMI_g(1=<18.5;2=18.5-<23;3=23-<27.5;4=≥27.5)				
	1	2	3	4	合计
0	268<br>86.73	3095<br>80.29	5662<br>75.58	1811<br>72.70	10836
1	41<br>13.27	760<br>19.71	1829<br>24.42	680<br>27.30	3310
合计	309	3855	7491	2491	14146

表“BMI_g-H_DIAB01”的统计量

统计量	自由度	值	概率
卡方	3	72.3406	<.0001
似然比卡方检验	3	75.0862	<.0001
Mantel-Haenszel 卡方	1	69.5503	<.0001
Phi 系数		0.0715	
列联系数		0.0713	
Cramer V		0.0715	

样本大小 = 14146

categorical variables, descriptive by group and chisq test

FREQ 过程

频数
列百分比

H_AF01-BMI_g表					
H_AF01(D.History: Heart disease category: Atrial fibrillation(Including medical history and hospitalization diagnosis); 0-No; 1-Yes;)	BMI_g(1=<18.5;2=18.5-<23;3=23-<27.5;4=≥27.5)				
	1	2	3	4	合计
0	267<br>86.41	3552<br>92.14	7002<br>93.47	2339<br>93.90	13160
1	42<br>13.59	303<br>7.86	489<br>6.53	152<br>6.10	986
合计	309	3855	7491	2491	14146

表“BMI_g-H_AF01”的统计量

统计量	自由度	值	概率
卡方	3	30.7593	<.0001
似然比卡方检验	3	26.5210	<.0001
Mantel-Haenszel 卡方	1	19.8032	<.0001
Phi 系数		0.0466	
列联系数		0.0466	
Cramer V		0.0466	

样本大小 = 14146

频数
列百分比

AI-BMI_g表					
AI(history:Myocardial infarction; 0=NO; 1=YES;)	BMI_g(1=<18.5;2=18.5-<23;3=23-<27.5;4=≥27.5)				
	1	2	3	4	合计
0	299<br>96.76	3786<br>98.21	7347<br>98.08	2436<br>97.79	13868
1	10<br>3.24	69<br>1.79	144<br>1.92	55<br>2.21	278
合计	309	3855	7491	2491	14146

表“BMI_g-AI”的统计量

统计量	自由度	值	概率
卡方	3	4.0396	0.2572
似然比卡方检验	3	3.6112	0.3066
Mantel-Haenszel 卡方	1	0.1742	0.6764
Phi 系数		0.0169	
列联系数		0.0169	
Cramer V		0.0169	

样本大小 = 14146

categorical variables, descriptive by group and chisq test

FREQ 过程

频数<br>列百分比

H_HYPT01-BMI_g表					
H_HYPT01(D.History: Hypertension; 0-No; 1-Yes;)	BMI_g(1=<18.5;2=18.5-<23;3=23-<27.5;4= ≥ 27.5)				
	1	2	3	4	合计
0	150<br>48.54	1770<br>45.91	2656<br>35.46	683<br>27.42	5259
1	159<br>51.46	2085<br>54.09	4835<br>64.54	1808<br>72.58	8887
合计	309	3855	7491	2491	14146

表“BMI_g-H_HYPT01”的统计量

统计量	自由度	值	概率
卡方	3	254.1643	<.0001
似然比卡方检验	3	255.1096	<.0001
Mantel-Haenszel 卡方	1	247.7651	<.0001
Phi 系数		0.1340	
列联系数		0.1329	
Cramer V		0.1340	

样本大小 = 14146

频数<br>列百分比

H_LIPID01-BMI_g表					
H_LIPID01(D.History: Lipid metabolism disorders; 0-No; 1-Yes;)	BMI_g(1=<18.5;2=18.5-<23;3=23-<27.5;4= ≥ 27.5)				
	1	2	3	4	合计
0	293<br>94.82	3629<br>94.14	6919<br>92.36	2230<br>89.52	13071
1	16<br>5.18	226<br>5.86	572<br>7.64	261<br>10.48	1075
合计	309	3855	7491	2491	14146

表“BMI_g-H_LIPID01”的统计量

统计量	自由度	值	概率
卡方	3	48.5466	<.0001
似然比卡方检验	3	47.2868	<.0001
Mantel-Haenszel 卡方	1	46.0074	<.0001
Phi 系数		0.0586	
列联系数		0.0585	
Cramer V		0.0586	

样本大小 = 14146

categorical variables, descriptive by group and chisq test

FREQ 过程

频数<br>列百分比	H_DRINK_H01-BMI_g表				
H_DRINK_H01(D.History: Heavy Drinking(Alcohol consumption>=20g/day); 0-No,1-Yes;)	BMI_g(1=<18.5;2=18.5-<23;3=23-<27.5;4=≥27.5)				
	1	2	3	4	合计
0	271<br>87.70	3303<br>85.68	6416<br>85.65	2146<br>86.15	12136
1	38<br>12.30	552<br>14.32	1075<br>14.35	345<br>13.85	2010
合计	309	3855	7491	2491	14146

表“BMI_g-H_DRINK_H01”的统计量

统计量	自由度	值	概率
卡方	3	1.3510	0.7171
似然比卡方检验	3	1.3897	0.7080
Mantel-Haenszel 卡方	1	0.0021	0.9632
Phi 系数		0.0098	
列联系数		0.0098	
Cramer V		0.0098	

样本大小 = 14146

频数<br>列百分比	H_SMK_C01-BMI_g表				
H_SMK_C01(D.History: Current Smoking; 0-No,1-Yes;)	BMI_g(1=<18.5;2=18.5-<23;3=23-<27.5;4=≥27.5)				
	1	2	3	4	合计
0	210<br>67.96	2645<br>68.61	5080<br>67.81	1708<br>68.57	9643
1	99<br>32.04	1210<br>31.39	2411<br>32.19	783<br>31.43	4503
合计	309	3855	7491	2491	14146

表“BMI_g-H_SMK_C01”的统计量

统计量	自由度	值	概率
卡方	3	0.9701	0.8085
似然比卡方检验	3	0.9705	0.8084
Mantel-Haenszel 卡方	1	0.0221	0.8819
Phi 系数		0.0083	
列联系数		0.0083	
Cramer V		0.0083	

样本大小 = 14146

categorical variables, descriptive by group and chisq test

FREQ 过程

频数<br>列百分比

IT-BMI_g表					
IT(intravenous thrombolysis, 1=YES,0=NO)	BMI_g(1=<18.5;2=18.5-<23;3=23-<27.5;4=≥27.5)				
	1	2	3	4	合计
0	254<br>82.20	3390<br>87.94	6740<br>89.97	2242<br>90.00	12626
1	55<br>17.80	465<br>12.06	751<br>10.03	249<br>10.00	1520
合计	309	3855	7491	2491	14146

表“BMI_g-IT”的统计量

统计量	自由度	值	概率
卡方	3	28.5105	<.0001
似然比卡方检验	3	26.0475	<.0001
Mantel-Haenszel 卡方	1	18.2370	<.0001
Phi 系数		0.0449	
列联系数		0.0448	
Cramer V		0.0449	

样本大小 = 14146

频数<br>列百分比

ET-BMI_g表					
ET(动脉溶栓或机械取栓, 1=YES,0=NO)	BMI_g(1=<18.5;2=18.5-<23;3=23-<27.5;4=≥27.5)				
	1	2	3	4	合计
0	306<br>99.03	3830<br>99.35	7459<br>99.57	2480<br>99.56	14075
1	3<br>0.97	25<br>0.65	32<br>0.43	11<br>0.44	71
合计	309	3855	7491	2491	14146

表“BMI_g-ET”的统计量

统计量	自由度	值	概率
卡方	3	4.0390	0.2573
似然比卡方检验	3	3.6573	0.3009
Mantel-Haenszel 卡方	1	2.7699	0.0960
Phi 系数		0.0169	
列联系数		0.0169	
Cramer V		0.0169	

样本大小 = 14146

categorical variables, descriptive by group and chisq test

FREQ 过程

频数
列百分比

IMG_C_TOAST-BMI_g表					
IMG_C_TOAST(K.Final diagnosis: cerebral infarction; Etiology according to TOAST system; 1-large artery atherosclerosis; 2-cardiogenic embolism; 3-small artery occlusion; 4-stroke of another determined cause; 5-stroke of an undetermined cause.)	BMI_g(1=<18.5;2=18.5-<23;3=23-<27.5;4=≥27.5)				
	1	2	3	4	合计
1	78<br>25.24	992<br>25.73	1936<br>25.84	661<br>26.54	3667
2	28<br>9.06	282<br>7.32	442<br>5.90	129<br>5.18	881
3	52<br>16.83	790<br>20.49	1723<br>23.00	572<br>22.96	3137
4	5<br>1.62	50<br>1.30	86<br>1.15	30<br>1.20	171
5	146<br>47.25	1741<br>45.16	3304<br>44.11	1099<br>44.12	6290
合计	309	3855	7491	2491	14146

表“BMI_g-IMG_C_TOAST”的统计量

统计量	自由度	值	概率
卡方	12	31.5348	0.0016
似然比卡方检验	12	31.3419	0.0017
Mantel-Haenszel 卡方	1	0.3101	0.5776
Phi 系数		0.0472	
列联系数		0.0472	
Cramer V		0.0273	

样本大小 = 14146

频数
列百分比

y1_stroke-BMI_g表					
y1_stroke(N12.Follow-up events at 12 months: Recurrence of stroke: 0-No; 1-Yes;)	BMI_g(1=<18.5;2=18.5-<23;3=23-<27.5;4=≥27.5)				
	1	2	3	4	合计
0	273<br>88.35	3473<br>90.09	6750<br>90.11	2226<br>89.36	12722
1	36<br>11.65	382<br>9.91	741<br>9.89	265<br>10.64	1424
合计	309	3855	7491	2491	14146

categorical variables, descriptive by group and chisq test

FREQ 过程

表“BMI_g-y1_stroke”的统计量

统计量	自由度	值	概率
卡方	3	2.1137	0.5491
似然比卡方检验	3	2.0638	0.5593
Mantel-Haenszel 卡方	1	0.1673	0.6826
Phi 系数		0.0122	
列联系数		0.0122	
Cramer V		0.0122	

样本大小 = 14146

频数
列百分比

y1_is-BMI_g表

y1_is(N12.Follow-up events at 12 months: recurrence of ischemic stroke: 0-No; 1-Yes;)	BMI_g(1=<18.5;2=18.5-<23;3=23-<27.5;4=≥27.5)				
	1	2	3	4	合计
0	274<br>88.67	3506<br>90.95	6804<br>90.83	2245<br>90.12	12829
1	35<br>11.33	349<br>9.05	687<br>9.17	246<br>9.88	1317
合计	309	3855	7491	2491	14146

表“BMI_g-y1_is”的统计量

统计量	自由度	值	概率
卡方	3	2.9049	0.4065
似然比卡方检验	3	2.8044	0.4228
Mantel-Haenszel 卡方	1	0.2154	0.6426
Phi 系数		0.0143	
列联系数		0.0143	
Cramer V		0.0143	

样本大小 = 14146

频数
列百分比

y1_HS-BMI_g表

y1_HS(N12.Follow-up events at 12 months: recurrence of hemorrhage stroke: 0-No; 1-Yes;)	BMI_g(1=<18.5;2=18.5-<23;3=23-<27.5;4=≥27.5)				
	1	2	3	4	合计
0	306<br>99.03	3818<br>99.04	7431<br>99.20	2467<br>99.04	14022
1	3<br>0.97	37<br>0.96	60<br>0.80	24<br>0.96	124
合计	309	3855	7491	2491	14146

categorical variables, descriptive by group and chisq test

FREQ 过程

表“BMI_g-y1_HS”的统计量

统计量	自由度	值	概率
卡方	3	1.0483	0.7896
似然比卡方检验	3	1.0459	0.7901
Mantel-Haenszel 卡方	1	0.0417	0.8382
Phi 系数		0.0086	
列联系数		0.0086	
Cramer V		0.0086	

样本大小 = 14146

频数
列百分比

I_IS_HT-BMI_g表

I_IS_HT(I.Inpatient Event: Hemorrhagic transformation after cerebral infarction; 1-No; 2-Yes; 98-UK;)	BMI_g(1=<18.5;2=18.5-<23;3=23-<27.5;4=≥27.5) 1	2	3	4	合计
1	297<br>96.43	3765<br>98.35	7348<br>98.75	2457<br>98.95	13867
2	11<br>3.57	63<br>1.65	93<br>1.25	26<br>1.05	193
合计	308	3828	7441	2483	14060
频数缺失 = 86					

表“BMI_g-I_IS_HT”的统计量

统计量	自由度	值	概率
卡方	3	15.8805	0.0012
似然比卡方检验	3	12.5988	0.0056
Mantel-Haenszel 卡方	1	10.4570	0.0012
Phi 系数		0.0336	
列联系数		0.0336	
Cramer V		0.0336	

样本大小 = 14060
频数缺失 = 86

频数
列百分比

death_cvd-BMI_g表

death_cvd(1年心血管源性死亡，0=NO; 1=YES)	BMI_g(1=<18.5;2=18.5-<23;3=23-<27.5;4=≥27.5) 1	2	3	4	合计
0	302<br>97.73	3793<br>98.39	7387<br>98.61	2467<br>99.04	13949
1	7<br>2.27	62<br>1.61	104<br>1.39	24<br>0.96	197
合计	309	3855	7491	2491	14146

categorical variables, descriptive by group and chisq test

FREQ 过程

表“BMI_g-death_cvd”的统计量

统计量	自由度	值	概率
卡方	3	6.3617	0.0953
似然比卡方检验	3	6.4307	0.0924
Mantel-Haenszel 卡方	1	5.9343	0.0148
Phi 系数		0.0212	
列联系数		0.0212	
Cramer V		0.0212	

样本大小 = 14146

频数
列百分比

y1_comb-BMI_g表					
y1_comb(N12.Follow-up events at 12 months:Occurrence of combined vascular event(including cardiovascular death,non-fatal stroke,non-fatal myocardial infarction):0-No;1-Yes;)	BMI_g(1=<18.5;2=18.5-<23;3=23-<27.5;4= ≥ 27.5)				
	1	2	3	4	合计
0	270<br>87.38	3448<br>89.44	6707<br>89.53	2216<br>88.96	12641
1	39<br>12.62	407<br>10.56	784<br>10.47	275<br>11.04	1505
合计	309	3855	7491	2491	14146

表“BMI_g-y1_comb”的统计量

统计量	自由度	值	概率
卡方	3	1.9609	0.5806
似然比卡方检验	3	1.8936	0.5948
Mantel-Haenszel 卡方	1	0.0011	0.9738
Phi 系数		0.0118	
列联系数		0.0118	
Cramer V		0.0118	

样本大小 = 14146

频数
列百分比

y1_death-BMI_g表					
y1_death(N12.Follow-up events at 12 months: Whether the patient died: 0-survival;1-death;)	BMI_g(1=<18.5;2=18.5-<23;3=23-<27.5;4= ≥ 27.5)				
	1	2	3	4	合计
0	277<br>89.64	3693<br>95.80	7256<br>96.86	2434<br>97.71	13660
1	32<br>10.36	162<br>4.20	235<br>3.14	57<br>2.29	486
合计	309	3855	7491	2491	14146

categorical variables, descriptive by group and chisq test

FREQ 过程

表“BMI_g-y1_death”的统计量

统计量	自由度	值	概率
卡方	3	63.3343	<.0001
似然比卡方检验	3	49.0477	<.0001
Mantel-Haenszel 卡方	1	42.0154	<.0001
Phi 系数		0.0669	
列联系数		0.0668	
Cramer V		0.0669	

样本大小 = 14146

categorical variables, descriptive by group and P for linear trend

FREQ 过程

频数<br>列百分比

GENDER-BMI_g表					
GENDER(A.Basic Information: Gender; 1-male; 2-female;)	BMI_g(1=<18.5;2=18.5-<23;3=23-<27.5;4= ≥ 27.5)				
	1	2	3	4	合计
1	177<br>57.28	2599<br>67.42	5303<br>70.79	1641<br>65.88	9720
2	132<br>42.72	1256<br>32.58	2188<br>29.21	850<br>34.12	4426
合计	309	3855	7491	2491	14146

表“BMI_g-GENDER”的统计量

<table>
<tr><th colspan="2">Cochran-Armitage
趋势检验</th></tr>
<tr><td>统计量 (Z)</td><td>1.2585</td></tr>
<tr><td>单侧 Pr > Z</td><td>0.1041</td></tr>
<tr><td>双侧 Pr > |Z|</td><td>0.2082</td></tr>
</table>

样本大小 = 14146

频数<br>列百分比

ETHNIC-BMI_g表					
ETHNIC(B.Demography: Race: 1-Han; 99-others;)	BMI_g(1=<18.5;2=18.5-<23;3=23-<27.5;4= ≥ 27.5)				
	1	2	3	4	合计
1	299<br>96.76	3776<br>97.95	7268<br>97.02	2387<br>95.82	13730
2	10<br>3.24	79<br>2.05	223<br>2.98	104<br>4.18	416
合计	309	3855	7491	2491	14146

表“BMI_g-ETHNIC”的统计量

<table>
<tr><th colspan="2">Cochran-Armitage
趋势检验</th></tr>
<tr><td>统计量 (Z)</td><td>-4.3830</td></tr>
<tr><td>单侧 Pr < Z</td><td><.0001</td></tr>
<tr><td>双侧 Pr > |Z|</td><td><.0001</td></tr>
</table>

样本大小 = 14146

频数<br>列百分比

H_STROKE01-BMI_g表					
H_STROKE01(D.History: Stroke History; 0-No; 1-Yes;)	BMI_g(1=<18.5;2=18.5-<23;3=23-<27.5;4= ≥ 27.5)				
	1	2	3	4	合计
0	229<br>74.11	3049<br>79.09	5828<br>77.80	1906<br>76.52	11012
1	80<br>25.89	806<br>20.91	1663<br>22.20	585<br>23.48	3134
合计	309	3855	7491	2491	14146

categorical variables, descriptive by group and P for linear trend

FREQ 过程

表“BMI_g-H_STROKE01”的统计量

Cochran-Armitage 趋势检验	
统计量 (Z)	-1.6371
单侧 Pr < Z	0.0508
双侧 Pr > |Z|	0.1016

样本大小 = 14146

频数
列百分比

H_DIAB01-BMI_g表					
H_DIAB01(D.History: Diabetes; 0-No; 1-Yes;)	BMI_g(1=<18.5;2=18.5-<23;3=23-<27.5;4=≥27.5)				
	1	2	3	4	合计
0	268<br>86.73	3095<br>80.29	5662<br>75.58	1811<br>72.70	10836
1	41<br>13.27	760<br>19.71	1829<br>24.42	680<br>27.30	3310
合计	309	3855	7491	2491	14146

表“BMI_g-H_DIAB01”的统计量

Cochran-Armitage 趋势检验	
统计量 (Z)	-8.3400
单侧 Pr < Z	<.0001
双侧 Pr > |Z|	<.0001

样本大小 = 14146

频数
列百分比

H_AF01-BMI_g表					
H_AF01(D.History: Heart disease category: Atrial fibrillation(Including medical history and hospitalization diagnosis); 0-No; 1-Yes;)	BMI_g(1=<18.5;2=18.5-<23;3=23-<27.5;4=≥27.5)				
	1	2	3	4	合计
0	267<br>86.41	3552<br>92.14	7002<br>93.47	2339<br>93.90	13160
1	42<br>13.59	303<br>7.86	489<br>6.53	152<br>6.10	986
合计	309	3855	7491	2491	14146

表“BMI_g-H_AF01”的统计量

Cochran-Armitage 趋势检验	
统计量 (Z)	4.4502
单侧 Pr > Z	<.0001
双侧 Pr > |Z|	<.0001

样本大小 = 14146

categorical variables, descriptive by group and P for linear trend

FREQ 过程

频数
列百分比

AI-BMI_g表

AI(history:Myocardial infarction; 0=NO; 1=YES;)	BMI_g(1=<18.5;2=18.5-<23;3=23-<27.5;4=≥27.5)				
	1	2	3	4	合计
0	299<br>96.76	3786<br>98.21	7347<br>98.08	2436<br>97.79	13868
1	10<br>3.24	69<br>1.79	144<br>1.92	55<br>2.21	278
合计	309	3855	7491	2491	14146

表“BMI_g-AI”的统计量

Cochran-Armitage<br>趋势检验	
统计量 (Z)	-0.4173
单侧 Pr < Z	0.3382
双侧 Pr > |Z|	0.6764

样本大小 = 14146

频数
列百分比

H_HYPT01-BMI_g表

H_HYPT01(D.History: Hypertension; 0-No; 1-Yes;)	BMI_g(1=<18.5;2=18.5-<23;3=23-<27.5;4=≥27.5)				
	1	2	3	4	合计
0	150<br>48.54	1770<br>45.91	2656<br>35.46	683<br>27.42	5259
1	159<br>51.46	2085<br>54.09	4835<br>64.54	1808<br>72.58	8887
合计	309	3855	7491	2491	14146

表“BMI_g-H_HYPT01”的统计量

Cochran-Armitage<br>趋势检验	
统计量 (Z)	-15.7411
单侧 Pr < Z	<.0001
双侧 Pr > |Z|	<.0001

样本大小 = 14146

频数
列百分比

H_LIPID01-BMI_g表

H_LIPID01(D.History: Lipid metabolism disorders; 0-No; 1-Yes;)	BMI_g(1=<18.5;2=18.5-<23;3=23-<27.5;4=≥27.5)				
	1	2	3	4	合计
0	293<br>94.82	3629<br>94.14	6919<br>92.36	2230<br>89.52	13071
1	16<br>5.18	226<br>5.86	572<br>7.64	261<br>10.48	1075
合计	309	3855	7491	2491	14146

categorical variables, descriptive by group and P for linear trend

FREQ 过程

表“BMI_g-H_LIPID01”的统计量

Cochran-Armitage 趋势检验	
统计量 (Z)	-6.7831
单侧 Pr < Z	<.0001
双侧 Pr > |Z|	<.0001

样本大小 = 14146

频数
列百分比

H_DRINK_H01-BMI_g表					
H_DRINK_H01(D.History: Heavy Drinking(Alcohol consumption>=20g/day); 0-No,1-Yes;)	BMI_g(1=<18.5;2=18.5-<23;3=23-<27.5;4= ≥ 27.5)				
	1	2	3	4	合计
0	271<br>87.70	3303<br>85.68	6416<br>85.65	2146<br>86.15	12136
1	38<br>12.30	552<br>14.32	1075<br>14.35	345<br>13.85	2010
合计	309	3855	7491	2491	14146

表“BMI_g-H_DRINK_H01”的统计量

Cochran-Armitage 趋势检验	
统计量 (Z)	0.0462
单侧 Pr > Z	0.4816
双侧 Pr > |Z|	0.9632

样本大小 = 14146

频数
列百分比

H_SMK_C01-BMI_g表					
H_SMK_C01(D.History: Current Smoking; 0-No,1-Yes;)	BMI_g(1=<18.5;2=18.5-<23;3=23-<27.5;4= ≥ 27.5)				
	1	2	3	4	合计
0	210<br>67.96	2645<br>68.61	5080<br>67.81	1708<br>68.57	9643
1	99<br>32.04	1210<br>31.39	2411<br>32.19	783<br>31.43	4503
合计	309	3855	7491	2491	14146

表“BMI_g-H_SMK_C01”的统计量

Cochran-Armitage 趋势检验	
统计量 (Z)	-0.1486
单侧 Pr < Z	0.4409
双侧 Pr > |Z|	0.8819

样本大小 = 14146

categorical variables, descriptive by group and P for linear trend

FREQ 过程

频数
列百分比

IT-BMI_g表

IT(intravenous thrombolysis, 1=YES,0=NO)	BMI_g(1=<18.5;2=18.5-<23;3=23-<27.5;4=≥27.5) 1	2	3	4	合计
0	254<br>82.20	3390<br>87.94	6740<br>89.97	2242<br>90.00	12626
1	55<br>17.80	465<br>12.06	751<br>10.03	249<br>10.00	1520
合计	309	3855	7491	2491	14146

表“BMI_g-IT”的统计量

Cochran-Armitage 趋势检验	
统计量 (Z)	4.2706
单侧 Pr > Z	<.0001
双侧 Pr > |Z|	<.0001

样本大小 = 14146

频数
列百分比

ET-BMI_g表

ET(动脉溶栓或机械取栓, 1=YES,0=NO)	BMI_g(1=<18.5;2=18.5-<23;3=23-<27.5;4=≥27.5) 1	2	3	4	合计
0	306<br>99.03	3830<br>99.35	7459<br>99.57	2480<br>99.56	14075
1	3<br>0.97	25<br>0.65	32<br>0.43	11<br>0.44	71
合计	309	3855	7491	2491	14146

表“BMI_g-ET”的统计量

Cochran-Armitage 趋势检验	
统计量 (Z)	1.6644
单侧 Pr > Z	0.0480
双侧 Pr > |Z|	0.0960

样本大小 = 14146

categorical variables, descriptive by group and P for linear trend

FREQ 过程

频数<br>列百分比

IMG_C_TOAST-BMI_g表

IMG_C_TOAST(K.Final diagnosis: cerebral infarction; Etiology according to TOAST system; 1-large artery atherosclerosis; 2-cardiogenic embolism; 3-small artery occlusion; 4-stroke of another determined cause; 5-stroke of an undetermined cause.)	BMI_g(1=<18.5;2=18.5-<23;3=23-<27.5;4=≥27.5)				
	1	2	3	4	合计
1	78<br>25.24	992<br>25.73	1936<br>25.84	661<br>26.54	3667
2	28<br>9.06	282<br>7.32	442<br>5.90	129<br>5.18	881
3	52<br>16.83	790<br>20.49	1723<br>23.00	572<br>22.96	3137
4	5<br>1.62	50<br>1.30	86<br>1.15	30<br>1.20	171
5	146<br>47.25	1741<br>45.16	3304<br>44.11	1099<br>44.12	6290
合计	309	3855	7491	2491	14146

频数<br>列百分比

y1_stroke-BMI_g表

y1_stroke(N12.Follow-up events at 12 months: Recurrence of stroke: 0-No; 1-Yes;)	BMI_g(1=<18.5;2=18.5-<23;3=23-<27.5;4=≥27.5)				
	1	2	3	4	合计
0	273<br>88.35	3473<br>90.09	6750<br>90.11	2226<br>89.36	12722
1	36<br>11.65	382<br>9.91	741<br>9.89	265<br>10.64	1424
合计	309	3855	7491	2491	14146

表“BMI_g-y1_stroke”的统计量

Cochran-Armitage 趋势检验	
统计量 (Z)	-0.4090
单侧 Pr < Z	0.3413
双侧 Pr > |Z|	0.6826

样本大小 = 14146

categorical variables, descriptive by group and P for linear trend

FREQ 过程

频数
列百分比

y1_is-BMI_g表					
y1_is(N12.Follow-up events at 12 months: recurrence of ischemic stroke: 0-No; 1-Yes;)	BMI_g(1=<18.5;2=18.5-<23;3=23-<27.5;4=≥27.5)				
	1	2	3	4	合计
0	274<br>88.67	3506<br>90.95	6804<br>90.83	2245<br>90.12	12829
1	35<br>11.33	349<br>9.05	687<br>9.17	246<br>9.88	1317
合计	309	3855	7491	2491	14146

表“BMI_g-y1_is”的统计量

Cochran-Armitage 趋势检验	
统计量 (Z)	-0.4641
单侧 Pr < Z	0.3213
双侧 Pr > |Z|	0.6426

样本大小 = 14146

频数
列百分比

y1_HS-BMI_g表					
y1_HS(N12.Follow-up events at 12 months: recurrence of hemorrhage stroke: 0-No; 1-Yes;)	BMI_g(1=<18.5;2=18.5-<23;3=23-<27.5;4=≥27.5)				
	1	2	3	4	合计
0	306<br>99.03	3818<br>99.04	7431<br>99.20	2467<br>99.04	14022
1	3<br>0.97	37<br>0.96	60<br>0.80	24<br>0.96	124
合计	309	3855	7491	2491	14146

表“BMI_g-y1_HS”的统计量

Cochran-Armitage 趋势检验	
统计量 (Z)	0.2041
单侧 Pr > Z	0.4191
双侧 Pr > |Z|	0.8382

样本大小 = 14146

categorical variables, descriptive by group and P for linear trend

FREQ 过程

频数<br>列百分比					
**I_IS_HT-BMI_g表**					
I_IS_HT(I.Inpatient Event: Hemorrhagic transformation after cerebral infarction; 1-No; 2-Yes; 98-UK;)	BMI_g(1=<18.5;2=18.5-<23;3=23-<27.5;4=≥27.5)				
	1	2	3	4	合计
1	297<br>96.43	3765<br>98.35	7348<br>98.75	2457<br>98.95	13867
2	11<br>3.57	63<br>1.65	93<br>1.25	26<br>1.05	193
合计	308	3828	7441	2483	14060
频数缺失 = 86					

表“BMI_g-I_IS_HT”的统计量

Cochran-Armitage 趋势检验	
统计量 (Z)	3.2338
单侧 Pr > Z	0.0006
双侧 Pr > |Z|	0.0012

样本大小 = 14060
频数缺失 = 86

频数<br>列百分比					
**death_cvd-BMI_g表**					
death_cvd(1年心血管源性死亡，0=NO; 1=YES)	BMI_g(1=<18.5;2=18.5-<23;3=23-<27.5;4=≥27.5)				
	1	2	3	4	合计
0	302<br>97.73	3793<br>98.39	7387<br>98.61	2467<br>99.04	13949
1	7<br>2.27	62<br>1.61	104<br>1.39	24<br>0.96	197
合计	309	3855	7491	2491	14146

表“BMI_g-death_cvd”的统计量

Cochran-Armitage 趋势检验	
统计量 (Z)	2.4361
单侧 Pr > Z	0.0074
双侧 Pr > |Z|	0.0148

样本大小 = 14146

categorical variables, descriptive by group and P for linear trend

FREQ 过程

频数<br>列百分比

y1_comb-BMI_g表					
y1_comb(N12.Follow-up events at 12 months:Occurrence of combined vascular event(including cardiovascular death,non-fatal stroke,non-fatal myocardial infarction):0-No;1-Yes;)	BMI_g(1=<18.5;2=18.5-<23;3=23-<27.5;4= ≥ 27.5)				
	1	2	3	4	合计
0	270<br>87.38	3448<br>89.44	6707<br>89.53	2216<br>88.96	12641
1	39<br>12.62	407<br>10.56	784<br>10.47	275<br>11.04	1505
合计	309	3855	7491	2491	14146

表“BMI_g-y1_comb”的统计量

Cochran-Armitage<br>趋势检验	
统计量 (Z)	-0.0329
单侧 Pr < Z	0.4869
双侧 Pr > |Z|	0.9738

样本大小 = 14146

频数<br>列百分比

y1_death-BMI_g表					
y1_death(N12.Follow-up events at 12 months: Whether the patient died: 0-survival;1-death;)	BMI_g(1=<18.5;2=18.5-<23;3=23-<27.5;4= ≥ 27.5)				
	1	2	3	4	合计
0	277<br>89.64	3693<br>95.80	7256<br>96.86	2434<br>97.71	13660
1	32<br>10.36	162<br>4.20	235<br>3.14	57<br>2.29	486
合计	309	3855	7491	2491	14146

表“BMI_g-y1_death”的统计量

Cochran-Armitage<br>趋势检验	
统计量 (Z)	6.4822
单侧 Pr > Z	<.0001
双侧 Pr > |Z|	<.0001

样本大小 = 14146
