# Supplementary material for: Association of body mass index and waist-to-height ratio with outcomes in ischemic stroke: results from the Third China National Stroke Registry
Source: BMC Neurol. 2023 Apr 14;23:152. doi: 10.1186/s12883-023-03165-y (PMC10103413; doi:10.1186/s12883-023-03165-y)
Supplement: Supplementary file 2 — Additional file 2. [file 12883_2023_3165_MOESM2_ESM.zip › raw data/Table-s5.pdf]

FREQ 过程

N12.Follow-up events at 12 months: Whether the patient died: 0-survival;1-death;		
y1_death	频数	累积频数
0	13660	13660
1	486	14146

等比例的卡方检验	
卡方	12268.7881
自由度	1
Pr > 卡方	<.0001

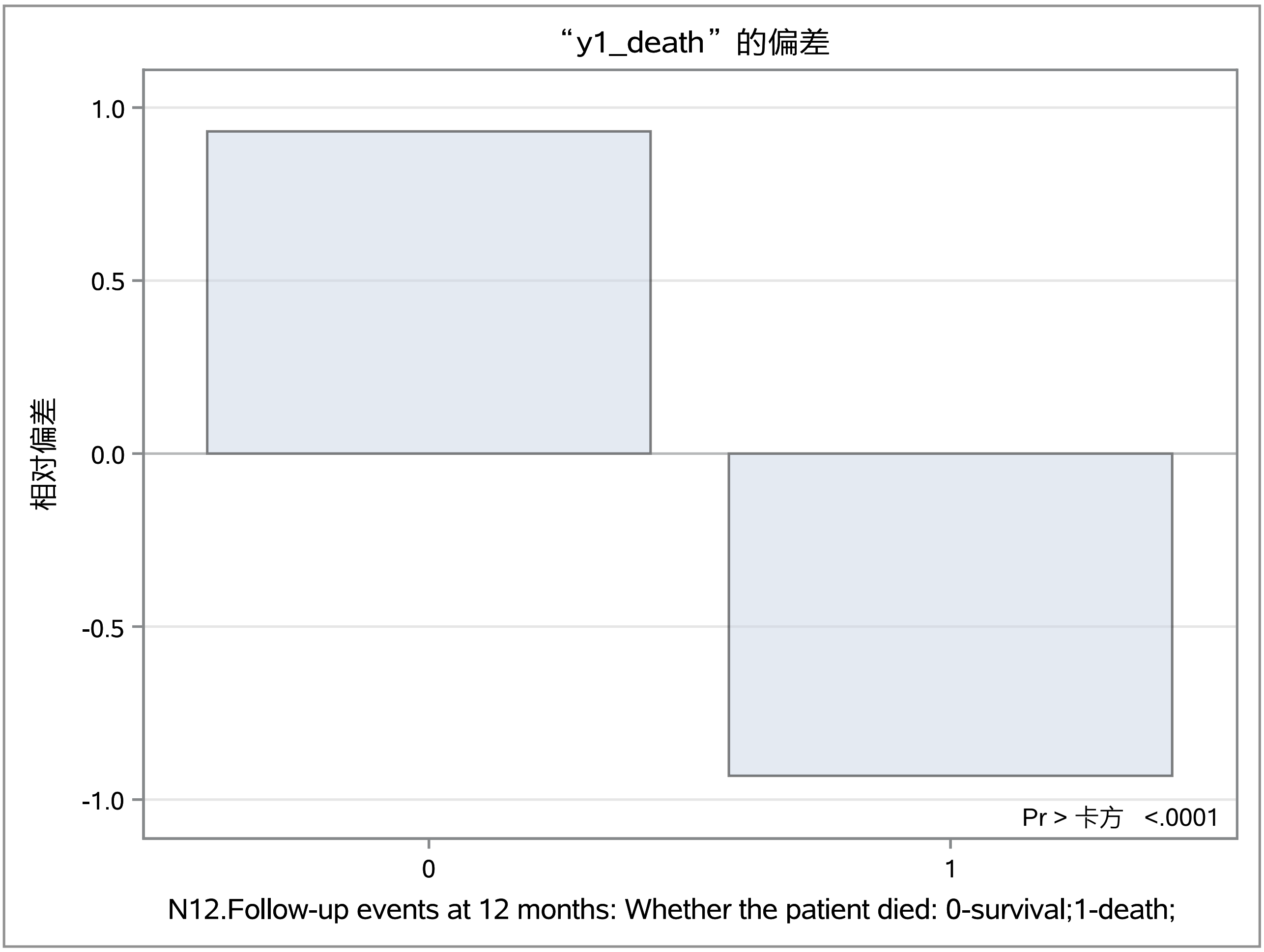

样本大小 = 14146

PHREG 过程

模型信息		
数据集	WORK.DATA2	
因变量	y1_death_dd	N12.Follow-up events at 12 months: Days from onset to death;(day);
删失变量	y1_death	N12.Follow-up events at 12 months: Whether the patient died: 0-survival;1-death;
删失值	0	
结值处理	BRESLOW	

读取的观测数	14146
使用的观测数	14146

事件和删失值个数汇总			
合计	事件	删失	删失百分比
14146	486	13660	96.56

收敛状态
满足收敛准则 (GCONV=1E-8)。

模型拟合统计量		
准则	无协变量	带协变量
-2 LOG L	9255.945	9214.018
AIC	9255.945	9216.018
SBC	9255.945	9220.204

检验全局原假设: BETA=0			
检验	卡方	自由度	Pr > 卡方
似然比	41.9270	1	<.0001
评分	39.2155	1	<.0001
Wald	40.2417	1	<.0001

最大似然估计分析									
参数	自由度	参数估计	标准误差	卡方	Pr > 卡方	危险率	95% 危险率置信限		标签
BMI	1	-0.09403	0.01482	40.2417	<.0001	0.910	0.884	0.937	F.Physical examination: Body mass index (kg/m2);

PHREG 过程

模型信息		
数据集	WORK.DATA2	
因变量	y1_death_dd	N12.Follow-up events at 12 months: Days from onset to death;(day);
删失变量	y1_death	N12.Follow-up events at 12 months: Whether the patient died: 0-survival;1-death;
删失值	0	
结值处理	BRESLOW	

读取的观测数<br>使用的观测数	14146<br>14146

分类水平信息					
分类	值	设计变量			
GENDER	2	1			
	1	0			
ETHNIC	2	1			
	1	0			
H_DIAB01	1	1			
	0	0			
H_AF01	1	1			
	0	0			
H_HYPT01	1	1			
	0	0			
H_LIPID01	1	1			
	0	0			
AI	1	1			
	0	0			
H_DRINK_H01	1	1			
	0	0			
H_SMK_C01	1	1			
	0	0			
IT	1	1			
	0	0			
ET	1	1			
	0	0			
IMG_C_TOAST	5	1	0	0	0
	4	0	1	0	0
	3	0	0	1	0
	2	0	0	0	1
	1	0	0	0	0

PHREG 过程

事件和删失值个数汇总			
合计	事件	删失	删失<br>百分比
14146	486	13660	96.56

收敛状态
满足收敛准则 (GCONV=1E-8)。

模型拟合统计量		
准则	无<br>协变量	带<br>协变量
-2 LOG L	9255.945	8612.779
AIC	9255.945	8648.779
SBC	9255.945	8724.130

检验全局原假设: BETA=0			
检验	卡方	自由度	Pr > 卡方
似然比	643.1662	18	<.0001
评分	864.4894	18	<.0001
Wald	753.3524	18	<.0001

3 型检验			
效应	自由度	Wald 卡方	Pr > 卡方
BMI	1	7.6342	0.0057
AGE	1	151.3622	<.0001
GENDER	1	1.4715	0.2251
ETHNIC	1	3.7080	0.0542
H_DIAB01	1	12.2288	0.0005
H_AF01	1	29.5276	<.0001
H_HYPT01	1	0.2046	0.6511
H_LIPID01	1	2.1541	0.1422
AI	1	5.2230	0.0223
H_DRINK_H01	1	6.9709	0.0083
H_SMK_C01	1	0.9672	0.3254
IT	1	19.2782	<.0001
ET	1	7.3996	0.0065
IMG_C_TOAST	4	35.0394	<.0001
A_NIHSS	1	228.5723	<.0001

PHREG 过程

最大似然估计分析									
参数		自由度	参数估计	标准误差	卡方	Pr > 卡方	危险率	95% 危险率置信限	
BMI		1	-0.03892	0.01409	7.6342	0.0057	0.962	0.936	0.989
AGE		1	0.05916	0.00481	151.3622	<.0001	1.061	1.051	1.071
GENDER	2	1	-0.12412	0.10232	1.4715	0.2251	0.883	0.723	1.079
ETHNIC	2	1	0.45284	0.23516	3.7080	0.0542	1.573	0.992	2.494
H_DIAB01	1	1	0.36395	0.10408	12.2288	0.0005	1.439	1.173	1.765
H_AF01	1	1	0.81245	0.14951	29.5276	<.0001	2.253	1.681	3.021
H_HYPT01	1	1	0.04425	0.09783	0.2046	0.6511	1.045	0.863	1.266
H_LIPID01	1	1	-0.30464	0.20757	2.1541	0.1422	0.737	0.491	1.108
AI	1	1	0.51703	0.22623	5.2230	0.0223	1.677	1.076	2.613
H_DRINK_H01	1	1	-0.51695	0.19580	6.9709	0.0083	0.596	0.406	0.875
H_SMK_C01	1	1	0.12210	0.12416	0.9672	0.3254	1.130	0.886	1.441
IT	1	1	-0.72950	0.16615	19.2782	<.0001	0.482	0.348	0.668
ET	1	1	0.85199	0.31321	7.3996	0.0065	2.344	1.269	4.331
IMG_C_TOAST	5	1	-0.23261	0.11130	4.3679	0.0366	0.792	0.637	0.986
IMG_C_TOAST	4	1	0.65387	0.32719	3.9938	0.0457	1.923	1.013	3.652
IMG_C_TOAST	3	1	-0.93227	0.18256	26.0775	<.0001	0.394	0.275	0.563
IMG_C_TOAST	2	1	-0.42895	0.19355	4.9118	0.0267	0.651	0.446	0.952
A_NIHSS		1	0.09488	0.00628	228.5723	<.0001	1.100	1.086	1.113

PHREG 过程

最大似然估计分析		
参数		标签
BMI		F.Physical examination: Body mass index (kg/m2);
AGE		A.Basic Information: Age (years old);
GENDER	2	A.Basic Information: Gender; 1-male; 2-female; 2
ETHNIC	2	B.Demography: Race: 1-Han; 99-others; 2
H_DIAB01	1	D.History: Diabetes; 0-No; 1-Yes; 1
H_AF01	1	D.History: Heart disease category: Atrial fibrillation(Including medical history and hospitalization diagnosis); 0-No; 1-Yes; 1
H_HYPT01	1	D.History: Hypertension; 0-No; 1-Yes; 1
H_LIPID01	1	D.History: Lipid metabolism disorders; 0-No; 1-Yes; 1
AI	1	history:Myocardial infarction; 0=NO; 1=YES; 1
H_DRINK_H01	1	D.History: Heavy Drinking(Alcohol consumption>=20g/day); 0-No,1-Yes; 1
H_SMK_C01	1	D.History: Current Smoking; 0-No,1-Yes; 1
IT	1	intravenous thrombolysis, 1=YES,0=NO 1
ET	1	动脉溶栓或机械取栓, 1=YES,0=NO 1
IMG_C_TOAST	5	K.Final diagnosis: cerebral infarction; Etiology according to TOAST system; 1-large artery atherosclerosis; 2-cardiogenic embolism; 3-small artery occlusion; 4-stroke of another determined cause; 5-stroke of an undetermined cause. 5
IMG_C_TOAST	4	K.Final diagnosis: cerebral infarction; Etiology according to TOAST system; 1-large artery atherosclerosis; 2-cardiogenic embolism; 3-small artery occlusion; 4-stroke of another determined cause; 5-stroke of an undetermined cause. 4
IMG_C_TOAST	3	K.Final diagnosis: cerebral infarction; Etiology according to TOAST system; 1-large artery atherosclerosis; 2-cardiogenic embolism; 3-small artery occlusion; 4-stroke of another determined cause; 5-stroke of an undetermined cause. 3
IMG_C_TOAST	2	K.Final diagnosis: cerebral infarction; Etiology according to TOAST system; 1-large artery atherosclerosis; 2-cardiogenic embolism; 3-small artery occlusion; 4-stroke of another determined cause; 5-stroke of an undetermined cause. 2
A_NIHSS		F.Admitting NIHSS: Total score;

BMI with y1_death: interaction with stroke subtype

PHREG 过程

模型信息		
数据集	WORK.DATA2	
因变量	y1_death_dd	N12.Follow-up events at 12 months: Days from onset to death;(day);
删失变量	y1_death	N12.Follow-up events at 12 months: Whether the patient died: 0-survival;1-death;
删失值	0	
结值处理	BRESLOW	

读取的观测数<br>使用的观测数	14146<br>14146

分类水平信息					
分类	值	设计变量			
GENDER	2	1			
	1	0			
ETHNIC	2	1			
	1	0			
H_DIAB01	1	1			
	0	0			
H_AF01	1	1			
	0	0			
H_HYPT01	1	1			
	0	0			
H_LIPID01	1	1			
	0	0			
AI	1	1			
	0	0			
H_DRINK_H01	1	1			
	0	0			
H_SMK_C01	1	1			
	0	0			
IT	1	1			
	0	0			
ET	1	1			
	0	0			
IMG_C_TOAST	5	1	0	0	0
	4	0	1	0	0
	3	0	0	1	0
	2	0	0	0	1
	1	0	0	0	0

BMI with y1_death: interaction with stroke subtype

PHREG 过程

事件和删失值个数汇总			
合计	事件	删失	删失百分比
14146	486	13660	96.56

收敛状态
满足收敛准则 (GCONV=1E-8)。

模型拟合统计量		
准则	无协变量	带协变量
-2 LOG L	9255.945	8604.849
AIC	9255.945	8648.849
SBC	9255.945	8740.946

检验全局原假设: BETA=0			
检验	卡方	自由度	Pr > 卡方
似然比	651.0956	22	<.0001
评分	882.2772	22	<.0001
Wald	771.3525	22	<.0001

联合检验			
效应	自由度	Wald 卡方	Pr > 卡方
BMI	1	0.0006	0.9803
IMG_C_TOAST	4	9.6019	0.0477
BMI*IMG_C_TOAST	4	7.7130	0.1027
AGE	1	149.7490	<.0001
GENDER	1	1.4925	0.2218
ETHNIC	1	3.3670	0.0665
H_DIAB01	1	12.1795	0.0005
H_AF01	1	29.0943	<.0001
H_HYPT01	1	0.2355	0.6275
H_LIPID01	1	2.3037	0.1291
AI	1	5.3766	0.0204
H_DRINK_H01	1	7.0698	0.0078
H_SMK_C01	1	1.0994	0.2944
IT	1	19.1919	<.0001
ET	1	7.8276	0.0051
A_NIHSS	1	225.6003	<.0001

Note: Under full-rank parameterizations, Type 3 effect tests are replaced by joint tests. The joint test for an effect is a test that all of the parameters associated with that effect are zero. Such joint tests might not be equivalent to Type 3 effect tests under GLM parameterization.

BMI with y1_death: interaction with stroke subtype

PHREG 过程

最大似然估计分析

参数		自由度	参数估计	标准误差	卡方	Pr > 卡方	危险率	95% 危险率置信限	
BMI		1	-0.0006048	0.02449	0.0006	0.9803	.	.	.
IMG_C_TOAST	5	1	1.36937	0.76936	3.1680	0.0751	.	.	.
IMG_C_TOAST	4	1	5.00261	2.46480	4.1194	0.0424	.	.	.
IMG_C_TOAST	3	1	-1.32487	1.31084	1.0215	0.3122	.	.	.
IMG_C_TOAST	2	1	0.43593	1.04831	0.1729	0.6775	.	.	.
BMI*IMG_C_TOAST	5	1	-0.06708	0.03178	4.4564	0.0348	.	.	.
BMI*IMG_C_TOAST	4	1	-0.18899	0.11044	2.9282	0.0870	.	.	.
BMI*IMG_C_TOAST	3	1	0.01555	0.05277	0.0869	0.7682	.	.	.
BMI*IMG_C_TOAST	2	1	-0.03556	0.04334	0.6730	0.4120	.	.	.
AGE		1	0.05886	0.00481	149.7490	<.0001	1.061	1.051	1.071
GENDER	2	1	-0.12511	0.10241	1.4925	0.2218	0.882	0.722	1.079
ETHNIC	2	1	0.43362	0.23631	3.3670	0.0665	1.543	0.971	2.452
H_DIAB01	1	1	0.36365	0.10420	12.1795	0.0005	1.439	1.173	1.765
H_AF01	1	1	0.80599	0.14943	29.0943	<.0001	2.239	1.670	3.001
H_HYPT01	1	1	0.04753	0.09794	0.2355	0.6275	1.049	0.866	1.271
H_LIPID01	1	1	-0.31527	0.20771	2.3037	0.1291	0.730	0.486	1.096
AI	1	1	0.52544	0.22661	5.3766	0.0204	1.691	1.085	2.637
H_DRINK_H01	1	1	-0.52042	0.19572	7.0698	0.0078	0.594	0.405	0.872
H_SMK_C01	1	1	0.13016	0.12414	1.0994	0.2944	1.139	0.893	1.453
IT	1	1	-0.72774	0.16612	19.1919	<.0001	0.483	0.349	0.669
ET	1	1	0.87728	0.31356	7.8276	0.0051	2.404	1.300	4.445
A_NIHSS		1	0.09449	0.00629	225.6003	<.0001	1.099	1.086	1.113

BMI with y1_death: interaction with stroke subtype

PHREG 过程

最大似然估计分析		
参数		标签
BMI		F.Physical examination: Body mass index (kg/m2);
IMG_C_TOAST	5	K.Final diagnosis: cerebral infarction; Etiology according to TOAST system; 1-large artery atherosclerosis; 2-cardiogenic embolism; 3-small artery occlusion; 4-stroke of another determined cause; 5-stroke of an undetermined cause. 5
IMG_C_TOAST	4	K.Final diagnosis: cerebral infarction; Etiology according to TOAST system; 1-large artery atherosclerosis; 2-cardiogenic embolism; 3-small artery occlusion; 4-stroke of another determined cause; 5-stroke of an undetermined cause. 4
IMG_C_TOAST	3	K.Final diagnosis: cerebral infarction; Etiology according to TOAST system; 1-large artery atherosclerosis; 2-cardiogenic embolism; 3-small artery occlusion; 4-stroke of another determined cause; 5-stroke of an undetermined cause. 3
IMG_C_TOAST	2	K.Final diagnosis: cerebral infarction; Etiology according to TOAST system; 1-large artery atherosclerosis; 2-cardiogenic embolism; 3-small artery occlusion; 4-stroke of another determined cause; 5-stroke of an undetermined cause. 2
BMI*IMG_C_TOAST	5	K.Final diagnosis: cerebral infarction; Etiology according to TOAST system; 1-large artery atherosclerosis; 2-cardiogenic embolism; 3-small artery occlusion; 4-stroke of another determined cause; 5-stroke of an undetermined cause. 5 * F.Physical examinatio
BMI*IMG_C_TOAST	4	K.Final diagnosis: cerebral infarction; Etiology according to TOAST system; 1-large artery atherosclerosis; 2-cardiogenic embolism; 3-small artery occlusion; 4-stroke of another determined cause; 5-stroke of an undetermined cause. 4 * F.Physical examinatio
BMI*IMG_C_TOAST	3	K.Final diagnosis: cerebral infarction; Etiology according to TOAST system; 1-large artery atherosclerosis; 2-cardiogenic embolism; 3-small artery occlusion; 4-stroke of another determined cause; 5-stroke of an undetermined cause. 3 * F.Physical examinatio
BMI*IMG_C_TOAST	2	K.Final diagnosis: cerebral infarction; Etiology according to TOAST system; 1-large artery atherosclerosis; 2-cardiogenic embolism; 3-small artery occlusion; 4-stroke of another determined cause; 5-stroke of an undetermined cause. 2 * F.Physical examinatio
AGE		A.Basic Information: Age (years old);
GENDER	2	A.Basic Information: Gender; 1-male; 2-female; 2
ETHNIC	2	B.Demography: Race: 1-Han; 99-others; 2
H_DIAB01	1	D.History: Diabetes; 0-No; 1-Yes; 1
H_AF01	1	D.History: Heart disease category: Atrial fibrillation(Including medical history and hospitalization diagnosis); 0-No; 1-Yes; 1
H_HYPT01	1	D.History: Hypertension; 0-No; 1-Yes; 1
H_LIPID01	1	D.History: Lipid metabolism disorders; 0-No; 1-Yes; 1
AI	1	history:Myocardial infarction; 0=NO; 1=YES; 1
H_DRINK_H01	1	D.History: Heavy Drinking(Alcohol consumption>=20g/day); 0-No,1-Yes; 1
H_SMK_C01	1	D.History: Current Smoking; 0-No,1-Yes; 1
IT	1	intravenous thrombolysis, 1=YES,0=NO 1
ET	1	动脉溶栓或机械取栓, 1=YES,0=NO 1
A_NIHSS		F.Admitting NIHSS: Total score;

FREQ 过程

N12.Follow-up events at 12 months:Occurrence of combined vascular event(including cardiovascular death,non-fatal stroke,non-fatal myocardial infarction):0-No;1-Yes;		
y1_comb	频数	累积频数
0	12641	12641
1	1505	14146

等比例的卡方检验	
卡方	8766.4708
自由度	1
Pr > 卡方	<.0001

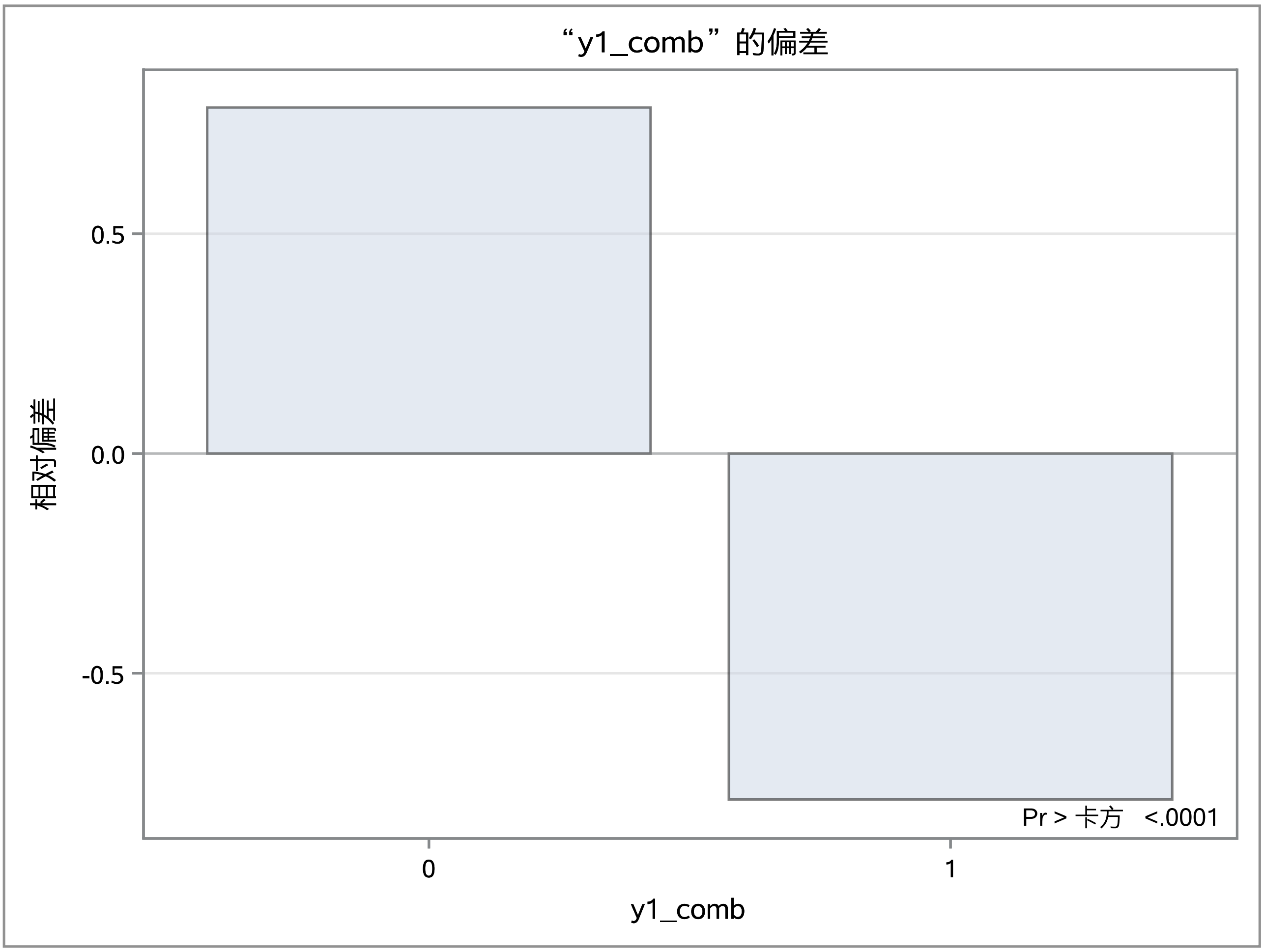

样本大小 = 14146

PHREG 过程

模型信息		
数据集	WORK.DATA2	
因变量	y1_comb_dd	N12.Follow-up events at 12 months: Days from onset to occurrence of combined vascular event;(day);
删失变量	y1_comb	N12.Follow-up events at 12 months:Occurrence of combined vascular event(including cardiovascular death,non-fatal stroke,non-fatal myocardial infarction):0-No;1-Yes;
删失值	0	
结值处理	BRESLOW	

读取的观测数	14146
使用的观测数	14146

事件和删失值个数汇总			
合计	事件	删失	删失百分比
14146	1505	12641	89.36

收敛状态
满足收敛准则 (GCONV=1E-8)。

模型拟合统计量		
准则	无协变量	带协变量
-2 LOG L	28553.433	28553.431
AIC	28553.433	28555.431
SBC	28553.433	28560.747

检验全局原假设: BETA=0			
检验	卡方	自由度	Pr > 卡方
似然比	0.0023	1	0.9622
评分	0.0023	1	0.9621
Wald	0.0023	1	0.9621

最大似然估计分析									
参数	自由度	参数估计	标准误差	卡方	Pr > 卡方	危险率	95% 危险率置信限		标签
BMI	1	0.0003691	0.00777	0.0023	0.9621	1.000	0.985	1.016	F.Physical examination: Body mass index (kg/m2);

BMI with y1_comb: adjusted model

PHREG 过程

模型信息		
数据集	WORK.DATA2	
因变量	y1_comb_dd	N12.Follow-up events at 12 months: Days from onset to occurrence of combined vascular event;(day);
删失变量	y1_comb	N12.Follow-up events at 12 months:Occurrence of combined vascular event(including cardiovascular death,non-fatal stroke,non-fatal myocardial infarction):0-No;1-Yes;
删失值	0	
结值处理	BRESLOW	

读取的观测数	14146
使用的观测数	14146

分类水平信息						
分类	值	设计变量				
GENDER	2	1				
	1	0				
ETHNIC	2	1				
	1	0				
H_DIAB01	1	1				
	0	0				
H_AF01	1	1				
	0	0				
H_HYPT01	1	1				
	0	0				
H_LIPID01	1	1				
	0	0				
AI	1	1				
	0	0				
H_DRINK_H01	1	1				
	0	0				
H_SMK_C01	1	1				
	0	0				
IT	1	1				
	0	0				
ET	1	1				
	0	0				
IMG_C_TOAST	5	1	0	0	0	
	4	0	1	0	0	
	3	0	0	1	0	
	2	0	0	0	1	
	1	0	0	0	0	

BMI with y1_comb: adjusted model

PHREG 过程

事件和删失值个数汇总			
合计	事件	删失	删失百分比
14146	1505	12641	89.36

收敛状态
满足收敛准则 (GCONV=1E-8)。

模型拟合统计量		
准则	无协变量	带协变量
-2 LOG L	28553.433	28382.468
AIC	28553.433	28418.468
SBC	28553.433	28514.166

检验全局原假设: BETA=0			
检验	卡方	自由度	Pr > 卡方
似然比	170.9650	18	<.0001
评分	182.2822	18	<.0001
Wald	178.1860	18	<.0001

3 型检验			
效应	自由度	Wald 卡方	Pr > 卡方
BMI	1	0.7350	0.3913
AGE	1	19.8835	<.0001
GENDER	1	0.2118	0.6453
ETHNIC	1	0.4049	0.5246
H_DIAB01	1	13.9884	0.0002
H_AF01	1	13.5341	0.0002
H_HYPT01	1	2.6768	0.1018
H_LIPID01	1	0.6356	0.4253
AI	1	2.1359	0.1439
H_DRINK_H01	1	1.3017	0.2539
H_SMK_C01	1	0.2681	0.6046
IT	1	0.2592	0.6107
ET	1	6.0265	0.0141
IMG_C_TOAST	4	54.3966	<.0001
A_NIHSS	1	16.2978	<.0001

BMI with y1_comb: adjusted model

PHREG 过程

最大似然估计分析									
参数		自由度	参数<br>估计	标准<br>误差	卡方	Pr > 卡方	危险<br>率	95%<br>危险率置信限	
BMI		1	0.00673	0.00786	0.7350	0.3913	1.007	0.991	1.022
AGE		1	0.01116	0.00250	19.8835	<.0001	1.011	1.006	1.016
GENDER	2	1	0.02789	0.06060	0.2118	0.6453	1.028	0.913	1.158
ETHNIC	2	1	-0.10217	0.16056	0.4049	0.5246	0.903	0.659	1.237
H_DIAB01	1	1	0.22038	0.05892	13.9884	0.0002	1.247	1.111	1.399
H_AF01	1	1	0.42527	0.11560	13.5341	0.0002	1.530	1.220	1.919
H_HYPT01	1	1	0.09141	0.05587	2.6768	0.1018	1.096	0.982	1.223
H_LIPID01	1	1	-0.07951	0.09973	0.6356	0.4253	0.924	0.760	1.123
AI	1	1	0.23084	0.15795	2.1359	0.1439	1.260	0.924	1.717
H_DRINK_H01	1	1	0.09400	0.08239	1.3017	0.2539	1.099	0.935	1.291
H_SMK_C01	1	1	-0.03480	0.06722	0.2681	0.6046	0.966	0.847	1.102
IT	1	1	0.04163	0.08177	0.2592	0.6107	1.043	0.888	1.224
ET	1	1	0.61300	0.24971	6.0265	0.0141	1.846	1.132	3.011
IMG_C_TOAST	5	1	-0.32929	0.06148	28.6838	<.0001	0.719	0.638	0.812
IMG_C_TOAST	4	1	-0.02493	0.22327	0.0125	0.9111	0.975	0.630	1.511
IMG_C_TOAST	3	1	-0.52910	0.07915	44.6864	<.0001	0.589	0.504	0.688
IMG_C_TOAST	2	1	-0.42497	0.13550	9.8357	0.0017	0.654	0.501	0.853
A_NIHSS		1	0.02302	0.00570	16.2978	<.0001	1.023	1.012	1.035

BMI with y1_comb: adjusted model

PHREG 过程

最大似然估计分析		
参数		标签
BMI		F.Physical examination: Body mass index (kg/m2);
AGE		A.Basic Information: Age (years old);
GENDER	2	A.Basic Information: Gender; 1-male; 2-female; 2
ETHNIC	2	B.Demography: Race: 1-Han; 99-others; 2
H_DIAB01	1	D.History: Diabetes; 0-No; 1-Yes; 1
H_AF01	1	D.History: Heart disease category: Atrial fibrillation(Including medical history and hospitalization diagnosis); 0-No; 1-Yes; 1
H_HYPT01	1	D.History: Hypertension; 0-No; 1-Yes; 1
H_LIPID01	1	D.History: Lipid metabolism disorders; 0-No; 1-Yes; 1
AI	1	history:Myocardial infarction; 0=NO; 1=YES; 1
H_DRINK_H01	1	D.History: Heavy Drinking(Alcohol consumption>=20g/day); 0-No,1-Yes; 1
H_SMK_C01	1	D.History: Current Smoking; 0-No,1-Yes; 1
IT	1	intravenous thrombolysis, 1=YES,0=NO 1
ET	1	动脉溶栓或机械取栓, 1=YES,0=NO 1
IMG_C_TOAST	5	K.Final diagnosis: cerebral infarction; Etiology according to TOAST system; 1-large artery atherosclerosis; 2-cardiogenic embolism; 3-small artery occlusion; 4-stroke of another determined cause; 5-stroke of an undetermined cause. 5
IMG_C_TOAST	4	K.Final diagnosis: cerebral infarction; Etiology according to TOAST system; 1-large artery atherosclerosis; 2-cardiogenic embolism; 3-small artery occlusion; 4-stroke of another determined cause; 5-stroke of an undetermined cause. 4
IMG_C_TOAST	3	K.Final diagnosis: cerebral infarction; Etiology according to TOAST system; 1-large artery atherosclerosis; 2-cardiogenic embolism; 3-small artery occlusion; 4-stroke of another determined cause; 5-stroke of an undetermined cause. 3
IMG_C_TOAST	2	K.Final diagnosis: cerebral infarction; Etiology according to TOAST system; 1-large artery atherosclerosis; 2-cardiogenic embolism; 3-small artery occlusion; 4-stroke of another determined cause; 5-stroke of an undetermined cause. 2
A_NIHSS		F.Admitting NIHSS: Total score;

BMI with y1_comb: interaction with stroke subtype

PHREG 过程

模型信息		
数据集	WORK.DATA2	
因变量	y1_comb_dd	N12.Follow-up events at 12 months: Days from onset to occurrence of combined vascular event;(day);
删失变量	y1_comb	N12.Follow-up events at 12 months:Occurrence of combined vascular event(including cardiovascular death,non-fatal stroke,non-fatal myocardial infarction):0-No;1-Yes;
删失值	0	
结值处理	BRESLOW	

读取的观测数	14146
使用的观测数	14146

分类水平信息						
分类	值	设计变量				
GENDER	2	1				
	1	0				
ETHNIC	2	1				
	1	0				
H_DIAB01	1	1				
	0	0				
H_AF01	1	1				
	0	0				
H_HYPT01	1	1				
	0	0				
H_LIPID01	1	1				
	0	0				
AI	1	1				
	0	0				
H_DRINK_H01	1	1				
	0	0				
H_SMK_C01	1	1				
	0	0				
IT	1	1				
	0	0				
ET	1	1				
	0	0				
IMG_C_TOAST	5	1	0	0	0	
	4	0	1	0	0	
	3	0	0	1	0	
	2	0	0	0	1	
	1	0	0	0	0	

BMI with y1_comb: interaction with stroke subtype

PHREG 过程

事件和删失值个数汇总			
合计	事件	删失	删失百分比
14146	1505	12641	89.36

收敛状态
满足收敛准则 (GCONV=1E-8)。

模型拟合统计量		
准则	无协变量	带协变量
-2 LOG L	28553.433	28381.118
AIC	28553.433	28425.118
SBC	28553.433	28542.082

检验全局原假设: BETA=0			
检验	卡方	自由度	Pr > 卡方
似然比	172.3148	22	<.0001
评分	183.9692	22	<.0001
Wald	179.8270	22	<.0001

联合检验			
效应	自由度	Wald 卡方	Pr > 卡方
BMI	1	0.4225	0.5157
IMG_C_TOAST	4	1.3505	0.8527
BMI*IMG_C_TOAST	4	1.3354	0.8553
AGE	1	19.9575	<.0001
GENDER	1	0.2019	0.6532
ETHNIC	1	0.4008	0.5267
H_DIAB01	1	14.2189	0.0002
H_AF01	1	13.6514	0.0002
H_HYPT01	1	2.7328	0.0983
H_LIPID01	1	0.6353	0.4254
AI	1	2.1618	0.1415
H_DRINK_H01	1	1.3098	0.2524
H_SMK_C01	1	0.2642	0.6072
IT	1	0.2322	0.6299
ET	1	6.1164	0.0134
A_NIHSS	1	16.0911	<.0001

Note: Under full-rank parameterizations, Type 3 effect tests are replaced by joint tests. The joint test for an effect is a test that all of the parameters associated with that effect are zero. Such joint tests might not be equivalent to Type 3 effect tests under GLM parameterization.

BMI with y1_comb: interaction with stroke subtype

PHREG 过程

最大似然估计分析									
参数		自由度	参数估计	标准误差	卡方	Pr > 卡方	危险率	95% 危险率置信限	
BMI		1	0.00886	0.01363	0.4225	0.5157	.	.	.
IMG_C_TOAST	5	1	-0.38413	0.44460	0.7465	0.3876	.	.	.
IMG_C_TOAST	4	1	-0.40146	1.20663	0.1107	0.7393	.	.	.
IMG_C_TOAST	3	1	-0.33131	0.59918	0.3057	0.5803	.	.	.
IMG_C_TOAST	2	1	0.26850	0.75077	0.1279	0.7206	.	.	.
BMI*IMG_C_TOAST	5	1	0.00222	0.01779	0.0156	0.9007	.	.	.
BMI*IMG_C_TOAST	4	1	0.01513	0.04732	0.1022	0.7492	.	.	.
BMI*IMG_C_TOAST	3	1	-0.00799	0.02397	0.1110	0.7390	.	.	.
BMI*IMG_C_TOAST	2	1	-0.02859	0.03048	0.8800	0.3482	.	.	.
AGE		1	0.01118	0.00250	19.9575	<.0001	1.011	1.006	1.016
GENDER	2	1	0.02724	0.06062	0.2019	0.6532	1.028	0.912	1.157
ETHNIC	2	1	-0.10166	0.16059	0.4008	0.5267	0.903	0.659	1.237
H_DIAB01	1	1	0.22231	0.05896	14.2189	0.0002	1.249	1.113	1.402
H_AF01	1	1	0.42727	0.11564	13.6514	0.0002	1.533	1.222	1.923
H_HYPT01	1	1	0.09240	0.05589	2.7328	0.0983	1.097	0.983	1.224
H_LIPID01	1	1	-0.07949	0.09973	0.6353	0.4254	0.924	0.760	1.123
AI	1	1	0.23221	0.15794	2.1618	0.1415	1.261	0.926	1.719
H_DRINK_H01	1	1	0.09432	0.08241	1.3098	0.2524	1.099	0.935	1.292
H_SMK_C01	1	1	-0.03457	0.06724	0.2642	0.6072	0.966	0.847	1.102
IT	1	1	0.03943	0.08183	0.2322	0.6299	1.040	0.886	1.221
ET	1	1	0.61785	0.24983	6.1164	0.0134	1.855	1.137	3.027
A_NIHSS		1	0.02290	0.00571	16.0911	<.0001	1.023	1.012	1.035

BMI with y1_comb: interaction with stroke subtype

PHREG 过程

最大似然估计分析		
参数		标签
BMI		F.Physical examination: Body mass index (kg/m2);
IMG_C_TOAST	5	K.Final diagnosis: cerebral infarction; Etiology according to TOAST system; 1-large artery atherosclerosis; 2-cardiogenic embolism; 3-small artery occlusion; 4-stroke of another determined cause; 5-stroke of an undetermined cause. 5
IMG_C_TOAST	4	K.Final diagnosis: cerebral infarction; Etiology according to TOAST system; 1-large artery atherosclerosis; 2-cardiogenic embolism; 3-small artery occlusion; 4-stroke of another determined cause; 5-stroke of an undetermined cause. 4
IMG_C_TOAST	3	K.Final diagnosis: cerebral infarction; Etiology according to TOAST system; 1-large artery atherosclerosis; 2-cardiogenic embolism; 3-small artery occlusion; 4-stroke of another determined cause; 5-stroke of an undetermined cause. 3
IMG_C_TOAST	2	K.Final diagnosis: cerebral infarction; Etiology according to TOAST system; 1-large artery atherosclerosis; 2-cardiogenic embolism; 3-small artery occlusion; 4-stroke of another determined cause; 5-stroke of an undetermined cause. 2
BMI*IMG_C_TOAST	5	K.Final diagnosis: cerebral infarction; Etiology according to TOAST system; 1-large artery atherosclerosis; 2-cardiogenic embolism; 3-small artery occlusion; 4-stroke of another determined cause; 5-stroke of an undetermined cause. 5 * F.Physical examinatio
BMI*IMG_C_TOAST	4	K.Final diagnosis: cerebral infarction; Etiology according to TOAST system; 1-large artery atherosclerosis; 2-cardiogenic embolism; 3-small artery occlusion; 4-stroke of another determined cause; 5-stroke of an undetermined cause. 4 * F.Physical examinatio
BMI*IMG_C_TOAST	3	K.Final diagnosis: cerebral infarction; Etiology according to TOAST system; 1-large artery atherosclerosis; 2-cardiogenic embolism; 3-small artery occlusion; 4-stroke of another determined cause; 5-stroke of an undetermined cause. 3 * F.Physical examinatio
BMI*IMG_C_TOAST	2	K.Final diagnosis: cerebral infarction; Etiology according to TOAST system; 1-large artery atherosclerosis; 2-cardiogenic embolism; 3-small artery occlusion; 4-stroke of another determined cause; 5-stroke of an undetermined cause. 2 * F.Physical examinatio
AGE		A.Basic Information: Age (years old);
GENDER	2	A.Basic Information: Gender; 1-male; 2-female; 2
ETHNIC	2	B.Demography: Race: 1-Han; 99-others; 2
H_DIAB01	1	D.History: Diabetes; 0-No; 1-Yes; 1
H_AF01	1	D.History: Heart disease category: Atrial fibrillation(Including medical history and hospitalization diagnosis); 0-No; 1-Yes; 1
H_HYPT01	1	D.History: Hypertension; 0-No; 1-Yes; 1
H_LIPID01	1	D.History: Lipid metabolism disorders; 0-No; 1-Yes; 1
AI	1	history:Myocardial infarction; 0=NO; 1=YES; 1
H_DRINK_H01	1	D.History: Heavy Drinking(Alcohol consumption>=20g/day); 0-No,1-Yes; 1
H_SMK_C01	1	D.History: Current Smoking; 0-No,1-Yes; 1
IT	1	intravenous thrombolysis, 1=YES,0=NO 1
ET	1	动脉溶栓或机械取栓, 1=YES,0=NO 1
A_NIHSS		F.Admitting NIHSS: Total score;

FREQ 过程

N12.Follow-up events at 12 months: Recurrence of stroke: 0-No; 1-Yes;		
y1_stroke	频数	累积频数
0	12722	12722
1	1424	14146

等比例的卡方检验	
卡方	9023.3850
自由度	1
Pr > 卡方	<.0001

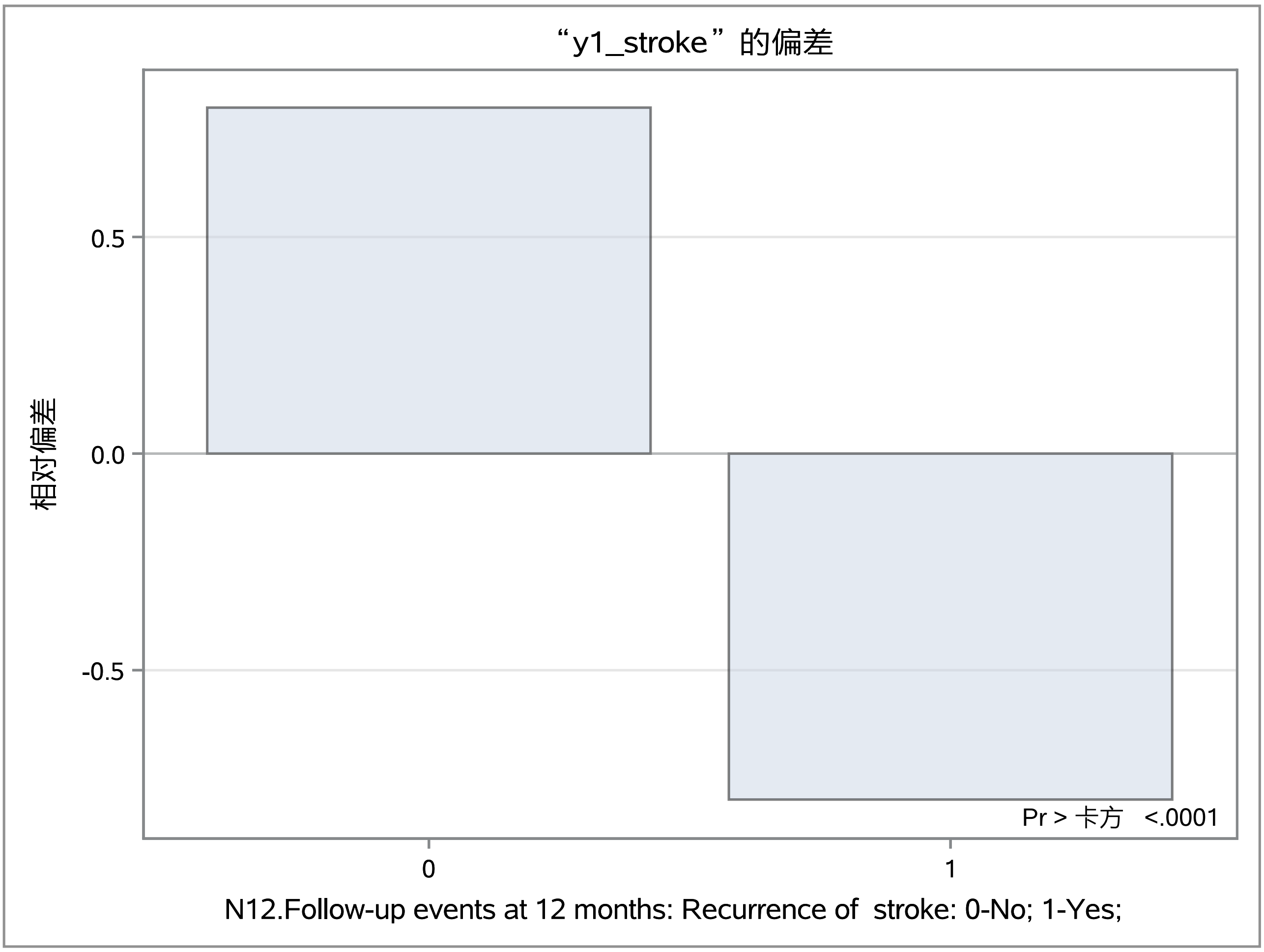

样本大小 = 14146

BMI with y1_stroke: crude model

PHREG 过程

模型信息		
数据集	WORK.DATA2	
因变量	y1_stroke_dd	N12.Follow-up events at 12 months: Days from onset to recurrence;(day);
删失变量	y1_stroke	N12.Follow-up events at 12 months: Recurrence of stroke: 0-No; 1-Yes;
删失值	0	
结值处理	BRESLOW	

读取的观测数<br>使用的观测数	14146<br>14146

事件和删失值个数汇总			
合计	事件	删失	删失<br>百分比
14146	1424	12722	89.93

收敛状态
满足收敛准则 (GCONV=1E-8)。

模型拟合统计量		
准则	无<br>协变量	带<br>协变量
-2 LOG L	27024.892	27024.650
AIC	27024.892	27026.650
SBC	27024.892	27031.911

检验全局原假设: BETA=0			
检验	卡方	自由度	Pr > 卡方
似然比	0.2419	1	0.6228
评分	0.2429	1	0.6221
Wald	0.2457	1	0.6201

最大似然估计分析									
参数	自由度	参数<br>估计	标准<br>误差	卡方	Pr > 卡方	危险<br>率	95%<br>危险率置信限		标签
BMI	1	0.00394	0.00795	0.2457	0.6201	1.004	0.988	1.020	F.Physical examination: Body mass index (kg/m2);

BMI with y1_stroke: adjusted model

PHREG 过程

模型信息		
数据集	WORK.DATA2	
因变量	y1_stroke_dd	N12.Follow-up events at 12 months: Days from onset to recurrence;(day);
删失变量	y1_stroke	N12.Follow-up events at 12 months: Recurrence of stroke: 0-No; 1-Yes;
删失值	0	
结值处理	BRESLOW	

读取的观测数<br>使用的观测数	14146<br>14146

分类水平信息					
分类	值	设计变量			
GENDER	2	1			
	1	0			
ETHNIC	2	1			
	1	0			
H_DIAB01	1	1			
	0	0			
H_AF01	1	1			
	0	0			
H_HYPT01	1	1			
	0	0			
H_LIPID01	1	1			
	0	0			
AI	1	1			
	0	0			
H_DRINK_H01	1	1			
	0	0			
H_SMK_C01	1	1			
	0	0			
IT	1	1			
	0	0			
ET	1	1			
	0	0			
IMG_C_TOAST	5	1	0	0	0
	4	0	1	0	0
	3	0	0	1	0
	2	0	0	0	1
	1	0	0	0	0

BMI with y1_stroke: adjusted model

PHREG 过程

事件和删失值个数汇总			
合计	事件	删失	删失<br>百分比
14146	1424	12722	89.93

收敛状态
满足收敛准则 (GCONV=1E-8)。

模型拟合统计量		
准则	无<br>协变量	带<br>协变量
-2 LOG L	27024.892	26878.721
AIC	27024.892	26914.721
SBC	27024.892	27009.423

检验全局原假设: BETA=0			
检验	卡方	自由度	Pr > 卡方
似然比	146.1706	18	<.0001
评分	154.8140	18	<.0001
Wald	151.6134	18	<.0001

3 型检验			
效应	自由度	Wald 卡方	Pr > 卡方
BMI	1	1.5617	0.2114
AGE	1	15.4044	<.0001
GENDER	1	0.5341	0.4649
ETHNIC	1	0.5707	0.4500
H_DIAB01	1	11.7512	0.0006
H_AF01	1	8.5658	0.0034
H_HYPT01	1	1.2781	0.2583
H_LIPID01	1	0.5972	0.4396
AI	1	1.0132	0.3141
H_DRINK_H01	1	3.0215	0.0822
H_SMK_C01	1	0.7008	0.4025
IT	1	1.1319	0.2874
ET	1	5.9079	0.0151
IMG_C_TOAST	4	52.8403	<.0001
A_NIHSS	1	12.9654	0.0003

BMI with y1_stroke: adjusted model

PHREG 过程

最大似然估计分析

参数		自由度	参数估计	标准误差	卡方	Pr > 卡方	危险率	95% 危险率置信限	
BMI		1	0.01005	0.00804	1.5617	0.2114	1.010	0.994	1.026
AGE		1	0.01006	0.00256	15.4044	<.0001	1.010	1.005	1.015
GENDER	2	1	0.04557	0.06235	0.5341	0.4649	1.047	0.926	1.183
ETHNIC	2	1	-0.12608	0.16689	0.5707	0.4500	0.882	0.636	1.223
H_DIAB01	1	1	0.20819	0.06073	11.7512	0.0006	1.231	1.093	1.387
H_AF01	1	1	0.35694	0.12196	8.5658	0.0034	1.429	1.125	1.815
H_HYPT01	1	1	0.06470	0.05723	1.2781	0.2583	1.067	0.954	1.193
H_LIPID01	1	1	-0.07924	0.10253	0.5972	0.4396	0.924	0.756	1.129
AI	1	1	0.16913	0.16802	1.0132	0.3141	1.184	0.852	1.646
H_DRINK_H01	1	1	0.14553	0.08372	3.0215	0.0822	1.157	0.982	1.363
H_SMK_C01	1	1	-0.05793	0.06920	0.7008	0.4025	0.944	0.824	1.081
IT	1	1	0.08837	0.08306	1.1319	0.2874	1.092	0.928	1.286
ET	1	1	0.62569	0.25742	5.9079	0.0151	1.870	1.129	3.096
IMG_C_TOAST	5	1	-0.33713	0.06298	28.6538	<.0001	0.714	0.631	0.808
IMG_C_TOAST	4	1	-0.03587	0.22878	0.0246	0.8754	0.965	0.616	1.511
IMG_C_TOAST	3	1	-0.52847	0.08089	42.6836	<.0001	0.590	0.503	0.691
IMG_C_TOAST	2	1	-0.44750	0.14207	9.9214	0.0016	0.639	0.484	0.844
A_NIHSS		1	0.02135	0.00593	12.9654	0.0003	1.022	1.010	1.034

BMI with y1_stroke: adjusted model

PHREG 过程

最大似然估计分析		
参数		标签
BMI		F.Physical examination: Body mass index (kg/m2);
AGE		A.Basic Information: Age (years old);
GENDER	2	A.Basic Information: Gender; 1-male; 2-female; 2
ETHNIC	2	B.Demography: Race: 1-Han; 99-others; 2
H_DIAB01	1	D.History: Diabetes; 0-No; 1-Yes; 1
H_AF01	1	D.History: Heart disease category: Atrial fibrillation(Including medical history and hospitalization diagnosis); 0-No; 1-Yes; 1
H_HYPT01	1	D.History: Hypertension; 0-No; 1-Yes; 1
H_LIPID01	1	D.History: Lipid metabolism disorders; 0-No; 1-Yes; 1
AI	1	history:Myocardial infarction; 0=NO; 1=YES; 1
H_DRINK_H01	1	D.History: Heavy Drinking(Alcohol consumption>=20g/day); 0-No,1-Yes; 1
H_SMK_C01	1	D.History: Current Smoking; 0-No,1-Yes; 1
IT	1	intravenous thrombolysis, 1=YES,0=NO 1
ET	1	动脉溶栓或机械取栓, 1=YES,0=NO 1
IMG_C_TOAST	5	K.Final diagnosis: cerebral infarction; Etiology according to TOAST system; 1-large artery atherosclerosis; 2-cardiogenic embolism; 3-small artery occlusion; 4-stroke of another determined cause; 5-stroke of an undetermined cause. 5
IMG_C_TOAST	4	K.Final diagnosis: cerebral infarction; Etiology according to TOAST system; 1-large artery atherosclerosis; 2-cardiogenic embolism; 3-small artery occlusion; 4-stroke of another determined cause; 5-stroke of an undetermined cause. 4
IMG_C_TOAST	3	K.Final diagnosis: cerebral infarction; Etiology according to TOAST system; 1-large artery atherosclerosis; 2-cardiogenic embolism; 3-small artery occlusion; 4-stroke of another determined cause; 5-stroke of an undetermined cause. 3
IMG_C_TOAST	2	K.Final diagnosis: cerebral infarction; Etiology according to TOAST system; 1-large artery atherosclerosis; 2-cardiogenic embolism; 3-small artery occlusion; 4-stroke of another determined cause; 5-stroke of an undetermined cause. 2
A_NIHSS		F.Admitting NIHSS: Total score;

BMI with y1_stroke: interaction with stroke subtype

PHREG 过程

模型信息		
数据集	WORK.DATA2	
因变量	y1_stroke_dd	N12.Follow-up events at 12 months: Days from onset to recurrence;(day);
删失变量	y1_stroke	N12.Follow-up events at 12 months: Recurrence of stroke: 0-No; 1-Yes;
删失值	0	
结值处理	BRESLOW	

读取的观测数<br>使用的观测数	14146<br>14146

分类水平信息					
分类	值	设计变量			
GENDER	2	1			
	1	0			
ETHNIC	2	1			
	1	0			
H_DIAB01	1	1			
	0	0			
H_AF01	1	1			
	0	0			
H_HYPT01	1	1			
	0	0			
H_LIPID01	1	1			
	0	0			
AI	1	1			
	0	0			
H_DRINK_H01	1	1			
	0	0			
H_SMK_C01	1	1			
	0	0			
IT	1	1			
	0	0			
ET	1	1			
	0	0			
IMG_C_TOAST	5	1	0	0	0
	4	0	1	0	0
	3	0	0	1	0
	2	0	0	0	1
	1	0	0	0	0

BMI with y1_stroke: interaction with stroke subtype

PHREG 过程

事件和删失值个数汇总			
合计	事件	删失	删失<br>百分比
14146	1424	12722	89.93

收敛状态
满足收敛准则 (GCONV=1E-8)。

模型拟合统计量		
准则	无<br>协变量	带<br>协变量
-2 LOG L	27024.892	26878.018
AIC	27024.892	26922.018
SBC	27024.892	27037.765

检验全局原假设: BETA=0			
检验	卡方	自由度	Pr > 卡方
似然比	146.8743	22	<.0001
评分	155.7789	22	<.0001
Wald	152.5498	22	<.0001

联合检验			
效应	自由度	Wald 卡方	Pr > 卡方
BMI	1	0.7083	0.4000
IMG_C_TOAST	4	0.9243	0.9211
BMI*IMG_C_TOAST	4	0.7120	0.9498
AGE	1	15.3961	<.0001
GENDER	1	0.5247	0.4688
ETHNIC	1	0.5651	0.4522
H_DIAB01	1	11.8983	0.0006
H_AF01	1	8.6321	0.0033
H_HYPT01	1	1.3082	0.2527
H_LIPID01	1	0.5997	0.4387
AI	1	1.0300	0.3102
H_DRINK_H01	1	3.0327	0.0816
H_SMK_C01	1	0.6977	0.4036
IT	1	1.0969	0.2949
ET	1	5.9700	0.0146
A_NIHSS	1	12.8188	0.0003

Note: Under full-rank parameterizations, Type 3 effect tests are replaced by joint tests. The joint test for an effect is a test that all of the parameters associated with that effect are zero. Such joint tests might not be equivalent to Type 3 effect tests under GLM parameterization.

BMI with y1_stroke: interaction with stroke subtype

PHREG 过程

最大似然估计分析									
参数		自由度	参数估计	标准误差	卡方	Pr > 卡方	危险率	95% 危险率置信限	
BMI		1	0.01168	0.01388	0.7083	0.4000	.	.	.
IMG_C_TOAST	5	1	-0.34848	0.45517	0.5862	0.4439	.	.	.
IMG_C_TOAST	4	1	-0.50067	1.20362	0.1730	0.6774	.	.	.
IMG_C_TOAST	3	1	-0.39598	0.60943	0.4222	0.5158	.	.	.
IMG_C_TOAST	2	1	0.05054	0.78756	0.0041	0.9488	.	.	.
BMI*IMG_C_TOAST	5	1	0.0004588	0.01819	0.0006	0.9799	.	.	.
BMI*IMG_C_TOAST	4	1	0.01860	0.04693	0.1571	0.6918	.	.	.
BMI*IMG_C_TOAST	3	1	-0.00534	0.02433	0.0482	0.8262	.	.	.
BMI*IMG_C_TOAST	2	1	-0.02047	0.03186	0.4129	0.5205	.	.	.
AGE		1	0.01006	0.00256	15.3961	<.0001	1.010	1.005	1.015
GENDER	2	1	0.04517	0.06236	0.5247	0.4688	1.046	0.926	1.182
ETHNIC	2	1	-0.12547	0.16691	0.5651	0.4522	0.882	0.636	1.223
H_DIAB01	1	1	0.20959	0.06076	11.8983	0.0006	1.233	1.095	1.389
H_AF01	1	1	0.35841	0.12199	8.6321	0.0033	1.431	1.127	1.818
H_HYPT01	1	1	0.06548	0.05725	1.3082	0.2527	1.068	0.954	1.194
H_LIPID01	1	1	-0.07940	0.10253	0.5997	0.4387	0.924	0.756	1.129
AI	1	1	0.17052	0.16802	1.0300	0.3102	1.186	0.853	1.648
H_DRINK_H01	1	1	0.14583	0.08374	3.0327	0.0816	1.157	0.982	1.363
H_SMK_C01	1	1	-0.05782	0.06923	0.6977	0.4036	0.944	0.824	1.081
IT	1	1	0.08704	0.08311	1.0969	0.2949	1.091	0.927	1.284
ET	1	1	0.62924	0.25753	5.9700	0.0146	1.876	1.133	3.108
A_NIHSS		1	0.02124	0.00593	12.8188	0.0003	1.021	1.010	1.033

BMI with y1_stroke: interaction with stroke subtype

PHREG 过程

最大似然估计分析		
参数		标签
BMI		F.Physical examination: Body mass index (kg/m2);
IMG_C_TOAST	5	K.Final diagnosis: cerebral infarction; Etiology according to TOAST system; 1-large artery atherosclerosis; 2-cardiogenic embolism; 3-small artery occlusion; 4-stroke of another determined cause; 5-stroke of an undetermined cause. 5
IMG_C_TOAST	4	K.Final diagnosis: cerebral infarction; Etiology according to TOAST system; 1-large artery atherosclerosis; 2-cardiogenic embolism; 3-small artery occlusion; 4-stroke of another determined cause; 5-stroke of an undetermined cause. 4
IMG_C_TOAST	3	K.Final diagnosis: cerebral infarction; Etiology according to TOAST system; 1-large artery atherosclerosis; 2-cardiogenic embolism; 3-small artery occlusion; 4-stroke of another determined cause; 5-stroke of an undetermined cause. 3
IMG_C_TOAST	2	K.Final diagnosis: cerebral infarction; Etiology according to TOAST system; 1-large artery atherosclerosis; 2-cardiogenic embolism; 3-small artery occlusion; 4-stroke of another determined cause; 5-stroke of an undetermined cause. 2
BMI*IMG_C_TOAST	5	K.Final diagnosis: cerebral infarction; Etiology according to TOAST system; 1-large artery atherosclerosis; 2-cardiogenic embolism; 3-small artery occlusion; 4-stroke of another determined cause; 5-stroke of an undetermined cause. 5 * F.Physical examinatio
BMI*IMG_C_TOAST	4	K.Final diagnosis: cerebral infarction; Etiology according to TOAST system; 1-large artery atherosclerosis; 2-cardiogenic embolism; 3-small artery occlusion; 4-stroke of another determined cause; 5-stroke of an undetermined cause. 4 * F.Physical examinatio
BMI*IMG_C_TOAST	3	K.Final diagnosis: cerebral infarction; Etiology according to TOAST system; 1-large artery atherosclerosis; 2-cardiogenic embolism; 3-small artery occlusion; 4-stroke of another determined cause; 5-stroke of an undetermined cause. 3 * F.Physical examinatio
BMI*IMG_C_TOAST	2	K.Final diagnosis: cerebral infarction; Etiology according to TOAST system; 1-large artery atherosclerosis; 2-cardiogenic embolism; 3-small artery occlusion; 4-stroke of another determined cause; 5-stroke of an undetermined cause. 2 * F.Physical examinatio
AGE		A.Basic Information: Age (years old);
GENDER	2	A.Basic Information: Gender; 1-male; 2-female; 2
ETHNIC	2	B.Demography: Race: 1-Han; 99-others; 2
H_DIAB01	1	D.History: Diabetes; 0-No; 1-Yes; 1
H_AF01	1	D.History: Heart disease category: Atrial fibrillation(Including medical history and hospitalization diagnosis); 0-No; 1-Yes; 1
H_HYPT01	1	D.History: Hypertension; 0-No; 1-Yes; 1
H_LIPID01	1	D.History: Lipid metabolism disorders; 0-No; 1-Yes; 1
AI	1	history:Myocardial infarction; 0=NO; 1=YES; 1
H_DRINK_H01	1	D.History: Heavy Drinking(Alcohol consumption>=20g/day); 0-No,1-Yes; 1
H_SMK_C01	1	D.History: Current Smoking; 0-No,1-Yes; 1
IT	1	intravenous thrombolysis, 1=YES,0=NO 1
ET	1	动脉溶栓或机械取栓, 1=YES,0=NO 1
A_NIHSS		F.Admitting NIHSS: Total score;

BMI_g with y1_death: Descriptive results

FREQ 过程

频数
行百分比

BMI_g-y1_death表			
	y1_death(N12.Follow-up events at 12 months: Whether the patient died: 0-survival;1-death;)		
BMI_g(1=<18.5;2=18.5-<24;3=24-<28;4=≥28)	0	1	合计
1	277<br>89.64	32<br>10.36	309
2	5447<br>95.93	231<br>4.07	5678
3	6040<br>97.18	175<br>2.82	6215
4	1896<br>97.53	48<br>2.47	1944
合计	13660	486	14146

表“y1_death-BMI_g”的统计量

统计量	自由度	值	概率
卡方	3	64.1292	<.0001
似然比卡方检验	3	49.6080	<.0001
Mantel-Haenszel 卡方	1	38.0924	<.0001
Phi 系数		0.0673	
列联系数		0.0672	
Cramer V		0.0673	

样本大小 = 14146

BMI_g with y1_death: crude model

PHREG 过程

模型信息		
数据集	WORK.DATA2	
因变量	y1_death_dd	N12.Follow-up events at 12 months: Days from onset to death;(day);
删失变量	y1_death	N12.Follow-up events at 12 months: Whether the patient died: 0-survival;1-death;
删失值	0	
结值处理	BRESLOW	

读取的观测数<br>使用的观测数	14146<br>14146

分类水平信息				
分类	值	设计变量		
BMI_g	4	1	0	0
	3	0	1	0
	2	0	0	0
	1	0	0	1

事件和删失值个数汇总			
合计	事件	删失	删失<br>百分比
14146	486	13660	96.56

收敛状态
满足收敛准则 (GCONV=1E-8)。

模型拟合统计量		
准则	无<br>协变量	带<br>协变量
-2 LOG L	9255.945	9206.274
AIC	9255.945	9212.274
SBC	9255.945	9224.833

检验全局原假设: BETA=0			
检验	卡方	自由度	Pr > 卡方
似然比	49.6707	3	<.0001
评分	65.9623	3	<.0001
Wald	59.8933	3	<.0001

3 型检验			
效应	自由度	Wald 卡方	Pr > 卡方
BMI_g	3	59.8933	<.0001

BMI_g with y1_death: crude model

PHREG 过程

最大似然估计分析

参数		自由度	参数估计	标准误差	卡方	Pr > 卡方	危险率	95% 危险率置信限		标签
BMI_g	4	1	-0.50836	0.15863	10.2701	0.0014	0.601	0.441	0.821	1=<18.5;2=18.5-<24;3=24-<28;4= ≥ 28 4
BMI_g	3	1	-0.37275	0.10022	13.8339	0.0002	0.689	0.566	0.838	1=<18.5;2=18.5-<24;3=24-<28;4= ≥ 28 3
BMI_g	1	1	0.97166	0.18859	26.5446	<.0001	2.642	1.826	3.824	1=<18.5;2=18.5-<24;3=24-<28;4= ≥ 28 1

BMI_g with y1_death: adjusted model

PHREG 过程

模型信息		
数据集	WORK.DATA2	
因变量	y1_death_dd	N12.Follow-up events at 12 months: Days from onset to death;(day);
删失变量	y1_death	N12.Follow-up events at 12 months: Whether the patient died: 0-survival;1-death;
删失值	0	
结值处理	BRESLOW	

读取的观测数	14146
使用的观测数	14146

分类水平信息					
分类	值	设计变量			
BMI_g	4	1	0	0	
	3	0	1	0	
	2	0	0	0	
	1	0	0	1	
GENDER	2	1			
	1	0			
ETHNIC	2	1			
	1	0			
H_DIAB01	1	1			
	0	0			
H_AF01	1	1			
	0	0			
H_HYPT01	1	1			
	0	0			
H_LIPID01	1	1			
	0	0			
AI	1	1			
	0	0			
H_DRINK_H01	1	1			
	0	0			
H_SMK_C01	1	1			
	0	0			
IT	1	1			
	0	0			
ET	1	1			
	0	0			
IMG_C_TOAST	5	1	0	0	0
	4	0	1	0	0
	3	0	0	1	0

BMI_g with y1_death: adjusted model

PHREG 过程

分类水平信息					
分类	值	设计变量			
	2	0	0	0	1
	1	0	0	0	0

事件和删失值个数汇总			
合计	事件	删失	删失百分比
14146	486	13660	96.56

收敛状态
满足收敛准则 (GCONV=1E-8)。

模型拟合统计量		
准则	无协变量	带协变量
-2 LOG L	9255.945	8609.223
AIC	9255.945	8649.223
SBC	9255.945	8732.947

检验全局原假设: BETA=0			
检验	卡方	自由度	Pr > 卡方
似然比	646.7222	20	<.0001
评分	881.5150	20	<.0001
Wald	757.7702	20	<.0001

3 型检验			
效应	自由度	Wald 卡方	Pr > 卡方
BMI_g	3	12.8399	0.0050
AGE	1	149.8023	<.0001
GENDER	1	1.6722	0.1960
ETHNIC	1	3.2522	0.0713
H_DIAB01	1	12.9800	0.0003
H_AF01	1	29.5848	<.0001
H_HYPT01	1	0.1544	0.6943
H_LIPID01	1	2.3080	0.1287
AI	1	5.0653	0.0244
H_DRINK_H01	1	6.9029	0.0086
H_SMK_C01	1	0.8929	0.3447
IT	1	19.2117	<.0001
ET	1	7.3391	0.0067
IMG_C_TOAST	4	34.4841	<.0001
A_NIHSS	1	225.3297	<.0001

BMI_g with y1_death: adjusted model

PHREG 过程

最大似然估计分析									
参数		自由度	参数估计	标准误差	卡方	Pr > 卡方	危险率	95% 危险率置信限	
BMI_g	4	1	-0.22354	0.16179	1.9090	0.1671	0.800	0.582	1.098
BMI_g	3	1	-0.15259	0.10222	2.2281	0.1355	0.858	0.703	1.049
BMI_g	1	1	0.51361	0.19151	7.1924	0.0073	1.671	1.148	2.433
AGE		1	0.05875	0.00480	149.8023	<.0001	1.061	1.051	1.071
GENDER	2	1	-0.13283	0.10272	1.6722	0.1960	0.876	0.716	1.071
ETHNIC	2	1	0.42511	0.23573	3.2522	0.0713	1.530	0.964	2.428
H_DIAB01	1	1	0.37605	0.10438	12.9800	0.0003	1.457	1.187	1.787
H_AF01	1	1	0.81205	0.14930	29.5848	<.0001	2.253	1.681	3.018
H_HYPT01	1	1	0.03836	0.09762	0.1544	0.6943	1.039	0.858	1.258
H_LIPID01	1	1	-0.31571	0.20781	2.3080	0.1287	0.729	0.485	1.096
AI	1	1	0.50961	0.22643	5.0653	0.0244	1.665	1.068	2.595
H_DRINK_H01	1	1	-0.51449	0.19582	6.9029	0.0086	0.598	0.407	0.877
H_SMK_C01	1	1	0.11735	0.12419	0.8929	0.3447	1.125	0.882	1.434
IT	1	1	-0.72852	0.16621	19.2117	<.0001	0.483	0.348	0.668
ET	1	1	0.84858	0.31324	7.3391	0.0067	2.336	1.264	4.317
IMG_C_TOAST	5	1	-0.24082	0.11133	4.6788	0.0305	0.786	0.632	0.978
IMG_C_TOAST	4	1	0.62972	0.32739	3.6997	0.0544	1.877	0.988	3.566
IMG_C_TOAST	3	1	-0.93180	0.18259	26.0444	<.0001	0.394	0.275	0.563
IMG_C_TOAST	2	1	-0.42070	0.19359	4.7228	0.0298	0.657	0.449	0.960
A_NIHSS		1	0.09457	0.00630	225.3297	<.0001	1.099	1.086	1.113

BMI_g with y1_death: adjusted model

PHREG 过程

最大似然估计分析		
参数		标签
BMI_g	4	1=<18.5;2=18.5-<24;3=24-<28;4= ≥ 28 4
BMI_g	3	1=<18.5;2=18.5-<24;3=24-<28;4= ≥ 28 3
BMI_g	1	1=<18.5;2=18.5-<24;3=24-<28;4= ≥ 28 1
AGE		A.Basic Information: Age (years old);
GENDER	2	A.Basic Information: Gender; 1-male; 2-female; 2
ETHNIC	2	B.Demography: Race: 1-Han; 99-others; 2
H_DIAB01	1	D.History: Diabetes; 0-No; 1-Yes; 1
H_AF01	1	D.History: Heart disease category: Atrial fibrillation(Including medical history and hospitalization diagnosis); 0-No; 1-Yes; 1
H_HYPT01	1	D.History: Hypertension; 0-No; 1-Yes; 1
H_LIPID01	1	D.History: Lipid metabolism disorders; 0-No; 1-Yes; 1
AI	1	history:Myocardial infarction; 0=NO; 1=YES; 1
H_DRINK_H01	1	D.History: Heavy Drinking(Alcohol consumption>=20g/day); 0-No,1-Yes; 1
H_SMK_C01	1	D.History: Current Smoking; 0-No,1-Yes; 1
IT	1	intravenous thrombolysis, 1=YES,0=NO 1
ET	1	动脉溶栓或机械取栓, 1=YES,0=NO 1
IMG_C_TOAST	5	K.Final diagnosis: cerebral infarction; Etiology according to TOAST system; 1-large artery atherosclerosis; 2-cardiogenic embolism; 3-small artery occlusion; 4-stroke of another determined cause; 5-stroke of an undetermined cause. 5
IMG_C_TOAST	4	K.Final diagnosis: cerebral infarction; Etiology according to TOAST system; 1-large artery atherosclerosis; 2-cardiogenic embolism; 3-small artery occlusion; 4-stroke of another determined cause; 5-stroke of an undetermined cause. 4
IMG_C_TOAST	3	K.Final diagnosis: cerebral infarction; Etiology according to TOAST system; 1-large artery atherosclerosis; 2-cardiogenic embolism; 3-small artery occlusion; 4-stroke of another determined cause; 5-stroke of an undetermined cause. 3
IMG_C_TOAST	2	K.Final diagnosis: cerebral infarction; Etiology according to TOAST system; 1-large artery atherosclerosis; 2-cardiogenic embolism; 3-small artery occlusion; 4-stroke of another determined cause; 5-stroke of an undetermined cause. 2
A_NIHSS		F.Admitting NIHSS: Total score;

BMI_g with y1_death: interaction with stroke subtype

PHREG 过程

模型信息		
数据集	WORK.DATA2	
因变量	y1_death_dd	N12.Follow-up events at 12 months: Days from onset to death;(day);
删失变量	y1_death	N12.Follow-up events at 12 months: Whether the patient died: 0-survival;1-death;
删失值	0	
结值处理	BRESLOW	

读取的观测数	14146
使用的观测数	14146

分类水平信息					
分类	值	设计变量			
BMI_g	4	1	0	0	
	3	0	1	0	
	2	0	0	0	
	1	0	0	1	
GENDER	2	1			
	1	0			
ETHNIC	2	1			
	1	0			
H_DIAB01	1	1			
	0	0			
H_AF01	1	1			
	0	0			
H_HYPT01	1	1			
	0	0			
H_LIPID01	1	1			
	0	0			
AI	1	1			
	0	0			
H_DRINK_H01	1	1			
	0	0			
H_SMK_C01	1	1			
	0	0			
IT	1	1			
	0	0			
ET	1	1			
	0	0			
IMG_C_TOAST	5	1	0	0	0
	4	0	1	0	0
	3	0	0	1	0

BMI_g with y1_death: interaction with stroke subtype

PHREG 过程

分类水平信息					
分类	值	设计变量			
	2	0	0	0	1
	1	0	0	0	0

事件和删失值个数汇总			
合计	事件	删失	删失百分比
14146	486	13660	96.56

收敛状态
满足收敛准则 (GCONV=1E-8)。

模型拟合统计量		
准则	无协变量	带协变量
-2 LOG L	9255.945	8595.552
AIC	9255.945	8659.552
SBC	9255.945	8793.510

检验全局原假设: BETA=0			
检验	卡方	自由度	Pr > 卡方
似然比	660.3930	32	<.0001
评分	933.0829	32	<.0001
Wald	784.0547	32	<.0001

联合检验			
效应	自由度	Wald 卡方	Pr > 卡方
BMI_g	3	0.3408	0.9522
IMG_C_TOAST	4	20.6740	0.0004
BMI_g*IMG_C_TOAST	12	10.3183	0.5881
AGE	1	148.8412	<.0001
GENDER	1	1.6026	0.2055
ETHNIC	1	2.9778	0.0844
H_DIAB01	1	12.6807	0.0004
H_AF01	1	29.5652	<.0001
H_HYPT01	1	0.2422	0.6226
H_LIPID01	1	2.2549	0.1332
AI	1	5.2160	0.0224
H_DRINK_H01	1	7.1127	0.0077
H_SMK_C01	1	1.0863	0.2973
IT	1	19.3504	<.0001

BMI_g with y1_death: interaction with stroke subtype

PHREG 过程

联合检验			
效应	自由度	Wald 卡方	Pr > 卡方
ET	1	8.0643	0.0045
A_NIHSS	1	218.5801	<.0001

Note: Under full-rank parameterizations, Type 3 effect tests are replaced by joint tests. The joint test for an effect is a test that all of the parameters associated with that effect are zero. Such joint tests might not be equivalent to Type 3 effect tests under GLM parameterization.

BMI_g with y1_death: interaction with stroke subtype

PHREG 过程

最大似然估计分析										
参数			自由度	参数估计	标准误差	卡方	Pr > 卡方	危险率	95% 危险率置信限	
BMI_g	4		1	-0.08929	0.27177	0.1080	0.7425	.	.	.
BMI_g	3		1	-0.07608	0.17574	0.1874	0.6651	.	.	.
BMI_g	1		1	-0.20897	0.51560	0.1643	0.6853	.	.	.
IMG_C_TOAST	5		1	-0.22511	0.15833	2.0215	0.1551	.	.	.
IMG_C_TOAST	4		1	0.66652	0.46326	2.0701	0.1502	.	.	.
IMG_C_TOAST	3		1	-1.28661	0.31303	16.8937	<.0001	.	.	.
IMG_C_TOAST	2		1	-0.19979	0.23695	0.7110	0.3991	.	.	.
BMI_g*IMG_C_TOAST	4	5	1	-0.17021	0.36416	0.2185	0.6402	.	.	.
BMI_g*IMG_C_TOAST	4	4	1	-11.05759	217.10062	0.0026	0.9594	.	.	.
BMI_g*IMG_C_TOAST	4	3	1	0.41624	0.59716	0.4859	0.4858	.	.	.
BMI_g*IMG_C_TOAST	4	2	1	-0.47886	0.52035	0.8469	0.3574	.	.	.
BMI_g*IMG_C_TOAST	3	5	1	-0.12011	0.23138	0.2695	0.6037	.	.	.
BMI_g*IMG_C_TOAST	3	4	1	-0.09044	0.75159	0.0145	0.9042	.	.	.
BMI_g*IMG_C_TOAST	3	3	1	0.52501	0.40819	1.6543	0.1984	.	.	.
BMI_g*IMG_C_TOAST	3	2	1	-0.50817	0.34241	2.2026	0.1378	.	.	.
BMI_g*IMG_C_TOAST	1	5	1	0.95713	0.56810	2.8385	0.0920	.	.	.
BMI_g*IMG_C_TOAST	1	4	1	1.42115	0.99592	2.0363	0.1536	.	.	.
BMI_g*IMG_C_TOAST	1	3	1	1.37250	0.92201	2.2159	0.1366	.	.	.
BMI_g*IMG_C_TOAST	1	2	1	0.14757	0.79196	0.0347	0.8522	.	.	.
AGE			1	0.05878	0.00482	148.8412	<.0001	1.061	1.051	1.071
GENDER	2		1	-0.13031	0.10294	1.6026	0.2055	0.878	0.717	1.074
ETHNIC	2		1	0.41600	0.24107	2.9778	0.0844	1.516	0.945	2.431
H_DIAB01	1		1	0.37258	0.10463	12.6807	0.0004	1.451	1.182	1.782
H_AF01	1		1	0.81188	0.14931	29.5652	<.0001	2.252	1.681	3.018
H_HYPT01	1		1	0.04824	0.09802	0.2422	0.6226	1.049	0.866	1.272

BMI_g with y1_death: interaction with stroke subtype

PHREG 过程

最大似然估计分析			
参数			标签
BMI_g	4		1=<18.5;2=18.5-<24;3=24-<28;4= ≥ 28 4
BMI_g	3		1=<18.5;2=18.5-<24;3=24-<28;4= ≥ 28 3
BMI_g	1		1=<18.5;2=18.5-<24;3=24-<28;4= ≥ 28 1
IMG_C_TOAST	5		K.Final diagnosis: cerebral infarction; Etiology according to TOAST system; 1-large artery atherosclerosis; 2-cardiogenic embolism; 3-small artery occlusion; 4-stroke of another determined cause; 5-stroke of an undetermined cause. 5
IMG_C_TOAST	4		K.Final diagnosis: cerebral infarction; Etiology according to TOAST system; 1-large artery atherosclerosis; 2-cardiogenic embolism; 3-small artery occlusion; 4-stroke of another determined cause; 5-stroke of an undetermined cause. 4
IMG_C_TOAST	3		K.Final diagnosis: cerebral infarction; Etiology according to TOAST system; 1-large artery atherosclerosis; 2-cardiogenic embolism; 3-small artery occlusion; 4-stroke of another determined cause; 5-stroke of an undetermined cause. 3
IMG_C_TOAST	2		K.Final diagnosis: cerebral infarction; Etiology according to TOAST system; 1-large artery atherosclerosis; 2-cardiogenic embolism; 3-small artery occlusion; 4-stroke of another determined cause; 5-stroke of an undetermined cause. 2
BMI_g*IMG_C_TOAST	4	5	1=<18.5;2=18.5-<24;3=24-<28;4= ≥ 28 4 * K.Final diagnosis: cerebral infarction; Etiology according to TOAST system; 1-large artery atherosclerosis; 2-cardiogenic embolism; 3-small artery occlusion; 4-stroke of another determined cause; 5-stroke of an undete
BMI_g*IMG_C_TOAST	4	4	1=<18.5;2=18.5-<24;3=24-<28;4= ≥ 28 4 * K.Final diagnosis: cerebral infarction; Etiology according to TOAST system; 1-large artery atherosclerosis; 2-cardiogenic embolism; 3-small artery occlusion; 4-stroke of another determined cause; 5-stroke of an undete
BMI_g*IMG_C_TOAST	4	3	1=<18.5;2=18.5-<24;3=24-<28;4= ≥ 28 4 * K.Final diagnosis: cerebral infarction; Etiology according to TOAST system; 1-large artery atherosclerosis; 2-cardiogenic embolism; 3-small artery occlusion; 4-stroke of another determined cause; 5-stroke of an undete
BMI_g*IMG_C_TOAST	4	2	1=<18.5;2=18.5-<24;3=24-<28;4= ≥ 28 4 * K.Final diagnosis: cerebral infarction; Etiology according to TOAST system; 1-large artery atherosclerosis; 2-cardiogenic embolism; 3-small artery occlusion; 4-stroke of another determined cause; 5-stroke of an undete
BMI_g*IMG_C_TOAST	3	5	1=<18.5;2=18.5-<24;3=24-<28;4= ≥ 28 3 * K.Final diagnosis: cerebral infarction; Etiology according to TOAST system; 1-large artery atherosclerosis; 2-cardiogenic embolism; 3-small artery occlusion; 4-stroke of another determined cause; 5-stroke of an undete
BMI_g*IMG_C_TOAST	3	4	1=<18.5;2=18.5-<24;3=24-<28;4= ≥ 28 3 * K.Final diagnosis: cerebral infarction; Etiology according to TOAST system; 1-large artery atherosclerosis; 2-cardiogenic embolism; 3-small artery occlusion; 4-stroke of another determined cause; 5-stroke of an undete
BMI_g*IMG_C_TOAST	3	3	1=<18.5;2=18.5-<24;3=24-<28;4= ≥ 28 3 * K.Final diagnosis: cerebral infarction; Etiology according to TOAST system; 1-large artery atherosclerosis; 2-cardiogenic embolism; 3-small artery occlusion; 4-stroke of another determined cause; 5-stroke of an undete
BMI_g*IMG_C_TOAST	3	2	1=<18.5;2=18.5-<24;3=24-<28;4= ≥ 28 3 * K.Final diagnosis: cerebral infarction; Etiology according to TOAST system; 1-large artery atherosclerosis; 2-cardiogenic embolism; 3-small artery occlusion; 4-stroke of another determined cause; 5-stroke of an undete
BMI_g*IMG_C_TOAST	1	5	1=<18.5;2=18.5-<24;3=24-<28;4= ≥ 28 1 * K.Final diagnosis: cerebral infarction; Etiology according to TOAST system; 1-large artery atherosclerosis; 2-cardiogenic embolism; 3-small artery occlusion; 4-stroke of another determined cause; 5-stroke of an undete
BMI_g*IMG_C_TOAST	1	4	1=<18.5;2=18.5-<24;3=24-<28;4= ≥ 28 1 * K.Final diagnosis: cerebral infarction; Etiology according to TOAST system; 1-large artery atherosclerosis; 2-cardiogenic embolism; 3-small artery occlusion; 4-stroke of another determined cause; 5-stroke of an undete
BMI_g*IMG_C_TOAST	1	3	1=<18.5;2=18.5-<24;3=24-<28;4= ≥ 28 1 * K.Final diagnosis: cerebral infarction; Etiology according to TOAST system; 1-large artery atherosclerosis; 2-cardiogenic embolism; 3-small artery occlusion; 4-stroke of another determined cause; 5-stroke of an undete
BMI_g*IMG_C_TOAST	1	2	1=<18.5;2=18.5-<24;3=24-<28;4= ≥ 28 1 * K.Final diagnosis: cerebral infarction; Etiology according to TOAST system; 1-large artery atherosclerosis; 2-cardiogenic embolism; 3-small artery occlusion; 4-stroke of another determined cause; 5-stroke of an undete
AGE			A.Basic Information: Age (years old);
GENDER	2		A.Basic Information: Gender; 1-male; 2-female; 2
ETHNIC	2		B.Demography: Race: 1-Han; 99-others; 2
H_DIAB01	1		D.History: Diabetes; 0-No; 1-Yes; 1
H_AF01	1		D.History: Heart disease category: Atrial fibrillation(Including medical history and hospitalization diagnosis); 0-No; 1-Yes; 1
H_HYPT01	1		D.History: Hypertension; 0-No; 1-Yes; 1

BMI_g with y1_death: interaction with stroke subtype

PHREG 过程

最大似然估计分析										
参数			自由度	参数估计	标准误差	卡方	Pr > 卡方	危险率	95% 危险率置信限	
H_LIPID01	1		1	-0.31254	0.20813	2.2549	0.1332	0.732	0.487	1.100
AI	1		1	0.51947	0.22745	5.2160	0.0224	1.681	1.076	2.626
H_DRINK_H01	1		1	-0.52240	0.19588	7.1127	0.0077	0.593	0.404	0.871
H_SMK_C01	1		1	0.12965	0.12439	1.0863	0.2973	1.138	0.892	1.453
IT	1		1	-0.73200	0.16640	19.3504	<.0001	0.481	0.347	0.666
ET	1		1	0.89261	0.31433	8.0643	0.0045	2.442	1.319	4.521
A_NIHSS			1	0.09316	0.00630	218.5801	<.0001	1.098	1.084	1.111

BMI_g with y1_death: interaction with stroke subtype

PHREG 过程

最大似然估计分析			
参数			标签
H_LIPID01	1		D.History: Lipid metabolism disorders; 0-No; 1-Yes; 1
AI	1		history:Myocardial infarction; 0=NO; 1=YES; 1
H_DRINK_H01	1		D.History: Heavy Drinking(Alcohol consumption>=20g/day); 0-No,1-Yes; 1
H_SMK_C01	1		D.History: Current Smoking; 0-No,1-Yes; 1
IT	1		intravenous thrombolysis, 1=YES,0=NO 1
ET	1		动脉溶栓或机械取栓, 1=YES,0=NO 1
A_NIHSS			F.Admitting NIHSS: Total score;

BMI_g with y1_comb: Descriptive results

FREQ 过程

频数<br>行百分比

BMI_g-y1_comb表			
	y1_comb(N12.Follow-up events at 12 months:Occurrence of combined vascular event(including cardiovascular death,non-fatal stroke,non-fatal myocardial infarction):0-No;1-Yes;)		
BMI_g(1=<18.5;2=18.5-<24;3=24-<28;4=≥28)	0	1	合计
1	270<br>87.38	39<br>12.62	309
2	5083<br>89.52	595<br>10.48	5678
3	5558<br>89.43	657<br>10.57	6215
4	1730<br>88.99	214<br>11.01	1944
合计	12641	1505	14146

表“y1_comb-BMI_g”的统计量

统计量	自由度	值	概率
卡方	3	1.7389	0.6283
似然比卡方检验	3	1.6729	0.6430
Mantel-Haenszel 卡方	1	0.0225	0.8807
Phi 系数		0.0111	
列联系数		0.0111	
Cramer V		0.0111	

样本大小 = 14146

BMI_g with y1_comb: crude model

PHREG 过程

模型信息		
数据集	WORK.DATA2	
因变量	y1_comb_dd	N12.Follow-up events at 12 months: Days from onset to occurrence of combined vascular event;(day);
删失变量	y1_comb	N12.Follow-up events at 12 months:Occurrence of combined vascular event(including cardiovascular death,non-fatal stroke,non-fatal myocardial infarction):0-No;1-Yes;
删失值	0	
结值处理	BRESLOW	

读取的观测数	14146
使用的观测数	14146

分类水平信息				
分类	值	设计变量		
BMI_g	4	1	0	0
	3	0	1	0
	2	0	0	0
	1	0	0	1

事件和删失值个数汇总			
合计	事件	删失	删失百分比
14146	1505	12641	89.36

收敛状态
满足收敛准则 (GCONV=1E-8)。

模型拟合统计量		
准则	无协变量	带协变量
-2 LOG L	28553.433	28551.337
AIC	28553.433	28557.337
SBC	28553.433	28573.287

检验全局原假设: BETA=0			
检验	卡方	自由度	Pr > 卡方
似然比	2.0958	3	0.5528
评分	2.2210	3	0.5278
Wald	2.2186	3	0.5283

3 型检验			
效应	自由度	Wald 卡方	Pr > 卡方
BMI_g	3	2.2186	0.5283

BMI_g with y1_comb: crude model

PHREG 过程

最大似然估计分析										
参数		自由度	参数估计	标准误差	卡方	Pr > 卡方	危险率	95% 危险率置信限		标签
BMI_g	4	1	0.05205	0.07971	0.4265	0.5137	1.053	0.901	1.232	1=<18.5;2=18.5-<24;3=24-<28;4= ≥ 28 4
BMI_g	3	1	0.00798	0.05659	0.0199	0.8879	1.008	0.902	1.126	1=<18.5;2=18.5-<24;3=24-<28;4= ≥ 28 3
BMI_g	1	1	0.22761	0.16527	1.8966	0.1685	1.256	0.908	1.736	1=<18.5;2=18.5-<24;3=24-<28;4= ≥ 28 1

BMI_g with y1_comb: adjusted model

PHREG 过程

模型信息		
数据集	WORK.DATA2	
因变量	y1_comb_dd	N12.Follow-up events at 12 months: Days from onset to occurrence of combined vascular event;(day);
删失变量	y1_comb	N12.Follow-up events at 12 months:Occurrence of combined vascular event(including cardiovascular death,non-fatal stroke,non-fatal myocardial infarction):0-No;1-Yes;
删失值	0	
结值处理	BRESLOW	

读取的观测数	14146
使用的观测数	14146

分类水平信息					
分类	值	设计变量			
BMI_g	4	1	0	0	
	3	0	1	0	
	2	0	0	0	
	1	0	0	1	
GENDER	2	1			
	1	0			
ETHNIC	2	1			
	1	0			
H_DIAB01	1	1			
	0	0			
H_AF01	1	1			
	0	0			
H_HYPT01	1	1			
	0	0			
H_LIPID01	1	1			
	0	0			
AI	1	1			
	0	0			
H_DRINK_H01	1	1			
	0	0			
H_SMK_C01	1	1			
	0	0			
IT	1	1			
	0	0			
ET	1	1			
	0	0			
IMG_C_TOAST	5	1	0	0	0
	4	0	1	0	0
	3	0	0	1	0

BMI_g with y1_comb: adjusted model

PHREG 过程

分类水平信息					
分类	值	设计变量			
	2	0	0	0	1
	1	0	0	0	0

事件和删失值个数汇总			
合计	事件	删失	删失百分比
14146	1505	12641	89.36

收敛状态
满足收敛准则 (GCONV=1E-8)。

模型拟合统计量		
准则	无协变量	带协变量
-2 LOG L	28553.433	28381.596
AIC	28553.433	28421.596
SBC	28553.433	28527.927

检验全局原假设: BETA=0			
检验	卡方	自由度	Pr > 卡方
似然比	171.8365	20	<.0001
评分	183.1530	20	<.0001
Wald	179.0204	20	<.0001

3 型检验			
效应	自由度	Wald 卡方	Pr > 卡方
BMI_g	3	1.6189	0.6551
AGE	1	19.5219	<.0001
GENDER	1	0.1693	0.6808
ETHNIC	1	0.4324	0.5108
H_DIAB01	1	14.1447	0.0002
H_AF01	1	13.4477	0.0002
H_HYPT01	1	2.6956	0.1006
H_LIPID01	1	0.6487	0.4206
AI	1	2.1366	0.1438
H_DRINK_H01	1	1.3082	0.2527
H_SMK_C01	1	0.3039	0.5814
IT	1	0.2295	0.6319
ET	1	6.0622	0.0138
IMG_C_TOAST	4	54.2725	<.0001
A_NIHSS	1	16.0875	<.0001

BMI_g with y1_comb: adjusted model

PHREG 过程

最大似然估计分析									
参数		自由度	参数估计	标准误差	卡方	Pr > 卡方	危险率	95% 危险率置信限	
BMI_g	4	1	0.08789	0.08147	1.1638	0.2807	1.092	0.931	1.281
BMI_g	3	1	0.03642	0.05737	0.4030	0.5255	1.037	0.927	1.161
BMI_g	1	1	0.12849	0.16633	0.5968	0.4398	1.137	0.821	1.575
AGE		1	0.01104	0.00250	19.5219	<.0001	1.011	1.006	1.016
GENDER	2	1	0.02503	0.06084	0.1693	0.6808	1.025	0.910	1.155
ETHNIC	2	1	-0.10562	0.16061	0.4324	0.5108	0.900	0.657	1.233
H_DIAB01	1	1	0.22183	0.05898	14.1447	0.0002	1.248	1.112	1.401
H_AF01	1	1	0.42404	0.11563	13.4477	0.0002	1.528	1.218	1.917
H_HYPT01	1	1	0.09168	0.05584	2.6956	0.1006	1.096	0.982	1.223
H_LIPID01	1	1	-0.08033	0.09974	0.6487	0.4206	0.923	0.759	1.122
AI	1	1	0.23085	0.15793	2.1366	0.1438	1.260	0.924	1.717
H_DRINK_H01	1	1	0.09424	0.08240	1.3082	0.2527	1.099	0.935	1.291
H_SMK_C01	1	1	-0.03707	0.06725	0.3039	0.5814	0.964	0.845	1.099
IT	1	1	0.03917	0.08178	0.2295	0.6319	1.040	0.886	1.221
ET	1	1	0.61487	0.24973	6.0622	0.0138	1.849	1.134	3.017
IMG_C_TOAST	5	1	-0.32878	0.06150	28.5828	<.0001	0.720	0.638	0.812
IMG_C_TOAST	4	1	-0.02463	0.22329	0.0122	0.9122	0.976	0.630	1.511
IMG_C_TOAST	3	1	-0.52858	0.07915	44.5960	<.0001	0.589	0.505	0.688
IMG_C_TOAST	2	1	-0.42488	0.13555	9.8254	0.0017	0.654	0.501	0.853
A_NIHSS		1	0.02291	0.00571	16.0875	<.0001	1.023	1.012	1.035

BMI_g with y1_comb: adjusted model

PHREG 过程

最大似然估计分析		
参数		标签
BMI_g	4	1=<18.5;2=18.5-<24;3=24-<28;4= ≥ 28 4
BMI_g	3	1=<18.5;2=18.5-<24;3=24-<28;4= ≥ 28 3
BMI_g	1	1=<18.5;2=18.5-<24;3=24-<28;4= ≥ 28 1
AGE		A.Basic Information: Age (years old);
GENDER	2	A.Basic Information: Gender; 1-male; 2-female; 2
ETHNIC	2	B.Demography: Race: 1-Han; 99-others; 2
H_DIAB01	1	D.History: Diabetes; 0-No; 1-Yes; 1
H_AF01	1	D.History: Heart disease category: Atrial fibrillation(Including medical history and hospitalization diagnosis); 0-No; 1-Yes; 1
H_HYPT01	1	D.History: Hypertension; 0-No; 1-Yes; 1
H_LIPID01	1	D.History: Lipid metabolism disorders; 0-No; 1-Yes; 1
AI	1	history:Myocardial infarction; 0=NO; 1=YES; 1
H_DRINK_H01	1	D.History: Heavy Drinking(Alcohol consumption>=20g/day); 0-No,1-Yes; 1
H_SMK_C01	1	D.History: Current Smoking; 0-No,1-Yes; 1
IT	1	intravenous thrombolysis, 1=YES,0=NO 1
ET	1	动脉溶栓或机械取栓, 1=YES,0=NO 1
IMG_C_TOAST	5	K.Final diagnosis: cerebral infarction; Etiology according to TOAST system; 1-large artery atherosclerosis; 2-cardiogenic embolism; 3-small artery occlusion; 4-stroke of another determined cause; 5-stroke of an undetermined cause. 5
IMG_C_TOAST	4	K.Final diagnosis: cerebral infarction; Etiology according to TOAST system; 1-large artery atherosclerosis; 2-cardiogenic embolism; 3-small artery occlusion; 4-stroke of another determined cause; 5-stroke of an undetermined cause. 4
IMG_C_TOAST	3	K.Final diagnosis: cerebral infarction; Etiology according to TOAST system; 1-large artery atherosclerosis; 2-cardiogenic embolism; 3-small artery occlusion; 4-stroke of another determined cause; 5-stroke of an undetermined cause. 3
IMG_C_TOAST	2	K.Final diagnosis: cerebral infarction; Etiology according to TOAST system; 1-large artery atherosclerosis; 2-cardiogenic embolism; 3-small artery occlusion; 4-stroke of another determined cause; 5-stroke of an undetermined cause. 2
A_NIHSS		F.Admitting NIHSS: Total score;

BMI_g with y1_comb: interaction with stroke subtype

PHREG 过程

模型信息		
数据集	WORK.DATA2	
因变量	y1_comb_dd	N12.Follow-up events at 12 months: Days from onset to occurrence of combined vascular event;(day);
删失变量	y1_comb	N12.Follow-up events at 12 months:Occurrence of combined vascular event(including cardiovascular death,non-fatal stroke,non-fatal myocardial infarction):0-No;1-Yes;
删失值	0	
结值处理	BRESLOW	

读取的观测数	14146
使用的观测数	14146

分类水平信息					
分类	值	设计变量			
BMI_g	4	1	0	0	
	3	0	1	0	
	2	0	0	0	
	1	0	0	1	
GENDER	2	1			
	1	0			
ETHNIC	2	1			
	1	0			
H_DIAB01	1	1			
	0	0			
H_AF01	1	1			
	0	0			
H_HYPT01	1	1			
	0	0			
H_LIPID01	1	1			
	0	0			
AI	1	1			
	0	0			
H_DRINK_H01	1	1			
	0	0			
H_SMK_C01	1	1			
	0	0			
IT	1	1			
	0	0			
ET	1	1			
	0	0			
IMG_C_TOAST	5	1	0	0	0
	4	0	1	0	0
	3	0	0	1	0

BMI_g with y1_comb: interaction with stroke subtype

PHREG 过程

分类水平信息					
分类	值	设计变量			
	2	0	0	0	1
	1	0	0	0	0

事件和删失值个数汇总			
合计	事件	删失	删失百分比
14146	1505	12641	89.36

收敛状态
满足收敛准则 (GCONV=1E-8)。

模型拟合统计量		
准则	无协变量	带协变量
-2 LOG L	28553.433	28368.483
AIC	28553.433	28432.483
SBC	28553.433	28602.613

检验全局原假设: BETA=0			
检验	卡方	自由度	Pr > 卡方
似然比	184.9496	32	<.0001
评分	197.3650	32	<.0001
Wald	192.5036	32	<.0001

联合检验			
效应	自由度	Wald 卡方	Pr > 卡方
BMI_g	3	0.7224	0.8679
IMG_C_TOAST	4	27.4794	<.0001
BMI_g*IMG_C_TOAST	12	13.3188	0.3463
AGE	1	19.7169	<.0001
GENDER	1	0.1761	0.6748
ETHNIC	1	0.4024	0.5258
H_DIAB01	1	13.7271	0.0002
H_AF01	1	13.9878	0.0002
H_HYPT01	1	2.7549	0.0970
H_LIPID01	1	0.6059	0.4363
AI	1	2.0238	0.1548
H_DRINK_H01	1	1.2808	0.2578
H_SMK_C01	1	0.3269	0.5675
IT	1	0.2460	0.6199

BMI_g with y1_comb: interaction with stroke subtype

PHREG 过程

联合检验			
效应	自由度	Wald 卡方	Pr > 卡方
ET	1	6.3966	0.0114
A_NIHSS	1	16.0247	<.0001

Note: Under full-rank parameterizations, Type 3 effect tests are replaced by joint tests. The joint test for an effect is a test that all of the parameters associated with that effect are zero. Such joint tests might not be equivalent to Type 3 effect tests under GLM parameterization.

BMI_g with y1_comb: interaction with stroke subtype

PHREG 过程

最大似然估计分析										
参数			自由度	参数估计	标准误差	卡方	Pr > 卡方	危险率	95% 危险率置信限	
BMI_g	4		1	-0.03686	0.14341	0.0661	0.7972	.	.	.
BMI_g	3		1	0.06430	0.09837	0.4273	0.5133	.	.	.
BMI_g	1		1	0.04585	0.31032	0.0218	0.8825	.	.	.
IMG_C_TOAST	5		1	-0.34175	0.09653	12.5348	0.0004	.	.	.
IMG_C_TOAST	4		1	-0.28694	0.38505	0.5553	0.4561	.	.	.
IMG_C_TOAST	3		1	-0.65152	0.13169	24.4758	<.0001	.	.	.
IMG_C_TOAST	2		1	-0.21092	0.17112	1.5193	0.2177	.	.	.
BMI_g*IMG_C_TOAST	4	5	1	0.21776	0.18754	1.3483	0.2456	.	.	.
BMI_g*IMG_C_TOAST	4	4	1	0.56010	0.64289	0.7590	0.3836	.	.	.
BMI_g*IMG_C_TOAST	4	3	1	0.22976	0.24524	0.8778	0.3488	.	.	.
BMI_g*IMG_C_TOAST	4	2	1	-0.08954	0.31643	0.0801	0.7772	.	.	.
BMI_g*IMG_C_TOAST	3	5	1	-0.04023	0.13146	0.0937	0.7596	.	.	.
BMI_g*IMG_C_TOAST	3	4	1	0.34199	0.51352	0.4435	0.5054	.	.	.
BMI_g*IMG_C_TOAST	3	3	1	0.15057	0.17396	0.7491	0.3867	.	.	.
BMI_g*IMG_C_TOAST	3	2	1	-0.51373	0.23159	4.9210	0.0265	.	.	.
BMI_g*IMG_C_TOAST	1	5	1	-0.01852	0.40351	0.0021	0.9634	.	.	.
BMI_g*IMG_C_TOAST	1	4	1	0.84925	1.11416	0.5810	0.4459	.	.	.
BMI_g*IMG_C_TOAST	1	3	1	0.78761	0.48340	2.6547	0.1032	.	.	.
BMI_g*IMG_C_TOAST	1	2	1	-0.51561	0.66864	0.5946	0.4406	.	.	.
AGE			1	0.01109	0.00250	19.7169	<.0001	1.011	1.006	1.016
GENDER	2		1	0.02554	0.06087	0.1761	0.6748	1.026	0.911	1.156
ETHNIC	2		1	-0.10200	0.16080	0.4024	0.5258	0.903	0.659	1.238
H_DIAB01	1		1	0.21874	0.05904	13.7271	0.0002	1.245	1.109	1.397
H_AF01	1		1	0.43148	0.11537	13.9878	0.0002	1.540	1.228	1.930
H_HYPT01	1		1	0.09276	0.05589	2.7549	0.0970	1.097	0.983	1.224

BMI_g with y1_comb: interaction with stroke subtype

PHREG 过程

最大似然估计分析			
参数			标签
BMI_g	4		1=<18.5;2=18.5-<24;3=24-<28;4= ≥ 28 4
BMI_g	3		1=<18.5;2=18.5-<24;3=24-<28;4= ≥ 28 3
BMI_g	1		1=<18.5;2=18.5-<24;3=24-<28;4= ≥ 28 1
IMG_C_TOAST	5		K.Final diagnosis: cerebral infarction; Etiology according to TOAST system; 1-large artery atherosclerosis; 2-cardiogenic embolism; 3-small artery occlusion; 4-stroke of another determined cause; 5-stroke of an undetermined cause. 5
IMG_C_TOAST	4		K.Final diagnosis: cerebral infarction; Etiology according to TOAST system; 1-large artery atherosclerosis; 2-cardiogenic embolism; 3-small artery occlusion; 4-stroke of another determined cause; 5-stroke of an undetermined cause. 4
IMG_C_TOAST	3		K.Final diagnosis: cerebral infarction; Etiology according to TOAST system; 1-large artery atherosclerosis; 2-cardiogenic embolism; 3-small artery occlusion; 4-stroke of another determined cause; 5-stroke of an undetermined cause. 3
IMG_C_TOAST	2		K.Final diagnosis: cerebral infarction; Etiology according to TOAST system; 1-large artery atherosclerosis; 2-cardiogenic embolism; 3-small artery occlusion; 4-stroke of another determined cause; 5-stroke of an undetermined cause. 2
BMI_g*IMG_C_TOAST	4	5	1=<18.5;2=18.5-<24;3=24-<28;4= ≥ 28 4 * K.Final diagnosis: cerebral infarction; Etiology according to TOAST system; 1-large artery atherosclerosis; 2-cardiogenic embolism; 3-small artery occlusion; 4-stroke of another determined cause; 5-stroke of an undete
BMI_g*IMG_C_TOAST	4	4	1=<18.5;2=18.5-<24;3=24-<28;4= ≥ 28 4 * K.Final diagnosis: cerebral infarction; Etiology according to TOAST system; 1-large artery atherosclerosis; 2-cardiogenic embolism; 3-small artery occlusion; 4-stroke of another determined cause; 5-stroke of an undete
BMI_g*IMG_C_TOAST	4	3	1=<18.5;2=18.5-<24;3=24-<28;4= ≥ 28 4 * K.Final diagnosis: cerebral infarction; Etiology according to TOAST system; 1-large artery atherosclerosis; 2-cardiogenic embolism; 3-small artery occlusion; 4-stroke of another determined cause; 5-stroke of an undete
BMI_g*IMG_C_TOAST	4	2	1=<18.5;2=18.5-<24;3=24-<28;4= ≥ 28 4 * K.Final diagnosis: cerebral infarction; Etiology according to TOAST system; 1-large artery atherosclerosis; 2-cardiogenic embolism; 3-small artery occlusion; 4-stroke of another determined cause; 5-stroke of an undete
BMI_g*IMG_C_TOAST	3	5	1=<18.5;2=18.5-<24;3=24-<28;4= ≥ 28 3 * K.Final diagnosis: cerebral infarction; Etiology according to TOAST system; 1-large artery atherosclerosis; 2-cardiogenic embolism; 3-small artery occlusion; 4-stroke of another determined cause; 5-stroke of an undete
BMI_g*IMG_C_TOAST	3	4	1=<18.5;2=18.5-<24;3=24-<28;4= ≥ 28 3 * K.Final diagnosis: cerebral infarction; Etiology according to TOAST system; 1-large artery atherosclerosis; 2-cardiogenic embolism; 3-small artery occlusion; 4-stroke of another determined cause; 5-stroke of an undete
BMI_g*IMG_C_TOAST	3	3	1=<18.5;2=18.5-<24;3=24-<28;4= ≥ 28 3 * K.Final diagnosis: cerebral infarction; Etiology according to TOAST system; 1-large artery atherosclerosis; 2-cardiogenic embolism; 3-small artery occlusion; 4-stroke of another determined cause; 5-stroke of an undete
BMI_g*IMG_C_TOAST	3	2	1=<18.5;2=18.5-<24;3=24-<28;4= ≥ 28 3 * K.Final diagnosis: cerebral infarction; Etiology according to TOAST system; 1-large artery atherosclerosis; 2-cardiogenic embolism; 3-small artery occlusion; 4-stroke of another determined cause; 5-stroke of an undete
BMI_g*IMG_C_TOAST	1	5	1=<18.5;2=18.5-<24;3=24-<28;4= ≥ 28 1 * K.Final diagnosis: cerebral infarction; Etiology according to TOAST system; 1-large artery atherosclerosis; 2-cardiogenic embolism; 3-small artery occlusion; 4-stroke of another determined cause; 5-stroke of an undete
BMI_g*IMG_C_TOAST	1	4	1=<18.5;2=18.5-<24;3=24-<28;4= ≥ 28 1 * K.Final diagnosis: cerebral infarction; Etiology according to TOAST system; 1-large artery atherosclerosis; 2-cardiogenic embolism; 3-small artery occlusion; 4-stroke of another determined cause; 5-stroke of an undete
BMI_g*IMG_C_TOAST	1	3	1=<18.5;2=18.5-<24;3=24-<28;4= ≥ 28 1 * K.Final diagnosis: cerebral infarction; Etiology according to TOAST system; 1-large artery atherosclerosis; 2-cardiogenic embolism; 3-small artery occlusion; 4-stroke of another determined cause; 5-stroke of an undete
BMI_g*IMG_C_TOAST	1	2	1=<18.5;2=18.5-<24;3=24-<28;4= ≥ 28 1 * K.Final diagnosis: cerebral infarction; Etiology according to TOAST system; 1-large artery atherosclerosis; 2-cardiogenic embolism; 3-small artery occlusion; 4-stroke of another determined cause; 5-stroke of an undete
AGE			A.Basic Information: Age (years old);
GENDER	2		A.Basic Information: Gender; 1-male; 2-female; 2
ETHNIC	2		B.Demography: Race: 1-Han; 99-others; 2
H_DIAB01	1		D.History: Diabetes; 0-No; 1-Yes; 1
H_AF01	1		D.History: Heart disease category: Atrial fibrillation(Including medical history and hospitalization diagnosis); 0-No; 1-Yes; 1
H_HYPT01	1		D.History: Hypertension; 0-No; 1-Yes; 1

BMI_g with y1_comb: interaction with stroke subtype

PHREG 过程

最大似然估计分析										
参数			自由度	参数<br>估计	标准<br>误差	卡方	Pr > 卡方	危险<br>率	95%<br>危险率置信限	
H_LIPID01	1		1	-0.07766	0.09977	0.6059	0.4363	0.925	0.761	1.125
AI	1		1	0.22482	0.15803	2.0238	0.1548	1.252	0.919	1.707
H_DRINK_H01	1		1	0.09331	0.08245	1.2808	0.2578	1.098	0.934	1.290
H_SMK_C01	1		1	-0.03848	0.06730	0.3269	0.5675	0.962	0.843	1.098
IT	1		1	0.04059	0.08185	0.2460	0.6199	1.041	0.887	1.223
ET	1		1	0.63229	0.25000	6.3966	0.0114	1.882	1.153	3.072
A_NIHSS			1	0.02281	0.00570	16.0247	<.0001	1.023	1.012	1.035

BMI_g with y1_comb: interaction with stroke subtype

PHREG 过程

最大似然估计分析			
参数			标签
H_LIPID01	1		D.History: Lipid metabolism disorders; 0-No; 1-Yes; 1
AI	1		history:Myocardial infarction; 0=NO; 1=YES; 1
H_DRINK_H01	1		D.History: Heavy Drinking(Alcohol consumption>=20g/day); 0-No,1-Yes; 1
H_SMK_C01	1		D.History: Current Smoking; 0-No,1-Yes; 1
IT	1		intravenous thrombolysis, 1=YES,0=NO 1
ET	1		动脉溶栓或机械取栓, 1=YES,0=NO 1
A_NIHSS			F.Admitting NIHSS: Total score;

BMI_g with y1_stroke: Descriptive results

FREQ 过程

频数<br>行百分比

BMI_g-y1_stroke表

BMI_g(1=<18.5;2=18.5-<24;3=24-<28;4= ≥ 28)	y1_stroke(N12.Follow-up events at 12 months: Recurrence of stroke: 0-No; 1-Yes;) 0	1	合计
1	273<br>88.35	36<br>11.65	309
2	5121<br>90.19	557<br>9.81	5678
3	5591<br>89.96	624<br>10.04	6215
4	1737<br>89.35	207<br>10.65	1944
合计	12722	1424	14146

表“y1_stroke-BMI_g”的统计量

统计量	自由度	值	概率
卡方	3	2.0009	0.5722
似然比卡方检验	3	1.9550	0.5818
Mantel-Haenszel 卡方	1	0.3804	0.5374
Phi 系数		0.0119	
列联系数		0.0119	
Cramer V		0.0119	

样本大小 = 14146

BMI_g with y1_stroke: crude model

PHREG 过程

模型信息		
数据集	WORK.DATA2	
因变量	y1_stroke_dd	N12.Follow-up events at 12 months: Days from onset to recurrence;(day);
删失变量	y1_stroke	N12.Follow-up events at 12 months: Recurrence of stroke: 0-No; 1-Yes;
删失值	0	
结值处理	BRESLOW	

读取的观测数	14146
使用的观测数	14146

分类水平信息				
分类	值	设计变量		
BMI_g	4	1	0	0
	3	0	1	0
	2	0	0	0
	1	0	0	1

事件和删失值个数汇总			
合计	事件	删失	删失 百分比
14146	1424	12722	89.93

收敛状态
满足收敛准则 (GCONV=1E-8)。

模型拟合统计量		
准则	无 协变量	带 协变量
-2 LOG L	27024.892	27022.626
AIC	27024.892	27028.626
SBC	27024.892	27044.409

检验全局原假设: BETA=0			
检验	卡方	自由度	Pr > 卡方
似然比	2.2666	3	0.5190
评分	2.3523	3	0.5026
Wald	2.3498	3	0.5030

3 型检验			
效应	自由度	Wald 卡方	Pr > 卡方
BMI_g	3	2.3498	0.5030

BMI_g with y1_stroke: crude model

PHREG 过程

最大似然估计分析										
参数		自由度	参数估计	标准误差	卡方	Pr > 卡方	危险率	95% 危险率置信限		标签
BMI_g	4	1	0.08538	0.08140	1.1000	0.2943	1.089	0.929	1.278	1=<18.5;2=18.5-<24;3=24-<28;4= ≥ 28 4
BMI_g	3	1	0.02273	0.05829	0.1520	0.6966	1.023	0.913	1.147	1=<18.5;2=18.5-<24;3=24-<28;4= ≥ 28 3
BMI_g	1	1	0.21166	0.17196	1.5151	0.2184	1.236	0.882	1.731	1=<18.5;2=18.5-<24;3=24-<28;4= ≥ 28 1

BMI_g with y1_stroke: adjusted model

PHREG 过程

模型信息		
数据集	WORK.DATA2	
因变量	y1_stroke_dd	N12.Follow-up events at 12 months: Days from onset to recurrence;(day);
删失变量	y1_stroke	N12.Follow-up events at 12 months: Recurrence of stroke: 0-No; 1-Yes;
删失值	0	
结值处理	BRESLOW	

读取的观测数	14146
使用的观测数	14146

分类水平信息					
分类	值	设计变量			
BMI_g	4	1	0	0	
	3	0	1	0	
	2	0	0	0	
	1	0	0	1	
GENDER	2	1			
	1	0			
ETHNIC	2	1			
	1	0			
H_DIAB01	1	1			
	0	0			
H_AF01	1	1			
	0	0			
H_HYPT01	1	1			
	0	0			
H_LIPID01	1	1			
	0	0			
AI	1	1			
	0	0			
H_DRINK_H01	1	1			
	0	0			
H_SMK_C01	1	1			
	0	0			
IT	1	1			
	0	0			
ET	1	1			
	0	0			
IMG_C_TOAST	5	1	0	0	0
	4	0	1	0	0
	3	0	0	1	0

BMI_g with y1_stroke: adjusted model

PHREG 过程

分类水平信息					
分类	值	设计变量			
	2	0	0	0	1
	1	0	0	0	0

事件和删失值个数汇总			
合计	事件	删失	删失百分比
14146	1424	12722	89.93

收敛状态
满足收敛准则 (GCONV=1E-8)。

模型拟合统计量		
准则	无协变量	带协变量
-2 LOG L	27024.892	26877.831
AIC	27024.892	26917.831
SBC	27024.892	27023.056

检验全局原假设: BETA=0			
检验	卡方	自由度	Pr > 卡方
似然比	147.0608	20	<.0001
评分	155.6643	20	<.0001
Wald	152.4330	20	<.0001

3 型检验			
效应	自由度	Wald 卡方	Pr > 卡方
BMI_g	3	2.4561	0.4833
AGE	1	15.0644	0.0001
GENDER	1	0.4636	0.4959
ETHNIC	1	0.6017	0.4379
H_DIAB01	1	11.8783	0.0006
H_AF01	1	8.5008	0.0035
H_HYPT01	1	1.2905	0.2560
H_LIPID01	1	0.6091	0.4351
AI	1	1.0183	0.3129
H_DRINK_H01	1	3.0259	0.0819
H_SMK_C01	1	0.7585	0.3838
IT	1	1.0582	0.3036
ET	1	5.9366	0.0148
IMG_C_TOAST	4	52.6711	<.0001
A_NIHSS	1	12.8160	0.0003

BMI_g with y1_stroke: adjusted model

PHREG 过程

最大似然估计分析									
参数		自由度	参数估计	标准误差	卡方	Pr > 卡方	危险率	95% 危险率置信限	
BMI_g	4	1	0.12067	0.08322	2.1022	0.1471	1.128	0.958	1.328
BMI_g	3	1	0.05109	0.05909	0.7476	0.3872	1.052	0.937	1.182
BMI_g	1	1	0.12048	0.17300	0.4850	0.4862	1.128	0.804	1.583
AGE		1	0.00993	0.00256	15.0644	0.0001	1.010	1.005	1.015
GENDER	2	1	0.04262	0.06259	0.4636	0.4959	1.044	0.923	1.180
ETHNIC	2	1	-0.12948	0.16693	0.6017	0.4379	0.879	0.633	1.219
H_DIAB01	1	1	0.20951	0.06079	11.8783	0.0006	1.233	1.095	1.389
H_AF01	1	1	0.35569	0.12200	8.5008	0.0035	1.427	1.124	1.813
H_HYPT01	1	1	0.06498	0.05720	1.2905	0.2560	1.067	0.954	1.194
H_LIPID01	1	1	-0.08003	0.10254	0.6091	0.4351	0.923	0.755	1.129
AI	1	1	0.16954	0.16801	1.0183	0.3129	1.185	0.852	1.647
H_DRINK_H01	1	1	0.14565	0.08373	3.0259	0.0819	1.157	0.982	1.363
H_SMK_C01	1	1	-0.06030	0.06923	0.7585	0.3838	0.941	0.822	1.078
IT	1	1	0.08545	0.08306	1.0582	0.3036	1.089	0.926	1.282
ET	1	1	0.62726	0.25744	5.9366	0.0148	1.872	1.131	3.101
IMG_C_TOAST	5	1	-0.33625	0.06300	28.4906	<.0001	0.714	0.631	0.808
IMG_C_TOAST	4	1	-0.03513	0.22880	0.0236	0.8780	0.965	0.617	1.512
IMG_C_TOAST	3	1	-0.52779	0.08089	42.5715	<.0001	0.590	0.503	0.691
IMG_C_TOAST	2	1	-0.44750	0.14212	9.9148	0.0016	0.639	0.484	0.845
A_NIHSS		1	0.02125	0.00594	12.8160	0.0003	1.021	1.010	1.033

BMI_g with y1_stroke: adjusted model

PHREG 过程

最大似然估计分析		
参数		标签
BMI_g	4	1=<18.5;2=18.5-<24;3=24-<28;4=≥28 4
BMI_g	3	1=<18.5;2=18.5-<24;3=24-<28;4=≥28 3
BMI_g	1	1=<18.5;2=18.5-<24;3=24-<28;4=≥28 1
AGE		A.Basic Information: Age (years old);
GENDER	2	A.Basic Information: Gender; 1-male; 2-female; 2
ETHNIC	2	B.Demography: Race: 1-Han; 99-others; 2
H_DIAB01	1	D.History: Diabetes; 0-No; 1-Yes; 1
H_AF01	1	D.History: Heart disease category: Atrial fibrillation(Including medical history and hospitalization diagnosis); 0-No; 1-Yes; 1
H_HYPT01	1	D.History: Hypertension; 0-No; 1-Yes; 1
H_LIPID01	1	D.History: Lipid metabolism disorders; 0-No; 1-Yes; 1
AI	1	history:Myocardial infarction; 0=NO; 1=YES; 1
H_DRINK_H01	1	D.History: Heavy Drinking(Alcohol consumption>=20g/day); 0-No,1-Yes; 1
H_SMK_C01	1	D.History: Current Smoking; 0-No,1-Yes; 1
IT	1	intravenous thrombolysis, 1=YES,0=NO 1
ET	1	动脉溶栓或机械取栓, 1=YES,0=NO 1
IMG_C_TOAST	5	K.Final diagnosis: cerebral infarction; Etiology according to TOAST system; 1-large artery atherosclerosis; 2-cardiogenic embolism; 3-small artery occlusion; 4-stroke of another determined cause; 5-stroke of an undetermined cause. 5
IMG_C_TOAST	4	K.Final diagnosis: cerebral infarction; Etiology according to TOAST system; 1-large artery atherosclerosis; 2-cardiogenic embolism; 3-small artery occlusion; 4-stroke of another determined cause; 5-stroke of an undetermined cause. 4
IMG_C_TOAST	3	K.Final diagnosis: cerebral infarction; Etiology according to TOAST system; 1-large artery atherosclerosis; 2-cardiogenic embolism; 3-small artery occlusion; 4-stroke of another determined cause; 5-stroke of an undetermined cause. 3
IMG_C_TOAST	2	K.Final diagnosis: cerebral infarction; Etiology according to TOAST system; 1-large artery atherosclerosis; 2-cardiogenic embolism; 3-small artery occlusion; 4-stroke of another determined cause; 5-stroke of an undetermined cause. 2
A_NIHSS		F.Admitting NIHSS: Total score;

BMI_g with y1_stroke: interaction with stroke subtype

PHREG 过程

模型信息		
数据集	WORK.DATA2	
因变量	y1_stroke_dd	N12.Follow-up events at 12 months: Days from onset to recurrence;(day);
删失变量	y1_stroke	N12.Follow-up events at 12 months: Recurrence of stroke: 0-No; 1-Yes;
删失值	0	
结值处理	BRESLOW	

读取的观测数	14146
使用的观测数	14146

分类水平信息					
分类	值	设计变量			
BMI_g	4	1	0	0	
	3	0	1	0	
	2	0	0	0	
	1	0	0	1	
GENDER	2	1			
	1	0			
ETHNIC	2	1			
	1	0			
H_DIAB01	1	1			
	0	0			
H_AF01	1	1			
	0	0			
H_HYPT01	1	1			
	0	0			
H_LIPID01	1	1			
	0	0			
AI	1	1			
	0	0			
H_DRINK_H01	1	1			
	0	0			
H_SMK_C01	1	1			
	0	0			
IT	1	1			
	0	0			
ET	1	1			
	0	0			
IMG_C_TOAST	5	1	0	0	0
	4	0	1	0	0
	3	0	0	1	0

BMI_g with y1_stroke: interaction with stroke subtype

PHREG 过程

分类水平信息					
分类	值	设计变量			
	2	0	0	0	1
	1	0	0	0	0

事件和删失值个数汇总			
合计	事件	删失	删失百分比
14146	1424	12722	89.93

收敛状态
满足收敛准则 (GCONV=1E-8)。

模型拟合统计量		
准则	无协变量	带协变量
-2 LOG L	27024.892	26864.208
AIC	27024.892	26928.208
SBC	27024.892	27096.567

检验全局原假设: BETA=0			
检验	卡方	自由度	Pr > 卡方
似然比	160.6839	32	<.0001
评分	169.7862	32	<.0001
Wald	165.8050	32	<.0001

联合检验			
效应	自由度	Wald 卡方	Pr > 卡方
BMI_g	3	0.6270	0.8902
IMG_C_TOAST	4	27.7089	<.0001
BMI_g*IMG_C_TOAST	12	13.7693	0.3157
AGE	1	15.1764	<.0001
GENDER	1	0.4820	0.4875
ETHNIC	1	0.5708	0.4499
H_DIAB01	1	11.4650	0.0007
H_AF01	1	8.9549	0.0028
H_HYPT01	1	1.3252	0.2497
H_LIPID01	1	0.5599	0.4543
AI	1	0.9383	0.3327
H_DRINK_H01	1	3.0166	0.0824
H_SMK_C01	1	0.7917	0.3736
IT	1	1.0839	0.2978

BMI_g with y1_stroke: interaction with stroke subtype

PHREG 过程

联合检验			
效应	自由度	Wald 卡方	Pr > 卡方
ET	1	6.2547	0.0124
A_NIHSS	1	12.7927	0.0003

Note: Under full-rank parameterizations, Type 3 effect tests are replaced by joint tests. The joint test for an effect is a test that all of the parameters associated with that effect are zero. Such joint tests might not be equivalent to Type 3 effect tests under GLM parameterization.

BMI_g with y1_stroke: interaction with stroke subtype

PHREG 过程

最大似然估计分析										
参数			自由度	参数估计	标准误差	卡方	Pr > 卡方	危险率	95% 危险率置信限	
BMI_g	4		1	-0.02368	0.14596	0.0263	0.8711	.	.	.
BMI_g	3		1	0.06582	0.10068	0.4274	0.5133	.	.	.
BMI_g	1		1	-0.0008313	0.32503	0.0000	0.9980	.	.	.
IMG_C_TOAST	5		1	-0.35727	0.09919	12.9744	0.0003	.	.	.
IMG_C_TOAST	4		1	-0.40383	0.41515	0.9462	0.3307	.	.	.
IMG_C_TOAST	3		1	-0.67031	0.13555	24.4526	<.0001	.	.	.
IMG_C_TOAST	2		1	-0.24528	0.17985	1.8600	0.1726	.	.	.
BMI_g*IMG_C_TOAST	4	5	1	0.23802	0.19113	1.5508	0.2130	.	.	.
BMI_g*IMG_C_TOAST	4	4	1	0.70719	0.66170	1.1422	0.2852	.	.	.
BMI_g*IMG_C_TOAST	4	3	1	0.28158	0.24827	1.2864	0.2567	.	.	.
BMI_g*IMG_C_TOAST	4	2	1	-0.09511	0.33480	0.0807	0.7764	.	.	.
BMI_g*IMG_C_TOAST	3	5	1	-0.03308	0.13500	0.0600	0.8064	.	.	.
BMI_g*IMG_C_TOAST	3	4	1	0.49697	0.53663	0.8576	0.3544	.	.	.
BMI_g*IMG_C_TOAST	3	3	1	0.16874	0.17854	0.8932	0.3446	.	.	.
BMI_g*IMG_C_TOAST	3	2	1	-0.46541	0.24241	3.6860	0.0549	.	.	.
BMI_g*IMG_C_TOAST	1	5	1	0.03625	0.42026	0.0074	0.9313	.	.	.
BMI_g*IMG_C_TOAST	1	4	1	1.04856	1.12891	0.8627	0.3530	.	.	.
BMI_g*IMG_C_TOAST	1	3	1	0.89334	0.49378	3.2731	0.0704	.	.	.
BMI_g*IMG_C_TOAST	1	2	1	-0.74603	0.79087	0.8898	0.3455	.	.	.
AGE			1	0.00998	0.00256	15.1764	<.0001	1.010	1.005	1.015
GENDER	2		1	0.04348	0.06262	0.4820	0.4875	1.044	0.924	1.181
ETHNIC	2		1	-0.12626	0.16712	0.5708	0.4499	0.881	0.635	1.223
H_DIAB01	1		1	0.20600	0.06084	11.4650	0.0007	1.229	1.091	1.384
H_AF01	1		1	0.36403	0.12165	8.9549	0.0028	1.439	1.134	1.827
H_HYPT01	1		1	0.06590	0.05725	1.3252	0.2497	1.068	0.955	1.195

BMI_g with y1_stroke: interaction with stroke subtype

PHREG 过程

最大似然估计分析			
参数			标签
BMI_g	4		1=<18.5;2=18.5-<24;3=24-<28;4= ≥ 28 4
BMI_g	3		1=<18.5;2=18.5-<24;3=24-<28;4= ≥ 28 3
BMI_g	1		1=<18.5;2=18.5-<24;3=24-<28;4= ≥ 28 1
IMG_C_TOAST	5		K.Final diagnosis: cerebral infarction; Etiology according to TOAST system; 1-large artery atherosclerosis; 2-cardiogenic embolism; 3-small artery occlusion; 4-stroke of another determined cause; 5-stroke of an undetermined cause. 5
IMG_C_TOAST	4		K.Final diagnosis: cerebral infarction; Etiology according to TOAST system; 1-large artery atherosclerosis; 2-cardiogenic embolism; 3-small artery occlusion; 4-stroke of another determined cause; 5-stroke of an undetermined cause. 4
IMG_C_TOAST	3		K.Final diagnosis: cerebral infarction; Etiology according to TOAST system; 1-large artery atherosclerosis; 2-cardiogenic embolism; 3-small artery occlusion; 4-stroke of another determined cause; 5-stroke of an undetermined cause. 3
IMG_C_TOAST	2		K.Final diagnosis: cerebral infarction; Etiology according to TOAST system; 1-large artery atherosclerosis; 2-cardiogenic embolism; 3-small artery occlusion; 4-stroke of another determined cause; 5-stroke of an undetermined cause. 2
BMI_g*IMG_C_TOAST	4	5	1=<18.5;2=18.5-<24;3=24-<28;4= ≥ 28 4 * K.Final diagnosis: cerebral infarction; Etiology according to TOAST system; 1-large artery atherosclerosis; 2-cardiogenic embolism; 3-small artery occlusion; 4-stroke of another determined cause; 5-stroke of an undete
BMI_g*IMG_C_TOAST	4	4	1=<18.5;2=18.5-<24;3=24-<28;4= ≥ 28 4 * K.Final diagnosis: cerebral infarction; Etiology according to TOAST system; 1-large artery atherosclerosis; 2-cardiogenic embolism; 3-small artery occlusion; 4-stroke of another determined cause; 5-stroke of an undete
BMI_g*IMG_C_TOAST	4	3	1=<18.5;2=18.5-<24;3=24-<28;4= ≥ 28 4 * K.Final diagnosis: cerebral infarction; Etiology according to TOAST system; 1-large artery atherosclerosis; 2-cardiogenic embolism; 3-small artery occlusion; 4-stroke of another determined cause; 5-stroke of an undete
BMI_g*IMG_C_TOAST	4	2	1=<18.5;2=18.5-<24;3=24-<28;4= ≥ 28 4 * K.Final diagnosis: cerebral infarction; Etiology according to TOAST system; 1-large artery atherosclerosis; 2-cardiogenic embolism; 3-small artery occlusion; 4-stroke of another determined cause; 5-stroke of an undete
BMI_g*IMG_C_TOAST	3	5	1=<18.5;2=18.5-<24;3=24-<28;4= ≥ 28 3 * K.Final diagnosis: cerebral infarction; Etiology according to TOAST system; 1-large artery atherosclerosis; 2-cardiogenic embolism; 3-small artery occlusion; 4-stroke of another determined cause; 5-stroke of an undete
BMI_g*IMG_C_TOAST	3	4	1=<18.5;2=18.5-<24;3=24-<28;4= ≥ 28 3 * K.Final diagnosis: cerebral infarction; Etiology according to TOAST system; 1-large artery atherosclerosis; 2-cardiogenic embolism; 3-small artery occlusion; 4-stroke of another determined cause; 5-stroke of an undete
BMI_g*IMG_C_TOAST	3	3	1=<18.5;2=18.5-<24;3=24-<28;4= ≥ 28 3 * K.Final diagnosis: cerebral infarction; Etiology according to TOAST system; 1-large artery atherosclerosis; 2-cardiogenic embolism; 3-small artery occlusion; 4-stroke of another determined cause; 5-stroke of an undete
BMI_g*IMG_C_TOAST	3	2	1=<18.5;2=18.5-<24;3=24-<28;4= ≥ 28 3 * K.Final diagnosis: cerebral infarction; Etiology according to TOAST system; 1-large artery atherosclerosis; 2-cardiogenic embolism; 3-small artery occlusion; 4-stroke of another determined cause; 5-stroke of an undete
BMI_g*IMG_C_TOAST	1	5	1=<18.5;2=18.5-<24;3=24-<28;4= ≥ 28 1 * K.Final diagnosis: cerebral infarction; Etiology according to TOAST system; 1-large artery atherosclerosis; 2-cardiogenic embolism; 3-small artery occlusion; 4-stroke of another determined cause; 5-stroke of an undete
BMI_g*IMG_C_TOAST	1	4	1=<18.5;2=18.5-<24;3=24-<28;4= ≥ 28 1 * K.Final diagnosis: cerebral infarction; Etiology according to TOAST system; 1-large artery atherosclerosis; 2-cardiogenic embolism; 3-small artery occlusion; 4-stroke of another determined cause; 5-stroke of an undete
BMI_g*IMG_C_TOAST	1	3	1=<18.5;2=18.5-<24;3=24-<28;4= ≥ 28 1 * K.Final diagnosis: cerebral infarction; Etiology according to TOAST system; 1-large artery atherosclerosis; 2-cardiogenic embolism; 3-small artery occlusion; 4-stroke of another determined cause; 5-stroke of an undete
BMI_g*IMG_C_TOAST	1	2	1=<18.5;2=18.5-<24;3=24-<28;4= ≥ 28 1 * K.Final diagnosis: cerebral infarction; Etiology according to TOAST system; 1-large artery atherosclerosis; 2-cardiogenic embolism; 3-small artery occlusion; 4-stroke of another determined cause; 5-stroke of an undete
AGE			A.Basic Information: Age (years old);
GENDER	2		A.Basic Information: Gender; 1-male; 2-female; 2
ETHNIC	2		B.Demography: Race: 1-Han; 99-others; 2
H_DIAB01	1		D.History: Diabetes; 0-No; 1-Yes; 1
H_AF01	1		D.History: Heart disease category: Atrial fibrillation(Including medical history and hospitalization diagnosis); 0-No; 1-Yes; 1
H_HYPT01	1		D.History: Hypertension; 0-No; 1-Yes; 1

BMI_g with y1_stroke: interaction with stroke subtype

PHREG 过程

最大似然估计分析										
参数			自由度	参数估计	标准误差	卡方	Pr > 卡方	危险率	95% 危险率置信限	
H_LIPID01	1		1	-0.07675	0.10257	0.5599	0.4543	0.926	0.757	1.132
AI	1		1	0.16282	0.16809	0.9383	0.3327	1.177	0.847	1.636
H_DRINK_H01	1		1	0.14551	0.08378	3.0166	0.0824	1.157	0.981	1.363
H_SMK_C01	1		1	-0.06165	0.06928	0.7917	0.3736	0.940	0.821	1.077
IT	1		1	0.08654	0.08312	1.0839	0.2978	1.090	0.926	1.283
ET	1		1	0.64449	0.25770	6.2547	0.0124	1.905	1.150	3.157
A_NIHSS			1	0.02119	0.00592	12.7927	0.0003	1.021	1.010	1.033

BMI_g with y1_stroke: interaction with stroke subtype

PHREG 过程

最大似然估计分析			
参数			标签
H_LIPID01	1		D.History: Lipid metabolism disorders; 0-No; 1-Yes; 1
AI	1		history:Myocardial infarction; 0=NO; 1=YES; 1
H_DRINK_H01	1		D.History: Heavy Drinking(Alcohol consumption>=20g/day); 0-No,1-Yes; 1
H_SMK_C01	1		D.History: Current Smoking; 0-No,1-Yes; 1
IT	1		intravenous thrombolysis, 1=YES,0=NO 1
ET	1		动脉溶栓或机械取栓, 1=YES,0=NO 1
A_NIHSS			F.Admitting NIHSS: Total score;
