# Supplementary material for: Association of body mass index and waist-to-height ratio with outcomes in ischemic stroke: results from the Third China National Stroke Registry
Source: BMC Neurol. 2023 Apr 14;23:152. doi: 10.1186/s12883-023-03165-y (PMC10103413; doi:10.1186/s12883-023-03165-y)
Supplement: Supplementary file 2 — Additional file 2. [file 12883_2023_3165_MOESM2_ESM.zip › raw data/Table-s4.pdf]

FREQ 过程

1=<18.5;2=18.5-<24;3=24-<28;4=≥28				
BMI_g	频数	百分比	累积频数	累积百分比
1	309	2.18	309	2.18
2	5678	40.14	5987	42.32
3	6215	43.93	12202	86.26
4	1944	13.74	14146	100.00

MEANS PROCEDURE

变量	标签	数目	缺失值个数	均值	标准差	最小值	下四分位数	中位数	上四分位数	最大值
WAIST	F.Physical examination: Waist circumference (cm) ;	4805	9341	85.57	14.18	30.00	78.00	86.00	94.00	170.00
AGE	A.Basic Information: Age (years old);	14146	0	62.31	11.30	19.00	54.00	63.00	70.00	96.00
A_NIHSS	F.Admitting NIHSS: Total score;	14146	0	4.41	4.18	0.00	2.00	3.00	6.00	40.00

FREQ 过程

A.Basic Information: Gender; 1-male; 2-female;				
GENDER	频数	百分比	累积频数	累积百分比
1	9720	68.71	9720	68.71
2	4426	31.29	14146	100.00

B.Demography: Race: 1-Han; 99-others;				
ETHNIC	频数	百分比	累积频数	累积百分比
1	13730	97.06	13730	97.06
2	416	2.94	14146	100.00

D.History: Stroke History; 0-No; 1-Yes;				
H_STROKE01	频数	百分比	累积频数	累积百分比
0	11012	77.85	11012	77.85
1	3134	22.15	14146	100.00

D.History: Diabetes; 0-No; 1-Yes;				
H_DIAB01	频数	百分比	累积频数	累积百分比
0	10836	76.60	10836	76.60
1	3310	23.40	14146	100.00

D.History: Heart disease category: Atrial fibrillation(Including medical history and hospitalization diagnosis); 0-No; 1-Yes;				
H_AF01	频数	百分比	累积频数	累积百分比
0	13160	93.03	13160	93.03
1	986	6.97	14146	100.00

history:Myocardial infarction; 0=NO; 1=YES;				
AI	频数	百分比	累积频数	累积百分比
0	13868	98.03	13868	98.03
1	278	1.97	14146	100.00

D.History: Hypertension; 0-No; 1-Yes;				
H_HYPT01	频数	百分比	累积频数	累积百分比
0	5259	37.18	5259	37.18
1	8887	62.82	14146	100.00

FREQ 过程

D.History: Lipid metabolism disorders; 0-No; 1-Yes;				
H_LIPID01	频数	百分比	累积频数	累积百分比
0	13071	92.40	13071	92.40
1	1075	7.60	14146	100.00

D.History: Heavy Drinking(Alcohol consumption>=20g/day); 0-No,1-Yes;				
H_DRINK_H01	频数	百分比	累积频数	累积百分比
0	12136	85.79	12136	85.79
1	2010	14.21	14146	100.00

D.History: Current Smoking; 0-No,1-Yes;				
H_SMK_C01	频数	百分比	累积频数	累积百分比
0	9643	68.17	9643	68.17
1	4503	31.83	14146	100.00

intravenous thrombolysis, 1=YES,0=NO				
IT	频数	百分比	累积频数	累积百分比
0	12626	89.25	12626	89.25
1	1520	10.75	14146	100.00

动脉溶栓或机械取栓, 1=YES,0=NO				
ET	频数	百分比	累积频数	累积百分比
0	14075	99.50	14075	99.50
1	71	0.50	14146	100.00

K.Final diagnosis: cerebral infarction; Etiology according to TOAST system; 1-large artery atherosclerosis; 2-cardiogenic embolism; 3-small artery occlusion; 4-stroke of another determined cause; 5-stroke of an undetermined cause.				
IMG_C_TOAST	频数	百分比	累积频数	累积百分比
1	3667	25.92	3667	25.92
2	881	6.23	4548	32.15
3	3137	22.18	7685	54.33
4	171	1.21	7856	55.54
5	6290	44.46	14146	100.00

FREQ 过程

N12.Follow-up events at 12 months: Recurrence of stroke: 0-No; 1-Yes;				
y1_stroke	频数	百分比	累积频数	累积百分比
0	12722	89.93	12722	89.93
1	1424	10.07	14146	100.00

N12.Follow-up events at 12 months: recurrence of ischemic stroke: 0-No; 1-Yes;				
y1_is	频数	百分比	累积频数	累积百分比
0	12829	90.69	12829	90.69
1	1317	9.31	14146	100.00

N12.Follow-up events at 12 months: recurrence of hemorrhage stroke: 0-No; 1-Yes;				
y1_HS	频数	百分比	累积频数	累积百分比
0	14022	99.12	14022	99.12
1	124	0.88	14146	100.00

I.Inpatient Event: Hemorrhagic transformation after cerebral infarction; 1-No; 2-Yes; 98-UK;				
I_IS_HT	频数	百分比	累积频数	累积百分比
.	86	0.61	86	0.61
1	13867	98.03	13953	98.64
2	193	1.36	14146	100.00

1年心血管源性死亡，0=NO; 1=YES				
death_cvd	频数	百分比	累积频数	累积百分比
0	13949	98.61	13949	98.61
1	197	1.39	14146	100.00

N12.Follow-up events at 12 months:Occurrence of combined vascular event(including cardiovascular death,non-fatal stroke,non-fatal myocardial infarction):0-No;1-Yes;				
y1_comb	频数	百分比	累积频数	累积百分比
0	12641	89.36	12641	89.36
1	1505	10.64	14146	100.00

N12.Follow-up events at 12 months: Whether the patient died: 0-survival;1-death;				
y1_death	频数	百分比	累积频数	累积百分比
0	13660	96.56	13660	96.56
1	486	3.44	14146	100.00

continuous variables, descriptive by group

MEANS PROCEDURE

1=<18.5;2=18.5-<24;3=24-<28;4=≥28	观测数	变量	标签	数目	缺失值个数	均值	标准差	最小值	下四分位数	中位数
1	309	WAIST<br>AGE<br>A_NIHSS	F.Physical examination: Waist circumference (cm) ;<br>A.Basic Information: Age (years old);<br>F.Admitting NIHSS: Total score;	133<br>309<br>309	176<br>0<br>0	73.40<br>69.26<br>6.07	9.95<br>12.08<br>5.48	35.00<br>27.00<br>0.00	69.00<br>61.00<br>2.00	72.00<br>72.00<br>5.00
2	5678	WAIST<br>AGE<br>A_NIHSS	F.Physical examination: Waist circumference (cm) ;<br>A.Basic Information: Age (years old);<br>F.Admitting NIHSS: Total score;	2048<br>5678<br>5678	3630<br>0<br>0	81.28<br>63.77<br>4.72	12.37<br>11.05<br>4.51	30.00<br>24.00<br>0.00	76.00<br>56.00<br>2.00	82.00<br>64.00<br>4.00
3	6215	WAIST<br>AGE<br>A_NIHSS	F.Physical examination: Waist circumference (cm) ;<br>A.Basic Information: Age (years old);<br>F.Admitting NIHSS: Total score;	1984<br>6215<br>6215	4231<br>0<br>0	87.48<br>61.55<br>4.16	13.03<br>10.95<br>3.87	30.00<br>19.00<br>0.00	81.00<br>54.00<br>2.00	88.00<br>62.00<br>3.00
4	1944	WAIST<br>AGE<br>A_NIHSS	F.Physical examination: Waist circumference (cm) ;<br>A.Basic Information: Age (years old);<br>F.Admitting NIHSS: Total score;	640<br>1944<br>1944	1304<br>0<br>0	95.92<br>59.38<br>4.09	16.27<br>11.89<br>3.71	30.00<br>20.00<br>0.00	89.00<br>51.00<br>1.00	98.00<br>60.00<br>3.00

continuous variables, descriptive by group

MEANS PROCEDURE

1=<18.5;2=18.5-<24;3=24-<28;4= ≥ 28	观测数	变量	标签	上四分位数	最大值
1	309	WAIST<br>AGE<br>A_NIHSS	F.Physical examination: Waist circumference (cm) ;<br>A.Basic Information: Age (years old);<br>F.Admitting NIHSS: Total score;	79.00<br>78.00<br>9.00	98.00<br>94.00<br>32.00
2	5678	WAIST<br>AGE<br>A_NIHSS	F.Physical examination: Waist circumference (cm) ;<br>A.Basic Information: Age (years old);<br>F.Admitting NIHSS: Total score;	88.00<br>72.00<br>6.00	165.00<br>96.00<br>40.00
3	6215	WAIST<br>AGE<br>A_NIHSS	F.Physical examination: Waist circumference (cm) ;<br>A.Basic Information: Age (years old);<br>F.Admitting NIHSS: Total score;	95.00<br>69.00<br>6.00	165.00<br>92.00<br>35.00
4	1944	WAIST<br>AGE<br>A_NIHSS	F.Physical examination: Waist circumference (cm) ;<br>A.Basic Information: Age (years old);<br>F.Admitting NIHSS: Total score;	105.00<br>68.00<br>6.00	170.00<br>89.00<br>36.00

Kruskal-Wallis Test among differenct group

NPAR1WAY 过程

变量“WAIST”的 Wilcoxon 评分（秩和）
按变量“BMI_g”分类

BMI_g	数目	评分汇总	H0 之下的期望值	H0 之下的标准差	均值评分
3	1984	5271299.50	4767552.0	47315.8283	2656.90499
2	2048	3901378.50	4921344.0	47524.4861	1904.96997
1	133	135671.50	319599.0	15765.6292	1020.08647
4	640	2238065.50	1537920.0	32653.6369	3496.97734

已将平均评分用于结值。

Kruskal-Wallis 检验

卡方	自由度	Pr > 卡方
861.6782	3	<.0001

WAIST的评分类型为Wilcoxon 评分的分布

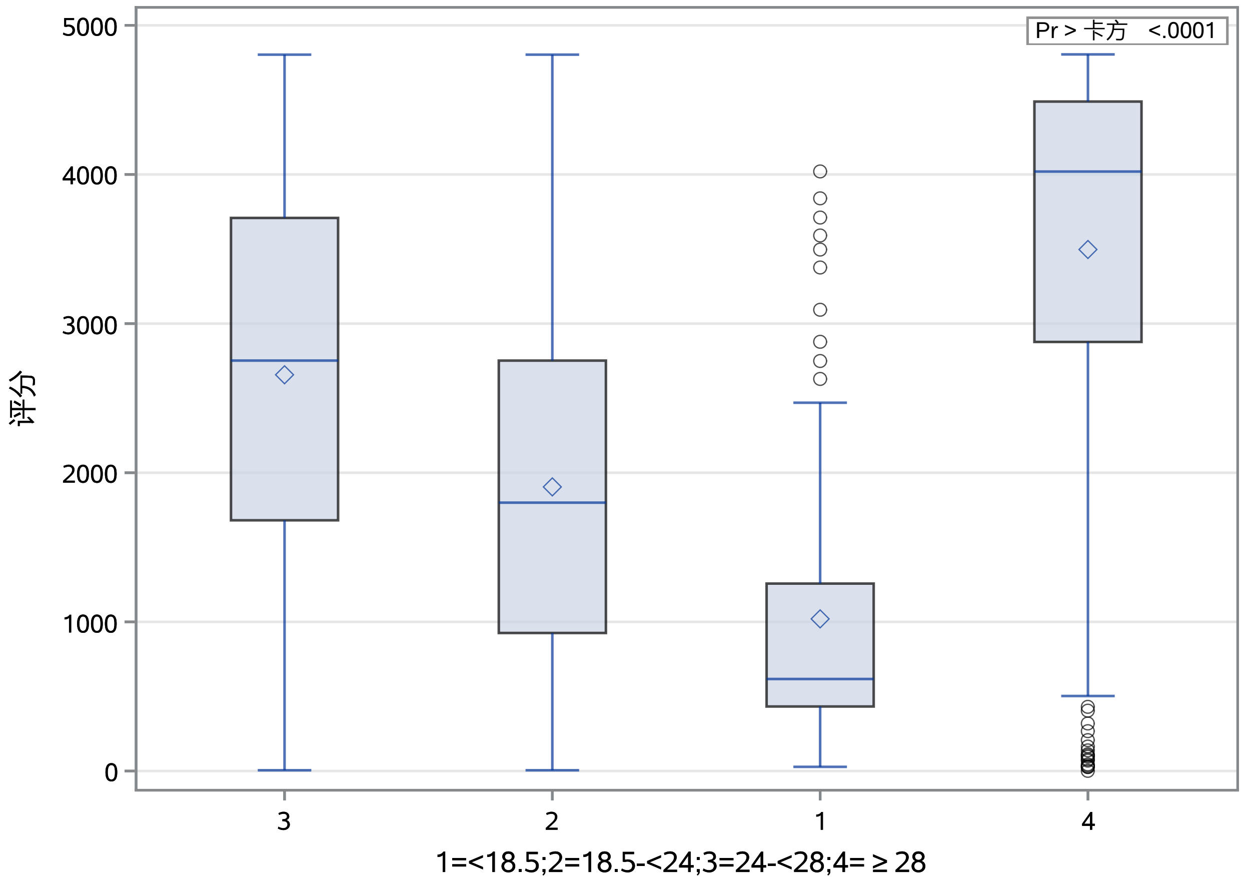

Kruskal-Wallis Test among differenct group

NPAR1WAY 过程

变量 “AGE” 的 Wilcoxon 评分（秩和）
按变量 “BMI_g” 分类

BMI_g	数目	评分汇总	H0 之下的期望值	H0 之下的标准差	均值评分
3	6215	42163077.0	43961802.5	240972.939	6784.08319
2	5678	43080195.5	40163333.0	237997.203	7587.21302
1	309	2946920.5	2185711.5	70971.493	9536.95955
4	1944	11871538.0	13750884.0	167165.778	6106.75823

已将平均评分用于结值。

Kruskal-Wallis 检验

卡方	自由度	Pr > 卡方
342.7013	3	<.0001

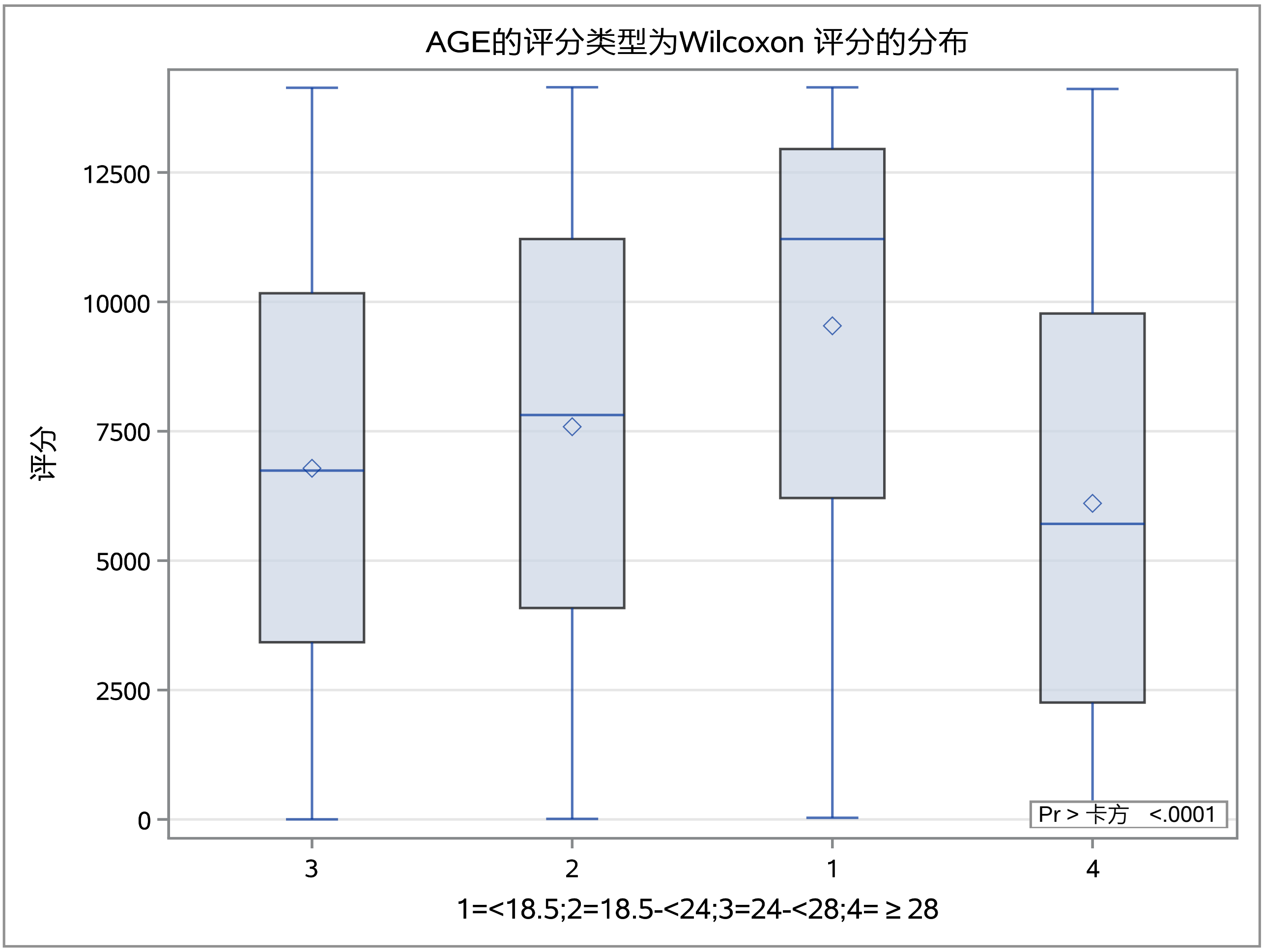

Kruskal-Wallis Test among differenct group

NPAR1WAY 过程

变量“A_NIHSS”的 Wilcoxon 评分（秩和）
按变量“BMI_g”分类

BMI_g	数目	评分汇总	H0 之下的期望值	H0 之下的标准差	均值评分
3	6215	42672059.0	43961802.5	239637.681	6865.97892
2	5678	41483498.5	40163333.0	236678.433	7306.00537
1	309	2575812.5	2185711.5	70578.232	8335.96278
4	1944	13330361.0	13750884.0	166239.494	6857.18158

已将平均评分用于结值。

Kruskal-Wallis 检验

卡方	自由度	Pr > 卡方
70.2671	3	<.0001

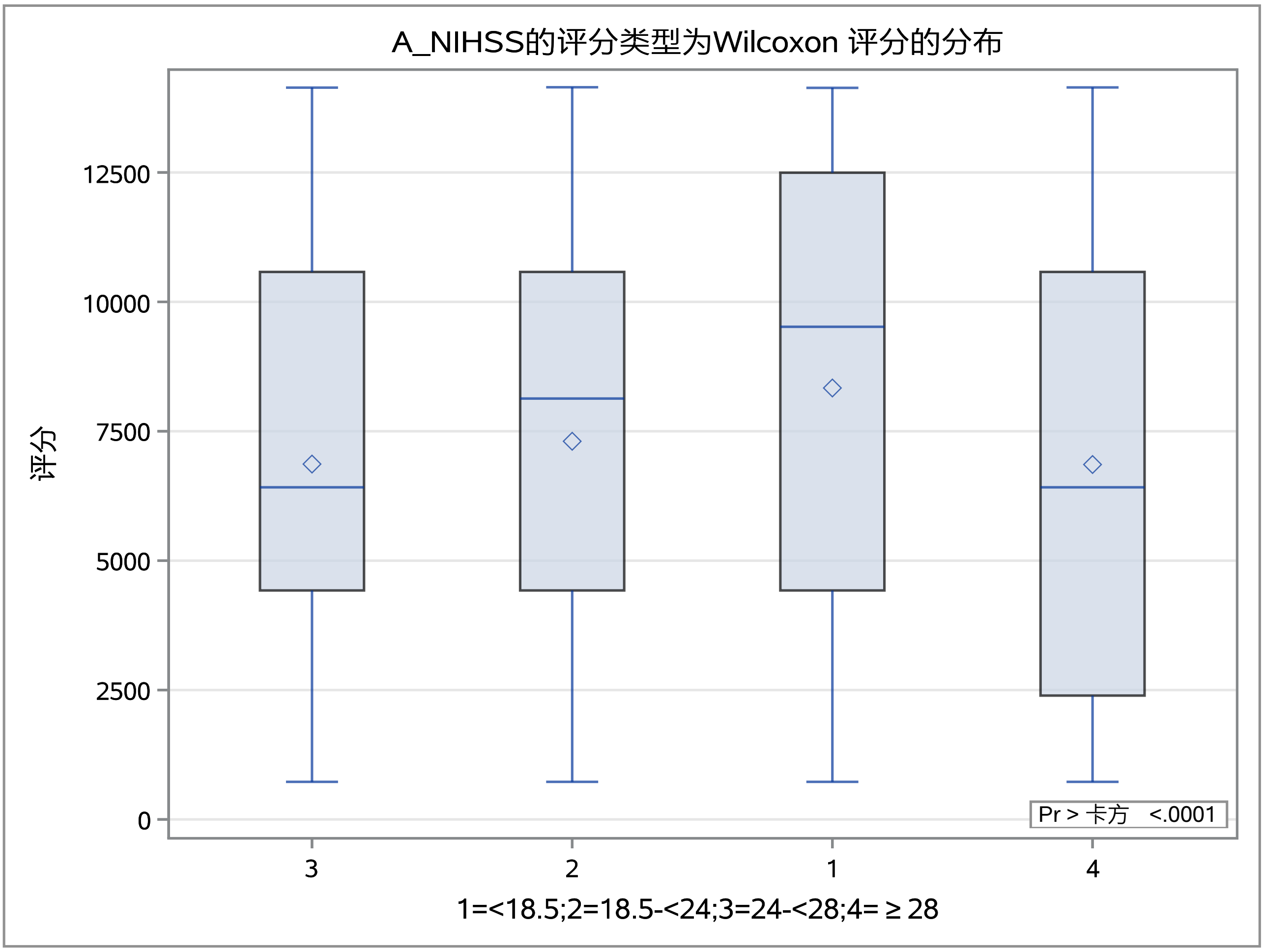

continuous variables,p for linear trend

CORR 过程

2 变量:	WAIST BMI_g

简单统计量							
变量	数目	均值	标准差	中位数	最小值	最大值	标签
WAIST	4805	85.57274	14.17565	86.00000	30.00000	170.00000	F.Physical examination: Waist circumference (cm) ;
BMI_g	14146	2.69235	0.72909	3.00000	1.00000	4.00000	1=<18.5;2=18.5-<24;3=24-<28;4= ≥ 28

Kendall Tau b 相关系数<br>Prob > |tau|, H0: Tau=0<br>观测数		
	WAIST	BMI_g
WAIST<br>F.Physical examination: Waist circumference (cm) ;	1.00000<br><br>4805	0.33777<br><.0001<br>4805
BMI_g<br>1=<18.5;2=18.5-<24;3=24-<28;4= ≥ 28	0.33777<br><.0001<br>4805	1.00000<br><br>14146

continuous variables,p for linear trend

CORR 过程

2 变量:	AGE BMI_g

简单统计量							
变量	数目	均值	标准差	中位数	最小值	最大值	标签
AGE	14146	62.31267	11.29549	63.00000	19.00000	96.00000	A.Basic Information: Age (years old);
BMI_g	14146	2.69235	0.72909	3.00000	1.00000	4.00000	1=<18.5;2=18.5-<24;3=24-<28;4=≥28

Kendall Tau b 相关系数, N = 14146<br>Prob > |tau|, H0: Tau=0		
	AGE	BMI_g
AGE<br>A.Basic Information: Age (years old);	1.00000	-0.11588<br><.0001
BMI_g<br>1=<18.5;2=18.5-<24;3=24-<28;4=≥28	-0.11588<br><.0001	1.00000

continuous variables,p for linear trend

CORR 过程

2 变量:	A_NIHSS BMI_g

简单统计量							
变量	数目	均值	标准差	中位数	最小值	最大值	标签
A_NIHSS	14146	4.41489	4.17815	3.00000	0	40.00000	F.Admitting NIHSS: Total score;
BMI_g	14146	2.69235	0.72909	3.00000	1.00000	4.00000	1=<18.5;2=18.5-<24;3=24-<28;4=≥28

Kendall Tau b 相关系数, N = 14146<br>Prob > |tau|, H0: Tau=0		
	A_NIHSS	BMI_g
A_NIHSS<br>F.Admitting NIHSS: Total score;	1.00000	-0.04906<br><.0001
BMI_g<br>1=<18.5;2=18.5-<24;3=24-<28;4=≥28	-0.04906<br><.0001	1.00000

categorical variables, descriptive by group and chisq test

FREQ 过程

频数
列百分比

GENDER-BMI_g表					
GENDER(A.Basic Information: Gender; 1-male; 2-female;)	BMI_g(1=<18.5;2=18.5-<24;3=24-<28;4=≥28)				
	1	2	3	4	合计
1	177<br>57.28	3858<br>67.95	4487<br>72.20	1198<br>61.63	9720
2	132<br>42.72	1820<br>32.05	1728<br>27.80	746<br>38.37	4426
合计	309	5678	6215	1944	14146

表“BMI_g-GENDER”的统计量

统计量	自由度	值	概率
卡方	3	100.8328	<.0001
似然比卡方检验	3	99.0094	<.0001
Mantel-Haenszel 卡方	1	0.3461	0.5563
Phi 系数		0.0844	
列联系数		0.0841	
Cramer V		0.0844	

样本大小 = 14146

频数
列百分比

ETHNIC-BMI_g表					
ETHNIC(B.Demography: Race: 1-Han; 99-others;)	BMI_g(1=<18.5;2=18.5-<24;3=24-<28;4=≥28)				
	1	2	3	4	合计
1	299<br>96.76	5547<br>97.69	6022<br>96.89	1862<br>95.78	13730
2	10<br>3.24	131<br>2.31	193<br>3.11	82<br>4.22	416
合计	309	5678	6215	1944	14146

表“BMI_g-ETHNIC”的统计量

统计量	自由度	值	概率
卡方	3	19.7836	0.0002
似然比卡方检验	3	19.1056	0.0003
Mantel-Haenszel 卡方	1	16.2085	<.0001
Phi 系数		0.0374	
列联系数		0.0374	
Cramer V		0.0374	

样本大小 = 14146

categorical variables, descriptive by group and chisq test

FREQ 过程

频数
列百分比

H_STROKE01-BMI_g表					
H_STROKE01(D.History: Stroke History; 0-No; 1-Yes;)	BMI_g(1=<18.5;2=18.5-<24;3=24-<28;4=≥28)				
	1	2	3	4	合计
0	229<br>74.11	4480<br>78.90	4807<br>77.35	1496<br>76.95	11012
1	80<br>25.89	1198<br>21.10	1408<br>22.65	448<br>23.05	3134
合计	309	5678	6215	1944	14146

表“BMI_g-H_STROKE01”的统计量

统计量	自由度	值	概率
卡方	3	7.9647	0.0467
似然比卡方检验	3	7.9025	0.0481
Mantel-Haenszel 卡方	1	2.2628	0.1325
Phi 系数		0.0237	
列联系数		0.0237	
Cramer V		0.0237	

样本大小 = 14146

频数
列百分比

H_DIAB01-BMI_g表					
H_DIAB01(D.History: Diabetes; 0-No; 1-Yes;)	BMI_g(1=<18.5;2=18.5-<24;3=24-<28;4=≥28)				
	1	2	3	4	合计
0	268<br>86.73	4515<br>79.52	4644<br>74.72	1409<br>72.48	10836
1	41<br>13.27	1163<br>20.48	1571<br>25.28	535<br>27.52	3310
合计	309	5678	6215	1944	14146

表“BMI_g-H_DIAB01”的统计量

统计量	自由度	值	概率
卡方	3	75.2976	<.0001
似然比卡方检验	3	77.5977	<.0001
Mantel-Haenszel 卡方	1	70.5293	<.0001
Phi 系数		0.0730	
列联系数		0.0728	
Cramer V		0.0730	

样本大小 = 14146

categorical variables, descriptive by group and chisq test

FREQ 过程

频数
列百分比

H_AF01-BMI_g表

H_AF01(D.History: Heart disease category: Atrial fibrillation(Including medical history and hospitalization diagnosis); 0-No; 1-Yes;)	BMI_g(1=<18.5;2=18.5-<24;3=24-<28;4=≥28)				
	1	2	3	4	合计
0	267<br>86.41	5265<br>92.73	5810<br>93.48	1818<br>93.52	13160
1	42<br>13.59	413<br>7.27	405<br>6.52	126<br>6.48	986
合计	309	5678	6215	1944	14146

表“BMI_g-H_AF01”的统计量

统计量	自由度	值	概率
卡方	3	24.3922	<.0001
似然比卡方检验	3	20.2100	0.0002
Mantel-Haenszel 卡方	1	9.3881	0.0022
Phi 系数		0.0415	
列联系数		0.0415	
Cramer V		0.0415	

样本大小 = 14146

频数
列百分比

AI-BMI_g表

AI(history:Myocardial infarction; 0=NO; 1=YES;)	BMI_g(1=<18.5;2=18.5-<24;3=24-<28;4=≥28)				
	1	2	3	4	合计
0	299<br>96.76	5572<br>98.13	6096<br>98.09	1901<br>97.79	13868
1	10<br>3.24	106<br>1.87	119<br>1.91	43<br>2.21	278
合计	309	5678	6215	1944	14146

表“BMI_g-AI”的统计量

统计量	自由度	值	概率
卡方	3	3.5726	0.3115
似然比卡方检验	3	3.1358	0.3712
Mantel-Haenszel 卡方	1	0.0441	0.8337
Phi 系数		0.0159	
列联系数		0.0159	
Cramer V		0.0159	

样本大小 = 14146

FREQ 过程

频数<br>列百分比

H_HYPT01-BMI_g表					
H_HYPT01(D.History: Hypertension; 0-No; 1-Yes;)	BMI_g(1=<18.5;2=18.5-<24;3=24-<28;4=≥28)				
	1	2	3	4	合计
0	150<br>48.54	2485<br>43.77	2125<br>34.19	499<br>25.67	5259
1	159<br>51.46	3193<br>56.23	4090<br>65.81	1445<br>74.33	8887
合计	309	5678	6215	1944	14146

表“BMI_g-H_HYPT01”的统计量

统计量	自由度	值	概率
卡方	3	256.5765	<.0001
似然比卡方检验	3	260.3864	<.0001
Mantel-Haenszel 卡方	1	254.1336	<.0001
Phi 系数		0.1347	
列联系数		0.1335	
Cramer V		0.1347	

样本大小 = 14146

频数<br>列百分比

H_LIPID01-BMI_g表					
H_LIPID01(D.History: Lipid metabolism disorders; 0-No; 1-Yes;)	BMI_g(1=<18.5;2=18.5-<24;3=24-<28;4=≥28)				
	1	2	3	4	合计
0	293<br>94.82	5323<br>93.75	5733<br>92.24	1722<br>88.58	13071
1	16<br>5.18	355<br>6.25	482<br>7.76	222<br>11.42	1075
合计	309	5678	6215	1944	14146

表“BMI_g-H_LIPID01”的统计量

统计量	自由度	值	概率
卡方	3	57.8783	<.0001
似然比卡方检验	3	54.0562	<.0001
Mantel-Haenszel 卡方	1	52.0134	<.0001
Phi 系数		0.0640	
列联系数		0.0638	
Cramer V		0.0640	

样本大小 = 14146

categorical variables, descriptive by group and chisq test

FREQ 过程

频数
列百分比

H_DRINK_H01-BMI_g表					
H_DRINK_H01(D.History: Heavy Drinking(Alcohol consumption>=20g/day); 0-No,1-Yes;)	BMI_g(1=<18.5;2=18.5-<24;3=24-<28;4= ≥ 28)				
	1	2	3	4	合计
0	271<br>87.70	4879<br>85.93	5281<br>84.97	1705<br>87.71	12136
1	38<br>12.30	799<br>14.07	934<br>15.03	239<br>12.29	2010
合计	309	5678	6215	1944	14146

表“BMI_g-H_DRINK_H01”的统计量

统计量	自由度	值	概率
卡方	3	10.2816	0.0163
似然比卡方检验	3	10.5015	0.0148
Mantel-Haenszel 卡方	1	0.3389	0.5605
Phi 系数		0.0270	
列联系数		0.0269	
Cramer V		0.0270	

样本大小 = 14146

频数
列百分比

H_SMK_C01-BMI_g表					
H_SMK_C01(D.History: Current Smoking; 0-No,1-Yes;)	BMI_g(1=<18.5;2=18.5-<24;3=24-<28;4= ≥ 28)				
	1	2	3	4	合计
0	210<br>67.96	3892<br>68.55	4189<br>67.40	1352<br>69.55	9643
1	99<br>32.04	1786<br>31.45	2026<br>32.60	592<br>30.45	4503
合计	309	5678	6215	1944	14146

表“BMI_g-H_SMK_C01”的统计量

统计量	自由度	值	概率
卡方	3	3.7659	0.2879
似然比卡方检验	3	3.7732	0.2870
Mantel-Haenszel 卡方	1	0.0272	0.8691
Phi 系数		0.0163	
列联系数		0.0163	
Cramer V		0.0163	

样本大小 = 14146

categorical variables, descriptive by group and chisq test

FREQ 过程

频数
列百分比

IT-BMI_g表

IT(intravenous thrombolysis, 1=YES,0=NO)	BMI_g(1=<18.5;2=18.5-<24;3=24-<28;4=≥28)				
	1	2	3	4	合计
0	254<br>82.20	5047<br>88.89	5576<br>89.72	1749<br>89.97	12626
1	55<br>17.80	631<br>11.11	639<br>10.28	195<br>10.03	1520
合计	309	5678	6215	1944	14146

表“BMI_g-IT”的统计量

统计量	自由度	值	概率
卡方	3	19.2611	0.0002
似然比卡方检验	3	16.9521	0.0007
Mantel-Haenszel 卡方	1	8.5173	0.0035
Phi 系数		0.0369	
列联系数		0.0369	
Cramer V		0.0369	

样本大小 = 14146

频数
列百分比

ET-BMI_g表

ET(动脉溶栓或机械取栓, 1=YES,0=NO)	BMI_g(1=<18.5;2=18.5-<24;3=24-<28;4=≥28)				
	1	2	3	4	合计
0	306<br>99.03	5644<br>99.40	6190<br>99.60	1935<br>99.54	14075
1	3<br>0.97	34<br>0.60	25<br>0.40	9<br>0.46	71
合计	309	5678	6215	1944	14146

表“BMI_g-ET”的统计量

统计量	自由度	值	概率
卡方	3	3.7233	0.2929
似然比卡方检验	3	3.4595	0.3261
Mantel-Haenszel 卡方	1	2.2329	0.1351
Phi 系数		0.0162	
列联系数		0.0162	
Cramer V		0.0162	

样本大小 = 14146

categorical variables, descriptive by group and chisq test

FREQ 过程

频数
列百分比

IMG_C_TOAST-BMI_g表

IMG_C_TOAST(K.Final diagnosis: cerebral infarction; Etiology according to TOAST system; 1-large artery atherosclerosis; 2-cardiogenic embolism; 3-small artery occlusion; 4-stroke of another determined cause; 5-stroke of an undetermined cause.)	BMI_g(1=<18.5;2=18.5-<24;3=24-<28;4=≥28)				
	1	2	3	4	合计
1	78<br>25.24	1454<br>25.61	1609<br>25.89	526<br>27.06	3667
2	28<br>9.06	384<br>6.76	359<br>5.78	110<br>5.66	881
3	52<br>16.83	1217<br>21.43	1413<br>22.74	455<br>23.41	3137
4	5<br>1.62	75<br>1.32	65<br>1.05	26<br>1.34	171
5	146<br>47.25	2548<br>44.87	2769<br>44.55	827<br>42.54	6290
合计	309	5678	6215	1944	14146

表“BMI_g-IMG_C_TOAST”的统计量

统计量	自由度	值	概率
卡方	12	23.5113	0.0237
似然比卡方检验	12	23.3757	0.0247
Mantel-Haenszel 卡方	1	1.7499	0.1859
Phi 系数		0.0408	
列联系数		0.0407	
Cramer V		0.0235	

样本大小 = 14146

频数
列百分比

y1_stroke-BMI_g表

y1_stroke(N12.Follow-up events at 12 months: Recurrence of stroke: 0-No; 1-Yes;)	BMI_g(1=<18.5;2=18.5-<24;3=24-<28;4=≥28)				
	1	2	3	4	合计
0	273<br>88.35	5121<br>90.19	5591<br>89.96	1737<br>89.35	12722
1	36<br>11.65	557<br>9.81	624<br>10.04	207<br>10.65	1424
合计	309	5678	6215	1944	14146

categorical variables, descriptive by group and chisq test

FREQ 过程

表“BMI_g-y1_stroke”的统计量

统计量	自由度	值	概率
卡方	3	2.0009	0.5722
似然比卡方检验	3	1.9550	0.5818
Mantel-Haenszel 卡方	1	0.3804	0.5374
Phi 系数		0.0119	
列联系数		0.0119	
Cramer V		0.0119	

样本大小 = 14146

频数
列百分比

y1_is-BMI_g表					
y1_is(N12.Follow-up events at 12 months: recurrence of ischemic stroke: 0-No; 1-Yes;)	BMI_g(1=<18.5;2=18.5-<24;3=24-<28;4=≥28)				
	1	2	3	4	合计
0	274<br>88.67	5167<br>91.00	5635<br>90.67	1753<br>90.17	12829
1	35<br>11.33	511<br>9.00	580<br>9.33	191<br>9.83	1317
合计	309	5678	6215	1944	14146

表“BMI_g-y1_is”的统计量

统计量	自由度	值	概率
卡方	3	2.7510	0.4316
似然比卡方检验	3	2.6606	0.4470
Mantel-Haenszel 卡方	1	0.3626	0.5471
Phi 系数		0.0139	
列联系数		0.0139	
Cramer V		0.0139	

样本大小 = 14146

频数
列百分比

y1_HS-BMI_g表					
y1_HS(N12.Follow-up events at 12 months: recurrence of hemorrhage stroke: 0-No; 1-Yes;)	BMI_g(1=<18.5;2=18.5-<24;3=24-<28;4=≥28)				
	1	2	3	4	合计
0	306<br>99.03	5626<br>99.08	6166<br>99.21	1924<br>98.97	14022
1	3<br>0.97	52<br>0.92	49<br>0.79	20<br>1.03	124
合计	309	5678	6215	1944	14146

categorical variables, descriptive by group and chisq test

FREQ 过程

表“BMI_g-y1_HS”的统计量

统计量	自由度	值	概率
卡方	3	1.2067	0.7514
似然比卡方检验	3	1.1962	0.7539
Mantel-Haenszel 卡方	1	0.0003	0.9853
Phi 系数		0.0092	
列联系数		0.0092	
Cramer V		0.0092	

样本大小 = 14146

频数
列百分比

I_IS_HT-BMI_g表					
I_IS_HT(I.Inpatient Event: Hemorrhagic transformation after cerebral infarction; 1-No; 2-Yes; 98-UK;)	BMI_g(1=<18.5;2=18.5-<24;3=24-<28;4= ≥ 28)				
	1	2	3	4	合计
1	297<br>96.43	5553<br>98.41	6100<br>98.85	1917<br>98.92	13867
2	11<br>3.57	90<br>1.59	71<br>1.15	21<br>1.08	193
合计	308	5643	6171	1938	14060
频数缺失 = 86					

表“BMI_g-I_IS_HT”的统计量

统计量	自由度	值	概率
卡方	3	16.5022	0.0009
似然比卡方检验	3	13.2698	0.0041
Mantel-Haenszel 卡方	1	9.9070	0.0016
Phi 系数		0.0343	
列联系数		0.0342	
Cramer V		0.0343	

样本大小 = 14060
频数缺失 = 86

频数
列百分比

death_cvd-BMI_g表					
death_cvd(1年心血管源性死亡，0=NO; 1=YES)	BMI_g(1=<18.5;2=18.5-<24;3=24-<28;4= ≥ 28)				
	1	2	3	4	合计
0	302<br>97.73	5585<br>98.36	6135<br>98.71	1927<br>99.13	13949
1	7<br>2.27	93<br>1.64	80<br>1.29	17<br>0.87	197
合计	309	5678	6215	1944	14146

categorical variables, descriptive by group and chisq test

FREQ 过程

表“BMI_g-death_cvd”的统计量

统计量	自由度	值	概率
卡方	3	8.5049	0.0367
似然比卡方检验	3	8.6905	0.0337
Mantel-Haenszel 卡方	1	8.3667	0.0038
Phi 系数		0.0245	
列联系数		0.0245	
Cramer V		0.0245	

样本大小 = 14146

频数
列百分比

y1_comb-BMI_g表

y1_comb(N12.Follow-up events at 12 months:Occurrence of combined vascular event(including cardiovascular death,non-fatal stroke,non-fatal myocardial infarction):0-No;1-Yes;)	BMI_g(1=<18.5;2=18.5-<24;3=24-<28;4=≥28)				
	1	2	3	4	合计
0	270<br>87.38	5083<br>89.52	5558<br>89.43	1730<br>88.99	12641
1	39<br>12.62	595<br>10.48	657<br>10.57	214<br>11.01	1505
合计	309	5678	6215	1944	14146

表“BMI_g-y1_comb”的统计量

统计量	自由度	值	概率
卡方	3	1.7389	0.6283
似然比卡方检验	3	1.6729	0.6430
Mantel-Haenszel 卡方	1	0.0225	0.8807
Phi 系数		0.0111	
列联系数		0.0111	
Cramer V		0.0111	

样本大小 = 14146

频数
列百分比

y1_death-BMI_g表

y1_death(N12.Follow-up events at 12 months: Whether the patient died: 0-survival;1-death;)	BMI_g(1=<18.5;2=18.5-<24;3=24-<28;4=≥28)				
	1	2	3	4	合计
0	277<br>89.64	5447<br>95.93	6040<br>97.18	1896<br>97.53	13660
1	32<br>10.36	231<br>4.07	175<br>2.82	48<br>2.47	486
合计	309	5678	6215	1944	14146

categorical variables, descriptive by group and chisq test

FREQ 过程

表“BMI_g-y1_death”的统计量

统计量	自由度	值	概率
卡方	3	64.1292	<.0001
似然比卡方检验	3	49.6080	<.0001
Mantel-Haenszel 卡方	1	38.0924	<.0001
Phi 系数		0.0673	
列联系数		0.0672	
Cramer V		0.0673	

样本大小 = 14146

categorical variables, descriptive by group and P for linear trend

FREQ 过程

频数
列百分比

GENDER-BMI_g表					
GENDER(A.Basic Information: Gender; 1-male; 2-female;)	BMI_g(1=<18.5;2=18.5-<24;3=24-<28;4=≥28)				
	1	2	3	4	合计
1	177<br>57.28	3858<br>67.95	4487<br>72.20	1198<br>61.63	9720
2	132<br>42.72	1820<br>32.05	1728<br>27.80	746<br>38.37	4426
合计	309	5678	6215	1944	14146

表“BMI_g-GENDER”的统计量

Cochran-Armitage 趋势检验	
统计量 (Z)	-0.5883
单侧 Pr < Z	0.2782
双侧 Pr > |Z|	0.5563

样本大小 = 14146

频数
列百分比

ETHNIC-BMI_g表					
ETHNIC(B.Demography: Race: 1-Han; 99-others;)	BMI_g(1=<18.5;2=18.5-<24;3=24-<28;4=≥28)				
	1	2	3	4	合计
1	299<br>96.76	5547<br>97.69	6022<br>96.89	1862<br>95.78	13730
2	10<br>3.24	131<br>2.31	193<br>3.11	82<br>4.22	416
合计	309	5678	6215	1944	14146

表“BMI_g-ETHNIC”的统计量

Cochran-Armitage 趋势检验	
统计量 (Z)	-4.0261
单侧 Pr < Z	<.0001
双侧 Pr > |Z|	<.0001

样本大小 = 14146

频数
列百分比

H_STROKE01-BMI_g表					
H_STROKE01(D.History: Stroke History; 0-No; 1-Yes;)	BMI_g(1=<18.5;2=18.5-<24;3=24-<28;4=≥28)				
	1	2	3	4	合计
0	229<br>74.11	4480<br>78.90	4807<br>77.35	1496<br>76.95	11012
1	80<br>25.89	1198<br>21.10	1408<br>22.65	448<br>23.05	3134
合计	309	5678	6215	1944	14146

categorical variables, descriptive by group and P for linear trend

FREQ 过程

表“BMI_g-H_STROKE01”的统计量

Cochran-Armitage 趋势检验	
统计量 (Z)	-1.5043
单侧 Pr < Z	0.0663
双侧 Pr > |Z|	0.1325

样本大小 = 14146

频数
列百分比

H_DIAB01-BMI_g表					
H_DIAB01(D.History: Diabetes; 0-No; 1-Yes;)	BMI_g(1=<18.5;2=18.5-<24;3=24-<28;4=≥28)				
	1	2	3	4	合计
0	268<br>86.73	4515<br>79.52	4644<br>74.72	1409<br>72.48	10836
1	41<br>13.27	1163<br>20.48	1571<br>25.28	535<br>27.52	3310
合计	309	5678	6215	1944	14146

表“BMI_g-H_DIAB01”的统计量

Cochran-Armitage 趋势检验	
统计量 (Z)	-8.3985
单侧 Pr < Z	<.0001
双侧 Pr > |Z|	<.0001

样本大小 = 14146

频数
列百分比

H_AF01-BMI_g表					
H_AF01(D.History: Heart disease category: Atrial fibrillation(Including medical history and hospitalization diagnosis); 0-No; 1-Yes;)	BMI_g(1=<18.5;2=18.5-<24;3=24-<28;4=≥28)				
	1	2	3	4	合计
0	267<br>86.41	5265<br>92.73	5810<br>93.48	1818<br>93.52	13160
1	42<br>13.59	413<br>7.27	405<br>6.52	126<br>6.48	986
合计	309	5678	6215	1944	14146

表“BMI_g-H_AF01”的统计量

Cochran-Armitage 趋势检验	
统计量 (Z)	3.0641
单侧 Pr > Z	0.0011
双侧 Pr > |Z|	0.0022

样本大小 = 14146

categorical variables, descriptive by group and P for linear trend

FREQ 过程

频数
列百分比

AI-BMI_g表

AI(history:Myocardial infarction; 0=NO; 1=YES;)	BMI_g(1=<18.5;2=18.5-<24;3=24-<28;4=≥28) 1	2	3	4	合计
0	299<br>96.76	5572<br>98.13	6096<br>98.09	1901<br>97.79	13868
1	10<br>3.24	106<br>1.87	119<br>1.91	43<br>2.21	278
合计	309	5678	6215	1944	14146

表“BMI_g-AI”的统计量

Cochran-Armitage 趋势检验	
统计量 (Z)	-0.2099
单侧 Pr < Z	0.4169
双侧 Pr > |Z|	0.8337

样本大小 = 14146

频数
列百分比

H_HYPT01-BMI_g表

H_HYPT01(D.History: Hypertension; 0-No; 1-Yes;)	BMI_g(1=<18.5;2=18.5-<24;3=24-<28;4=≥28) 1	2	3	4	合计
0	150<br>48.54	2485<br>43.77	2125<br>34.19	499<br>25.67	5259
1	159<br>51.46	3193<br>56.23	4090<br>65.81	1445<br>74.33	8887
合计	309	5678	6215	1944	14146

表“BMI_g-H_HYPT01”的统计量

Cochran-Armitage 趋势检验	
统计量 (Z)	-15.9421
单侧 Pr < Z	<.0001
双侧 Pr > |Z|	<.0001

样本大小 = 14146

频数
列百分比

H_LIPID01-BMI_g表

H_LIPID01(D.History: Lipid metabolism disorders; 0-No; 1-Yes;)	BMI_g(1=<18.5;2=18.5-<24;3=24-<28;4=≥28) 1	2	3	4	合计
0	293<br>94.82	5323<br>93.75	5733<br>92.24	1722<br>88.58	13071
1	16<br>5.18	355<br>6.25	482<br>7.76	222<br>11.42	1075
合计	309	5678	6215	1944	14146

categorical variables, descriptive by group and P for linear trend

FREQ 过程

表“BMI_g-H_LIPID01”的统计量

Cochran-Armitage 趋势检验	
统计量 (Z)	-7.2123
单侧 Pr < Z	<.0001
双侧 Pr > |Z|	<.0001

样本大小 = 14146

频数
列百分比

H_DRINK_H01-BMI_g表

H_DRINK_H01(D.History: Heavy Drinking(Alcohol consumption>=20g/day); 0-No,1-Yes;)	BMI_g(1=<18.5;2=18.5-<24;3=24-<28;4= ≥ 28)				
	1	2	3	4	合计
0	271<br>87.70	4879<br>85.93	5281<br>84.97	1705<br>87.71	12136
1	38<br>12.30	799<br>14.07	934<br>15.03	239<br>12.29	2010
合计	309	5678	6215	1944	14146

表“BMI_g-H_DRINK_H01”的统计量

Cochran-Armitage 趋势检验	
统计量 (Z)	0.5822
单侧 Pr > Z	0.2802
双侧 Pr > |Z|	0.5604

样本大小 = 14146

频数
列百分比

H_SMK_C01-BMI_g表

H_SMK_C01(D.History: Current Smoking; 0-No,1-Yes;)	BMI_g(1=<18.5;2=18.5-<24;3=24-<28;4= ≥ 28)				
	1	2	3	4	合计
0	210<br>67.96	3892<br>68.55	4189<br>67.40	1352<br>69.55	9643
1	99<br>32.04	1786<br>31.45	2026<br>32.60	592<br>30.45	4503
合计	309	5678	6215	1944	14146

表“BMI_g-H_SMK_C01”的统计量

Cochran-Armitage 趋势检验	
统计量 (Z)	0.1648
单侧 Pr > Z	0.4345
双侧 Pr > |Z|	0.8691

样本大小 = 14146

categorical variables, descriptive by group and P for linear trend

FREQ 过程

频数
列百分比

IT-BMI_g表

IT(intravenous thrombolysis, 1=YES,0=NO)	BMI_g(1=<18.5;2=18.5-<24;3=24-<28;4= ≥ 28)				
	1	2	3	4	合计
0	254<br>82.20	5047<br>88.89	5576<br>89.72	1749<br>89.97	12626
1	55<br>17.80	631<br>11.11	639<br>10.28	195<br>10.03	1520
合计	309	5678	6215	1944	14146

表“BMI_g-IT”的统计量

Cochran-Armitage 趋势检验	
统计量 (Z)	2.9185
单侧 Pr > Z	0.0018
双侧 Pr > |Z|	0.0035

样本大小 = 14146

频数
列百分比

ET-BMI_g表

ET(动脉溶栓或机械取栓, 1=YES,0=NO)	BMI_g(1=<18.5;2=18.5-<24;3=24-<28;4= ≥ 28)				
	1	2	3	4	合计
0	306<br>99.03	5644<br>99.40	6190<br>99.60	1935<br>99.54	14075
1	3<br>0.97	34<br>0.60	25<br>0.40	9<br>0.46	71
合计	309	5678	6215	1944	14146

表“BMI_g-ET”的统计量

Cochran-Armitage 趋势检验	
统计量 (Z)	1.4943
单侧 Pr > Z	0.0675
双侧 Pr > |Z|	0.1351

样本大小 = 14146

categorical variables, descriptive by group and P for linear trend

FREQ 过程

频数<br>列百分比

IMG_C_TOAST-BMI_g表

IMG_C_TOAST(K.Final diagnosis: cerebral infarction; Etiology according to TOAST system; 1-large artery atherosclerosis; 2-cardiogenic embolism; 3-small artery occlusion; 4-stroke of another determined cause; 5-stroke of an undetermined cause.)	BMI_g(1=<18.5;2=18.5-<24;3=24-<28;4=≥28)				
	1	2	3	4	合计
1	78<br>25.24	1454<br>25.61	1609<br>25.89	526<br>27.06	3667
2	28<br>9.06	384<br>6.76	359<br>5.78	110<br>5.66	881
3	52<br>16.83	1217<br>21.43	1413<br>22.74	455<br>23.41	3137
4	5<br>1.62	75<br>1.32	65<br>1.05	26<br>1.34	171
5	146<br>47.25	2548<br>44.87	2769<br>44.55	827<br>42.54	6290
合计	309	5678	6215	1944	14146

频数<br>列百分比

y1_stroke-BMI_g表

y1_stroke(N12.Follow-up events at 12 months: Recurrence of stroke: 0-No; 1-Yes;)	BMI_g(1=<18.5;2=18.5-<24;3=24-<28;4=≥28)				
	1	2	3	4	合计
0	273<br>88.35	5121<br>90.19	5591<br>89.96	1737<br>89.35	12722
1	36<br>11.65	557<br>9.81	624<br>10.04	207<br>10.65	1424
合计	309	5678	6215	1944	14146

表“BMI_g-y1_stroke”的统计量

Cochran-Armitage 趋势检验	
统计量 (Z)	-0.6168
单侧 Pr < Z	0.2687
双侧 Pr > |Z|	0.5374

样本大小 = 14146

categorical variables, descriptive by group and P for linear trend

FREQ 过程

频数<br>列百分比

y1_is-BMI_g表					
y1_is(N12.Follow-up events at 12 months: recurrence of ischemic stroke: 0-No; 1-Yes;)	BMI_g(1=<18.5;2=18.5-<24;3=24-<28;4= ≥ 28)				
	1	2	3	4	合计
0	274<br>88.67	5167<br>91.00	5635<br>90.67	1753<br>90.17	12829
1	35<br>11.33	511<br>9.00	580<br>9.33	191<br>9.83	1317
合计	309	5678	6215	1944	14146

表“BMI_g-y1_is”的统计量

Cochran-Armitage<br>趋势检验	
统计量 (Z)	-0.6022
单侧 Pr < Z	0.2735
双侧 Pr > |Z|	0.5470

样本大小 = 14146

频数<br>列百分比

y1_HS-BMI_g表					
y1_HS(N12.Follow-up events at 12 months: recurrence of hemorrhage stroke: 0-No; 1-Yes;)	BMI_g(1=<18.5;2=18.5-<24;3=24-<28;4= ≥ 28)				
	1	2	3	4	合计
0	306<br>99.03	5626<br>99.08	6166<br>99.21	1924<br>98.97	14022
1	3<br>0.97	52<br>0.92	49<br>0.79	20<br>1.03	124
合计	309	5678	6215	1944	14146

表“BMI_g-y1_HS”的统计量

Cochran-Armitage<br>趋势检验	
统计量 (Z)	-0.0184
单侧 Pr < Z	0.4927
双侧 Pr > |Z|	0.9853

样本大小 = 14146

categorical variables, descriptive by group and P for linear trend

FREQ 过程

频数
列百分比

I_IS_HT-BMI_g表

I_IS_HT(I.Inpatient Event: Hemorrhagic transformation after cerebral infarction; 1-No; 2-Yes; 98-UK;)	BMI_g(1=<18.5;2=18.5-<24;3=24-<28;4=≥28)				
	1	2	3	4	合计
1	297<br>96.43	5553<br>98.41	6100<br>98.85	1917<br>98.92	13867
2	11<br>3.57	90<br>1.59	71<br>1.15	21<br>1.08	193
合计	308	5643	6171	1938	14060
频数缺失 = 86					

表“BMI_g-I_IS_HT”的统计量

Cochran-Armitage 趋势检验	
统计量 (Z)	3.1477
单侧 Pr > Z	0.0008
双侧 Pr > |Z|	0.0016

样本大小 = 14060
频数缺失 = 86

频数
列百分比

death_cvd-BMI_g表

death_cvd(1年心血管源性死亡，0=NO; 1=YES)	BMI_g(1=<18.5;2=18.5-<24;3=24-<28;4=≥28)				
	1	2	3	4	合计
0	302<br>97.73	5585<br>98.36	6135<br>98.71	1927<br>99.13	13949
1	7<br>2.27	93<br>1.64	80<br>1.29	17<br>0.87	197
合计	309	5678	6215	1944	14146

表“BMI_g-death_cvd”的统计量

Cochran-Armitage 趋势检验	
统计量 (Z)	2.8926
单侧 Pr > Z	0.0019
双侧 Pr > |Z|	0.0038

样本大小 = 14146

categorical variables, descriptive by group and P for linear trend

FREQ 过程

频数<br>列百分比	y1_comb-BMI_g表				
y1_comb(N12.Follow-up events at 12 months:Occurrence of combined vascular event(including cardiovascular death,non-fatal stroke,non-fatal myocardial infarction):0-No;1-Yes;)	BMI_g(1=<18.5;2=18.5-<24;3=24-<28;4= ≥ 28)				
	1	2	3	4	合计
0	270<br>87.38	5083<br>89.52	5558<br>89.43	1730<br>88.99	12641
1	39<br>12.62	595<br>10.48	657<br>10.57	214<br>11.01	1505
合计	309	5678	6215	1944	14146

表“BMI_g-y1_comb”的统计量

Cochran-Armitage<br>趋势检验	
统计量 (Z)	-0.1500
单侧 Pr < Z	0.4404
双侧 Pr > |Z|	0.8807

样本大小 = 14146

频数<br>列百分比	y1_death-BMI_g表				
y1_death(N12.Follow-up events at 12 months: Whether the patient died: 0-survival;1-death;)	BMI_g(1=<18.5;2=18.5-<24;3=24-<28;4= ≥ 28)				
	1	2	3	4	合计
0	277<br>89.64	5447<br>95.93	6040<br>97.18	1896<br>97.53	13660
1	32<br>10.36	231<br>4.07	175<br>2.82	48<br>2.47	486
合计	309	5678	6215	1944	14146

表“BMI_g-y1_death”的统计量

Cochran-Armitage<br>趋势检验	
统计量 (Z)	6.1721
单侧 Pr > Z	<.0001
双侧 Pr > |Z|	<.0001

样本大小 = 14146
