# Supplementary material for: Association of body mass index and waist-to-height ratio with outcomes in ischemic stroke: results from the Third China National Stroke Registry
Source: BMC Neurol. 2023 Apr 14;23:152. doi: 10.1186/s12883-023-03165-y (PMC10103413; doi:10.1186/s12883-023-03165-y)
Supplement: Supplementary file 2 — Additional file 2. [file 12883_2023_3165_MOESM2_ESM.zip › raw data/Table-s3.pdf]

male: BMI_g with y1_death: Descriptive results

FREQ 过程

频数<br>行百分比

BMI_g-y1_death表			
	y1_death(N12.Follow-up events at 12 months: Whether the patient died: 0-survival;1-death;)		
BMI_g(1=<18.5;2=18.5-<23;3=23-<27.5;4=≥27.5)	0	1	合计
1	164<br>92.66	13<br>7.34	177
2	2496<br>96.04	103<br>3.96	2599
3	5145<br>97.02	158<br>2.98	5303
4	1607<br>97.93	34<br>2.07	1641
合计	9412	308	9720

表“y1_death-BMI_g”的统计量

统计量	自由度	值	概率
卡方	3	22.4572	<.0001
似然比卡方检验	3	20.2989	0.0001
Mantel-Haenszel 卡方	1	19.4969	<.0001
Phi 系数		0.0481	
列联系数		0.0480	
Cramer V		0.0481	

样本大小 = 9720

male: BMI_g with y1_death: adjusted model

PHREG 过程

模型信息		
数据集	WORK.MALE	
因变量	y1_death_dd	N12.Follow-up events at 12 months: Days from onset to death;(day);
删失变量	y1_death	N12.Follow-up events at 12 months: Whether the patient died: 0-survival;1-death;
删失值	0	
结值处理	BRESLOW	

读取的观测数<br>使用的观测数	9720<br>9720

分类水平信息					
分类	值	设计变量			
BMI_g	4	1	0	0	
	3	0	1	0	
	2	0	0	0	
	1	0	0	1	
ETHNIC	2	1			
	1	0			
H_DIAB01	1	1			
	0	0			
H_AF01	1	1			
	0	0			
H_HYPT01	1	1			
	0	0			
H_LIPID01	1	1			
	0	0			
AI	1	1			
	0	0			
H_DRINK_H01	1	1			
	0	0			
H_SMK_C01	1	1			
	0	0			
IT	1	1			
	0	0			
ET	1	1			
	0	0			
IMG_C_TOAST	5	1	0	0	0
	4	0	1	0	0
	3	0	0	1	0
	2	0	0	0	1
	1	0	0	0	0

male: BMI_g with y1_death: adjusted model

PHREG 过程

事件和删失值个数汇总			
合计	事件	删失	删失百分比
9720	308	9412	96.83

收敛状态
满足收敛准则 (GCONV=1E-8)。

模型拟合统计量		
准则	无协变量	带协变量
-2 LOG L	5634.956	5233.281
AIC	5634.956	5271.281
SBC	5634.956	5342.153

检验全局原假设: BETA=0			
检验	卡方	自由度	Pr > 卡方
似然比	401.6743	19	<.0001
评分	548.3797	19	<.0001
Wald	466.1042	19	<.0001

3 型检验			
效应	自由度	Wald 卡方	Pr > 卡方
BMI_g	3	3.6450	0.3024
AGE	1	85.8033	<.0001
ETHNIC	1	1.7814	0.1820
H_DIAB01	1	10.7027	0.0011
H_AF01	1	19.9059	<.0001
H_HYPT01	1	0.2175	0.6409
H_LIPID01	1	0.5877	0.4433
AI	1	0.7083	0.4000
H_DRINK_H01	1	5.4401	0.0197
H_SMK_C01	1	0.0169	0.8965
IT	1	11.6360	0.0006
ET	1	8.9229	0.0028
IMG_C_TOAST	4	24.3614	<.0001
A_NIHSS	1	159.3452	<.0001

male: BMI_g with y1_death: adjusted model

PHREG 过程

最大似然估计分析									
参数		自由度	参数估计	标准误差	卡方	Pr > 卡方	危险率	95% 危险率置信限	
BMI_g	4	1	-0.33579	0.20278	2.7421	0.0977	0.715	0.480	1.064
BMI_g	3	1	-0.09649	0.12907	0.5589	0.4547	0.908	0.705	1.169
BMI_g	1	1	0.20349	0.29832	0.4653	0.4952	1.226	0.683	2.199
AGE		1	0.05474	0.00591	85.8033	<.0001	1.056	1.044	1.069
ETHNIC	2	1	0.39539	0.29624	1.7814	0.1820	1.485	0.831	2.654
H_DIAB01	1	1	0.42677	0.13045	10.7027	0.0011	1.532	1.187	1.979
H_AF01	1	1	0.87999	0.19724	19.9059	<.0001	2.411	1.638	3.549
H_HYPT01	1	1	0.05657	0.12130	0.2175	0.6409	1.058	0.834	1.342
H_LIPID01	1	1	-0.19363	0.25257	0.5877	0.4433	0.824	0.502	1.352
AI	1	1	0.25169	0.29905	0.7083	0.4000	1.286	0.716	2.311
H_DRINK_H01	1	1	-0.46016	0.19729	5.4401	0.0197	0.631	0.429	0.929
H_SMK_C01	1	1	-0.01735	0.13335	0.0169	0.8965	0.983	0.757	1.276
IT	1	1	-0.69478	0.20368	11.6360	0.0006	0.499	0.335	0.744
ET	1	1	1.03856	0.34768	8.9229	0.0028	2.825	1.429	5.584
IMG_C_TOAST	5	1	-0.23033	0.13668	2.8396	0.0920	0.794	0.608	1.038
IMG_C_TOAST	4	1	0.75115	0.42179	3.1715	0.0749	2.119	0.927	4.845
IMG_C_TOAST	3	1	-0.91181	0.22194	16.8788	<.0001	0.402	0.260	0.621
IMG_C_TOAST	2	1	-0.54532	0.25675	4.5110	0.0337	0.580	0.350	0.959
A_NIHSS		1	0.10235	0.00811	159.3452	<.0001	1.108	1.090	1.126

male: BMI_g with y1_death: adjusted model

PHREG 过程

最大似然估计分析		
参数		标签
BMI_g	4	1=<18.5;2=18.5-<23;3=23-<27.5;4=≥27.5 4
BMI_g	3	1=<18.5;2=18.5-<23;3=23-<27.5;4=≥27.5 3
BMI_g	1	1=<18.5;2=18.5-<23;3=23-<27.5;4=≥27.5 1
AGE		A.Basic Information: Age (years old);
ETHNIC	2	B.Demography: Race: 1-Han; 99-others; 2
H_DIAB01	1	D.History: Diabetes; 0-No; 1-Yes; 1
H_AF01	1	D.History: Heart disease category: Atrial fibrillation(Including medical history and hospitalization diagnosis); 0-No; 1-Yes; 1
H_HYPT01	1	D.History: Hypertension; 0-No; 1-Yes; 1
H_LIPID01	1	D.History: Lipid metabolism disorders; 0-No; 1-Yes; 1
AI	1	history:Myocardial infarction; 0=NO; 1=YES; 1
H_DRINK_H01	1	D.History: Heavy Drinking(Alcohol consumption>=20g/day); 0-No,1-Yes; 1
H_SMK_C01	1	D.History: Current Smoking; 0-No,1-Yes; 1
IT	1	intravenous thrombolysis, 1=YES,0=NO 1
ET	1	动脉溶栓或机械取栓, 1=YES,0=NO 1
IMG_C_TOAST	5	K.Final diagnosis: cerebral infarction; Etiology according to TOAST system; 1-large artery atherosclerosis; 2-cardiogenic embolism; 3-small artery occlusion; 4-stroke of another determined cause; 5-stroke of an undetermined cause. 5
IMG_C_TOAST	4	K.Final diagnosis: cerebral infarction; Etiology according to TOAST system; 1-large artery atherosclerosis; 2-cardiogenic embolism; 3-small artery occlusion; 4-stroke of another determined cause; 5-stroke of an undetermined cause. 4
IMG_C_TOAST	3	K.Final diagnosis: cerebral infarction; Etiology according to TOAST system; 1-large artery atherosclerosis; 2-cardiogenic embolism; 3-small artery occlusion; 4-stroke of another determined cause; 5-stroke of an undetermined cause. 3
IMG_C_TOAST	2	K.Final diagnosis: cerebral infarction; Etiology according to TOAST system; 1-large artery atherosclerosis; 2-cardiogenic embolism; 3-small artery occlusion; 4-stroke of another determined cause; 5-stroke of an undetermined cause. 2
A_NIHSS		F.Admitting NIHSS: Total score;

PHREG 过程

模型信息		
数据集	WORK.DATA_OVERALL	
因变量	y1_death_dd	N12.Follow-up events at 12 months: Days from onset to death;(day);
删失变量	y1_death	N12.Follow-up events at 12 months: Whether the patient died: 0-survival;1-death;
删失值	0	
结值处理	BRESLOW	

读取的观测数<br>使用的观测数	14146<br>14146

分类水平信息						
分类	值	设计变量				
BMI_g	4	1	0	0	
	3	0	1	0	
	2	0	0	0	
	1	0	0	1	
ETHNIC	2	1			
	1	0			
H_DIAB01	1	1			
	0	0			
H_AF01	1	1			
	0	0			
H_HYPT01	1	1			
	0	0			
H_LIPID01	1	1			
	0	0			
AI	1	1			
	0	0			
H_DRINK_H01	1	1			
	0	0			
H_SMK_C01	1	1			
	0	0			
IT	1	1			
	0	0			
ET	1	1			
	0	0			
IMG_C_TOAST	5	1	0	0	0
	4	0	1	0	0
	3	0	0	1	0
	2	0	0	0	1
	1	0	0	0	0

PHREG 过程

事件和删失值个数汇总			
合计	事件	删失	删失百分比
14146	486	13660	96.56

收敛状态
满足收敛准则 (GCONV=1E-8)。

模型拟合统计量		
准则	无协变量	带协变量
-2 LOG L	9255.945	8606.088
AIC	9255.945	8652.088
SBC	9255.945	8748.371

检验全局原假设: BETA=0			
检验	卡方	自由度	Pr > 卡方
似然比	649.8566	23	<.0001
评分	891.6098	23	<.0001
Wald	758.4266	23	<.0001

联合检验			
效应	自由度	Wald 卡方	Pr > 卡方
BMI_g	3	1.1775	0.7584
GENDER	1	1.8258	0.1766
GENDER*BMI_g	3	3.0076	0.3904
AGE	1	150.7508	<.0001
ETHNIC	1	3.0866	0.0789
H_DIAB01	1	12.8226	0.0003
H_AF01	1	28.8683	<.0001
H_HYPT01	1	0.1198	0.7293
H_LIPID01	1	2.3003	0.1294
AI	1	5.3780	0.0204
H_DRINK_H01	1	6.9570	0.0083
H_SMK_C01	1	0.9710	0.3244
IT	1	19.8678	<.0001
ET	1	7.6676	0.0056
IMG_C_TOAST	4	34.8224	<.0001
A_NIHSS	1	226.3090	<.0001

Note: Under full-rank parameterizations, Type 3 effect tests are replaced by joint tests. The joint test for an effect is a test that all of the parameters associated with that effect are zero. Such joint tests might not be equivalent to Type 3 effect tests under GLM parameterization.

PHREG 过程

最大似然估计分析										
参数		自由度	参数估计	标准误差	卡方	Pr > 卡方	危险率	95% 危险率置信限	
BMI_g	4	1	-0.41353	0.46907	0.7772	0.3780	.	.	.
BMI_g	3	1	-0.17392	0.30735	0.3202	0.5715	.	.	.
BMI_g	1	1	-0.50088	0.64834	0.5968	0.4398	.	.	.
GENDER		1	-0.22824	0.16892	1.8258	0.1766	.	.	.
GENDER*BMI_g	4	1	0.10072	0.31694	0.1010	0.7507	.	.	.
GENDER*BMI_g	3	1	0.08469	0.21498	0.1552	0.6936	.	.	.
GENDER*BMI_g	1	1	0.68533	0.39686	2.9821	0.0842	.	.	.
AGE		1	0.05908	0.00481	150.7508	<.0001	1.061	1.051	1.071
ETHNIC	2	1	0.41434	0.23584	3.0866	0.0789	1.513	0.953	2.403
H_DIAB01	1	1	0.37403	0.10445	12.8226	0.0003	1.454	1.184	1.784
H_AF01	1	1	0.80187	0.14924	28.8683	<.0001	2.230	1.664	2.987
H_HYPT01	1	1	0.03382	0.09772	0.1198	0.7293	1.034	0.854	1.253
H_LIPID01	1	1	-0.31533	0.20791	2.3003	0.1294	0.730	0.485	1.097
AI	1	1	0.52512	0.22644	5.3780	0.0204	1.691	1.085	2.635
H_DRINK_H01	1	1	-0.51671	0.19590	6.9570	0.0083	0.596	0.406	0.876
H_SMK_C01	1	1	0.12248	0.12430	0.9710	0.3244	1.130	0.886	1.442
IT	1	1	-0.74102	0.16625	19.8678	<.0001	0.477	0.344	0.660
ET	1	1	0.86759	0.31332	7.6676	0.0056	2.381	1.289	4.400
IMG_C_TOAST	5	1	-0.24185	0.11139	4.7141	0.0299	0.785	0.631	0.977
IMG_C_TOAST	4	1	0.65361	0.32740	3.9854	0.0459	1.922	1.012	3.652
IMG_C_TOAST	3	1	-0.92963	0.18262	25.9125	<.0001	0.395	0.276	0.565
IMG_C_TOAST	2	1	-0.42431	0.19330	4.8185	0.0282	0.654	0.448	0.956
A_NIHSS		1	0.09550	0.00635	226.3090	<.0001	1.100	1.087	1.114

PHREG 过程

最大似然估计分析		
参数		标签
BMI_g	4	1=<18.5;2=18.5-<23;3=23-<27.5;4=≥27.5 4
BMI_g	3	1=<18.5;2=18.5-<23;3=23-<27.5;4=≥27.5 3
BMI_g	1	1=<18.5;2=18.5-<23;3=23-<27.5;4=≥27.5 1
GENDER		A.Basic Information: Gender; 1-male; 2-female;
GENDER*BMI_g	4	1=<18.5;2=18.5-<23;3=23-<27.5;4=≥27.5 4 * A.Basic Information: Gender; 1-male; 2-female;
GENDER*BMI_g	3	1=<18.5;2=18.5-<23;3=23-<27.5;4=≥27.5 3 * A.Basic Information: Gender; 1-male; 2-female;
GENDER*BMI_g	1	1=<18.5;2=18.5-<23;3=23-<27.5;4=≥27.5 1 * A.Basic Information: Gender; 1-male; 2-female;
AGE		A.Basic Information: Age (years old);
ETHNIC	2	B.Demography: Race: 1-Han; 99-others; 2
H_DIAB01	1	D.History: Diabetes; 0-No; 1-Yes; 1
H_AF01	1	D.History: Heart disease category: Atrial fibrillation(Including medical history and hospitalization diagnosis); 0-No; 1-Yes; 1
H_HYPT01	1	D.History: Hypertension; 0-No; 1-Yes; 1
H_LIPID01	1	D.History: Lipid metabolism disorders; 0-No; 1-Yes; 1
AI	1	history:Myocardial infarction; 0=NO; 1=YES; 1
H_DRINK_H01	1	D.History: Heavy Drinking(Alcohol consumption>=20g/day); 0-No,1-Yes; 1
H_SMK_C01	1	D.History: Current Smoking; 0-No,1-Yes; 1
IT	1	intravenous thrombolysis, 1=YES,0=NO 1
ET	1	动脉溶栓或机械取栓, 1=YES,0=NO 1
IMG_C_TOAST	5	K.Final diagnosis: cerebral infarction; Etiology according to TOAST system; 1-large artery atherosclerosis; 2-cardiogenic embolism; 3-small artery occlusion; 4-stroke of another determined cause; 5-stroke of an undetermined cause. 5
IMG_C_TOAST	4	K.Final diagnosis: cerebral infarction; Etiology according to TOAST system; 1-large artery atherosclerosis; 2-cardiogenic embolism; 3-small artery occlusion; 4-stroke of another determined cause; 5-stroke of an undetermined cause. 4
IMG_C_TOAST	3	K.Final diagnosis: cerebral infarction; Etiology according to TOAST system; 1-large artery atherosclerosis; 2-cardiogenic embolism; 3-small artery occlusion; 4-stroke of another determined cause; 5-stroke of an undetermined cause. 3
IMG_C_TOAST	2	K.Final diagnosis: cerebral infarction; Etiology according to TOAST system; 1-large artery atherosclerosis; 2-cardiogenic embolism; 3-small artery occlusion; 4-stroke of another determined cause; 5-stroke of an undetermined cause. 2
A_NIHSS		F.Admitting NIHSS: Total score;

male: BMI_g with y1_comb: Descriptive results

FREQ 过程

频数<br>行百分比

BMI_g-y1_comb表			
	y1_comb(N12.Follow-up events at 12 months:Occurrence of combined vascular event(including cardiovascular death,non-fatal stroke,non-fatal myocardial infarction):0-No;1-Yes;)		
BMI_g(1=<18.5;2=18.5-<23;3=23-<27.5;4= ≥ 27.5)	0	1	合计
1	158<br>89.27	19<br>10.73	177
2	2332<br>89.73	267<br>10.27	2599
3	4764<br>89.84	539<br>10.16	5303
4	1464<br>89.21	177<br>10.79	1641
合计	8718	1002	9720

表“y1_comb-BMI_g”的统计量

统计量	自由度	值	概率
卡方	3	0.5628	0.9049
似然比卡方检验	3	0.5574	0.9061
Mantel-Haenszel 卡方	1	0.1191	0.7300
Phi 系数		0.0076	
列联系数		0.0076	
Cramer V		0.0076	

样本大小 = 9720

male: BMI_g with y1_comb: adjusted model

PHREG 过程

模型信息		
数据集	WORK.MALE	
因变量	y1_comb_dd	N12.Follow-up events at 12 months: Days from onset to occurrence of combined vascular event;(day);
删失变量	y1_comb	N12.Follow-up events at 12 months:Occurrence of combined vascular event(including cardiovascular death,non-fatal stroke,non-fatal myocardial infarction):0-No;1-Yes;
删失值	0	
结值处理	BRESLOW	

读取的观测数	9720
使用的观测数	9720

分类水平信息					
分类	值	设计变量			
BMI_g	4	1	0	0	
	3	0	1	0	
	2	0	0	0	
	1	0	0	1	
ETHNIC	2	1			
	1	0			
H_DIAB01	1	1			
	0	0			
H_AF01	1	1			
	0	0			
H_HYPT01	1	1			
	0	0			
H_LIPID01	1	1			
	0	0			
AI	1	1			
	0	0			
H_DRINK_H01	1	1			
	0	0			
H_SMK_C01	1	1			
	0	0			
IT	1	1			
	0	0			
ET	1	1			
	0	0			
IMG_C_TOAST	5	1	0	0	0
	4	0	1	0	0
	3	0	0	1	0
	2	0	0	0	1
	1	0	0	0	0

male: BMI_g with y1_comb: adjusted model

PHREG 过程

事件和删失值个数汇总			
合计	事件	删失	删失百分比
9720	1002	8718	89.69

收敛状态
满足收敛准则 (GCONV=1E-8)。

模型拟合统计量		
准则	无协变量	带协变量
-2 LOG L	18261.254	18138.646
AIC	18261.254	18176.646
SBC	18261.254	18269.932

检验全局原假设: BETA=0			
检验	卡方	自由度	Pr > 卡方
似然比	122.6077	19	<.0001
评分	132.7455	19	<.0001
Wald	129.1213	19	<.0001

3 型检验			
效应	自由度	Wald 卡方	Pr > 卡方
BMI_g	3	0.9160	0.8216
AGE	1	8.8209	0.0030
ETHNIC	1	0.0017	0.9675
H_DIAB01	1	11.8061	0.0006
H_AF01	1	5.2098	0.0225
H_HYPT01	1	2.4897	0.1146
H_LIPID01	1	0.1707	0.6795
AI	1	1.0761	0.2996
H_DRINK_H01	1	1.7237	0.1892
H_SMK_C01	1	1.5056	0.2198
IT	1	0.0121	0.9124
ET	1	5.8583	0.0155
IMG_C_TOAST	4	37.5006	<.0001
A_NIHSS	1	16.8091	<.0001

male: BMI_g with y1_comb: adjusted model

PHREG 过程

最大似然估计分析									
参数		自由度	参数估计	标准误差	卡方	Pr > 卡方	危险率	95% 危险率置信限	
BMI_g	4	1	0.08379	0.09970	0.7063	0.4007	1.087	0.894	1.322
BMI_g	3	1	0.00491	0.07588	0.0042	0.9484	1.005	0.866	1.166
BMI_g	1	1	-0.00347	0.23827	0.0002	0.9884	0.997	0.625	1.590
AGE		1	0.00905	0.00305	8.8209	0.0030	1.009	1.003	1.015
ETHNIC	2	1	-0.00758	0.18581	0.0017	0.9675	0.992	0.690	1.428
H_DIAB01	1	1	0.25250	0.07349	11.8061	0.0006	1.287	1.115	1.487
H_AF01	1	1	0.35157	0.15403	5.2098	0.0225	1.421	1.051	1.922
H_HYPT01	1	1	0.10619	0.06730	2.4897	0.1146	1.112	0.975	1.269
H_LIPID01	1	1	-0.04958	0.12001	0.1707	0.6795	0.952	0.752	1.204
AI	1	1	0.19394	0.18695	1.0761	0.2996	1.214	0.842	1.751
H_DRINK_H01	1	1	0.10928	0.08324	1.7237	0.1892	1.115	0.948	1.313
H_SMK_C01	1	1	-0.08676	0.07070	1.5056	0.2198	0.917	0.798	1.053
IT	1	1	0.01099	0.09991	0.0121	0.9124	1.011	0.831	1.230
ET	1	1	0.71744	0.29642	5.8583	0.0155	2.049	1.146	3.663
IMG_C_TOAST	5	1	-0.32079	0.07482	18.3809	<.0001	0.726	0.627	0.840
IMG_C_TOAST	4	1	0.04966	0.29437	0.0285	0.8660	1.051	0.590	1.871
IMG_C_TOAST	3	1	-0.54675	0.09626	32.2610	<.0001	0.579	0.479	0.699
IMG_C_TOAST	2	1	-0.27449	0.17283	2.5223	0.1122	0.760	0.542	1.066
A_NIHSS		1	0.02854	0.00696	16.8091	<.0001	1.029	1.015	1.043

male: BMI_g with y1_comb: adjusted model

PHREG 过程

最大似然估计分析		
参数		标签
BMI_g	4	1=<18.5;2=18.5-<23;3=23-<27.5;4= ≥ 27.5 4
BMI_g	3	1=<18.5;2=18.5-<23;3=23-<27.5;4= ≥ 27.5 3
BMI_g	1	1=<18.5;2=18.5-<23;3=23-<27.5;4= ≥ 27.5 1
AGE		A.Basic Information: Age (years old);
ETHNIC	2	B.Demography: Race: 1-Han; 99-others; 2
H_DIAB01	1	D.History: Diabetes; 0-No; 1-Yes; 1
H_AF01	1	D.History: Heart disease category: Atrial fibrillation(Including medical history and hospitalization diagnosis); 0-No; 1-Yes; 1
H_HYPT01	1	D.History: Hypertension; 0-No; 1-Yes; 1
H_LIPID01	1	D.History: Lipid metabolism disorders; 0-No; 1-Yes; 1
AI	1	history:Myocardial infarction; 0=NO; 1=YES; 1
H_DRINK_H01	1	D.History: Heavy Drinking(Alcohol consumption>=20g/day); 0-No,1-Yes; 1
H_SMK_C01	1	D.History: Current Smoking; 0-No,1-Yes; 1
IT	1	intravenous thrombolysis, 1=YES,0=NO 1
ET	1	动脉溶栓或机械取栓, 1=YES,0=NO 1
IMG_C_TOAST	5	K.Final diagnosis: cerebral infarction; Etiology according to TOAST system; 1-large artery atherosclerosis; 2-cardiogenic embolism; 3-small artery occlusion; 4-stroke of another determined cause; 5-stroke of an undetermined cause. 5
IMG_C_TOAST	4	K.Final diagnosis: cerebral infarction; Etiology according to TOAST system; 1-large artery atherosclerosis; 2-cardiogenic embolism; 3-small artery occlusion; 4-stroke of another determined cause; 5-stroke of an undetermined cause. 4
IMG_C_TOAST	3	K.Final diagnosis: cerebral infarction; Etiology according to TOAST system; 1-large artery atherosclerosis; 2-cardiogenic embolism; 3-small artery occlusion; 4-stroke of another determined cause; 5-stroke of an undetermined cause. 3
IMG_C_TOAST	2	K.Final diagnosis: cerebral infarction; Etiology according to TOAST system; 1-large artery atherosclerosis; 2-cardiogenic embolism; 3-small artery occlusion; 4-stroke of another determined cause; 5-stroke of an undetermined cause. 2
A_NIHSS		F.Admitting NIHSS: Total score;

BMI_g with y1_comb: interaction

PHREG 过程

模型信息		
数据集	WORK.DATA_OVERALL	
因变量	y1_comb_dd	N12.Follow-up events at 12 months: Days from onset to occurrence of combined vascular event;(day);
删失变量	y1_comb	N12.Follow-up events at 12 months:Occurrence of combined vascular event(including cardiovascular death,non-fatal stroke,non-fatal myocardial infarction):0-No;1-Yes;
删失值	0	
结值处理	BRESLOW	

读取的观测数	14146
使用的观测数	14146

分类水平信息						
分类	值	设计变量				
BMI_g	4	1	0	0		
	3	0	1	0		
	2	0	0	0		
	1	0	0	1		
ETHNIC	2	1				
	1	0				
H_DIAB01	1	1				
	0	0				
H_AF01	1	1				
	0	0				
H_HYPT01	1	1				
	0	0				
H_LIPID01	1	1				
	0	0				
AI	1	1				
	0	0				
H_DRINK_H01	1	1				
	0	0				
H_SMK_C01	1	1				
	0	0				
IT	1	1				
	0	0				
ET	1	1				
	0	0				
IMG_C_TOAST	5	1	0	0	0	
	4	0	1	0	0	
	3	0	0	1	0	
	2	0	0	0	1	
	1	0	0	0	0	

BMI_g with y1_comb: interaction

PHREG 过程

事件和删失值个数汇总			
合计	事件	删失	删失百分比
14146	1505	12641	89.36

收敛状态
满足收敛准则 (GCONV=1E-8)。

模型拟合统计量		
准则	无协变量	带协变量
-2 LOG L	28553.433	28380.441
AIC	28553.433	28426.441
SBC	28553.433	28548.722

检验全局原假设: BETA=0			
检验	卡方	自由度	Pr > 卡方
似然比	172.9917	23	<.0001
评分	184.7459	23	<.0001
Wald	180.5634	23	<.0001

联合检验			
效应	自由度	Wald 卡方	Pr > 卡方
BMI_g	3	0.8445	0.8388
GENDER	1	0.0013	0.9708
GENDER*BMI_g	3	0.9483	0.8138
AGE	1	19.7272	<.0001
ETHNIC	1	0.4570	0.4990
H_DIAB01	1	14.1516	0.0002
H_AF01	1	13.2816	0.0003
H_HYPT01	1	2.6923	0.1008
H_LIPID01	1	0.6429	0.4227
AI	1	2.1480	0.1428
H_DRINK_H01	1	1.2781	0.2583
H_SMK_C01	1	0.2813	0.5958
IT	1	0.2124	0.6449
ET	1	5.9946	0.0143
IMG_C_TOAST	4	54.5175	<.0001
A_NIHSS	1	15.9017	<.0001

Note: Under full-rank parameterizations, Type 3 effect tests are replaced by joint tests. The joint test for an effect is a test that all of the parameters associated with that effect are zero. Such joint tests might not be equivalent to Type 3 effect tests under GLM parameterization.

BMI_g with y1_comb: interaction

PHREG 过程

最大似然估计分析									
参数		自由度	参数估计	标准误差	卡方	Pr > 卡方	危险率	95%危险率置信限	
BMI_g	4	1	0.11681	0.23583	0.2453	0.6204	.	.	.
BMI_g	3	1	-0.02099	0.18386	0.0130	0.9091	.	.	.
BMI_g	1	1	-0.32277	0.53238	0.3676	0.5443	.	.	.
GENDER		1	0.00394	0.10768	0.0013	0.9708	.	.	.
GENDER*BMI_g	4	1	-0.01751	0.16382	0.0114	0.9149	.	.	.
GENDER*BMI_g	3	1	0.03186	0.12984	0.0602	0.8062	.	.	.
GENDER*BMI_g	1	1	0.30720	0.33744	0.8288	0.3626	.	.	.
AGE		1	0.01111	0.00250	19.7272	<.0001	1.011	1.006	1.016
ETHNIC	2	1	-0.10860	0.16065	0.4570	0.4990	0.897	0.655	1.229
H_DIAB01	1	1	0.22185	0.05897	14.1516	0.0002	1.248	1.112	1.401
H_AF01	1	1	0.42156	0.11567	13.2816	0.0003	1.524	1.215	1.912
H_HYPT01	1	1	0.09166	0.05586	2.6923	0.1008	1.096	0.982	1.223
H_LIPID01	1	1	-0.07995	0.09972	0.6429	0.4227	0.923	0.759	1.122
AI	1	1	0.23157	0.15800	2.1480	0.1428	1.261	0.925	1.718
H_DRINK_H01	1	1	0.09316	0.08240	1.2781	0.2583	1.098	0.934	1.290
H_SMK_C01	1	1	-0.03567	0.06725	0.2813	0.5958	0.965	0.846	1.101
IT	1	1	0.03772	0.08183	0.2124	0.6449	1.038	0.885	1.219
ET	1	1	0.61175	0.24986	5.9946	0.0143	1.844	1.130	3.009
IMG_C_TOAST	5	1	-0.33023	0.06150	28.8340	<.0001	0.719	0.637	0.811
IMG_C_TOAST	4	1	-0.02407	0.22330	0.0116	0.9141	0.976	0.630	1.512
IMG_C_TOAST	3	1	-0.52953	0.07916	44.7522	<.0001	0.589	0.504	0.688
IMG_C_TOAST	2	1	-0.42385	0.13555	9.7780	0.0018	0.655	0.502	0.854
A_NIHSS		1	0.02276	0.00571	15.9017	<.0001	1.023	1.012	1.035

BMI_g with y1_comb: interaction

PHREG 过程

最大似然估计分析		
参数		标签
BMI_g	4	1=<18.5;2=18.5-<23;3=23-<27.5;4= ≥ 27.5 4
BMI_g	3	1=<18.5;2=18.5-<23;3=23-<27.5;4= ≥ 27.5 3
BMI_g	1	1=<18.5;2=18.5-<23;3=23-<27.5;4= ≥ 27.5 1
GENDER		A.Basic Information: Gender; 1-male; 2-female;
GENDER*BMI_g	4	1=<18.5;2=18.5-<23;3=23-<27.5;4= ≥ 27.5 4 * A.Basic Information: Gender; 1-male; 2-female;
GENDER*BMI_g	3	1=<18.5;2=18.5-<23;3=23-<27.5;4= ≥ 27.5 3 * A.Basic Information: Gender; 1-male; 2-female;
GENDER*BMI_g	1	1=<18.5;2=18.5-<23;3=23-<27.5;4= ≥ 27.5 1 * A.Basic Information: Gender; 1-male; 2-female;
AGE		A.Basic Information: Age (years old);
ETHNIC	2	B.Demography: Race: 1-Han; 99-others; 2
H_DIAB01	1	D.History: Diabetes; 0-No; 1-Yes; 1
H_AF01	1	D.History: Heart disease category: Atrial fibrillation(Including medical history and hospitalization diagnosis); 0-No; 1-Yes; 1
H_HYPT01	1	D.History: Hypertension; 0-No; 1-Yes; 1
H_LIPID01	1	D.History: Lipid metabolism disorders; 0-No; 1-Yes; 1
AI	1	history:Myocardial infarction; 0=NO; 1=YES; 1
H_DRINK_H01	1	D.History: Heavy Drinking(Alcohol consumption>=20g/day); 0-No,1-Yes; 1
H_SMK_C01	1	D.History: Current Smoking; 0-No,1-Yes; 1
IT	1	intravenous thrombolysis, 1=YES,0=NO 1
ET	1	动脉溶栓或机械取栓, 1=YES,0=NO 1
IMG_C_TOAST	5	K.Final diagnosis: cerebral infarction; Etiology according to TOAST system; 1-large artery atherosclerosis; 2-cardiogenic embolism; 3-small artery occlusion; 4-stroke of another determined cause; 5-stroke of an undetermined cause. 5
IMG_C_TOAST	4	K.Final diagnosis: cerebral infarction; Etiology according to TOAST system; 1-large artery atherosclerosis; 2-cardiogenic embolism; 3-small artery occlusion; 4-stroke of another determined cause; 5-stroke of an undetermined cause. 4
IMG_C_TOAST	3	K.Final diagnosis: cerebral infarction; Etiology according to TOAST system; 1-large artery atherosclerosis; 2-cardiogenic embolism; 3-small artery occlusion; 4-stroke of another determined cause; 5-stroke of an undetermined cause. 3
IMG_C_TOAST	2	K.Final diagnosis: cerebral infarction; Etiology according to TOAST system; 1-large artery atherosclerosis; 2-cardiogenic embolism; 3-small artery occlusion; 4-stroke of another determined cause; 5-stroke of an undetermined cause. 2
A_NIHSS		F.Admitting NIHSS: Total score;

male: BMI_g with y1_stroke: Descriptive results

FREQ 过程

频数<br>行百分比

BMI_g-y1_stroke表			
	y1_stroke(N12.Follow-up events at 12 months: Recurrence of stroke: 0-No; 1-Yes;)		
BMI_g(1=<18.5;2=18.5-<23;3=23-<27.5;4=≥27.5)	0	1	合计
1	160<br>90.40	17<br>9.60	177
2	2348<br>90.34	251<br>9.66	2599
3	4795<br>90.42	508<br>9.58	5303
4	1471<br>89.64	170<br>10.36	1641
合计	8774	946	9720

表“y1_stroke-BMI_g”的统计量

统计量	自由度	值	概率
卡方	3	0.8957	0.8265
似然比卡方检验	3	0.8827	0.8296
Mantel-Haenszel 卡方	1	0.3842	0.5354
Phi 系数		0.0096	
列联系数		0.0096	
Cramer V		0.0096	

样本大小 = 9720

male: BMI_g with y1_stroke: adjusted model

PHREG 过程

模型信息		
数据集	WORK.MALE	
因变量	y1_stroke_dd	N12.Follow-up events at 12 months: Days from onset to recurrence;(day);
删失变量	y1_stroke	N12.Follow-up events at 12 months: Recurrence of stroke: 0-No; 1-Yes;
删失值	0	
结值处理	BRESLOW	

读取的观测数<br>使用的观测数	9720<br>9720

分类水平信息					
分类	值	设计变量			
BMI_g	4	1	0	0	
	3	0	1	0	
	2	0	0	0	
	1	0	0	1	
ETHNIC	2	1			
	1	0			
H_DIAB01	1	1			
	0	0			
H_AF01	1	1			
	0	0			
H_HYPT01	1	1			
	0	0			
H_LIPID01	1	1			
	0	0			
AI	1	1			
	0	0			
H_DRINK_H01	1	1			
	0	0			
H_SMK_C01	1	1			
	0	0			
IT	1	1			
	0	0			
ET	1	1			
	0	0			
IMG_C_TOAST	5	1	0	0	0
	4	0	1	0	0
	3	0	0	1	0
	2	0	0	0	1
	1	0	0	0	0

male: BMI_g with y1_stroke: adjusted model

PHREG 过程

事件和删失值个数汇总			
合计	事件	删失	删失百分比
9720	946	8774	90.27

收敛状态
满足收敛准则 (GCONV=1E-8)。

模型拟合统计量		
准则	无协变量	带协变量
-2 LOG L	17246.103	17142.012
AIC	17246.103	17180.012
SBC	17246.103	17272.205

检验全局原假设: BETA=0			
检验	卡方	自由度	Pr > 卡方
似然比	104.0910	19	<.0001
评分	111.9011	19	<.0001
Wald	109.1093	19	<.0001

3 型检验			
效应	自由度	Wald 卡方	Pr > 卡方
BMI_g	3	1.4515	0.6935
AGE	1	6.1528	0.0131
ETHNIC	1	0.0140	0.9059
H_DIAB01	1	9.2109	0.0024
H_AF01	1	2.2564	0.1331
H_HYPT01	1	1.1172	0.2905
H_LIPID01	1	0.3574	0.5499
AI	1	0.3603	0.5483
H_DRINK_H01	1	3.6726	0.0553
H_SMK_C01	1	2.4511	0.1174
IT	1	0.1596	0.6896
ET	1	5.1953	0.0226
IMG_C_TOAST	4	37.0652	<.0001
A_NIHSS	1	13.8682	0.0002

male: BMI_g with y1_stroke: adjusted model

PHREG 过程

最大似然估计分析									
参数		自由度	参数估计	标准误差	卡方	Pr > 卡方	危险率	95% 危险率置信限	
BMI_g	4	1	0.10768	0.10219	1.1105	0.2920	1.114	0.912	1.361
BMI_g	3	1	0.00988	0.07822	0.0159	0.8995	1.010	0.866	1.177
BMI_g	1	1	-0.04666	0.25142	0.0344	0.8528	0.954	0.583	1.562
AGE		1	0.00775	0.00313	6.1528	0.0131	1.008	1.002	1.014
ETHNIC	2	1	-0.02274	0.19229	0.0140	0.9059	0.978	0.671	1.425
H_DIAB01	1	1	0.23080	0.07605	9.2109	0.0024	1.260	1.085	1.462
H_AF01	1	1	0.24628	0.16395	2.2564	0.1331	1.279	0.928	1.764
H_HYPT01	1	1	0.07289	0.06896	1.1172	0.2905	1.076	0.940	1.231
H_LIPID01	1	1	-0.07462	0.12482	0.3574	0.5499	0.928	0.727	1.185
AI	1	1	0.12030	0.20041	0.3603	0.5483	1.128	0.761	1.670
H_DRINK_H01	1	1	0.16213	0.08460	3.6726	0.0553	1.176	0.996	1.388
H_SMK_C01	1	1	-0.11394	0.07278	2.4511	0.1174	0.892	0.774	1.029
IT	1	1	0.04079	0.10213	0.1596	0.6896	1.042	0.853	1.272
ET	1	1	0.70535	0.30946	5.1953	0.0226	2.025	1.104	3.713
IMG_C_TOAST	5	1	-0.34416	0.07680	20.0825	<.0001	0.709	0.610	0.824
IMG_C_TOAST	4	1	-0.00204	0.30723	0.0000	0.9947	0.998	0.547	1.822
IMG_C_TOAST	3	1	-0.54516	0.09818	30.8315	<.0001	0.580	0.478	0.703
IMG_C_TOAST	2	1	-0.24616	0.18012	1.8676	0.1717	0.782	0.549	1.113
A_NIHSS		1	0.02696	0.00724	13.8682	0.0002	1.027	1.013	1.042

male: BMI_g with y1_stroke: adjusted model

PHREG 过程

最大似然估计分析		
参数		标签
BMI_g	4	1=<18.5;2=18.5-<23;3=23-<27.5;4=≥27.5 4
BMI_g	3	1=<18.5;2=18.5-<23;3=23-<27.5;4=≥27.5 3
BMI_g	1	1=<18.5;2=18.5-<23;3=23-<27.5;4=≥27.5 1
AGE		A.Basic Information: Age (years old);
ETHNIC	2	B.Demography: Race: 1-Han; 99-others; 2
H_DIAB01	1	D.History: Diabetes; 0-No; 1-Yes; 1
H_AF01	1	D.History: Heart disease category: Atrial fibrillation(Including medical history and hospitalization diagnosis); 0-No; 1-Yes; 1
H_HYPT01	1	D.History: Hypertension; 0-No; 1-Yes; 1
H_LIPID01	1	D.History: Lipid metabolism disorders; 0-No; 1-Yes; 1
AI	1	history:Myocardial infarction; 0=NO; 1=YES; 1
H_DRINK_H01	1	D.History: Heavy Drinking(Alcohol consumption>=20g/day); 0-No,1-Yes; 1
H_SMK_C01	1	D.History: Current Smoking; 0-No,1-Yes; 1
IT	1	intravenous thrombolysis, 1=YES,0=NO 1
ET	1	动脉溶栓或机械取栓, 1=YES,0=NO 1
IMG_C_TOAST	5	K.Final diagnosis: cerebral infarction; Etiology according to TOAST system; 1-large artery atherosclerosis; 2-cardiogenic embolism; 3-small artery occlusion; 4-stroke of another determined cause; 5-stroke of an undetermined cause. 5
IMG_C_TOAST	4	K.Final diagnosis: cerebral infarction; Etiology according to TOAST system; 1-large artery atherosclerosis; 2-cardiogenic embolism; 3-small artery occlusion; 4-stroke of another determined cause; 5-stroke of an undetermined cause. 4
IMG_C_TOAST	3	K.Final diagnosis: cerebral infarction; Etiology according to TOAST system; 1-large artery atherosclerosis; 2-cardiogenic embolism; 3-small artery occlusion; 4-stroke of another determined cause; 5-stroke of an undetermined cause. 3
IMG_C_TOAST	2	K.Final diagnosis: cerebral infarction; Etiology according to TOAST system; 1-large artery atherosclerosis; 2-cardiogenic embolism; 3-small artery occlusion; 4-stroke of another determined cause; 5-stroke of an undetermined cause. 2
A_NIHSS		F.Admitting NIHSS: Total score;

BMI_g with y1_stroke: interaction

PHREG 过程

模型信息		
数据集	WORK.DATA_OVERALL	
因变量	y1_stroke_dd	N12.Follow-up events at 12 months: Days from onset to recurrence;(day);
删失变量	y1_stroke	N12.Follow-up events at 12 months: Recurrence of stroke: 0-No; 1-Yes;
删失值	0	
结值处理	BRESLOW	

读取的观测数<br>使用的观测数	14146<br>14146

分类水平信息						
分类	值	设计变量				
BMI_g	4	1	0	0	
	3	0	1	0	
	2	0	0	0	
	1	0	0	1	
ETHNIC	2	1			
	1	0			
H_DIAB01	1	1			
	0	0			
H_AF01	1	1			
	0	0			
H_HYPT01	1	1			
	0	0			
H_LIPID01	1	1			
	0	0			
AI	1	1			
	0	0			
H_DRINK_H01	1	1			
	0	0			
H_SMK_C01	1	1			
	0	0			
IT	1	1			
	0	0			
ET	1	1			
	0	0			
IMG_C_TOAST	5	1	0	0	0
	4	0	1	0	0
	3	0	0	1	0
	2	0	0	0	1
	1	0	0	0	0

BMI_g with y1_stroke: interaction

PHREG 过程

事件和删失值个数汇总			
合计	事件	删失	删失百分比
14146	1424	12722	89.93

收敛状态
满足收敛准则 (GCONV=1E-8)。

模型拟合统计量		
准则	无协变量	带协变量
-2 LOG L	27024.892	26876.607
AIC	27024.892	26922.607
SBC	27024.892	27043.616

检验全局原假设: BETA=0			
检验	卡方	自由度	Pr > 卡方
似然比	148.2847	23	<.0001
评分	157.3531	23	<.0001
Wald	154.0667	23	<.0001

联合检验			
效应	自由度	Wald 卡方	Pr > 卡方
BMI_g	3	1.1676	0.7608
GENDER	1	0.0120	0.9129
GENDER*BMI_g	3	1.2310	0.7456
AGE	1	15.1460	<.0001
ETHNIC	1	0.6311	0.4270
H_DIAB01	1	11.9198	0.0006
H_AF01	1	8.3774	0.0038
H_HYPT01	1	1.3225	0.2501
H_LIPID01	1	0.5953	0.4404
AI	1	1.0313	0.3099
H_DRINK_H01	1	2.9756	0.0845
H_SMK_C01	1	0.7192	0.3964
IT	1	1.0128	0.3142
ET	1	5.8682	0.0154
IMG_C_TOAST	4	52.9659	<.0001
A_NIHSS	1	12.5836	0.0004

Note: Under full-rank parameterizations, Type 3 effect tests are replaced by joint tests. The joint test for an effect is a test that all of the parameters associated with that effect are zero. Such joint tests might not be equivalent to Type 3 effect tests under GLM parameterization.

BMI_g with y1_stroke: interaction

PHREG 过程

最大似然估计分析									
参数		自由度	参数估计	标准误差	卡方	Pr > 卡方	危险率	95% 危险率置信限	
BMI_g	4	1	0.12574	0.24156	0.2710	0.6027	.	.	.
BMI_g	3	1	-0.02883	0.18953	0.0231	0.8791	.	.	.
BMI_g	1	1	-0.43117	0.55884	0.5953	0.4404	.	.	.
GENDER		1	0.01217	0.11126	0.0120	0.9129	.	.	.
GENDER*BMI_g	4	1	-0.00524	0.16771	0.0010	0.9751	.	.	.
GENDER*BMI_g	3	1	0.04337	0.13384	0.1050	0.7459	.	.	.
GENDER*BMI_g	1	1	0.37280	0.35134	1.1259	0.2887	.	.	.
AGE		1	0.00998	0.00256	15.1460	<.0001	1.010	1.005	1.015
ETHNIC	2	1	-0.13264	0.16697	0.6311	0.4270	0.876	0.631	1.215
H_DIAB01	1	1	0.20985	0.06078	11.9198	0.0006	1.233	1.095	1.390
H_AF01	1	1	0.35321	0.12203	8.3774	0.0038	1.424	1.121	1.808
H_HYPT01	1	1	0.06581	0.05722	1.3225	0.2501	1.068	0.955	1.195
H_LIPID01	1	1	-0.07910	0.10252	0.5953	0.4404	0.924	0.756	1.130
AI	1	1	0.17069	0.16808	1.0313	0.3099	1.186	0.853	1.649
H_DRINK_H01	1	1	0.14444	0.08374	2.9756	0.0845	1.155	0.981	1.361
H_SMK_C01	1	1	-0.05871	0.06923	0.7192	0.3964	0.943	0.823	1.080
IT	1	1	0.08366	0.08313	1.0128	0.3142	1.087	0.924	1.280
ET	1	1	0.62396	0.25758	5.8682	0.0154	1.866	1.127	3.092
IMG_C_TOAST	5	1	-0.33808	0.06300	28.8004	<.0001	0.713	0.630	0.807
IMG_C_TOAST	4	1	-0.03466	0.22881	0.0230	0.8796	0.966	0.617	1.513
IMG_C_TOAST	3	1	-0.52896	0.08089	42.7567	<.0001	0.589	0.503	0.690
IMG_C_TOAST	2	1	-0.44622	0.14210	9.8606	0.0017	0.640	0.484	0.846
A_NIHSS		1	0.02105	0.00593	12.5836	0.0004	1.021	1.009	1.033

BMI_g with y1_stroke: interaction

PHREG 过程

最大似然估计分析		
参数		标签
BMI_g	4	1=<18.5;2=18.5-<23;3=23-<27.5;4=≥27.5 4
BMI_g	3	1=<18.5;2=18.5-<23;3=23-<27.5;4=≥27.5 3
BMI_g	1	1=<18.5;2=18.5-<23;3=23-<27.5;4=≥27.5 1
GENDER		A.Basic Information: Gender; 1-male; 2-female;
GENDER*BMI_g	4	1=<18.5;2=18.5-<23;3=23-<27.5;4=≥27.5 4 * A.Basic Information: Gender; 1-male; 2-female;
GENDER*BMI_g	3	1=<18.5;2=18.5-<23;3=23-<27.5;4=≥27.5 3 * A.Basic Information: Gender; 1-male; 2-female;
GENDER*BMI_g	1	1=<18.5;2=18.5-<23;3=23-<27.5;4=≥27.5 1 * A.Basic Information: Gender; 1-male; 2-female;
AGE		A.Basic Information: Age (years old);
ETHNIC	2	B.Demography: Race: 1-Han; 99-others; 2
H_DIAB01	1	D.History: Diabetes; 0-No; 1-Yes; 1
H_AF01	1	D.History: Heart disease category: Atrial fibrillation(Including medical history and hospitalization diagnosis); 0-No; 1-Yes; 1
H_HYPT01	1	D.History: Hypertension; 0-No; 1-Yes; 1
H_LIPID01	1	D.History: Lipid metabolism disorders; 0-No; 1-Yes; 1
AI	1	history:Myocardial infarction; 0=NO; 1=YES; 1
H_DRINK_H01	1	D.History: Heavy Drinking(Alcohol consumption>=20g/day); 0-No,1-Yes; 1
H_SMK_C01	1	D.History: Current Smoking; 0-No,1-Yes; 1
IT	1	intravenous thrombolysis, 1=YES,0=NO 1
ET	1	动脉溶栓或机械取栓, 1=YES,0=NO 1
IMG_C_TOAST	5	K.Final diagnosis: cerebral infarction; Etiology according to TOAST system; 1-large artery atherosclerosis; 2-cardiogenic embolism; 3-small artery occlusion; 4-stroke of another determined cause; 5-stroke of an undetermined cause. 5
IMG_C_TOAST	4	K.Final diagnosis: cerebral infarction; Etiology according to TOAST system; 1-large artery atherosclerosis; 2-cardiogenic embolism; 3-small artery occlusion; 4-stroke of another determined cause; 5-stroke of an undetermined cause. 4
IMG_C_TOAST	3	K.Final diagnosis: cerebral infarction; Etiology according to TOAST system; 1-large artery atherosclerosis; 2-cardiogenic embolism; 3-small artery occlusion; 4-stroke of another determined cause; 5-stroke of an undetermined cause. 3
IMG_C_TOAST	2	K.Final diagnosis: cerebral infarction; Etiology according to TOAST system; 1-large artery atherosclerosis; 2-cardiogenic embolism; 3-small artery occlusion; 4-stroke of another determined cause; 5-stroke of an undetermined cause. 2
A_NIHSS		F.Admitting NIHSS: Total score;

female: BMI_g with y1_death: Descriptive results

FREQ 过程

频数<br>行百分比

BMI_g-y1_death表			
	y1_death(N12.Follow-up events at 12 months: Whether the patient died: 0-survival;1-death;)		
BMI_g(1=<18.5;2=18.5-<23;3=23-<27.5;4=≥27.5)	0	1	合计
1	113<br>85.61	19<br>14.39	132
2	1197<br>95.30	59<br>4.70	1256
3	2111<br>96.48	77<br>3.52	2188
4	827<br>97.29	23<br>2.71	850
合计	4248	178	4426

表“y1_death-BMI_g”的统计量

统计量	自由度	值	概率
卡方	3	43.5205	<.0001
似然比卡方检验	3	29.8046	<.0001
Mantel-Haenszel 卡方	1	22.6445	<.0001
Phi 系数		0.0992	
列联系数		0.0987	
Cramer V		0.0992	

样本大小 = 4426

female: BMI_g with y1_death: adjusted model

PHREG 过程

模型信息		
数据集	WORK.FEMALE	
因变量	y1_death_dd	N12.Follow-up events at 12 months: Days from onset to death;(day);
删失变量	y1_death	N12.Follow-up events at 12 months: Whether the patient died: 0-survival;1-death;
删失值	0	
结值处理	BRESLOW	

读取的观测数<br>使用的观测数	4426<br>4426

分类水平信息					
分类	值	设计变量			
BMI_g	4	1	0	0	
	3	0	1	0	
	2	0	0	0	
	1	0	0	1	
ETHNIC	2	1			
	1	0			
H_DIAB01	1	1			
	0	0			
H_AF01	1	1			
	0	0			
H_HYPT01	1	1			
	0	0			
H_LIPID01	1	1			
	0	0			
AI	1	1			
	0	0			
H_DRINK_H01	1	1			
	0	0			
H_SMK_C01	1	1			
	0	0			
IT	1	1			
	0	0			
ET	1	1			
	0	0			
IMG_C_TOAST	5	1	0	0	0
	4	0	1	0	0
	3	0	0	1	0
	2	0	0	0	1
	1	0	0	0	0

female: BMI_g with y1_death: adjusted model

PHREG 过程

事件和删失值个数汇总			
合计	事件	删失	删失百分比
4426	178	4248	95.98

收敛状态
满足收敛准则 (GCONV=1E-8)。

模型拟合统计量		
准则	无协变量	带协变量
-2 LOG L	2976.098	2718.597
AIC	2976.098	2756.597
SBC	2976.098	2817.051

检验全局原假设: BETA=0			
检验	卡方	自由度	Pr > 卡方
似然比	257.5013	19	<.0001
评分	351.8088	19	<.0001
Wald	293.1410	19	<.0001

3 型检验			
效应	自由度	Wald 卡方	Pr > 卡方
BMI_g	3	13.0982	0.0044
AGE	1	57.9335	<.0001
ETHNIC	1	1.2101	0.2713
H_DIAB01	1	2.9524	0.0857
H_AF01	1	12.0280	0.0005
H_HYPT01	1	0.0714	0.7893
H_LIPID01	1	2.2203	0.1362
AI	1	6.7355	0.0095
H_DRINK_H01	1	0.0015	0.9687
H_SMK_C01	1	9.8400	0.0017
IT	1	9.6152	0.0019
ET	1	0.3628	0.5470
IMG_C_TOAST	4	11.8486	0.0185
A_NIHSS	1	69.1424	<.0001

female: BMI_g with y1_death: adjusted model

PHREG 过程

最大似然估计分析									
参数		自由度	参数估计	标准误差	卡方	Pr > 卡方	危险率	95% 危险率置信限	
BMI_g	4	1	-0.23681	0.25239	0.8804	0.3481	0.789	0.481	1.294
BMI_g	3	1	0.00856	0.17845	0.0023	0.9617	1.009	0.711	1.431
BMI_g	1	1	0.84236	0.26907	9.8009	0.0017	2.322	1.370	3.934
AGE		1	0.06410	0.00842	57.9335	<.0001	1.066	1.049	1.084
ETHNIC	2	1	0.42981	0.39072	1.2101	0.2713	1.537	0.715	3.306
H_DIAB01	1	1	0.29979	0.17448	2.9524	0.0857	1.350	0.959	1.900
H_AF01	1	1	0.80519	0.23217	12.0280	0.0005	2.237	1.419	3.526
H_HYPT01	1	1	-0.04417	0.16529	0.0714	0.7893	0.957	0.692	1.323
H_LIPID01	1	1	-0.54646	0.36673	2.2203	0.1362	0.579	0.282	1.188
AI	1	1	0.91157	0.35124	6.7355	0.0095	2.488	1.250	4.953
H_DRINK_H01	1	1	-10.85358	276.78037	0.0015	0.9687	0.000	0.000	7.62E230
H_SMK_C01	1	1	0.96251	0.30684	9.8400	0.0017	2.618	1.435	4.777
IT	1	1	-0.90324	0.29129	9.6152	0.0019	0.405	0.229	0.717
ET	1	1	0.44022	0.73087	0.3628	0.5470	1.553	0.371	6.506
IMG_C_TOAST	5	1	-0.27476	0.19276	2.0318	0.1540	0.760	0.521	1.109
IMG_C_TOAST	4	1	0.56317	0.52119	1.1676	0.2799	1.756	0.632	4.878
IMG_C_TOAST	3	1	-0.97454	0.32244	9.1350	0.0025	0.377	0.201	0.710
IMG_C_TOAST	2	1	-0.31199	0.29820	1.0946	0.2955	0.732	0.408	1.313
A_NIHSS		1	0.08648	0.01040	69.1424	<.0001	1.090	1.068	1.113

female: BMI_g with y1_death: adjusted model

PHREG 过程

最大似然估计分析		
参数		标签
BMI_g	4	1=<18.5;2=18.5-<23;3=23-<27.5;4= ≥ 27.5 4
BMI_g	3	1=<18.5;2=18.5-<23;3=23-<27.5;4= ≥ 27.5 3
BMI_g	1	1=<18.5;2=18.5-<23;3=23-<27.5;4= ≥ 27.5 1
AGE		A.Basic Information: Age (years old);
ETHNIC	2	B.Demography: Race: 1-Han; 99-others; 2
H_DIAB01	1	D.History: Diabetes; 0-No; 1-Yes; 1
H_AF01	1	D.History: Heart disease category: Atrial fibrillation(Including medical history and hospitalization diagnosis); 0-No; 1-Yes; 1
H_HYPT01	1	D.History: Hypertension; 0-No; 1-Yes; 1
H_LIPID01	1	D.History: Lipid metabolism disorders; 0-No; 1-Yes; 1
AI	1	history:Myocardial infarction; 0=NO; 1=YES; 1
H_DRINK_H01	1	D.History: Heavy Drinking(Alcohol consumption>=20g/day); 0-No,1-Yes; 1
H_SMK_C01	1	D.History: Current Smoking; 0-No,1-Yes; 1
IT	1	intravenous thrombolysis, 1=YES,0=NO 1
ET	1	动脉溶栓或机械取栓, 1=YES,0=NO 1
IMG_C_TOAST	5	K.Final diagnosis: cerebral infarction; Etiology according to TOAST system; 1-large artery atherosclerosis; 2-cardiogenic embolism; 3-small artery occlusion; 4-stroke of another determined cause; 5-stroke of an undetermined cause. 5
IMG_C_TOAST	4	K.Final diagnosis: cerebral infarction; Etiology according to TOAST system; 1-large artery atherosclerosis; 2-cardiogenic embolism; 3-small artery occlusion; 4-stroke of another determined cause; 5-stroke of an undetermined cause. 4
IMG_C_TOAST	3	K.Final diagnosis: cerebral infarction; Etiology according to TOAST system; 1-large artery atherosclerosis; 2-cardiogenic embolism; 3-small artery occlusion; 4-stroke of another determined cause; 5-stroke of an undetermined cause. 3
IMG_C_TOAST	2	K.Final diagnosis: cerebral infarction; Etiology according to TOAST system; 1-large artery atherosclerosis; 2-cardiogenic embolism; 3-small artery occlusion; 4-stroke of another determined cause; 5-stroke of an undetermined cause. 2
A_NIHSS		F.Admitting NIHSS: Total score;

BMI_g with y1_death: interaction

PHREG 过程

模型信息		
数据集	WORK.DATA_OVERALL	
因变量	y1_death_dd	N12.Follow-up events at 12 months: Days from onset to death;(day);
删失变量	y1_death	N12.Follow-up events at 12 months: Whether the patient died: 0-survival;1-death;
删失值	0	
结值处理	BRESLOW	

读取的观测数<br>使用的观测数	14146<br>14146

分类水平信息						
分类	值	设计变量				
BMI_g	4	1	0	0	
	3	0	1	0	
	2	0	0	0	
	1	0	0	1	
ETHNIC	2	1			
	1	0			
H_DIAB01	1	1			
	0	0			
H_AF01	1	1			
	0	0			
H_HYPT01	1	1			
	0	0			
H_LIPID01	1	1			
	0	0			
AI	1	1			
	0	0			
H_DRINK_H01	1	1			
	0	0			
H_SMK_C01	1	1			
	0	0			
IT	1	1			
	0	0			
ET	1	1			
	0	0			
IMG_C_TOAST	5	1	0	0	0
	4	0	1	0	0
	3	0	0	1	0
	2	0	0	0	1
	1	0	0	0	0

BMI_g with y1_death: interaction

PHREG 过程

事件和删失值个数汇总			
合计	事件	删失	删失百分比
14146	486	13660	96.56

收敛状态
满足收敛准则 (GCONV=1E-8)。

模型拟合统计量		
准则	无协变量	带协变量
-2 LOG L	9255.945	8606.088
AIC	9255.945	8652.088
SBC	9255.945	8748.371

检验全局原假设: BETA=0			
检验	卡方	自由度	Pr > 卡方
似然比	649.8566	23	<.0001
评分	891.6098	23	<.0001
Wald	758.4266	23	<.0001

联合检验			
效应	自由度	Wald 卡方	Pr > 卡方
BMI_g	3	1.1775	0.7584
GENDER	1	1.8258	0.1766
GENDER*BMI_g	3	3.0076	0.3904
AGE	1	150.7508	<.0001
ETHNIC	1	3.0866	0.0789
H_DIAB01	1	12.8226	0.0003
H_AF01	1	28.8683	<.0001
H_HYPT01	1	0.1198	0.7293
H_LIPID01	1	2.3003	0.1294
AI	1	5.3780	0.0204
H_DRINK_H01	1	6.9570	0.0083
H_SMK_C01	1	0.9710	0.3244
IT	1	19.8678	<.0001
ET	1	7.6676	0.0056
IMG_C_TOAST	4	34.8224	<.0001
A_NIHSS	1	226.3090	<.0001

Note: Under full-rank parameterizations, Type 3 effect tests are replaced by joint tests. The joint test for an effect is a test that all of the parameters associated with that effect are zero. Such joint tests might not be equivalent to Type 3 effect tests under GLM parameterization.

BMI_g with y1_death: interaction

PHREG 过程

最大似然估计分析									
参数		自由度	参数估计	标准误差	卡方	Pr > 卡方	危险率	95% 危险率置信限	
BMI_g	4	1	-0.41353	0.46907	0.7772	0.3780	.	.	.
BMI_g	3	1	-0.17392	0.30735	0.3202	0.5715	.	.	.
BMI_g	1	1	-0.50088	0.64834	0.5968	0.4398	.	.	.
GENDER		1	-0.22824	0.16892	1.8258	0.1766	.	.	.
GENDER*BMI_g	4	1	0.10072	0.31694	0.1010	0.7507	.	.	.
GENDER*BMI_g	3	1	0.08469	0.21498	0.1552	0.6936	.	.	.
GENDER*BMI_g	1	1	0.68533	0.39686	2.9821	0.0842	.	.	.
AGE		1	0.05908	0.00481	150.7508	<.0001	1.061	1.051	1.071
ETHNIC	2	1	0.41434	0.23584	3.0866	0.0789	1.513	0.953	2.403
H_DIAB01	1	1	0.37403	0.10445	12.8226	0.0003	1.454	1.184	1.784
H_AF01	1	1	0.80187	0.14924	28.8683	<.0001	2.230	1.664	2.987
H_HYPT01	1	1	0.03382	0.09772	0.1198	0.7293	1.034	0.854	1.253
H_LIPID01	1	1	-0.31533	0.20791	2.3003	0.1294	0.730	0.485	1.097
AI	1	1	0.52512	0.22644	5.3780	0.0204	1.691	1.085	2.635
H_DRINK_H01	1	1	-0.51671	0.19590	6.9570	0.0083	0.596	0.406	0.876
H_SMK_C01	1	1	0.12248	0.12430	0.9710	0.3244	1.130	0.886	1.442
IT	1	1	-0.74102	0.16625	19.8678	<.0001	0.477	0.344	0.660
ET	1	1	0.86759	0.31332	7.6676	0.0056	2.381	1.289	4.400
IMG_C_TOAST	5	1	-0.24185	0.11139	4.7141	0.0299	0.785	0.631	0.977
IMG_C_TOAST	4	1	0.65361	0.32740	3.9854	0.0459	1.922	1.012	3.652
IMG_C_TOAST	3	1	-0.92963	0.18262	25.9125	<.0001	0.395	0.276	0.565
IMG_C_TOAST	2	1	-0.42431	0.19330	4.8185	0.0282	0.654	0.448	0.956
A_NIHSS		1	0.09550	0.00635	226.3090	<.0001	1.100	1.087	1.114

BMI_g with y1_death: interaction

PHREG 过程

最大似然估计分析		
参数		标签
BMI_g	4	1=<18.5;2=18.5-<23;3=23-<27.5;4= ≥ 27.5 4
BMI_g	3	1=<18.5;2=18.5-<23;3=23-<27.5;4= ≥ 27.5 3
BMI_g	1	1=<18.5;2=18.5-<23;3=23-<27.5;4= ≥ 27.5 1
GENDER		A.Basic Information: Gender; 1-male; 2-female;
GENDER*BMI_g	4	1=<18.5;2=18.5-<23;3=23-<27.5;4= ≥ 27.5 4 * A.Basic Information: Gender; 1-male; 2-female;
GENDER*BMI_g	3	1=<18.5;2=18.5-<23;3=23-<27.5;4= ≥ 27.5 3 * A.Basic Information: Gender; 1-male; 2-female;
GENDER*BMI_g	1	1=<18.5;2=18.5-<23;3=23-<27.5;4= ≥ 27.5 1 * A.Basic Information: Gender; 1-male; 2-female;
AGE		A.Basic Information: Age (years old);
ETHNIC	2	B.Demography: Race: 1-Han; 99-others; 2
H_DIAB01	1	D.History: Diabetes; 0-No; 1-Yes; 1
H_AF01	1	D.History: Heart disease category: Atrial fibrillation(Including medical history and hospitalization diagnosis); 0-No; 1-Yes; 1
H_HYPT01	1	D.History: Hypertension; 0-No; 1-Yes; 1
H_LIPID01	1	D.History: Lipid metabolism disorders; 0-No; 1-Yes; 1
AI	1	history:Myocardial infarction; 0=NO; 1=YES; 1
H_DRINK_H01	1	D.History: Heavy Drinking(Alcohol consumption>=20g/day); 0-No,1-Yes; 1
H_SMK_C01	1	D.History: Current Smoking; 0-No,1-Yes; 1
IT	1	intravenous thrombolysis, 1=YES,0=NO 1
ET	1	动脉溶栓或机械取栓, 1=YES,0=NO 1
IMG_C_TOAST	5	K.Final diagnosis: cerebral infarction; Etiology according to TOAST system; 1-large artery atherosclerosis; 2-cardiogenic embolism; 3-small artery occlusion; 4-stroke of another determined cause; 5-stroke of an undetermined cause. 5
IMG_C_TOAST	4	K.Final diagnosis: cerebral infarction; Etiology according to TOAST system; 1-large artery atherosclerosis; 2-cardiogenic embolism; 3-small artery occlusion; 4-stroke of another determined cause; 5-stroke of an undetermined cause. 4
IMG_C_TOAST	3	K.Final diagnosis: cerebral infarction; Etiology according to TOAST system; 1-large artery atherosclerosis; 2-cardiogenic embolism; 3-small artery occlusion; 4-stroke of another determined cause; 5-stroke of an undetermined cause. 3
IMG_C_TOAST	2	K.Final diagnosis: cerebral infarction; Etiology according to TOAST system; 1-large artery atherosclerosis; 2-cardiogenic embolism; 3-small artery occlusion; 4-stroke of another determined cause; 5-stroke of an undetermined cause. 2
A_NIHSS		F.Admitting NIHSS: Total score;

female: BMI_g with y1_comb: Descriptive results

FREQ 过程

频数
行百分比

BMI_g-y1_comb表

BMI_g(1=<18.5;2=18.5-<23;3=23-<27.5;4= ≥ 27.5)	y1_comb(N12.Follow-up events at 12 months:Occurrence of combined vascular event(including cardiovascular death,non-fatal stroke,non-fatal myocardial infarction):0-No;1-Yes;)		
	0	1	合计
1	112<br>84.85	20<br>15.15	132
2	1116<br>88.85	140<br>11.15	1256
3	1943<br>88.80	245<br>11.20	2188
4	752<br>88.47	98<br>11.53	850
合计	3923	503	4426

表“y1_comb-BMI_g”的统计量

统计量	自由度	值	概率
卡方	3	2.0222	0.5678
似然比卡方检验	3	1.8665	0.6006
Mantel-Haenszel 卡方	1	0.1344	0.7139
Phi 系数		0.0214	
列联系数		0.0214	
Cramer V		0.0214	

样本大小 = 4426

female: BMI_g with y1_comb: adjusted model

PHREG 过程

模型信息		
数据集	WORK.FEMALE	
因变量	y1_comb_dd	N12.Follow-up events at 12 months: Days from onset to occurrence of combined vascular event;(day);
删失变量	y1_comb	N12.Follow-up events at 12 months:Occurrence of combined vascular event(including cardiovascular death,non-fatal stroke,non-fatal myocardial infarction):0-No;1-Yes;
删失值	0	
结值处理	BRESLOW	

读取的观测数	4426
使用的观测数	4426

分类水平信息					
分类	值	设计变量			
BMI_g	4	1	0	0	
	3	0	1	0	
	2	0	0	0	
	1	0	0	1	
ETHNIC	2	1			
	1	0			
H_DIAB01	1	1			
	0	0			
H_AF01	1	1			
	0	0			
H_HYPT01	1	1			
	0	0			
H_LIPID01	1	1			
	0	0			
AI	1	1			
	0	0			
H_DRINK_H01	1	1			
	0	0			
H_SMK_C01	1	1			
	0	0			
IT	1	1			
	0	0			
ET	1	1			
	0	0			
IMG_C_TOAST	5	1	0	0	0
	4	0	1	0	0
	3	0	0	1	0
	2	0	0	0	1
	1	0	0	0	0

female: BMI_g with y1_comb: adjusted model

PHREG 过程

事件和删失值个数汇总			
合计	事件	删失	删失百分比
4426	503	3923	88.64

收敛状态
满足收敛准则 (GCONV=1E-8)。

模型拟合统计量		
准则	无协变量	带协变量
-2 LOG L	8371.050	8310.741
AIC	8371.050	8348.741
SBC	8371.050	8428.932

检验全局原假设: BETA=0			
检验	卡方	自由度	Pr > 卡方
似然比	60.3095	19	<.0001
评分	63.6803	19	<.0001
Wald	62.6226	19	<.0001

3 型检验			
效应	自由度	Wald 卡方	Pr > 卡方
BMI_g	3	1.5077	0.6805
AGE	1	10.2764	0.0013
ETHNIC	1	1.3660	0.2425
H_DIAB01	1	3.1096	0.0778
H_AF01	1	8.9866	0.0027
H_HYPT01	1	0.2384	0.6254
H_LIPID01	1	0.7676	0.3810
AI	1	1.1266	0.2885
H_DRINK_H01	1	0.0303	0.8619
H_SMK_C01	1	4.9363	0.0263
IT	1	0.3965	0.5289
ET	1	0.8172	0.3660
IMG_C_TOAST	4	19.1178	0.0007
A_NIHSS	1	1.5017	0.2204

female: BMI_g with y1_comb: adjusted model

PHREG 过程

最大似然估计分析									
参数		自由度	参数估计	标准误差	卡方	Pr > 卡方	危险率	95% 危险率置信限	
BMI_g	4	1	0.08786	0.13407	0.4295	0.5122	1.092	0.840	1.420
BMI_g	3	1	0.05301	0.10728	0.2441	0.6212	1.054	0.854	1.301
BMI_g	1	1	0.27830	0.24154	1.3275	0.2492	1.321	0.823	2.121
AGE		1	0.01416	0.00442	10.2764	0.0013	1.014	1.006	1.023
ETHNIC	2	1	-0.37434	0.32029	1.3660	0.2425	0.688	0.367	1.288
H_DIAB01	1	1	0.17391	0.09862	3.1096	0.0778	1.190	0.981	1.444
H_AF01	1	1	0.53041	0.17693	8.9866	0.0027	1.700	1.202	2.404
H_HYPT01	1	1	0.04880	0.09995	0.2384	0.6254	1.050	0.863	1.277
H_LIPID01	1	1	-0.15722	0.17945	0.7676	0.3810	0.855	0.601	1.215
AI	1	1	0.31412	0.29595	1.1266	0.2885	1.369	0.766	2.445
H_DRINK_H01	1	1	0.12612	0.72474	0.0303	0.8619	1.134	0.274	4.695
H_SMK_C01	1	1	0.45143	0.20318	4.9363	0.0263	1.571	1.055	2.339
IT	1	1	0.09009	0.14306	0.3965	0.5289	1.094	0.827	1.448
ET	1	1	0.41853	0.46298	0.8172	0.3660	1.520	0.613	3.766
IMG_C_TOAST	5	1	-0.35269	0.10819	10.6270	0.0011	0.703	0.569	0.869
IMG_C_TOAST	4	1	-0.11467	0.34357	0.1114	0.7386	0.892	0.455	1.748
IMG_C_TOAST	3	1	-0.48553	0.13934	12.1423	0.0005	0.615	0.468	0.809
IMG_C_TOAST	2	1	-0.66433	0.22233	8.9285	0.0028	0.515	0.333	0.796
A_NIHSS		1	0.01224	0.00999	1.5017	0.2204	1.012	0.993	1.032

female: BMI_g with y1_comb: adjusted model

PHREG 过程

最大似然估计分析		
参数		标签
BMI_g	4	1=<18.5;2=18.5-<23;3=23-<27.5;4=≥27.5 4
BMI_g	3	1=<18.5;2=18.5-<23;3=23-<27.5;4=≥27.5 3
BMI_g	1	1=<18.5;2=18.5-<23;3=23-<27.5;4=≥27.5 1
AGE		A.Basic Information: Age (years old);
ETHNIC	2	B.Demography: Race: 1-Han; 99-others; 2
H_DIAB01	1	D.History: Diabetes; 0-No; 1-Yes; 1
H_AF01	1	D.History: Heart disease category: Atrial fibrillation(Including medical history and hospitalization diagnosis); 0-No; 1-Yes; 1
H_HYPT01	1	D.History: Hypertension; 0-No; 1-Yes; 1
H_LIPID01	1	D.History: Lipid metabolism disorders; 0-No; 1-Yes; 1
AI	1	history:Myocardial infarction; 0=NO; 1=YES; 1
H_DRINK_H01	1	D.History: Heavy Drinking(Alcohol consumption>=20g/day); 0-No,1-Yes; 1
H_SMK_C01	1	D.History: Current Smoking; 0-No,1-Yes; 1
IT	1	intravenous thrombolysis, 1=YES,0=NO 1
ET	1	动脉溶栓或机械取栓, 1=YES,0=NO 1
IMG_C_TOAST	5	K.Final diagnosis: cerebral infarction; Etiology according to TOAST system; 1-large artery atherosclerosis; 2-cardiogenic embolism; 3-small artery occlusion; 4-stroke of another determined cause; 5-stroke of an undetermined cause. 5
IMG_C_TOAST	4	K.Final diagnosis: cerebral infarction; Etiology according to TOAST system; 1-large artery atherosclerosis; 2-cardiogenic embolism; 3-small artery occlusion; 4-stroke of another determined cause; 5-stroke of an undetermined cause. 4
IMG_C_TOAST	3	K.Final diagnosis: cerebral infarction; Etiology according to TOAST system; 1-large artery atherosclerosis; 2-cardiogenic embolism; 3-small artery occlusion; 4-stroke of another determined cause; 5-stroke of an undetermined cause. 3
IMG_C_TOAST	2	K.Final diagnosis: cerebral infarction; Etiology according to TOAST system; 1-large artery atherosclerosis; 2-cardiogenic embolism; 3-small artery occlusion; 4-stroke of another determined cause; 5-stroke of an undetermined cause. 2
A_NIHSS		F.Admitting NIHSS: Total score;

BMI_g with y1_comb: interaction

PHREG 过程

模型信息		
数据集	WORK.DATA_OVERALL	
因变量	y1_comb_dd	N12.Follow-up events at 12 months: Days from onset to occurrence of combined vascular event;(day);
删失变量	y1_comb	N12.Follow-up events at 12 months:Occurrence of combined vascular event(including cardiovascular death,non-fatal stroke,non-fatal myocardial infarction):0-No;1-Yes;
删失值	0	
结值处理	BRESLOW	

读取的观测数	14146
使用的观测数	14146

分类水平信息					
分类	值	设计变量			
BMI_g	4	1	0	0	
	3	0	1	0	
	2	0	0	0	
	1	0	0	1	
ETHNIC	2	1			
	1	0			
H_DIAB01	1	1			
	0	0			
H_AF01	1	1			
	0	0			
H_HYPT01	1	1			
	0	0			
H_LIPID01	1	1			
	0	0			
AI	1	1			
	0	0			
H_DRINK_H01	1	1			
	0	0			
H_SMK_C01	1	1			
	0	0			
IT	1	1			
	0	0			
ET	1	1			
	0	0			
IMG_C_TOAST	5	1	0	0	0
	4	0	1	0	0
	3	0	0	1	0
	2	0	0	0	1
	1	0	0	0	0

BMI_g with y1_comb: interaction

PHREG 过程

事件和删失值个数汇总			
合计	事件	删失	删失百分比
14146	1505	12641	89.36

收敛状态
满足收敛准则 (GCONV=1E-8)。

模型拟合统计量		
准则	无协变量	带协变量
-2 LOG L	28553.433	28380.441
AIC	28553.433	28426.441
SBC	28553.433	28548.722

检验全局原假设: BETA=0			
检验	卡方	自由度	Pr > 卡方
似然比	172.9917	23	<.0001
评分	184.7459	23	<.0001
Wald	180.5634	23	<.0001

联合检验			
效应	自由度	Wald 卡方	Pr > 卡方
BMI_g	3	0.8445	0.8388
GENDER	1	0.0013	0.9708
GENDER*BMI_g	3	0.9483	0.8138
AGE	1	19.7272	<.0001
ETHNIC	1	0.4570	0.4990
H_DIAB01	1	14.1516	0.0002
H_AF01	1	13.2816	0.0003
H_HYPT01	1	2.6923	0.1008
H_LIPID01	1	0.6429	0.4227
AI	1	2.1480	0.1428
H_DRINK_H01	1	1.2781	0.2583
H_SMK_C01	1	0.2813	0.5958
IT	1	0.2124	0.6449
ET	1	5.9946	0.0143
IMG_C_TOAST	4	54.5175	<.0001
A_NIHSS	1	15.9017	<.0001

Note: Under full-rank parameterizations, Type 3 effect tests are replaced by joint tests. The joint test for an effect is a test that all of the parameters associated with that effect are zero. Such joint tests might not be equivalent to Type 3 effect tests under GLM parameterization.

BMI_g with y1_comb: interaction

PHREG 过程

最大似然估计分析

参数		自由度	参数估计	标准误差	卡方	Pr > 卡方	危险率	95% 危险率置信限	
BMI_g	4	1	0.11681	0.23583	0.2453	0.6204	.	.	.
BMI_g	3	1	-0.02099	0.18386	0.0130	0.9091	.	.	.
BMI_g	1	1	-0.32277	0.53238	0.3676	0.5443	.	.	.
GENDER		1	0.00394	0.10768	0.0013	0.9708	.	.	.
GENDER*BMI_g	4	1	-0.01751	0.16382	0.0114	0.9149	.	.	.
GENDER*BMI_g	3	1	0.03186	0.12984	0.0602	0.8062	.	.	.
GENDER*BMI_g	1	1	0.30720	0.33744	0.8288	0.3626	.	.	.
AGE		1	0.01111	0.00250	19.7272	<.0001	1.011	1.006	1.016
ETHNIC	2	1	-0.10860	0.16065	0.4570	0.4990	0.897	0.655	1.229
H_DIAB01	1	1	0.22185	0.05897	14.1516	0.0002	1.248	1.112	1.401
H_AF01	1	1	0.42156	0.11567	13.2816	0.0003	1.524	1.215	1.912
H_HYPT01	1	1	0.09166	0.05586	2.6923	0.1008	1.096	0.982	1.223
H_LIPID01	1	1	-0.07995	0.09972	0.6429	0.4227	0.923	0.759	1.122
AI	1	1	0.23157	0.15800	2.1480	0.1428	1.261	0.925	1.718
H_DRINK_H01	1	1	0.09316	0.08240	1.2781	0.2583	1.098	0.934	1.290
H_SMK_C01	1	1	-0.03567	0.06725	0.2813	0.5958	0.965	0.846	1.101
IT	1	1	0.03772	0.08183	0.2124	0.6449	1.038	0.885	1.219
ET	1	1	0.61175	0.24986	5.9946	0.0143	1.844	1.130	3.009
IMG_C_TOAST	5	1	-0.33023	0.06150	28.8340	<.0001	0.719	0.637	0.811
IMG_C_TOAST	4	1	-0.02407	0.22330	0.0116	0.9141	0.976	0.630	1.512
IMG_C_TOAST	3	1	-0.52953	0.07916	44.7522	<.0001	0.589	0.504	0.688
IMG_C_TOAST	2	1	-0.42385	0.13555	9.7780	0.0018	0.655	0.502	0.854
A_NIHSS		1	0.02276	0.00571	15.9017	<.0001	1.023	1.012	1.035

BMI_g with y1_comb: interaction

PHREG 过程

最大似然估计分析		
参数		标签
BMI_g	4	1=<18.5;2=18.5-<23;3=23-<27.5;4= ≥ 27.5 4
BMI_g	3	1=<18.5;2=18.5-<23;3=23-<27.5;4= ≥ 27.5 3
BMI_g	1	1=<18.5;2=18.5-<23;3=23-<27.5;4= ≥ 27.5 1
GENDER		A.Basic Information: Gender; 1-male; 2-female;
GENDER*BMI_g	4	1=<18.5;2=18.5-<23;3=23-<27.5;4= ≥ 27.5 4 * A.Basic Information: Gender; 1-male; 2-female;
GENDER*BMI_g	3	1=<18.5;2=18.5-<23;3=23-<27.5;4= ≥ 27.5 3 * A.Basic Information: Gender; 1-male; 2-female;
GENDER*BMI_g	1	1=<18.5;2=18.5-<23;3=23-<27.5;4= ≥ 27.5 1 * A.Basic Information: Gender; 1-male; 2-female;
AGE		A.Basic Information: Age (years old);
ETHNIC	2	B.Demography: Race: 1-Han; 99-others; 2
H_DIAB01	1	D.History: Diabetes; 0-No; 1-Yes; 1
H_AF01	1	D.History: Heart disease category: Atrial fibrillation(Including medical history and hospitalization diagnosis); 0-No; 1-Yes; 1
H_HYPT01	1	D.History: Hypertension; 0-No; 1-Yes; 1
H_LIPID01	1	D.History: Lipid metabolism disorders; 0-No; 1-Yes; 1
AI	1	history:Myocardial infarction; 0=NO; 1=YES; 1
H_DRINK_H01	1	D.History: Heavy Drinking(Alcohol consumption>=20g/day); 0-No,1-Yes; 1
H_SMK_C01	1	D.History: Current Smoking; 0-No,1-Yes; 1
IT	1	intravenous thrombolysis, 1=YES,0=NO 1
ET	1	动脉溶栓或机械取栓, 1=YES,0=NO 1
IMG_C_TOAST	5	K.Final diagnosis: cerebral infarction; Etiology according to TOAST system; 1-large artery atherosclerosis; 2-cardiogenic embolism; 3-small artery occlusion; 4-stroke of another determined cause; 5-stroke of an undetermined cause. 5
IMG_C_TOAST	4	K.Final diagnosis: cerebral infarction; Etiology according to TOAST system; 1-large artery atherosclerosis; 2-cardiogenic embolism; 3-small artery occlusion; 4-stroke of another determined cause; 5-stroke of an undetermined cause. 4
IMG_C_TOAST	3	K.Final diagnosis: cerebral infarction; Etiology according to TOAST system; 1-large artery atherosclerosis; 2-cardiogenic embolism; 3-small artery occlusion; 4-stroke of another determined cause; 5-stroke of an undetermined cause. 3
IMG_C_TOAST	2	K.Final diagnosis: cerebral infarction; Etiology according to TOAST system; 1-large artery atherosclerosis; 2-cardiogenic embolism; 3-small artery occlusion; 4-stroke of another determined cause; 5-stroke of an undetermined cause. 2
A_NIHSS		F.Admitting NIHSS: Total score;

female: BMI_g with y1_stroke: Descriptive results

FREQ 过程

频数<br>行百分比

BMI_g-y1_stroke表			
	y1_stroke(N12.Follow-up events at 12 months: Recurrence of stroke: 0-No; 1-Yes;)		
BMI_g(1=<18.5;2=18.5-<23;3=23-<27.5;4=≥27.5)	0	1	合计
1	113<br>85.61	19<br>14.39	132
2	1125<br>89.57	131<br>10.43	1256
3	1955<br>89.35	233<br>10.65	2188
4	755<br>88.82	95<br>11.18	850
合计	3948	478	4426

表“y1_stroke-BMI_g”的统计量

统计量	自由度	值	概率
卡方	3	2.1252	0.5468
似然比卡方检验	3	1.9779	0.5770
Mantel-Haenszel 卡方	1	0.0110	0.9163
Phi 系数		0.0219	
列联系数		0.0219	
Cramer V		0.0219	

样本大小 = 4426

female: BMI_g with y1_stroke: adjusted model

PHREG 过程

模型信息		
数据集	WORK.FEMALE	
因变量	y1_stroke_dd	N12.Follow-up events at 12 months: Days from onset to recurrence;(day);
删失变量	y1_stroke	N12.Follow-up events at 12 months: Recurrence of stroke: 0-No; 1-Yes;
删失值	0	
结值处理	BRESLOW	

读取的观测数	4426
使用的观测数	4426

分类水平信息						
分类	值	设计变量				
BMI_g	4	1	0	0	
	3	0	1	0	
	2	0	0	0	
	1	0	0	1	
ETHNIC	2	1			
	1	0			
H_DIAB01	1	1			
	0	0			
H_AF01	1	1			
	0	0			
H_HYPT01	1	1			
	0	0			
H_LIPID01	1	1			
	0	0			
AI	1	1			
	0	0			
H_DRINK_H01	1	1			
	0	0			
H_SMK_C01	1	1			
	0	0			
IT	1	1			
	0	0			
ET	1	1			
	0	0			
IMG_C_TOAST	5	1	0	0	0
	4	0	1	0	0
	3	0	0	1	0
	2	0	0	0	1
	1	0	0	0	0

female: BMI_g with y1_stroke: adjusted model

PHREG 过程

事件和删失值个数汇总			
合计	事件	删失	删失百分比
4426	478	3948	89.20

收敛状态
满足收敛准则 (GCONV=1E-8)。

模型拟合统计量		
准则	无协变量	带协变量
-2 LOG L	7957.805	7902.779
AIC	7957.805	7940.779
SBC	7957.805	8020.002

检验全局原假设: BETA=0			
检验	卡方	自由度	Pr > 卡方
似然比	55.0256	19	<.0001
评分	57.8594	19	<.0001
Wald	57.0794	19	<.0001

3 型检验			
效应	自由度	Wald 卡方	Pr > 卡方
BMI_g	3	1.7950	0.6160
AGE	1	8.6091	0.0033
ETHNIC	1	1.6266	0.2022
H_DIAB01	1	3.1789	0.0746
H_AF01	1	7.2322	0.0072
H_HYPT01	1	0.1495	0.6991
H_LIPID01	1	0.3158	0.5741
AI	1	0.8283	0.3628
H_DRINK_H01	1	0.0508	0.8217
H_SMK_C01	1	4.7864	0.0287
IT	1	1.3769	0.2406
ET	1	1.1102	0.2920
IMG_C_TOAST	4	19.2696	0.0007
A_NIHSS	1	0.9901	0.3197

female: BMI_g with y1_stroke: adjusted model

PHREG 过程

最大似然估计分析									
参数		自由度	参数估计	标准误差	卡方	Pr > 卡方	危险率	95% 危险率置信限	
BMI_g	4	1	0.11793	0.13717	0.7392	0.3899	1.125	0.860	1.472
BMI_g	3	1	0.06759	0.11057	0.3737	0.5410	1.070	0.861	1.329
BMI_g	1	1	0.29441	0.24806	1.4086	0.2353	1.342	0.825	2.183
AGE		1	0.01325	0.00452	8.6091	0.0033	1.013	1.004	1.022
ETHNIC	2	1	-0.43029	0.33738	1.6266	0.2022	0.650	0.336	1.260
H_DIAB01	1	1	0.18011	0.10102	3.1789	0.0746	1.197	0.982	1.460
H_AF01	1	1	0.49544	0.18423	7.2322	0.0072	1.641	1.144	2.355
H_HYPT01	1	1	0.03961	0.10245	0.1495	0.6991	1.040	0.851	1.272
H_LIPID01	1	1	-0.10108	0.17985	0.3158	0.5741	0.904	0.635	1.286
AI	1	1	0.28109	0.30886	0.8283	0.3628	1.325	0.723	2.427
H_DRINK_H01	1	1	0.16352	0.72567	0.0508	0.8217	1.178	0.284	4.883
H_SMK_C01	1	1	0.45366	0.20736	4.7864	0.0287	1.574	1.048	2.363
IT	1	1	0.16842	0.14354	1.3769	0.2406	1.183	0.893	1.568
ET	1	1	0.48882	0.46393	1.1102	0.2920	1.630	0.657	4.047
IMG_C_TOAST	5	1	-0.32978	0.11047	8.9121	0.0028	0.719	0.579	0.893
IMG_C_TOAST	4	1	-0.06648	0.34413	0.0373	0.8468	0.936	0.477	1.837
IMG_C_TOAST	3	1	-0.48805	0.14302	11.6457	0.0006	0.614	0.464	0.812
IMG_C_TOAST	2	1	-0.75764	0.23599	10.3071	0.0013	0.469	0.295	0.744
A_NIHSS		1	0.01032	0.01037	0.9901	0.3197	1.010	0.990	1.031

female: BMI_g with y1_stroke: adjusted model

PHREG 过程

最大似然估计分析		
参数		标签
BMI_g	4	1=<18.5;2=18.5-<23;3=23-<27.5;4= ≥ 27.5 4
BMI_g	3	1=<18.5;2=18.5-<23;3=23-<27.5;4= ≥ 27.5 3
BMI_g	1	1=<18.5;2=18.5-<23;3=23-<27.5;4= ≥ 27.5 1
AGE		A.Basic Information: Age (years old);
ETHNIC	2	B.Demography: Race: 1-Han; 99-others; 2
H_DIAB01	1	D.History: Diabetes; 0-No; 1-Yes; 1
H_AF01	1	D.History: Heart disease category: Atrial fibrillation(Including medical history and hospitalization diagnosis); 0-No; 1-Yes; 1
H_HYPT01	1	D.History: Hypertension; 0-No; 1-Yes; 1
H_LIPID01	1	D.History: Lipid metabolism disorders; 0-No; 1-Yes; 1
AI	1	history:Myocardial infarction; 0=NO; 1=YES; 1
H_DRINK_H01	1	D.History: Heavy Drinking(Alcohol consumption>=20g/day); 0-No,1-Yes; 1
H_SMK_C01	1	D.History: Current Smoking; 0-No,1-Yes; 1
IT	1	intravenous thrombolysis, 1=YES,0=NO 1
ET	1	动脉溶栓或机械取栓, 1=YES,0=NO 1
IMG_C_TOAST	5	K.Final diagnosis: cerebral infarction; Etiology according to TOAST system; 1-large artery atherosclerosis; 2-cardiogenic embolism; 3-small artery occlusion; 4-stroke of another determined cause; 5-stroke of an undetermined cause. 5
IMG_C_TOAST	4	K.Final diagnosis: cerebral infarction; Etiology according to TOAST system; 1-large artery atherosclerosis; 2-cardiogenic embolism; 3-small artery occlusion; 4-stroke of another determined cause; 5-stroke of an undetermined cause. 4
IMG_C_TOAST	3	K.Final diagnosis: cerebral infarction; Etiology according to TOAST system; 1-large artery atherosclerosis; 2-cardiogenic embolism; 3-small artery occlusion; 4-stroke of another determined cause; 5-stroke of an undetermined cause. 3
IMG_C_TOAST	2	K.Final diagnosis: cerebral infarction; Etiology according to TOAST system; 1-large artery atherosclerosis; 2-cardiogenic embolism; 3-small artery occlusion; 4-stroke of another determined cause; 5-stroke of an undetermined cause. 2
A_NIHSS		F.Admitting NIHSS: Total score;

BMI_g with y1_stroke: interaction

PHREG 过程

模型信息		
数据集	WORK.DATA_OVERALL	
因变量	y1_stroke_dd	N12.Follow-up events at 12 months: Days from onset to recurrence;(day);
删失变量	y1_stroke	N12.Follow-up events at 12 months: Recurrence of stroke: 0-No; 1-Yes;
删失值	0	
结值处理	BRESLOW	

读取的观测数<br>使用的观测数	14146<br>14146

分类水平信息						
分类	值	设计变量				
BMI_g	4	1	0	0		
	3	0	1	0		
	2	0	0	0		
	1	0	0	1		
ETHNIC	2	1				
	1	0				
H_DIAB01	1	1				
	0	0				
H_AF01	1	1				
	0	0				
H_HYPT01	1	1				
	0	0				
H_LIPID01	1	1				
	0	0				
AI	1	1				
	0	0				
H_DRINK_H01	1	1				
	0	0				
H_SMK_C01	1	1				
	0	0				
IT	1	1				
	0	0				
ET	1	1				
	0	0				
IMG_C_TOAST	5	1	0	0	0	
	4	0	1	0	0	
	3	0	0	1	0	
	2	0	0	0	1	
	1	0	0	0	0	

BMI_g with y1_stroke: interaction

PHREG 过程

事件和删失值个数汇总			
合计	事件	删失	删失百分比
14146	1424	12722	89.93

收敛状态
满足收敛准则 (GCONV=1E-8)。

模型拟合统计量		
准则	无协变量	带协变量
-2 LOG L	27024.892	26876.607
AIC	27024.892	26922.607
SBC	27024.892	27043.616

检验全局原假设: BETA=0			
检验	卡方	自由度	Pr > 卡方
似然比	148.2847	23	<.0001
评分	157.3531	23	<.0001
Wald	154.0667	23	<.0001

联合检验			
效应	自由度	Wald 卡方	Pr > 卡方
BMI_g	3	1.1676	0.7608
GENDER	1	0.0120	0.9129
GENDER*BMI_g	3	1.2310	0.7456
AGE	1	15.1460	<.0001
ETHNIC	1	0.6311	0.4270
H_DIAB01	1	11.9198	0.0006
H_AF01	1	8.3774	0.0038
H_HYPT01	1	1.3225	0.2501
H_LIPID01	1	0.5953	0.4404
AI	1	1.0313	0.3099
H_DRINK_H01	1	2.9756	0.0845
H_SMK_C01	1	0.7192	0.3964
IT	1	1.0128	0.3142
ET	1	5.8682	0.0154
IMG_C_TOAST	4	52.9659	<.0001
A_NIHSS	1	12.5836	0.0004

Note: Under full-rank parameterizations, Type 3 effect tests are replaced by joint tests. The joint test for an effect is a test that all of the parameters associated with that effect are zero. Such joint tests might not be equivalent to Type 3 effect tests under GLM parameterization.

BMI_g with y1_stroke: interaction

PHREG 过程

最大似然估计分析

参数		自由度	参数估计	标准误差	卡方	Pr > 卡方	危险率	95% 危险率置信限	
BMI_g	4	1	0.12574	0.24156	0.2710	0.6027	.	.	.
BMI_g	3	1	-0.02883	0.18953	0.0231	0.8791	.	.	.
BMI_g	1	1	-0.43117	0.55884	0.5953	0.4404	.	.	.
GENDER		1	0.01217	0.11126	0.0120	0.9129	.	.	.
GENDER*BMI_g	4	1	-0.00524	0.16771	0.0010	0.9751	.	.	.
GENDER*BMI_g	3	1	0.04337	0.13384	0.1050	0.7459	.	.	.
GENDER*BMI_g	1	1	0.37280	0.35134	1.1259	0.2887	.	.	.
AGE		1	0.00998	0.00256	15.1460	<.0001	1.010	1.005	1.015
ETHNIC	2	1	-0.13264	0.16697	0.6311	0.4270	0.876	0.631	1.215
H_DIAB01	1	1	0.20985	0.06078	11.9198	0.0006	1.233	1.095	1.390
H_AF01	1	1	0.35321	0.12203	8.3774	0.0038	1.424	1.121	1.808
H_HYPT01	1	1	0.06581	0.05722	1.3225	0.2501	1.068	0.955	1.195
H_LIPID01	1	1	-0.07910	0.10252	0.5953	0.4404	0.924	0.756	1.130
AI	1	1	0.17069	0.16808	1.0313	0.3099	1.186	0.853	1.649
H_DRINK_H01	1	1	0.14444	0.08374	2.9756	0.0845	1.155	0.981	1.361
H_SMK_C01	1	1	-0.05871	0.06923	0.7192	0.3964	0.943	0.823	1.080
IT	1	1	0.08366	0.08313	1.0128	0.3142	1.087	0.924	1.280
ET	1	1	0.62396	0.25758	5.8682	0.0154	1.866	1.127	3.092
IMG_C_TOAST	5	1	-0.33808	0.06300	28.8004	<.0001	0.713	0.630	0.807
IMG_C_TOAST	4	1	-0.03466	0.22881	0.0230	0.8796	0.966	0.617	1.513
IMG_C_TOAST	3	1	-0.52896	0.08089	42.7567	<.0001	0.589	0.503	0.690
IMG_C_TOAST	2	1	-0.44622	0.14210	9.8606	0.0017	0.640	0.484	0.846
A_NIHSS		1	0.02105	0.00593	12.5836	0.0004	1.021	1.009	1.033

BMI_g with y1_stroke: interaction

PHREG 过程

最大似然估计分析		
参数		标签
BMI_g	4	1=<18.5;2=18.5-<23;3=23-<27.5;4= ≥ 27.5 4
BMI_g	3	1=<18.5;2=18.5-<23;3=23-<27.5;4= ≥ 27.5 3
BMI_g	1	1=<18.5;2=18.5-<23;3=23-<27.5;4= ≥ 27.5 1
GENDER		A.Basic Information: Gender; 1-male; 2-female;
GENDER*BMI_g	4	1=<18.5;2=18.5-<23;3=23-<27.5;4= ≥ 27.5 4 * A.Basic Information: Gender; 1-male; 2-female;
GENDER*BMI_g	3	1=<18.5;2=18.5-<23;3=23-<27.5;4= ≥ 27.5 3 * A.Basic Information: Gender; 1-male; 2-female;
GENDER*BMI_g	1	1=<18.5;2=18.5-<23;3=23-<27.5;4= ≥ 27.5 1 * A.Basic Information: Gender; 1-male; 2-female;
AGE		A.Basic Information: Age (years old);
ETHNIC	2	B.Demography: Race: 1-Han; 99-others; 2
H_DIAB01	1	D.History: Diabetes; 0-No; 1-Yes; 1
H_AF01	1	D.History: Heart disease category: Atrial fibrillation(Including medical history and hospitalization diagnosis); 0-No; 1-Yes; 1
H_HYPT01	1	D.History: Hypertension; 0-No; 1-Yes; 1
H_LIPID01	1	D.History: Lipid metabolism disorders; 0-No; 1-Yes; 1
AI	1	history:Myocardial infarction; 0=NO; 1=YES; 1
H_DRINK_H01	1	D.History: Heavy Drinking(Alcohol consumption>=20g/day); 0-No,1-Yes; 1
H_SMK_C01	1	D.History: Current Smoking; 0-No,1-Yes; 1
IT	1	intravenous thrombolysis, 1=YES,0=NO 1
ET	1	动脉溶栓或机械取栓, 1=YES,0=NO 1
IMG_C_TOAST	5	K.Final diagnosis: cerebral infarction; Etiology according to TOAST system; 1-large artery atherosclerosis; 2-cardiogenic embolism; 3-small artery occlusion; 4-stroke of another determined cause; 5-stroke of an undetermined cause. 5
IMG_C_TOAST	4	K.Final diagnosis: cerebral infarction; Etiology according to TOAST system; 1-large artery atherosclerosis; 2-cardiogenic embolism; 3-small artery occlusion; 4-stroke of another determined cause; 5-stroke of an undetermined cause. 4
IMG_C_TOAST	3	K.Final diagnosis: cerebral infarction; Etiology according to TOAST system; 1-large artery atherosclerosis; 2-cardiogenic embolism; 3-small artery occlusion; 4-stroke of another determined cause; 5-stroke of an undetermined cause. 3
IMG_C_TOAST	2	K.Final diagnosis: cerebral infarction; Etiology according to TOAST system; 1-large artery atherosclerosis; 2-cardiogenic embolism; 3-small artery occlusion; 4-stroke of another determined cause; 5-stroke of an undetermined cause. 2
A_NIHSS		F.Admitting NIHSS: Total score;
