# Supplementary material for: Association of body mass index and waist-to-height ratio with outcomes in ischemic stroke: results from the Third China National Stroke Registry
Source: BMC Neurol. 2023 Apr 14;23:152. doi: 10.1186/s12883-023-03165-y (PMC10103413; doi:10.1186/s12883-023-03165-y)
Supplement: Supplementary file 2 — Additional file 2. [file 12883_2023_3165_MOESM2_ESM.zip › raw data/table3 and fig3fig4-whtr.pdf]

data1: whtr with y1_death: crude model

PHREG 过程

模型信息		
数据集	WORK.DATA1	
因变量	y1_death_dd	N12.Follow-up events at 12 months: Days from onset to death;(day);
删失变量	y1_death	N12.Follow-up events at 12 months: Whether the patient died: 0-survival;1-death;
删失值	0	
结值处理	BRESLOW	

读取的观测数	4805
使用的观测数	4805

事件和删失值个数汇总			
合计	事件	删失	删失百分比
4805	190	4615	96.05

收敛状态
满足收敛准则 (GCONV=1E-8)。

模型拟合统计量		
准则	无协变量	带协变量
-2 LOG L	3206.595	3195.724
AIC	3206.595	3197.724
SBC	3206.595	3200.971

检验全局原假设: BETA=0			
检验	卡方	自由度	Pr > 卡方
似然比	10.8709	1	0.0010
评分	11.2073	1	0.0008
Wald	11.4275	1	0.0007

最大似然估计分析								
参数	自由度	参数估计	标准误差	卡方	Pr > 卡方	危险率	95% 危险率置信限	
whtr	1	-2.69663	0.79771	11.4275	0.0007	0.067	0.014	0.322

data1: whtr with y1_death: adjusted model

PHREG 过程

模型信息		
数据集	WORK.DATA1	
因变量	y1_death_dd	N12.Follow-up events at 12 months: Days from onset to death;(day);
删失变量	y1_death	N12.Follow-up events at 12 months: Whether the patient died: 0-survival;1-death;
删失值	0	
结值处理	BRESLOW	

读取的观测数<br>使用的观测数	4805<br>4805

分类水平信息		
分类	值	设计变量
GENDER	2	1
	1	0
ETHNIC	2	1
	1	0
H_DIAB01	1	1
	0	0
H_AF01	1	1
	0	0
H_HYPT01	1	1
	0	0
H_LIPID01	1	1
	0	0
AI	1	1
	0	0
H_DRINK_H01	1	1
	0	0
H_SMK_C01	1	1
	0	0
IT	1	1
	0	0
ET	1	1
	0	0

事件和删失值个数汇总			
合计	事件	删失	删失<br>百分比
4805	190	4615	96.05

收敛状态
满足收敛准则 (GCONV=1E-8)。

data1: whtr with y1_death: adjusted model

PHREG 过程

模型拟合统计量		
准则	无<br>协变量	带<br>协变量
-2 LOG L	3206.595	2927.540
AIC	3206.595	2957.540
SBC	3206.595	3006.246

检验全局原假设: BETA=0			
检验	卡方	自由度	Pr > 卡方
似然比	279.0542	15	<.0001
评分	390.8651	15	<.0001
Wald	342.7411	15	<.0001

3 型检验			
效应	自由度	Wald 卡方	Pr > 卡方
whtr	1	3.2729	0.0704
AGE	1	82.8720	<.0001
GENDER	1	0.8559	0.3549
ETHNIC	1	7.6534	0.0057
H_DIAB01	1	2.6994	0.1004
H_AF01	1	16.7899	<.0001
H_HYPT01	1	0.2143	0.6434
H_LIPID01	1	1.8308	0.1760
AI	1	0.0276	0.8681
H_DRINK_H01	1	2.4910	0.1145
H_SMK_C01	1	0.1583	0.6908
IT	1	5.2634	0.0218
ET	1	0.5406	0.4622
A_NIHSS	1	80.7836	<.0001
IMG_C_TOAST	1	0.1054	0.7454

data1: whtr with y1_death: adjusted model

PHREG 过程

最大似然估计分析									
参数		自由度	参数估计	标准误差	卡方	Pr > 卡方	危险率	95% 危险率置信限	
whtr		1	-1.50624	0.83258	3.2729	0.0704	0.222	0.043	1.134
AGE		1	0.07132	0.00783	82.8720	<.0001	1.074	1.058	1.091
GENDER	2	1	-0.15073	0.16292	0.8559	0.3549	0.860	0.625	1.184
ETHNIC	2	1	0.78228	0.28277	7.6534	0.0057	2.186	1.256	3.806
H_DIAB01	1	1	0.28625	0.17423	2.6994	0.1004	1.331	0.946	1.873
H_AF01	1	1	0.76175	0.18590	16.7899	<.0001	2.142	1.488	3.084
H_HYPT01	1	1	0.07332	0.15836	0.2143	0.6434	1.076	0.789	1.468
H_LIPID01	1	1	-0.56590	0.41824	1.8308	0.1760	0.568	0.250	1.289
AI	1	1	0.07718	0.46478	0.0276	0.8681	1.080	0.434	2.686
H_DRINK_H01	1	1	-0.56967	0.36094	2.4910	0.1145	0.566	0.279	1.148
H_SMK_C01	1	1	-0.08644	0.21728	0.1583	0.6908	0.917	0.599	1.404
IT	1	1	-0.59740	0.26039	5.2634	0.0218	0.550	0.330	0.917
ET	1	1	0.44214	0.60137	0.5406	0.4622	1.556	0.479	5.057
A_NIHSS		1	0.08885	0.00989	80.7836	<.0001	1.093	1.072	1.114
IMG_C_TOAST		1	-0.01417	0.04363	0.1054	0.7454	0.986	0.905	1.074

最大似然估计分析		
参数		标签
whtr		
AGE		A.Basic Information: Age (years old);
GENDER	2	A.Basic Information: Gender; 1-male; 2-female; 2
ETHNIC	2	B.Demography: Race: 1-Han; 99-others; 2
H_DIAB01	1	D.History: Diabetes; 0-No; 1-Yes; 1
H_AF01	1	D.History: Heart disease category: Atrial fibrillation(Including medical history and hospitalization diagnosis); 0-No; 1-Yes; 1
H_HYPT01	1	D.History: Hypertension; 0-No; 1-Yes; 1
H_LIPID01	1	D.History: Lipid metabolism disorders; 0-No; 1-Yes; 1
AI	1	history:Myocardial infarction; 0=NO; 1=YES; 1
H_DRINK_H01	1	D.History: Heavy Drinking(Alcohol consumption>=20g/day); 0-No,1-Yes; 1
H_SMK_C01	1	D.History: Current Smoking; 0-No,1-Yes; 1
IT	1	intravenous thrombolysis, 1=YES,0=NO 1
ET	1	动脉溶栓或机械取栓, 1=YES,0=NO 1
A_NIHSS		F.Admitting NIHSS: Total score;
IMG_C_TOAST		K.Final diagnosis: cerebral infarction; Etiology according to TOAST system; 1-large artery atherosclerosis; 2-cardiogenic embolism; 3-small artery occlusion; 4-stroke of another determined cause; 5-stroke of an undetermined cause.

data1: whtr with y1_death: interaction model

PHREG 过程

模型信息		
数据集	WORK.DATA1	
因变量	y1_death_dd	N12.Follow-up events at 12 months: Days from onset to death;(day);
删失变量	y1_death	N12.Follow-up events at 12 months: Whether the patient died: 0-survival;1-death;
删失值	0	
结值处理	BRESLOW	

读取的观测数<br>使用的观测数	4805<br>4805

分类水平信息					
分类	值	设计变量			
GENDER	2	1			
	1	0			
IMG_C_TOAST	5	1	0	0	0
	4	0	1	0	0
	3	0	0	1	0
	2	0	0	0	1
	1	0	0	0	0
ETHNIC	2	1			
	1	0			
H_DIAB01	1	1			
	0	0			
H_AF01	1	1			
	0	0			
H_HYPT01	1	1			
	0	0			
H_LIPID01	1	1			
	0	0			
AI	1	1			
	0	0			
H_DRINK_H01	1	1			
	0	0			
H_SMK_C01	1	1			
	0	0			
IT	1	1			
	0	0			
ET	1	1			
	0	0			

data1: whtr with y1_death: interaction model

PHREG 过程

事件和删失值个数汇总			
合计	事件	删失	删失百分比
4805	190	4615	96.05

收敛状态
满足收敛准则 (GCONV=1E-8)。

模型拟合统计量		
准则	无协变量	带协变量
-2 LOG L	3206.595	2912.232
AIC	3206.595	2956.232
SBC	3206.595	3027.667

检验全局原假设: BETA=0			
检验	卡方	自由度	Pr > 卡方
似然比	294.3625	22	<.0001
评分	415.3084	22	<.0001
Wald	347.2772	22	<.0001

联合检验			
效应	自由度	Wald 卡方	Pr > 卡方
whtr	1	0.4173	0.5183
IMG_C_TOAST	4	1.4847	0.8294
whtr*IMG_C_TOAST	4	2.6489	0.6182
AGE	1	80.3659	<.0001
GENDER	1	1.3534	0.2447
ETHNIC	1	7.5633	0.0060
H_DIAB01	1	3.3546	0.0670
H_AF01	1	15.8794	<.0001
H_HYPT01	1	0.1121	0.7377
H_LIPID01	1	1.7557	0.1852
AI	1	0.1108	0.7392
H_DRINK_H01	1	2.3158	0.1281
H_SMK_C01	1	0.2197	0.6393
IT	1	5.6444	0.0175
ET	1	0.6325	0.4264
A_NIHSS	1	69.8940	<.0001

Note: Under full-rank parameterizations, Type 3 effect tests are replaced by joint tests. The joint test for an effect is a test that all of the parameters associated with that effect are zero. Such joint tests might not be equivalent to Type 3 effect tests under GLM parameterization.

data1: whtr with y1_death: interaction model

PHREG 过程

最大似然估计分析

参数		自由度	参数估计	标准误差	卡方	Pr > 卡方	危险率	95% 危险率置信限	
whtr		1	-1.04603	1.61919	0.4173	0.5183	.	.	.
IMG_C_TOAST	5	1	-0.46628	1.04448	0.1993	0.6553	.	.	.
IMG_C_TOAST	4	1	0.08426	2.86360	0.0009	0.9765	.	.	.
IMG_C_TOAST	3	1	0.66803	1.52777	0.1912	0.6619	.	.	.
IMG_C_TOAST	2	1	0.73103	1.23359	0.3512	0.5534	.	.	.
whtr*IMG_C_TOAST	5	1	0.52176	2.03360	0.0658	0.7975	.	.	.
whtr*IMG_C_TOAST	4	1	1.58607	5.67205	0.0782	0.7798	.	.	.
whtr*IMG_C_TOAST	3	1	-2.72646	3.04570	0.8014	0.3707	.	.	.
whtr*IMG_C_TOAST	2	1	-2.36368	2.46062	0.9228	0.3368	.	.	.
AGE		1	0.07006	0.00781	80.3659	<.0001	1.073	1.056	1.089
GENDER	2	1	-0.19068	0.16391	1.3534	0.2447	0.826	0.599	1.139
ETHNIC	2	1	0.77873	0.28316	7.5633	0.0060	2.179	1.251	3.795
H_DIAB01	1	1	0.32033	0.17489	3.3546	0.0670	1.378	0.978	1.941
H_AF01	1	1	0.93698	0.23513	15.8794	<.0001	2.552	1.610	4.046
H_HYPT01	1	1	0.05327	0.15910	0.1121	0.7377	1.055	0.772	1.441
H_LIPID01	1	1	-0.55524	0.41904	1.7557	0.1852	0.574	0.252	1.305
AI	1	1	0.15517	0.46619	0.1108	0.7392	1.168	0.468	2.912
H_DRINK_H01	1	1	-0.55059	0.36181	2.3158	0.1281	0.577	0.284	1.172
H_SMK_C01	1	1	-0.10211	0.21785	0.2197	0.6393	0.903	0.589	1.384
IT	1	1	-0.64266	0.27051	5.6444	0.0175	0.526	0.309	0.894
ET	1	1	0.47900	0.60230	0.6325	0.4264	1.614	0.496	5.257
A_NIHSS		1	0.08479	0.01014	69.8940	<.0001	1.088	1.067	1.110

data1: whtr with y1_death: interaction model

PHREG 过程

最大似然估计分析		
参数		标签
whtr		
IMG_C_TOAST	5	K.Final diagnosis: cerebral infarction; Etiology according to TOAST system; 1-large artery atherosclerosis; 2-cardiogenic embolism; 3-small artery occlusion; 4-stroke of another determined cause; 5-stroke of an undetermined cause. 5
IMG_C_TOAST	4	K.Final diagnosis: cerebral infarction; Etiology according to TOAST system; 1-large artery atherosclerosis; 2-cardiogenic embolism; 3-small artery occlusion; 4-stroke of another determined cause; 5-stroke of an undetermined cause. 4
IMG_C_TOAST	3	K.Final diagnosis: cerebral infarction; Etiology according to TOAST system; 1-large artery atherosclerosis; 2-cardiogenic embolism; 3-small artery occlusion; 4-stroke of another determined cause; 5-stroke of an undetermined cause. 3
IMG_C_TOAST	2	K.Final diagnosis: cerebral infarction; Etiology according to TOAST system; 1-large artery atherosclerosis; 2-cardiogenic embolism; 3-small artery occlusion; 4-stroke of another determined cause; 5-stroke of an undetermined cause. 2
whtr*IMG_C_TOAST	5	K.Final diagnosis: cerebral infarction; Etiology according to TOAST system; 1-large artery atherosclerosis; 2-cardiogenic embolism; 3-small artery occlusion; 4-stroke of another determined cause; 5-stroke of an undetermined cause. 5 * whtr
whtr*IMG_C_TOAST	4	K.Final diagnosis: cerebral infarction; Etiology according to TOAST system; 1-large artery atherosclerosis; 2-cardiogenic embolism; 3-small artery occlusion; 4-stroke of another determined cause; 5-stroke of an undetermined cause. 4 * whtr
whtr*IMG_C_TOAST	3	K.Final diagnosis: cerebral infarction; Etiology according to TOAST system; 1-large artery atherosclerosis; 2-cardiogenic embolism; 3-small artery occlusion; 4-stroke of another determined cause; 5-stroke of an undetermined cause. 3 * whtr
whtr*IMG_C_TOAST	2	K.Final diagnosis: cerebral infarction; Etiology according to TOAST system; 1-large artery atherosclerosis; 2-cardiogenic embolism; 3-small artery occlusion; 4-stroke of another determined cause; 5-stroke of an undetermined cause. 2 * whtr
AGE		A.Basic Information: Age (years old);
GENDER	2	A.Basic Information: Gender; 1-male; 2-female; 2
ETHNIC	2	B.Demography: Race: 1-Han; 99-others; 2
H_DIAB01	1	D.History: Diabetes; 0-No; 1-Yes; 1
H_AF01	1	D.History: Heart disease category: Atrial fibrillation(Including medical history and hospitalization diagnosis); 0-No; 1-Yes; 1
H_HYPT01	1	D.History: Hypertension; 0-No; 1-Yes; 1
H_LIPID01	1	D.History: Lipid metabolism disorders; 0-No; 1-Yes; 1
AI	1	history:Myocardial infarction; 0=NO; 1=YES; 1
H_DRINK_H01	1	D.History: Heavy Drinking(Alcohol consumption>=20g/day); 0-No,1-Yes; 1
H_SMK_C01	1	D.History: Current Smoking; 0-No,1-Yes; 1
IT	1	intravenous thrombolysis, 1=YES,0=NO 1
ET	1	动脉溶栓或机械取栓, 1=YES,0=NO 1
A_NIHSS		F.Admitting NIHSS: Total score;

data1: whtr_g with y1_death: Descriptive results

FREQ 过程

频数<br>行百分比

whtr_g-y1_death表			
	y1_death(N12.Follow-up events at 12 months: Whether the patient died: 0-survival;1-death;)		
whtr_g(1=Q1(<0.47);2=Q2(0.47-<0.52);3=Q3(0.52-<0.57);4=Q4(>=0.57))	0	1	合计
1	1102<br>94.27	67<br>5.73	1169
2	1253<br>95.21	63<br>4.79	1316
3	1165<br>97.90	25<br>2.10	1190
4	1095<br>96.90	35<br>3.10	1130
合计	4615	190	4805

表“y1_death-whtr_g”的统计量

统计量	自由度	值	概率
卡方	3	25.0738	<.0001
似然比卡方检验	3	26.0976	<.0001
Mantel-Haenszel 卡方	1	17.5691	<.0001
Phi 系数		0.0722	
列联系数		0.0720	
Cramer V		0.0722	

样本大小 = 4805

data1: whtr_g with y1_death: crude model

PHREG 过程

模型信息		
数据集	WORK.DATA1	
因变量	y1_death_dd	N12.Follow-up events at 12 months: Days from onset to death;(day);
删失变量	y1_death	N12.Follow-up events at 12 months: Whether the patient died: 0-survival;1-death;
删失值	0	
结值处理	BRESLOW	

读取的观测数	4805
使用的观测数	4805

分类水平信息				
分类	值	设计变量		
whtr_g	4	1	0	0
	3	0	1	0
	2	0	0	0
	1	0	0	1

事件和删失值个数汇总			
合计	事件	删失	删失百分比
4805	190	4615	96.05

收敛状态
满足收敛准则 (GCONV=1E-8)。

模型拟合统计量		
准则	无协变量	带协变量
-2 LOG L	3206.595	3180.022
AIC	3206.595	3186.022
SBC	3206.595	3195.763

检验全局原假设: BETA=0			
检验	卡方	自由度	Pr > 卡方
似然比	26.5732	3	<.0001
评分	25.5262	3	<.0001
Wald	24.0816	3	<.0001

3 型检验			
效应	自由度	Wald 卡方	Pr > 卡方
whtr_g	3	24.0816	<.0001

data1: whtr_g with y1_death: crude model

PHREG 过程

最大似然估计分析									
参数		自由度	参数估计	标准误差	卡方	Pr > 卡方	危险率	95% 危险率置信限	
whtr_g	4	1	-0.44278	0.21082	4.4111	0.0357	0.642	0.425	0.971
whtr_g	3	1	-0.84354	0.23638	12.7353	0.0004	0.430	0.271	0.684
whtr_g	1	1	0.19019	0.17550	1.1744	0.2785	1.209	0.857	1.706

最大似然估计分析		
参数		标签
whtr_g	4	1=Q1(<0.47);2=Q2(0.47-<0.52);3=Q3(0.52-<0.57);4=Q4(>=0.57) 4
whtr_g	3	1=Q1(<0.47);2=Q2(0.47-<0.52);3=Q3(0.52-<0.57);4=Q4(>=0.57) 3
whtr_g	1	1=Q1(<0.47);2=Q2(0.47-<0.52);3=Q3(0.52-<0.57);4=Q4(>=0.57) 1

data1: whtr_g with y1_death: adjusted model

PHREG 过程

模型信息		
数据集	WORK.DATA1	
因变量	y1_death_dd	N12.Follow-up events at 12 months: Days from onset to death;(day);
删失变量	y1_death	N12.Follow-up events at 12 months: Whether the patient died: 0-survival;1-death;
删失值	0	
结值处理	BRESLOW	

读取的观测数	4805
使用的观测数	4805

分类水平信息				
分类	值	设计变量		
whtr_g	4	1	0	0
	3	0	1	0
	2	0	0	0
	1	0	0	1
GENDER	2	1		
	1	0		
ETHNIC	2	1		
	1	0		
H_DIAB01	1	1		
	0	0		
H_AF01	1	1		
	0	0		
H_HYPT01	1	1		
	0	0		
H_LIPID01	1	1		
	0	0		
AI	1	1		
	0	0		
H_DRINK_H01	1	1		
	0	0		
H_SMK_C01	1	1		
	0	0		
IT	1	1		
	0	0		
ET	1	1		
	0	0		

data1: whtr_g with y1_death: adjusted model

PHREG 过程

事件和删失值个数汇总			
合计	事件	删失	删失百分比
4805	190	4615	96.05

收敛状态
满足收敛准则 (GCONV=1E-8)。

模型拟合统计量		
准则	无协变量	带协变量
-2 LOG L	3206.595	2913.856
AIC	3206.595	2947.856
SBC	3206.595	3003.055

检验全局原假设: BETA=0			
检验	卡方	自由度	Pr > 卡方
似然比	292.7386	17	<.0001
评分	404.6438	17	<.0001
Wald	355.3554	17	<.0001

3 型检验			
效应	自由度	Wald 卡方	Pr > 卡方
whtr_g	3	14.8117	0.0020
AGE	1	80.6309	<.0001
GENDER	1	0.6707	0.4128
ETHNIC	1	7.6854	0.0056
H_DIAB01	1	2.9408	0.0864
H_AF01	1	16.8350	<.0001
H_HYPT01	1	0.1411	0.7072
H_LIPID01	1	1.7907	0.1808
AI	1	0.1943	0.6593
H_DRINK_H01	1	2.5988	0.1069
H_SMK_C01	1	0.2163	0.6418
IT	1	4.8279	0.0280
ET	1	0.4862	0.4856
A_NIHSS	1	80.6167	<.0001
IMG_C_TOAST	1	0.1217	0.7272

data1: whtr_g with y1_death: adjusted model

PHREG 过程

最大似然估计分析									
参数		自由度	参数估计	标准误差	卡方	Pr > 卡方	危险率	95% 危险率置信限	
whtr_g	4	1	-0.35704	0.21556	2.7435	0.0977	0.700	0.459	1.068
whtr_g	3	1	-0.86460	0.23755	13.2467	0.0003	0.421	0.264	0.671
whtr_g	1	1	-0.09632	0.18054	0.2846	0.5937	0.908	0.638	1.294
AGE		1	0.07068	0.00787	80.6309	<.0001	1.073	1.057	1.090
GENDER	2	1	-0.13455	0.16430	0.6707	0.4128	0.874	0.633	1.206
ETHNIC	2	1	0.78456	0.28300	7.6854	0.0056	2.191	1.258	3.816
H_DIAB01	1	1	0.29917	0.17446	2.9408	0.0864	1.349	0.958	1.899
H_AF01	1	1	0.76405	0.18621	16.8350	<.0001	2.147	1.490	3.093
H_HYPT01	1	1	0.05937	0.15804	0.1411	0.7072	1.061	0.779	1.446
H_LIPID01	1	1	-0.56086	0.41912	1.7907	0.1808	0.571	0.251	1.298
AI	1	1	0.20453	0.46394	0.1943	0.6593	1.227	0.494	3.046
H_DRINK_H01	1	1	-0.58259	0.36139	2.5988	0.1069	0.558	0.275	1.134
H_SMK_C01	1	1	-0.10103	0.21720	0.2163	0.6418	0.904	0.591	1.384
IT	1	1	-0.56741	0.25824	4.8279	0.0280	0.567	0.342	0.941
ET	1	1	0.41881	0.60065	0.4862	0.4856	1.520	0.468	4.934
A_NIHSS		1	0.08870	0.00988	80.6167	<.0001	1.093	1.072	1.114
IMG_C_TOAST		1	-0.01520	0.04357	0.1217	0.7272	0.985	0.904	1.073

最大似然估计分析		
参数		标签
whtr_g	4	1=Q1(<0.47);2=Q2(0.47-<0.52);3=Q3(0.52-<0.57);4=Q4(>=0.57) 4
whtr_g	3	1=Q1(<0.47);2=Q2(0.47-<0.52);3=Q3(0.52-<0.57);4=Q4(>=0.57) 3
whtr_g	1	1=Q1(<0.47);2=Q2(0.47-<0.52);3=Q3(0.52-<0.57);4=Q4(>=0.57) 1
AGE		A.Basic Information: Age (years old);
GENDER	2	A.Basic Information: Gender; 1-male; 2-female; 2
ETHNIC	2	B.Demography: Race: 1-Han; 99-others; 2
H_DIAB01	1	D.History: Diabetes; 0-No; 1-Yes; 1
H_AF01	1	D.History: Heart disease category: Atrial fibrillation(Including medical history and hospitalization diagnosis); 0-No; 1-Yes; 1
H_HYPT01	1	D.History: Hypertension; 0-No; 1-Yes; 1
H_LIPID01	1	D.History: Lipid metabolism disorders; 0-No; 1-Yes; 1
AI	1	history:Myocardial infarction; 0=NO; 1=YES; 1
H_DRINK_H01	1	D.History: Heavy Drinking(Alcohol consumption>=20g/day); 0-No,1-Yes; 1
H_SMK_C01	1	D.History: Current Smoking; 0-No,1-Yes; 1
IT	1	intravenous thrombolysis, 1=YES,0=NO 1
ET	1	动脉溶栓或机械取栓, 1=YES,0=NO 1
A_NIHSS		F.Admitting NIHSS: Total score;
IMG_C_TOAST		K.Final diagnosis: cerebral infarction; Etiology according to TOAST system; 1-large artery atherosclerosis; 2-cardiogenic embolism; 3-small artery occlusion; 4-stroke of another determined cause; 5-stroke of an undetermined cause.

data1: whtr_g with y1_death: interaction model

PHREG 过程

模型信息		
数据集	WORK.DATA1	
因变量	y1_death_dd	N12.Follow-up events at 12 months: Days from onset to death;(day);
删失变量	y1_death	N12.Follow-up events at 12 months: Whether the patient died: 0-survival;1-death;
删失值	0	
结值处理	BRESLOW	

读取的观测数<br>使用的观测数	4805<br>4805

分类水平信息						
分类	值	设计变量				
whtr_g	4	1	0	0	
	3	0	1	0	
	2	0	0	0	
	1	0	0	1	
IMG_C_TOAST	5	1	0	0	0
	4	0	1	0	0
	3	0	0	1	0
	2	0	0	0	1
	1	0	0	0	0
GENDER	2	1			
	1	0			
ETHNIC	2	1			
	1	0			
H_DIAB01	1	1			
	0	0			
H_AF01	1	1			
	0	0			
H_HYPT01	1	1			
	0	0			
H_LIPID01	1	1			
	0	0			
AI	1	1			
	0	0			
H_DRINK_H01	1	1			
	0	0			
H_SMK_C01	1	1			
	0	0			
IT	1	1			
	0	0			

data1: whtr_g with y1_death: interaction model

PHREG 过程

分类水平信息					
分类	值	设计变量			
ET	1	1			
	0	0			

事件和删失值个数汇总			
合计	事件	删失	删失百分比
4805	190	4615	96.05

收敛状态
满足收敛准则 (GCONV=1E-8)。

模型拟合统计量		
准则	无协变量	带协变量
-2 LOG L	3206.595	2891.059
AIC	3206.595	2955.059
SBC	3206.595	3058.963

检验全局原假设: BETA=0			
检验	卡方	自由度	Pr > 卡方
似然比	315.5360	32	<.0001
评分	441.9023	32	<.0001
Wald	367.2156	32	<.0001

联合检验			
效应	自由度	Wald 卡方	Pr > 卡方
whtr_g	3	8.2223	0.0416
IMG_C_TOAST	4	7.1586	0.1277
whtr_g*IMG_C_TOAST	12	9.3661	0.6714
AGE	1	76.7588	<.0001
GENDER	1	1.0113	0.3146
ETHNIC	1	7.3920	0.0066
H_DIAB01	1	3.9266	0.0475
H_AF01	1	14.9845	0.0001
H_HYPT01	1	0.0789	0.7787
H_LIPID01	1	1.8327	0.1758
AI	1	0.3155	0.5743
H_DRINK_H01	1	2.4976	0.1140
H_SMK_C01	1	0.2898	0.5904
IT	1	4.6407	0.0312

data1: whtr_g with y1_death: interaction model

PHREG 过程

联合检验			
效应	自由度	Wald 卡方	Pr > 卡方
ET	1	0.7099	0.3995
A_NIHSS	1	68.4979	<.0001

Note: Under full-rank parameterizations, Type 3 effect tests are replaced by joint tests. The joint test for an effect is a test that all of the parameters associated with that effect are zero. Such joint tests might not be equivalent to Type 3 effect tests under GLM parameterization.

data1: whtr_g with y1_death: interaction model

PHREG 过程

最大似然估计分析										
参数			自由度	参数估计	标准误差	卡方	Pr > 卡方	危险率	95% 危险率置信限	
whtr_g	4		1	-0.62619	0.38326	2.6695	0.1023	.	.	.
whtr_g	3		1	-1.22722	0.45943	7.1353	0.0076	.	.	.
whtr_g	1		1	-0.45010	0.33997	1.7528	0.1855	.	.	.
IMG_C_TOAST	5		1	-0.70018	0.31122	5.0616	0.0245	.	.	.
IMG_C_TOAST	4		1	0.26140	1.02672	0.0648	0.7990	.	.	.
IMG_C_TOAST	3		1	-0.83920	0.45978	3.3313	0.0680	.	.	.
IMG_C_TOAST	2		1	-0.27596	0.41383	0.4447	0.5049	.	.	.
whtr_g*IMG_C_TOAST	4	5	1	0.78773	0.49565	2.5258	0.1120	.	.	.
whtr_g*IMG_C_TOAST	4	4	1	0.75647	1.46786	0.2656	0.6063	.	.	.
whtr_g*IMG_C_TOAST	4	3	1	-0.01088	0.80330	0.0002	0.9892	.	.	.
whtr_g*IMG_C_TOAST	4	2	1	-0.72652	0.85514	0.7218	0.3956	.	.	.
whtr_g*IMG_C_TOAST	3	5	1	0.87858	0.58025	2.2926	0.1300	.	.	.
whtr_g*IMG_C_TOAST	3	4	1	1.47056	1.31082	1.2586	0.2619	.	.	.
whtr_g*IMG_C_TOAST	3	3	1	0.13329	0.93686	0.0202	0.8869	.	.	.
whtr_g*IMG_C_TOAST	3	2	1	-0.68279	0.89535	0.5816	0.4457	.	.	.
whtr_g*IMG_C_TOAST	1	5	1	0.67036	0.44331	2.2867	0.1305	.	.	.
whtr_g*IMG_C_TOAST	1	4	1	0.63772	1.21362	0.2761	0.5993	.	.	.
whtr_g*IMG_C_TOAST	1	3	1	0.48487	0.67052	0.5229	0.4696	.	.	.
whtr_g*IMG_C_TOAST	1	2	1	0.10423	0.53461	0.0380	0.8454	.	.	.
AGE			1	0.06896	0.00787	76.7588	<.0001	1.071	1.055	1.088
GENDER	2		1	-0.16719	0.16625	1.0113	0.3146	0.846	0.611	1.172
ETHNIC	2		1	0.77478	0.28497	7.3920	0.0066	2.170	1.241	3.794
H_DIAB01	1		1	0.34786	0.17555	3.9266	0.0475	1.416	1.004	1.998
H_AF01	1		1	0.92149	0.23805	14.9845	0.0001	2.513	1.576	4.007
H_HYPT01	1		1	0.04480	0.15946	0.0789	0.7787	1.046	0.765	1.430

data1: whtr_g with y1_death: interaction model

PHREG 过程

最大似然估计分析			
参数			标签
whtr_g	4		1=Q1(<0.47);2=Q2(0.47-<0.52);3=Q3(0.52-<0.57);4=Q4(>=0.57) 4
whtr_g	3		1=Q1(<0.47);2=Q2(0.47-<0.52);3=Q3(0.52-<0.57);4=Q4(>=0.57) 3
whtr_g	1		1=Q1(<0.47);2=Q2(0.47-<0.52);3=Q3(0.52-<0.57);4=Q4(>=0.57) 1
IMG_C_TOAST	5		K.Final diagnosis: cerebral infarction; Etiology according to TOAST system; 1-large artery atherosclerosis; 2-cardiogenic embolism; 3-small artery occlusion; 4-stroke of another determined cause; 5-stroke of an undetermined cause. 5
IMG_C_TOAST	4		K.Final diagnosis: cerebral infarction; Etiology according to TOAST system; 1-large artery atherosclerosis; 2-cardiogenic embolism; 3-small artery occlusion; 4-stroke of another determined cause; 5-stroke of an undetermined cause. 4
IMG_C_TOAST	3		K.Final diagnosis: cerebral infarction; Etiology according to TOAST system; 1-large artery atherosclerosis; 2-cardiogenic embolism; 3-small artery occlusion; 4-stroke of another determined cause; 5-stroke of an undetermined cause. 3
IMG_C_TOAST	2		K.Final diagnosis: cerebral infarction; Etiology according to TOAST system; 1-large artery atherosclerosis; 2-cardiogenic embolism; 3-small artery occlusion; 4-stroke of another determined cause; 5-stroke of an undetermined cause. 2
whtr_g*IMG_C_TOAST	4	5	1=Q1(<0.47);2=Q2(0.47-<0.52);3=Q3(0.52-<0.57);4=Q4(>=0.57) 4 * K.Final diagnosis: cerebral infarction; Etiology according to TOAST system; 1-large artery atherosclerosis; 2-cardiogenic embolism; 3-small artery occlusion; 4-stroke of another determined caus
whtr_g*IMG_C_TOAST	4	4	1=Q1(<0.47);2=Q2(0.47-<0.52);3=Q3(0.52-<0.57);4=Q4(>=0.57) 4 * K.Final diagnosis: cerebral infarction; Etiology according to TOAST system; 1-large artery atherosclerosis; 2-cardiogenic embolism; 3-small artery occlusion; 4-stroke of another determined caus
whtr_g*IMG_C_TOAST	4	3	1=Q1(<0.47);2=Q2(0.47-<0.52);3=Q3(0.52-<0.57);4=Q4(>=0.57) 4 * K.Final diagnosis: cerebral infarction; Etiology according to TOAST system; 1-large artery atherosclerosis; 2-cardiogenic embolism; 3-small artery occlusion; 4-stroke of another determined caus
whtr_g*IMG_C_TOAST	4	2	1=Q1(<0.47);2=Q2(0.47-<0.52);3=Q3(0.52-<0.57);4=Q4(>=0.57) 4 * K.Final diagnosis: cerebral infarction; Etiology according to TOAST system; 1-large artery atherosclerosis; 2-cardiogenic embolism; 3-small artery occlusion; 4-stroke of another determined caus
whtr_g*IMG_C_TOAST	3	5	1=Q1(<0.47);2=Q2(0.47-<0.52);3=Q3(0.52-<0.57);4=Q4(>=0.57) 3 * K.Final diagnosis: cerebral infarction; Etiology according to TOAST system; 1-large artery atherosclerosis; 2-cardiogenic embolism; 3-small artery occlusion; 4-stroke of another determined caus
whtr_g*IMG_C_TOAST	3	4	1=Q1(<0.47);2=Q2(0.47-<0.52);3=Q3(0.52-<0.57);4=Q4(>=0.57) 3 * K.Final diagnosis: cerebral infarction; Etiology according to TOAST system; 1-large artery atherosclerosis; 2-cardiogenic embolism; 3-small artery occlusion; 4-stroke of another determined caus
whtr_g*IMG_C_TOAST	3	3	1=Q1(<0.47);2=Q2(0.47-<0.52);3=Q3(0.52-<0.57);4=Q4(>=0.57) 3 * K.Final diagnosis: cerebral infarction; Etiology according to TOAST system; 1-large artery atherosclerosis; 2-cardiogenic embolism; 3-small artery occlusion; 4-stroke of another determined caus
whtr_g*IMG_C_TOAST	3	2	1=Q1(<0.47);2=Q2(0.47-<0.52);3=Q3(0.52-<0.57);4=Q4(>=0.57) 3 * K.Final diagnosis: cerebral infarction; Etiology according to TOAST system; 1-large artery atherosclerosis; 2-cardiogenic embolism; 3-small artery occlusion; 4-stroke of another determined caus
whtr_g*IMG_C_TOAST	1	5	1=Q1(<0.47);2=Q2(0.47-<0.52);3=Q3(0.52-<0.57);4=Q4(>=0.57) 1 * K.Final diagnosis: cerebral infarction; Etiology according to TOAST system; 1-large artery atherosclerosis; 2-cardiogenic embolism; 3-small artery occlusion; 4-stroke of another determined caus
whtr_g*IMG_C_TOAST	1	4	1=Q1(<0.47);2=Q2(0.47-<0.52);3=Q3(0.52-<0.57);4=Q4(>=0.57) 1 * K.Final diagnosis: cerebral infarction; Etiology according to TOAST system; 1-large artery atherosclerosis; 2-cardiogenic embolism; 3-small artery occlusion; 4-stroke of another determined caus
whtr_g*IMG_C_TOAST	1	3	1=Q1(<0.47);2=Q2(0.47-<0.52);3=Q3(0.52-<0.57);4=Q4(>=0.57) 1 * K.Final diagnosis: cerebral infarction; Etiology according to TOAST system; 1-large artery atherosclerosis; 2-cardiogenic embolism; 3-small artery occlusion; 4-stroke of another determined caus
whtr_g*IMG_C_TOAST	1	2	1=Q1(<0.47);2=Q2(0.47-<0.52);3=Q3(0.52-<0.57);4=Q4(>=0.57) 1 * K.Final diagnosis: cerebral infarction; Etiology according to TOAST system; 1-large artery atherosclerosis; 2-cardiogenic embolism; 3-small artery occlusion; 4-stroke of another determined caus
AGE			A.Basic Information: Age (years old);
GENDER	2		A.Basic Information: Gender; 1-male; 2-female; 2
ETHNIC	2		B.Demography: Race: 1-Han; 99-others; 2
H_DIAB01	1		D.History: Diabetes; 0-No; 1-Yes; 1
H_AF01	1		D.History: Heart disease category: Atrial fibrillation(Including medical history and hospitalization diagnosis); 0-No; 1-Yes; 1
H_HYPT01	1		D.History: Hypertension; 0-No; 1-Yes; 1

data1: whtr_g with y1_death: interaction model

PHREG 过程

最大似然估计分析										
参数			自由度	参数估计	标准误差	卡方	Pr > 卡方	危险率	95% 危险率置信限	
H_LIPID01	1		1	-0.56934	0.42057	1.8327	0.1758	0.566	0.248	1.290
AI	1		1	0.26205	0.46653	0.3155	0.5743	1.300	0.521	3.243
H_DRINK_H01	1		1	-0.57382	0.36309	2.4976	0.1140	0.563	0.277	1.148
H_SMK_C01	1		1	-0.11759	0.21845	0.2898	0.5904	0.889	0.579	1.364
IT	1		1	-0.55883	0.25941	4.6407	0.0312	0.572	0.344	0.951
ET	1		1	0.50672	0.60139	0.7099	0.3995	1.660	0.511	5.395
A_NIHSS			1	0.08511	0.01028	68.4979	<.0001	1.089	1.067	1.111

data1: whtr_g with y1_death: interaction model

PHREG 过程

最大似然估计分析			
参数			标签
H_LIPID01	1		D.History: Lipid metabolism disorders; 0-No; 1-Yes; 1
AI	1		history:Myocardial infarction; 0=NO; 1=YES; 1
H_DRINK_H01	1		D.History: Heavy Drinking(Alcohol consumption>=20g/day); 0-No,1-Yes; 1
H_SMK_C01	1		D.History: Current Smoking; 0-No,1-Yes; 1
IT	1		intravenous thrombolysis, 1=YES,0=NO 1
ET	1		动脉溶栓或机械取栓, 1=YES,0=NO 1
A_NIHSS			F.Admitting NIHSS: Total score;

data1: whtr with y1_comb: crude model

PHREG 过程

模型信息		
数据集	WORK.DATA1	
因变量	y1_comb_dd	N12.Follow-up events at 12 months: Days from onset to occurrence of combined vascular event;(day);
删失变量	y1_comb	N12.Follow-up events at 12 months:Occurrence of combined vascular event(including cardiovascular death,non-fatal stroke,non-fatal myocardial infarction):0-No;1-Yes;
删失值	0	
结值处理	BRESLOW	

读取的观测数	4805
使用的观测数	4805

事件和删失值个数汇总			
合计	事件	删失	删失百分比
4805	520	4285	89.18

收敛状态
满足收敛准则 (GCONV=1E-8)。

模型拟合统计量		
准则	无协变量	带协变量
-2 LOG L	8739.718	8736.394
AIC	8739.718	8738.394
SBC	8739.718	8742.647

检验全局原假设: BETA=0			
检验	卡方	自由度	Pr > 卡方
似然比	3.3245	1	0.0683
评分	3.3074	1	0.0690
Wald	3.3188	1	0.0685

最大似然估计分析								
参数	自由度	参数估计	标准误差	卡方	Pr > 卡方	危险率	95% 危险率置信限	
whtr	1	0.94195	0.51706	3.3188	0.0685	2.565	0.931	7.066

data1: whtr with y1_comb: adjusted model

PHREG 过程

模型信息		
数据集	WORK.DATA1	
因变量	y1_comb_dd	N12.Follow-up events at 12 months: Days from onset to occurrence of combined vascular event;(day);
删失变量	y1_comb	N12.Follow-up events at 12 months:Occurrence of combined vascular event(including cardiovascular death,non-fatal stroke,non-fatal myocardial infarction):0-No;1-Yes;
删失值	0	
结值处理	BRESLOW	

读取的观测数	4805
使用的观测数	4805

分类水平信息		
分类	值	设计变量
GENDER	2	1
	1	0
ETHNIC	2	1
	1	0
H_DIAB01	1	1
	0	0
H_AF01	1	1
	0	0
H_HYPT01	1	1
	0	0
H_LIPID01	1	1
	0	0
AI	1	1
	0	0
H_DRINK_H01	1	1
	0	0
H_SMK_C01	1	1
	0	0
IT	1	1
	0	0
ET	1	1
	0	0

事件和删失值个数汇总			
合计	事件	删失	删失百分比
4805	520	4285	89.18

收敛状态
满足收敛准则 (GCONV=1E-8)。

data1: whtr with y1_comb: adjusted model

PHREG 过程

模型拟合统计量		
准则	无<br>协变量	带<br>协变量
-2 LOG L	8739.718	8686.408
AIC	8739.718	8716.408
SBC	8739.718	8780.216

检验全局原假设: BETA=0			
检验	卡方	自由度	Pr > 卡方
似然比	53.3098	15	<.0001
评分	57.1770	15	<.0001
Wald	56.5425	15	<.0001

3 型检验			
效应	自由度	Wald 卡方	Pr > 卡方
whtr	1	5.2201	0.0223
AGE	1	3.4965	0.0615
GENDER	1	2.3505	0.1252
ETHNIC	1	0.9030	0.3420
H_DIAB01	1	13.1057	0.0003
H_AF01	1	3.7144	0.0539
H_HYPT01	1	0.0170	0.8963
H_LIPID01	1	0.6509	0.4198
AI	1	0.8928	0.3447
H_DRINK_H01	1	1.6509	0.1988
H_SMK_C01	1	2.6369	0.1044
IT	1	0.0065	0.9356
ET	1	1.5944	0.2067
A_NIHSS	1	12.1883	0.0005
IMG_C_TOAST	1	3.2206	0.0727

data1: whtr with y1_comb: adjusted model

PHREG 过程

最大似然估计分析									
参数		自由度	参数估计	标准误差	卡方	Pr > 卡方	危险率	95% 危险率置信限	
whtr		1	1.19491	0.52299	5.2201	0.0223	3.303	1.185	9.207
AGE		1	0.00779	0.00416	3.4965	0.0615	1.008	1.000	1.016
GENDER	2	1	-0.15861	0.10346	2.3505	0.1252	0.853	0.697	1.045
ETHNIC	2	1	-0.21792	0.22932	0.9030	0.3420	0.804	0.513	1.261
H_DIAB01	1	1	0.35800	0.09889	13.1057	0.0003	1.430	1.178	1.736
H_AF01	1	1	0.28440	0.14757	3.7144	0.0539	1.329	0.995	1.775
H_HYPT01	1	1	0.01232	0.09447	0.0170	0.8963	1.012	0.841	1.218
H_LIPID01	1	1	-0.14869	0.18430	0.6509	0.4198	0.862	0.601	1.237
AI	1	1	-0.36337	0.38456	0.8928	0.3447	0.695	0.327	1.478
H_DRINK_H01	1	1	0.18433	0.14346	1.6509	0.1988	1.202	0.908	1.593
H_SMK_C01	1	1	-0.19103	0.11764	2.6369	0.1044	0.826	0.656	1.040
IT	1	1	0.01131	0.14002	0.0065	0.9356	1.011	0.769	1.331
ET	1	1	0.53192	0.42126	1.5944	0.2067	1.702	0.745	3.887
A_NIHSS		1	0.03305	0.00947	12.1883	0.0005	1.034	1.015	1.053
IMG_C_TOAST		1	-0.04723	0.02632	3.2206	0.0727	0.954	0.906	1.004

最大似然估计分析		
参数		标签
whtr		
AGE		A.Basic Information: Age (years old);
GENDER	2	A.Basic Information: Gender; 1-male; 2-female; 2
ETHNIC	2	B.Demography: Race: 1-Han; 99-others; 2
H_DIAB01	1	D.History: Diabetes; 0-No; 1-Yes; 1
H_AF01	1	D.History: Heart disease category: Atrial fibrillation(Including medical history and hospitalization diagnosis); 0-No; 1-Yes; 1
H_HYPT01	1	D.History: Hypertension; 0-No; 1-Yes; 1
H_LIPID01	1	D.History: Lipid metabolism disorders; 0-No; 1-Yes; 1
AI	1	history:Myocardial infarction; 0=NO; 1=YES; 1
H_DRINK_H01	1	D.History: Heavy Drinking(Alcohol consumption>=20g/day); 0-No,1-Yes; 1
H_SMK_C01	1	D.History: Current Smoking; 0-No,1-Yes; 1
IT	1	intravenous thrombolysis, 1=YES,0=NO 1
ET	1	动脉溶栓或机械取栓, 1=YES,0=NO 1
A_NIHSS		F.Admitting NIHSS: Total score;
IMG_C_TOAST		K.Final diagnosis: cerebral infarction; Etiology according to TOAST system; 1-large artery atherosclerosis; 2-cardiogenic embolism; 3-small artery occlusion; 4-stroke of another determined cause; 5-stroke of an undetermined cause.

data1: whtr with y1_comb: interaction model

PHREG 过程

模型信息		
数据集	WORK.DATA1	
因变量	y1_comb_dd	N12.Follow-up events at 12 months: Days from onset to occurrence of combined vascular event;(day);
删失变量	y1_comb	N12.Follow-up events at 12 months:Occurrence of combined vascular event(including cardiovascular death,non-fatal stroke,non-fatal myocardial infarction):0-No;1-Yes;
删失值	0	
结值处理	BRESLOW	

读取的观测数	4805
使用的观测数	4805

分类水平信息						
分类	值	设计变量				
GENDER	2	1				
	1	0				
IMG_C_TOAST	5	1	0	0	0	
	4	0	1	0	0	
	3	0	0	1	0	
	2	0	0	0	1	
	1	0	0	0	0	
ETHNIC	2	1				
	1	0				
H_DIAB01	1	1				
	0	0				
H_AF01	1	1				
	0	0				
H_HYPT01	1	1				
	0	0				
H_LIPID01	1	1				
	0	0				
AI	1	1				
	0	0				
H_DRINK_H01	1	1				
	0	0				
H_SMK_C01	1	1				
	0	0				
IT	1	1				
	0	0				
ET	1	1				
	0	0				

data1: whtr with y1_comb: interaction model

PHREG 过程

事件和删失值个数汇总			
合计	事件	删失	删失百分比
4805	520	4285	89.18

收敛状态
满足收敛准则 (GCONV=1E-8)。

模型拟合统计量		
准则	无协变量	带协变量
-2 LOG L	8739.718	8669.748
AIC	8739.718	8713.748
SBC	8739.718	8807.332

检验全局原假设: BETA=0			
检验	卡方	自由度	Pr > 卡方
似然比	69.9700	22	<.0001
评分	77.2623	22	<.0001
Wald	76.5300	22	<.0001

联合检验			
效应	自由度	Wald 卡方	Pr > 卡方
whtr	1	10.3770	0.0013
IMG_C_TOAST	4	2.7069	0.6080
whtr*IMG_C_TOAST	4	5.1184	0.2754
AGE	1	3.4038	0.0650
GENDER	1	2.9827	0.0842
ETHNIC	1	0.8003	0.3710
H_DIAB01	1	13.1560	0.0003
H_AF01	1	9.8407	0.0017
H_HYPT01	1	0.0303	0.8618
H_LIPID01	1	0.6903	0.4061
AI	1	0.8471	0.3574
H_DRINK_H01	1	1.5399	0.2146
H_SMK_C01	1	2.6450	0.1039
IT	1	0.0070	0.9333
ET	1	1.7799	0.1822
A_NIHSS	1	9.0902	0.0026

Note: Under full-rank parameterizations, Type 3 effect tests are replaced by joint tests. The joint test for an effect is a test that all of the parameters associated with that effect are zero. Such joint tests might not be equivalent to Type 3 effect tests under GLM parameterization.

data1: whtr with y1_comb: interaction model

PHREG 过程

最大似然估计分析

参数		自由度	参数估计	标准误差	卡方	Pr > 卡方	危险率	95% 危险率置信限	
whtr		1	2.68345	0.83302	10.3770	0.0013	.	.	.
IMG_C_TOAST	5	1	1.00985	0.61586	2.6887	0.1011	.	.	.
IMG_C_TOAST	4	1	0.73132	2.39909	0.0929	0.7605	.	.	.
IMG_C_TOAST	3	1	0.60708	0.80116	0.5742	0.4486	.	.	.
IMG_C_TOAST	2	1	0.65001	1.09400	0.3530	0.5524	.	.	.
whtr*IMG_C_TOAST	5	1	-2.47768	1.15143	4.6304	0.0314	.	.	.
whtr*IMG_C_TOAST	4	1	-1.17731	4.62126	0.0649	0.7989	.	.	.
whtr*IMG_C_TOAST	3	1	-1.82332	1.49371	1.4900	0.2222	.	.	.
whtr*IMG_C_TOAST	2	1	-2.53341	2.09741	1.4590	0.2271	.	.	.
AGE		1	0.00766	0.00415	3.4038	0.0650	1.008	1.000	1.016
GENDER	2	1	-0.17955	0.10396	2.9827	0.0842	0.836	0.682	1.025
ETHNIC	2	1	-0.20506	0.22923	0.8003	0.3710	0.815	0.520	1.277
H_DIAB01	1	1	0.35931	0.09906	13.1560	0.0003	1.432	1.180	1.739
H_AF01	1	1	0.58429	0.18626	9.8407	0.0017	1.794	1.245	2.584
H_HYPT01	1	1	0.01644	0.09447	0.0303	0.8618	1.017	0.845	1.223
H_LIPID01	1	1	-0.15328	0.18448	0.6903	0.4061	0.858	0.598	1.232
AI	1	1	-0.35410	0.38474	0.8471	0.3574	0.702	0.330	1.492
H_DRINK_H01	1	1	0.17827	0.14366	1.5399	0.2146	1.195	0.902	1.584
H_SMK_C01	1	1	-0.19146	0.11772	2.6450	0.1039	0.826	0.656	1.040
IT	1	1	0.01176	0.14051	0.0070	0.9333	1.012	0.768	1.333
ET	1	1	0.56271	0.42177	1.7799	0.1822	1.755	0.768	4.012
A_NIHSS		1	0.02897	0.00961	9.0902	0.0026	1.029	1.010	1.049

data1: whtr with y1_comb: interaction model

PHREG 过程

最大似然估计分析		
参数		标签
whtr		
IMG_C_TOAST	5	K.Final diagnosis: cerebral infarction; Etiology according to TOAST system; 1-large artery atherosclerosis; 2-cardiogenic embolism; 3-small artery occlusion; 4-stroke of another determined cause; 5-stroke of an undetermined cause. 5
IMG_C_TOAST	4	K.Final diagnosis: cerebral infarction; Etiology according to TOAST system; 1-large artery atherosclerosis; 2-cardiogenic embolism; 3-small artery occlusion; 4-stroke of another determined cause; 5-stroke of an undetermined cause. 4
IMG_C_TOAST	3	K.Final diagnosis: cerebral infarction; Etiology according to TOAST system; 1-large artery atherosclerosis; 2-cardiogenic embolism; 3-small artery occlusion; 4-stroke of another determined cause; 5-stroke of an undetermined cause. 3
IMG_C_TOAST	2	K.Final diagnosis: cerebral infarction; Etiology according to TOAST system; 1-large artery atherosclerosis; 2-cardiogenic embolism; 3-small artery occlusion; 4-stroke of another determined cause; 5-stroke of an undetermined cause. 2
whtr*IMG_C_TOAST	5	K.Final diagnosis: cerebral infarction; Etiology according to TOAST system; 1-large artery atherosclerosis; 2-cardiogenic embolism; 3-small artery occlusion; 4-stroke of another determined cause; 5-stroke of an undetermined cause. 5 * whtr
whtr*IMG_C_TOAST	4	K.Final diagnosis: cerebral infarction; Etiology according to TOAST system; 1-large artery atherosclerosis; 2-cardiogenic embolism; 3-small artery occlusion; 4-stroke of another determined cause; 5-stroke of an undetermined cause. 4 * whtr
whtr*IMG_C_TOAST	3	K.Final diagnosis: cerebral infarction; Etiology according to TOAST system; 1-large artery atherosclerosis; 2-cardiogenic embolism; 3-small artery occlusion; 4-stroke of another determined cause; 5-stroke of an undetermined cause. 3 * whtr
whtr*IMG_C_TOAST	2	K.Final diagnosis: cerebral infarction; Etiology according to TOAST system; 1-large artery atherosclerosis; 2-cardiogenic embolism; 3-small artery occlusion; 4-stroke of another determined cause; 5-stroke of an undetermined cause. 2 * whtr
AGE		A.Basic Information: Age (years old);
GENDER	2	A.Basic Information: Gender; 1-male; 2-female; 2
ETHNIC	2	B.Demography: Race: 1-Han; 99-others; 2
H_DIAB01	1	D.History: Diabetes; 0-No; 1-Yes; 1
H_AF01	1	D.History: Heart disease category: Atrial fibrillation(Including medical history and hospitalization diagnosis); 0-No; 1-Yes; 1
H_HYPT01	1	D.History: Hypertension; 0-No; 1-Yes; 1
H_LIPID01	1	D.History: Lipid metabolism disorders; 0-No; 1-Yes; 1
AI	1	history:Myocardial infarction; 0=NO; 1=YES; 1
H_DRINK_H01	1	D.History: Heavy Drinking(Alcohol consumption>=20g/day); 0-No,1-Yes; 1
H_SMK_C01	1	D.History: Current Smoking; 0-No,1-Yes; 1
IT	1	intravenous thrombolysis, 1=YES,0=NO 1
ET	1	动脉溶栓或机械取栓, 1=YES,0=NO 1
A_NIHSS		F.Admitting NIHSS: Total score;

data1: whtr_g with y1_comb: Descriptive results

FREQ 过程

频数<br>行百分比

whtr_g-y1_comb表			
	y1_comb(N12.Follow-up events at 12 months:Occurrence of combined vascular event(including cardiovascular death,non-fatal stroke,non-fatal myocardial infarction):0-No;1-Yes;)		
whtr_g(1=Q1(<0.47);2=Q2(0.47-<0.52);3=Q3(0.52-<0.57);4=Q4(>=0.57))	0	1	合计
1	1054<br>90.16	115<br>9.84	1169
2	1158<br>87.99	158<br>12.01	1316
3	1083<br>91.01	107<br>8.99	1190
4	990<br>87.61	140<br>12.39	1130
合计	4285	520	4805

表“y1_comb-whtr_g”的统计量

统计量	自由度	值	概率
卡方	3	10.0936	0.0178
似然比卡方检验	3	10.1789	0.0171
Mantel-Haenszel 卡方	1	1.1304	0.2877
Phi 系数		0.0458	
列联系数		0.0458	
Cramer V		0.0458	

样本大小 = 4805

data1: whtr_g with y1_comb: crude model

PHREG 过程

模型信息		
数据集	WORK.DATA1	
因变量	y1_comb_dd	N12.Follow-up events at 12 months: Days from onset to occurrence of combined vascular event;(day);
删失变量	y1_comb	N12.Follow-up events at 12 months:Occurrence of combined vascular event(including cardiovascular death,non-fatal stroke,non-fatal myocardial infarction):0-No;1-Yes;
删失值	0	
结值处理	BRESLOW	

读取的观测数	4805
使用的观测数	4805

分类水平信息				
分类	值	设计变量		
whtr_g	4	1	0	0
	3	0	1	0
	2	0	0	0
	1	0	0	1

事件和删失值个数汇总			
合计	事件	删失	删失百分比
4805	520	4285	89.18

收敛状态
满足收敛准则 (GCONV=1E-8)。

模型拟合统计量		
准则	无协变量	带协变量
-2 LOG L	8739.718	8729.633
AIC	8739.718	8735.633
SBC	8739.718	8748.395

检验全局原假设: BETA=0			
检验	卡方	自由度	Pr > 卡方
似然比	10.0848	3	0.0179
评分	9.9758	3	0.0188
Wald	9.9037	3	0.0194

3 型检验			
效应	自由度	Wald 卡方	Pr > 卡方
whtr_g	3	9.9037	0.0194

data1: whtr_g with y1_comb: crude model

PHREG 过程

最大似然估计分析									
参数		自由度	参数估计	标准误差	卡方	Pr > 卡方	危险率	95% 危险率置信限	
whtr_g	4	1	0.03582	0.11607	0.0953	0.7576	1.036	0.826	1.301
whtr_g	3	1	-0.30651	0.12520	5.9932	0.0144	0.736	0.576	0.941
whtr_g	1	1	-0.20131	0.12258	2.6973	0.1005	0.818	0.643	1.040

最大似然估计分析		
参数		标签
whtr_g	4	1=Q1(<0.47);2=Q2(0.47-<0.52);3=Q3(0.52-<0.57);4=Q4(>=0.57) 4
whtr_g	3	1=Q1(<0.47);2=Q2(0.47-<0.52);3=Q3(0.52-<0.57);4=Q4(>=0.57) 3
whtr_g	1	1=Q1(<0.47);2=Q2(0.47-<0.52);3=Q3(0.52-<0.57);4=Q4(>=0.57) 1

data1: whtr_g with y1_comb: adjusted model

PHREG 过程

模型信息		
数据集	WORK.DATA1	
因变量	y1_comb_dd	N12.Follow-up events at 12 months: Days from onset to occurrence of combined vascular event;(day);
删失变量	y1_comb	N12.Follow-up events at 12 months:Occurrence of combined vascular event(including cardiovascular death,non-fatal stroke,non-fatal myocardial infarction):0-No;1-Yes;
删失值	0	
结值处理	BRESLOW	

读取的观测数	4805
使用的观测数	4805

分类水平信息				
分类	值	设计变量		
whtr_g	4	1	0	0
	3	0	1	0
	2	0	0	0
	1	0	0	1
GENDER	2	1		
	1	0		
ETHNIC	2	1		
	1	0		
H_DIAB01	1	1		
	0	0		
H_AF01	1	1		
	0	0		
H_HYPT01	1	1		
	0	0		
H_LIPID01	1	1		
	0	0		
AI	1	1		
	0	0		
H_DRINK_H01	1	1		
	0	0		
H_SMK_C01	1	1		
	0	0		
IT	1	1		
	0	0		
ET	1	1		
	0	0		

data1: whtr_g with y1_comb: adjusted model

PHREG 过程

事件和删失值个数汇总			
合计	事件	删失	删失百分比
4805	520	4285	89.18

收敛状态
满足收敛准则 (GCONV=1E-8)。

模型拟合统计量		
准则	无协变量	带协变量
-2 LOG L	8739.718	8679.901
AIC	8739.718	8713.901
SBC	8739.718	8786.216

检验全局原假设: BETA=0			
检验	卡方	自由度	Pr > 卡方
似然比	59.8174	17	<.0001
评分	63.6451	17	<.0001
Wald	62.8519	17	<.0001

3 型检验			
效应	自由度	Wald 卡方	Pr > 卡方
whtr_g	3	11.6158	0.0088
AGE	1	3.0911	0.0787
GENDER	1	2.0809	0.1491
ETHNIC	1	0.8639	0.3526
H_DIAB01	1	13.0836	0.0003
H_AF01	1	4.0221	0.0449
H_HYPT01	1	0.0223	0.8814
H_LIPID01	1	0.6066	0.4361
AI	1	0.7698	0.3803
H_DRINK_H01	1	1.5680	0.2105
H_SMK_C01	1	2.6568	0.1031
IT	1	0.0013	0.9717
ET	1	1.4683	0.2256
A_NIHSS	1	12.5997	0.0004
IMG_C_TOAST	1	3.2180	0.0728

data1: whtr_g with y1_comb: adjusted model

PHREG 过程

最大似然估计分析									
参数		自由度	参数估计	标准误差	卡方	Pr > 卡方	危险率	95% 危险率置信限	
whtr_g	4	1	0.06734	0.11791	0.3262	0.5679	1.070	0.849	1.348
whtr_g	3	1	-0.29378	0.12567	5.4648	0.0194	0.745	0.583	0.954
whtr_g	1	1	-0.24007	0.12306	3.8058	0.0511	0.787	0.618	1.001
AGE		1	0.00733	0.00417	3.0911	0.0787	1.007	0.999	1.016
GENDER	2	1	-0.14934	0.10353	2.0809	0.1491	0.861	0.703	1.055
ETHNIC	2	1	-0.21312	0.22928	0.8639	0.3526	0.808	0.516	1.267
H_DIAB01	1	1	0.35777	0.09891	13.0836	0.0003	1.430	1.178	1.736
H_AF01	1	1	0.29622	0.14770	4.0221	0.0449	1.345	1.007	1.796
H_HYPT01	1	1	0.01410	0.09450	0.0223	0.8814	1.014	0.843	1.221
H_LIPID01	1	1	-0.14358	0.18436	0.6066	0.4361	0.866	0.604	1.243
AI	1	1	-0.33744	0.38459	0.7698	0.3803	0.714	0.336	1.516
H_DRINK_H01	1	1	0.18012	0.14384	1.5680	0.2105	1.197	0.903	1.587
H_SMK_C01	1	1	-0.19218	0.11790	2.6568	0.1031	0.825	0.655	1.040
IT	1	1	0.00497	0.14020	0.0013	0.9717	1.005	0.764	1.323
ET	1	1	0.51012	0.42099	1.4683	0.2256	1.665	0.730	3.801
A_NIHSS		1	0.03354	0.00945	12.5997	0.0004	1.034	1.015	1.053
IMG_C_TOAST		1	-0.04718	0.02630	3.2180	0.0728	0.954	0.906	1.004

最大似然估计分析		
参数		标签
whtr_g	4	1=Q1(<0.47);2=Q2(0.47-<0.52);3=Q3(0.52-<0.57);4=Q4(>=0.57) 4
whtr_g	3	1=Q1(<0.47);2=Q2(0.47-<0.52);3=Q3(0.52-<0.57);4=Q4(>=0.57) 3
whtr_g	1	1=Q1(<0.47);2=Q2(0.47-<0.52);3=Q3(0.52-<0.57);4=Q4(>=0.57) 1
AGE		A.Basic Information: Age (years old);
GENDER	2	A.Basic Information: Gender; 1-male; 2-female; 2
ETHNIC	2	B.Demography: Race: 1-Han; 99-others; 2
H_DIAB01	1	D.History: Diabetes; 0-No; 1-Yes; 1
H_AF01	1	D.History: Heart disease category: Atrial fibrillation(Including medical history and hospitalization diagnosis); 0-No; 1-Yes; 1
H_HYPT01	1	D.History: Hypertension; 0-No; 1-Yes; 1
H_LIPID01	1	D.History: Lipid metabolism disorders; 0-No; 1-Yes; 1
AI	1	history:Myocardial infarction; 0=NO; 1=YES; 1
H_DRINK_H01	1	D.History: Heavy Drinking(Alcohol consumption>=20g/day); 0-No,1-Yes; 1
H_SMK_C01	1	D.History: Current Smoking; 0-No,1-Yes; 1
IT	1	intravenous thrombolysis, 1=YES,0=NO 1
ET	1	动脉溶栓或机械取栓, 1=YES,0=NO 1
A_NIHSS		F.Admitting NIHSS: Total score;
IMG_C_TOAST		K.Final diagnosis: cerebral infarction; Etiology according to TOAST system; 1-large artery atherosclerosis; 2-cardiogenic embolism; 3-small artery occlusion; 4-stroke of another determined cause; 5-stroke of an undetermined cause.

data1: whtr_g with y1_comb: interaction model

PHREG 过程

模型信息		
数据集	WORK.DATA1	
因变量	y1_comb_dd	N12.Follow-up events at 12 months: Days from onset to occurrence of combined vascular event;(day);
删失变量	y1_comb	N12.Follow-up events at 12 months:Occurrence of combined vascular event(including cardiovascular death,non-fatal stroke,non-fatal myocardial infarction):0-No;1-Yes;
删失值	0	
结值处理	BRESLOW	

读取的观测数	4805
使用的观测数	4805

分类水平信息					
分类	值	设计变量			
whtr_g	4	1	0	0	
	3	0	1	0	
	2	0	0	0	
	1	0	0	1	
IMG_C_TOAST	5	1	0	0	0
	4	0	1	0	0
	3	0	0	1	0
	2	0	0	0	1
	1	0	0	0	0
GENDER	2	1			
	1	0			
ETHNIC	2	1			
	1	0			
H_DIAB01	1	1			
	0	0			
H_AF01	1	1			
	0	0			
H_HYPT01	1	1			
	0	0			
H_LIPID01	1	1			
	0	0			
AI	1	1			
	0	0			
H_DRINK_H01	1	1			
	0	0			
H_SMK_C01	1	1			
	0	0			
IT	1	1			
	0	0			

data1: whtr_g with y1_comb: interaction model

PHREG 过程

分类水平信息					
分类	值	设计变量			
ET	1	1			
	0	0			

事件和删失值个数汇总			
合计	事件	删失	删失<br>百分比
4805	520	4285	89.18

收敛状态
满足收敛准则 (GCONV=1E-8)。

模型拟合统计量		
准则	无<br>协变量	带<br>协变量
-2 LOG L	8739.718	8653.882
AIC	8739.718	8717.882
SBC	8739.718	8854.004

检验全局原假设: BETA=0			
检验	卡方	自由度	Pr > 卡方
似然比	85.8363	32	<.0001
评分	90.4255	32	<.0001
Wald	85.8389	32	<.0001

联合检验			
效应	自由度	Wald 卡方	Pr > 卡方
whtr_g	3	10.1117	0.0176
IMG_C_TOAST	4	7.1498	0.1282
whtr_g*IMG_C_TOAST	12	6.6441	0.8802
AGE	1	2.8668	0.0904
GENDER	1	2.1973	0.1382
ETHNIC	1	0.8978	0.3434
H_DIAB01	1	13.4597	0.0002
H_AF01	1	9.8290	0.0017
H_HYPT01	1	0.0235	0.8781
H_LIPID01	1	0.6069	0.4360
AI	1	0.6636	0.4153
H_DRINK_H01	1	1.3722	0.2414
H_SMK_C01	1	2.5306	0.1117
IT	1	0.0084	0.9270

data1: whtr_g with y1_comb: interaction model

PHREG 过程

联合检验			
效应	自由度	Wald 卡方	Pr > 卡方
ET	1	1.8182	0.1775
A_NIHSS	1	9.6247	0.0019

Note: Under full-rank parameterizations, Type 3 effect tests are replaced by joint tests. The joint test for an effect is a test that all of the parameters associated with that effect are zero. Such joint tests might not be equivalent to Type 3 effect tests under GLM parameterization.

data1: whtr_g with y1_comb: interaction model

PHREG 过程

最大似然估计分析										
参数			自由度	参数<br>估计	标准<br>误差	卡方	Pr > 卡方	危险<br>率	95%<br>危险率置信限	
whtr_g	4		1	0.06422	0.19843	0.1047	0.7462	.	.	.
whtr_g	3		1	-0.38543	0.22275	2.9941	0.0836	.	.	.
whtr_g	1		1	-0.58530	0.23847	6.0243	0.0141	.	.	.
IMG_C_TOAST	5		1	-0.47356	0.18791	6.3513	0.0117	.	.	.
IMG_C_TOAST	4		1	-11.32018	163.02812	0.0048	0.9446	.	.	.
IMG_C_TOAST	3		1	-0.38747	0.23303	2.7648	0.0964	.	.	.
IMG_C_TOAST	2		1	-0.50137	0.33458	2.2456	0.1340	.	.	.
whtr_g*IMG_C_TOAST	4	5	1	0.10193	0.27038	0.1421	0.7062	.	.	.
whtr_g*IMG_C_TOAST	4	4	1	11.26536	163.02972	0.0048	0.9449	.	.	.
whtr_g*IMG_C_TOAST	4	3	1	-0.05990	0.33223	0.0325	0.8569	.	.	.
whtr_g*IMG_C_TOAST	4	2	1	-0.67261	0.55806	1.4527	0.2281	.	.	.
whtr_g*IMG_C_TOAST	3	5	1	0.17750	0.29865	0.3532	0.5523	.	.	.
whtr_g*IMG_C_TOAST	3	4	1	12.04090	163.02898	0.0055	0.9411	.	.	.
whtr_g*IMG_C_TOAST	3	3	1	-0.00758	0.37150	0.0004	0.9837	.	.	.
whtr_g*IMG_C_TOAST	3	2	1	-0.09657	0.48275	0.0400	0.8414	.	.	.
whtr_g*IMG_C_TOAST	1	5	1	0.57972	0.30128	3.7026	0.0543	.	.	.
whtr_g*IMG_C_TOAST	1	4	1	12.14781	163.02900	0.0056	0.9406	.	.	.
whtr_g*IMG_C_TOAST	1	3	1	0.29998	0.38115	0.6194	0.4313	.	.	.
whtr_g*IMG_C_TOAST	1	2	1	0.01675	0.49076	0.0012	0.9728	.	.	.
AGE			1	0.00704	0.00416	2.8668	0.0904	1.007	0.999	1.015
GENDER	2		1	-0.15418	0.10401	2.1973	0.1382	0.857	0.699	1.051
ETHNIC	2		1	-0.21747	0.22951	0.8978	0.3434	0.805	0.513	1.262
H_DIAB01	1		1	0.36371	0.09914	13.4597	0.0002	1.439	1.185	1.747
H_AF01	1		1	0.58692	0.18721	9.8290	0.0017	1.798	1.246	2.596
H_HYPT01	1		1	0.01450	0.09458	0.0235	0.8781	1.015	0.843	1.221

data1: whtr_g with y1_comb: interaction model

PHREG 过程

最大似然估计分析			
参数			标签
whtr_g	4		1=Q1(<0.47);2=Q2(0.47-<0.52);3=Q3(0.52-<0.57);4=Q4(>=0.57) 4
whtr_g	3		1=Q1(<0.47);2=Q2(0.47-<0.52);3=Q3(0.52-<0.57);4=Q4(>=0.57) 3
whtr_g	1		1=Q1(<0.47);2=Q2(0.47-<0.52);3=Q3(0.52-<0.57);4=Q4(>=0.57) 1
IMG_C_TOAST	5		K.Final diagnosis: cerebral infarction; Etiology according to TOAST system; 1-large artery atherosclerosis; 2-cardiogenic embolism; 3-small artery occlusion; 4-stroke of another determined cause; 5-stroke of an undetermined cause. 5
IMG_C_TOAST	4		K.Final diagnosis: cerebral infarction; Etiology according to TOAST system; 1-large artery atherosclerosis; 2-cardiogenic embolism; 3-small artery occlusion; 4-stroke of another determined cause; 5-stroke of an undetermined cause. 4
IMG_C_TOAST	3		K.Final diagnosis: cerebral infarction; Etiology according to TOAST system; 1-large artery atherosclerosis; 2-cardiogenic embolism; 3-small artery occlusion; 4-stroke of another determined cause; 5-stroke of an undetermined cause. 3
IMG_C_TOAST	2		K.Final diagnosis: cerebral infarction; Etiology according to TOAST system; 1-large artery atherosclerosis; 2-cardiogenic embolism; 3-small artery occlusion; 4-stroke of another determined cause; 5-stroke of an undetermined cause. 2
whtr_g*IMG_C_TOAST	4	5	1=Q1(<0.47);2=Q2(0.47-<0.52);3=Q3(0.52-<0.57);4=Q4(>=0.57) 4 * K.Final diagnosis: cerebral infarction; Etiology according to TOAST system; 1-large artery atherosclerosis; 2-cardiogenic embolism; 3-small artery occlusion; 4-stroke of another determined caus
whtr_g*IMG_C_TOAST	4	4	1=Q1(<0.47);2=Q2(0.47-<0.52);3=Q3(0.52-<0.57);4=Q4(>=0.57) 4 * K.Final diagnosis: cerebral infarction; Etiology according to TOAST system; 1-large artery atherosclerosis; 2-cardiogenic embolism; 3-small artery occlusion; 4-stroke of another determined caus
whtr_g*IMG_C_TOAST	4	3	1=Q1(<0.47);2=Q2(0.47-<0.52);3=Q3(0.52-<0.57);4=Q4(>=0.57) 4 * K.Final diagnosis: cerebral infarction; Etiology according to TOAST system; 1-large artery atherosclerosis; 2-cardiogenic embolism; 3-small artery occlusion; 4-stroke of another determined caus
whtr_g*IMG_C_TOAST	4	2	1=Q1(<0.47);2=Q2(0.47-<0.52);3=Q3(0.52-<0.57);4=Q4(>=0.57) 4 * K.Final diagnosis: cerebral infarction; Etiology according to TOAST system; 1-large artery atherosclerosis; 2-cardiogenic embolism; 3-small artery occlusion; 4-stroke of another determined caus
whtr_g*IMG_C_TOAST	3	5	1=Q1(<0.47);2=Q2(0.47-<0.52);3=Q3(0.52-<0.57);4=Q4(>=0.57) 3 * K.Final diagnosis: cerebral infarction; Etiology according to TOAST system; 1-large artery atherosclerosis; 2-cardiogenic embolism; 3-small artery occlusion; 4-stroke of another determined caus
whtr_g*IMG_C_TOAST	3	4	1=Q1(<0.47);2=Q2(0.47-<0.52);3=Q3(0.52-<0.57);4=Q4(>=0.57) 3 * K.Final diagnosis: cerebral infarction; Etiology according to TOAST system; 1-large artery atherosclerosis; 2-cardiogenic embolism; 3-small artery occlusion; 4-stroke of another determined caus
whtr_g*IMG_C_TOAST	3	3	1=Q1(<0.47);2=Q2(0.47-<0.52);3=Q3(0.52-<0.57);4=Q4(>=0.57) 3 * K.Final diagnosis: cerebral infarction; Etiology according to TOAST system; 1-large artery atherosclerosis; 2-cardiogenic embolism; 3-small artery occlusion; 4-stroke of another determined caus
whtr_g*IMG_C_TOAST	3	2	1=Q1(<0.47);2=Q2(0.47-<0.52);3=Q3(0.52-<0.57);4=Q4(>=0.57) 3 * K.Final diagnosis: cerebral infarction; Etiology according to TOAST system; 1-large artery atherosclerosis; 2-cardiogenic embolism; 3-small artery occlusion; 4-stroke of another determined caus
whtr_g*IMG_C_TOAST	1	5	1=Q1(<0.47);2=Q2(0.47-<0.52);3=Q3(0.52-<0.57);4=Q4(>=0.57) 1 * K.Final diagnosis: cerebral infarction; Etiology according to TOAST system; 1-large artery atherosclerosis; 2-cardiogenic embolism; 3-small artery occlusion; 4-stroke of another determined caus
whtr_g*IMG_C_TOAST	1	4	1=Q1(<0.47);2=Q2(0.47-<0.52);3=Q3(0.52-<0.57);4=Q4(>=0.57) 1 * K.Final diagnosis: cerebral infarction; Etiology according to TOAST system; 1-large artery atherosclerosis; 2-cardiogenic embolism; 3-small artery occlusion; 4-stroke of another determined caus
whtr_g*IMG_C_TOAST	1	3	1=Q1(<0.47);2=Q2(0.47-<0.52);3=Q3(0.52-<0.57);4=Q4(>=0.57) 1 * K.Final diagnosis: cerebral infarction; Etiology according to TOAST system; 1-large artery atherosclerosis; 2-cardiogenic embolism; 3-small artery occlusion; 4-stroke of another determined caus
whtr_g*IMG_C_TOAST	1	2	1=Q1(<0.47);2=Q2(0.47-<0.52);3=Q3(0.52-<0.57);4=Q4(>=0.57) 1 * K.Final diagnosis: cerebral infarction; Etiology according to TOAST system; 1-large artery atherosclerosis; 2-cardiogenic embolism; 3-small artery occlusion; 4-stroke of another determined caus
AGE			A.Basic Information: Age (years old);
GENDER	2		A.Basic Information: Gender; 1-male; 2-female; 2
ETHNIC	2		B.Demography: Race: 1-Han; 99-others; 2
H_DIAB01	1		D.History: Diabetes; 0-No; 1-Yes; 1
H_AF01	1		D.History: Heart disease category: Atrial fibrillation(Including medical history and hospitalization diagnosis); 0-No; 1-Yes; 1
H_HYPT01	1		D.History: Hypertension; 0-No; 1-Yes; 1

data1: whtr_g with y1_comb: interaction model

PHREG 过程

最大似然估计分析										
参数			自由度	参数估计	标准误差	卡方	Pr > 卡方	危险率	95% 危险率置信限	
H_LIPID01	1		1	-0.14398	0.18482	0.6069	0.4360	0.866	0.603	1.244
AI	1		1	-0.31353	0.38488	0.6636	0.4153	0.731	0.344	1.554
H_DRINK_H01	1		1	0.16887	0.14416	1.3722	0.2414	1.184	0.893	1.571
H_SMK_C01	1		1	-0.18774	0.11802	2.5306	0.1117	0.829	0.658	1.045
IT	1		1	0.01285	0.14032	0.0084	0.9270	1.013	0.769	1.334
ET	1		1	0.56831	0.42147	1.8182	0.1775	1.765	0.773	4.032
A_NIHSS			1	0.02970	0.00957	9.6247	0.0019	1.030	1.011	1.050

data1: whtr_g with y1_comb: interaction model

PHREG 过程

最大似然估计分析			
参数			标签
H_LIPID01	1		D.History: Lipid metabolism disorders; 0-No; 1-Yes; 1
AI	1		history:Myocardial infarction; 0=NO; 1=YES; 1
H_DRINK_H01	1		D.History: Heavy Drinking(Alcohol consumption>=20g/day); 0-No,1-Yes; 1
H_SMK_C01	1		D.History: Current Smoking; 0-No,1-Yes; 1
IT	1		intravenous thrombolysis, 1=YES,0=NO 1
ET	1		动脉溶栓或机械取栓, 1=YES,0=NO 1
A_NIHSS			F.Admitting NIHSS: Total score;

data1: whtr with y1_stroke: crude model

PHREG 过程

模型信息		
数据集	WORK.DATA1	
因变量	y1_stroke_dd	N12.Follow-up events at 12 months: Days from onset to recurrence;(day);
删失变量	y1_stroke	N12.Follow-up events at 12 months: Recurrence of stroke: 0-No; 1-Yes;
删失值	0	
结值处理	BRESLOW	

读取的观测数	4805
使用的观测数	4805

事件和删失值个数汇总			
合计	事件	删失	删失百分比
4805	489	4316	89.82

收敛状态
满足收敛准则 (GCONV=1E-8)。

模型拟合统计量		
准则	无协变量	带协变量
-2 LOG L	8222.067	8219.037
AIC	8222.067	8221.037
SBC	8222.067	8225.229

检验全局原假设: BETA=0			
检验	卡方	自由度	Pr > 卡方
似然比	3.0296	1	0.0818
评分	3.0144	1	0.0825
Wald	3.0244	1	0.0820

最大似然估计分析								
参数	自由度	参数估计	标准误差	卡方	Pr > 卡方	危险率	95% 危险率置信限	
whtr	1	0.92733	0.53323	3.0244	0.0820	2.528	0.889	7.188

data1: whtr with y1_stroke: adjusted model

PHREG 过程

模型信息		
数据集	WORK.DATA1	
因变量	y1_stroke_dd	N12.Follow-up events at 12 months: Days from onset to recurrence;(day);
删失变量	y1_stroke	N12.Follow-up events at 12 months: Recurrence of stroke: 0-No; 1-Yes;
删失值	0	
结值处理	BRESLOW	

读取的观测数	4805
使用的观测数	4805

分类水平信息		
分类	值	设计变量
GENDER	2	1
	1	0
ETHNIC	2	1
	1	0
H_DIAB01	1	1
	0	0
H_AF01	1	1
	0	0
H_HYPT01	1	1
	0	0
H_LIPID01	1	1
	0	0
AI	1	1
	0	0
H_DRINK_H01	1	1
	0	0
H_SMK_C01	1	1
	0	0
IT	1	1
	0	0
ET	1	1
	0	0

事件和删失值个数汇总			
合计	事件	删失	删失百分比
4805	489	4316	89.82

收敛状态
满足收敛准则 (GCONV=1E-8)。

data1: whtr with y1_stroke: adjusted model

PHREG 过程

模型拟合统计量		
准则	无<br>协变量	带<br>协变量
-2 LOG L	8222.067	8175.779
AIC	8222.067	8205.779
SBC	8222.067	8268.665

检验全局原假设: BETA=0			
检验	卡方	自由度	Pr > 卡方
似然比	46.2873	15	<.0001
评分	49.3076	15	<.0001
Wald	48.7343	15	<.0001

3 型检验			
效应	自由度	Wald 卡方	Pr > 卡方
whtr	1	4.7695	0.0290
AGE	1	2.4135	0.1203
GENDER	1	2.1295	0.1445
ETHNIC	1	1.6928	0.1932
H_DIAB01	1	12.0935	0.0005
H_AF01	1	1.1084	0.2924
H_HYPT01	1	0.0179	0.8936
H_LIPID01	1	0.2116	0.6455
AI	1	1.1018	0.2939
H_DRINK_H01	1	2.7097	0.0997
H_SMK_C01	1	3.1081	0.0779
IT	1	0.0663	0.7968
ET	1	2.2078	0.1373
A_NIHSS	1	10.3909	0.0013
IMG_C_TOAST	1	2.9644	0.0851

data1: whtr with y1_stroke: adjusted model

PHREG 过程

最大似然估计分析									
参数		自由度	参数估计	标准误差	卡方	Pr > 卡方	危险率	95% 危险率置信限	
whtr		1	1.17716	0.53901	4.7695	0.0290	3.245	1.128	9.333
AGE		1	0.00665	0.00428	2.4135	0.1203	1.007	0.998	1.015
GENDER	2	1	-0.15606	0.10694	2.1295	0.1445	0.856	0.694	1.055
ETHNIC	2	1	-0.32284	0.24813	1.6928	0.1932	0.724	0.445	1.178
H_DIAB01	1	1	0.35454	0.10195	12.0935	0.0005	1.426	1.167	1.741
H_AF01	1	1	0.16727	0.15888	1.1084	0.2924	1.182	0.866	1.614
H_HYPT01	1	1	-0.01298	0.09705	0.0179	0.8936	0.987	0.816	1.194
H_LIPID01	1	1	-0.08499	0.18475	0.2116	0.6455	0.919	0.639	1.319
AI	1	1	-0.43527	0.41467	1.1018	0.2939	0.647	0.287	1.459
H_DRINK_H01	1	1	0.23957	0.14554	2.7097	0.0997	1.271	0.955	1.690
H_SMK_C01	1	1	-0.21362	0.12117	3.1081	0.0779	0.808	0.637	1.024
IT	1	1	0.03697	0.14359	0.0663	0.7968	1.038	0.783	1.375
ET	1	1	0.62724	0.42214	2.2078	0.1373	1.872	0.819	4.283
A_NIHSS		1	0.03194	0.00991	10.3909	0.0013	1.032	1.013	1.053
IMG_C_TOAST		1	-0.04668	0.02711	2.9644	0.0851	0.954	0.905	1.006

最大似然估计分析		
参数		标签
whtr		
AGE		A.Basic Information: Age (years old);
GENDER	2	A.Basic Information: Gender; 1-male; 2-female; 2
ETHNIC	2	B.Demography: Race: 1-Han; 99-others; 2
H_DIAB01	1	D.History: Diabetes; 0-No; 1-Yes; 1
H_AF01	1	D.History: Heart disease category: Atrial fibrillation(Including medical history and hospitalization diagnosis); 0-No; 1-Yes; 1
H_HYPT01	1	D.History: Hypertension; 0-No; 1-Yes; 1
H_LIPID01	1	D.History: Lipid metabolism disorders; 0-No; 1-Yes; 1
AI	1	history:Myocardial infarction; 0=NO; 1=YES; 1
H_DRINK_H01	1	D.History: Heavy Drinking(Alcohol consumption>=20g/day); 0-No,1-Yes; 1
H_SMK_C01	1	D.History: Current Smoking; 0-No,1-Yes; 1
IT	1	intravenous thrombolysis, 1=YES,0=NO 1
ET	1	动脉溶栓或机械取栓, 1=YES,0=NO 1
A_NIHSS		F.Admitting NIHSS: Total score;
IMG_C_TOAST		K.Final diagnosis: cerebral infarction; Etiology according to TOAST system; 1-large artery atherosclerosis; 2-cardiogenic embolism; 3-small artery occlusion; 4-stroke of another determined cause; 5-stroke of an undetermined cause.

data1: whtr with y1_stroke: interaction model

PHREG 过程

模型信息		
数据集	WORK.DATA1	
因变量	y1_stroke_dd	N12.Follow-up events at 12 months: Days from onset to recurrence;(day);
删失变量	y1_stroke	N12.Follow-up events at 12 months: Recurrence of stroke: 0-No; 1-Yes;
删失值	0	
结值处理	BRESLOW	

读取的观测数	4805
使用的观测数	4805

分类水平信息					
分类	值	设计变量			
GENDER	2	1			
	1	0			
IMG_C_TOAST	5	1	0	0	0
	4	0	1	0	0
	3	0	0	1	0
	2	0	0	0	1
	1	0	0	0	0
ETHNIC	2	1			
	1	0			
H_DIAB01	1	1			
	0	0			
H_AF01	1	1			
	0	0			
H_HYPT01	1	1			
	0	0			
H_LIPID01	1	1			
	0	0			
AI	1	1			
	0	0			
H_DRINK_H01	1	1			
	0	0			
H_SMK_C01	1	1			
	0	0			
IT	1	1			
	0	0			
ET	1	1			
	0	0			

data1: whtr with y1_stroke: interaction model

PHREG 过程

事件和删失值个数汇总			
合计	事件	删失	删失<br>百分比
4805	489	4316	89.82

收敛状态
满足收敛准则 (GCONV=1E-8)。

模型拟合统计量		
准则	无<br>协变量	带<br>协变量
-2 LOG L	8222.067	8156.496
AIC	8222.067	8200.496
SBC	8222.067	8292.728

检验全局原假设: BETA=0			
检验	卡方	自由度	Pr > 卡方
似然比	65.5704	22	<.0001
评分	72.2396	22	<.0001
Wald	71.7138	22	<.0001

联合检验			
效应	自由度	Wald 卡方	Pr > 卡方
whtr	1	10.2255	0.0014
IMG_C_TOAST	4	3.3754	0.4971
whtr*IMG_C_TOAST	4	5.5434	0.2359
AGE	1	2.3449	0.1257
GENDER	1	2.7549	0.0970
ETHNIC	1	1.5470	0.2136
H_DIAB01	1	12.0094	0.0005
H_AF01	1	6.9159	0.0085
H_HYPT01	1	0.0076	0.9307
H_LIPID01	1	0.2357	0.6273
AI	1	1.0349	0.3090
H_DRINK_H01	1	2.5966	0.1071
H_SMK_C01	1	3.1256	0.0771
IT	1	0.0820	0.7746
ET	1	2.5007	0.1138
A_NIHSS	1	7.5945	0.0059

Note: Under full-rank parameterizations, Type 3 effect tests are replaced by joint tests. The joint test for an effect is a test that all of the parameters associated with that effect are zero. Such joint tests might not be equivalent to Type 3 effect tests under GLM parameterization.

data1: whtr with y1_stroke: interaction model

PHREG 过程

最大似然估计分析									
参数		自由度	参数估计	标准误差	卡方	Pr > 卡方	危险率	95% 危险率置信限	
whtr		1	2.70949	0.84731	10.2255	0.0014	.	.	.
IMG_C_TOAST	5	1	1.13751	0.62877	3.2729	0.0704	.	.	.
IMG_C_TOAST	4	1	0.83261	2.40008	0.1203	0.7287	.	.	.
IMG_C_TOAST	3	1	0.56756	0.82277	0.4758	0.4903	.	.	.
IMG_C_TOAST	2	1	0.27922	1.21685	0.0527	0.8185	.	.	.
whtr*IMG_C_TOAST	5	1	-2.73875	1.17701	5.4143	0.0200	.	.	.
whtr*IMG_C_TOAST	4	1	-1.28712	4.62266	0.0775	0.7807	.	.	.
whtr*IMG_C_TOAST	3	1	-1.77064	1.53312	1.3338	0.2481	.	.	.
whtr*IMG_C_TOAST	2	1	-2.02754	2.32809	0.7585	0.3838	.	.	.
AGE		1	0.00654	0.00427	2.3449	0.1257	1.007	0.998	1.015
GENDER	2	1	-0.17841	0.10749	2.7549	0.0970	0.837	0.678	1.033
ETHNIC	2	1	-0.30852	0.24804	1.5470	0.2136	0.735	0.452	1.194
H_DIAB01	1	1	0.35382	0.10210	12.0094	0.0005	1.424	1.166	1.740
H_AF01	1	1	0.51950	0.19754	6.9159	0.0085	1.681	1.141	2.476
H_HYPT01	1	1	-0.00844	0.09705	0.0076	0.9307	0.992	0.820	1.199
H_LIPID01	1	1	-0.08980	0.18498	0.2357	0.6273	0.914	0.636	1.314
AI	1	1	-0.42199	0.41481	1.0349	0.3090	0.656	0.291	1.478
H_DRINK_H01	1	1	0.23482	0.14573	2.5966	0.1071	1.265	0.950	1.683
H_SMK_C01	1	1	-0.21438	0.12126	3.1256	0.0771	0.807	0.636	1.024
IT	1	1	0.04121	0.14393	0.0820	0.7746	1.042	0.786	1.382
ET	1	1	0.66819	0.42254	2.5007	0.1138	1.951	0.852	4.465
A_NIHSS		1	0.02770	0.01005	7.5945	0.0059	1.028	1.008	1.049

data1: whtr with y1_stroke: interaction model

PHREG 过程

最大似然估计分析		
参数		标签
whtr		
IMG_C_TOAST	5	K.Final diagnosis: cerebral infarction; Etiology according to TOAST system; 1-large artery atherosclerosis; 2-cardiogenic embolism; 3-small artery occlusion; 4-stroke of another determined cause; 5-stroke of an undetermined cause. 5
IMG_C_TOAST	4	K.Final diagnosis: cerebral infarction; Etiology according to TOAST system; 1-large artery atherosclerosis; 2-cardiogenic embolism; 3-small artery occlusion; 4-stroke of another determined cause; 5-stroke of an undetermined cause. 4
IMG_C_TOAST	3	K.Final diagnosis: cerebral infarction; Etiology according to TOAST system; 1-large artery atherosclerosis; 2-cardiogenic embolism; 3-small artery occlusion; 4-stroke of another determined cause; 5-stroke of an undetermined cause. 3
IMG_C_TOAST	2	K.Final diagnosis: cerebral infarction; Etiology according to TOAST system; 1-large artery atherosclerosis; 2-cardiogenic embolism; 3-small artery occlusion; 4-stroke of another determined cause; 5-stroke of an undetermined cause. 2
whtr*IMG_C_TOAST	5	K.Final diagnosis: cerebral infarction; Etiology according to TOAST system; 1-large artery atherosclerosis; 2-cardiogenic embolism; 3-small artery occlusion; 4-stroke of another determined cause; 5-stroke of an undetermined cause. 5 * whtr
whtr*IMG_C_TOAST	4	K.Final diagnosis: cerebral infarction; Etiology according to TOAST system; 1-large artery atherosclerosis; 2-cardiogenic embolism; 3-small artery occlusion; 4-stroke of another determined cause; 5-stroke of an undetermined cause. 4 * whtr
whtr*IMG_C_TOAST	3	K.Final diagnosis: cerebral infarction; Etiology according to TOAST system; 1-large artery atherosclerosis; 2-cardiogenic embolism; 3-small artery occlusion; 4-stroke of another determined cause; 5-stroke of an undetermined cause. 3 * whtr
whtr*IMG_C_TOAST	2	K.Final diagnosis: cerebral infarction; Etiology according to TOAST system; 1-large artery atherosclerosis; 2-cardiogenic embolism; 3-small artery occlusion; 4-stroke of another determined cause; 5-stroke of an undetermined cause. 2 * whtr
AGE		A.Basic Information: Age (years old);
GENDER	2	A.Basic Information: Gender; 1-male; 2-female; 2
ETHNIC	2	B.Demography: Race: 1-Han; 99-others; 2
H_DIAB01	1	D.History: Diabetes; 0-No; 1-Yes; 1
H_AF01	1	D.History: Heart disease category: Atrial fibrillation(Including medical history and hospitalization diagnosis); 0-No; 1-Yes; 1
H_HYPT01	1	D.History: Hypertension; 0-No; 1-Yes; 1
H_LIPID01	1	D.History: Lipid metabolism disorders; 0-No; 1-Yes; 1
AI	1	history:Myocardial infarction; 0=NO; 1=YES; 1
H_DRINK_H01	1	D.History: Heavy Drinking(Alcohol consumption>=20g/day); 0-No,1-Yes; 1
H_SMK_C01	1	D.History: Current Smoking; 0-No,1-Yes; 1
IT	1	intravenous thrombolysis, 1=YES,0=NO 1
ET	1	动脉溶栓或机械取栓, 1=YES,0=NO 1
A_NIHSS		F.Admitting NIHSS: Total score;

data1: whtr_g with y1_stroke: Descriptive results

FREQ 过程

频数<br>行百分比

whtr_g-y1_stroke表

whtr_g(1=Q1(<0.47);2=Q2(0.47-<0.52);3=Q3(0.52-<0.57);4=Q4(>=0.57))	y1_stroke(N12.Follow-up events at 12 months: Recurrence of stroke: 0-No; 1-Yes;) 0	1	合计
1	1058<br>90.50	111<br>9.50	1169
2	1171<br>88.98	145<br>11.02	1316
3	1089<br>91.51	101<br>8.49	1190
4	998<br>88.32	132<br>11.68	1130
合计	4316	489	4805

表“y1_stroke-whtr_g”的统计量

统计量	自由度	值	概率
卡方	3	8.1272	0.0435
似然比卡方检验	3	8.2010	0.0420
Mantel-Haenszel 卡方	1	0.9021	0.3422
Phi 系数		0.0411	
列联系数		0.0411	
Cramer V		0.0411	

样本大小 = 4805

data1: whtr_g with y1_stroke: crude model

PHREG 过程

模型信息		
数据集	WORK.DATA1	
因变量	y1_stroke_dd	N12.Follow-up events at 12 months: Days from onset to recurrence;(day);
删失变量	y1_stroke	N12.Follow-up events at 12 months: Recurrence of stroke: 0-No; 1-Yes;
删失值	0	
结值处理	BRESLOW	

读取的观测数	4805
使用的观测数	4805

分类水平信息				
分类	值	设计变量		
whtr_g	4	1	0	0
	3	0	1	0
	2	0	0	0
	1	0	0	1

事件和删失值个数汇总			
合计	事件	删失	删失百分比
4805	489	4316	89.82

收敛状态
满足收敛准则 (GCONV=1E-8)。

模型拟合统计量		
准则	无协变量	带协变量
-2 LOG L	8222.067	8213.802
AIC	8222.067	8219.802
SBC	8222.067	8232.379

检验全局原假设: BETA=0			
检验	卡方	自由度	Pr > 卡方
似然比	8.2645	3	0.0408
评分	8.1674	3	0.0427
Wald	8.1128	3	0.0437

3 型检验			
效应	自由度	Wald 卡方	Pr > 卡方
whtr_g	3	8.1128	0.0437

data1: whtr_g with y1_stroke: crude model

PHREG 过程

最大似然估计分析

参数		自由度	参数估计	标准误差	卡方	Pr > 卡方	危险率	95% 危险率置信限	
whtr_g	4	1	0.06287	0.12030	0.2731	0.6012	1.065	0.841	1.348
whtr_g	3	1	-0.27800	0.12961	4.6006	0.0320	0.757	0.587	0.976
whtr_g	1	1	-0.15012	0.12612	1.4168	0.2339	0.861	0.672	1.102

最大似然估计分析

参数		标签
whtr_g	4	1=Q1(<0.47);2=Q2(0.47-<0.52);3=Q3(0.52-<0.57);4=Q4(>=0.57) 4
whtr_g	3	1=Q1(<0.47);2=Q2(0.47-<0.52);3=Q3(0.52-<0.57);4=Q4(>=0.57) 3
whtr_g	1	1=Q1(<0.47);2=Q2(0.47-<0.52);3=Q3(0.52-<0.57);4=Q4(>=0.57) 1

data1: whtr_g with y1_stroke: adjusted model

PHREG 过程

模型信息		
数据集	WORK.DATA1	
因变量	y1_stroke_dd	N12.Follow-up events at 12 months: Days from onset to recurrence;(day);
删失变量	y1_stroke	N12.Follow-up events at 12 months: Recurrence of stroke: 0-No; 1-Yes;
删失值	0	
结值处理	BRESLOW	

读取的观测数<br>使用的观测数	4805<br>4805

分类水平信息				
分类	值	设计变量		
whtr_g	4	1	0	0
	3	0	1	0
	2	0	0	0
	1	0	0	1
GENDER	2	1		
	1	0		
ETHNIC	2	1		
	1	0		
H_DIAB01	1	1		
	0	0		
H_AF01	1	1		
	0	0		
H_HYPT01	1	1		
	0	0		
H_LIPID01	1	1		
	0	0		
AI	1	1		
	0	0		
H_DRINK_H01	1	1		
	0	0		
H_SMK_C01	1	1		
	0	0		
IT	1	1		
	0	0		
ET	1	1		
	0	0		

data1: whtr_g with y1_stroke: adjusted model

PHREG 过程

事件和删失值个数汇总			
合计	事件	删失	删失百分比
4805	489	4316	89.82

收敛状态
满足收敛准则 (GCONV=1E-8)。

模型拟合统计量		
准则	无协变量	带协变量
-2 LOG L	8222.067	8171.096
AIC	8222.067	8205.096
SBC	8222.067	8276.366

检验全局原假设: BETA=0			
检验	卡方	自由度	Pr > 卡方
似然比	50.9709	17	<.0001
评分	53.9768	17	<.0001
Wald	53.2847	17	<.0001

3 型检验			
效应	自由度	Wald 卡方	Pr > 卡方
whtr_g	3	9.3934	0.0245
AGE	1	2.0760	0.1496
GENDER	1	1.9022	0.1678
ETHNIC	1	1.6261	0.2022
H_DIAB01	1	12.0078	0.0005
H_AF01	1	1.2600	0.2616
H_HYPT01	1	0.0130	0.9091
H_LIPID01	1	0.1868	0.6656
AI	1	0.9985	0.3177
H_DRINK_H01	1	2.6330	0.1047
H_SMK_C01	1	3.0982	0.0784
IT	1	0.0503	0.8225
ET	1	2.0298	0.1542
A_NIHSS	1	10.6937	0.0011
IMG_C_TOAST	1	2.9724	0.0847

data1: whtr_g with y1_stroke: adjusted model

PHREG 过程

最大似然估计分析

参数		自由度	参数估计	标准误差	卡方	Pr > 卡方	危险率	95% 危险率置信限	
whtr_g	4	1	0.09548	0.12217	0.6108	0.4345	1.100	0.866	1.398
whtr_g	3	1	-0.26207	0.13010	4.0580	0.0440	0.769	0.596	0.993
whtr_g	1	1	-0.18496	0.12659	2.1348	0.1440	0.831	0.649	1.065
AGE		1	0.00617	0.00428	2.0760	0.1496	1.006	0.998	1.015
GENDER	2	1	-0.14760	0.10702	1.9022	0.1678	0.863	0.700	1.064
ETHNIC	2	1	-0.31638	0.24810	1.6261	0.2022	0.729	0.448	1.185
H_DIAB01	1	1	0.35333	0.10197	12.0078	0.0005	1.424	1.166	1.739
H_AF01	1	1	0.17853	0.15904	1.2600	0.2616	1.195	0.875	1.633
H_HYPT01	1	1	-0.01109	0.09710	0.0130	0.9091	0.989	0.818	1.196
H_LIPID01	1	1	-0.07989	0.18483	0.1868	0.6656	0.923	0.643	1.326
AI	1	1	-0.41442	0.41473	0.9985	0.3177	0.661	0.293	1.489
H_DRINK_H01	1	1	0.23677	0.14591	2.6330	0.1047	1.267	0.952	1.687
H_SMK_C01	1	1	-0.21377	0.12145	3.0982	0.0784	0.808	0.636	1.025
IT	1	1	0.03226	0.14382	0.0503	0.8225	1.033	0.779	1.369
ET	1	1	0.60120	0.42198	2.0298	0.1542	1.824	0.798	4.171
A_NIHSS		1	0.03231	0.00988	10.6937	0.0011	1.033	1.013	1.053
IMG_C_TOAST		1	-0.04672	0.02710	2.9724	0.0847	0.954	0.905	1.006

最大似然估计分析

参数		标签
whtr_g	4	1=Q1(<0.47);2=Q2(0.47-<0.52);3=Q3(0.52-<0.57);4=Q4(>=0.57) 4
whtr_g	3	1=Q1(<0.47);2=Q2(0.47-<0.52);3=Q3(0.52-<0.57);4=Q4(>=0.57) 3
whtr_g	1	1=Q1(<0.47);2=Q2(0.47-<0.52);3=Q3(0.52-<0.57);4=Q4(>=0.57) 1
AGE		A.Basic Information: Age (years old);
GENDER	2	A.Basic Information: Gender; 1-male; 2-female; 2
ETHNIC	2	B.Demography: Race: 1-Han; 99-others; 2
H_DIAB01	1	D.History: Diabetes; 0-No; 1-Yes; 1
H_AF01	1	D.History: Heart disease category: Atrial fibrillation(Including medical history and hospitalization diagnosis); 0-No; 1-Yes; 1
H_HYPT01	1	D.History: Hypertension; 0-No; 1-Yes; 1
H_LIPID01	1	D.History: Lipid metabolism disorders; 0-No; 1-Yes; 1
AI	1	history:Myocardial infarction; 0=NO; 1=YES; 1
H_DRINK_H01	1	D.History: Heavy Drinking(Alcohol consumption>=20g/day); 0-No,1-Yes; 1
H_SMK_C01	1	D.History: Current Smoking; 0-No,1-Yes; 1
IT	1	intravenous thrombolysis, 1=YES,0=NO 1
ET	1	动脉溶栓或机械取栓, 1=YES,0=NO 1
A_NIHSS		F.Admitting NIHSS: Total score;
IMG_C_TOAST		K.Final diagnosis: cerebral infarction; Etiology according to TOAST system; 1-large artery atherosclerosis; 2-cardiogenic embolism; 3-small artery occlusion; 4-stroke of another determined cause; 5-stroke of an undetermined cause.

data1: whtr_g with y1_stroke: interaction model

PHREG 过程

模型信息		
数据集	WORK.DATA1	
因变量	y1_stroke_dd	N12.Follow-up events at 12 months: Days from onset to recurrence;(day);
删失变量	y1_stroke	N12.Follow-up events at 12 months: Recurrence of stroke: 0-No; 1-Yes;
删失值	0	
结值处理	BRESLOW	

读取的观测数	4805
使用的观测数	4805

分类水平信息						
分类	值	设计变量				
whtr_g	4	1	0	0	
	3	0	1	0	
	2	0	0	0	
	1	0	0	1	
IMG_C_TOAST	5	1	0	0	0
	4	0	1	0	0
	3	0	0	1	0
	2	0	0	0	1
	1	0	0	0	0
GENDER	2	1			
	1	0			
ETHNIC	2	1			
	1	0			
H_DIAB01	1	1			
	0	0			
H_AF01	1	1			
	0	0			
H_HYPT01	1	1			
	0	0			
H_LIPID01	1	1			
	0	0			
AI	1	1			
	0	0			
H_DRINK_H01	1	1			
	0	0			
H_SMK_C01	1	1			
	0	0			
IT	1	1			
	0	0			

data1: whtr_g with y1_stroke: interaction model

PHREG 过程

分类水平信息					
分类	值	设计变量			
ET	1	1			
	0	0			

事件和删失值个数汇总			
合计	事件	删失	删失<br>百分比
4805	489	4316	89.82

收敛状态
满足收敛准则 (GCONV=1E-8)。

模型拟合统计量		
准则	无<br>协变量	带<br>协变量
-2 LOG L	8222.067	8143.634
AIC	8222.067	8207.634
SBC	8222.067	8341.789

检验全局原假设: BETA=0			
检验	卡方	自由度	Pr > 卡方
似然比	78.4331	32	<.0001
评分	82.4111	32	<.0001
Wald	78.2335	32	<.0001

联合检验			
效应	自由度	Wald 卡方	Pr > 卡方
whtr_g	3	9.2122	0.0266
IMG_C_TOAST	4	7.7525	0.1011
whtr_g*IMG_C_TOAST	12	6.1155	0.9101
AGE	1	1.8950	0.1686
GENDER	1	2.0651	0.1507
ETHNIC	1	1.6538	0.1984
H_DIAB01	1	12.1149	0.0005
H_AF01	1	6.9709	0.0083
H_HYPT01	1	0.0114	0.9149
H_LIPID01	1	0.1784	0.6727
AI	1	0.8454	0.3579
H_DRINK_H01	1	2.4364	0.1185
H_SMK_C01	1	2.9771	0.0845
IT	1	0.0888	0.7657

data1: whtr_g with y1_stroke: interaction model

PHREG 过程

联合检验			
效应	自由度	Wald 卡方	Pr > 卡方
ET	1	2.5042	0.1135
A_NIHSS	1	8.0260	0.0046

Note: Under full-rank parameterizations, Type 3 effect tests are replaced by joint tests. The joint test for an effect is a test that all of the parameters associated with that effect are zero. Such joint tests might not be equivalent to Type 3 effect tests under GLM parameterization.

data1: whtr_g with y1_stroke: interaction model

PHREG 过程

最大似然估计分析										
参数			自由度	参数估计	标准误差	卡方	Pr > 卡方	危险率	95% 危险率置信限	
whtr_g	4		1	0.08107	0.20332	0.1590	0.6901	.	.	.
whtr_g	3		1	-0.35882	0.22736	2.4906	0.1145	.	.	.
whtr_g	1		1	-0.56362	0.24386	5.3417	0.0208	.	.	.
IMG_C_TOAST	5		1	-0.47926	0.19372	6.1206	0.0134	.	.	.
IMG_C_TOAST	4		1	-11.33238	168.44563	0.0045	0.9464	.	.	.
IMG_C_TOAST	3		1	-0.44924	0.24427	3.3823	0.0659	.	.	.
IMG_C_TOAST	2		1	-0.63853	0.36682	3.0300	0.0817	.	.	.
whtr_g*IMG_C_TOAST	4	5	1	0.06239	0.27920	0.0499	0.8232	.	.	.
whtr_g*IMG_C_TOAST	4	4	1	11.30526	168.44718	0.0045	0.9465	.	.	.
whtr_g*IMG_C_TOAST	4	3	1	0.00721	0.34342	0.0004	0.9832	.	.	.
whtr_g*IMG_C_TOAST	4	2	1	-0.43466	0.57703	0.5674	0.4513	.	.	.
whtr_g*IMG_C_TOAST	3	5	1	0.17398	0.30585	0.3236	0.5695	.	.	.
whtr_g*IMG_C_TOAST	3	4	1	12.08874	168.44647	0.0052	0.9428	.	.	.
whtr_g*IMG_C_TOAST	3	3	1	0.02948	0.38379	0.0059	0.9388	.	.	.
whtr_g*IMG_C_TOAST	3	2	1	-0.12913	0.53518	0.0582	0.8093	.	.	.
whtr_g*IMG_C_TOAST	1	5	1	0.60883	0.30796	3.9084	0.0480	.	.	.
whtr_g*IMG_C_TOAST	1	4	1	12.20909	168.44649	0.0053	0.9422	.	.	.
whtr_g*IMG_C_TOAST	1	3	1	0.39750	0.39008	1.0384	0.3082	.	.	.
whtr_g*IMG_C_TOAST	1	2	1	-0.01621	0.54294	0.0009	0.9762	.	.	.
AGE			1	0.00588	0.00427	1.8950	0.1686	1.006	0.998	1.014
GENDER	2		1	-0.15456	0.10755	2.0651	0.1507	0.857	0.694	1.058
ETHNIC	2		1	-0.31932	0.24831	1.6538	0.1984	0.727	0.447	1.182
H_DIAB01	1		1	0.35563	0.10217	12.1149	0.0005	1.427	1.168	1.743
H_AF01	1		1	0.52341	0.19824	6.9709	0.0083	1.688	1.144	2.489
H_HYPT01	1		1	-0.01038	0.09719	0.0114	0.9149	0.990	0.818	1.197

data1: whtr_g with y1_stroke: interaction model

PHREG 过程

最大似然估计分析			
参数			标签
whtr_g	4		1=Q1(<0.47);2=Q2(0.47-<0.52);3=Q3(0.52-<0.57);4=Q4(>=0.57) 4
whtr_g	3		1=Q1(<0.47);2=Q2(0.47-<0.52);3=Q3(0.52-<0.57);4=Q4(>=0.57) 3
whtr_g	1		1=Q1(<0.47);2=Q2(0.47-<0.52);3=Q3(0.52-<0.57);4=Q4(>=0.57) 1
IMG_C_TOAST	5		K.Final diagnosis: cerebral infarction; Etiology according to TOAST system; 1-large artery atherosclerosis; 2-cardiogenic embolism; 3-small artery occlusion; 4-stroke of another determined cause; 5-stroke of an undetermined cause. 5
IMG_C_TOAST	4		K.Final diagnosis: cerebral infarction; Etiology according to TOAST system; 1-large artery atherosclerosis; 2-cardiogenic embolism; 3-small artery occlusion; 4-stroke of another determined cause; 5-stroke of an undetermined cause. 4
IMG_C_TOAST	3		K.Final diagnosis: cerebral infarction; Etiology according to TOAST system; 1-large artery atherosclerosis; 2-cardiogenic embolism; 3-small artery occlusion; 4-stroke of another determined cause; 5-stroke of an undetermined cause. 3
IMG_C_TOAST	2		K.Final diagnosis: cerebral infarction; Etiology according to TOAST system; 1-large artery atherosclerosis; 2-cardiogenic embolism; 3-small artery occlusion; 4-stroke of another determined cause; 5-stroke of an undetermined cause. 2
whtr_g*IMG_C_TOAST	4	5	1=Q1(<0.47);2=Q2(0.47-<0.52);3=Q3(0.52-<0.57);4=Q4(>=0.57) 4 * K.Final diagnosis: cerebral infarction; Etiology according to TOAST system; 1-large artery atherosclerosis; 2-cardiogenic embolism; 3-small artery occlusion; 4-stroke of another determined caus
whtr_g*IMG_C_TOAST	4	4	1=Q1(<0.47);2=Q2(0.47-<0.52);3=Q3(0.52-<0.57);4=Q4(>=0.57) 4 * K.Final diagnosis: cerebral infarction; Etiology according to TOAST system; 1-large artery atherosclerosis; 2-cardiogenic embolism; 3-small artery occlusion; 4-stroke of another determined caus
whtr_g*IMG_C_TOAST	4	3	1=Q1(<0.47);2=Q2(0.47-<0.52);3=Q3(0.52-<0.57);4=Q4(>=0.57) 4 * K.Final diagnosis: cerebral infarction; Etiology according to TOAST system; 1-large artery atherosclerosis; 2-cardiogenic embolism; 3-small artery occlusion; 4-stroke of another determined caus
whtr_g*IMG_C_TOAST	4	2	1=Q1(<0.47);2=Q2(0.47-<0.52);3=Q3(0.52-<0.57);4=Q4(>=0.57) 4 * K.Final diagnosis: cerebral infarction; Etiology according to TOAST system; 1-large artery atherosclerosis; 2-cardiogenic embolism; 3-small artery occlusion; 4-stroke of another determined caus
whtr_g*IMG_C_TOAST	3	5	1=Q1(<0.47);2=Q2(0.47-<0.52);3=Q3(0.52-<0.57);4=Q4(>=0.57) 3 * K.Final diagnosis: cerebral infarction; Etiology according to TOAST system; 1-large artery atherosclerosis; 2-cardiogenic embolism; 3-small artery occlusion; 4-stroke of another determined caus
whtr_g*IMG_C_TOAST	3	4	1=Q1(<0.47);2=Q2(0.47-<0.52);3=Q3(0.52-<0.57);4=Q4(>=0.57) 3 * K.Final diagnosis: cerebral infarction; Etiology according to TOAST system; 1-large artery atherosclerosis; 2-cardiogenic embolism; 3-small artery occlusion; 4-stroke of another determined caus
whtr_g*IMG_C_TOAST	3	3	1=Q1(<0.47);2=Q2(0.47-<0.52);3=Q3(0.52-<0.57);4=Q4(>=0.57) 3 * K.Final diagnosis: cerebral infarction; Etiology according to TOAST system; 1-large artery atherosclerosis; 2-cardiogenic embolism; 3-small artery occlusion; 4-stroke of another determined caus
whtr_g*IMG_C_TOAST	3	2	1=Q1(<0.47);2=Q2(0.47-<0.52);3=Q3(0.52-<0.57);4=Q4(>=0.57) 3 * K.Final diagnosis: cerebral infarction; Etiology according to TOAST system; 1-large artery atherosclerosis; 2-cardiogenic embolism; 3-small artery occlusion; 4-stroke of another determined caus
whtr_g*IMG_C_TOAST	1	5	1=Q1(<0.47);2=Q2(0.47-<0.52);3=Q3(0.52-<0.57);4=Q4(>=0.57) 1 * K.Final diagnosis: cerebral infarction; Etiology according to TOAST system; 1-large artery atherosclerosis; 2-cardiogenic embolism; 3-small artery occlusion; 4-stroke of another determined caus
whtr_g*IMG_C_TOAST	1	4	1=Q1(<0.47);2=Q2(0.47-<0.52);3=Q3(0.52-<0.57);4=Q4(>=0.57) 1 * K.Final diagnosis: cerebral infarction; Etiology according to TOAST system; 1-large artery atherosclerosis; 2-cardiogenic embolism; 3-small artery occlusion; 4-stroke of another determined caus
whtr_g*IMG_C_TOAST	1	3	1=Q1(<0.47);2=Q2(0.47-<0.52);3=Q3(0.52-<0.57);4=Q4(>=0.57) 1 * K.Final diagnosis: cerebral infarction; Etiology according to TOAST system; 1-large artery atherosclerosis; 2-cardiogenic embolism; 3-small artery occlusion; 4-stroke of another determined caus
whtr_g*IMG_C_TOAST	1	2	1=Q1(<0.47);2=Q2(0.47-<0.52);3=Q3(0.52-<0.57);4=Q4(>=0.57) 1 * K.Final diagnosis: cerebral infarction; Etiology according to TOAST system; 1-large artery atherosclerosis; 2-cardiogenic embolism; 3-small artery occlusion; 4-stroke of another determined caus
AGE			A.Basic Information: Age (years old);
GENDER	2		A.Basic Information: Gender; 1-male; 2-female; 2
ETHNIC	2		B.Demography: Race: 1-Han; 99-others; 2
H_DIAB01	1		D.History: Diabetes; 0-No; 1-Yes; 1
H_AF01	1		D.History: Heart disease category: Atrial fibrillation(Including medical history and hospitalization diagnosis); 0-No; 1-Yes; 1
H_HYPT01	1		D.History: Hypertension; 0-No; 1-Yes; 1

data1: whtr_g with y1_stroke: interaction model

PHREG 过程

最大似然估计分析										
参数			自由度	参数估计	标准误差	卡方	Pr > 卡方	危险率	95% 危险率置信限	
H_LIPID01	1		1	-0.07827	0.18531	0.1784	0.6727	0.925	0.643	1.330
AI	1		1	-0.38145	0.41487	0.8454	0.3579	0.683	0.303	1.540
H_DRINK_H01	1		1	0.22819	0.14619	2.4364	0.1185	1.256	0.943	1.673
H_SMK_C01	1		1	-0.20969	0.12153	2.9771	0.0845	0.811	0.639	1.029
IT	1		1	0.04286	0.14384	0.0888	0.7657	1.044	0.787	1.384
ET	1		1	0.66815	0.42222	2.5042	0.1135	1.951	0.853	4.462
A_NIHSS			1	0.02838	0.01002	8.0260	0.0046	1.029	1.009	1.049

data1: whtr_g with y1_stroke: interaction model

PHREG 过程

最大似然估计分析			
参数			标签
H_LIPID01	1		D.History: Lipid metabolism disorders; 0-No; 1-Yes; 1
AI	1		history:Myocardial infarction; 0=NO; 1=YES; 1
H_DRINK_H01	1		D.History: Heavy Drinking(Alcohol consumption>=20g/day); 0-No,1-Yes; 1
H_SMK_C01	1		D.History: Current Smoking; 0-No,1-Yes; 1
IT	1		intravenous thrombolysis, 1=YES,0=NO 1
ET	1		动脉溶栓或机械取栓, 1=YES,0=NO 1
A_NIHSS			F.Admitting NIHSS: Total score;
