# Supplementary material for: Association of body mass index and waist-to-height ratio with outcomes in ischemic stroke: results from the Third China National Stroke Registry
Source: BMC Neurol. 2023 Apr 14;23:152. doi: 10.1186/s12883-023-03165-y (PMC10103413; doi:10.1186/s12883-023-03165-y)
Supplement: Supplementary file 2 — Additional file 2. [file 12883_2023_3165_MOESM2_ESM.zip › raw data/Table-S2.pdf]

age1: BMI_g with y1_death: Descriptive results

FREQ 过程

频数<br>行百分比

BMI_g-y1_death表

BMI_g(1=<18.5;2=18.5-<23;3=23-<27.5;4=≥27.5)	y1_death(N12.Follow-up events at 12 months: Whether the patient died: 0-survival;1-death;) 0	1	合计
1	95<br>96.94	3<br>3.06	98
2	1884<br>97.87	41<br>2.13	1925
3	4376<br>98.38	72<br>1.62	4448
4	1596<br>98.88	18<br>1.12	1614
合计	7951	134	8085

表“y1_death-BMI_g”的统计量

统计量	自由度	值	概率
卡方	3	6.7728	0.0795
似然比卡方检验	3	6.6975	0.0822
Mantel-Haenszel 卡方	1	6.6744	0.0098
Phi 系数		0.0289	
列联系数		0.0289	
Cramer V		0.0289	

样本大小 = 8085

age1: BMI_g with y1_death: adjusted model

PHREG 过程

模型信息		
数据集	WORK.AGE1	
因变量	y1_death_dd	N12.Follow-up events at 12 months: Days from onset to death;(day);
删失变量	y1_death	N12.Follow-up events at 12 months: Whether the patient died: 0-survival;1-death;
删失值	0	
结值处理	BRESLOW	

读取的观测数<br>使用的观测数	8085<br>8085

分类水平信息						
分类	值	设计变量				
BMI_g	4	1	0	0	
	3	0	1	0	
	2	0	0	0	
	1	0	0	1	
ETHNIC	2	1			
	1	0			
H_DIAB01	1	1			
	0	0			
H_AF01	1	1			
	0	0			
H_HYPT01	1	1			
	0	0			
H_LIPID01	1	1			
	0	0			
AI	1	1			
	0	0			
H_DRINK_H01	1	1			
	0	0			
H_SMK_C01	1	1			
	0	0			
IT	1	1			
	0	0			
ET	1	1			
	0	0			
IMG_C_TOAST	5	1	0	0	0
	4	0	1	0	0
	3	0	0	1	0
	2	0	0	0	1
	1	0	0	0	0

age1: BMI_g with y1_death: adjusted model

PHREG 过程

事件和删失值个数汇总			
合计	事件	删失	删失百分比
8085	134	7951	98.34

收敛状态
满足收敛准则 (GCONV=1E-8)。

模型拟合统计量		
准则	无协变量	带协变量
-2 LOG L	2404.162	2279.459
AIC	2404.162	2319.459
SBC	2404.162	2377.416

检验全局原假设: BETA=0			
检验	卡方	自由度	Pr > 卡方
似然比	124.7027	20	<.0001
评分	179.1171	20	<.0001
Wald	158.6481	20	<.0001

3 型检验			
效应	自由度	Wald 卡方	Pr > 卡方
BMI_g	3	4.5032	0.2120
AGE	1	4.0554	0.0440
GENDER	1	2.1687	0.1408
ETHNIC	1	0.6318	0.4267
H_DIAB01	1	7.7154	0.0055
H_AF01	1	23.2489	<.0001
H_HYPT01	1	0.3691	0.5435
H_LIPID01	1	0.6335	0.4261
AI	1	7.8329	0.0051
H_DRINK_H01	1	2.7129	0.0995
H_SMK_C01	1	1.3951	0.2376
IT	1	7.6398	0.0057
ET	1	0.5251	0.4687
IMG_C_TOAST	4	17.4076	0.0016
A_NIHSS	1	53.5456	<.0001

age1: BMI_g with y1_death: adjusted model

PHREG 过程

最大似然估计分析									
参数		自由度	参数估计	标准误差	卡方	Pr > 卡方	危险率	95% 危险率置信限	
BMI_g	4	1	-0.51844	0.29044	3.1864	0.0743	0.595	0.337	1.052
BMI_g	3	1	-0.16951	0.20059	0.7141	0.3981	0.844	0.570	1.251
BMI_g	1	1	0.51558	0.60095	0.7361	0.3909	1.675	0.516	5.438
AGE		1	0.02649	0.01316	4.0554	0.0440	1.027	1.001	1.054
GENDER		1	-0.31657	0.21497	2.1687	0.1408	0.729	0.478	1.110
ETHNIC	2	1	0.33514	0.42162	0.6318	0.4267	1.398	0.612	3.195
H_DIAB01	1	1	0.53360	0.19210	7.7154	0.0055	1.705	1.170	2.485
H_AF01	1	1	1.67689	0.34778	23.2489	<.0001	5.349	2.705	10.575
H_HYPT01	1	1	-0.11141	0.18339	0.3691	0.5435	0.895	0.624	1.281
H_LIPID01	1	1	-0.27971	0.35143	0.6335	0.4261	0.756	0.380	1.505
AI	1	1	1.12422	0.40169	7.8329	0.0051	3.078	1.401	6.763
H_DRINK_H01	1	1	-0.48367	0.29366	2.7129	0.0995	0.617	0.347	1.096
H_SMK_C01	1	1	-0.25190	0.21327	1.3951	0.2376	0.777	0.512	1.181
IT	1	1	-0.94284	0.34111	7.6398	0.0057	0.390	0.200	0.760
ET	1	1	0.39388	0.54352	0.5251	0.4687	1.483	0.511	4.302
IMG_C_TOAST	5	1	-0.39694	0.20142	3.8837	0.0488	0.672	0.453	0.998
IMG_C_TOAST	4	1	0.29820	0.59790	0.2487	0.6180	1.347	0.417	4.350
IMG_C_TOAST	3	1	-0.84437	0.29278	8.3174	0.0039	0.430	0.242	0.763
IMG_C_TOAST	2	1	-1.91135	0.60420	10.0075	0.0016	0.148	0.045	0.483
A_NIHSS		1	0.09880	0.01350	53.5456	<.0001	1.104	1.075	1.133

age1: BMI_g with y1_death: adjusted model

PHREG 过程

最大似然估计分析

参数		标签
BMI_g	4	1=<18.5;2=18.5-<23;3=23-<27.5;4= ≥ 27.5 4
BMI_g	3	1=<18.5;2=18.5-<23;3=23-<27.5;4= ≥ 27.5 3
BMI_g	1	1=<18.5;2=18.5-<23;3=23-<27.5;4= ≥ 27.5 1
AGE		A.Basic Information: Age (years old);
GENDER		A.Basic Information: Gender; 1-male; 2-female;
ETHNIC	2	B.Demography: Race: 1-Han; 99-others; 2
H_DIAB01	1	D.History: Diabetes; 0-No; 1-Yes; 1
H_AF01	1	D.History: Heart disease category: Atrial fibrillation(Including medical history and hospitalization diagnosis); 0-No; 1-Yes; 1
H_HYPT01	1	D.History: Hypertension; 0-No; 1-Yes; 1
H_LIPID01	1	D.History: Lipid metabolism disorders; 0-No; 1-Yes; 1
AI	1	history:Myocardial infarction; 0=NO; 1=YES; 1
H_DRINK_H01	1	D.History: Heavy Drinking(Alcohol consumption>=20g/day); 0-No,1-Yes; 1
H_SMK_C01	1	D.History: Current Smoking; 0-No,1-Yes; 1
IT	1	intravenous thrombolysis, 1=YES,0=NO 1
ET	1	动脉溶栓或机械取栓, 1=YES,0=NO 1
IMG_C_TOAST	5	K.Final diagnosis: cerebral infarction; Etiology according to TOAST system; 1-large artery atherosclerosis; 2-cardiogenic embolism; 3-small artery occlusion; 4-stroke of another determined cause; 5-stroke of an undetermined cause. 5
IMG_C_TOAST	4	K.Final diagnosis: cerebral infarction; Etiology according to TOAST system; 1-large artery atherosclerosis; 2-cardiogenic embolism; 3-small artery occlusion; 4-stroke of another determined cause; 5-stroke of an undetermined cause. 4
IMG_C_TOAST	3	K.Final diagnosis: cerebral infarction; Etiology according to TOAST system; 1-large artery atherosclerosis; 2-cardiogenic embolism; 3-small artery occlusion; 4-stroke of another determined cause; 5-stroke of an undetermined cause. 3
IMG_C_TOAST	2	K.Final diagnosis: cerebral infarction; Etiology according to TOAST system; 1-large artery atherosclerosis; 2-cardiogenic embolism; 3-small artery occlusion; 4-stroke of another determined cause; 5-stroke of an undetermined cause. 2
A_NIHSS		F.Admitting NIHSS: Total score;

BMI_g with y1_death: interaction with age_group

PHREG 过程

模型信息		
数据集	WORK.DATA_OVERALL	
因变量	y1_death_dd	N12.Follow-up events at 12 months: Days from onset to death;(day);
删失变量	y1_death	N12.Follow-up events at 12 months: Whether the patient died: 0-survival;1-death;
删失值	0	
结值处理	BRESLOW	

读取的观测数	14146
使用的观测数	14146

分类水平信息						
分类	值	设计变量				
BMI_g	4	1	0	0	
	3	0	1	0	
	2	0	0	0	
	1	0	0	1	
ETHNIC	2	1			
	1	0			
H_DIAB01	1	1			
	0	0			
H_AF01	1	1			
	0	0			
H_HYPT01	1	1			
	0	0			
H_LIPID01	1	1			
	0	0			
AI	1	1			
	0	0			
H_DRINK_H01	1	1			
	0	0			
H_SMK_C01	1	1			
	0	0			
IT	1	1			
	0	0			
ET	1	1			
	0	0			
IMG_C_TOAST	5	1	0	0	0
	4	0	1	0	0
	3	0	0	1	0
	2	0	0	0	1
	1	0	0	0	0

BMI_g with y1_death: interaction with age_group

PHREG 过程

事件和删失值个数汇总			
合计	事件	删失	删失百分比
14146	486	13660	96.56

收敛状态
满足收敛准则 (GCONV=1E-8)。

模型拟合统计量		
准则	无协变量	带协变量
-2 LOG L	9255.945	8678.485
AIC	9255.945	8724.485
SBC	9255.945	8820.768

检验全局原假设: BETA=0			
检验	卡方	自由度	Pr > 卡方
似然比	577.4600	23	<.0001
评分	833.1321	23	<.0001
Wald	698.4128	23	<.0001

联合检验			
效应	自由度	Wald 卡方	Pr > 卡方
BMI_g	3	1.8060	0.6136
age_group	1	24.7221	<.0001
age_group*BMI_g	3	0.3651	0.9473
GENDER	1	1.3142	0.2516
ETHNIC	1	2.4636	0.1165
H_DIAB01	1	11.3115	0.0008
H_AF01	1	40.1273	<.0001
H_HYPT01	1	0.5508	0.4580
H_LIPID01	1	2.4115	0.1205
AI	1	4.9108	0.0267
H_DRINK_H01	1	7.9483	0.0048
H_SMK_C01	1	0.0101	0.9199
IT	1	20.1519	<.0001
ET	1	5.5470	0.0185
IMG_C_TOAST	4	34.9058	<.0001
A_NIHSS	1	233.6415	<.0001

Note: Under full-rank parameterizations, Type 3 effect tests are replaced by joint tests. The joint test for an effect is a test that all of the parameters associated with that effect are zero. Such joint tests might not be equivalent to Type 3 effect tests under GLM parameterization.

BMI_g with y1_death: interaction with age_group

PHREG 过程

最大似然估计分析

参数		自由度	参数估计	标准误差	卡方	Pr > 卡方	危险率	95% 危险率置信限	
BMI_g	4	1	-0.74790	0.59657	1.5717	0.2100	.	.	.
BMI_g	3	1	-0.22391	0.41164	0.2959	0.5865	.	.	.
BMI_g	1	1	0.35064	1.21610	0.0831	0.7731	.	.	.
age_group		1	0.91776	0.18458	24.7221	<.0001	.	.	.
age_group*BMI_g	4	1	0.19970	0.33817	0.3487	0.5548	.	.	.
age_group*BMI_g	3	1	0.05802	0.23053	0.0633	0.8013	.	.	.
age_group*BMI_g	1	1	0.13657	0.63445	0.0463	0.8296	.	.	.
GENDER		1	-0.11795	0.10289	1.3142	0.2516	0.889	0.726	1.087
ETHNIC	2	1	0.36936	0.23532	2.4636	0.1165	1.447	0.912	2.295
H_DIAB01	1	1	0.35038	0.10418	11.3115	0.0008	1.420	1.157	1.741
H_AF01	1	1	0.94786	0.14963	40.1273	<.0001	2.580	1.924	3.460
H_HYPT01	1	1	0.07255	0.09776	0.5508	0.4580	1.075	0.888	1.302
H_LIPID01	1	1	-0.32256	0.20772	2.4115	0.1205	0.724	0.482	1.088
AI	1	1	0.50480	0.22779	4.9108	0.0267	1.657	1.060	2.589
H_DRINK_H01	1	1	-0.54982	0.19502	7.9483	0.0048	0.577	0.394	0.846
H_SMK_C01	1	1	0.01239	0.12324	0.0101	0.9199	1.012	0.795	1.289
IT	1	1	-0.74154	0.16519	20.1519	<.0001	0.476	0.345	0.659
ET	1	1	0.73803	0.31336	5.5470	0.0185	2.092	1.132	3.866
IMG_C_TOAST	5	1	-0.23482	0.11145	4.4390	0.0351	0.791	0.636	0.984
IMG_C_TOAST	4	1	0.58592	0.32745	3.2017	0.0736	1.797	0.946	3.413
IMG_C_TOAST	3	1	-0.94131	0.18276	26.5290	<.0001	0.390	0.273	0.558
IMG_C_TOAST	2	1	-0.46379	0.19527	5.6412	0.0175	0.629	0.429	0.922
A_NIHSS		1	0.09573	0.00626	233.6415	<.0001	1.100	1.087	1.114

BMI_g with y1_death: interaction with age_group

PHREG 过程

最大似然估计分析		
参数		标签
BMI_g	4	1=<18.5;2=18.5-<23;3=23-<27.5;4=≥27.5 4
BMI_g	3	1=<18.5;2=18.5-<23;3=23-<27.5;4=≥27.5 3
BMI_g	1	1=<18.5;2=18.5-<23;3=23-<27.5;4=≥27.5 1
age_group		1=age<65;2=age>=65
age_group*BMI_g	4	1=<18.5;2=18.5-<23;3=23-<27.5;4=≥27.5 4 * 1=age<65;2=age>=65
age_group*BMI_g	3	1=<18.5;2=18.5-<23;3=23-<27.5;4=≥27.5 3 * 1=age<65;2=age>=65
age_group*BMI_g	1	1=<18.5;2=18.5-<23;3=23-<27.5;4=≥27.5 1 * 1=age<65;2=age>=65
GENDER		A.Basic Information: Gender; 1-male; 2-female;
ETHNIC	2	B.Demography: Race: 1-Han; 99-others; 2
H_DIAB01	1	D.History: Diabetes; 0-No; 1-Yes; 1
H_AF01	1	D.History: Heart disease category: Atrial fibrillation(Including medical history and hospitalization diagnosis); 0-No; 1-Yes; 1
H_HYPT01	1	D.History: Hypertension; 0-No; 1-Yes; 1
H_LIPID01	1	D.History: Lipid metabolism disorders; 0-No; 1-Yes; 1
AI	1	history:Myocardial infarction; 0=NO; 1=YES; 1
H_DRINK_H01	1	D.History: Heavy Drinking(Alcohol consumption>=20g/day); 0-No,1-Yes; 1
H_SMK_C01	1	D.History: Current Smoking; 0-No,1-Yes; 1
IT	1	intravenous thrombolysis, 1=YES,0=NO 1
ET	1	动脉溶栓或机械取栓, 1=YES,0=NO 1
IMG_C_TOAST	5	K.Final diagnosis: cerebral infarction; Etiology according to TOAST system; 1-large artery atherosclerosis; 2-cardiogenic embolism; 3-small artery occlusion; 4-stroke of another determined cause; 5-stroke of an undetermined cause. 5
IMG_C_TOAST	4	K.Final diagnosis: cerebral infarction; Etiology according to TOAST system; 1-large artery atherosclerosis; 2-cardiogenic embolism; 3-small artery occlusion; 4-stroke of another determined cause; 5-stroke of an undetermined cause. 4
IMG_C_TOAST	3	K.Final diagnosis: cerebral infarction; Etiology according to TOAST system; 1-large artery atherosclerosis; 2-cardiogenic embolism; 3-small artery occlusion; 4-stroke of another determined cause; 5-stroke of an undetermined cause. 3
IMG_C_TOAST	2	K.Final diagnosis: cerebral infarction; Etiology according to TOAST system; 1-large artery atherosclerosis; 2-cardiogenic embolism; 3-small artery occlusion; 4-stroke of another determined cause; 5-stroke of an undetermined cause. 2
A_NIHSS		F.Admitting NIHSS: Total score;

age1: BMI_g with y1_comb: Descriptive results

FREQ 过程

频数<br>行百分比

BMI_g-y1_comb表

BMI_g(1=<18.5;2=18.5-<23;3=23-<27.5;4= ≥ 27.5)	y1_comb(N12.Follow-up events at 12 months:Occurrence of combined vascular event(including cardiovascular death,non-fatal stroke,non-fatal myocardial infarction):0-No;1-Yes;) 0	1	合计
1	86<br>87.76	12<br>12.24	98
2	1726<br>89.66	199<br>10.34	1925
3	4035<br>90.71	413<br>9.29	4448
4	1451<br>89.90	163<br>10.10	1614
合计	7298	787	8085

表“y1_comb-BMI_g”的统计量

统计量	自由度	值	概率
卡方	3	2.7667	0.4290
似然比卡方检验	3	2.7162	0.4375
Mantel-Haenszel 卡方	1	0.3309	0.5651
Phi 系数		0.0185	
列联系数		0.0185	
Cramer V		0.0185	

样本大小 = 8085

age1: BMI_g with y1_comb: adjusted model

PHREG 过程

模型信息		
数据集	WORK.AGE1	
因变量	y1_comb_dd	N12.Follow-up events at 12 months: Days from onset to occurrence of combined vascular event;(day);
删失变量	y1_comb	N12.Follow-up events at 12 months:Occurrence of combined vascular event(including cardiovascular death,non-fatal stroke,non-fatal myocardial infarction):0-No;1-Yes;
删失值	0	
结值处理	BRESLOW	

读取的观测数	8085
使用的观测数	8085

分类水平信息					
分类	值	设计变量			
BMI_g	4	1	0	0	
	3	0	1	0	
	2	0	0	0	
	1	0	0	1	
ETHNIC	2	1			
	1	0			
H_DIAB01	1	1			
	0	0			
H_AF01	1	1			
	0	0			
H_HYPT01	1	1			
	0	0			
H_LIPID01	1	1			
	0	0			
AI	1	1			
	0	0			
H_DRINK_H01	1	1			
	0	0			
H_SMK_C01	1	1			
	0	0			
IT	1	1			
	0	0			
ET	1	1			
	0	0			
IMG_C_TOAST	5	1	0	0	0
	4	0	1	0	0
	3	0	0	1	0
	2	0	0	0	1
	1	0	0	0	0

age1: BMI_g with y1_comb: adjusted model

PHREG 过程

事件和删失值个数汇总			
合计	事件	删失	删失百分比
8085	787	7298	90.27

收敛状态
满足收敛准则 (GCONV=1E-8)。

模型拟合统计量		
准则	无协变量	带协变量
-2 LOG L	14063.857	13993.342
AIC	14063.857	14033.342
SBC	14063.857	14126.706

检验全局原假设: BETA=0			
检验	卡方	自由度	Pr > 卡方
似然比	70.5152	20	<.0001
评分	74.7952	20	<.0001
Wald	73.1628	20	<.0001

3 型检验			
效应	自由度	Wald 卡方	Pr > 卡方
BMI_g	3	2.5948	0.4584
AGE	1	7.8517	0.0051
GENDER	1	0.0142	0.9053
ETHNIC	1	0.3748	0.5404
H_DIAB01	1	2.7450	0.0976
H_AF01	1	3.2108	0.0732
H_HYPT01	1	5.0027	0.0253
H_LIPID01	1	0.2225	0.6371
AI	1	0.2200	0.6390
H_DRINK_H01	1	2.3264	0.1272
H_SMK_C01	1	2.2779	0.1312
IT	1	0.1582	0.6908
ET	1	4.7553	0.0292
IMG_C_TOAST	4	23.9830	<.0001
A_NIHSS	1	3.0929	0.0786

age1: BMI_g with y1_comb: adjusted model

PHREG 过程

最大似然估计分析									
参数		自由度	参数估计	标准误差	卡方	Pr > 卡方	危险率	95% 危险率置信限	
BMI_g	4	1	-0.00860	0.10831	0.0063	0.9367	0.991	0.802	1.226
BMI_g	3	1	-0.10746	0.08733	1.5142	0.2185	0.898	0.757	1.066
BMI_g	1	1	0.15670	0.29798	0.2765	0.5990	1.170	0.652	2.097
AGE		1	0.01444	0.00515	7.8517	0.0051	1.015	1.004	1.025
GENDER		1	-0.01070	0.08991	0.0142	0.9053	0.989	0.830	1.180
ETHNIC	2	1	-0.13276	0.21686	0.3748	0.5404	0.876	0.572	1.339
H_DIAB01	1	1	0.13838	0.08352	2.7450	0.0976	1.148	0.975	1.353
H_AF01	1	1	0.40512	0.22609	3.2108	0.0732	1.499	0.963	2.336
H_HYPT01	1	1	0.17361	0.07762	5.0027	0.0253	1.190	1.022	1.385
H_LIPID01	1	1	-0.06191	0.13122	0.2225	0.6371	0.940	0.727	1.216
AI	1	1	0.12388	0.26410	0.2200	0.6390	1.132	0.675	1.899
H_DRINK_H01	1	1	0.15390	0.10090	2.3264	0.1272	1.166	0.957	1.421
H_SMK_C01	1	1	-0.13210	0.08752	2.2779	0.1312	0.876	0.738	1.040
IT	1	1	0.04533	0.11395	0.1582	0.6908	1.046	0.837	1.308
ET	1	1	0.72419	0.33210	4.7553	0.0292	2.063	1.076	3.955
IMG_C_TOAST	5	1	-0.25864	0.08405	9.4705	0.0021	0.772	0.655	0.910
IMG_C_TOAST	4	1	-0.01122	0.28528	0.0015	0.9686	0.989	0.565	1.730
IMG_C_TOAST	3	1	-0.49101	0.10557	21.6325	<.0001	0.612	0.498	0.753
IMG_C_TOAST	2	1	-0.45395	0.25181	3.2498	0.0714	0.635	0.388	1.040
A_NIHSS		1	0.01536	0.00873	3.0929	0.0786	1.015	0.998	1.033

age1: BMI_g with y1_comb: adjusted model

PHREG 过程

最大似然估计分析		
参数		标签
BMI_g	4	1=<18.5;2=18.5-<23;3=23-<27.5;4=≥27.5 4
BMI_g	3	1=<18.5;2=18.5-<23;3=23-<27.5;4=≥27.5 3
BMI_g	1	1=<18.5;2=18.5-<23;3=23-<27.5;4=≥27.5 1
AGE		A.Basic Information: Age (years old);
GENDER		A.Basic Information: Gender; 1-male; 2-female;
ETHNIC	2	B.Demography: Race: 1-Han; 99-others; 2
H_DIAB01	1	D.History: Diabetes; 0-No; 1-Yes; 1
H_AF01	1	D.History: Heart disease category: Atrial fibrillation(Including medical history and hospitalization diagnosis); 0-No; 1-Yes; 1
H_HYPT01	1	D.History: Hypertension; 0-No; 1-Yes; 1
H_LIPID01	1	D.History: Lipid metabolism disorders; 0-No; 1-Yes; 1
AI	1	history:Myocardial infarction; 0=NO; 1=YES; 1
H_DRINK_H01	1	D.History: Heavy Drinking(Alcohol consumption>=20g/day); 0-No,1-Yes; 1
H_SMK_C01	1	D.History: Current Smoking; 0-No,1-Yes; 1
IT	1	intravenous thrombolysis, 1=YES,0=NO 1
ET	1	动脉溶栓或机械取栓, 1=YES,0=NO 1
IMG_C_TOAST	5	K.Final diagnosis: cerebral infarction; Etiology according to TOAST system; 1-large artery atherosclerosis; 2-cardiogenic embolism; 3-small artery occlusion; 4-stroke of another determined cause; 5-stroke of an undetermined cause. 5
IMG_C_TOAST	4	K.Final diagnosis: cerebral infarction; Etiology according to TOAST system; 1-large artery atherosclerosis; 2-cardiogenic embolism; 3-small artery occlusion; 4-stroke of another determined cause; 5-stroke of an undetermined cause. 4
IMG_C_TOAST	3	K.Final diagnosis: cerebral infarction; Etiology according to TOAST system; 1-large artery atherosclerosis; 2-cardiogenic embolism; 3-small artery occlusion; 4-stroke of another determined cause; 5-stroke of an undetermined cause. 3
IMG_C_TOAST	2	K.Final diagnosis: cerebral infarction; Etiology according to TOAST system; 1-large artery atherosclerosis; 2-cardiogenic embolism; 3-small artery occlusion; 4-stroke of another determined cause; 5-stroke of an undetermined cause. 2
A_NIHSS		F.Admitting NIHSS: Total score;

BMI_g with y1_comb: interaction with age_group

PHREG 过程

模型信息		
数据集	WORK.DATA_OVERALL	
因变量	y1_comb_dd	N12.Follow-up events at 12 months: Days from onset to occurrence of combined vascular event;(day);
删失变量	y1_comb	N12.Follow-up events at 12 months:Occurrence of combined vascular event(including cardiovascular death,non-fatal stroke,non-fatal myocardial infarction):0-No;1-Yes;
删失值	0	
结值处理	BRESLOW	

读取的观测数	14146
使用的观测数	14146

分类水平信息					
分类	值	设计变量			
BMI_g	4	1	0	0	
	3	0	1	0	
	2	0	0	0	
	1	0	0	1	
ETHNIC	2	1			
	1	0			
H_DIAB01	1	1			
	0	0			
H_AF01	1	1			
	0	0			
H_HYPT01	1	1			
	0	0			
H_LIPID01	1	1			
	0	0			
AI	1	1			
	0	0			
H_DRINK_H01	1	1			
	0	0			
H_SMK_C01	1	1			
	0	0			
IT	1	1			
	0	0			
ET	1	1			
	0	0			
IMG_C_TOAST	5	1	0	0	0
	4	0	1	0	0
	3	0	0	1	0
	2	0	0	0	1
	1	0	0	0	0

BMI_g with y1_comb: interaction with age_group

PHREG 过程

事件和删失值个数汇总			
合计	事件	删失	删失百分比
14146	1505	12641	89.36

收敛状态
满足收敛准则 (GCONV=1E-8)。

模型拟合统计量		
准则	无协变量	带协变量
-2 LOG L	28553.433	28391.951
AIC	28553.433	28437.951
SBC	28553.433	28560.232

检验全局原假设: BETA=0			
检验	卡方	自由度	Pr > 卡方
似然比	161.4815	23	<.0001
评分	173.8273	23	<.0001
Wald	169.7249	23	<.0001

联合检验			
效应	自由度	Wald 卡方	Pr > 卡方
BMI_g	3	3.0456	0.3847
age_group	1	0.0231	0.8791
age_group*BMI_g	3	3.3167	0.3453
GENDER	1	0.2851	0.5934
ETHNIC	1	0.4702	0.4929
H_DIAB01	1	14.6036	0.0001
H_AF01	1	16.3244	<.0001
H_HYPT01	1	3.6829	0.0550
H_LIPID01	1	0.7085	0.3999
AI	1	2.4306	0.1190
H_DRINK_H01	1	1.1465	0.2843
H_SMK_C01	1	0.7793	0.3774
IT	1	0.1869	0.6655
ET	1	5.2005	0.0226
IMG_C_TOAST	4	55.0719	<.0001
A_NIHSS	1	16.6632	<.0001

Note: Under full-rank parameterizations, Type 3 effect tests are replaced by joint tests. The joint test for an effect is a test that all of the parameters associated with that effect are zero. Such joint tests might not be equivalent to Type 3 effect tests under GLM parameterization.

BMI_g with y1_comb: interaction with age_group

PHREG 过程

最大似然估计分析									
参数		自由度	参数估计	标准误差	卡方	Pr > 卡方	危险率	95% 危险率置信限	
BMI_g	4	1	-0.19472	0.24248	0.6449	0.4220	.	.	.
BMI_g	3	1	-0.32519	0.19365	2.8200	0.0931	.	.	.
BMI_g	1	1	0.07256	0.62934	0.0133	0.9082	.	.	.
age_group		1	-0.01529	0.10052	0.0231	0.8791	.	.	.
age_group*BMI_g	4	1	0.16715	0.15799	1.1194	0.2900	.	.	.
age_group*BMI_g	3	1	0.22000	0.12242	3.2295	0.0723	.	.	.
age_group*BMI_g	1	1	0.06197	0.36127	0.0294	0.8638	.	.	.
GENDER		1	0.03244	0.06075	0.2851	0.5934	1.033	0.917	1.164
ETHNIC	2	1	-0.11014	0.16061	0.4702	0.4929	0.896	0.654	1.227
H_DIAB01	1	1	0.22540	0.05898	14.6036	0.0001	1.253	1.116	1.406
H_AF01	1	1	0.46639	0.11543	16.3244	<.0001	1.594	1.271	1.999
H_HYPT01	1	1	0.10717	0.05584	3.6829	0.0550	1.113	0.998	1.242
H_LIPID01	1	1	-0.08394	0.09973	0.7085	0.3999	0.919	0.756	1.118
AI	1	1	0.24630	0.15798	2.4306	0.1190	1.279	0.939	1.744
H_DRINK_H01	1	1	0.08822	0.08239	1.1465	0.2843	1.092	0.929	1.284
H_SMK_C01	1	1	-0.05921	0.06708	0.7793	0.3774	0.943	0.826	1.075
IT	1	1	0.03538	0.08183	0.1869	0.6655	1.036	0.882	1.216
ET	1	1	0.56976	0.24984	5.2005	0.0226	1.768	1.083	2.885
IMG_C_TOAST	5	1	-0.33469	0.06152	29.5998	<.0001	0.716	0.634	0.807
IMG_C_TOAST	4	1	-0.05769	0.22323	0.0668	0.7961	0.944	0.609	1.462
IMG_C_TOAST	3	1	-0.53304	0.07918	45.3229	<.0001	0.587	0.502	0.685
IMG_C_TOAST	2	1	-0.42326	0.13576	9.7200	0.0018	0.655	0.502	0.855
A_NIHSS		1	0.02328	0.00570	16.6632	<.0001	1.024	1.012	1.035

BMI_g with y1_comb: interaction with age_group

PHREG 过程

最大似然估计分析		
参数		标签
BMI_g	4	1=<18.5;2=18.5-<23;3=23-<27.5;4= ≥ 27.5 4
BMI_g	3	1=<18.5;2=18.5-<23;3=23-<27.5;4= ≥ 27.5 3
BMI_g	1	1=<18.5;2=18.5-<23;3=23-<27.5;4= ≥ 27.5 1
age_group		1=age<65;2=age>=65
age_group*BMI_g	4	1=<18.5;2=18.5-<23;3=23-<27.5;4= ≥ 27.5 4 * 1=age<65;2=age>=65
age_group*BMI_g	3	1=<18.5;2=18.5-<23;3=23-<27.5;4= ≥ 27.5 3 * 1=age<65;2=age>=65
age_group*BMI_g	1	1=<18.5;2=18.5-<23;3=23-<27.5;4= ≥ 27.5 1 * 1=age<65;2=age>=65
GENDER		A.Basic Information: Gender; 1-male; 2-female;
ETHNIC	2	B.Demography: Race: 1-Han; 99-others; 2
H_DIAB01	1	D.History: Diabetes; 0-No; 1-Yes; 1
H_AF01	1	D.History: Heart disease category: Atrial fibrillation(Including medical history and hospitalization diagnosis); 0-No; 1-Yes; 1
H_HYPT01	1	D.History: Hypertension; 0-No; 1-Yes; 1
H_LIPID01	1	D.History: Lipid metabolism disorders; 0-No; 1-Yes; 1
AI	1	history:Myocardial infarction; 0=NO; 1=YES; 1
H_DRINK_H01	1	D.History: Heavy Drinking(Alcohol consumption>=20g/day); 0-No,1-Yes; 1
H_SMK_C01	1	D.History: Current Smoking; 0-No,1-Yes; 1
IT	1	intravenous thrombolysis, 1=YES,0=NO 1
ET	1	动脉溶栓或机械取栓, 1=YES,0=NO 1
IMG_C_TOAST	5	K.Final diagnosis: cerebral infarction; Etiology according to TOAST system; 1-large artery atherosclerosis; 2-cardiogenic embolism; 3-small artery occlusion; 4-stroke of another determined cause; 5-stroke of an undetermined cause. 5
IMG_C_TOAST	4	K.Final diagnosis: cerebral infarction; Etiology according to TOAST system; 1-large artery atherosclerosis; 2-cardiogenic embolism; 3-small artery occlusion; 4-stroke of another determined cause; 5-stroke of an undetermined cause. 4
IMG_C_TOAST	3	K.Final diagnosis: cerebral infarction; Etiology according to TOAST system; 1-large artery atherosclerosis; 2-cardiogenic embolism; 3-small artery occlusion; 4-stroke of another determined cause; 5-stroke of an undetermined cause. 3
IMG_C_TOAST	2	K.Final diagnosis: cerebral infarction; Etiology according to TOAST system; 1-large artery atherosclerosis; 2-cardiogenic embolism; 3-small artery occlusion; 4-stroke of another determined cause; 5-stroke of an undetermined cause. 2
A_NIHSS		F.Admitting NIHSS: Total score;

age1: BMI_g with y1_stroke: Descriptive results

FREQ 过程

频数<br>行百分比

BMI_g-y1_stroke表			
	y1_stroke(N12.Follow-up events at 12 months: Recurrence of stroke: 0-No; 1-Yes;)		
BMI_g(1=<18.5;2=18.5-<23;3=23-<27.5;4=≥27.5)	0	1	合计
1	88<br>89.80	10<br>10.20	98
2	1739<br>90.34	186<br>9.66	1925
3	4054<br>91.14	394<br>8.86	4448
4	1453<br>90.02	161<br>9.98	1614
合计	7334	751	8085

表“y1_stroke-BMI_g”的统计量

统计量	自由度	值	概率
卡方	3	2.2988	0.5127
似然比卡方检验	3	2.2870	0.5150
Mantel-Haenszel 卡方	1	0.0133	0.9080
Phi 系数		0.0169	
列联系数		0.0169	
Cramer V		0.0169	

样本大小 = 8085

age1: BMI_g with y1_stroke: adjusted model

PHREG 过程

模型信息		
数据集	WORK.AGE1	
因变量	y1_stroke_dd	N12.Follow-up events at 12 months: Days from onset to recurrence;(day);
删失变量	y1_stroke	N12.Follow-up events at 12 months: Recurrence of stroke: 0-No; 1-Yes;
删失值	0	
结值处理	BRESLOW	

读取的观测数	8085
使用的观测数	8085

分类水平信息						
分类	值	设计变量				
BMI_g	4	1	0	0	
	3	0	1	0	
	2	0	0	0	
	1	0	0	1	
ETHNIC	2	1			
	1	0			
H_DIAB01	1	1			
	0	0			
H_AF01	1	1			
	0	0			
H_HYPT01	1	1			
	0	0			
H_LIPID01	1	1			
	0	0			
AI	1	1			
	0	0			
H_DRINK_H01	1	1			
	0	0			
H_SMK_C01	1	1			
	0	0			
IT	1	1			
	0	0			
ET	1	1			
	0	0			
IMG_C_TOAST	5	1	0	0	0
	4	0	1	0	0
	3	0	0	1	0
	2	0	0	0	1
	1	0	0	0	0

age1: BMI_g with y1_stroke: adjusted model

PHREG 过程

事件和删失值个数汇总			
合计	事件	删失	删失<br>百分比
8085	751	7334	90.71

收敛状态
满足收敛准则 (GCONV=1E-8)。

模型拟合统计量		
准则	无<br>协变量	带<br>协变量
-2 LOG L	13424.430	13358.312
AIC	13424.430	13398.312
SBC	13424.430	13490.740

检验全局原假设: BETA=0			
检验	卡方	自由度	Pr > 卡方
似然比	66.1180	20	<.0001
评分	70.9122	20	<.0001
Wald	69.1271	20	<.0001

3 型检验			
效应	自由度	Wald 卡方	Pr > 卡方
BMI_g	3	2.2914	0.5142
AGE	1	6.6064	0.0102
GENDER	1	0.0252	0.8739
ETHNIC	1	0.3474	0.5556
H_DIAB01	1	2.3759	0.1232
H_AF01	1	1.9080	0.1672
H_HYPT01	1	2.8594	0.0908
H_LIPID01	1	0.0966	0.7560
AI	1	0.2128	0.6446
H_DRINK_H01	1	4.2486	0.0393
H_SMK_C01	1	2.6056	0.1065
IT	1	0.7055	0.4009
ET	1	5.5794	0.0182
IMG_C_TOAST	4	23.2982	0.0001
A_NIHSS	1	3.3222	0.0683

age1: BMI_g with y1_stroke: adjusted model

PHREG 过程

最大似然估计分析									
参数		自由度	参数估计	标准误差	卡方	Pr > 卡方	危险率	95% 危险率置信限	
BMI_g	4	1	0.05653	0.11043	0.2621	0.6087	1.058	0.852	1.314
BMI_g	3	1	-0.07808	0.09002	0.7524	0.3857	0.925	0.775	1.103
BMI_g	1	1	0.02862	0.32535	0.0077	0.9299	1.029	0.544	1.947
AGE		1	0.01348	0.00524	6.6064	0.0102	1.014	1.003	1.024
GENDER		1	0.01460	0.09201	0.0252	0.8739	1.015	0.847	1.215
ETHNIC	2	1	-0.13080	0.22193	0.3474	0.5556	0.877	0.568	1.356
H_DIAB01	1	1	0.13211	0.08571	2.3759	0.1232	1.141	0.965	1.350
H_AF01	1	1	0.32750	0.23709	1.9080	0.1672	1.387	0.872	2.208
H_HYPT01	1	1	0.13376	0.07910	2.8594	0.0908	1.143	0.979	1.335
H_LIPID01	1	1	-0.04145	0.13338	0.0966	0.7560	0.959	0.739	1.246
AI	1	1	-0.14156	0.30689	0.2128	0.6446	0.868	0.476	1.584
H_DRINK_H01	1	1	0.21096	0.10235	4.2486	0.0393	1.235	1.010	1.509
H_SMK_C01	1	1	-0.14496	0.08980	2.6056	0.1065	0.865	0.725	1.032
IT	1	1	0.09637	0.11473	0.7055	0.4009	1.101	0.879	1.379
ET	1	1	0.78479	0.33224	5.5794	0.0182	2.192	1.143	4.204
IMG_C_TOAST	5	1	-0.26890	0.08583	9.8143	0.0017	0.764	0.646	0.904
IMG_C_TOAST	4	1	-0.04914	0.29664	0.0274	0.8684	0.952	0.532	1.703
IMG_C_TOAST	3	1	-0.49273	0.10775	20.9120	<.0001	0.611	0.495	0.755
IMG_C_TOAST	2	1	-0.46891	0.26224	3.1972	0.0738	0.626	0.374	1.046
A_NIHSS		1	0.01623	0.00890	3.3222	0.0683	1.016	0.999	1.034

age1: BMI_g with y1_stroke: adjusted model

PHREG 过程

最大似然估计分析		
参数		标签
BMI_g	4	1=<18.5;2=18.5-<23;3=23-<27.5;4= ≥ 27.5 4
BMI_g	3	1=<18.5;2=18.5-<23;3=23-<27.5;4= ≥ 27.5 3
BMI_g	1	1=<18.5;2=18.5-<23;3=23-<27.5;4= ≥ 27.5 1
AGE		A.Basic Information: Age (years old);
GENDER		A.Basic Information: Gender; 1-male; 2-female;
ETHNIC	2	B.Demography: Race: 1-Han; 99-others; 2
H_DIAB01	1	D.History: Diabetes; 0-No; 1-Yes; 1
H_AF01	1	D.History: Heart disease category: Atrial fibrillation(Including medical history and hospitalization diagnosis); 0-No; 1-Yes; 1
H_HYPT01	1	D.History: Hypertension; 0-No; 1-Yes; 1
H_LIPID01	1	D.History: Lipid metabolism disorders; 0-No; 1-Yes; 1
AI	1	history:Myocardial infarction; 0=NO; 1=YES; 1
H_DRINK_H01	1	D.History: Heavy Drinking(Alcohol consumption>=20g/day); 0-No,1-Yes; 1
H_SMK_C01	1	D.History: Current Smoking; 0-No,1-Yes; 1
IT	1	intravenous thrombolysis, 1=YES,0=NO 1
ET	1	动脉溶栓或机械取栓, 1=YES,0=NO 1
IMG_C_TOAST	5	K.Final diagnosis: cerebral infarction; Etiology according to TOAST system; 1-large artery atherosclerosis; 2-cardiogenic embolism; 3-small artery occlusion; 4-stroke of another determined cause; 5-stroke of an undetermined cause. 5
IMG_C_TOAST	4	K.Final diagnosis: cerebral infarction; Etiology according to TOAST system; 1-large artery atherosclerosis; 2-cardiogenic embolism; 3-small artery occlusion; 4-stroke of another determined cause; 5-stroke of an undetermined cause. 4
IMG_C_TOAST	3	K.Final diagnosis: cerebral infarction; Etiology according to TOAST system; 1-large artery atherosclerosis; 2-cardiogenic embolism; 3-small artery occlusion; 4-stroke of another determined cause; 5-stroke of an undetermined cause. 3
IMG_C_TOAST	2	K.Final diagnosis: cerebral infarction; Etiology according to TOAST system; 1-large artery atherosclerosis; 2-cardiogenic embolism; 3-small artery occlusion; 4-stroke of another determined cause; 5-stroke of an undetermined cause. 2
A_NIHSS		F.Admitting NIHSS: Total score;

BMI_g with y1_stroke: interaction with age_group

PHREG 过程

模型信息		
数据集	WORK.DATA_OVERALL	
因变量	y1_stroke_dd	N12.Follow-up events at 12 months: Days from onset to recurrence;(day);
删失变量	y1_stroke	N12.Follow-up events at 12 months: Recurrence of stroke: 0-No; 1-Yes;
删失值	0	
结值处理	BRESLOW	

读取的观测数<br>使用的观测数	14146<br>14146

分类水平信息						
分类	值	设计变量				
BMI_g	4	1	0	0	
	3	0	1	0	
	2	0	0	0	
	1	0	0	1	
ETHNIC	2	1			
	1	0			
H_DIAB01	1	1			
	0	0			
H_AF01	1	1			
	0	0			
H_HYPT01	1	1			
	0	0			
H_LIPID01	1	1			
	0	0			
AI	1	1			
	0	0			
H_DRINK_H01	1	1			
	0	0			
H_SMK_C01	1	1			
	0	0			
IT	1	1			
	0	0			
ET	1	1			
	0	0			
IMG_C_TOAST	5	1	0	0	0
	4	0	1	0	0
	3	0	0	1	0
	2	0	0	0	1
	1	0	0	0	0

BMI_g with y1_stroke: interaction with age_group

PHREG 过程

事件和删失值个数汇总			
合计	事件	删失	删失百分比
14146	1424	12722	89.93

收敛状态
满足收敛准则 (GCONV=1E-8)。

模型拟合统计量		
准则	无协变量	带协变量
-2 LOG L	27024.892	26885.374
AIC	27024.892	26931.374
SBC	27024.892	27052.382

检验全局原假设: BETA=0			
检验	卡方	自由度	Pr > 卡方
似然比	139.5185	23	<.0001
评分	148.8691	23	<.0001
Wald	145.6058	23	<.0001

联合检验			
效应	自由度	Wald 卡方	Pr > 卡方
BMI_g	3	2.1633	0.5392
age_group	1	0.0036	0.9524
age_group*BMI_g	3	2.3730	0.4987
GENDER	1	0.6475	0.4210
ETHNIC	1	0.6400	0.4237
H_DIAB01	1	12.3721	0.0004
H_AF01	1	10.3310	0.0013
H_HYPT01	1	1.8904	0.1692
H_LIPID01	1	0.6589	0.4169
AI	1	1.2035	0.2726
H_DRINK_H01	1	2.8726	0.0901
H_SMK_C01	1	1.3004	0.2541
IT	1	1.0157	0.3135
ET	1	5.2570	0.0219
IMG_C_TOAST	4	53.4902	<.0001
A_NIHSS	1	13.0395	0.0003

Note: Under full-rank parameterizations, Type 3 effect tests are replaced by joint tests. The joint test for an effect is a test that all of the parameters associated with that effect are zero. Such joint tests might not be equivalent to Type 3 effect tests under GLM parameterization.

BMI_g with y1_stroke: interaction with age_group

PHREG 过程

最大似然估计分析									
参数		自由度	参数估计	标准误差	卡方	Pr > 卡方	危险率	95%危险率置信限	
BMI_g	4	1	-0.05820	0.24802	0.0551	0.8145	.	.	.
BMI_g	3	1	-0.26849	0.19965	1.8086	0.1787	.	.	.
BMI_g	1	1	-0.20841	0.68259	0.0932	0.7601	.	.	.
age_group		1	0.00619	0.10371	0.0036	0.9524	.	.	.
age_group*BMI_g	4	1	0.09381	0.16239	0.3337	0.5635	.	.	.
age_group*BMI_g	3	1	0.18963	0.12624	2.2565	0.1331	.	.	.
age_group*BMI_g	1	1	0.21575	0.38635	0.3118	0.5766	.	.	.
GENDER		1	0.05028	0.06248	0.6475	0.4210	1.052	0.930	1.189
ETHNIC	2	1	-0.13355	0.16694	0.6400	0.4237	0.875	0.631	1.214
H_DIAB01	1	1	0.21382	0.06079	12.3721	0.0004	1.238	1.099	1.395
H_AF01	1	1	0.39140	0.12177	10.3310	0.0013	1.479	1.165	1.878
H_HYPT01	1	1	0.07864	0.05719	1.8904	0.1692	1.082	0.967	1.210
H_LIPID01	1	1	-0.08323	0.10253	0.6589	0.4169	0.920	0.753	1.125
AI	1	1	0.18434	0.16803	1.2035	0.2726	1.202	0.865	1.671
H_DRINK_H01	1	1	0.14189	0.08372	2.8726	0.0901	1.152	0.978	1.358
H_SMK_C01	1	1	-0.07875	0.06906	1.3004	0.2541	0.924	0.807	1.058
IT	1	1	0.08376	0.08311	1.0157	0.3135	1.087	0.924	1.280
ET	1	1	0.59046	0.25752	5.2570	0.0219	1.805	1.089	2.990
IMG_C_TOAST	5	1	-0.34266	0.06301	29.5705	<.0001	0.710	0.627	0.803
IMG_C_TOAST	4	1	-0.06426	0.22873	0.0789	0.7788	0.938	0.599	1.468
IMG_C_TOAST	3	1	-0.53192	0.08092	43.2155	<.0001	0.587	0.501	0.688
IMG_C_TOAST	2	1	-0.44568	0.14230	9.8098	0.0017	0.640	0.485	0.846
A_NIHSS		1	0.02141	0.00593	13.0395	0.0003	1.022	1.010	1.034

BMI_g with y1_stroke: interaction with age_group

PHREG 过程

最大似然估计分析		
参数		标签
BMI_g	4	1=<18.5;2=18.5-<23;3=23-<27.5;4=≥27.5 4
BMI_g	3	1=<18.5;2=18.5-<23;3=23-<27.5;4=≥27.5 3
BMI_g	1	1=<18.5;2=18.5-<23;3=23-<27.5;4=≥27.5 1
age_group		1=age<65;2=age>=65
age_group*BMI_g	4	1=<18.5;2=18.5-<23;3=23-<27.5;4=≥27.5 4 * 1=age<65;2=age>=65
age_group*BMI_g	3	1=<18.5;2=18.5-<23;3=23-<27.5;4=≥27.5 3 * 1=age<65;2=age>=65
age_group*BMI_g	1	1=<18.5;2=18.5-<23;3=23-<27.5;4=≥27.5 1 * 1=age<65;2=age>=65
GENDER		A.Basic Information: Gender; 1-male; 2-female;
ETHNIC	2	B.Demography: Race: 1-Han; 99-others; 2
H_DIAB01	1	D.History: Diabetes; 0-No; 1-Yes; 1
H_AF01	1	D.History: Heart disease category: Atrial fibrillation(Including medical history and hospitalization diagnosis); 0-No; 1-Yes; 1
H_HYPT01	1	D.History: Hypertension; 0-No; 1-Yes; 1
H_LIPID01	1	D.History: Lipid metabolism disorders; 0-No; 1-Yes; 1
AI	1	history:Myocardial infarction; 0=NO; 1=YES; 1
H_DRINK_H01	1	D.History: Heavy Drinking(Alcohol consumption>=20g/day); 0-No,1-Yes; 1
H_SMK_C01	1	D.History: Current Smoking; 0-No,1-Yes; 1
IT	1	intravenous thrombolysis, 1=YES,0=NO 1
ET	1	动脉溶栓或机械取栓, 1=YES,0=NO 1
IMG_C_TOAST	5	K.Final diagnosis: cerebral infarction; Etiology according to TOAST system; 1-large artery atherosclerosis; 2-cardiogenic embolism; 3-small artery occlusion; 4-stroke of another determined cause; 5-stroke of an undetermined cause. 5
IMG_C_TOAST	4	K.Final diagnosis: cerebral infarction; Etiology according to TOAST system; 1-large artery atherosclerosis; 2-cardiogenic embolism; 3-small artery occlusion; 4-stroke of another determined cause; 5-stroke of an undetermined cause. 4
IMG_C_TOAST	3	K.Final diagnosis: cerebral infarction; Etiology according to TOAST system; 1-large artery atherosclerosis; 2-cardiogenic embolism; 3-small artery occlusion; 4-stroke of another determined cause; 5-stroke of an undetermined cause. 3
IMG_C_TOAST	2	K.Final diagnosis: cerebral infarction; Etiology according to TOAST system; 1-large artery atherosclerosis; 2-cardiogenic embolism; 3-small artery occlusion; 4-stroke of another determined cause; 5-stroke of an undetermined cause. 2
A_NIHSS		F.Admitting NIHSS: Total score;

age2: BMI_g with y1_death: Descriptive results

FREQ 过程

频数<br>行百分比

BMI_g-y1_death表			
	y1_death(N12.Follow-up events at 12 months: Whether the patient died: 0-survival;1-death;)		
BMI_g(1=<18.5;2=18.5-<23;3=23-<27.5;4=≥27.5)	0	1	合计
1	182<br>86.26	29<br>13.74	211
2	1809<br>93.73	121<br>6.27	1930
3	2880<br>94.64	163<br>5.36	3043
4	838<br>95.55	39<br>4.45	877
合计	5709	352	6061

表“y1_death-BMI_g”的统计量

统计量	自由度	值	概率
卡方	3	29.1475	<.0001
似然比卡方检验	3	23.0341	<.0001
Mantel-Haenszel 卡方	1	16.3926	<.0001
Phi 系数		0.0693	
列联系数		0.0692	
Cramer V		0.0693	

样本大小 = 6061

age2: BMI_g with y1_death: adjusted model

PHREG 过程

模型信息		
数据集	WORK.AGE2	
因变量	y1_death_dd	N12.Follow-up events at 12 months: Days from onset to death;(day);
删失变量	y1_death	N12.Follow-up events at 12 months: Whether the patient died: 0-survival;1-death;
删失值	0	
结值处理	BRESLOW	

读取的观测数	6061
使用的观测数	6061

分类水平信息						
分类	值	设计变量				
BMI_g	4	1	0	0	
	3	0	1	0	
	2	0	0	0	
	1	0	0	1	
ETHNIC	2	1			
	1	0			
H_DIAB01	1	1			
	0	0			
H_AF01	1	1			
	0	0			
H_HYPT01	1	1			
	0	0			
H_LIPID01	1	1			
	0	0			
AI	1	1			
	0	0			
H_DRINK_H01	1	1			
	0	0			
H_SMK_C01	1	1			
	0	0			
IT	1	1			
	0	0			
ET	1	1			
	0	0			
IMG_C_TOAST	5	1	0	0	0
	4	0	1	0	0
	3	0	0	1	0
	2	0	0	0	1
	1	0	0	0	0

age2: BMI_g with y1_death: adjusted model

PHREG 过程

事件和删失值个数汇总			
合计	事件	删失	删失百分比
6061	352	5709	94.19

收敛状态
满足收敛准则 (GCONV=1E-8)。

模型拟合统计量		
准则	无协变量	带协变量
-2 LOG L	6098.509	5728.926
AIC	6098.509	5768.926
SBC	6098.509	5846.199

检验全局原假设: BETA=0			
检验	卡方	自由度	Pr > 卡方
似然比	369.5830	20	<.0001
评分	509.9448	20	<.0001
Wald	453.4298	20	<.0001

3 型检验			
效应	自由度	Wald 卡方	Pr > 卡方
BMI_g	3	8.0359	0.0453
AGE	1	76.2497	<.0001
GENDER	1	0.2086	0.6479
ETHNIC	1	2.7068	0.0999
H_DIAB01	1	6.7461	0.0094
H_AF01	1	15.0331	0.0001
H_HYPT01	1	0.9256	0.3360
H_LIPID01	1	1.7936	0.1805
AI	1	1.7827	0.1818
H_DRINK_H01	1	3.5312	0.0602
H_SMK_C01	1	4.7228	0.0298
IT	1	12.7453	0.0004
ET	1	7.0109	0.0081
IMG_C_TOAST	4	23.5005	0.0001
A_NIHSS	1	171.1912	<.0001

age2: BMI_g with y1_death: adjusted model

PHREG 过程

最大似然估计分析									
参数		自由度	参数估计	标准误差	卡方	Pr > 卡方	危险率	95% 危险率置信限	
BMI_g	4	1	-0.17265	0.18807	0.8428	0.3586	0.841	0.582	1.216
BMI_g	3	1	-0.00915	0.12311	0.0055	0.9407	0.991	0.778	1.261
BMI_g	1	1	0.50889	0.20934	5.9096	0.0151	1.663	1.104	2.507
AGE		1	0.07349	0.00842	76.2497	<.0001	1.076	1.059	1.094
GENDER		1	-0.05363	0.11742	0.2086	0.6479	0.948	0.753	1.193
ETHNIC	2	1	0.47050	0.28598	2.7068	0.0999	1.601	0.914	2.804
H_DIAB01	1	1	0.32588	0.12547	6.7461	0.0094	1.385	1.083	1.771
H_AF01	1	1	0.63187	0.16297	15.0331	0.0001	1.881	1.367	2.589
H_HYPT01	1	1	0.11230	0.11673	0.9256	0.3360	1.119	0.890	1.406
H_LIPID01	1	1	-0.34621	0.25851	1.7936	0.1805	0.707	0.426	1.174
AI	1	1	0.36782	0.27548	1.7827	0.1818	1.445	0.842	2.479
H_DRINK_H01	1	1	-0.49502	0.26343	3.5312	0.0602	0.610	0.364	1.022
H_SMK_C01	1	1	0.32668	0.15032	4.7228	0.0298	1.386	1.033	1.861
IT	1	1	-0.68651	0.19230	12.7453	0.0004	0.503	0.345	0.734
ET	1	1	1.03705	0.39166	7.0109	0.0081	2.821	1.309	6.078
IMG_C_TOAST	5	1	-0.17898	0.13462	1.7677	0.1837	0.836	0.642	1.089
IMG_C_TOAST	4	1	0.75823	0.39228	3.7359	0.0533	2.134	0.989	4.605
IMG_C_TOAST	3	1	-0.98990	0.23592	17.6058	<.0001	0.372	0.234	0.590
IMG_C_TOAST	2	1	-0.18396	0.21074	0.7620	0.3827	0.832	0.550	1.257
A_NIHSS		1	0.09459	0.00723	171.1912	<.0001	1.099	1.084	1.115

age2: BMI_g with y1_death: adjusted model

PHREG 过程

最大似然估计分析		
参数		标签
BMI_g	4	1=<18.5;2=18.5-<23;3=23-<27.5;4= ≥ 27.5 4
BMI_g	3	1=<18.5;2=18.5-<23;3=23-<27.5;4= ≥ 27.5 3
BMI_g	1	1=<18.5;2=18.5-<23;3=23-<27.5;4= ≥ 27.5 1
AGE		A.Basic Information: Age (years old);
GENDER		A.Basic Information: Gender; 1-male; 2-female;
ETHNIC	2	B.Demography: Race: 1-Han; 99-others; 2
H_DIAB01	1	D.History: Diabetes; 0-No; 1-Yes; 1
H_AF01	1	D.History: Heart disease category: Atrial fibrillation(Including medical history and hospitalization diagnosis); 0-No; 1-Yes; 1
H_HYPT01	1	D.History: Hypertension; 0-No; 1-Yes; 1
H_LIPID01	1	D.History: Lipid metabolism disorders; 0-No; 1-Yes; 1
AI	1	history:Myocardial infarction; 0=NO; 1=YES; 1
H_DRINK_H01	1	D.History: Heavy Drinking(Alcohol consumption>=20g/day); 0-No,1-Yes; 1
H_SMK_C01	1	D.History: Current Smoking; 0-No,1-Yes; 1
IT	1	intravenous thrombolysis, 1=YES,0=NO 1
ET	1	动脉溶栓或机械取栓, 1=YES,0=NO 1
IMG_C_TOAST	5	K.Final diagnosis: cerebral infarction; Etiology according to TOAST system; 1-large artery atherosclerosis; 2-cardiogenic embolism; 3-small artery occlusion; 4-stroke of another determined cause; 5-stroke of an undetermined cause. 5
IMG_C_TOAST	4	K.Final diagnosis: cerebral infarction; Etiology according to TOAST system; 1-large artery atherosclerosis; 2-cardiogenic embolism; 3-small artery occlusion; 4-stroke of another determined cause; 5-stroke of an undetermined cause. 4
IMG_C_TOAST	3	K.Final diagnosis: cerebral infarction; Etiology according to TOAST system; 1-large artery atherosclerosis; 2-cardiogenic embolism; 3-small artery occlusion; 4-stroke of another determined cause; 5-stroke of an undetermined cause. 3
IMG_C_TOAST	2	K.Final diagnosis: cerebral infarction; Etiology according to TOAST system; 1-large artery atherosclerosis; 2-cardiogenic embolism; 3-small artery occlusion; 4-stroke of another determined cause; 5-stroke of an undetermined cause. 2
A_NIHSS		F.Admitting NIHSS: Total score;

BMI_g with y1_death: interaction with age_group

PHREG 过程

模型信息		
数据集	WORK.DATA_OVERALL	
因变量	y1_death_dd	N12.Follow-up events at 12 months: Days from onset to death;(day);
删失变量	y1_death	N12.Follow-up events at 12 months: Whether the patient died: 0-survival;1-death;
删失值	0	
结值处理	BRESLOW	

读取的观测数	14146
使用的观测数	14146

分类水平信息						
分类	值	设计变量				
BMI_g	4	1	0	0	
	3	0	1	0	
	2	0	0	0	
	1	0	0	1	
ETHNIC	2	1			
	1	0			
H_DIAB01	1	1			
	0	0			
H_AF01	1	1			
	0	0			
H_HYPT01	1	1			
	0	0			
H_LIPID01	1	1			
	0	0			
AI	1	1			
	0	0			
H_DRINK_H01	1	1			
	0	0			
H_SMK_C01	1	1			
	0	0			
IT	1	1			
	0	0			
ET	1	1			
	0	0			
IMG_C_TOAST	5	1	0	0	0
	4	0	1	0	0
	3	0	0	1	0
	2	0	0	0	1
	1	0	0	0	0

BMI_g with y1_death: interaction with age_group

PHREG 过程

事件和删失值个数汇总			
合计	事件	删失	删失百分比
14146	486	13660	96.56

收敛状态
满足收敛准则 (GCONV=1E-8)。

模型拟合统计量		
准则	无协变量	带协变量
-2 LOG L	9255.945	8678.485
AIC	9255.945	8724.485
SBC	9255.945	8820.768

检验全局原假设: BETA=0			
检验	卡方	自由度	Pr > 卡方
似然比	577.4600	23	<.0001
评分	833.1321	23	<.0001
Wald	698.4128	23	<.0001

联合检验			
效应	自由度	Wald 卡方	Pr > 卡方
BMI_g	3	1.8060	0.6136
age_group	1	24.7221	<.0001
age_group*BMI_g	3	0.3651	0.9473
GENDER	1	1.3142	0.2516
ETHNIC	1	2.4636	0.1165
H_DIAB01	1	11.3115	0.0008
H_AF01	1	40.1273	<.0001
H_HYPT01	1	0.5508	0.4580
H_LIPID01	1	2.4115	0.1205
AI	1	4.9108	0.0267
H_DRINK_H01	1	7.9483	0.0048
H_SMK_C01	1	0.0101	0.9199
IT	1	20.1519	<.0001
ET	1	5.5470	0.0185
IMG_C_TOAST	4	34.9058	<.0001
A_NIHSS	1	233.6415	<.0001

Note: Under full-rank parameterizations, Type 3 effect tests are replaced by joint tests. The joint test for an effect is a test that all of the parameters associated with that effect are zero. Such joint tests might not be equivalent to Type 3 effect tests under GLM parameterization.

BMI_g with y1_death: interaction with age_group

PHREG 过程

最大似然估计分析									
参数		自由度	参数估计	标准误差	卡方	Pr > 卡方	危险率	95% 危险率置信限	
BMI_g	4	1	-0.74790	0.59657	1.5717	0.2100	.	.	.
BMI_g	3	1	-0.22391	0.41164	0.2959	0.5865	.	.	.
BMI_g	1	1	0.35064	1.21610	0.0831	0.7731	.	.	.
age_group		1	0.91776	0.18458	24.7221	<.0001	.	.	.
age_group*BMI_g	4	1	0.19970	0.33817	0.3487	0.5548	.	.	.
age_group*BMI_g	3	1	0.05802	0.23053	0.0633	0.8013	.	.	.
age_group*BMI_g	1	1	0.13657	0.63445	0.0463	0.8296	.	.	.
GENDER		1	-0.11795	0.10289	1.3142	0.2516	0.889	0.726	1.087
ETHNIC	2	1	0.36936	0.23532	2.4636	0.1165	1.447	0.912	2.295
H_DIAB01	1	1	0.35038	0.10418	11.3115	0.0008	1.420	1.157	1.741
H_AF01	1	1	0.94786	0.14963	40.1273	<.0001	2.580	1.924	3.460
H_HYPT01	1	1	0.07255	0.09776	0.5508	0.4580	1.075	0.888	1.302
H_LIPID01	1	1	-0.32256	0.20772	2.4115	0.1205	0.724	0.482	1.088
AI	1	1	0.50480	0.22779	4.9108	0.0267	1.657	1.060	2.589
H_DRINK_H01	1	1	-0.54982	0.19502	7.9483	0.0048	0.577	0.394	0.846
H_SMK_C01	1	1	0.01239	0.12324	0.0101	0.9199	1.012	0.795	1.289
IT	1	1	-0.74154	0.16519	20.1519	<.0001	0.476	0.345	0.659
ET	1	1	0.73803	0.31336	5.5470	0.0185	2.092	1.132	3.866
IMG_C_TOAST	5	1	-0.23482	0.11145	4.4390	0.0351	0.791	0.636	0.984
IMG_C_TOAST	4	1	0.58592	0.32745	3.2017	0.0736	1.797	0.946	3.413
IMG_C_TOAST	3	1	-0.94131	0.18276	26.5290	<.0001	0.390	0.273	0.558
IMG_C_TOAST	2	1	-0.46379	0.19527	5.6412	0.0175	0.629	0.429	0.922
A_NIHSS		1	0.09573	0.00626	233.6415	<.0001	1.100	1.087	1.114

BMI_g with y1_death: interaction with age_group

PHREG 过程

最大似然估计分析		
参数		标签
BMI_g	4	1=<18.5;2=18.5-<23;3=23-<27.5;4=≥27.5 4
BMI_g	3	1=<18.5;2=18.5-<23;3=23-<27.5;4=≥27.5 3
BMI_g	1	1=<18.5;2=18.5-<23;3=23-<27.5;4=≥27.5 1
age_group		1=age<65;2=age>=65
age_group*BMI_g	4	1=<18.5;2=18.5-<23;3=23-<27.5;4=≥27.5 4 * 1=age<65;2=age>=65
age_group*BMI_g	3	1=<18.5;2=18.5-<23;3=23-<27.5;4=≥27.5 3 * 1=age<65;2=age>=65
age_group*BMI_g	1	1=<18.5;2=18.5-<23;3=23-<27.5;4=≥27.5 1 * 1=age<65;2=age>=65
GENDER		A.Basic Information: Gender; 1-male; 2-female;
ETHNIC	2	B.Demography: Race: 1-Han; 99-others; 2
H_DIAB01	1	D.History: Diabetes; 0-No; 1-Yes; 1
H_AF01	1	D.History: Heart disease category: Atrial fibrillation(Including medical history and hospitalization diagnosis); 0-No; 1-Yes; 1
H_HYPT01	1	D.History: Hypertension; 0-No; 1-Yes; 1
H_LIPID01	1	D.History: Lipid metabolism disorders; 0-No; 1-Yes; 1
AI	1	history:Myocardial infarction; 0=NO; 1=YES; 1
H_DRINK_H01	1	D.History: Heavy Drinking(Alcohol consumption>=20g/day); 0-No,1-Yes; 1
H_SMK_C01	1	D.History: Current Smoking; 0-No,1-Yes; 1
IT	1	intravenous thrombolysis, 1=YES,0=NO 1
ET	1	动脉溶栓或机械取栓, 1=YES,0=NO 1
IMG_C_TOAST	5	K.Final diagnosis: cerebral infarction; Etiology according to TOAST system; 1-large artery atherosclerosis; 2-cardiogenic embolism; 3-small artery occlusion; 4-stroke of another determined cause; 5-stroke of an undetermined cause. 5
IMG_C_TOAST	4	K.Final diagnosis: cerebral infarction; Etiology according to TOAST system; 1-large artery atherosclerosis; 2-cardiogenic embolism; 3-small artery occlusion; 4-stroke of another determined cause; 5-stroke of an undetermined cause. 4
IMG_C_TOAST	3	K.Final diagnosis: cerebral infarction; Etiology according to TOAST system; 1-large artery atherosclerosis; 2-cardiogenic embolism; 3-small artery occlusion; 4-stroke of another determined cause; 5-stroke of an undetermined cause. 3
IMG_C_TOAST	2	K.Final diagnosis: cerebral infarction; Etiology according to TOAST system; 1-large artery atherosclerosis; 2-cardiogenic embolism; 3-small artery occlusion; 4-stroke of another determined cause; 5-stroke of an undetermined cause. 2
A_NIHSS		F.Admitting NIHSS: Total score;

age2: BMI_g with y1_comb: Descriptive results

FREQ 过程

频数<br>行百分比

BMI_g-y1_comb表			
	y1_comb(N12.Follow-up events at 12 months:Occurrence of combined vascular event(including cardiovascular death,non-fatal stroke,non-fatal myocardial infarction):0-No;1-Yes;)		
BMI_g(1=<18.5;2=18.5-<23;3=23-<27.5;4=≥27.5)	0	1	合计
1	184<br>87.20	27<br>12.80	211
2	1722<br>89.22	208<br>10.78	1930
3	2672<br>87.81	371<br>12.19	3043
4	765<br>87.23	112<br>12.77	877
合计	5343	718	6061

表“y1_comb-BMI_g”的统计量

统计量	自由度	值	概率
卡方	3	3.3606	0.3393
似然比卡方检验	3	3.3957	0.3345
Mantel-Haenszel 卡方	1	1.7789	0.1823
Phi 系数		0.0235	
列联系数		0.0235	
Cramer V		0.0235	

样本大小 = 6061

age2: BMI_g with y1_comb: adjusted model

PHREG 过程

模型信息		
数据集	WORK.AGE2	
因变量	y1_comb_dd	N12.Follow-up events at 12 months: Days from onset to occurrence of combined vascular event;(day);
删失变量	y1_comb	N12.Follow-up events at 12 months:Occurrence of combined vascular event(including cardiovascular death,non-fatal stroke,non-fatal myocardial infarction):0-No;1-Yes;
删失值	0	
结值处理	BRESLOW	

读取的观测数	6061
使用的观测数	6061

分类水平信息					
分类	值	设计变量			
BMI_g	4	1	0	0	
	3	0	1	0	
	2	0	0	0	
	1	0	0	1	
ETHNIC	2	1			
	1	0			
H_DIAB01	1	1			
	0	0			
H_AF01	1	1			
	0	0			
H_HYPT01	1	1			
	0	0			
H_LIPID01	1	1			
	0	0			
AI	1	1			
	0	0			
H_DRINK_H01	1	1			
	0	0			
H_SMK_C01	1	1			
	0	0			
IT	1	1			
	0	0			
ET	1	1			
	0	0			
IMG_C_TOAST	5	1	0	0	0
	4	0	1	0	0
	3	0	0	1	0
	2	0	0	0	1
	1	0	0	0	0

age2: BMI_g with y1_comb: adjusted model

PHREG 过程

事件和删失值个数汇总			
合计	事件	删失	删失百分比
6061	718	5343	88.15

收敛状态
满足收敛准则 (GCONV=1E-8)。

模型拟合统计量		
准则	无协变量	带协变量
-2 LOG L	12388.894	12286.558
AIC	12388.894	12326.558
SBC	12388.894	12418.088

检验全局原假设: BETA=0			
检验	卡方	自由度	Pr > 卡方
似然比	102.3360	20	<.0001
评分	108.3134	20	<.0001
Wald	105.8286	20	<.0001

3 型检验			
效应	自由度	Wald 卡方	Pr > 卡方
BMI_g	3	3.5294	0.3170
AGE	1	9.8468	0.0017
GENDER	1	0.3756	0.5400
ETHNIC	1	0.0764	0.7823
H_DIAB01	1	13.2728	0.0003
H_AF01	1	10.7755	0.0010
H_HYPT01	1	0.0042	0.9485
H_LIPID01	1	0.3477	0.5554
AI	1	2.1034	0.1470
H_DRINK_H01	1	0.0482	0.8263
H_SMK_C01	1	1.0295	0.3103
IT	1	0.1107	0.7394
ET	1	1.9258	0.1652
IMG_C_TOAST	4	32.9054	<.0001
A_NIHSS	1	14.5912	0.0001

age2: BMI_g with y1_comb: adjusted model

PHREG 过程

最大似然估计分析									
参数		自由度	参数估计	标准误差	卡方	Pr > 卡方	危险率	95% 危险率置信限	
BMI_g	4	1	0.18487	0.11905	2.4114	0.1205	1.203	0.953	1.519
BMI_g	3	1	0.14586	0.08765	2.7697	0.0961	1.157	0.974	1.374
BMI_g	1	1	0.13358	0.20614	0.4199	0.5170	1.143	0.763	1.712
AGE		1	0.01993	0.00635	9.8468	0.0017	1.020	1.008	1.033
GENDER		1	0.05059	0.08255	0.3756	0.5400	1.052	0.895	1.237
ETHNIC	2	1	-0.06617	0.23946	0.0764	0.7823	0.936	0.585	1.497
H_DIAB01	1	1	0.30580	0.08394	13.2728	0.0003	1.358	1.152	1.600
H_AF01	1	1	0.45261	0.13788	10.7755	0.0010	1.572	1.200	2.060
H_HYPT01	1	1	-0.00522	0.08079	0.0042	0.9485	0.995	0.849	1.165
H_LIPID01	1	1	-0.09069	0.15382	0.3477	0.5554	0.913	0.676	1.235
AI	1	1	0.28673	0.19770	2.1034	0.1470	1.332	0.904	1.963
H_DRINK_H01	1	1	-0.03188	0.14529	0.0482	0.8263	0.969	0.729	1.288
H_SMK_C01	1	1	0.10652	0.10498	1.0295	0.3103	1.112	0.906	1.367
IT	1	1	0.03930	0.11814	0.1107	0.7394	1.040	0.825	1.311
ET	1	1	0.53491	0.38546	1.9258	0.1652	1.707	0.802	3.634
IMG_C_TOAST	5	1	-0.41983	0.09102	21.2764	<.0001	0.657	0.550	0.785
IMG_C_TOAST	4	1	-0.03963	0.36020	0.0121	0.9124	0.961	0.474	1.947
IMG_C_TOAST	3	1	-0.57778	0.12075	22.8968	<.0001	0.561	0.443	0.711
IMG_C_TOAST	2	1	-0.45650	0.16592	7.5696	0.0059	0.633	0.458	0.877
A_NIHSS		1	0.02888	0.00756	14.5912	0.0001	1.029	1.014	1.045

age2: BMI_g with y1_comb: adjusted model

PHREG 过程

最大似然估计分析		
参数		标签
BMI_g	4	1=<18.5;2=18.5-<23;3=23-<27.5;4= ≥ 27.5 4
BMI_g	3	1=<18.5;2=18.5-<23;3=23-<27.5;4= ≥ 27.5 3
BMI_g	1	1=<18.5;2=18.5-<23;3=23-<27.5;4= ≥ 27.5 1
AGE		A.Basic Information: Age (years old);
GENDER		A.Basic Information: Gender; 1-male; 2-female;
ETHNIC	2	B.Demography: Race: 1-Han; 99-others; 2
H_DIAB01	1	D.History: Diabetes; 0-No; 1-Yes; 1
H_AF01	1	D.History: Heart disease category: Atrial fibrillation(Including medical history and hospitalization diagnosis); 0-No; 1-Yes; 1
H_HYPT01	1	D.History: Hypertension; 0-No; 1-Yes; 1
H_LIPID01	1	D.History: Lipid metabolism disorders; 0-No; 1-Yes; 1
AI	1	history:Myocardial infarction; 0=NO; 1=YES; 1
H_DRINK_H01	1	D.History: Heavy Drinking(Alcohol consumption>=20g/day); 0-No,1-Yes; 1
H_SMK_C01	1	D.History: Current Smoking; 0-No,1-Yes; 1
IT	1	intravenous thrombolysis, 1=YES,0=NO 1
ET	1	动脉溶栓或机械取栓, 1=YES,0=NO 1
IMG_C_TOAST	5	K.Final diagnosis: cerebral infarction; Etiology according to TOAST system; 1-large artery atherosclerosis; 2-cardiogenic embolism; 3-small artery occlusion; 4-stroke of another determined cause; 5-stroke of an undetermined cause. 5
IMG_C_TOAST	4	K.Final diagnosis: cerebral infarction; Etiology according to TOAST system; 1-large artery atherosclerosis; 2-cardiogenic embolism; 3-small artery occlusion; 4-stroke of another determined cause; 5-stroke of an undetermined cause. 4
IMG_C_TOAST	3	K.Final diagnosis: cerebral infarction; Etiology according to TOAST system; 1-large artery atherosclerosis; 2-cardiogenic embolism; 3-small artery occlusion; 4-stroke of another determined cause; 5-stroke of an undetermined cause. 3
IMG_C_TOAST	2	K.Final diagnosis: cerebral infarction; Etiology according to TOAST system; 1-large artery atherosclerosis; 2-cardiogenic embolism; 3-small artery occlusion; 4-stroke of another determined cause; 5-stroke of an undetermined cause. 2
A_NIHSS		F.Admitting NIHSS: Total score;

BMI_g with y1_comb: interaction with age_group

PHREG 过程

模型信息		
数据集	WORK.DATA_OVERALL	
因变量	y1_comb_dd	N12.Follow-up events at 12 months: Days from onset to occurrence of combined vascular event;(day);
删失变量	y1_comb	N12.Follow-up events at 12 months:Occurrence of combined vascular event(including cardiovascular death,non-fatal stroke,non-fatal myocardial infarction):0-No;1-Yes;
删失值	0	
结值处理	BRESLOW	

读取的观测数	14146
使用的观测数	14146

分类水平信息						
分类	值	设计变量				
BMI_g	4	1	0	0		
	3	0	1	0		
	2	0	0	0		
	1	0	0	1		
ETHNIC	2	1				
	1	0				
H_DIAB01	1	1				
	0	0				
H_AF01	1	1				
	0	0				
H_HYPT01	1	1				
	0	0				
H_LIPID01	1	1				
	0	0				
AI	1	1				
	0	0				
H_DRINK_H01	1	1				
	0	0				
H_SMK_C01	1	1				
	0	0				
IT	1	1				
	0	0				
ET	1	1				
	0	0				
IMG_C_TOAST	5	1	0	0	0	
	4	0	1	0	0	
	3	0	0	1	0	
	2	0	0	0	1	
	1	0	0	0	0	

BMI_g with y1_comb: interaction with age_group

PHREG 过程

事件和删失值个数汇总			
合计	事件	删失	删失百分比
14146	1505	12641	89.36

收敛状态
满足收敛准则 (GCONV=1E-8)。

模型拟合统计量		
准则	无协变量	带协变量
-2 LOG L	28553.433	28391.951
AIC	28553.433	28437.951
SBC	28553.433	28560.232

检验全局原假设: BETA=0			
检验	卡方	自由度	Pr > 卡方
似然比	161.4815	23	<.0001
评分	173.8273	23	<.0001
Wald	169.7249	23	<.0001

联合检验			
效应	自由度	Wald 卡方	Pr > 卡方
BMI_g	3	3.0456	0.3847
age_group	1	0.0231	0.8791
age_group*BMI_g	3	3.3167	0.3453
GENDER	1	0.2851	0.5934
ETHNIC	1	0.4702	0.4929
H_DIAB01	1	14.6036	0.0001
H_AF01	1	16.3244	<.0001
H_HYPT01	1	3.6829	0.0550
H_LIPID01	1	0.7085	0.3999
AI	1	2.4306	0.1190
H_DRINK_H01	1	1.1465	0.2843
H_SMK_C01	1	0.7793	0.3774
IT	1	0.1869	0.6655
ET	1	5.2005	0.0226
IMG_C_TOAST	4	55.0719	<.0001
A_NIHSS	1	16.6632	<.0001

Note: Under full-rank parameterizations, Type 3 effect tests are replaced by joint tests. The joint test for an effect is a test that all of the parameters associated with that effect are zero. Such joint tests might not be equivalent to Type 3 effect tests under GLM parameterization.

BMI_g with y1_comb: interaction with age_group

PHREG 过程

最大似然估计分析									
参数		自由度	参数估计	标准误差	卡方	Pr > 卡方	危险率	95% 危险率置信限	
BMI_g	4	1	-0.19472	0.24248	0.6449	0.4220	.	.	.
BMI_g	3	1	-0.32519	0.19365	2.8200	0.0931	.	.	.
BMI_g	1	1	0.07256	0.62934	0.0133	0.9082	.	.	.
age_group		1	-0.01529	0.10052	0.0231	0.8791	.	.	.
age_group*BMI_g	4	1	0.16715	0.15799	1.1194	0.2900	.	.	.
age_group*BMI_g	3	1	0.22000	0.12242	3.2295	0.0723	.	.	.
age_group*BMI_g	1	1	0.06197	0.36127	0.0294	0.8638	.	.	.
GENDER		1	0.03244	0.06075	0.2851	0.5934	1.033	0.917	1.164
ETHNIC	2	1	-0.11014	0.16061	0.4702	0.4929	0.896	0.654	1.227
H_DIAB01	1	1	0.22540	0.05898	14.6036	0.0001	1.253	1.116	1.406
H_AF01	1	1	0.46639	0.11543	16.3244	<.0001	1.594	1.271	1.999
H_HYPT01	1	1	0.10717	0.05584	3.6829	0.0550	1.113	0.998	1.242
H_LIPID01	1	1	-0.08394	0.09973	0.7085	0.3999	0.919	0.756	1.118
AI	1	1	0.24630	0.15798	2.4306	0.1190	1.279	0.939	1.744
H_DRINK_H01	1	1	0.08822	0.08239	1.1465	0.2843	1.092	0.929	1.284
H_SMK_C01	1	1	-0.05921	0.06708	0.7793	0.3774	0.943	0.826	1.075
IT	1	1	0.03538	0.08183	0.1869	0.6655	1.036	0.882	1.216
ET	1	1	0.56976	0.24984	5.2005	0.0226	1.768	1.083	2.885
IMG_C_TOAST	5	1	-0.33469	0.06152	29.5998	<.0001	0.716	0.634	0.807
IMG_C_TOAST	4	1	-0.05769	0.22323	0.0668	0.7961	0.944	0.609	1.462
IMG_C_TOAST	3	1	-0.53304	0.07918	45.3229	<.0001	0.587	0.502	0.685
IMG_C_TOAST	2	1	-0.42326	0.13576	9.7200	0.0018	0.655	0.502	0.855
A_NIHSS		1	0.02328	0.00570	16.6632	<.0001	1.024	1.012	1.035

BMI_g with y1_comb: interaction with age_group

PHREG 过程

最大似然估计分析		
参数		标签
BMI_g	4	1=<18.5;2=18.5-<23;3=23-<27.5;4= ≥ 27.5 4
BMI_g	3	1=<18.5;2=18.5-<23;3=23-<27.5;4= ≥ 27.5 3
BMI_g	1	1=<18.5;2=18.5-<23;3=23-<27.5;4= ≥ 27.5 1
age_group		1=age<65;2=age>=65
age_group*BMI_g	4	1=<18.5;2=18.5-<23;3=23-<27.5;4= ≥ 27.5 4 * 1=age<65;2=age>=65
age_group*BMI_g	3	1=<18.5;2=18.5-<23;3=23-<27.5;4= ≥ 27.5 3 * 1=age<65;2=age>=65
age_group*BMI_g	1	1=<18.5;2=18.5-<23;3=23-<27.5;4= ≥ 27.5 1 * 1=age<65;2=age>=65
GENDER		A.Basic Information: Gender; 1-male; 2-female;
ETHNIC	2	B.Demography: Race: 1-Han; 99-others; 2
H_DIAB01	1	D.History: Diabetes; 0-No; 1-Yes; 1
H_AF01	1	D.History: Heart disease category: Atrial fibrillation(Including medical history and hospitalization diagnosis); 0-No; 1-Yes; 1
H_HYPT01	1	D.History: Hypertension; 0-No; 1-Yes; 1
H_LIPID01	1	D.History: Lipid metabolism disorders; 0-No; 1-Yes; 1
AI	1	history:Myocardial infarction; 0=NO; 1=YES; 1
H_DRINK_H01	1	D.History: Heavy Drinking(Alcohol consumption>=20g/day); 0-No,1-Yes; 1
H_SMK_C01	1	D.History: Current Smoking; 0-No,1-Yes; 1
IT	1	intravenous thrombolysis, 1=YES,0=NO 1
ET	1	动脉溶栓或机械取栓, 1=YES,0=NO 1
IMG_C_TOAST	5	K.Final diagnosis: cerebral infarction; Etiology according to TOAST system; 1-large artery atherosclerosis; 2-cardiogenic embolism; 3-small artery occlusion; 4-stroke of another determined cause; 5-stroke of an undetermined cause. 5
IMG_C_TOAST	4	K.Final diagnosis: cerebral infarction; Etiology according to TOAST system; 1-large artery atherosclerosis; 2-cardiogenic embolism; 3-small artery occlusion; 4-stroke of another determined cause; 5-stroke of an undetermined cause. 4
IMG_C_TOAST	3	K.Final diagnosis: cerebral infarction; Etiology according to TOAST system; 1-large artery atherosclerosis; 2-cardiogenic embolism; 3-small artery occlusion; 4-stroke of another determined cause; 5-stroke of an undetermined cause. 3
IMG_C_TOAST	2	K.Final diagnosis: cerebral infarction; Etiology according to TOAST system; 1-large artery atherosclerosis; 2-cardiogenic embolism; 3-small artery occlusion; 4-stroke of another determined cause; 5-stroke of an undetermined cause. 2
A_NIHSS		F.Admitting NIHSS: Total score;

age2: BMI_g with y1_stroke: Descriptive results

FREQ 过程

频数<br>行百分比

BMI_g-y1_stroke表			
	y1_stroke(N12.Follow-up events at 12 months: Recurrence of stroke: 0-No; 1-Yes;)		
BMI_g(1=<18.5;2=18.5-<23;3=23-<27.5;4=≥27.5)	0	1	合计
1	185<br>87.68	26<br>12.32	211
2	1734<br>89.84	196<br>10.16	1930
3	2696<br>88.60	347<br>11.40	3043
4	773<br>88.14	104<br>11.86	877
合计	5388	673	6061

表“y1_stroke-BMI_g”的统计量

统计量	自由度	值	概率
卡方	3	2.8585	0.4140
似然比卡方检验	3	2.8828	0.4100
Mantel-Haenszel 卡方	1	1.2039	0.2725
Phi 系数		0.0217	
列联系数		0.0217	
Cramer V		0.0217	

样本大小 = 6061

age2: BMI_g with y1_stroke: adjusted model

PHREG 过程

模型信息		
数据集	WORK.AGE2	
因变量	y1_stroke_dd	N12.Follow-up events at 12 months: Days from onset to recurrence;(day);
删失变量	y1_stroke	N12.Follow-up events at 12 months: Recurrence of stroke: 0-No; 1-Yes;
删失值	0	
结值处理	BRESLOW	

读取的观测数	6061
使用的观测数	6061

分类水平信息						
分类	值	设计变量				
BMI_g	4	1	0	0	
	3	0	1	0	
	2	0	0	0	
	1	0	0	1	
ETHNIC	2	1			
	1	0			
H_DIAB01	1	1			
	0	0			
H_AF01	1	1			
	0	0			
H_HYPT01	1	1			
	0	0			
H_LIPID01	1	1			
	0	0			
AI	1	1			
	0	0			
H_DRINK_H01	1	1			
	0	0			
H_SMK_C01	1	1			
	0	0			
IT	1	1			
	0	0			
ET	1	1			
	0	0			
IMG_C_TOAST	5	1	0	0	0
	4	0	1	0	0
	3	0	0	1	0
	2	0	0	0	1
	1	0	0	0	0

age2: BMI_g with y1_stroke: adjusted model

PHREG 过程

事件和删失值个数汇总			
合计	事件	删失	删失百分比
6061	673	5388	88.90

收敛状态
满足收敛准则 (GCONV=1E-8)。

模型拟合统计量		
准则	无协变量	带协变量
-2 LOG L	11616.983	11535.353
AIC	11616.983	11575.353
SBC	11616.983	11665.588

检验全局原假设: BETA=0			
检验	卡方	自由度	Pr > 卡方
似然比	81.6305	20	<.0001
评分	85.6888	20	<.0001
Wald	83.9656	20	<.0001

3 型检验			
效应	自由度	Wald 卡方	Pr > 卡方
BMI_g	3	2.8573	0.4142
AGE	1	5.2001	0.0226
GENDER	1	0.5091	0.4755
ETHNIC	1	0.2323	0.6299
H_DIAB01	1	10.9989	0.0009
H_AF01	1	7.2646	0.0070
H_HYPT01	1	0.0402	0.8411
H_LIPID01	1	0.5531	0.4571
AI	1	2.6470	0.1037
H_DRINK_H01	1	0.0022	0.9629
H_SMK_C01	1	0.4262	0.5138
IT	1	0.4744	0.4910
ET	1	1.2839	0.2572
IMG_C_TOAST	4	31.3239	<.0001
A_NIHSS	1	9.7895	0.0018

age2: BMI_g with y1_stroke: adjusted model

PHREG 过程

最大似然估计分析									
参数		自由度	参数估计	标准误差	卡方	Pr > 卡方	危险率	95% 危险率置信限	
BMI_g	4	1	0.16237	0.12318	1.7376	0.1874	1.176	0.924	1.497
BMI_g	3	1	0.13410	0.09038	2.2015	0.1379	1.144	0.958	1.365
BMI_g	1	1	0.17569	0.21039	0.6974	0.4037	1.192	0.789	1.800
AGE		1	0.01507	0.00661	5.2001	0.0226	1.015	1.002	1.028
GENDER		1	0.06087	0.08531	0.5091	0.4755	1.063	0.899	1.256
ETHNIC	2	1	-0.12230	0.25377	0.2323	0.6299	0.885	0.538	1.455
H_DIAB01	1	1	0.28804	0.08685	10.9989	0.0009	1.334	1.125	1.581
H_AF01	1	1	0.39235	0.14557	7.2646	0.0070	1.480	1.113	1.969
H_HYPT01	1	1	-0.01669	0.08324	0.0402	0.8411	0.983	0.835	1.158
H_LIPID01	1	1	-0.11957	0.16078	0.5531	0.4571	0.887	0.647	1.216
AI	1	1	0.32795	0.20158	2.6470	0.1037	1.388	0.935	2.061
H_DRINK_H01	1	1	0.00691	0.14829	0.0022	0.9629	1.007	0.753	1.347
H_SMK_C01	1	1	0.07104	0.10881	0.4262	0.5138	1.074	0.867	1.329
IT	1	1	0.08331	0.12095	0.4744	0.4910	1.087	0.857	1.378
ET	1	1	0.47125	0.41591	1.2839	0.2572	1.602	0.709	3.620
IMG_C_TOAST	5	1	-0.42128	0.09354	20.2824	<.0001	0.656	0.546	0.788
IMG_C_TOAST	4	1	0.00612	0.36060	0.0003	0.9865	1.006	0.496	2.040
IMG_C_TOAST	3	1	-0.57107	0.12355	21.3645	<.0001	0.565	0.443	0.720
IMG_C_TOAST	2	1	-0.47467	0.17415	7.4295	0.0064	0.622	0.442	0.875
A_NIHSS		1	0.02496	0.00798	9.7895	0.0018	1.025	1.009	1.041

age2: BMI_g with y1_stroke: adjusted model

PHREG 过程

最大似然估计分析		
参数		标签
BMI_g	4	1=<18.5;2=18.5-<23;3=23-<27.5;4= ≥ 27.5 4
BMI_g	3	1=<18.5;2=18.5-<23;3=23-<27.5;4= ≥ 27.5 3
BMI_g	1	1=<18.5;2=18.5-<23;3=23-<27.5;4= ≥ 27.5 1
AGE		A.Basic Information: Age (years old);
GENDER		A.Basic Information: Gender; 1-male; 2-female;
ETHNIC	2	B.Demography: Race: 1-Han; 99-others; 2
H_DIAB01	1	D.History: Diabetes; 0-No; 1-Yes; 1
H_AF01	1	D.History: Heart disease category: Atrial fibrillation(Including medical history and hospitalization diagnosis); 0-No; 1-Yes; 1
H_HYPT01	1	D.History: Hypertension; 0-No; 1-Yes; 1
H_LIPID01	1	D.History: Lipid metabolism disorders; 0-No; 1-Yes; 1
AI	1	history:Myocardial infarction; 0=NO; 1=YES; 1
H_DRINK_H01	1	D.History: Heavy Drinking(Alcohol consumption>=20g/day); 0-No,1-Yes; 1
H_SMK_C01	1	D.History: Current Smoking; 0-No,1-Yes; 1
IT	1	intravenous thrombolysis, 1=YES,0=NO 1
ET	1	动脉溶栓或机械取栓, 1=YES,0=NO 1
IMG_C_TOAST	5	K.Final diagnosis: cerebral infarction; Etiology according to TOAST system; 1-large artery atherosclerosis; 2-cardiogenic embolism; 3-small artery occlusion; 4-stroke of another determined cause; 5-stroke of an undetermined cause. 5
IMG_C_TOAST	4	K.Final diagnosis: cerebral infarction; Etiology according to TOAST system; 1-large artery atherosclerosis; 2-cardiogenic embolism; 3-small artery occlusion; 4-stroke of another determined cause; 5-stroke of an undetermined cause. 4
IMG_C_TOAST	3	K.Final diagnosis: cerebral infarction; Etiology according to TOAST system; 1-large artery atherosclerosis; 2-cardiogenic embolism; 3-small artery occlusion; 4-stroke of another determined cause; 5-stroke of an undetermined cause. 3
IMG_C_TOAST	2	K.Final diagnosis: cerebral infarction; Etiology according to TOAST system; 1-large artery atherosclerosis; 2-cardiogenic embolism; 3-small artery occlusion; 4-stroke of another determined cause; 5-stroke of an undetermined cause. 2
A_NIHSS		F.Admitting NIHSS: Total score;

BMI_g with y1_stroke: interaction with age_group

PHREG 过程

模型信息		
数据集	WORK.DATA_OVERALL	
因变量	y1_stroke_dd	N12.Follow-up events at 12 months: Days from onset to recurrence;(day);
删失变量	y1_stroke	N12.Follow-up events at 12 months: Recurrence of stroke: 0-No; 1-Yes;
删失值	0	
结值处理	BRESLOW	

读取的观测数	14146
使用的观测数	14146

分类水平信息						
分类	值	设计变量				
BMI_g	4	1	0	0	
	3	0	1	0	
	2	0	0	0	
	1	0	0	1	
ETHNIC	2	1			
	1	0			
H_DIAB01	1	1			
	0	0			
H_AF01	1	1			
	0	0			
H_HYPT01	1	1			
	0	0			
H_LIPID01	1	1			
	0	0			
AI	1	1			
	0	0			
H_DRINK_H01	1	1			
	0	0			
H_SMK_C01	1	1			
	0	0			
IT	1	1			
	0	0			
ET	1	1			
	0	0			
IMG_C_TOAST	5	1	0	0	0
	4	0	1	0	0
	3	0	0	1	0
	2	0	0	0	1
	1	0	0	0	0

BMI_g with y1_stroke: interaction with age_group

PHREG 过程

事件和删失值个数汇总			
合计	事件	删失	删失 百分比
14146	1424	12722	89.93

收敛状态
满足收敛准则 (GCONV=1E-8)。

模型拟合统计量		
准则	无 协变量	带 协变量
-2 LOG L	27024.892	26885.374
AIC	27024.892	26931.374
SBC	27024.892	27052.382

检验全局原假设: BETA=0			
检验	卡方	自由度	Pr > 卡方
似然比	139.5185	23	<.0001
评分	148.8691	23	<.0001
Wald	145.6058	23	<.0001

联合检验			
效应	自由度	Wald 卡方	Pr > 卡方
BMI_g	3	2.1633	0.5392
age_group	1	0.0036	0.9524
age_group*BMI_g	3	2.3730	0.4987
GENDER	1	0.6475	0.4210
ETHNIC	1	0.6400	0.4237
H_DIAB01	1	12.3721	0.0004
H_AF01	1	10.3310	0.0013
H_HYPT01	1	1.8904	0.1692
H_LIPID01	1	0.6589	0.4169
AI	1	1.2035	0.2726
H_DRINK_H01	1	2.8726	0.0901
H_SMK_C01	1	1.3004	0.2541
IT	1	1.0157	0.3135
ET	1	5.2570	0.0219
IMG_C_TOAST	4	53.4902	<.0001
A_NIHSS	1	13.0395	0.0003

Note: Under full-rank parameterizations, Type 3 effect tests are replaced by joint tests. The joint test for an effect is a test that all of the parameters associated with that effect are zero. Such joint tests might not be equivalent to Type 3 effect tests under GLM parameterization.

BMI_g with y1_stroke: interaction with age_group

PHREG 过程

最大似然估计分析									
参数		自由度	参数估计	标准误差	卡方	Pr > 卡方	危险率	95% 危险率置信限	
BMI_g	4	1	-0.05820	0.24802	0.0551	0.8145	.	.	.
BMI_g	3	1	-0.26849	0.19965	1.8086	0.1787	.	.	.
BMI_g	1	1	-0.20841	0.68259	0.0932	0.7601	.	.	.
age_group		1	0.00619	0.10371	0.0036	0.9524	.	.	.
age_group*BMI_g	4	1	0.09381	0.16239	0.3337	0.5635	.	.	.
age_group*BMI_g	3	1	0.18963	0.12624	2.2565	0.1331	.	.	.
age_group*BMI_g	1	1	0.21575	0.38635	0.3118	0.5766	.	.	.
GENDER		1	0.05028	0.06248	0.6475	0.4210	1.052	0.930	1.189
ETHNIC	2	1	-0.13355	0.16694	0.6400	0.4237	0.875	0.631	1.214
H_DIAB01	1	1	0.21382	0.06079	12.3721	0.0004	1.238	1.099	1.395
H_AF01	1	1	0.39140	0.12177	10.3310	0.0013	1.479	1.165	1.878
H_HYPT01	1	1	0.07864	0.05719	1.8904	0.1692	1.082	0.967	1.210
H_LIPID01	1	1	-0.08323	0.10253	0.6589	0.4169	0.920	0.753	1.125
AI	1	1	0.18434	0.16803	1.2035	0.2726	1.202	0.865	1.671
H_DRINK_H01	1	1	0.14189	0.08372	2.8726	0.0901	1.152	0.978	1.358
H_SMK_C01	1	1	-0.07875	0.06906	1.3004	0.2541	0.924	0.807	1.058
IT	1	1	0.08376	0.08311	1.0157	0.3135	1.087	0.924	1.280
ET	1	1	0.59046	0.25752	5.2570	0.0219	1.805	1.089	2.990
IMG_C_TOAST	5	1	-0.34266	0.06301	29.5705	<.0001	0.710	0.627	0.803
IMG_C_TOAST	4	1	-0.06426	0.22873	0.0789	0.7788	0.938	0.599	1.468
IMG_C_TOAST	3	1	-0.53192	0.08092	43.2155	<.0001	0.587	0.501	0.688
IMG_C_TOAST	2	1	-0.44568	0.14230	9.8098	0.0017	0.640	0.485	0.846
A_NIHSS		1	0.02141	0.00593	13.0395	0.0003	1.022	1.010	1.034

BMI_g with y1_stroke: interaction with age_group

PHREG 过程

最大似然估计分析		
参数		标签
BMI_g	4	1=<18.5;2=18.5-<23;3=23-<27.5;4=≥27.5 4
BMI_g	3	1=<18.5;2=18.5-<23;3=23-<27.5;4=≥27.5 3
BMI_g	1	1=<18.5;2=18.5-<23;3=23-<27.5;4=≥27.5 1
age_group		1=age<65;2=age>=65
age_group*BMI_g	4	1=<18.5;2=18.5-<23;3=23-<27.5;4=≥27.5 4 * 1=age<65;2=age>=65
age_group*BMI_g	3	1=<18.5;2=18.5-<23;3=23-<27.5;4=≥27.5 3 * 1=age<65;2=age>=65
age_group*BMI_g	1	1=<18.5;2=18.5-<23;3=23-<27.5;4=≥27.5 1 * 1=age<65;2=age>=65
GENDER		A.Basic Information: Gender; 1-male; 2-female;
ETHNIC	2	B.Demography: Race: 1-Han; 99-others; 2
H_DIAB01	1	D.History: Diabetes; 0-No; 1-Yes; 1
H_AF01	1	D.History: Heart disease category: Atrial fibrillation(Including medical history and hospitalization diagnosis); 0-No; 1-Yes; 1
H_HYPT01	1	D.History: Hypertension; 0-No; 1-Yes; 1
H_LIPID01	1	D.History: Lipid metabolism disorders; 0-No; 1-Yes; 1
AI	1	history:Myocardial infarction; 0=NO; 1=YES; 1
H_DRINK_H01	1	D.History: Heavy Drinking(Alcohol consumption>=20g/day); 0-No,1-Yes; 1
H_SMK_C01	1	D.History: Current Smoking; 0-No,1-Yes; 1
IT	1	intravenous thrombolysis, 1=YES,0=NO 1
ET	1	动脉溶栓或机械取栓, 1=YES,0=NO 1
IMG_C_TOAST	5	K.Final diagnosis: cerebral infarction; Etiology according to TOAST system; 1-large artery atherosclerosis; 2-cardiogenic embolism; 3-small artery occlusion; 4-stroke of another determined cause; 5-stroke of an undetermined cause. 5
IMG_C_TOAST	4	K.Final diagnosis: cerebral infarction; Etiology according to TOAST system; 1-large artery atherosclerosis; 2-cardiogenic embolism; 3-small artery occlusion; 4-stroke of another determined cause; 5-stroke of an undetermined cause. 4
IMG_C_TOAST	3	K.Final diagnosis: cerebral infarction; Etiology according to TOAST system; 1-large artery atherosclerosis; 2-cardiogenic embolism; 3-small artery occlusion; 4-stroke of another determined cause; 5-stroke of an undetermined cause. 3
IMG_C_TOAST	2	K.Final diagnosis: cerebral infarction; Etiology according to TOAST system; 1-large artery atherosclerosis; 2-cardiogenic embolism; 3-small artery occlusion; 4-stroke of another determined cause; 5-stroke of an undetermined cause. 2
A_NIHSS		F.Admitting NIHSS: Total score;
