# Supplementary material for: Association of body mass index and waist-to-height ratio with outcomes in ischemic stroke: results from the Third China National Stroke Registry
Source: BMC Neurol. 2023 Apr 14;23:152. doi: 10.1186/s12883-023-03165-y (PMC10103413; doi:10.1186/s12883-023-03165-y)
Supplement: Supplementary file 2 — Additional file 2. [file 12883_2023_3165_MOESM2_ESM.zip › raw data/Table-s1.pdf]

FREQ 过程

1:有腰围数据；0：腰围缺失				
EXCLUDE	频数	百分比	累积频数	累积百分比
0	9341	66.03	9341	66.03
1	4805	33.97	14146	100.00

MEANS PROCEDURE

变量	标签	数目	缺失值个数	均值	标准差	最小值	下四分位数	中位数	上四分位数	最大值
AGE	A.Basic Information: Age (years old);	14146	0	62.3126679	11.2954852	19.0000000	54.0000000	63.0000000	70.0000000	96.0000000
BMI	F.Physical examination: Body mass index (kg/m2);	14146	0	24.6865455	3.3248886	13.8410000	22.5980000	24.4900000	26.5630000	51.0730000
A_NIHSS	F.Admitting NIHSS: Total score;	14146	0	4.4148876	4.1781470	0	2.0000000	3.0000000	6.0000000	40.0000000

FREQ 过程

A.Basic Information: Gender; 1-male; 2-female;				
GENDER	频数	百分比	累积频数	累积百分比
1	9720	68.71	9720	68.71
2	4426	31.29	14146	100.00

B.Demography: Race: 1-Han; 99-others;				
ETHNIC	频数	百分比	累积频数	累积百分比
1	13730	97.06	13730	97.06
2	416	2.94	14146	100.00

D.History: Stroke History; 0-No; 1-Yes;				
H_STROKE01	频数	百分比	累积频数	累积百分比
0	11012	77.85	11012	77.85
1	3134	22.15	14146	100.00

D.History: Diabetes; 0-No; 1-Yes;				
H_DIAB01	频数	百分比	累积频数	累积百分比
0	10836	76.60	10836	76.60
1	3310	23.40	14146	100.00

D.History: Heart disease category: Atrial fibrillation(Including medical history and hospitalization diagnosis); 0-No; 1-Yes;				
H_AF01	频数	百分比	累积频数	累积百分比
0	13160	93.03	13160	93.03
1	986	6.97	14146	100.00

history:Myocardial infarction; 0=NO; 1=YES;				
AI	频数	百分比	累积频数	累积百分比
0	13868	98.03	13868	98.03
1	278	1.97	14146	100.00

D.History: Hypertension; 0-No; 1-Yes;				
H_HYPT01	频数	百分比	累积频数	累积百分比
0	5259	37.18	5259	37.18
1	8887	62.82	14146	100.00

FREQ 过程

D.History: Lipid metabolism disorders; 0-No; 1-Yes;				
H_LIPID01	频数	百分比	累积频数	累积百分比
0	13071	92.40	13071	92.40
1	1075	7.60	14146	100.00

D.History: Heavy Drinking(Alcohol consumption>=20g/day); 0-No,1-Yes;				
H_DRINK_H01	频数	百分比	累积频数	累积百分比
0	12136	85.79	12136	85.79
1	2010	14.21	14146	100.00

D.History: Current Smoking; 0-No,1-Yes;				
H_SMK_C01	频数	百分比	累积频数	累积百分比
0	9643	68.17	9643	68.17
1	4503	31.83	14146	100.00

intravenous thrombolysis, 1=YES,0=NO				
IT	频数	百分比	累积频数	累积百分比
0	12626	89.25	12626	89.25
1	1520	10.75	14146	100.00

动脉溶栓或机械取栓, 1=YES,0=NO				
ET	频数	百分比	累积频数	累积百分比
0	14075	99.50	14075	99.50
1	71	0.50	14146	100.00

K.Final diagnosis: cerebral infarction; Etiology according to TOAST system; 1-large artery atherosclerosis; 2-cardiogenic embolism; 3-small artery occlusion; 4-stroke of another determined cause; 5-stroke of an undetermined cause.				
IMG_C_TOAST	频数	百分比	累积频数	累积百分比
1	3667	25.92	3667	25.92
2	881	6.23	4548	32.15
3	3137	22.18	7685	54.33
4	171	1.21	7856	55.54
5	6290	44.46	14146	100.00

FREQ 过程

N12.Follow-up events at 12 months: Recurrence of stroke: 0-No; 1-Yes;				
y1_stroke	频数	百分比	累积频数	累积百分比
0	12722	89.93	12722	89.93
1	1424	10.07	14146	100.00

N12.Follow-up events at 12 months: recurrence of ischemic stroke: 0-No; 1-Yes;				
y1_is	频数	百分比	累积频数	累积百分比
0	12829	90.69	12829	90.69
1	1317	9.31	14146	100.00

N12.Follow-up events at 12 months: recurrence of hemorrhage stroke: 0-No; 1-Yes;				
y1_HS	频数	百分比	累积频数	累积百分比
0	14022	99.12	14022	99.12
1	124	0.88	14146	100.00

I.Inpatient Event: Hemorrhagic transformation after cerebral infarction; 1-No; 2-Yes; 98-UK;				
I_IS_HT	频数	百分比	累积频数	累积百分比
.	86	0.61	86	0.61
1	13867	98.03	13953	98.64
2	193	1.36	14146	100.00

1年心血管源性死亡，0=NO; 1=YES				
death_cvd	频数	百分比	累积频数	累积百分比
0	13949	98.61	13949	98.61
1	197	1.39	14146	100.00

N12.Follow-up events at 12 months:Occurrence of combined vascular event(including cardiovascular death,non-fatal stroke,non-fatal myocardial infarction):0-No;1-Yes;				
y1_comb	频数	百分比	累积频数	累积百分比
0	12641	89.36	12641	89.36
1	1505	10.64	14146	100.00

N12.Follow-up events at 12 months: Whether the patient died: 0-survival;1-death;				
y1_death	频数	百分比	累积频数	累积百分比
0	13660	96.56	13660	96.56
1	486	3.44	14146	100.00

continuous variables, descriptive by group

MEANS PROCEDURE

1:有腰围数据；0：腰围缺失	观测数	变量	标签	数目	缺失值个数	均值	标准差	最小值	下四分位数
0	9341	AGE<br>BMI<br>A_NIHSS	A.Basic Information: Age (years old);<br>F.Physical examination: Body mass index (kg/m2);<br>F.Admitting NIHSS: Total score;	9341<br>9341<br>9341	0<br>0<br>0	61.7290440<br>24.7910738<br>4.4082004	11.2139559<br>3.2661988<br>4.1771343	19.0000000<br>13.8410000<br>0	54.0000000<br>22.8370000<br>2.0000000
1	4805	AGE<br>BMI<br>A_NIHSS	A.Basic Information: Age (years old);<br>F.Physical examination: Body mass index (kg/m2);<br>F.Admitting NIHSS: Total score;	4805<br>4805<br>4805	0<br>0<br>0	63.4472425<br>24.4833409<br>4.4278876	11.3680560<br>3.4273485<br>4.1805192	23.0000000<br>13.8410000<br>0	56.0000000<br>22.3130000<br>2.0000000

1:有腰围数据；0：腰围缺失	观测数	变量	标签	中位数	上四分位数	最大值
0	9341	AGE<br>BMI<br>A_NIHSS	A.Basic Information: Age (years old);<br>F.Physical examination: Body mass index (kg/m2);<br>F.Admitting NIHSS: Total score;	62.0000000<br>24.4900000<br>3.0000000	70.0000000<br>26.5730000<br>6.0000000	96.0000000<br>50.5990000<br>38.0000000
1	4805	AGE<br>BMI<br>A_NIHSS	A.Basic Information: Age (years old);<br>F.Physical examination: Body mass index (kg/m2);<br>F.Admitting NIHSS: Total score;	64.0000000<br>24.2210000<br>3.0000000	72.0000000<br>26.3700000<br>6.0000000	93.0000000<br>51.0730000<br>40.0000000

Kruskal-Wallis Test among differenct group

NPAR1WAY 过程

变量“AGE”的 Wilcoxon 评分（秩和）<br>按变量“EXCLUDE”分类					
EXCLUDE	数目	评分汇总	H0 之下的期望值	H0 之下的标准差	均值评分
1	4805	35958473.0	33988167.5	229946.665	7483.55317
0	9341	64103258.0	66073563.5	229946.665	6862.56910
已将平均评分用于结值。					

Wilcoxon 双样本检验					
				t 近似值	
统计量	Z	Pr > Z	Pr > |Z|	Pr > Z	Pr > |Z|
35958473	8.5685	<.0001	<.0001	<.0001	<.0001
Z 包括 0.5 的连续性校正。					

Kruskal-Wallis 检验		
卡方	自由度	Pr > 卡方
73.4198	1	<.0001

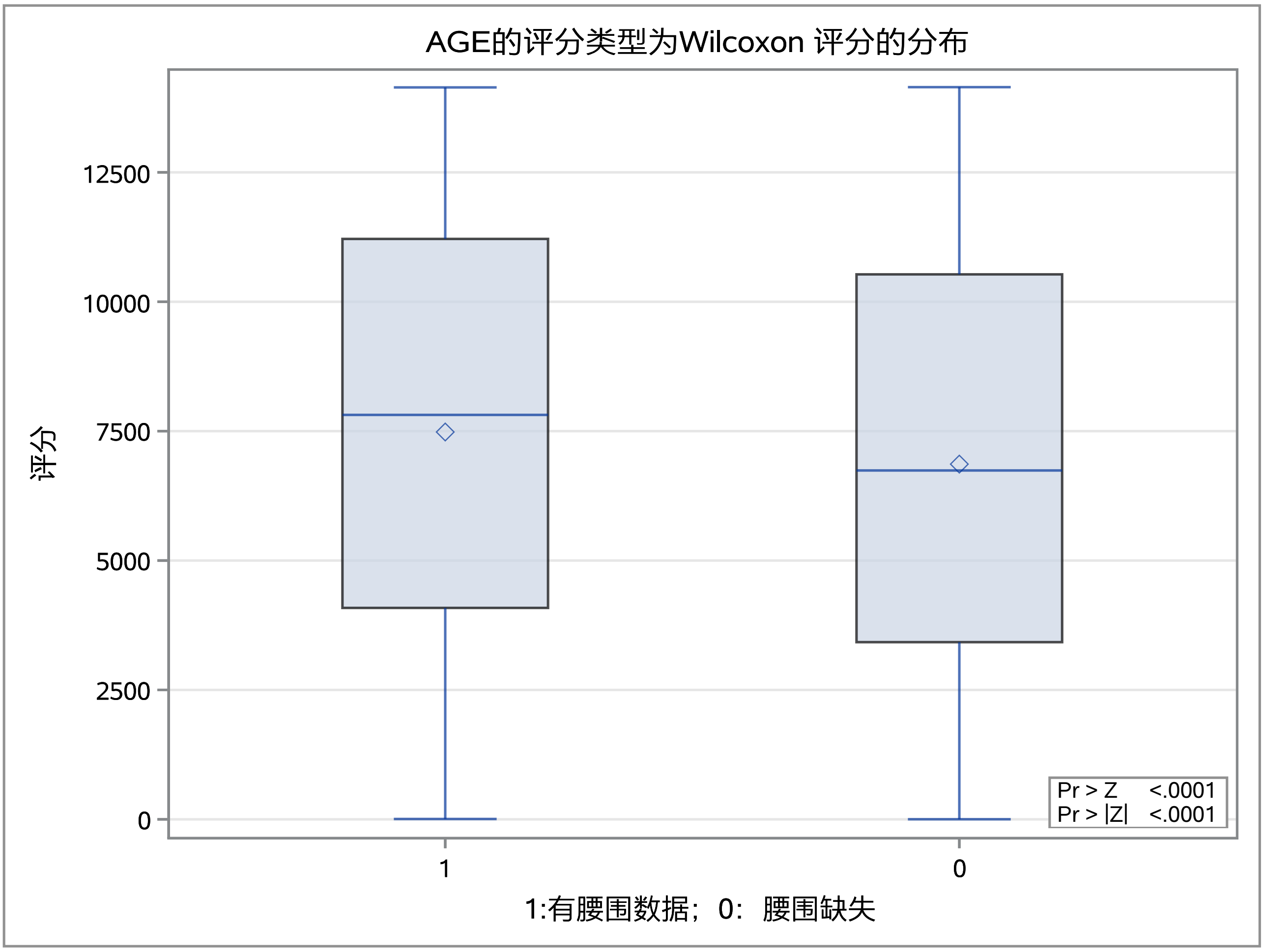

Kruskal-Wallis Test among differenct group

NPAR1WAY 过程

变量 “BMI” 的 Wilcoxon 评分（秩和）按变量 “EXCLUDE” 分类					
EXCLUDE	数目	评分汇总	H0 之下的期望值	H0 之下的标准差	均值评分
1	4805	32691217.5	33988167.5	230021.210	6803.58325
0	9341	67370513.5	66073563.5	230021.210	7212.34488
已将平均评分用于结值。					

Wilcoxon 双样本检验					
				t 近似值	
统计量	Z	Pr < Z	Pr > |Z|	Pr < Z	Pr > |Z|
32691218	-5.6384	<.0001	<.0001	<.0001	<.0001
Z 包括 0.5 的连续性校正。					

Kruskal-Wallis 检验		
卡方	自由度	Pr > 卡方
31.7915	1	<.0001

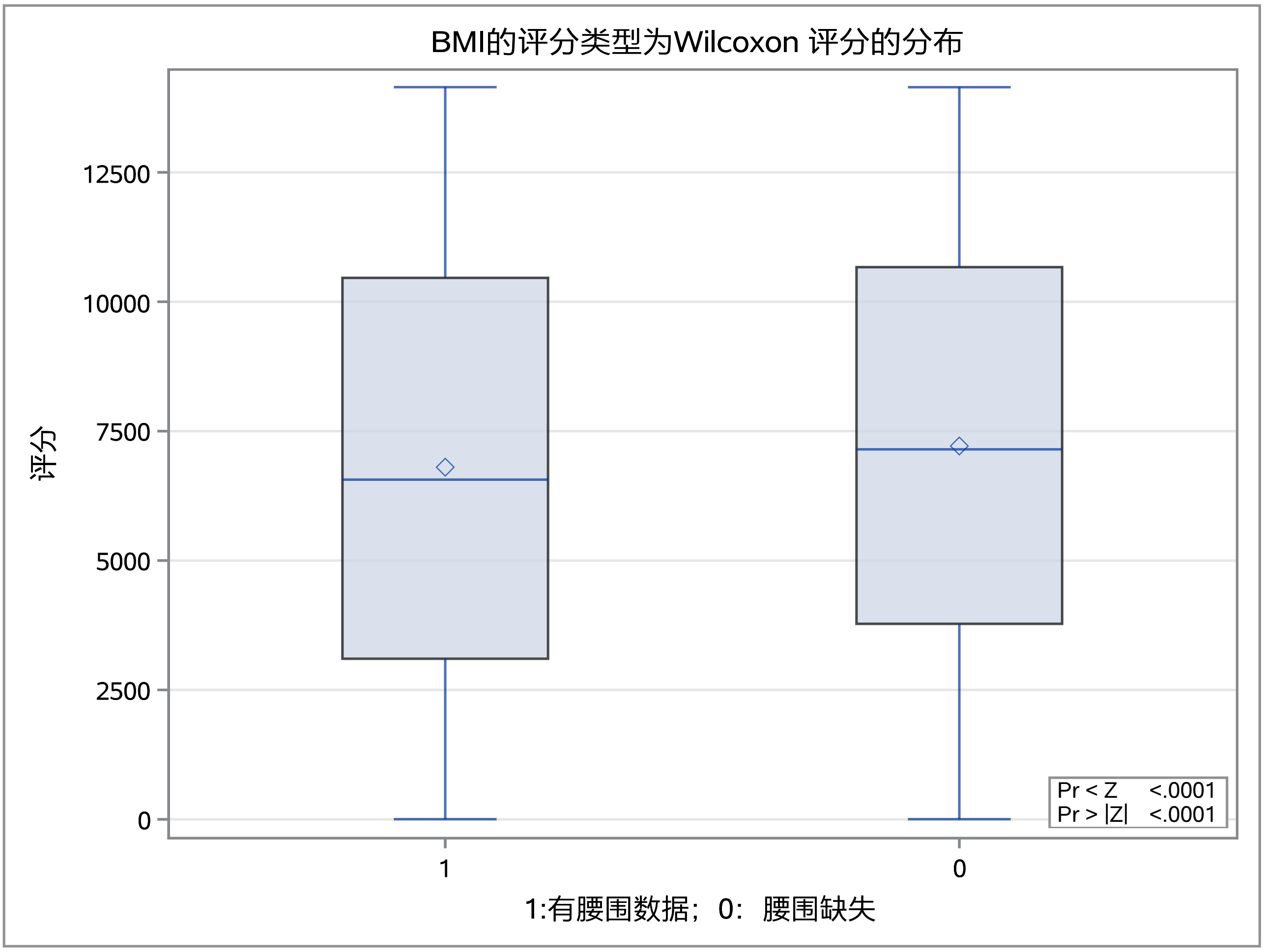

Kruskal-Wallis Test among differenct group

NPAR1WAY 过程

变量 “A_NIHSS” 的 Wilcoxon 评分（秩和）按变量 “EXCLUDE” 分类					
EXCLUDE	数目	评分汇总	H0 之下的期望值	H0 之下的标准差	均值评分
1	4805	34065579.5	33988167.5	228672.505	7089.61072
0	9341	65996151.5	66073563.5	228672.505	7065.21266
已将平均评分用于结值。					

Wilcoxon 双样本检验					
				t 近似值	
统计量	Z	Pr > Z	Pr > |Z|	Pr > Z	Pr > |Z|
34065580	0.3385	0.3675	0.7350	0.3675	0.7350
Z 包括 0.5 的连续性校正。					

Kruskal-Wallis 检验		
卡方	自由度	Pr > 卡方
0.1146	1	0.7350

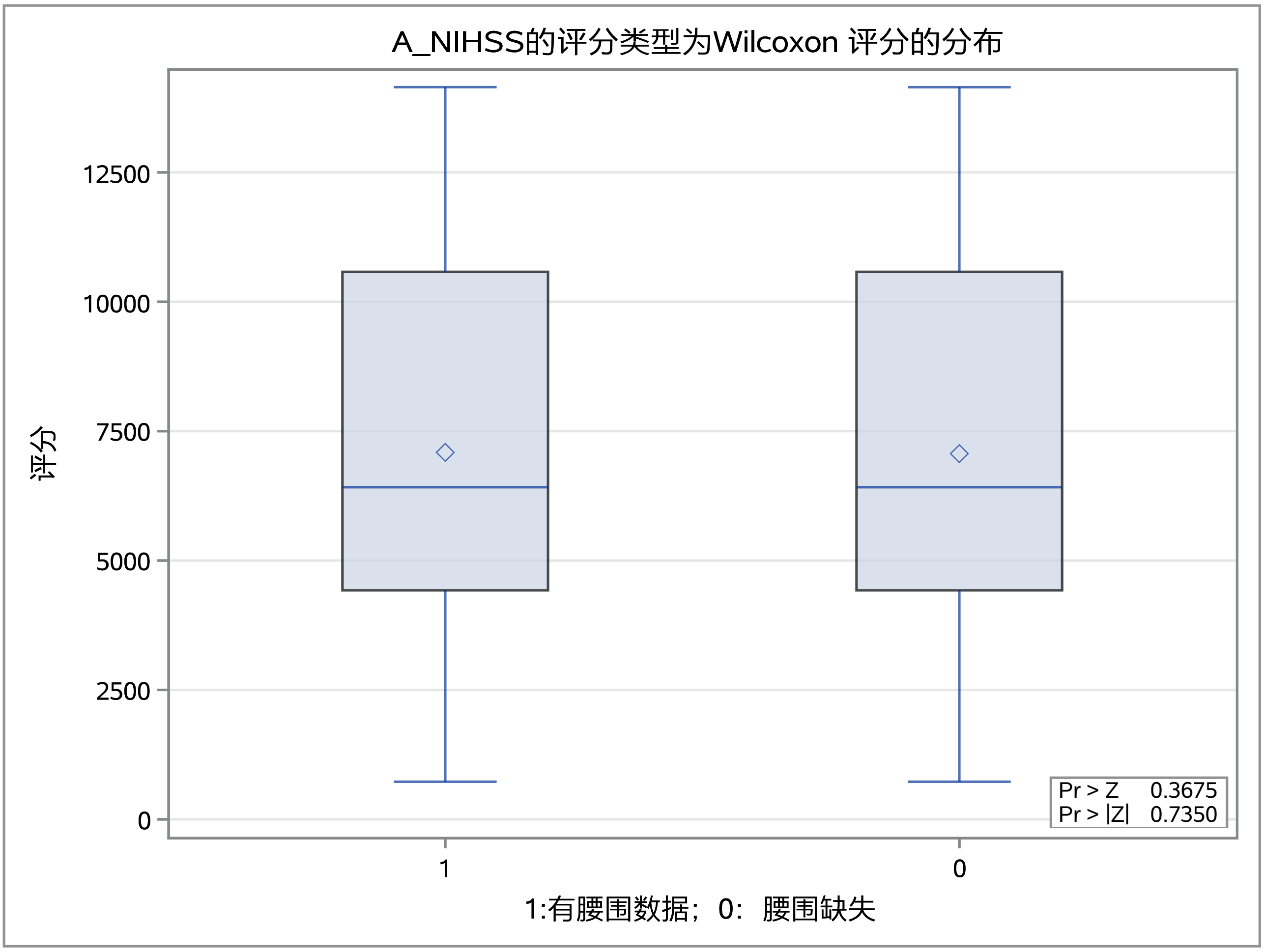

categorical variables, descriptive by group and chisq test

FREQ 过程

频数<br>列百分比	GENDER-EXCLUDE表		
GENDER(A.Basic Information: Gender; 1-male; 2-female;)	EXCLUDE(1:有腰围数据；0：腰围缺失)		
	0	1	合计
1	6506<br>69.65	3214<br>66.89	9720
2	2835<br>30.35	1591<br>33.11	4426
合计	9341	4805	14146

表“EXCLUDE-GENDER”的统计量

统计量	自由度	值	概率
卡方	1	11.2528	0.0008
似然比卡方检验	1	11.1985	0.0008
连续调整卡方	1	11.1247	0.0009
Mantel-Haenszel 卡方	1	11.2520	0.0008
Phi 系数		0.0282	
列联系数		0.0282	
Cramer V		0.0282	

Fisher 的精确检验	
单元格 (1,1) 频数 (F)	6506
左侧 Pr <= F	0.9996
右侧 Pr >= F	0.0004
表概率 (P)	<.0001
双侧 Pr <= P	0.0009

样本大小 = 14146

频数<br>列百分比	ETHNIC-EXCLUDE表		
ETHNIC(B.Demography: Race: 1-Han; 99-others;)	EXCLUDE(1:有腰围数据；0：腰围缺失)		
	0	1	合计
1	9147<br>97.92	4583<br>95.38	13730
2	194<br>2.08	222<br>4.62	416
合计	9341	4805	14146

categorical variables, descriptive by group and chisq test

FREQ 过程

表“EXCLUDE-ETHNIC”的统计量

统计量	自由度	值	概率
卡方	1	71.9050	<.0001
似然比卡方检验	1	67.7554	<.0001
连续调整卡方	1	71.0167	<.0001
Mantel-Haenszel 卡方	1	71.8999	<.0001
Phi 系数		0.0713	
列联系数		0.0711	
Cramer V		0.0713	

Fisher 的精确检验	
单元格 (1,1) 频数 (F)	9147
左侧 Pr <= F	1.0000
右侧 Pr >= F	<.0001
表概率 (P)	<.0001
双侧 Pr <= P	<.0001

样本大小 = 14146

频数<br>列百分比

H_STROKE01-EXCLUDE表			
H_STROKE01(D.History: Stroke History; 0-No; 1-Yes;)	EXCLUDE(1:有腰围数据；0: 腰围缺失)		
	0	1	合计
0	7139<br>76.43	3873<br>80.60	11012
1	2202<br>23.57	932<br>19.40	3134
合计	9341	4805	14146

categorical variables, descriptive by group and chisq test

FREQ 过程

表“EXCLUDE-H_STROKE01”的统计量

统计量	自由度	值	概率
卡方	1	32.0989	<.0001
似然比卡方检验	1	32.6145	<.0001
连续调整卡方	1	31.8572	<.0001
Mantel-Haenszel 卡方	1	32.0967	<.0001
Phi 系数		-0.0476	
列联系数		0.0476	
Cramer V		-0.0476	

Fisher 的精确检验	
单元格 (1,1) 频数 (F)	7139
左侧 Pr <= F	<.0001
右侧 Pr >= F	1.0000
表概率 (P)	<.0001
双侧 Pr <= P	<.0001

样本大小 = 14146

频数
列百分比

H_DIAB01-EXCLUDE表			
H_DIAB01(D.History: Diabetes; 0-No; 1-Yes;)	EXCLUDE(1:有腰围数据；0：腰围缺失)		
	0	1	合计
0	7138<br>76.42	3698<br>76.96	10836
1	2203<br>23.58	1107<br>23.04	3310
合计	9341	4805	14146

categorical variables, descriptive by group and chisq test

FREQ 过程

表“EXCLUDE-H_DIAB01”的统计量

统计量	自由度	值	概率
卡方	1	0.5271	0.4678
似然比卡方检验	1	0.5281	0.4674
连续调整卡方	1	0.4971	0.4808
Mantel-Haenszel 卡方	1	0.5271	0.4678
Phi 系数		-0.0061	
列联系数		0.0061	
Cramer V		-0.0061	

Fisher 的精确检验	
单元格 (1,1) 频数 (F)	7138
左侧 Pr <= F	0.2406
右侧 Pr >= F	0.7723
表概率 (P)	0.0129
双侧 Pr <= P	0.4760

样本大小 = 14146

频数
列百分比

H_AF01-EXCLUDE表			
H_AF01(D.History: Heart disease category: Atrial fibrillation(Including medical history and hospitalization diagnosis); 0-No; 1-Yes;)	EXCLUDE(1:有腰围数据；0：腰围缺失)		
	0	1	合计
0	8739<br>93.56	4421<br>92.01	13160
1	602<br>6.44	384<br>7.99	986
合计	9341	4805	14146

categorical variables, descriptive by group and chisq test

FREQ 过程

表“EXCLUDE-H_AF01”的统计量

统计量	自由度	值	概率
卡方	1	11.7098	0.0006
似然比卡方检验	1	11.4802	0.0007
连续调整卡方	1	11.4725	0.0007
Mantel-Haenszel 卡方	1	11.7090	0.0006
Phi 系数		0.0288	
列联系数		0.0288	
Cramer V		0.0288	

Fisher 的精确检验	
单元格 (1,1) 频数 (F)	8739
左侧 Pr <= F	0.9997
右侧 Pr >= F	0.0004
表概率 (P)	<.0001
双侧 Pr <= P	0.0007

样本大小 = 14146

频数
列百分比

AI-EXCLUDE表			
AI(history:Myocardial infarction; 0=NO; 1=YES;)	EXCLUDE(1:有腰围数据；0：腰围缺失)		
	0	1	合计
0	9136<br>97.81	4732<br>98.48	13868
1	205<br>2.19	73<br>1.52	278
合计	9341	4805	14146

categorical variables, descriptive by group and chisq test

FREQ 过程

表“EXCLUDE-AI”的统计量

统计量	自由度	值	概率
卡方	1	7.5119	0.0061
似然比卡方检验	1	7.8361	0.0051
连续调整卡方	1	7.1655	0.0074
Mantel-Haenszel 卡方	1	7.5114	0.0061
Phi 系数		-0.0230	
列联系数		0.0230	
Cramer V		-0.0230	

Fisher 的精确检验	
单元格 (1,1) 频数 (F)	9136
左侧 Pr <= F	0.0032
右侧 Pr >= F	0.9979
表概率 (P)	0.0011
双侧 Pr <= P	0.0059

样本大小 = 14146

频数<br>列百分比

H_HYPT01-EXCLUDE表			
H_HYPT01(D.History: Hypertension; 0-No; 1-Yes;)	EXCLUDE(1:有腰围数据；0：腰围缺失)		
	0	1	合计
0	3561<br>38.12	1698<br>35.34	5259
1	5780<br>61.88	3107<br>64.66	8887
合计	9341	4805	14146

categorical variables, descriptive by group and chisq test

FREQ 过程

表“EXCLUDE-H_HYPT01”的统计量

统计量	自由度	值	概率
卡方	1	10.5298	0.0012
似然比卡方检验	1	10.5667	0.0012
连续调整卡方	1	10.4110	0.0013
Mantel-Haenszel 卡方	1	10.5291	0.0012
Phi 系数		0.0273	
列联系数		0.0273	
Cramer V		0.0273	

Fisher 的精确检验	
单元格 (1,1) 频数 (F)	3561
左侧 Pr <= F	0.9995
右侧 Pr >= F	0.0006
表概率 (P)	<.0001
双侧 Pr <= P	0.0012

样本大小 = 14146

频数
列百分比

H_LIPID01-EXCLUDE表			
H_LIPID01(D.History: Lipid metabolism disorders; 0-No; 1-Yes;)	EXCLUDE(1:有腰围数据；0: 腰围缺失)		
	0	1	合计
0	8596<br>92.02	4475<br>93.13	13071
1	745<br>7.98	330<br>6.87	1075
合计	9341	4805	14146

categorical variables, descriptive by group and chisq test

FREQ 过程

表“EXCLUDE-H_LIPID01”的统计量

统计量	自由度	值	概率
卡方	1	5.5448	0.0185
似然比卡方检验	1	5.6303	0.0177
连续调整卡方	1	5.3881	0.0203
Mantel-Haenszel 卡方	1	5.5444	0.0185
Phi 系数		-0.0198	
列联系数		0.0198	
Cramer V		-0.0198	

Fisher 的精确检验	
单元格 (1,1) 频数 (F)	8596
左侧 Pr <= F	0.0098
右侧 Pr >= F	0.9919
表概率 (P)	0.0016
双侧 Pr <= P	0.0190

样本大小 = 14146

频数<br>列百分比

H_DRINK_H01-EXCLUDE表			
H_DRINK_H01(D.History: Heavy Drinking(Alcohol consumption>=20g/day); 0-No,1-Yes;)	EXCLUDE(1:有腰围数据；0：腰围缺失)		
	0	1	合计
0	7923<br>84.82	4213<br>87.68	12136
1	1418<br>15.18	592<br>12.32	2010
合计	9341	4805	14146

categorical variables, descriptive by group and chisq test

FREQ 过程

表“EXCLUDE-H_DRINK_H01”的统计量

统计量	自由度	值	概率
卡方	1	21.2886	<.0001
似然比卡方检验	1	21.7142	<.0001
连续调整卡方	1	21.0546	<.0001
Mantel-Haenszel 卡方	1	21.2871	<.0001
Phi 系数		-0.0388	
列联系数		0.0388	
Cramer V		-0.0388	

Fisher 的精确检验	
单元格 (1,1) 频数 (F)	7923
左侧 Pr <= F	<.0001
右侧 Pr >= F	1.0000
表概率 (P)	<.0001
双侧 Pr <= P	<.0001

样本大小 = 14146

频数
列百分比

H_SMK_C01-EXCLUDE表			
H_SMK_C01(D.History: Current Smoking; 0-No,1-Yes;)	EXCLUDE(1:有腰围数据；0：腰围缺失)		
	0	1	合计
0	6227<br>66.66	3416<br>71.09	9643
1	3114<br>33.34	1389<br>28.91	4503
合计	9341	4805	14146

categorical variables, descriptive by group and chisq test

FREQ 过程

表“EXCLUDE-H_SMK_C01”的统计量

统计量	自由度	值	概率
卡方	1	28.6892	<.0001
似然比卡方检验	1	28.9403	<.0001
连续调整卡方	1	28.4854	<.0001
Mantel-Haenszel 卡方	1	28.6872	<.0001
Phi 系数		-0.0450	
列联系数		0.0450	
Cramer V		-0.0450	

Fisher 的精确检验	
单元格 (1,1) 频数 (F)	6227
左侧 Pr <= F	<.0001
右侧 Pr >= F	1.0000
表概率 (P)	<.0001
双侧 Pr <= P	<.0001

样本大小 = 14146

频数
列百分比

IT-EXCLUDE表			
IT(intravenous thrombolysis, 1=YES,0=NO)	EXCLUDE(1:有腰围数据；0：腰围缺失)		
	0	1	合计
0	8357<br>89.47	4269<br>88.84	12626
1	984<br>10.53	536<br>11.16	1520
合计	9341	4805	14146

categorical variables, descriptive by group and chisq test

FREQ 过程

表“EXCLUDE-IT”的统计量

统计量	自由度	值	概率
卡方	1	1.2752	0.2588
似然比卡方检验	1	1.2685	0.2601
连续调整卡方	1	1.2113	0.2711
Mantel-Haenszel 卡方	1	1.2751	0.2588
Phi 系数		0.0095	
列联系数		0.0095	
Cramer V		0.0095	

Fisher 的精确检验	
单元格 (1,1) 频数 (F)	8357
左侧 Pr <= F	0.8764
右侧 Pr >= F	0.1356
表概率 (P)	0.0120
双侧 Pr <= P	0.2637

样本大小 = 14146

频数
列百分比

ET-EXCLUDE表			
	EXCLUDE(1:有腰围数据；0: 腰围缺失)		
ET(动脉溶栓或机械取栓, 1=YES,0=NO)	0	1	合计
0	9298<br>99.54	4777<br>99.42	14075
1	43<br>0.46	28<br>0.58	71
合计	9341	4805	14146

categorical variables, descriptive by group and chisq test

FREQ 过程

表“EXCLUDE-ET”的统计量

统计量	自由度	值	概率
卡方	1	0.9517	0.3293
似然比卡方检验	1	0.9299	0.3349
连续调整卡方	1	0.7224	0.3954
Mantel-Haenszel 卡方	1	0.9516	0.3293
Phi 系数		0.0082	
列联系数		0.0082	
Cramer V		0.0082	

Fisher 的精确检验	
单元格 (1,1) 频数 (F)	9298
左侧 Pr <= F	0.8641
右侧 Pr >= F	0.1967
表概率 (P)	0.0608
双侧 Pr <= P	0.3791

样本大小 = 14146

频数
列百分比

IMG_C_TOAST-EXCLUDE表			
IMG_C_TOAST(K.Final diagnosis: cerebral infarction; Etiology according to TOAST system; 1-large artery atherosclerosis; 2-cardiogenic embolism; 3-small artery occlusion; 4-stroke of another determined cause; 5-stroke of an undetermined cause.)	EXCLUDE(1:有腰围数据；0: 腰围缺失)		
	0	1	合计
1	2443<br>26.15	1224<br>25.47	3667
2	540<br>5.78	341<br>7.10	881
3	2089<br>22.36	1048<br>21.81	3137
4	94<br>1.01	77<br>1.60	171
5	4175<br>44.70	2115<br>44.02	6290
合计	9341	4805	14146

categorical variables, descriptive by group and chisq test

FREQ 过程

表“EXCLUDE-IMG_C_TOAST”的统计量

统计量	自由度	值	概率
卡方	4	19.4824	0.0006
似然比卡方检验	4	18.9228	0.0008
Mantel-Haenszel 卡方	1	0.0585	0.8088
Phi 系数		0.0371	
列联系数		0.0371	
Cramer V		0.0371	

样本大小 = 14146

频数
列百分比

y1_stroke-EXCLUDE表			
y1_stroke(N12.Follow-up events at 12 months: Recurrence of stroke: 0-No; 1-Yes;)	EXCLUDE(1:有腰围数据；0: 腰围缺失)		
	0	1	合计
0	8406<br>89.99	4316<br>89.82	12722
1	935<br>10.01	489<br>10.18	1424
合计	9341	4805	14146

表“EXCLUDE-y1_stroke”的统计量

统计量	自由度	值	概率
卡方	1	0.0981	0.7542
似然比卡方检验	1	0.0979	0.7544
连续调整卡方	1	0.0804	0.7767
Mantel-Haenszel 卡方	1	0.0980	0.7542
Phi 系数		0.0026	
列联系数		0.0026	
Cramer V		0.0026	

Fisher 的精确检验	
单元格 (1,1) 频数 (F)	8406
左侧 Pr <= F	0.6349
右侧 Pr >= F	0.3875
表概率 (P)	0.0224
双侧 Pr <= P	0.7680

样本大小 = 14146

categorical variables, descriptive by group and chisq test

FREQ 过程

频数<br>列百分比

y1_is-EXCLUDE表			
y1_is(N12.Follow-up events at 12 months: recurrence of ischemic stroke: 0-No; 1-Yes;)	EXCLUDE(1:有腰围数据；0：腰围缺失)		
	0	1	合计
0	8469<br>90.66	4360<br>90.74	12829
1	872<br>9.34	445<br>9.26	1317
合计	9341	4805	14146

表“EXCLUDE-y1_is”的统计量

统计量	自由度	值	概率
卡方	1	0.0206	0.8859
似然比卡方检验	1	0.0206	0.8859
连续调整卡方	1	0.0127	0.9101
Mantel-Haenszel 卡方	1	0.0206	0.8859
Phi 系数		-0.0012	
列联系数		0.0012	
Cramer V		-0.0012	

Fisher 的精确检验	
单元格 (1,1) 频数 (F)	8469
左侧 Pr <= F	0.4561
右侧 Pr >= F	0.5681
表概率 (P)	0.0241
双侧 Pr <= P	0.9028

样本大小 = 14146

频数<br>列百分比

y1_HS-EXCLUDE表			
y1_HS(N12.Follow-up events at 12 months: recurrence of hemorrhage stroke: 0-No; 1-Yes;)	EXCLUDE(1:有腰围数据；0：腰围缺失)		
	0	1	合计
0	9267<br>99.21	4755<br>98.96	14022
1	74<br>0.79	50<br>1.04	124
合计	9341	4805	14146

categorical variables, descriptive by group and chisq test

FREQ 过程

表“EXCLUDE-y1_HS”的统计量

统计量	自由度	值	概率
卡方	1	2.2527	0.1334
似然比卡方检验	1	2.1941	0.1385
连续调整卡方	1	1.9759	0.1598
Mantel-Haenszel 卡方	1	2.2526	0.1334
Phi 系数		0.0126	
列联系数		0.0126	
Cramer V		0.0126	

Fisher 的精确检验	
单元格 (1,1) 频数 (F)	9267
左侧 Pr <= F	0.9433
右侧 Pr >= F	0.0812
表概率 (P)	0.0244
双侧 Pr <= P	0.1528

样本大小 = 14146

频数
列百分比

I_IS_HT-EXCLUDE表			
I_IS_HT(I.Inpatient Event: Hemorrhagic transformation after cerebral infarction; 1-No; 2-Yes; 98-UK;)	EXCLUDE(1:有腰围数据；0：腰围缺失)		
	0	1	合计
1	9208<br>98.81	4659<br>98.27	13867
2	111<br>1.19	82<br>1.73	193
合计	9319	4741	14060
频数缺失 = 86			

categorical variables, descriptive by group and chisq test

FREQ 过程

表“EXCLUDE-I_IS_HT”的统计量

统计量	自由度	值	概率
卡方	1	6.7301	0.0095
似然比卡方检验	1	6.4976	0.0108
连续调整卡方	1	6.3382	0.0118
Mantel-Haenszel 卡方	1	6.7296	0.0095
Phi 系数		0.0219	
列联系数		0.0219	
Cramer V		0.0219	

Fisher 的精确检验	
单元格 (1,1) 频数 (F)	9208
左侧 Pr <= F	0.9957
右侧 Pr >= F	0.0065
表概率 (P)	0.0023
双侧 Pr <= P	0.0113

样本大小 = 14060
频数缺失 = 86

频数<br>列百分比			

death_cvd-EXCLUDE表			
death_cvd(1年心血管源性死亡，0=NO;<br>1=YES)	EXCLUDE(1:有腰围数据；0: 腰围缺失)		
	0	1	合计
0	9209<br>98.59	4740<br>98.65	13949
1	132<br>1.41	65<br>1.35	197
合计	9341	4805	14146

categorical variables, descriptive by group and chisq test

FREQ 过程

表“EXCLUDE-death_cvd”的统计量

统计量	自由度	值	概率
卡方	1	0.0842	0.7717
似然比卡方检验	1	0.0846	0.7712
连续调整卡方	1	0.0460	0.8302
Mantel-Haenszel 卡方	1	0.0842	0.7717
Phi 系数		-0.0024	
列联系数		0.0024	
Cramer V		-0.0024	

Fisher 的精确检验	
单元格 (1,1) 频数 (F)	9209
左侧 Pr <= F	0.4181
右侧 Pr >= F	0.6402
表概率 (P)	0.0583
双侧 Pr <= P	0.8204

样本大小 = 14146

频数
列百分比

y1_comb-EXCLUDE表			
y1_comb(N12.Follow-up events at 12 months:Occurrence of combined vascular event(including cardiovascular death,non-fatal stroke,non-fatal myocardial infarction):0-No;1-Yes;)	EXCLUDE(1:有腰围数据；0：腰围缺失)		
	0	1	合计
0	8356<br>89.46	4285<br>89.18	12641
1	985<br>10.54	520<br>10.82	1505
合计	9341	4805	14146

categorical variables, descriptive by group and chisq test

FREQ 过程

表“EXCLUDE-y1_comb”的统计量

统计量	自由度	值	概率
卡方	1	0.2564	0.6126
似然比卡方检验	1	0.2557	0.6131
连续调整卡方	1	0.2280	0.6330
Mantel-Haenszel 卡方	1	0.2563	0.6127
Phi 系数		0.0043	
列联系数		0.0043	
Cramer V		0.0043	

Fisher 的精确检验	
单元格 (1,1) 频数 (F)	8356
左侧 Pr <= F	0.7043
右侧 Pr >= F	0.3158
表概率 (P)	0.0201
双侧 Pr <= P	0.6246

样本大小 = 14146

频数<br>列百分比

y1_death-EXCLUDE表			
y1_death(N12.Follow-up events at 12 months: Whether the patient died: 0-survival;1-death;)	EXCLUDE(1:有腰围数据；0: 腰围缺失)		
	0	1	合计
0	9045<br>96.83	4615<br>96.05	13660
1	296<br>3.17	190<br>3.95	486
合计	9341	4805	14146

categorical variables, descriptive by group and chisq test

FREQ 过程

表“EXCLUDE-y1_death”的统计量

统计量	自由度	值	概率
卡方	1	5.8993	0.0151
似然比卡方检验	1	5.7755	0.0163
连续调整卡方	1	5.6650	0.0173
Mantel-Haenszel 卡方	1	5.8989	0.0152
Phi 系数		0.0204	
列联系数		0.0204	
Cramer V		0.0204	

Fisher 的精确检验	
单元格 (1,1) 频数 (F)	9045
左侧 Pr <= F	0.9930
右侧 Pr >= F	0.0091
表概率 (P)	0.0021
双侧 Pr <= P	0.0169

样本大小 = 14146
